# Catalytic Asymmetric Cationic Geranyl Cyclizations

**DOI:** 10.1021/jacs.6c08353

**Published:** 2026-07-20

**Authors:** Na Luo, Mathias Turberg, Nobuya Tsuji, Markus Leutzsch, Sebastian Brunen, Benjamin List

**Affiliations:** † Max-Planck-Institut für Kohlenforschung, Mülheim an der Ruhr 45470, Germany; ‡ Institute for Chemical Reaction Design and Discovery, 12810 Hokkaido University, Sapporo 001-0021, Japan

## Abstract

Cyclogeranyl motifs are important in natural products and terpene synthesis, yet their asymmetric formation from acyclic dienes remains challenging. Here, we report a general catalytic asymmetric cyclization of geranyl derivatives using confined chiral imidodiphosphorimidate (IDPi) Brønsted acids. In combination with fluorinated alcohol additives, the reaction proceeds under mild conditions with high efficiency and excellent regio- and enantioselectivity. The method accommodates diverse substrates, enabling access to fragrance-relevant scaffolds, retinal precursors, complex polycyclic terpenes, and even selective squalene cyclization. Mechanistic studies support a cooperative catalyst–additive hydrogen-bonding network and an asynchronous concerted, enzyme-like transition state within a confined chiral environment. This work provides a general solution for asymmetric geranyl cyclizations and expands the utility of confined Brønsted acid catalysis in biomimetic terpene synthesis.

## Graphical abstract

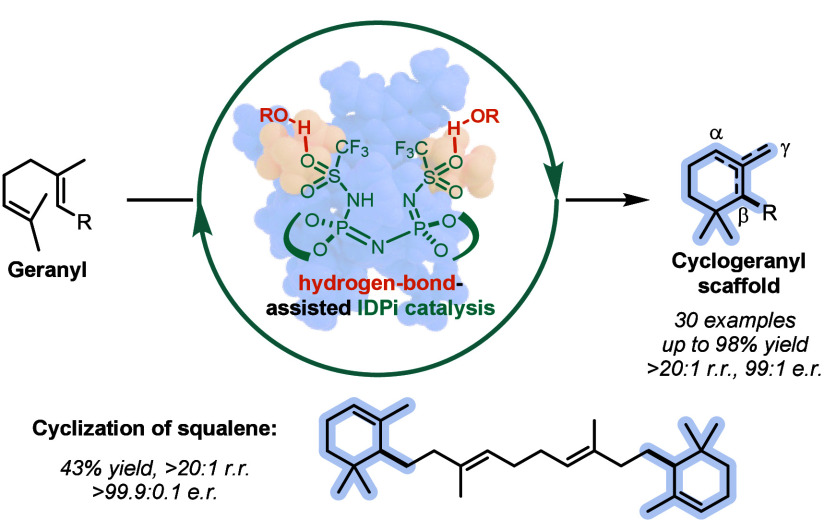

Terpenoids represent one of the most structurally diverse and complex classes of natural products, exhibiting a wide range of biological functions and applications.^1,2^ Central to terpene biosynthesis are enzyme-catalyzed polyene cyclizations, which convert linear isoprenoid precursors into complex polycyclic architectures with high stereochemical fidelity. These polycyclization cascades constitute a long-standing paradigm in terpene chemistry and have strongly inspired biomimetic and synthetic strategies, tracing back to the seminal contributions of Eschenmoser, Stork, and et al.^3–9^ In contrast, general catalytic methods for selective cyclogeranyl formation from simple acyclic precursors remain limited. While polycyclization represents a central and long-standing challenge in the field, selective monocyclization is an equally important yet comparatively less explored problem. The cyclogeranyl motif is present in numerous biologically relevant molecules, including ionone, damascone, ambrox, retinal derivatives, totarol, and related terpenoids (Figure 1A).^10–16^

**Figure 1:**
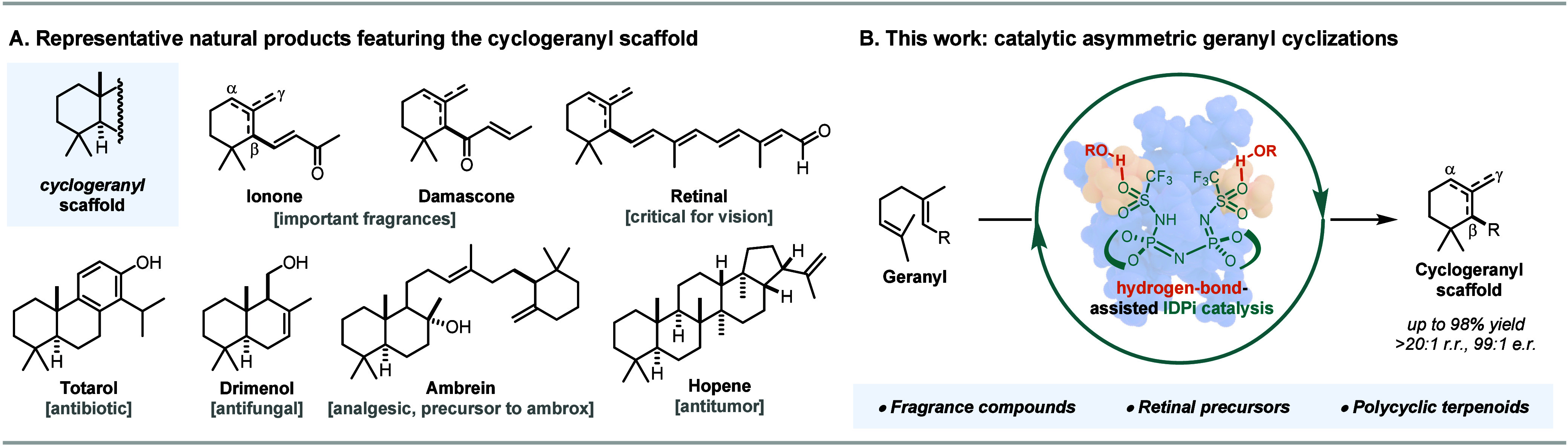
Representative bioactive terpenes and the present work. (A) Bioactive terpenes containing the cyclogeranyl scaffold. (B) This work: catalytic asymmetric cyclization of geranyl derivatives enabled by hydrogen-bonding assisted IDPi catalysis.

To date, relevant examples are largely limited to engineered squalene–hopene cyclases, catalyzing cation–olefin cyclizations, which are restricted to specific substrate types and cannot be generalized to access structurally diverse cyclogeranyl scaffolds.^17–20^ In the context of chemical synthesis, general geranyl cyclization remains highly challenging, requiring activation of electronically unbiased terminal olefins and precise stereochemical control of flexible polyene chains during C–C bond formation. Regioselectivity further complicates the process, while selective monocyclization of polyenes with more than two double bonds requires terminal protonation and deprotonation over competing downstream cyclization pathways.

We have recently developed strong and confined chiral imidodiphosphorimidate (IDPi) Brønsted acids, which exhibit the exceptional ability to convert unactivated olefins.^21–26^ In previous studies, these catalysts enabled highly enantioselective polyene cyclization cascades of acyclic precursors en route to the naturally occurring and industrially important odorant (−)-ambrox.^26^ Notably, during the construction of complex polycyclic frameworks, discrete monocyclic intermediates were observed, underscoring the potential of confined Brønsted acids to control stereochemistry at individual ring-forming steps.

Herein, we report a general asymmetric cyclization of geranyl derivatives enabled by IDPi acids and fluorinated alcohol additives (Figure 1B). The method affords cyclogeranyl products with excellent regio- and enantioselectivity through a biomimetic, enzyme-like folding of the substrate within the confined catalyst cavity, providing direct access to fragrance compounds, retinal precursors, polycyclic terpenes, and selectively cyclized squalene derivatives.

At the outset of our study, (*E*)-2,6-dimethylocta-2,6-diene **1a** was selected as a model substrate. Treatment with 2 mol % IDPi **5a** in toluene resulted in minimal conversion. Addition of fluorinated alcohols, recently exploited to stabilize carbocationic intermediates,^27–31^ markedly enhanced reactivity. In particular, perfluoro-*tert*-butanol (PFTB, 2 eq.) significantly improved reaction efficiency, affording predominantly the α-cyclized product **2a** in 92% yield and 78:22 e.r. (Table 1. entry 1). Among the IDPi catalyst library, 2-fluorenyl-substituted derivatives showed the most promising enantioselectivities and were therefore examined in detail. Progressing from **5a** to **5c** improved enantioselectivity from 78:22 to 93:7 e.r. while maintaining high yields (90–95%, entries 1–3). Further steric tuning with *tert*-butyl-substituted IDPi **5d** increased stereocontrol to 95:5 e.r. without compromising efficiency (entry 4), highlighting the importance of a confined catalyst architecture.

**Table 1:**
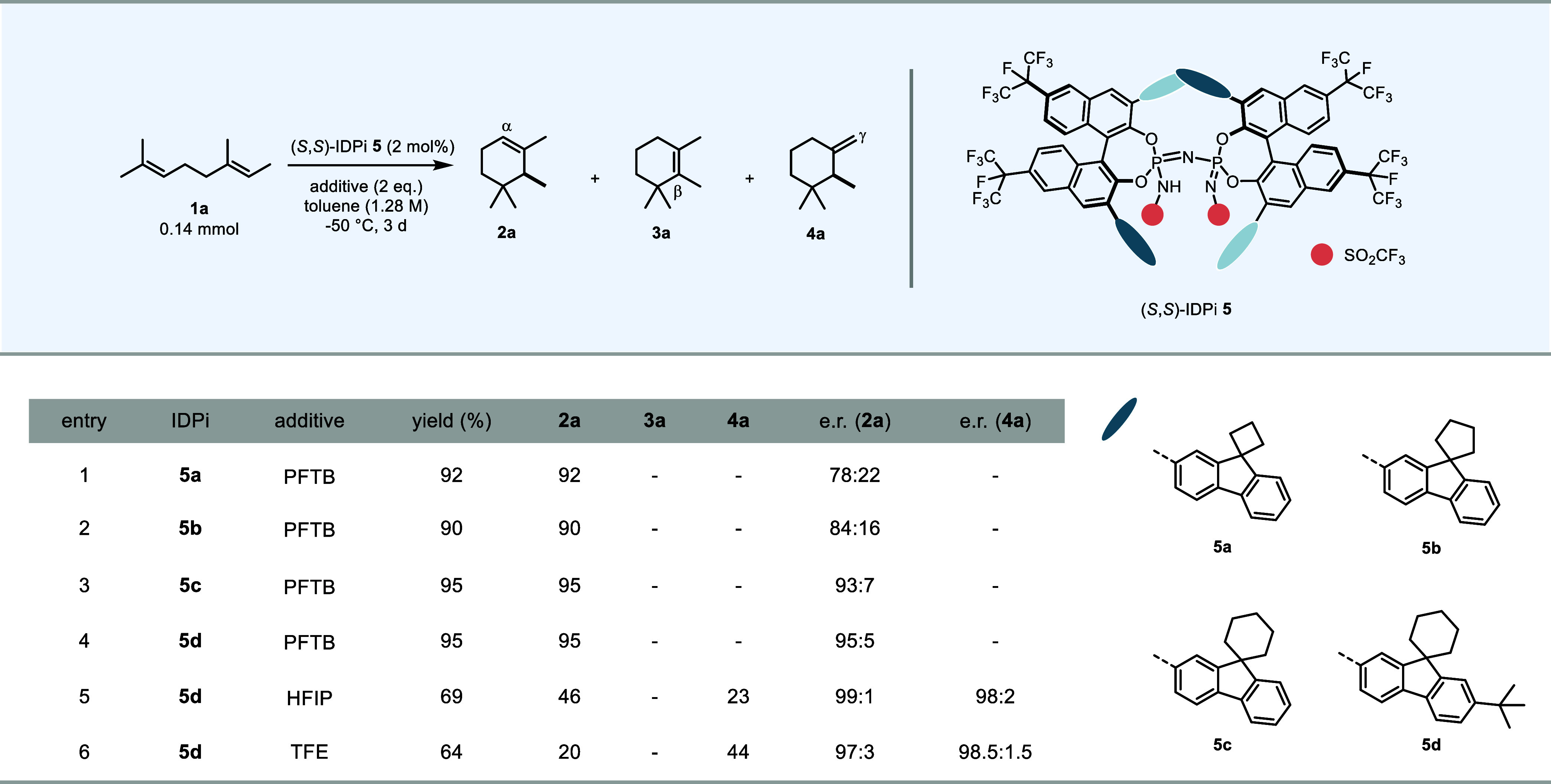
Catalyst Optimization

The influence of different fluorinated alcohol additives was then examined. Using 1,1,1,3,3,3-hexafluoropropan-2-ol (HFIP) led to high enantioselectivity but partial formation of the γ-cyclized **4a** (entry 5, **2a**:**4a** = 67:33), while 2,2,2-trifluoroethanol (TFE) further increased the ratio of **4a** (entry 6, **2a**:**4a** = 31:69). Importantly, both **2a** and **4a** were obtained with comparable enantioselectivities, indicating that enantiodiscrimination is dictated by the confined chiral IDPi framework, while the fluorinated alcohol additive primarily governs the yield and regioselectivity.

The reaction scope was evaluated with a range of alkyl- and aryl-substituted dienes (Figure 2A). For linear alkyl substrates **1b**–**1d**, increasing chain length slightly reduced regioselectivity, leading to higher proportions of γ-cyclized products. Nevertheless, high yields (85–98%) and excellent enantioselectivities were maintained for both α-products (up to 96.5:3.5 e.r.) and γ-products (up to 99:1 e.r.). Sterically demanding isopropyl (**1e**) and *tert*-butyl (**1f**) substituents were also well tolerated. Aryl-substituted dienes showed similarly broad compatibility. Phenyl substrate **1g** afforded **2g** in 88% yield and 97.5:2.5 e.r., while methyl-, alkoxy-, and halogen-substituted derivatives (**1h**–**1l**) were readily accommodated, although mild heating was required to promote isomerization. Benzyl- and phenethyl-substituted dienes furnished the corresponding products in 91% yield (**2m**, 86:14 e.r.) and 88% combined yield (**2n** + **4n**: **2n**, 89.5:10.5 e.r.; **4n**, 91:9 e.r.), respectively. Their longer reaction times likely reflect increased conformational flexibility and altered electronic properties, reducing effective preorganization within the confined catalyst environment. Substitution at C_5_ strongly affected the outcome: an ethyl substituent gave **2o** and **4o** in 88% yield (**2o**:**4o** = 72:28; **2o**: 90.5:9.5 e.r.), whereas a phenyl group diminished both yield and enantioselectivity (**2p**, 56%, 84:16 e.r.). Replacing the terminal dimethyl groups with cyclohexyl (**1q**) completely suppressed reactivity, likely due to steric congestion within the confined catalytic pocket. The absolute configurations of **2a** and **2f** were established by X-ray analysis of their osmate esters,^32^ and all other products were assigned by analogy.

**Figure 2:**
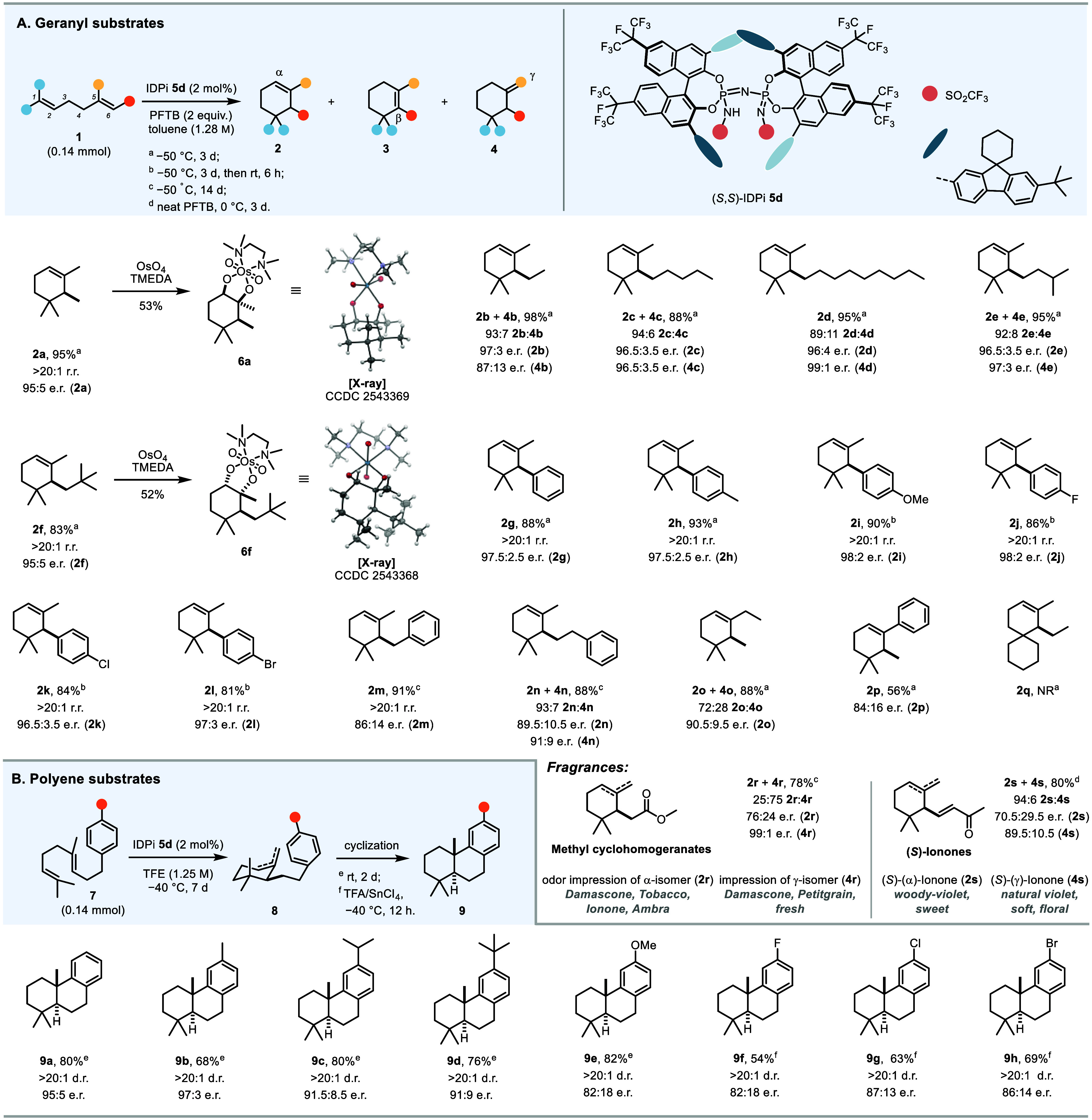
Substrate scope. (A) Geranyl substrates for monocyclization. (B) Polyene substrates for polycyclization to polycyclic terpenoids.

To showcase synthetic utility, aroma-relevant cyclohomogeranate esters and ionones were synthesized.^33,34^ Cyclization of homogeranate ester **1r** afforded **2r** and **4r** in 78% yield (**2r**:**4r** = 25:75) with good enantioselectivities (**2r**: 76:24 e.r.; **4r**: 99:1 e.r.). Notably, the IDPi catalyst favored γ-isomer **4r**, whereas previous methods typically produced α-isomer and β-isomer.^33^ Under neat PFTB at 0 °C, pseudoionone **1s** gave ionones in 80% yield (**2s**:**4s** = 94:6; **2s**: 70.5:29.5 e.r.; **4s**: 89.5:10.5 e.r.), underscoring the efficiency and selectivity of the method in constructing cyclogeranyl frameworks with important olfactory properties.

The strategy further enabled enantioselective polyene cyclizations to access polycyclic terpenoid frameworks (Figure 2B).^35–37^ Under optimized conditions, substrate **7** underwent enantioselective monocyclization to isolable intermediate **8**, followed by temperature or acid promoted intramolecular aryl addition to furnish tricyclic product **9** with excellent diastereoselectivity while preserving enantiomeric purity, indicating that stereochemical information is established in the initial cyclization and relayed throughout the cascade. Electron-neutral **7a** afforded **9a** in 80% yield (>20:1 d.r., 95:5 e.r.), while methyl-substituted **7b** gave **9b** in 68% yield and 97:3 e.r. Bulky *para*-substituents (**7c**, **7d**) slightly reduced enantioselectivity, and methoxy-substituted **7e** afforded **9e** in 82% yield and 82:18 e.r. Halogen-substituted substrates (**7f**–**7h**) were also tolerated, although slower secondary cyclization required additional promoters.

To gain insight into the reaction mechanism, cyclization of **1a** catalyzed by IDPi **5d** was monitored by time-resolved ^1^H NMR spectroscopy (Figure 3A). Both α- and γ-cyclized products (**2a** and **4a**) formed simultaneously at an early stage, followed by complete conversion of **4a** into the thermodynamically favored **2a**, indicating initial kinetic product formation and subsequent isomerization. Independent resubjection experiments confirmed that only **4a** converted to **2a** (see SI), identifying **4a** as a transient kinetic product, consistent with our previous study.^26^ Consumption of **1a** followed first-order kinetics, indicating involvement of a single substrate molecule in the rate-determining step.^25,38^

**Figure 3:**
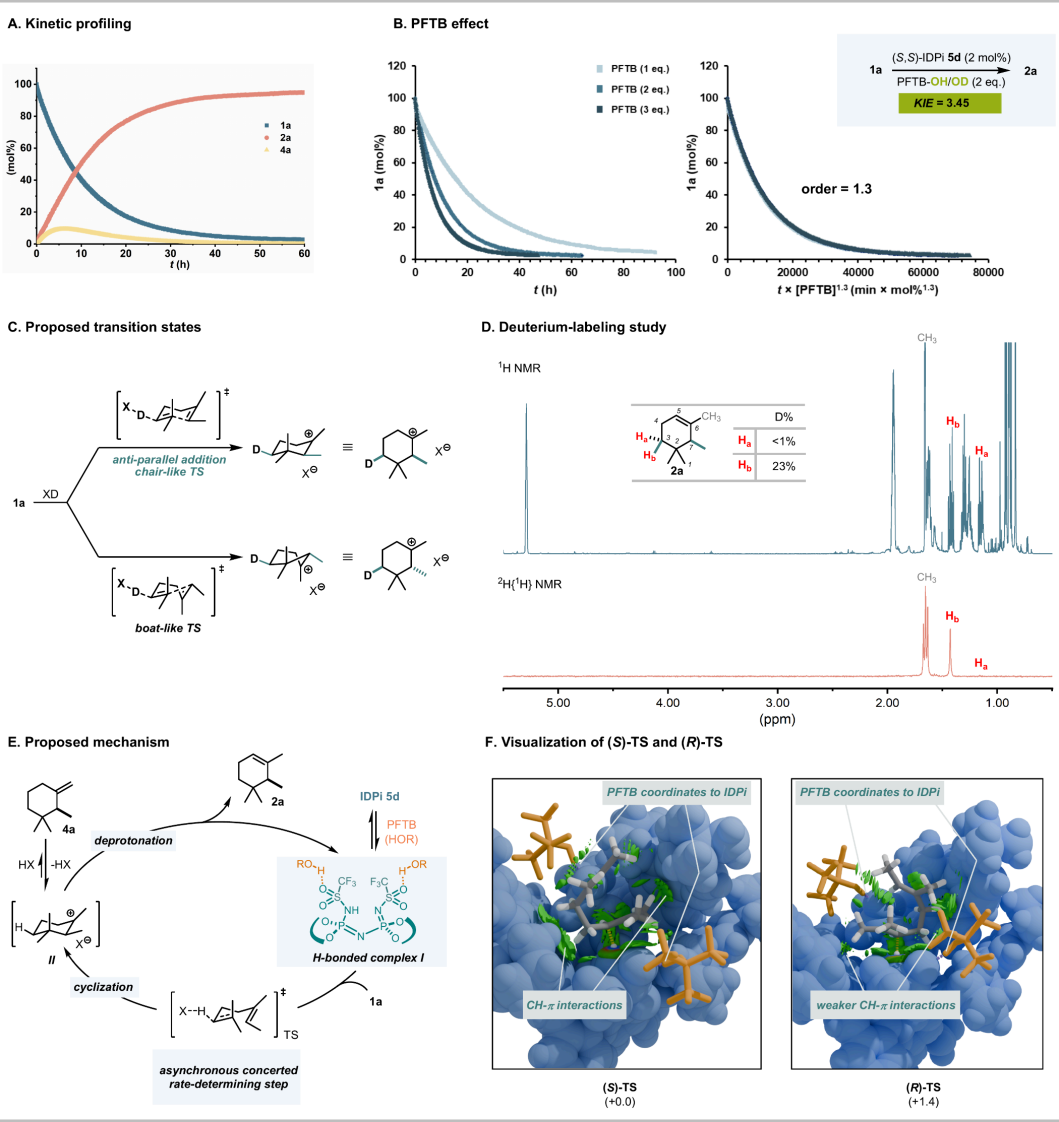
Mechanistic studies. (A) Reaction profiles monitored by ^1^H NMR spectroscopy. (B) Determination of PFTB order by VTNA analysis and KIE measurements using PFTB–OH/OD. (C) Proposed transition states for the acid-catalyzed (XD) geranyl cyclization. (D) Deuterium-labeling experiment with PFTB–OD, showing ^1^H and ^2^H­{^1^H} NMR spectra of product **2a**. (E) Plausible catalytic cycle. (F) Visualization of transition-state structures (*S*)-TS and (*R*)-TS. DFT calculations were performed at the SMD­(Toluene)-ωB97M-V/def2-TZVPP//r^2^SCAN-3c level of theory (Gibbs free energies in kcal mol^–1^). The isosurface in the IGMH map corresponds to δG^inter^ of 0.004 au.

To clarify the role of additive PFTB, low-temperature NMR titrations were performed, motivated by the known ability of fluorinated alcohols to form catalytically active hydrogen-bonding networks.^39^ Incremental addition of PFTB to a solution of IDPi caused subtle aromatic chemical-shift changes and pronounced *tert*-butyl signal splitting, with analogous effects in the ^31^P NMR spectra, indicating formation of a defined catalyst–additive complex (see SI). Variable time normalization analysis (VTNA) revealed a fractional reaction order of 1.3 in PFTB (Figure 3B),^40,41^ indicating a cooperative involvement of PFTB in the catalytic process. On the basis of these observations, we propose that PFTB forms a hydrogen-bonding network with the IDPi catalyst, thereby likely enhancing its effective Brønsted acidity.^42^

Furthermore, a kinetic isotope effect of 3.45 was observed using PFTB–OH/OD. Although consistent with exchangeable protons within the catalytic assembly,^27,43^ DFT calculations (see Supporting Information (SI)) suggest that the effect arises primarily from H/D exchange and catalyst-related isotope effects rather than direct proton transfer from PFTB in the rate-determining step.

Analysis of products obtained in PFTB–OD by ^1^H and ^2^H­{^1^H} NMR revealed deuterium incorporation at the C_3_-equatorial position H_b_ (Figure 3D). The observed stereochemical outcome can be rationalized in the context of a concerted (potentially asynchronous) cyclization model,^26,44,45^ as commonly proposed for acid-catalyzed terpene cyclizations proceeding through preorganized chairlike transition states.^45^ The newly formed C_7_ stereocenter displays a syn relationship between H_b_ and the methyl group, reflecting antiparallel addition of the electrophilic and nucleophilic termini across the olefin (Figure 3C).

Further mechanistic insight was obtained from β-secondary deuterium kinetic isotope effect experiments. Comparison of the initial rates of **1a** and (*E*)-6-methyl-2-(methyl-*d*_3_)­octa-2,6-diene-1,1,1,3-*d*_4_ (**1a**-*d*_*7*_) gave a normal isotope effect (k_H_/k_D_ = 1.30). Together with the more complex behavior observed for C_3_-monodeuterated substrate **1a**-*d*_*1*_ (see SI for details), these results implicate both C_2_ and C_3_ in the rate-determining step and are consistent with π-system polarization in the cyclization transition state.

To gain insight into the reaction mechanism and enantioselectivity, density functional theory (DFT) calculations were performed on **1a** and **5d** using a model containing two explicit PFTB molecules at the SMD­(Toluene)-wB97M-V/def2-TZVPP//r^2^SCAN-3c level of theory (see SI). The 2:1 PFTB:IDPi stoichiometry was selected to represent cooperative PFTB participation, consistent with the positive fractional order determined by VTNA (Figure 3B). In the optimized structure, two PFTB molecules coordinate to the sulfonyl oxygens of **5d**, forming a hydrogen-bonded complex (**I**) that stabilizes the chiral counteranion, enhances acidity, and increases steric confinement (Figure 3F). For both enantiomers, protonation of **1a** occurs from a prefolded state and is followed by spontaneous cyclization to tertiary carbocation II, supporting a concerted protonation–cyclization pathway with asynchronous bond formation (Figure 3E).

This model also accounts for the observed enantioselectivity: (*S*)-TS leading to major product (*S*)-**2a** is energetically favored over (*R*)-TS (ΔΔG^‡^ = 1.4 kcal mol^–1^, e.r.(calc.) = 96:4). To further elucidate the origin of enantioselectivity, a distortion-interaction analysis following the Houk–Bickelhaupt protocol was performed,^46^ suggesting that substrate cation–counteranion interactions are the predominant factor controlling enantioselectivity. To evaluate these key interactions, noncovalent interactions were visualized using the Independent Gradient Model based on Hirshfeld partition (IGMH).^47–49^ While prominent C–H···π interactions between polarized substrate C–H bonds and fluorenyl substituents are observed in (*S*)-TS,^25,50^ these interactions are much weaker in the (*R*)-TS, presumably due to an expanded active site required to accommodate the substrate, resulting in a higher TS energy.

Given the enzyme-like recognition of prefolded geranyl fragments, increasingly complex polyenes were investigated (Figure 4). Triene **10a** underwent selective monocyclization to afford **11a** in 51% yield and 96:4 e.r., with no detectable γ-cyclized product. This likely reflects rapid downstream bicyclization of the γ-isomer and is consistent with our previous mechanistic studies.^26,51^ More flexible substrate **10b**, (2*E*,4*E*,6*E*,10*E*)-didehydrogeranylgeranial, underwent selective monocyclization to provide vitamin A precursors.^52–54^ Initial cyclization furnished a 1:1 mixture of α- and γ-products in 60% yield with excellent enantioselectivities (**11b**: >99.9:0.1 e.r.; **12b**: 98:2 e.r.). Subsequent mild heating partially converted the γ-isomer into the α-isomer without erosion of enantiopurity. Remarkably, squalene **10c**, a highly flexible linear triterpene bearing multiple olefins, underwent selective terminal cyclization to furnish **11c** in 43% yield with excellent regio- and enantioselectivity (>20:1 r.r.; >99.9:0.1 e.r.).

**Figure 4:**
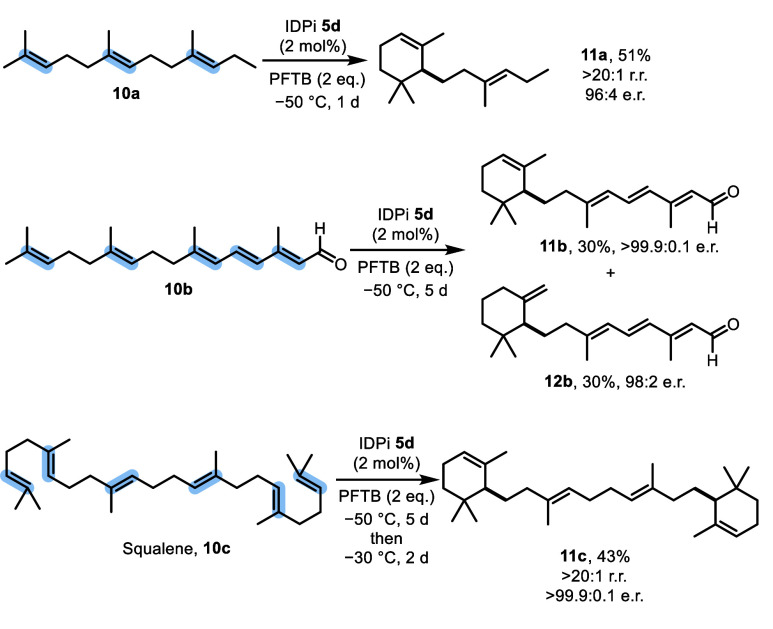
Cyclization of polyenes with multiple olefinic sites. Reactions were performed on a 0.14 mmol scale with IDPi **5d** (2 mol %) and PFTB (2 eq.).

In summary, we developed a general asymmetric cyclization of geranyl derivatives, providing access to valuable cyclogeranyl scaffolds. Mechanistic studies support a cooperative IDPi and PFTB hydrogen-bonding network operating through a confined, enzyme-like transition state. The method delivers high regio- and enantioselectivity across simple and complex polyenes, highlighting its robustness and utility for terpenoid synthesis.

## Supplementary Material



## Notes

Experimental details and analytical data for all new compounds, NMR spectra, and computational studies (PDF)
