# Supplementary material for: Catalytic Asymmetric Cationic Geranyl Cyclizations
Source: J Am Chem Soc. 2026 Jul 20;148(30):31649–55. doi: 10.1021/jacs.6c08353 (PMC13449958; doi:10.1021/jacs.6c08353)
Supplement: Supplementary file 1 [file ja6c08353_si_001.pdf]

Supplementary Information

Catalytic Asymmetric Cationic Geranyl Cyclizations

Na Luo,[a] Mathias Turberg,[a] Nobuya Tsuji,[b] Markus Leutzsch,[a] Sebastian Brunen,[a] Benjamin List[a,b*]

[a] Max-Planck-Institut für Kohlenforschung, Mülheim an der Ruhr 45470, Germany
[b] Institute for Chemical Reaction Design and Discovery, Hokkaido University, Sapporo 001-0021, Japan

*e-mail: list@kofo.mpg.de

Table of Contents

1. General information and instrumentation 2
2. Synthesis and characterization of substrates 4
 2.1 Synthesis of geranyl substrates **1a**–**1s** 4
 2.2 Synthesis of polyene substrates **7a**–**7h** 15
 2.3 Synthesis of polyene substrates **10a**–**10c** 19
3. Procedure for reaction development 19
 3.1 Development of the catalytic asymmetric geranyl cyclization 19
 3.2 Development of the catalytic asymmetric polyene cyclization 21
4. Catalytic asymmetric geranyl cyclization 23
5 Catalytic asymmetric polyene cyclization to polycyclic terpenoids 31
6. Cyclization of polyenes with multiple olefinic sites 36
7. Synthesis of Imidodiphosphorimidate IDPi catalysts 39
8. Determination of the absolute configuration of products 41
9. Mechanistic studies 43
 9.1 Kinetic profiling 43
 9.2 PFTB effect 47
 9.3 Deuterium Labeling study 51
 9.4 β-Secondary deuterium kinetic isotope effect (β-DKIE) 55
10. GC and HPLC traces of the products 64
 10.1. Copies of GC traces 64
 10.2. Copies of HPLC traces 80
11. ^{1}H, ^{13}C, ^{31}P and ^{19}F NMR spectra of substrates and products 102
12. Crystallographic data 184
13. Computational Studies 193
 13.1 Method 193
 13.2 Results and Discussion 193
 13.3 Cartesian coordinates of the optimized structure 195
References 304

1. General information and instrumentation

Chemicals: Unless otherwise indicated, starting materials were obtained from Sigma-Aldrich, ABCR-GmbH, TCI. Commercially available reagents were used without additional purification.

Solvents: Solvents (pentane, *n*-hexane, Et_2O, THF, acetone, CH_2Cl_2, $CHCl_3$ and toluene) were dried by distillation from an appropriate drying agent in the technical department of the Max-Planck-Institut für Kohlenforschung and received in Schlenk flasks under argon. In addition, more solvents (EtOAc, ^{i}PrOH and MeCN) were purchased from commercial suppliers and dried over molecular sieves.

Inert Gas: Dry argon was purchased from Air Liquide with >99.5% purity.

Thin layer chromatography: Thin layer chromatography (TLC) was performed using silica gel pre-coated plastic sheets (Polygram SIL G/UV254, 0.2 mm, with fluorescent indicator; Macherey-Nagel) which was visualized with a UV lamp (254 nm) and/or phosphomolybdic acid (PMA). PMA stain: PMA (20 g) in EtOH (200 mL).

Column chromatography: Column chromatography was carried out using Merck silica gel (60 Å, 230–400 mesh, particle size 0.040–0.063 mm) using technical grade solvents. Elution was accelerated using compressed argon. All reported yields, unless otherwise specified, refer to spectroscopically and chromatographically pure compounds.

Nuclear magnetic resonance spectroscopy: ^{1}H, ^{2}H, ^{13}C, ^{19}F, ^{31}P nuclear magnetic resonance (NMR) spectra were acquired on Bruker Avance™ NEO 600 MHz (equipped with a BBO CryoProbe), Avance™ III 600 MHz (equipped with a TCI CryoProbe), or Avance™ III 500 MHz in a suitable deuterated solvent. The respective measuring frequencies and utilized solvents are specified in the individual experiment. ^{1}H and ^{2}H chemical shifts are reported in ppm (δ) relative to tetramethylsilane (TMS) with the residual solvent resonance serving as the internal reference (δ 7.26 ppm for $CDCl_3$, δ 2.09 ppm for toluene-d_8). Data are provided as follows: chemical shift in ppm, resonance multiplicity (s = singlet, d = doublet, t = triplet, q = quartet, p = pentet, s = sextet, h = heptet, m = multiplet, br = broad), coupling constant J in Hz and integration/number of protons. ^{13}C chemical shifts are reported in ppm from tetramethylsilane (TMS) with the solvent resonance as the internal standard ($CDCl_3$ δ 77.16 ppm, δ 20.40 ppm for toluene-d_8). ^{13}C, ^{19}F, ^{31}P NMR spectra were referenced according to the respective Ξ-values (following the IUPAC recommendations from 2008) relative to the internal references set in ^{1}H NMR spectra. Heteronuclear NMR spectra (^{2}H, ^{13}C, ^{19}F, ^{31}P) were generally acquired with broadband proton decoupling unless noted otherwise.

Mass spectrometry: Electrospray ionization (ESI) mass spectrometry was conducted on a Bruker ESQ 3000 spectrometer. High resolution mass spectrometry (HRMS) was performed on a Finnigan MAT 95 (EI) or Bruker APEX III FTMS (7 T magnet, ESI). The ionization method and mode of detection employed is indicated for the respective experiment. The ionization method and mode of detection employed is indicated for the respective experiment and all masses are reported in atomic units per elementary charge (*m*/*z*) with an intensity normalized to the most intense peak.

Specific rotations: Specific rotations were measured with a Rudolph RA Autopol IV Automatic Polarimeter at the indicated temperature with a sodium lamp (sodium D line, λ = 589 nm). Measurements were performed in an acid resistant 1 mL cell (50 mm length) with concentrations (g/(100 mL)) reported in the corresponding solvent.

Gas chromatography (GC): Gas Chromatography was performed on HP 6890 and 5890 Series instruments equipped with a split-mode capillary injection system and a flame ionization detector (FID) using hydrogen (H_2) as carrier gas. Enantiomeric ratios were determined by comparing the sample with an appropriate corresponding racemic mixture. The (chiral) stationary phases, mobile phases and detailed conditions are provided in the individual experiment.

High performance liquid chromatography: High performance liquid chromatography (HPLC) was performed on a Shimadzu LC-20AD liquid chromatograph SIL-20AC auto sampler, CMB-20A using Daicel/Merck columns with a chiral stationary phase. All solvents used were HPLC-grade solvents purchased from Sigma-Aldrich. The column employed and the respective solvent mixture are indicated for each experiment.

X-ray crystallography: Single crystals suitable for X-ray diffraction were grown as specified in the respective experiment. X-ray crystal structure analyses were performed on a Bruker AXS Enraf-Nonius KappaCCD diffractometer with a FR591 rotating Mo-anode X-ray source Incoatec Helios focusing multilayer optics Bruker-AXS Kappa Mach3 with APEX-II detector and FR591 rotating anode X-ray source with Incoatec Helios mirrors Bruker-AXS Kappa Mach3 with APEX-II detector and lμS microfocus Mo-anode X-ray source and Incoatec Helios mirrors. Data were faceindexed absorption corrected and scaled using the program SADABS (Bruker AXS, 2014). The structure was refined using the programs SHELXS and SHELXL, both programs from G. M. Sheldrick or OLEX29 and NoSpherA210 routine. The X-ray crystal structure analyses were performed in the X-ray department of the Max-Planck-Institut für Kohlenforschung. Crystal structures were visualized and rendered using the program Mercury using version 4.2.0 developed by The Cambridge Crystallographic Data Centre (CCDC).

Nomenclature: Nomenclature follows the suggestions proposed by the computer program ChemBioDraw (12.0.3.1216) of CBD/cambridgesoft.

Abbreviations: e.r. = enantiomeric ratio, r.r. = regioisomeric ratio, TLC = thin layer chromatography, PFTB = perfluoro-*tert*-butanol, HFIP = hexafluoroisopropanol, TFE = 2,2,2-trifluoroethanol, THF = tetrahydrofuran, MeCN = acetonitrile, Tf = SO_2CF_3, MOM = methoxymethyl ether.

2. Synthesis and characterization of substrates

2.1 Synthesis of geranyl substrates 1a–1s

1a, and **1r** were synthesized according to literature procedures.[1–3] **1s** is commercially available pseudoionone**.** The synthesis of the other substrates is described as follows:

General procedure 1 (GP 1):

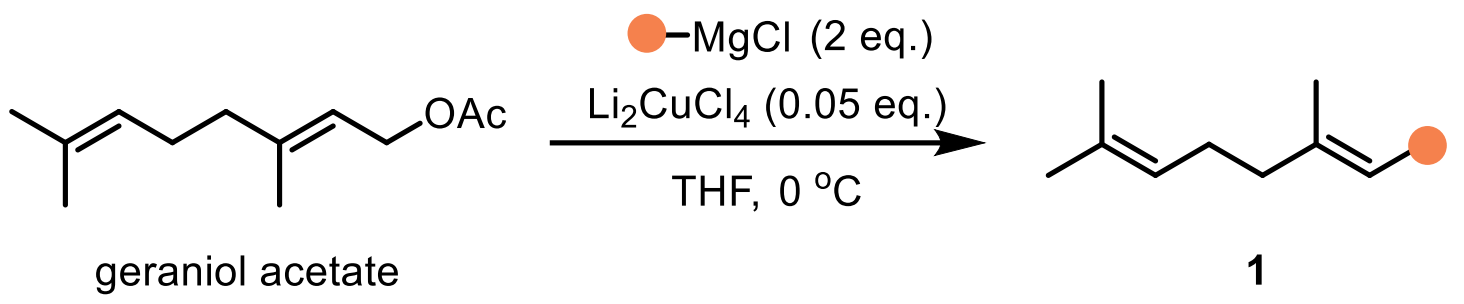

A flame-dried flask under argon was charged with geraniol acetate (5.0 mmol, 1.0 eq.) and dry THF (0.5 M). 0.1 M Li_2CuCl_4 (0.05 eq) solution in THF was added dropwise, then a solution of Grignard reagent (2 eq.) was added dropwise at 0 °C. After 2 h at 0 °C, the mixture was quenched with saturated aqueous NH_4Cl solution and the aqueous layer extracted with diethyl ether. Combined organic layers were washed with brine, dried over Na_2SO_4, and concentrated under reduced pressure. The residue was purified by silica gel column chromatography (pentane) to afford the coupling product.

General procedure 2 (GP 2):

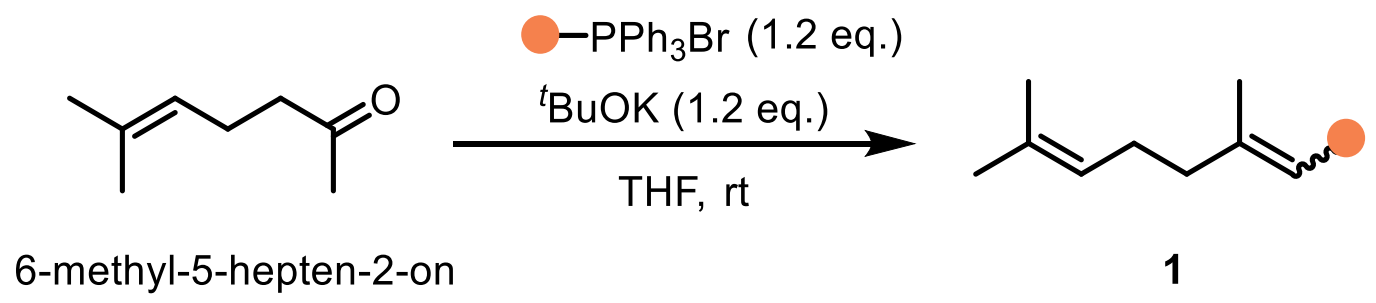

To a suspension of ^{t}BuOK (6.0 mmol, 1.2 eq.) in anhydrous THF (20 mL) was added $RPPh_3Br$ (6.0 mmol, 1.2 eq.) under argon atmosphere. The suspension was stirred at room temperature for 1 h. Then 6-methyl-5-hepten-2-on (5.0 mmol, 1.0 eq.) was added and the reaction mixture was stirred at the same temperature for 1 h. Then the mixture was filtered through a short pad of silica gel, which was subsequently washed with diethyl ether (50 mL). After evaporation of the organic solvent, the residue was purified by silica gel column chromatography to provide diene.

(*E*)-2,6-dimethylocta-2,6-diene 1a

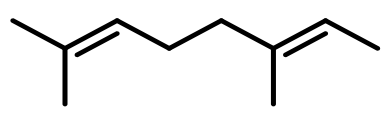

1a

Prepared according to literature[1], colorless oil.

***R*$_F$** (pentane) = 0.9.

^{1}H NMR (501 MHz, $CDCl_3$): δ = 5.22–5.20 (m, 1H), 5.12–5.09 (m, 1H), 2.09–2.05 (m, 2H), 2.00–1.96 (m, 2H), 1.69 (s, 3H), 1.60 (s, 6H), 1.58 (d, J = 6.6 Hz, 3H).

^{13}C NMR (126 MHz, $CDCl_3$): δ = 136.0, 131.5, 124.6, 118.4, 39.9, 26.9, 25.8, 17.8, 15.8, 13.5.

(*E*)-2,6-dimethylnona-2,6-diene 1b

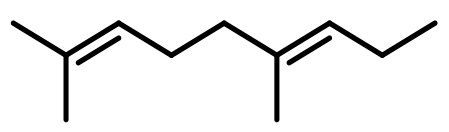

1b

Prepared according to **GP 1**, stirred for 2 h, eluent: pentane, 600 mg (80%), colorless oil.

R_F (pentane) = 0.9.

^{1}H NMR (501 MHz, $CDCl_3$): δ = 5.14–5.09 (m, 2H), 2.08–2.05 (m, 2H), 2.01–1.96 (m, 4H), 1.68 (s, 3H), 1.61 (s, 3H), 1.60 (s, 3H), 0.95 (t, J = 7.5 Hz, 3H).

^{13}C NMR (126 MHz, $CDCl_3$): δ = 134.5, 131.4, 126.6, 124.6, 39.8, 26.9, 25.8, 27.3, 17.8, 16.0, 14.5.

HRMS *m*/*z* (GC-EI): calculated for $C_{11}H_{20}^+$ ($[M]^+$): 152.15595; found: 152.15611.

(*E*)-2,6-dimethyldodeca-2,6-diene 1c

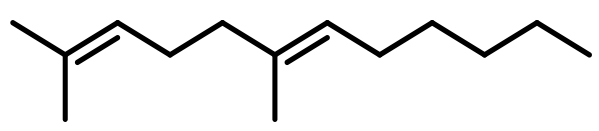

1c

Prepared according to **GP 1**, stirred for 2 h, eluent: pentane, 708 mg (73%), colorless oil.

R_F (pentane) = 0.9.

^{1}H NMR (501 MHz, $CDCl_3$): δ = 5.14–5.08 (m, 2H), 2.09–2.05 (m, 2H), 2.00–1.95 (m, 4H), 1.68 (s, 3H), 1.60 (s, 3H), 1.59 (s, 3H), 1.35–1.25 (m, 6H), 0.90 (t, J = 6.8 Hz, 3H).

^{13}C NMR (126 MHz, $CDCl_3$): δ = 134.9, 131.4, 125.0, 124.6, 39.9, 31.7, 29.7, 28.0, 26.9, 25.8, 22.8, 17.8, 16.1, 14.3.

HRMS *m*/*z* (GC-EI): calculated for $C_{14}H_{26}^+$ ($[M]^+$): 194.20290; found: 194.20300.

(*E*)-2,6-dimethylhexadeca-2,6-diene 1d

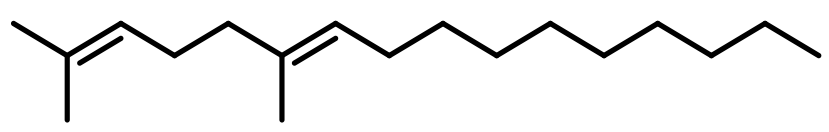

1d

Prepared according to **GP 1**, stirred for 2 h, eluent: pentane, 1020 mg (81%), colorless oil.

R_F (pentane) = 0.9.

^{1}H NMR (501 MHz, $CDCl_3$): δ = 5.14–5.08 (m, 2H), 2.09–2.05 (m, 2H), 2.00–1.96 (m, 4H), 1.68 (s, 3H), 1.60 (s, 3H), 1.59 (s, 3H), 1.31–1.26 (m, 14H), 0.89 (t, J = 6.8 Hz, 3H).

^{13}C NMR (126 MHz, $CDCl_3$): δ = 134.9, 131.4, 125.0, 124.6, 39.9, 32.1, 30.0, 29.84, 29.83, 29.53, 29.52, 28.1, 26.9, 25.8, 22.8, 17.8, 16.1.

HRMS *m*/*z* (GC-EI): calculated for $C_{18}H_{34}^+$ ($[M]^+$): 250.26550; found: 250.26546.

(*E*)-2,6,10-trimethylundeca-2,6-diene 1e

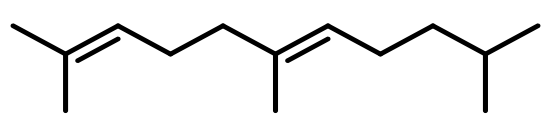

1e

Prepared according to **GP 1**, stirred for 2 h, eluent: pentane, 853 mg (88%), colorless oil.

R_F (pentane) = 0.9.

^{1}H NMR (501 MHz, $CDCl_3$): δ = 5.13–5.08 (m, 2H), 2.09–2.05 (m, 2H), 2.00–1.96 (m, 4H), 1.68 (s, 3H), 1.60 (s, 6H), 1.33–1.25 (m, 1H), 1.24–1.19 (m, 2H), 0.89 (d, *J* = 6.6 Hz, 6H).

^{13}C NMR (126 MHz, $CDCl_3$): δ = 134.7, 131.4, 125.1, 124.6, 39.9, 39.2, 27.7, 26.9, 25.9, 25.8, 22.7, 17.8, 16.1, 14.2.

HRMS *m*/*z* (GC-EI): calculated for $C_{14}H_{26}^+$ ($[M]^+$): 194.20290; found: 194.20281.

(*E*)-2,6,9,9-tetramethyldeca-2,6-diene 1f

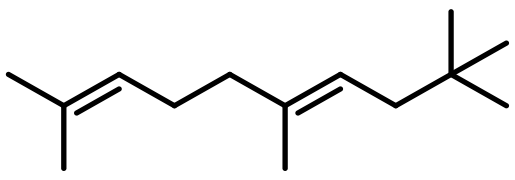

1f

Prepared according to **GP 1**, stirred for 2 h, eluent: pentane, 822 mg (85%), colorless oil.

R$_F$ (pentane) = 0.9.

^{1}H NMR (501 MHz, $CDCl_3$): δ = 5.23–5.19 (m, 1H), 5.12–5.08 (m, 1H), 2.12–2.07 (m, 2H), 2.04–2.01 (m, 2H), 1.88 (d, *J* = 7.7 Hz, 2H), 1.68 (s, 3H), 1.61 (s, 3H), 1.59 (s, 3H), 0.87 (s, 9H).

^{13}C NMR (126 MHz, $CDCl_3$): δ = 136.2, 131.4, 124.7, 122.1, 42.0, 40.3, 31.9, 29.4, 26.9, 25.9, 17.8, 16.2.

HRMS *m*/*z* (GC-EI): calculated for $C_{14}H_{26}^+$ ($[M]^+$): 194.20290; found: 194.20290.

(2,6-dimethylhepta-1,5-dien-1-yl)benzene 1g

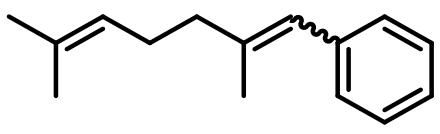

1g

Prepared according to **GP 2**, eluent: pentane, 847 mg (84%), *Z*:*E* = 39:61, The mixture was further separated by preparative HPLC (250 mm Chiralpak OJ-H, 20 mm i.d. 5 µm, MeOH/H_2O 90.0:10.0 v/v, 20.0 mL/min, 16.1 MPa, 308 K, 205 nm, UV) to furnish pure *Z* and *E* diene.

(*E*)-(2,6-dimethylhepta-1,5-dien-1-yl)benzene (*E*)-1g

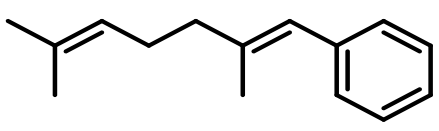

(*E*)-**1g**

colorless oil, ***R$_F$*** (pentane) = 0.8.

^{1}H NMR (501 MHz, $CDCl_3$): δ = 7.33–7.30 (m, 2H), 7.25–7.23 (m, 2H), 7.19–7.17 (m, 1H), 6.28 (s, 1H), 5.19–5.16 (m, 1H), 2.21–2.20 (m, 4H), 1.87 (d, *J* = 1.4 Hz, 3H), 1.71 (s, 3H), 1.64 (s, 3H).

^{13}C NMR (126 MHz, $CDCl_3$): δ = 139.1, 138.8, 131.9, 129.0, 128.1, 125.9, 125.1, 124.1, 40.9, 26.9, 25.9, 18.0, 17.9.

HRMS *m*/*z* (GC-EI): calculated for $C_{15}H_{20}^+$ ($[M]^+$): 200.15595; found: 200.15590.

(*Z*)-(2,6-dimethylhepta-1,5-dien-1-yl)benzene (*Z*)-1g

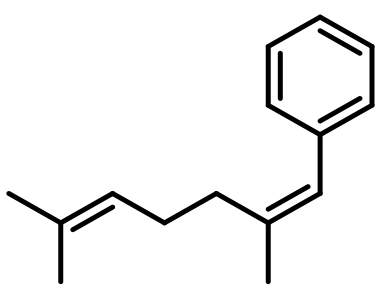

(*Z*)-**1g**

colorless oil, ***R$_F$*** (pentane) = 0.8.

^{1}H NMR (501 MHz, $CDCl_3$): δ = 7.32–7.29 (m, 2H), 7.22–7.18 (m, 3H), 6.30 (s, 1H), 5.14–5.11 (m, 1H), 2.28-2.24 (m, 2H), 2.21–2.17 (m, 2H), 1.91 (d, *J* = 1.5 Hz, 3H), 1.67 (s, 3H), 1.62 (s, 3H).

^{13}C NMR (126 MHz, $CDCl_3$): δ = 139.4, 138.7, 132.0, 128.7, 128.2, 126.0, 125.9, 125.8, 124.2, 124.1, 32.8, 26.8, 25.8, 24.3, 17.8.

HRMS *m*/*z* (GC-EI): calculated for $C_{15}H_{20}^{+}$ ($[M]^{+}$): 200.15595; found: 200.15605.

1-(2,6-dimethylhepta-1,5-dien-1-yl)-4-methylbenzene 1h

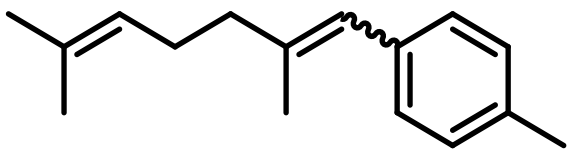

1h

Prepared according to **GP 2**, eluent: pentane, 892 mg (83%), *Z*:*E* = 39:61, The mixture was further separated by preparative HPLC (250 mm Chiralpak OJ-H, 20 mm i.d. 5 µm, MeOH/H_2O 95.0:5.0 v/v, 20.0 mL/min, 16.1 MPa, 308 K, 205 nm, UV) to furnish pure *Z* and *E* diene.

(*E*)-1-(2,6-dimethylhepta-1,5-dien-1-yl)-4-methylbenzene (*E*)-1h

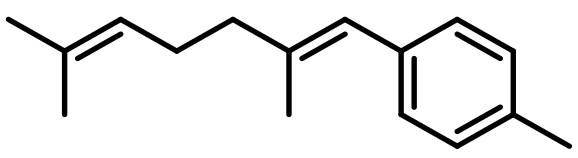

(*E*)-**1h**

colorless oil, ***R***R_F (pentane) = 0.8.

^{1}H NMR (501 MHz, $CDCl_3$): δ = 7.26–7.12 (m, 4H), 6.26 (s, 1H), 5.18–5.16 (m, 1H), 2.34 (s, 3H), 2.21–2.17 (m, 4H), 1.86 (s, 3H), 1.71 (s, 3H), 1.64 (s, 3H).

^{13}C NMR (126 MHz, $CDCl_3$): δ = 138.4, 135.9, 135.5, 131.8, 128.9, 124.9, 124.2, 40.9, 26.9, 25.9, 21.3, 18.0, 17.9.

HRMS *m*/*z* (GC-EI): calculated for $C_{16}H_{22}^{+}$ ($[M]^{+}$): 214.17160; found: 214.17176.

(*Z*)-1-(2,6-dimethylhepta-1,5-dien-1-yl)-4-methylbenzene (*Z*)-1h

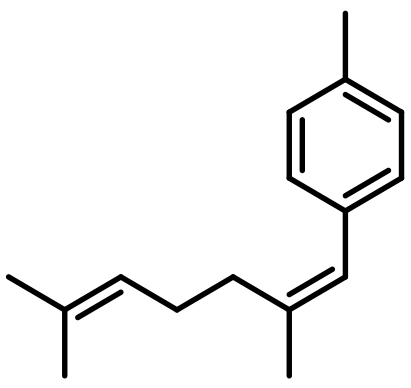

(*Z*)-**1h**

colorless oil, R_F (pentane) = 0.8.

^{1}H NMR (501 MHz, $CDCl_3$): δ = 7.12–7.09 (m, 4H), 6.25 (s, 1H), 5.14–5.10 (m, 1H), 2.33 (s, 3H), 2.26–2.15 (m, 4H), 1.88 (d, *J* = 1.5 Hz, 3H), 1.68 (s, 3H), 1.62 (s, 3H).

^{13}C NMR (126 MHz, $CDCl_3$): δ = 138.8, 135.8, 135.5, 131.9, 128.9, 128.6, 125.7, 124.2, 32.8, 26.8, 25.9, 24.3, 24.2, 17.8.

HRMS *m*/*z* (GC-EI): calculated for $C_{16}H_{22}^{+}$ ($[M]^{+}$): 214.17160; found: 214.17133.

1-(2,6-dimethylhepta-1,5-dien-1-yl)-4-methoxybenzene 1i

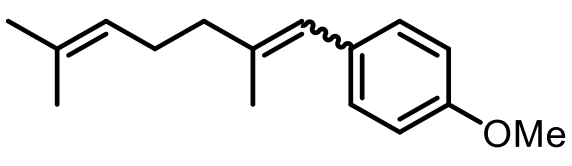

1i

Prepared according to **GP 2**, eluent: pentane/Et_2O = 20:1, 700 mg (61%), *Z*:*E* = 50:50, The mixture was further separated by preparative HPLC (250 mm Chiralpak OJ-H, 20 mm i.d. 5 µm, MeOH/H_2O 95.0:5.0 v/v, 20.0 mL/min, 16.1 MPa, 308 K, 205 nm, UV) to furnish pure *Z* and *E* diene.

(*E*)-1-(2,6-dimethylhepta-1,5-dien-1-yl)-4-methoxybenzene (*E*)-1i

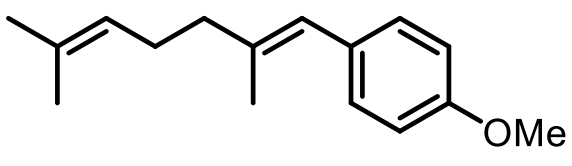

(*E*)-**1i**

colorless oil, R_F (pentane/Et_2O = 20:1) = 0.8.

^{1}H NMR (501 MHz, $CDCl_3$): δ = 7.18 (d, *J* = 8.7 Hz, 2H), 6.87 (d, *J* = 8.7 Hz, 2H), 6.21 (s, 1H), 5.18-5.15 (m, 1H), 3.81 (s, 3H), 2.20–2.15 (m, 4H), 1.852 (d, *J* = 1.4 Hz, 3H), 1.71 (s, 3H), 1.64 (s, 3H).

^{13}C NMR (126 MHz, $CDCl_3$): δ = 157.8, 137.6, 131.8, 131.5, 130.0, 124.4, 124.2, 113.6, 55.4, 40.9, 27.0, 25.9, 17.93, 17.90.

HRMS *m*/*z* (GC-EI): calculated for $C_{16}H_{22}O^+$ ($[M]^+$): 230.16651; found: 230.16664.

(Z)-1-(2,6-dimethylhepta-1,5-dien-1-yl)-4-methoxybenzene (*Z*)-1i

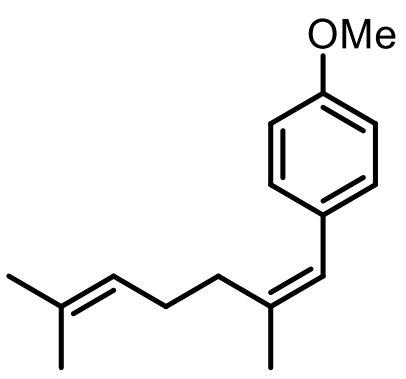

(*Z*)-**1i**

colorless oil, R_F (pentane/Et_2O = 20:1) = 0.8.

^{1}H NMR (501 MHz, $CDCl_3$): δ = 7.15 (d, *J* = 8.5 Hz, 2H), 6.85 (d, *J* = 8.5 Hz, 2H), 6.23 (s, 1H), 5.14–5.10 (m, 1H), 3.80 (s, 3H), 2.26–2.14 (m, 4H), 1.88 (d, *J* = 1.6 Hz, 3H), 1.68 (s, 3H), 1.62 (s, 3H).

^{13}C NMR (126 MHz, $CDCl_3$): δ = 157.9, 138.2, 131.9, 131.3, 129.8, 125.2, 124.3, 113.6, 55.4, 32.8, 26.8, 25.9, 24.2, 17.8.

HRMS *m*/*z* (GC-EI): calculated for $C_{16}H_{22}O^+$ ($[M]^+$): 230.16651; found: 230.16634.

1-(2,6-dimethylhepta-1,5-dien-1-yl)-4-fluorobenzene 1j

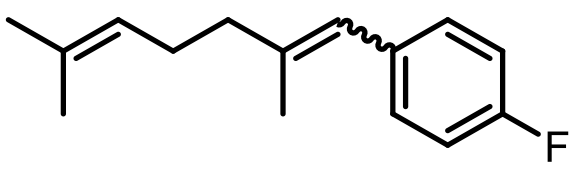

1j

Prepared according to **GP 2**, eluent: pentane, 867 mg (79%), *Z*:*E* = 44:56, The mixture was further separated by preparative HPLC (250 mm Chiralpak OJ-H, 20 mm i.d. 5 µm, MeOH/H_2O 95.0:5.0 v/v, 20.0 mL/min, 16.1 MPa, 308 K, 205 nm, UV) to furnish pure *Z* and *E* diene.

(*E*)-1-(2,6-dimethylhepta-1,5-dien-1-yl)-4-fluorobenzene (*E*)-1j

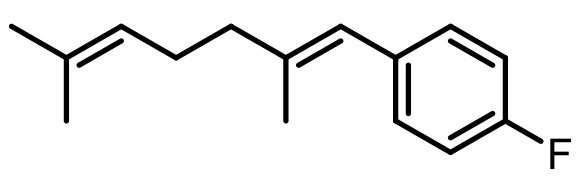

(*E*)-**1j**

colorless oil, $\boldsymbol{R_F}$ (pentane) = 0.8.

^{1}H NMR (501 MHz, $CDCl_3$): δ = 7.19–7.17 (m, 2H), 7.01–6.98 (m, 2H), 6.22 (s, 1H), 5.17–5.14 (m, 1H), 2.22–2.16 (m, 4H), 1.83 (d, *J* = 1.4 Hz, 3H), 1.71 (s, 3H), 1.64 (s, 3H).

^{13}C NMR (126 MHz, $CDCl_3$): δ = 162.2 (d, *J* = 245.0 Hz), 139.0, 134.8 (d, *J* = 3.4 Hz), 132.0, 130.4 (d, *J* = 7.5 Hz), 124.1, 124.0, 115.0 (d, *J* = 21.2 Hz), 40.8, 26.9, 25.9, 17.9.

^{19}F NMR (471 MHz, $CDCl_3$): δ = −117.0 (s, 1F).

HRMS *m*/*z* (GC-EI): calculated for $C_{15}H_{19}F^+$ ($[M]^+$): 218.14653; found: 218.14664.

(Z)-1-(2,6-dimethylhepta-1,5-dien-1-yl)-4-fluorobenzene (*Z*)-1j

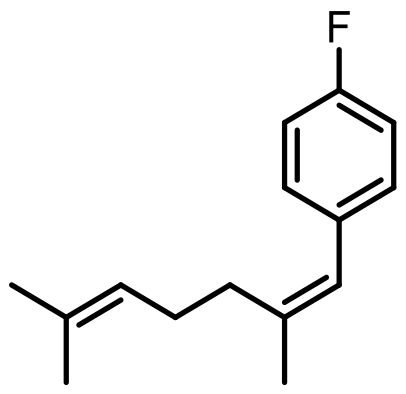

(*Z*)-**1j**

colorless oil, $\boldsymbol{R_F}$ (pentane) = 0.8.

^{1}H NMR (501 MHz, $CDCl_3$): δ = 7.16–7.13 (m, 2H), 7.00–6.96 (m, 3H), 6.24 (s, 1H), 5.11–5.07 (m, 1H), 2.22–2.14 (m, 4H), 1.88 (d, *J* = 1.5 Hz, 3H), 1.68 (s, 3H), 1.60 (s, 3H).

^{13}C NMR (126 MHz, $CDCl_3$): δ = 162.3 (d, *J* = 244.8 Hz), 139.5, 134.7 (d, *J* = 3.4 Hz), 132.1, 130.2 (d, *J* = 7.5 Hz), 124.7, 124.0, 115.1 (d, *J* = 21.2 Hz), 32.7, 26.7, 25.8, 24.1, 17.8.

^{19}F NMR (471 MHz, $CDCl_3$): δ = −117.0 (s, 1F).

HRMS *m*/*z* (GC-EI): calculated for $C_{15}H_{19}F^+$ ($[M]^+$): 218.14653; found: 218.14644.

1-chloro-4-(2,6-dimethylhepta-1,5-dien-1-yl)benzene 1k

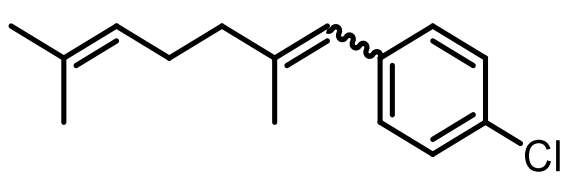

1k

Prepared according to **GP 2**, eluent: pentane, 587 mg (50%), *Z*:*E* = 38:62, The mixture was further separated by preparative HPLC (250 mm Chiralpak OJ-H, 20 mm i.d. 5 µm, MeOH/H_2O 90.0:10.0 v/v, 20.0 mL/min, 16.1 MPa, 308 K, 205 nm, UV) to furnish pure *Z* and *E* diene.

(*E*)-1-chloro-4-(2,6-dimethylhepta-1,5-dien-1-yl)benzene (*E*)-1k

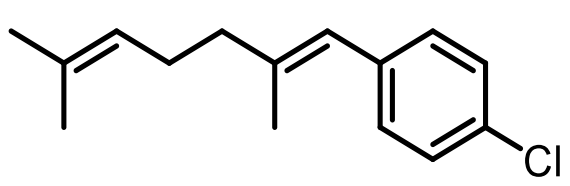

(*E*)-**1k**

colorless oil, $\boldsymbol{R_F}$ (pentane) = 0.8.

^{1}H NMR (501 MHz, $CDCl_3$): δ = 7.28 (d, *J* = 8.4 Hz, 2H), 7.16 (d, *J* = 8.4 Hz, 2H), 6.21 (s, 1H), 5.16–5.14 (m, 1H), 2.20–2.16 (m, 4H), 1.84 (d, *J* = 1.4 Hz, 3H), 1.70 (s, 3H), 1.63 (s, 3H).

^{13}C NMR (126 MHz, $CDCl_3$): δ = 139.9, 137.2, 132.0, 131.6, 130.2, 128.3, 124.0, 123.9, 40.9, 26.8, 25.9, 18.0, 17.9.

HRMS *m*/*z* (GC-EI): calculated for $C_{15}H_{19}Cl^+$ ($[M]^+$): 234.11698; found: 234.11698.

(Z)-1-chloro-4-(2,6-dimethylhepta-1,5-dien-1-yl)benzene (*Z*)-1k

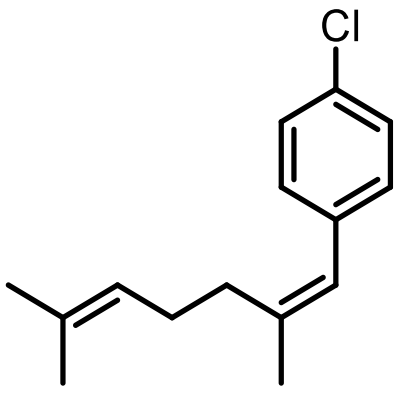

(*Z*)-**1k**

colorless oil, ***R**$_F$* (pentane) = 0.8.

^{1}H NMR (501 MHz, $CDCl_3$): δ = 7.26 (d, *J* = 8.4 Hz, 2H), 7.13 (d, *J* = 8.4 Hz, 2H), 6.22 (s, 1H), 5.10–5.07 (m, 1H), 2.22–2.13 (m, 4H), 1.88 (d, *J* = 1.5 Hz, 3H), 1.68 (s, 3H), 1.60 (s, 3H).

^{13}C NMR (126 MHz, $CDCl_3$): δ = 140.3, 137.1, 132.2, 130.0, 128.4, 128.3, 124.7, 123.9, 32.7, 26.7, 25.8, 24.2, 17.8.

HRMS *m*/*z* (GC-EI): calculated for $C_{15}H_{19}Cl^+$ ($[M]^+$): 234.11698; found: 234.11707.

1-bromo-4-(2,6-dimethylhepta-1,5-dien-1-yl)benzene 1l

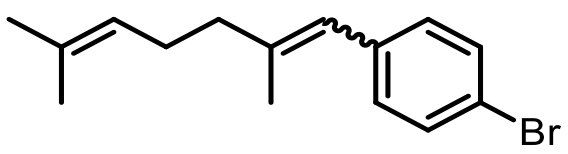

1l

Prepared according to **GP 2**, eluent: pentane, 760 mg (54%), *Z*:*E* = 34:66, The *Z* and *E* mixture was further separated by preparative HPLC (250 mm Chiralpak OJ-H, 20 mm i.d. 5 µm, MeOH/H_2O 90.0:10.0 v/v, 20.0 mL/min, 16.1 MPa, 308 K, 205 nm, UV) to furnish pure *Z* and *E* diene.

(*E*)-1- bromo-4-(2,6-dimethylhepta-1,5-dien-1-yl)benzene (*E*)-1l

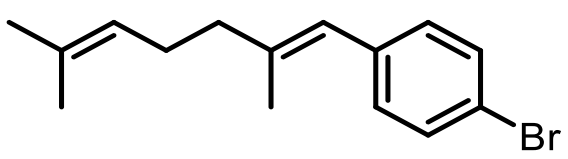

(*E*)-**1l**

colorless oil, ***R**$_F$* (pentane) = 0.8.

^{1}H NMR (501 MHz, $CDCl_3$): δ = 7.43 (d, *J* = 8.4 Hz, 2H), 7.10 (d, *J* = 8.4 Hz, 2H), 6.19 (s, 1H), 5.17–5.13 (m, 1H), 2.20–2.17 (m, 4H), 1.84 (d, *J* = 1.4 Hz, 3H), 1.71 (s, 3H), 1.63 (s, 3H).

^{13}C NMR (126 MHz, $CDCl_3$): δ = 140.1, 137.7, 132.0, 121.2, 130.6, 124.0, 119.7, 40.9, 26.8, 25.9, 18.0, 17.9.

HRMS *m*/*z* (GC-EI): calculated for $C_{15}H_{19}Br^+$ ($[M]^+$): 278.06648; found: 278.06631.

(*Z*)-1- bromo-4-(2,6-dimethylhepta-1,5-dien-1-yl)benzene (*Z*)-1l

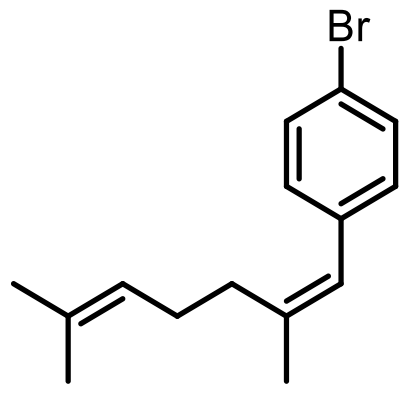

(Z)-**1l**

colorless oil, ***R_F*** (pentane) = 0.8.

¹H NMR (501 MHz, $CDCl_3$): δ = 7.42 (d, *J* = 8.4 Hz, 2H), 7.07 (d, *J* = 8.4 Hz, 2H), 6.20 (s, 1H), 5.10–5.07 (m, 1H), 2.21–2.13 (m, 4H), 1.88 (d, *J* = 1.5 Hz, 3H), 1.68 (s, 3H), 1.60 (s, 3H).

¹³C NMR (126 MHz, $CDCl_3$): δ = 140.4, 137.6, 131.4, 131.3, 130.4, 124.7, 123.9, 32.7, 26.7, 25.8, 24.2, 17.8.

HRMS *m*/*z* (GC-EI): calculated for $C_{15}H_{19}Br^+$ ($[M]^+$): 278.06648; found: 278.06643.

(*E*)-(3,7-dimethylocta-2,6-dien-1-yl)benzene 1m

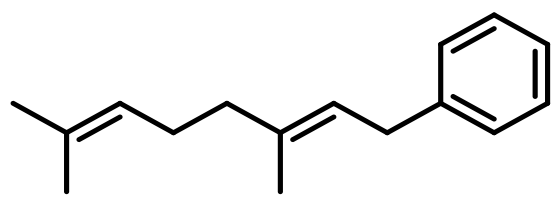

1m

Prepared according to **GP 1**, stirred for 2 h, eluent: pentane, 885 mg (83%), colorless oil.

R_F (pentane) = 0.7.

¹H NMR (501 MHz, $CDCl_3$): δ = 7.32–7.28 (m, 2H), 7.22–7.21 (m, 3H), 5.39–5.36 (m, 1H), 5.16–5.12 (m, 1H), 3.40 (d, *J* = 7.3 Hz, 2H), 2.17–2.07 (m, 4H), 1.74 (s, 3H), 1.72 (s, 3H), 1.63 (s, 3H).

¹³C NMR (126 MHz, $CDCl_3$): δ = 141.9, 136.4, 131.6, 128.54, 128.53, 125.8, 124.4, 123.2, 39.9, 34.3, 26.8, 25.9, 17.8, 16.3.

HRMS *m*/*z* (GC-EI): calculated for $C_{16}H_{22}^+$ ($[M]^+$) 214.17160; found: 214.17191.

(*E*)-(3,7-dimethylocta-2,6-dien-1-yl)benzene 1n

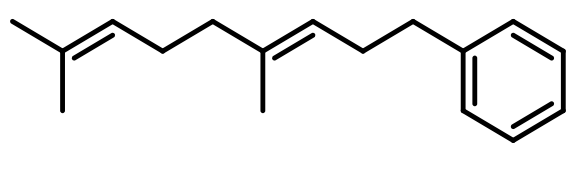

1n

Prepared according to **GP 1**, stirred for 2 h, eluent: pentane, 912 mg (80%), colorless oil.

R_F (pentane) = 0.7.

¹H NMR (501 MHz, $CDCl_3$): δ = 7.29–7.26 (m, 2H), 7.21–7.18 (m, 3H), 5.21–5.17 (m, 1H), 5.11–5.08 (m, 1H), 2.66 (t, *J* = 7.5 Hz, 2H), 2.33–2.29 (m, 2H), 2.09–1.97 (m, 4H), 1.69 (s, 3H), 1.61 (s, 3H), 1.56 (s, 3H).

¹³C NMR (126 MHz, $CDCl_3$): δ = 142.6, 135.9, 131.5, 128.6, 128.3, 125.8, 124.5, 123.7, 39.9, 36.3, 30.1, 26.9, 25.8, 17.8, 16.1.

HRMS *m*/*z* (GC-CI): calculated for $C_{17}H_{28}N^+$ ($[M+NH_4]^+$) 246.22162; found: 246.22180.

6-ethyl-2-methylocta-2,6-diene 1o

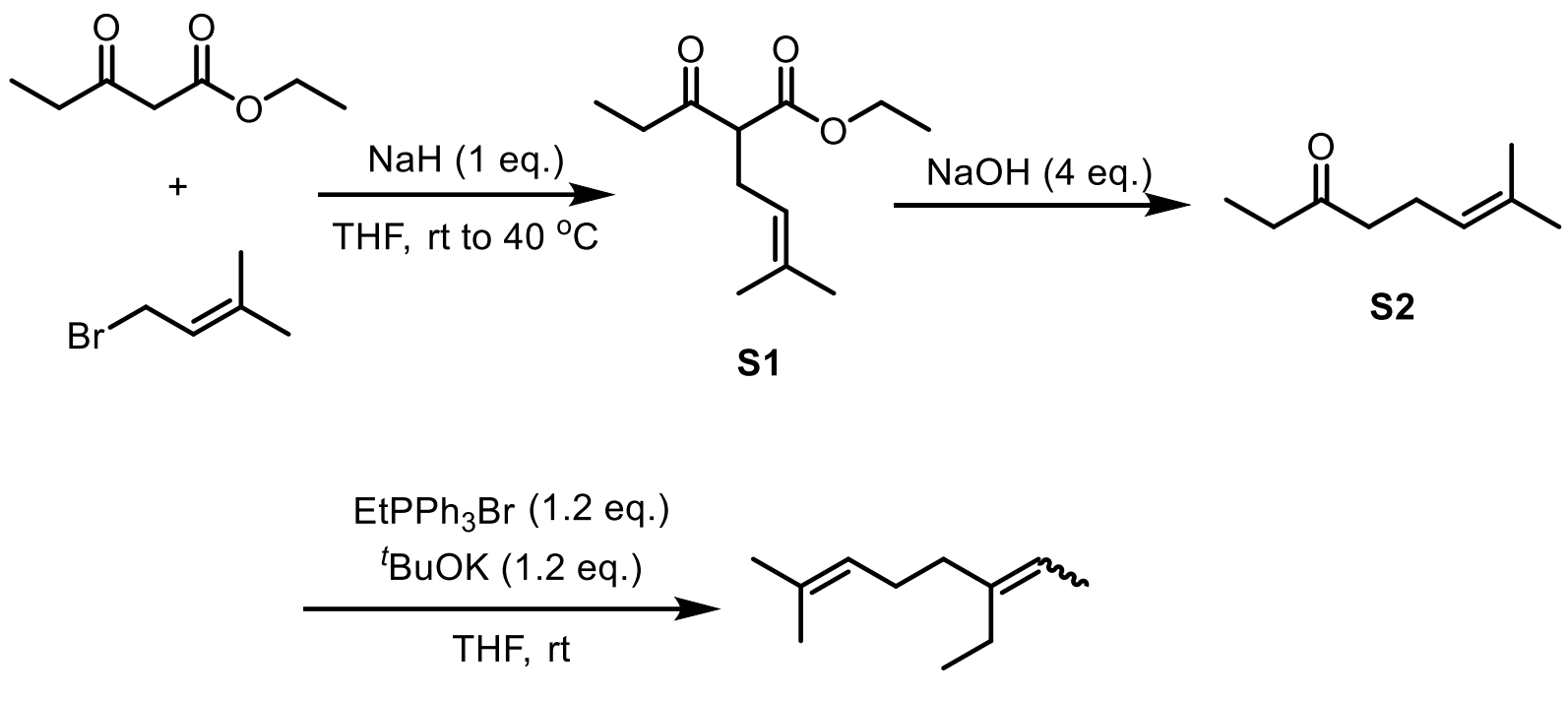

S1 and **S2** were synthesized according to a literature procedure.[2] A solution of ethyl propionylacetate (15 mmol, 1.0 eq.) in THF (60 mL) was treated with NaH (1.0 eq., 60% in mineral oil), stirred for 1 h and treated with prenyl bromide (1.1 eq.). The mixture was warmed to 40 °C and stirred overnight. The mixture was cooled to room temperature and MeOH was added. The crude product was purified by column chromatography on silica gel eluting with pentane/Et_2O (95:5) got a colorless oil **S1** in 64% yield.

A solution of the **S1** (5.20 mmol, 1.0 eq.) in MeOH–H_2O (52 mL, 2:1) was treated with NaOH (4.0 eq.) and heated under reflux for 20 h. The mixture was cooled to room temperature and the MeOH was removed. EtOAc (50 mL) was added and the layers were separated. The aqueous layer was then washed with EtOAc (20 mL × 3) and the combined organic fractions were dried ($MgSO_4$), filtered and evaporated. Purification by column chromatography on silica gel, eluting with pentane/Et_2O = 99:1, gave **S2** as a colorless oil in 99% yield.

To a suspension of *ᵗ*BuOK (1.2 eq.) in anhydrous THF (20 mL) was added $EtPPh_3Br$ (1.2 eq.) under argon atmosphere. The suspension was stirred at room temperature for 1 h. Then corresponding ketone (5 mmol, 1.0 eq.) was added and the reaction mixture was stirred at the same temperature for 1 h. Then the mixture was filtered through a short pad of silica gel, which was subsequently washed with ethyl acetate (200 mL). After evaporation of the organic solvent, the residue was purified by silica gel column chromatography to provide alkene as a colorless oil **1o** in 90% yield. The *Z* and *E* mixture was further separated by preparative HPLC (250 mm Chiralpak OJ-H, 20 mm i.d. 5 µm, MeOH/H_2O 80.0:20.0 v/v, 20.0 mL/min, 13.2 MPa, 308 K, 205 nm, UV) to furnish pure *Z* and *E* diene.

(*E*)-6-ethyl-2-methylocta-2,6-diene (*E*)-1o

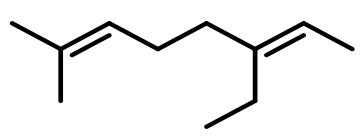

(*E*)-1o

***R**$_F$* (pentane) = 0.9.

^{1}H NMR (501 MHz, $CDCl_3$): δ = 5.20 (q, *J* = 6.7 Hz, 1H), 5.13–5.10 (m, 1H), 2.07–1.97 (m, 6H), 1.69 (s, 3H), 1.61 (s, 3H), 1.59 (d, *J* = 6.7 Hz, 3H), 0.98 (t, *J* = 7.6 Hz, 3H)

^{13}C NMR (126 MHz, $CDCl_3$): δ = 141.9, 131.4, 124.7, 117.8, 36.8, 27.1, 25.8, 23.0, 17.8, 13.1, 13.0.

HRMS *m*/*z* (GC-EI): calculated for $C_{11}H_{20}^+$ ($[M]^+$): 152.15595; found: 152.15571.

(*Z*)-(7-methylocta-2,6-dien-3-yl)benzene 1p

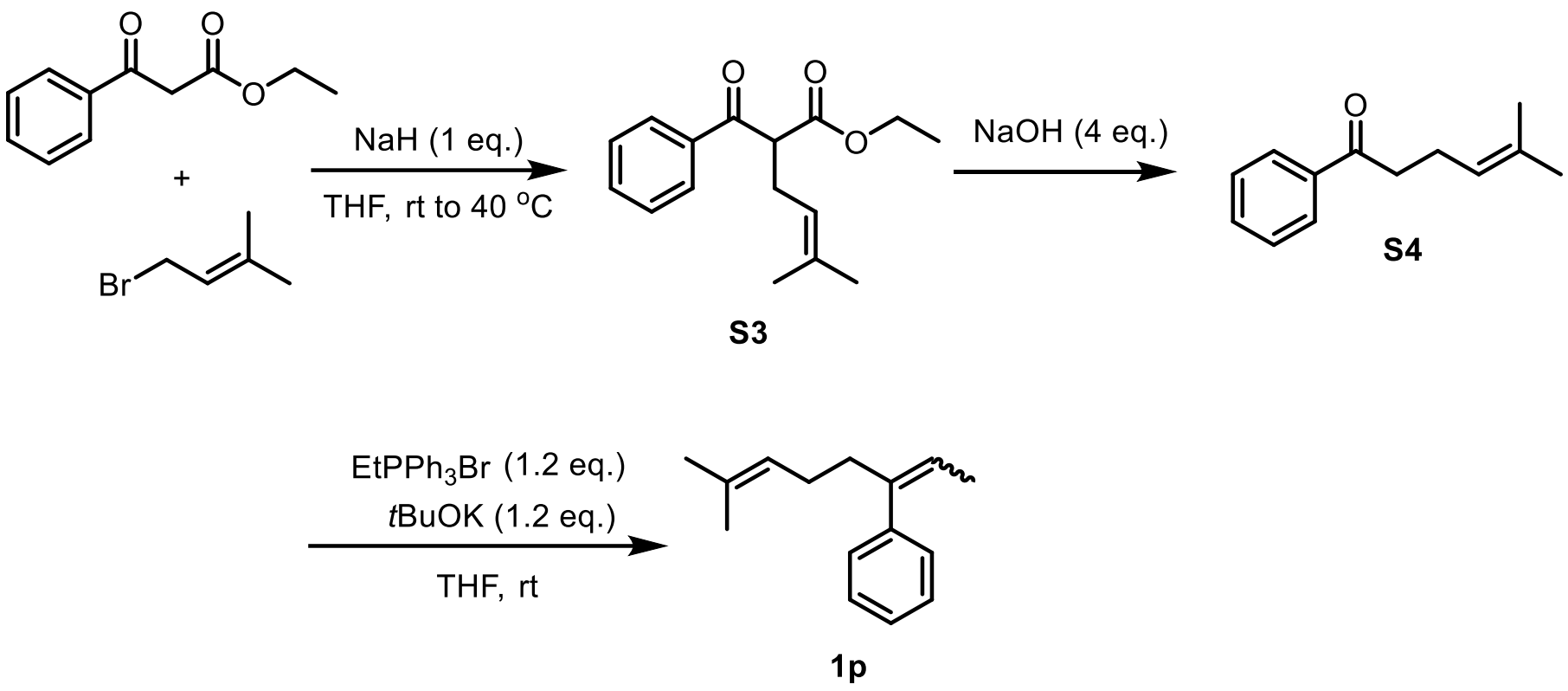

S3 and S4 were synthesized according to a literature procedure.[3] A solution of ethyl-benzoylacetate (15 mmol, 1.0 eq.) in THF (60 mL) was treated with NaH (1.0 eq., 60% in mineral oil), stirred for 1 h and treated with prenyl bromide (1.1 eq.). The mixture was warmed to 40 °C and stirred overnight. The mixture was cooled to room temperature and MeOH was added. The crude product was purified by column chromatography on silica gel eluting with pentane/Et_2O (95:5) got a colorless oil **S3** in 73% yield.

A solution of the **S3** (10.4 mmol, 1.0 eq.) in MeOH/H_2O (52 mL, 2:1) was treated with NaOH (4.0 eq.) and heated under reflux for 20 h. The mixture was cooled to room temperature and the MeOH was removed. EtOAc (50 mL) was added and the layers were separated. The aqueous layer was then washed with EtOAc (20 mL × 3) and the combined organic fractions were dried ($MgSO_4$), filtered and evaporated. Purification by column chromatography on silica gel, eluting with pentane/Et_2O = 99:1, gave **S4** as a colorless oil in 87% yield.

To a suspension of ^{t}BuOK (1.2 eq.) in anhydrous THF (20 mL) was added $EtPPh_3Br$ (1.2 eq.) under argon atmosphere. The suspension was stirred at room temperature for 1 h. Then corresponding ketone (5 mmol, 1.0 eq.) was added and the reaction mixture was stirred at the same temperature for 1 h. Then the mixture was filtered through a short pad of silica gel, which was subsequently washed with ethyl acetate (200 mL). After evaporation of the organic solvent, the residue was purified by silica gel column chromatography to provide alkene as a colorless oil **1p** in 50% yield.

(*Z*)-(7-methylocta-2,6-dien-3-yl)benzene (*Z*)-1p

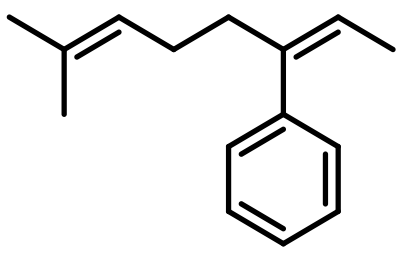

(Z)-1p

***R**$_F$* (pentane) = 0.7.

^{1}H NMR (501 MHz, $CDCl_3$): δ = 7.34 (t, *J* = 7.7 Hz, 2H), 7.24 (t, *J* = 7.7 Hz, 1H), 7.16–7.14 (m, 2H), 5.57 (q, *J* = 6.8 Hz, 1H), 5.11–5.07 (m, 1H), 2.37–2.33 (m, 2H), 2.00–1.96 (m, 2H), 1.66 (s, 3H), 1.57 (d, *J* = 6.8 Hz, 3H), 1.52 (s, 3H).

^{13}C NMR (126 MHz, $CDCl_3$): δ = 141.7, 141.2, 131.6, 128.7, 128.1, 126.4, 124.3, 121.3, 39.4, 27.1, 25.8, 17.8, 14.8.

HRMS *m*/*z* (GC-CI): calculated for $C_{15}H_{24}N^+$ ($[M+NH_4]^+$) 218.19032; found: 218.19007.

(*E*)-(4-methylhept-4-en-1-ylidene)cyclohexane 1q

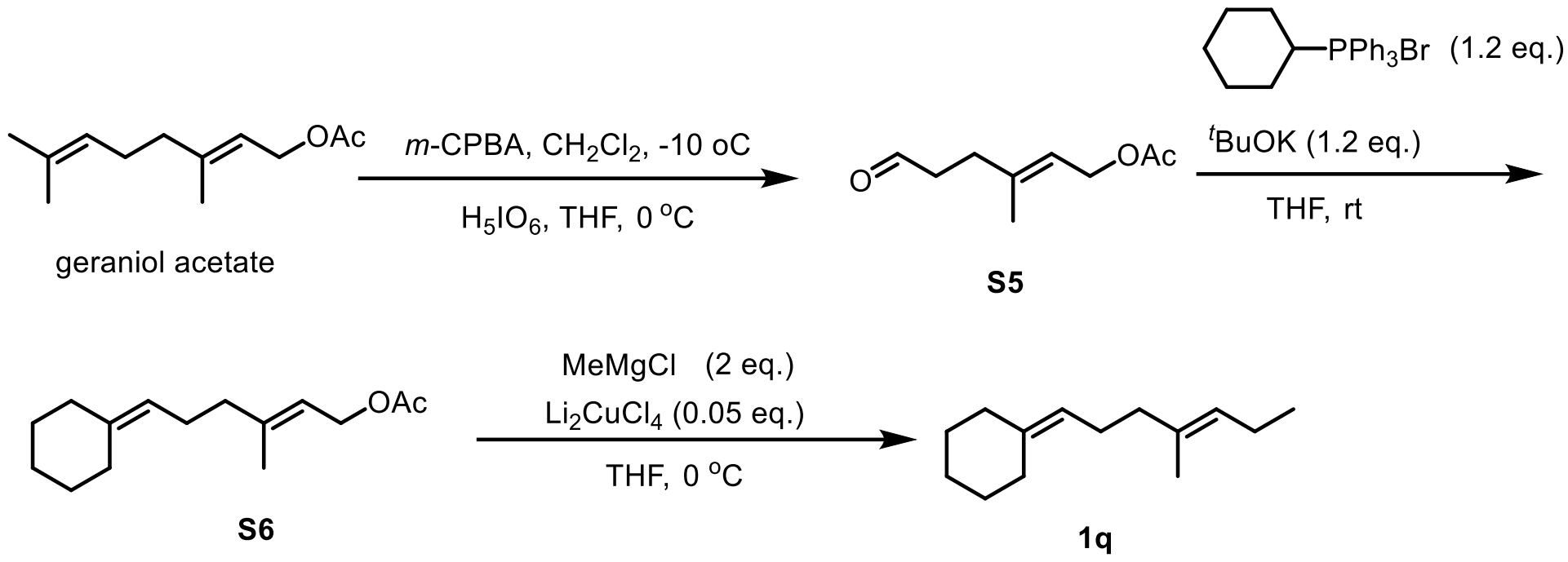

S5 was synthesized according to a literature procedure.[4] To a cooled (−10 °C) solution of geranyl acetate (10 mmol, 1 eq.) in dichloromethane 200 mL was added a solution of m-CPBA (77% wt, 1 eq.) in 20 mL dichloromethane dropwise over 2 h. The resulting slurry was then warmed to room temperature, stirred for 2 h, and then poured into 2 M NaOH (50 mL). The layers were separated and the aqueous phase extracted with dichloromethane (500 mL). The combined organics were then dried (Na_2SO_4), filtered, and concentrated. The resulting clear oil was dissolved in diethyl ether (20 mL), and this solution was then added dropwise to a cooled (0 °C) solution of H_5IO_6 (2.3 g, 10 mmol) in THF (50 mL). After 30 min, the reaction mixture was poured into saturated sodium bicarbonate (25 mL) and the mixture was extracted with diethyl ether (2 × 25mL). The combined organic layers were washed with brine, dried ($MgSO_4$), filtered, and concentrated. The residue was purified by flash chromatography (pentane/EtOAc = 5:1) to give 1.42 g (83%) of aldehyde **S5** as a clear oil.

To a suspension of ^{t}BuOK (6 mmol, 1.2 eq.) in anhydrous THF (20 mL) was added cyclohexyltriphenylphosphonium bromide (1.2 eq.) under argon atmosphere. The suspension was stirred at room temperature for 1 h. Then corresponding ketone **S5** (5 mmol, 1.0 eq.) was added and the reaction mixture was stirred at the same temperature for 1 h. Then the mixture was filtered through a short pad of silica gel, which was subsequently washed with ethyl acetate (20 mL). After evaporation of the organic solvent, the residue was purified by silica gel column chromatography (pentane/EtOAc = 5:1) to give 831 mg (70%) of aldehyde **S6** as a yellow oil.

To a solution of **S6** (3 mmol, 1 eq.) in THF (10 mL) at 0 °C, 0.1 M Li_2CuCl_4 solution in THF (0.05 eq) was added dropwise and then a solution of Grignard reagent MeMgCl (2 eq) was added dropwise at 0 °C. After 2 h at 0 °C, the mixture was quenched with saturated aqueous NH_4Cl solution and the aqueous layer extracted with diethyl ether. Combined organic layers were washed with brine, dried over Na_2SO_4, and concentrated under reduced pressure. The residue was purified by silica gel column chromatography (pentane) to afford 465 mg **1q** (82%) as a clear oil. **^{1}H NMR** (501 MHz, $CDCl_3$): δ = 5.14 (t, *J* = 6.4 Hz, 1H), 5.06 (t, *J* = 6.9 Hz, 1H), 2.13-1.96 (m, 10H), 1.54–1.48 (m, 6H), 0.95 (t, *J* = 7.5 Hz, 3H). **^{13}C NMR** (126 MHz, $CDCl_3$): δ = 139.7, 134.4, 126.6, 121.2, 40.2, 37.3, 28.9, 28.0, 27.1, 25.9, 21.3, 16.0, 14.6. **HRMS** *m/z* (GC-EI): calculated for $C_{14}H_{24}^+$ ($[M]^+$) 192.187250; found: 192.187170.

Methyl (*E*)-4,8-dimethylnona-3,7-dienoate, methyl (*E*)-homogeranate 1r

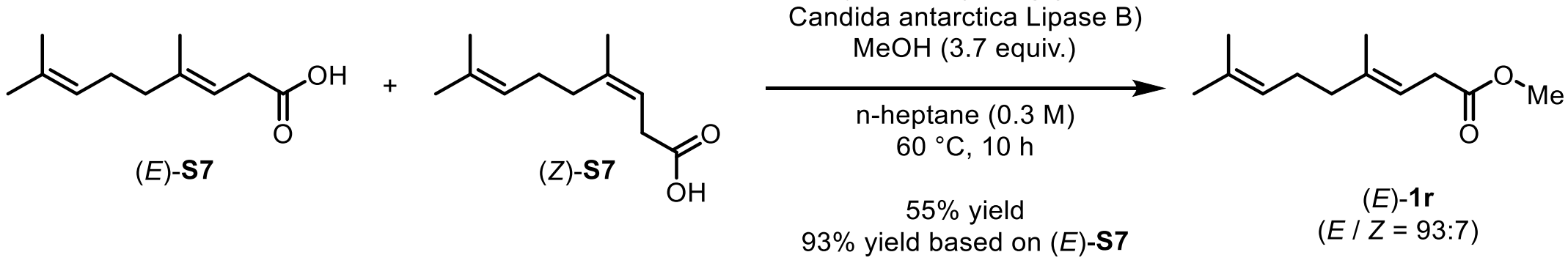

Resolution of an (*E*/*Z*)-mixture of carboxylic acid **S7** was accomplished according to a literature procedure reported by Schelwies and coworkers.[5,6] A 100 mL round-bottom flask equipped with a PTFE-coated magnetic stir bar was charged with (*E*/*Z*)-**S7** (1.82 g, 10.0 mmol, 1.00 eq.). The substrate was dissolved in *n*-heptane (30 mL, 0.3 m). Then, methanol (1.48 mL, 1.17 g, 36.6 mmol, 3.66 eq.) was added and the resulting colorless solution was immersed in a preheated oil bath at 60 °C. Subsequently, Lipase B from *Candida antarctica* (CALB, 36.5 mg, 20 mg/g, 2 wt%) was added in one portion and the resulting colorless suspension was stirred at 50 °C for 24 h. The progress of the reaction was regularly monitored by taking samples. At each time point, approximately 0.1 mL of the reaction solution were collected with a syringe, transferred to a 1.5 mL Eppendorf® tube, diluted with MTBE (0.5 mL) and centrifuged at 13400 g for 5 min to remove the immobilized CALB. The supernatant solution was collected with a syringe, filtered over a 0.45 µm syringe filter and concentrated under reduced pressure. The residual colorless oil was subsequently dissolved in $CDCl_3$ and the reaction progress was analyzed by ^{1}H NMR spectroscopy. After additional 8 h at 60 °C, the reaction mixture was filtered over cotton wool to remove the immobilized lipase and concentrated under reduced pressure. The residue was dissolved in hexanes (50 mL) and cooled to 0 °C, water (20 mL), methanol (5 mL) and NaOH (25 wt% in water, 2 mL) were added and the biphasic mixture was stirred for 5 min. Additional water (20 mL) was added, the mixture was transferred to a separatory funnel, the organic phase was separated, and the aqueous phase was extracted with hexanes (50 mL). The combined organic phases were dried over Na_2SO_4, filtered and concentrated under reduced pressure to afford **1r** (1.08 g, 5.50 mmol, 55% yield, 93% yield based on the *E*-isomer, 93:7 *E*/*Z*). Purification by flash column chromatography on silica gel using hexanes/MTBE (gradient elution, 19:1 → 9:1 *v*/*v*) as eluent enabled a slight further enrichment of the *E*-isomer (94:6 *E*/*Z*).

Physical state: colorless oil.
TLC (SiO_2, hexanes/MTBE 9:1 *v*/*v*): R_F = 0.35 ($KMnO_4$ or CAM stain).
^{1}H NMR (501 MHz, $CDCl_3$): δ (ppm) = 5.32 (th, *J* = 7.1, 1.4 Hz, 1H), 5.08 (thept, *J* = 6.9, 1.4 Hz, 1H), 3.68 (s, 3H), 3.05 (dq, *J* = 7.1, 1.0 Hz, 2H), 2.13–2.06 (m, 2H), 2.06–2.00 (m, 2H), 1.67 (q, *J* = 1.3 Hz, 3H), 1.63 (q, *J* = 1.0 Hz, 3H), 1.59 (d, *J* = 1.3 Hz, 3H).
^{13}C{^{1}H} NMR (126 MHz, $CDCl_3$): δ (ppm) = 173.1, 139.2, 131.8, 124.1, 115.7, 51.8, 39.7, 33.7, 26.6, 25.8, 17.8, 16.5.
HRMS *m*/*z* (ESI^+): calculated for $C_{12}H_{20}O_2Na^+$ $[M+Na]^+$: 219.13555, found: 219.13575.

2.2 Synthesis of polyene substrates 7a–7h

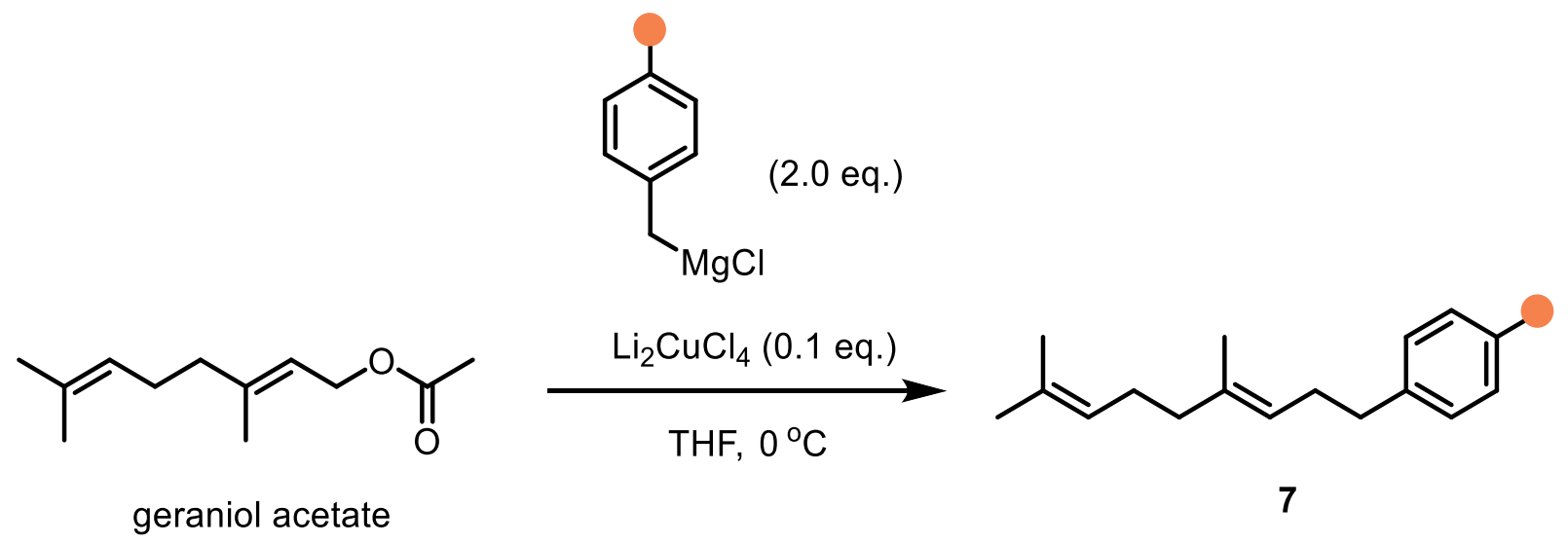

General Procedure 3 (GP 3): A flame-dried flask under argon was charged with geraniol acetate (5 mmol, 1.0 eq.) and dry THF (0.5 M). 0.1 M Li_2CuCl_4 (0.1 eq) solution in THF was added dropwise, then a solution of Grignard

reagent (2.0 eq) was added dropwise at 0 ºC. After 2 h at 0 ºC, the mixture was quenched with saturated aqueous NH_4Cl solution and the aqueous layer extracted with diethyl ether. Combined organic layers were washed with brine, dried over Na_2SO_4, and concentrated under reduced pressure. The residue was purified by silica gel column chromatography to afford the coupling product **7**.

(*E*)-(3,7-dimethylocta-2,6-dien-1-yl)benzene 7a

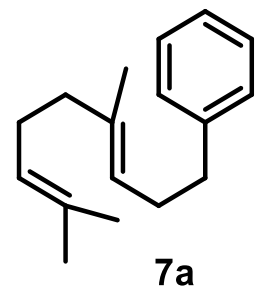

7a

Prepared according to **GP 3**, stirred for 2 h, eluent: pentane, 912 mg (80%), colorless oil.

R_F (pentane) = 0.7.

^{1}H NMR (501 MHz, $CDCl_3$): δ = 7.29–7.26 (m, 2H), 7.21–7.18 (m, 3H), 5.21–5.17 (m, 1H), 5.11–5.08 (m, 1H), 2.66 (t, *J* = 7.5 Hz, 2H), 2.33–2.29 (m, 2H), 2.09–1.97 (m, 4H), 1.69 (s, 3H), 1.61 (s, 3H), 1.56 (s, 3H).

^{13}C NMR (126 MHz, $CDCl_3$): δ = 142.6, 135.9, 131.5, 128.6, 128.3, 125.8, 124.5, 123.7, 39.9, 36.3, 30.1, 26.9, 25.8, 17.8, 16.1.

HRMS *m*/*z* (GC-CI): calculated for $C_{17}H_{28}N^+$ ($[M+NH_4]^+$) 246.22162; found: 246.22180.

(*E*)-1-(4,8-dimethylnona-3,7-dien-1-yl)-4-methylbenzene 7b

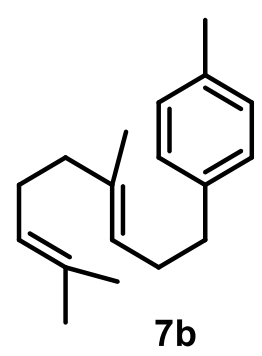

7b

Prepared according to **GP 3**, stirred for 2 h, eluent: pentane, 850 mg (70%), colorless oil.

R_F (pentane) = 0.7.

^{1}H NMR (501 MHz, $CDCl_3$): δ = 7.09 (br, 4H), 5.21–5.17 (m, 1H), 5.11–5.07 (m, 1H), 2.61–2.58 (m, 2H), 2.32 (s, 3H), 2.31–2.26 (m, 2H), 2.09–2.04 (m, 2H), 2.00–1.97 (m, 2H), 1.69 (s, 3H), 1.61 (s, 3H), 1.57 (s, 3H).

^{13}C NMR (126 MHz, $CDCl_3$): δ = 139.5, 135.8, 135.2, 131.5, 129.0, 128.5, 124.5, 123.9, 39.9, 35.8, 30.2, 26.9, 25.8, 21.2, 17.8, 16.1.

HRMS *m*/*z* (GC-CI): calculated for $C_{18}H_{30}N^+$ ($[M+NH_4]^+$) 260.23728; found: 260.237200.

(*E*)-1-(4,8-dimethylnona-3,7-dien-1-yl)-4-isopropylbenzene 7c

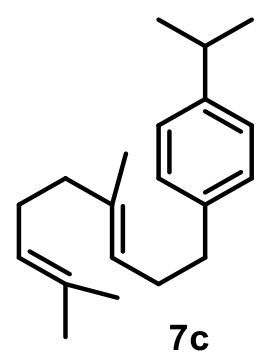

7c

Prepared according to **GP 3**, stirred for 2 h, eluent: pentane, 750 mg (55%), colorless oil.

R_F (pentane) = 0.7.

^{1}H NMR (501 MHz, $CDCl_3$): δ =7.16–7.11 (br, 4H), 5.22–5.18 (m, 1H), 5.11–5.09 (m, 1H), 2.91 (t, *J* = 6.9 Hz, 1H), 2.62 (t, *J* = 7.4 Hz, 2H), 2.32–2.27 (m, 2H), 2.10–1.97 (m, 4H), 1.69 (s, 3H), 1.61 (s, 3H), 1.57 (s, 3H), 1.25 (d, *J* = 6.9 Hz, 6H).
^{13}C NMR (126 MHz, $CDCl_3$): δ = 146.3, 139.9, 135.8, 131.5, 128.5, 126.4, 124.5, 123.9, 39.9, 35.9, 33.8, 30.1, 26.9, 25.9, 24.2, 17.8, 16.1.
HRMS *m*/*z* (GC-CI): calculated for $C_{20}H_{34}N^+$ ($[M+NH_4]^+$) 288.26858; found: 288.26837.

(*E*)-1-(tert-butyl)-4-(4,8-dimethylnona-3,7-dien-1-yl)benzene 7d

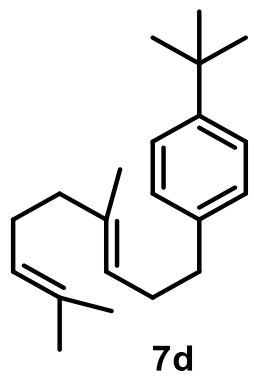

7d

Prepared according to **GP 3**, stirred for 2 h, eluent: pentane, 808 mg (57%), colorless oil.
R$_F$ (pentane) = 0.7.
^{1}H NMR (501 MHz, $CDCl_3$): δ = 7.31 (d, *J* = 8.2 Hz, 2H), 7.15 (d, *J* = 8.2 Hz, 2H), 5.22–5.19 (m, 1H), 5.12–5.09 (m, 1H), 2.63 (t, *J* = 7.5 Hz, 2H), 2.32 (m, 2H), 2.08–1.97 (m, 4H), 1.69 (s, 3H), 1.61 (s, 3H), 1.57 (s, 3H), 1.32 (s, 9H).
^{13}C NMR (126 MHz, $CDCl_3$): δ = 148.6, 139.5, 135.8, 131.5, 128.2, 125.2, 124.5, 124.0, 39.9, 35.7, 34.5, 31.6, 30.1, 26.9, 25.9, 17.8, 16.1.
HRMS *m*/*z* (GC-CI): calculated for $C_{21}H_{36}N^+$ ($[M+NH_4]^+$) 302.28423; found: 302.28371.

(*E*)-1-(4,8-dimethylnona-3,7-dien-1-yl)-4-methoxybenzene 7e

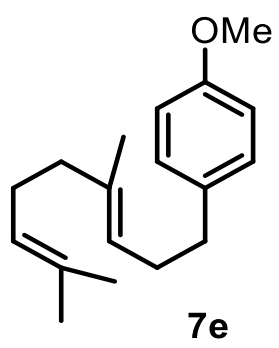

7e

Prepared according to **GP 3**, stirred for 2 h, eluent: pentane/Et_2O 10:1, 800 mg (63%), colorless oil.
R$_F$ (pentane) = 0.7.
^{1}H NMR (501 MHz, $CDCl_3$): δ = 7.12 (d, *J* = 8.7 Hz, 2H), 6.83 (d, *J* = 8.7 Hz, 2H), 5.20–5.16 (m, 1H), 5.11–5.08 (m, 1H), 3.79 (s, 3H), 2.60 (t, *J* = 7.5 Hz, 2H), 2.29–2.25 (m, 2H), 2.09–1.96 (m, 4H), 1.69 (s, 3H), 1.61 (s, 3H), 1.56 (s, 3H).
^{13}C NMR (126 MHz, $CDCl_3$): δ = 157.8, 135.8, 134.7, 131.5, 129.5, 124.5, 123.8, 113.8, 55.4, 39.9, 35.4, 30.3, 26.9, 25.8, 17.8, 16.1.
HRMS *m*/*z* (GC-CI): calculated for $C_{18}H_{27}O^+$ ($[M+H]^+$) 259.20564; found: 259.20573.

(*E*)-1-(4,8-dimethylnona-3,7-dien-1-yl)-4-fluorobenzene 7f

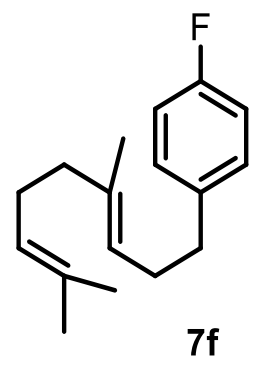

7f

Prepared according to **GP 3**, stirred for 2 h, eluent: pentane, 800 mg (65%), colorless oil.

***R*$_F$** (pentane) = 0.7.

^{1}H NMR (501 MHz, $CDCl_3$): δ = 7.14–7.11 (m, 2H), 6.97–6.93 (m, 2H), 5.17–5.14 (m, 1H), 5.10–5.06 (m, 1H), 2.62 (t, *J* = 7.5 Hz, 2H), 2.30–2.25 (m, 2H), 2.06–1.95 (m, 4H), 1.69 (s, 3H), 1.60 (s, 3H), 1.53 (s, 3H).

^{13}C NMR (126 MHz, $CDCl_3$): δ = 162.3 (d, *J* = 243.1 Hz), 138.11 (d, *J* = 3.2 Hz), 136.2, 131.5, 130.0 (d, *J* = 7.7 Hz), 124.4, 123.4, 115.1 (d, *J* = 21.0 Hz), 39.8, 35.4, 30.1, 26.8, 25.8, 17.8, 16.1.

^{19}F NMR (471 MHz, $CDCl_3$) δ = −118.14.

HRMS *m*/*z* (GC-CI): calculated for $C_{17}H_{27}NF^+$ ($[M+NH_4]^+$) 264.21220; found: 264.21178.

(*E*)-1-chloro-4-(4,8-dimethylnona-3,7-dien-1-yl)benzene 7g

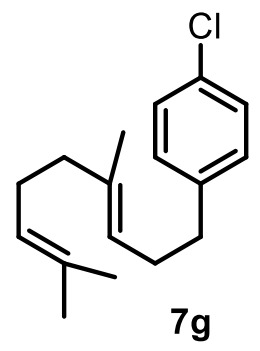

7g

Prepared according to **GP 3**, stirred for 2 h, eluent: pentane, 870 mg (64%), colorless oil.

***R*$_F$** (pentane) = 0.7.

^{1}H NMR (501 MHz, $CDCl_3$): δ = 7.24 (d, *J* = 8.4 Hz, 2H), 7.12 (d, *J* = 8.4 Hz, 2H), 5.16–5.12 (m, 1H), 5.10–5.06 (m, 1H), 2.62 (t, *J* = 7.5 Hz, 2H), 2.30–2.25 (m, 2H), 2.07–1.96 (m, 4H), 1.68 (s, 3H), 1.60 (s, 3H), 1.54 (s, 3H).

^{13}C NMR (126 MHz, $CDCl_3$): δ = 140.9, 136.3, 131.54, 131.49, 130.0, 128.4, 124.4, 123.3, 39.8, 35.6, 29.9, 26.8, 25.8, 17.8, 16.1.

HRMS *m*/*z* (GC-CI): calculated for $C_{17}H_{27}NCl^+$ ($[M+NH_4]^+$) 280.18265; found: 280.18237.

(*E*)-1-bromo-4-(4,8-dimethylnona-3,7-dien-1-yl)benzene 7h

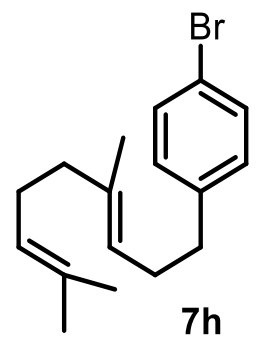

7h

Prepared according to **GP 3**, stirred for 2 h, eluent: pentane, 750 mg (49%), colorless oil.

***R*$_F$** (pentane) = 0.7.

^{1}H NMR (501 MHz, $CDCl_3$): δ = 7.39 (d, *J* = 8.4 Hz, 2H), 7.06 (d, *J* = 8.4 Hz, 2H), 5.16–5.12 (m, 1H), 5.10–5.06 (m, 1H), 2.61 (t, *J* = 7.5 Hz, 2H), 2.30–2.25 (m, 2H), 2.07–1.96 (m, 4H), 1.68 (s, 3H), 1.60 (s, 3H), 1.54 (s, 3H).

^{13}C NMR (126 MHz, $CDCl_3$): δ = 141.5, 136.3, 131.5, 131.4, 130.4, 124.4, 123.3, 119.5, 39.8, 35.6, 29.8, 26.8, 25.8, 17.8, 16.1.

HRMS *m*/*z* (GC-CI): calculated for $C_{17}H_{27}NBr^+$ ($[M+NH_4]^+$) 324.132149; found: 324.131560.

2.3 Synthesis of polyene substrates 10a–10c

10b and **10c** are commercially available. The synthesis of **10a** is described as follows:

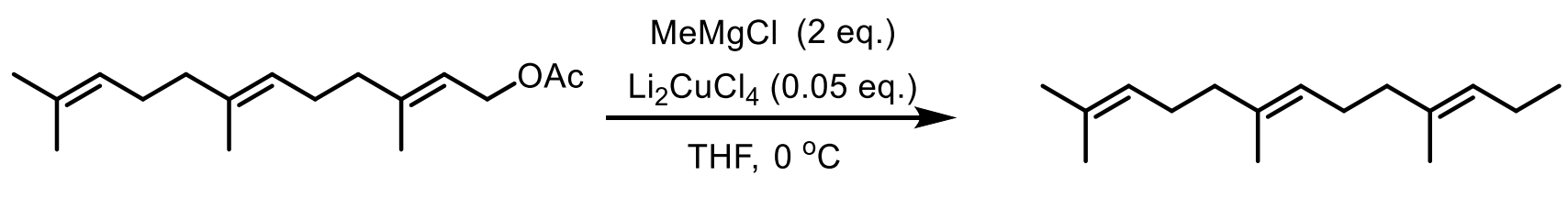

(*E*)-2,6-dimethylnona-2,6-diene 10a A flame-dried flask under argon was charged with farnesyl acetate (5 mmol, 1.0 eq.) and dry THF (0.5 M). 0.1 M Li_2CuCl_4 (0.05 eq.) solution in THF was added dropwise and then a solution of Grignard reagent MeMgCl (2 eq.) was added dropwise at 0 °C. After 2 h at 0 °C, the mixture was quenched with saturated aqueous NH_4Cl solution and the aqueous layer extracted with diethyl ether. Combined organic layers were washed with brine, dried over Na_2SO_4, and concentrated under reduced pressure. The residue was purified by silica gel column chromatography (pentane) to afford the coupling product **10a** 764 mg (69%), colorless oil.

^{1}H NMR (501 MHz, $CDCl_3$): δ = 5.14–5.09 (m, 3H), 2.09–2.06 (m, 4H), 2.01–1.96 (m, 6H), 1.68 (s, 3H), 1.60 (s, 9H), 0.95 (t, *J* = 7.5 Hz, 3H).

^{13}C NMR (126 MHz, $CDCl_3$): δ = 135.0, 134.4, 131.4, 126.6, 124.6, 124.4, 39.9, 39.8, 26.9, 26.8, 25.8, 21.3, 17.8, 16.2, 16.0, 14.5.

HRMS *m*/*z* (GC-CI): calculated for $C_{16}H_{32}N^+$ ($[M+NH_4]^+$) 238,25292; found: 238,25303.

3. Procedure for reaction development

3.1 Development of the catalytic asymmetric geranyl cyclization

General Procedure: An oven-dried 1.5 mL vial was charged with catalyst and a magnetic stir bar under an atmosphere of argon, 107 uL dry solvent and additive were added. The vial was cooled to required reaction temperature. Then **1a** (25.0 µL, 0.137 mmol, 1.0 eq.) was added and the reaction was stirred for 3 days.

The reaction mixture was quenched with triethylamine (0.257 mmol, 3.8 µL, 0.2 eq.) at reaction temperature, then CH_2Br_2 (internal standard, 9.6 µL, 0.137 mmol, 1.0 eq.) was added. Then an aliquot of the reaction mixture was taken to determine the yield and regioisomeric ratio (r.r.) by crude ^{1}H NMR in $CDCl_3$. The product was purified by silica gel column chromatography (pentane) and the enantiomeric ratio (e.r.) was determined by chiral GC.

Table S1. Results of initial screening of catalysts

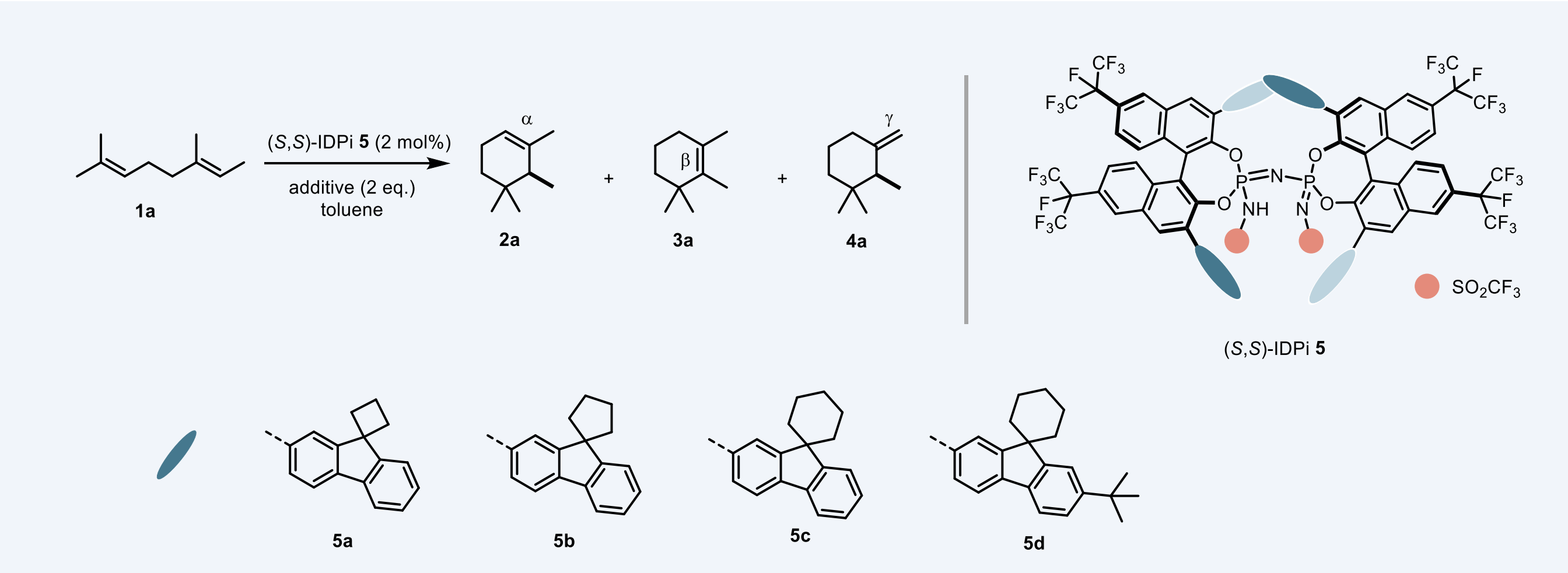

entry	IDPi	additive	solvent	temp.	yield (%)	**2a**	**3a**	**4a**	e.r. (**2a**)	e.r. (**4a**)
1	**5a**	PFTB	toluene	-50 °C	92	92	-	-	78:22	-
2	**5b**	PFTB	toluene	-50 °C	90	90	-	-	84:16	-
3	**5c**	PFTB	toluene	-50 °C	95	95	-	-	93:7	-
4	**5d**	PFTB	toluene	-50 °C	95	95	-	-	95:5	-

Table S2. Results of initial screening of additive

entry	IDPi	additive	solvent	temp.	yield (%)	**2a**	**3a**	**4a**	e.r. (**2a**)	e.r. (**4a**)
1	**5d**	PFTB	toluene	-50 °C	95	95	-	-	95.0:5.0	-
2	**5d**	HFIP	toluene	-50 °C	69	46	-	23	99:1	98:2
3	**5d**	TFE	toluene	-50 °C	64	20	-	44	97.0:3.0	98.5:1.5

Table S3. Results of screening of additive (eq.)

entry	IDPi	PFTB	solvent	temp.	yield (%)	**2a**	**3a**	**4a**	e.r. (**2a**)	e.r. (**4a**)
1	**5d**	0.02 eq.	toluene	-50 °C	trace	-	-	2	93:7	95.5:4.5
2	**5d**	0.06 eq.	toluene	-50 °C	9	2	-	7	93.5:6.5	95.0:5.0
3	**5d**	0.2 eq.	toluene	-50 °C	61	23	-	18	96:4	95:5
4	**5d**	0.5 eq.	toluene	-50 °C	94	88	-	6	96.5:3.5	-
5	**5d**	1 eq.	toluene	-50 °C	92	92	-	-	95:5	-
6	**5d**	2 eq.	toluene	-50 °C	95	95	-	-	95:5	-

Table S4. Results of screening of solvent

entry	IDPi	additive	solvent	temp.	yield (%)	**2a**	**3a**	**4a**	e.r. (**2a**)	e.r. (**4a**)
1	**5d**	PFTB	toluene	-50 °C	95	95	-	-	95:5	-
2	**5d**	PFTB	$CHCl_3$	-50 °C	91	91	-	-	96:4	-
3	**5d**	PFTB	THF	-50 °C	NR	-	-	-	-	-
4	**5d**	PFTB	*n*-hexane	-50 °C	94	94	-	-	96:4	-
5	**5d**	PFTB	Et_2O	-50 °C	NR	-	-	-	-	-
6	**5d**	PFTB	acetone	-50 °C	NR	-	-	-	-	-
7	**5d**	PFTB	EtOAc	-50 °C	NR	-	-	-	-	-
8	**5d**	PFTB	CH_3CN	-50 °C	NR	-	-	-	-	-
9	**5d**	PFTB	iPrOH	-50 °C	NR	-	-	-	-	-

3.2 Development of the catalytic asymmetric polyene cyclization

General Procedure: An oven-dried 5 mL vial was charged with IDPi catalyst (2 mol%) and a magnetic stir bar under an atmosphere of argon, 112 uL PFTB were added. The vial was cooled to required reaction temperature. Then **8a** (0.14 mmol, 1.0 eq.) was added and the reaction was stirred at desired temperature.

The reaction mixture was quenched with triethylamine (0.28 mmol, 2 eq.) at reaction temperature, then CH_2Br_2 (internal standard, 0.14 mmol, 1.0 eq.) was added. Then an aliquot of the reaction mixture was taken to determine the yield and regioisomeric ratio (r.r.) by crude ^1H NMR in $CDCl_3$. The product was purified by silica gel column chromatography and the enantiomeric ratio (e.r.) was determined by chiral GC or HPLC.

Table S5. Results of initial screening of catalysts

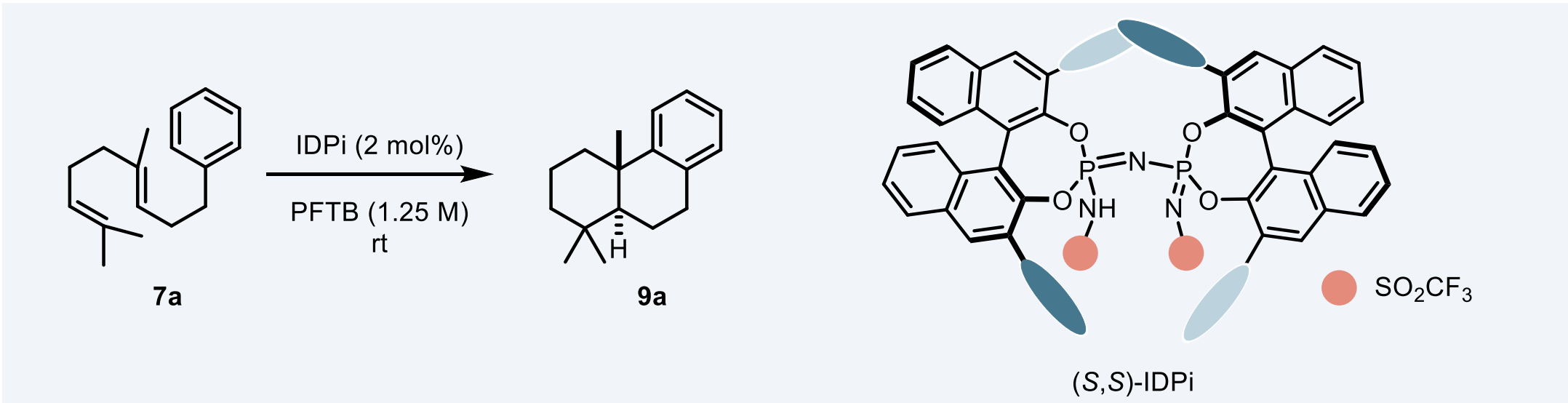

catalyst screening

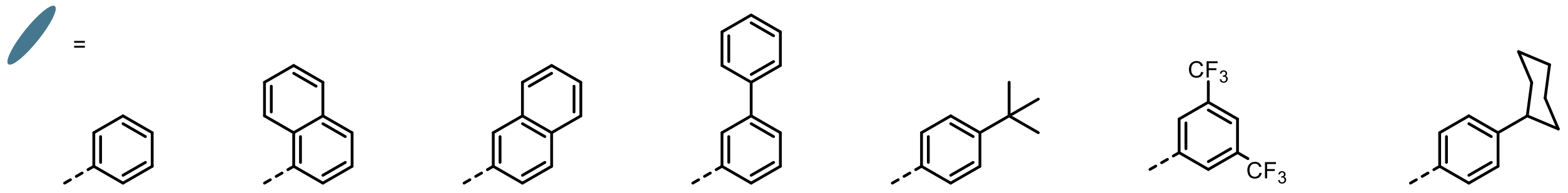

40%	35%	45%	45%	25%	51%	29%
47:53 e.r. (**9a**)	48:52 e.r. (**9a**)	45:55 e.r. (**9a**)	46:54 e.r. (**9a**)	47:53 e.r. (**9a**)	50:50 e.r. (**9a**)	48:52 e.r. (**9a**)

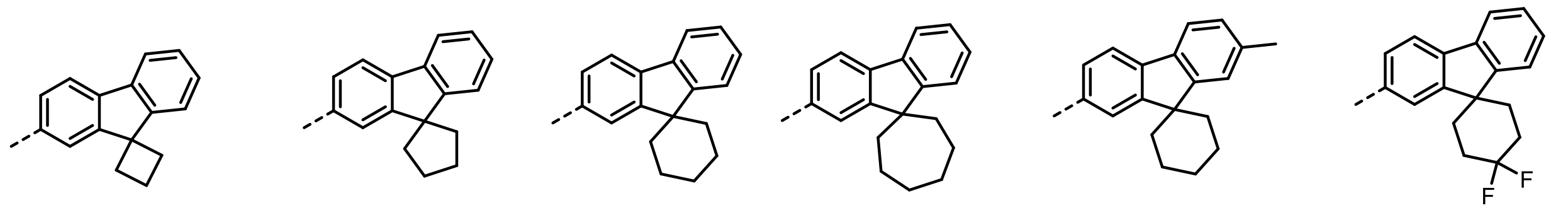

39%	36%	33%	45%	25%	51%
51:49 e.r. (**9a**)	50:50 e.r. (**9a**)	48:52 e.r. (**9a**)	49:51 e.r. (**9a**)	47:53 e.r. (**9a**)	42:58 e.r. (**9a**)

Table S6. Results of screening of solvent and temperature

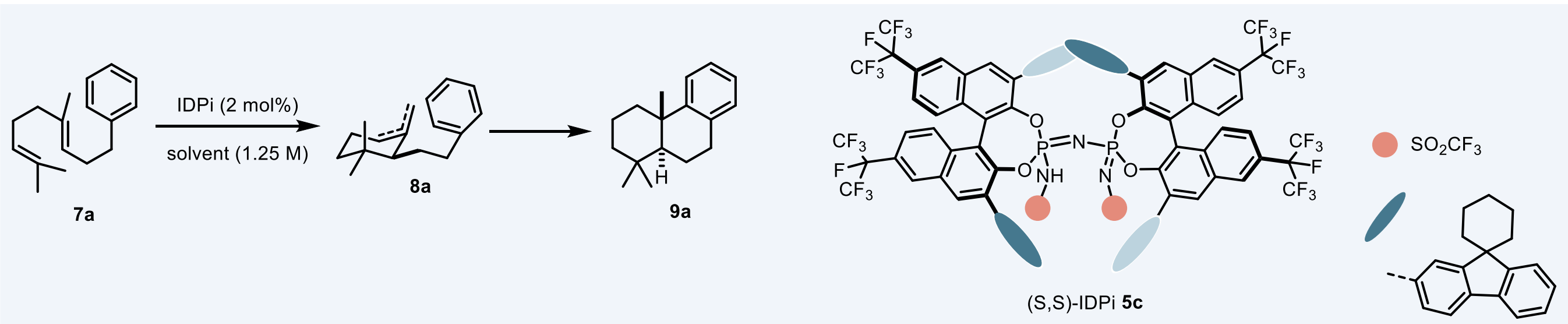

solvent screening

in PFTB, rt.	in HFIP, rt.	in TFE, rt.
12 h, 45%	12 h, 55%	12 h, 61%
>20:1 d.r.	>20:1 d.r.	>20:1 d.r.
60:40 e.r. (**9a**)	66:34 e.r. (**9a**)	72:28 e.r. (**9a**)

temperature screening

in TFE, -40 °C
48 h, 5%
>20:1 d.r.
85:15 e.r. (**9a**)

\+

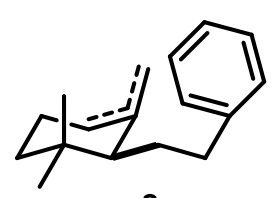

8a

78% yield
α:γ = 52:48
92:8 e.r. (α)
98:2 e.r. (γ)

in TFE, -40 °C, 48 h	in TFE, -40 °C, 48 h
then 0 °C. 24 h	then rt. 24 h,
78%, **8a**, (α:γ > 20:1)	70%, **9a**, >20:1 d.r.
94:6 e.r. (**8a**)	93:7 e.r. (**9a**)

Table S7. Results of screening of catalysts

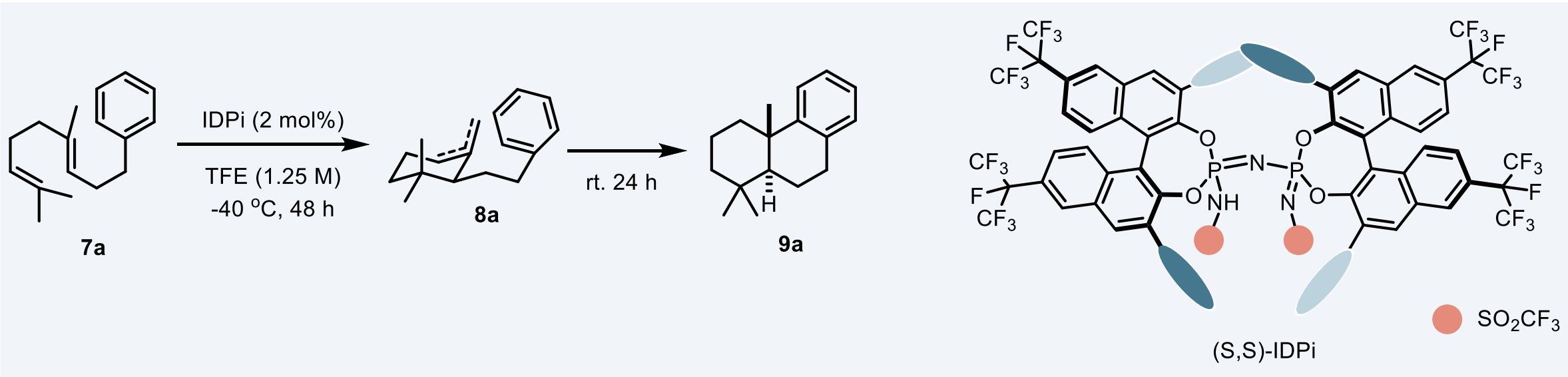

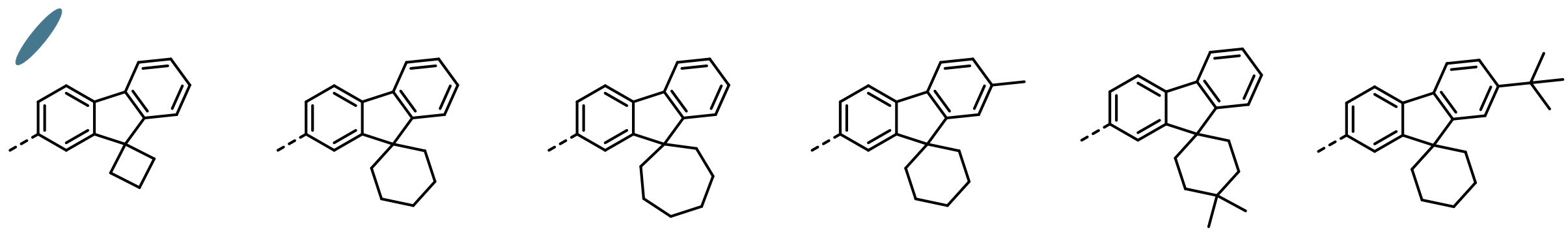

75% yield<br>>20:1 d.r.<br>88:12 e.r. (**9a**)	70% yield<br>>20:1 d.r.<br>93:7 e.r. (**9a**)	81% yield<br>>20:1 d.r.<br>77:23 e.r. (**9a**)	74% yield<br>>20:1 d.r.<br>86:14 e.r. (**9a**)	78% yield<br>>20:1 d.r.<br>76:24 e.r. (**9a**)	80% yield<br>>20:1 d.r.<br>95:5 e.r. (**9a**)

4. Catalytic asymmetric geranyl cyclization

General Procedure: A 5 ml vial was charged with IDPi **5d** (2.7 µmol, 2 mol%) and a magnetic stir bar under an atmosphere of argon. Dry toluene (0.107 mL) and PFTB (0.275 mmol, 2 eq.) added. The vial was cooled to −50 °C. The substrate **1** (0.137 mmol, 1.0 eq.) was added dropwise and the reaction was stirred at desired temperature. Afterwards, the reaction mixture was treated with triethylamine (0.257 mmol, 3.8 µL, 0.2 eq.). After the mixture was stirred for 10 min, the solvent of the reaction mixture was directly removed under reduced pressure. The product was purified by column chromatography on silica gel (pentane), then analyzed by ^{1}H NMR and chiral GC or chiral HPLC to determine regioisomeric ratio (r.r.) and the enantiomeric ratio (e.r.).

Racemate synthesis: The racemic product was synthesized under the above conditions via racemic IDPi **5d** catalysis.

(*S*)-1,5,5,6-tetramethylcyclohex-1-ene 2a

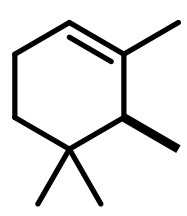

2a

At −50 °C for 3 d. 95% yield;

***R**$_F$* (pentane) = 0.9.

r.r. >20:1;

e.r. of **2a**: 95.0:5.0.

^{1}H NMR (600 MHz, $CDCl_3$): δ = 5.31–5.29 (m, 1H), 1.98–1.94 (m, 2H), 1.67–1.66 (m, 3H), 1.65–1.61 (m, 1H), 1.46–1.41 (m, 1H), 1.18–1.14 (m, 1H), 0.94 (s, 3H), 0.93 (s, 3H), 0.90 (s, 3H), 0.84 (s, 3H).

^{13}C NMR (151 MHz, $CDCl_3$): δ = 137.7, 120.0, 44.1, 31.94, 31.90, 27.6, 26.1, 23.2, 22.9, 14.8.

HRMS *m*/*z* (GC-EI): calculated for $C_{10}H_{18}^{+}$ ($[M]^{+}$): 138.14030; found: 138.14033.

Chiral GC (BGB 176 0.25/0.25df, 30.0 m, temperature: 220/ 80 iso 30 min, 5/min, 220 iso 5 min, 0.60 bar H_2, sample size: 0.2 µL): t_{R1} = 9.8 min (ent-**2a**), t_{R2} = 10.1 min (**2a**).
$[\alpha]_D^{25}$ = −32.7 (c = 0.60, $CHCl_3$).

(*S*)-6-ethyl-1,5,5-trimethylcyclohex-1-ene 2b

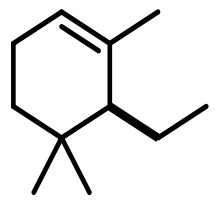

2b

At −50 °C for 3 d. 98% yield;
R

R_F (pentane) = 0.9.
2b:**4b** = 93:7;
e.r. of **2b**: 97:3.
e.r. of **4b**: 87:13.
^{1}H NMR (501 MHz, $CDCl_3$): δ = 5.30–5.29 (m, 0.93H), 4.75–4.74 (m, 0.07H), 4.534–4.532 (m, 0.07H), 1.96–1.94 (m, 2H) 1.68–1.67 (m, 3H), 1.54–1.34 (m, 4H), 1.13–1.09 (m, 1H), 0.95–0.91 (m, 6H), 0.86 (s, 3H).
^{13}C NMR (126 MHz, $CDCl_3$): δ = 136.8, 120.1, 51.0, 32.7, 31.9, 27.9, 27.6, 23.74, 23.71, 23.2, 14.8.
HRMS *m/z* (GC-EI): calculated for $C_{11}H_{20}^+$ ($[M]^+$): 152.15595; found: 152.15593.
GC (Chiral) (BGB 176 0.25/0.25df, 30.0 m, temperature: 220/80 iso 30 min, 5/min, 220 iso 5 min, 0.60 bar H_2, sample size: 0.2 µL): t_{R1} = 12.3 min (ent-**4b**), t_{R2} = 12.6 min (**4b**), t_{R3} = 13.5 min (**2a**), t_{R4} = 14.3 min (ent-**2a**).
$[\alpha]_D^{25}$ = −67.4 (c = 1.30, $CHCl_3$).

(*S*)-1,5,5-trimethyl-6-pentylcyclohex-1-ene 2c

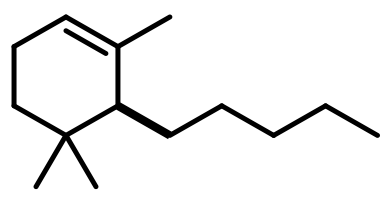

2c

At −50 °C for 3 d. 88% yield;
R_F (pentane) = 0.9.
2c:**4c** = 94:6;
e.r. of **2c**: 96.5:3.5.
e.r. of **4c**: 96.5:3.5.
^{1}H NMR (501 MHz, $CDCl_3$): δ = 5.264–5.262 (m, 0.94H), 4.74–4.73 (m, 0.06H), 4.53–4.52 (m, 0.06H), 1.96–1.93 (m, 2H) 1.674–1.671 (m, 3H), 1.46–1.21 (m, 10H), 1.13–1.08 (m, 1H), 0.90–0.86 (m, 9H).
^{13}C NMR (126 MHz, $CDCl_3$): δ = 137.3, 119.7, 49.6, 32.9, 32.7, 31.7, 31.5, 30.4, 27.7, 27.5, 23.7, 23.2, 22.8, 14.3.
HRMS *m/z* (GC-CI): calculated for $C_{14}H_{30}N^+$ ($[M+NH_4]^+$): 212.23727; found: 212.23733.
GC (Chiral) (Ivadex-7/PS086 0.25/0.15df, 25.0 m, temperature: 220/60 iso 110 min, 8/min, 220 iso 5 min, 0.60 bar H_2, sample size: 0.2 µL): t_{R1} = 70.6 min (ent-**4c**), t_{R2} = 73.1 min (**4c**), t_{R3} = 84.4 min (**2c**), t_{R4} = 104.9 min (ent-**2c**).
$[\alpha]_D^{25}$ = −70.9 (c = 1.84, $CHCl_3$).

(*S*)-1,5,5-trimethyl-6-nonylcyclohex-1-ene 2d

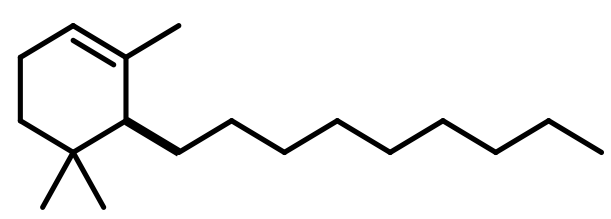

2d

At −50 °C for 3 d. 95% yield;

R_F (pentane) = 0.9.

2d:**4d** = 89:11;

e.r. of **2d**: 96:4.

e.r. of **4d**: 99:1.

^{1}H NMR (501 MHz, $CDCl_3$): δ = 5.27–5.25 (m, 0.89H), 4.74–4.73 (m, 0.11H), 4.53–4.52 (m, 0.11H), 1.97–1.93 (m, 2H) 1.68–1.67 (m, 3H), 1.46–1.25 (m, 18H), 1.13–1.08 (m, 1H), 0.90–0.86 (m, 9H).

^{13}C NMR (126 MHz, $CDCl_3$): δ = 137.3, 119.7, 49.7, 32.7, 32.1, 31.7, 31.5, 30.8, 30.7, 29.9, 29.8, 29.5, 27.7, 27.5, 23.7, 23.2, 22.9, 14.3.

HRMS *m*/*z* (GC-CI): calculated for $C_{18}H_{38}N^+$ ($[M+NH_4]^+$): 268.29987; found: 268.29985.

GC (Chiral) (G-TA 0.25/0.15df, 30.0 m, temperature: 220/65 iso 1000 min, 8/min, 220 iso 5 min, 0.60 bar H_2, sample size: 0.2 µL): t_{R1} = 824.6 min (**4d**), t_{R2} = 872.2 min (ent-**4d**), t_{R3} = 908.3 min (**2d**), t_{R4} = 955.0 min (ent-**2d**).

$[\alpha]_D^{25}$ = −68.9 (*c* = 0.84, $CHCl_3$).

(*S*)-6-isopentyl-1,5,5-trimethylcyclohex-1-ene 2e

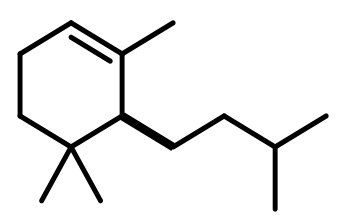

2e

At −50 °C for 3 d. 95% yield;

R_F (pentane) = 0.9.

2e:**4e** = 92:8;

e.r. of **2e**: 96.5:3.5.

e.r. of **4e**: 97:3.

^{1}H NMR (501 MHz, $CDCl_3$): δ = 5.28–5.26 (m, 0.92H), 4.73 (d, *J* = 2.6 Hz, 0.08H), 4.52 (d, *J* = 2.6 Hz, 0.08H), 1.97–1.88 (m, 3H), 1.67 (s, 2.91H), 1.53–1.37 (m, 3H), 1.24–1.22 (m, 3H), 1.13–1.09 (m, 1H), 0.92–0.86 (m, 12H).

^{13}C NMR (126 MHz, $CDCl_3$): δ = 137.3, 119.7, 49.8, 40.1, 32.7, 31.8, 29.1, 29.0, 28.8, 27.7, 27.6, 23.6, 23.2, 22.81, 22.78, 22.7.

HRMS *m*/*z* (GC-EI): calculated for $C_{14}H_{26}^+$ ($[M]^+$): 194.20290; found: 194.20283.

Chiral GC (Lipodex-G 0.25/0.25df, 25.0 m, temperature: 220/70 iso 27 min, 8/min, 220 iso 5 min, 0.60 bar H_2, sample size: 0.2 µL): t_{R1} = 19.3 min (**4e**), t_{R2} = 20.2 min (ent-**4e**), t_{R3} = 21.7 min (**2e**), t_{R4} = 23.8 min (ent-**2e**).

$[\alpha]_D^{25}$ = −57.5 (*c* = 0.4, $CHCl_3$).

(*S*)-1,5,5-trimethyl-6-neopentylcyclohex-1-ene 2f

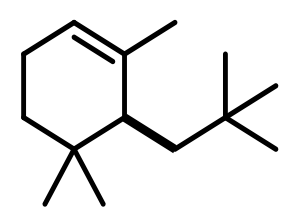

2f

At −50 °C for 3 d. 83% yield;

R_F (pentane) = 0.9.

r.r. >20:1;

e.r. of **2f**: 95:5.

^{1}H NMR (501 MHz, $CDCl_3$): δ = 5.19–5.18 (m, 1H), 1.96–1.92 (m, 2H) 1.71–1.70 (m, 3H), 1.53–1.38 (m, 3H), 1.08-1.04 (m, 2H), 0.92 (s, 9H), 0.88 (s, 6H)

^{13}C NMR (126 MHz, $CDCl_3$): δ = 139.6, 119.2, 45.6, 44.7, 33.0, 31.2, 31.0, 30.9, 28.8, 27.2, 24.8, 23.3.

HRMS *m/z* (GC-EI): calculated for $C_{14}H_{26}^+$ ($[M]^+$): 194.20290; found: 194.20260.

Chiral GC (BGB 176 0.25/0.25df, 30.0 m, temperature: 220/100 iso 30 min, 5/min, 220 iso 5 min, 0.60 bar H_2, sample size: 0.2 µL): t_{R1} = 22.3 min (**2f**), t_{R2} = 24.4 min (ent-**2f**).

$[\alpha]_D^{25}$ = −96.5 (c = 0.60, $CHCl_3$).

(*S*)-2,2,6-trimethyl-1,2,3,4-tetrahydro-1,1'-biphenyl 2g

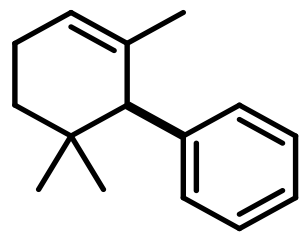

2g

At −50 °C for 3 d. 88% yield;

R_F (pentane) = 0.9.

r.r. >20:1;

e.r. of **2g**: 97.5:2.5.

^{1}H NMR (501 MHz, $CDCl_3$): δ = 7.29–7.26 (m, 2H), 7.23–7.18 (m, 3H), 5.63–5.61 (m, 1H), 2.79 (s, 1H), 2.17–2.15 (m, 2H), 1.56–1.51 (m, 4H), 1.16–1.11 (m, 1H), 1.05 (s, 3H), 0.59 (s, 3H).

^{13}C NMR (126 MHz, $CDCl_3$): δ = 142.5, 134.6, 127.6, 126.1, 121.8, 57.5, 32.9, 30.5, 28.9, 28.1, 23.6, 23.1.

HRMS *m/z* (GC-EI): calculated for $C_{15}H_{20}^+$ ($[M]^+$): 200.15595; found: 200.15583.

Chiral GC (Ivadex-1 0.25/0.25df, 25.0 m, temperature: 220/100 iso 30 min, 5/min, 220 iso 5 min, 0.60 bar H_2, sample size: 0.2 µL): t_{R1} = 21.4 min (**2g**), t_{R2} = 21.9 min (ent-**2g**).

$[\alpha]_D^{25}$ = −85.5 (c = 0.68, $CHCl_3$).

(*S*)-2,2,4',6-tetramethyl-1,2,3,4-tetrahydro-1,1'-biphenyl 2h

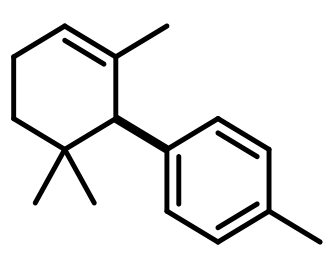

2h

At −50 °C for 3 d. 93% yield;

R_F (pentane) = 0.9.

r.r. >20:1;

e.r. of **2h**: 97.5:2.5.

^{1}H NMR (501 MHz, $CDCl_3$): δ = 7.07–7.03 (m, 4H), 5.58–5.56 (m, 1H), 2.72 (s, 1H), 2.14–2.10 (m, 2H), 1.52–1.46 (m, 4H), 1.12–1.07 (m, 1H), 1.01 (s, 3H), 0.56 (s, 3H).

^{13}C NMR (126 MHz, $CDCl_3$): δ = 139.4, 135.6, 134.8, 128.4, 121.6, 57.1, 32.8, 30.5, 28.9, 23.6, 23.2, 21.2.

HRMS *m/z* (GC-EI): calculated for $C_{16}H_{22}^+$ ($[M]^+$): 214.17160; found: 214.17146.

Chiral HPLC (150 mm, OJ-3R, 4.6 mm; MeOH/H_2O = 95:5; Flow rate = 1 mL/min; λ = 220 nm): t_{R1} = 6.0 min (**2h**), t_{R2} = 8.5 min (ent-**2h**).

$[\alpha]_D^{25}$ = −71.5 (*c* = 0.47, $CHCl_3$).

(*S*)-4'-methoxy-2,2,6-trimethyl-1,2,3,4-tetrahydro-1,1'-biphenyl 2i

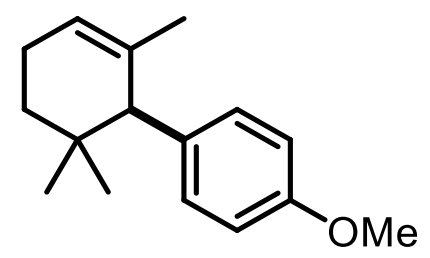

2i

At −50 °C for 3 d, then at rt for 6 h. 90% yield;

R_F (pentane/Et_2O 10:1) = 0.7.

r.r. >20:1;

e.r. of **2i**: 98:2.

^{1}H NMR (501 MHz, $CDCl_3$): δ = 7.08 (d, *J* = 8.7 Hz, 2H), 6.81 (d, *J* = 8.7 Hz, 2H), 5.57–5.55 (m, 1H), 3.79 (s, 3H), 2.70 (s, 1H), 2.13–2.10 (m, 2H), 1.50–1.46 (m, 4H), 1.12–1.07 (m, 1H), 1.01 (s, 3H), 0.56 (s, 3H).

^{13}C NMR (126 MHz, $CDCl_3$): δ = 158.1, 134.9, 134.6, 131.3, 121.6, 112.9, 56.6, 55.3, 32.9, 30.5, 28.9, 27.9, 23.6, 23.1.

HRMS *m/z* (GC-EI): calculated for $C_{16}H_{22}O^+$ ($[M]^+$): 230.16651; found: 230.16654.

Chiral HPLC (150 mm, OJ-3R, 4.6 mm; MeOH/H_2O = 95:5; Flow rate = 1 mL/min; λ = 220 nm): t_{R1} = 6.0 min (**2i**), t_{R2} = 7.6 min (ent-**2i**).

$[\alpha]_D^{25}$ = −99.7 (*c* = 0.80, $CHCl_3$).

(*S*)-4'-fluoro-2,2,6-trimethyl-1,2,3,4-tetrahydro-1,1'-biphenyl 2j

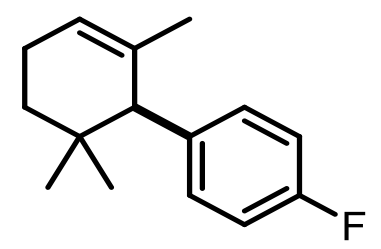

2j

At −50 °C for 3 d, then at rt for 6 h. 86% yield;

R_F (pentane) = 0.8.

r.r. >20:1;

e.r. of **2j**: 98:2.

¹H NMR (501 MHz, $CDCl_3$): δ = 7.12–7.10 (m, 2H), 6.96–6.92 (m, 2H), 5.584–5.581 (m, 1H), 2.74 (s, 1H), 2.14–2.11 (m, 2H), 1.48–1.42 (m, 4H), 1.13–1.08 (m, 1H), 1.01 (s, 3H), 0.55 (s, 3H).

¹³C NMR (126 MHz, $CDCl_3$): δ = 162.6 (d, J = 243.6 Hz), 138.1 (d, J = 3.1 Hz), 134.5, 131.7 (d, J = 7.6 Hz), 122.0, 114.5 (d, J = 20.8 Hz), 56.7, 32.8, 30.3, 28.9, 28.0, 23.5, 23.1.

¹⁹F NMR (471 MHz, $CDCl_3$) δ = −117.7.

HRMS *m/z* (GC-CI): calculated for $C_{15}H_{19}F^+$ ($[M]^+$): 218.14653; found: 218.14668.

Chiral HPLC (150 mm, OJ-3R, 4.6 mm; MeOH/H_2O = 95:5; Flow rate = 1 mL/min; λ = 220 nm): t_{R1} = 4.2 min (**2j**), t_{R2} = 5.2 min (ent-**2j**).

$[\alpha]_D^{25}$ = −92.4 (c = 0.61, $CHCl_3$).

(S)-4'-chloro-2,2,6-trimethyl-1,2,3,4-tetrahydro-1,1'-biphenyl 2k

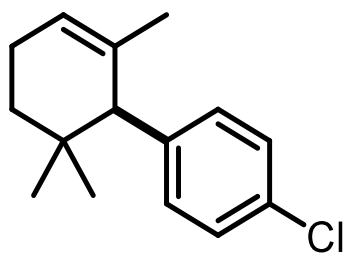

2k

At −50 °C for 3 d, then at rt for 6 h. 84% yield;

$\boldsymbol{R_F}$ (pentane) = 0.8.

r.r. >20:1;

e.r. of **2k**: 96.5:3.5.

¹H NMR (501 MHz, $CDCl_3$): δ = 7.23 (d, J = 8.4 Hz, 2H), 7.10 (d, J = 8.4 Hz, 2H), 5.60–5.59 (m, 1H), 2.73 (s, 1H), 2.14–2.11 (m, 2H), 1.47–1.41 (m, 4H), 1.14–1.09 (m, 1H), 1.01 (s, 3H), 0.55 (s, 3H).

¹³C NMR (126 MHz, $CDCl_3$): δ = 141.1, 134.2, 131.9, 131.7, 127.8, 122.2, 56.9, 32.8, 30.4, 28.9, 28.0, 23.5, 23.1.

HRMS *m/z* (GC-EI): calculated for $C_{15}H_{19}Cl^+$ ($[M]^+$): 234.11698; found: 234.11711.

Chiral HPLC (150 mm, OJ-3R, 4.6 mm; MeOH/H_2O = 90:10; Flow rate = 1 mL/min; λ = 220 nm): t_{R1} = 7.9 min (**2k**), t_{R2} = 11.1 min (ent-**2k**).

$[\alpha]_D^{25}$ = −27.7 (c = 0.32, $CHCl_3$).

(*S*)-4'-bromo-2,2,6-trimethyl-1,2,3,4-tetrahydro-1,1'-biphenyl 2l

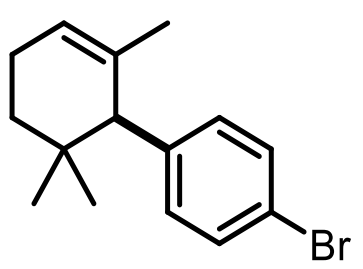

2l

At −50 °C for 3 d, then at rt for 6 h. 81% yield;

$\boldsymbol{R_F}$ (pentane) = 0.8.

r.r. >20:1;

e.r. of **2l**: 96.5:3.5.

¹H NMR (501 MHz, $CDCl_3$): δ = 7.38 (d, J = 8.4 Hz, 2H), 7.05 (d, J = 8.4 Hz, 2H), 5.60 (m, 1H), 2.72 (s, 1H), 2.13–2.11 (m, 2H), 1.47–1.40 (m, 4H), 1.14–1.09 (m, 1H), 1.01 (s, 3H), 0.55 (s, 3H).

^{13}C NMR (126 MHz, $CDCl_3$): δ = 141.6, 134.2, 132.1, 130.8, 122.3, 120.0, 56.9, 32.8, 30.4, 28.9, 28.0, 23.5, 23.1.
HRMS *m*/*z* (GC-EI): calculated for $C_{15}H_{19}Br^+$ ($[M]^+$): 278.06647; found: 278.06654.
Chiral HPLC (150 mm, OJ-3R, 4.6 mm; MeOH/H_2O = 90:10; Flow rate = 1 mL/min; λ = 220 nm): t_{R1} = 8.4 min (**2l**), t_{R2} = 12.2 min (ent-**2l**).
$[\alpha]_D^{25}$ = −54.5 (c = 0.69, $CHCl_3$).

(*S*)-((2,6,6-trimethylcyclohex-2-en-1-yl)methyl)benzene 2m

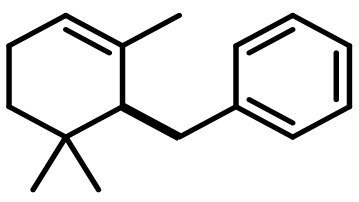

2m

At −50 °C for 7 d, then at rt for 6 h. 91% yield;
***R**$_F$* (pentane) = 0.8.
r.r. >20:1;
e.r. of **2m**: 86:14.
^{1}H NMR (501 MHz, $CDCl_3$): δ = 7.27–7.21 (m, 4H), 7.17–7.14 (m, 1H), 5.32–5.30 (m, 1H), 2.87 (dd, *J* = 14.3, 5.6 Hz, 1H), 2.54 (dd, *J* = 14.3, 5.6 Hz, 1H), 2.02–1.98 (m, 2H), 1.94–1.92 (m, 1H), 1.52–1.45 (m, 4H), 1.19–1.14 (m, 1H), 0.90 (s, 6H).
^{13}C NMR (126 MHz, $CDCl_3$): δ = 143.6, 137.0, 129.2, 128.2, 125.5, 120.6, 51.5, 37.9, 33.1, 31.1, 28.1, 27.4, 24.2, 23.3.
HRMS *m*/*z* (GC-EI): calculated for $C_{16}H_{22}^+$ ($[M]^+$): 214.17160; found: 214.17141.
Chiral HPLC (150 mm, OJ-3R, 4.6 mm; MeOH/H_2O = 95:5; Flow rate = 1 mL/min; λ = 220 nm): t_{R1} = 4.8 min (ent-**2m**), t_{R2} = 5.1 min (**2m**).
$[\alpha]_D^{25}$ = −105.5 (c = 0.62, $CHCl_3$).

(*S*)-(2-(2,6,6-trimethylcyclohex-2-en-1-yl)ethyl)benzene 2n

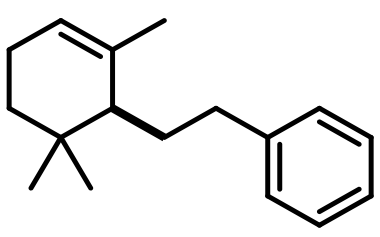

2n

At −50 °C for 7 d, 88% yield;
***R**$_F$* (pentane) = 0.8.
2n:**4n** = 93:7;
e.r. of **2n**: 89.5:10.5.
e.r. of **4n**: 91:9.
(**2n**:**4n** = 52:48) **^{1}H NMR** (501 MHz, $CDCl_3$): δ = 7.32–7.28 (m, 2H), 7.22–7.20 (m, 3H), 5.354–5.351 (m, 0.52H), 4.87–7.86 (m, 0.48H), 4.663–4.662 (m, 0.48H), 2.70–2.61 (m, 1.48H), 2.42–2.35 (m, 0.52H), 2.10–1.99 (m, 2H), 1.84–1.48 (m, 6.52H), 1.27–1.16 (m, 1H), 1.02 (s, 1.56H), 0.95 (s, 1.44H), 0.92 (s, 1.44H), 0.86 (s, 1.56H).
^{13}C NMR (126 MHz, $CDCl_3$): δ = 149.4, 143.4, 136.6, 128.53, 128.52, 128.44, 128.42, 125.8, 125.7, 120.4, 109.3, 54.0, 49.2, 36.8, 36.3, 35.0, 34.7, 33.6, 32.8, 32.6, 31.8, 28.8, 28.5, 27.7, 27.6, 26.5, 23.9, 23.6, 23.2.

HRMS *m/z* (GC-CI): calculated for $C_{17}H_{28}N^+$ ($[M+NH_4]^+$) 246.22162; found: 246.22143.
Chiral GC (Lipodex-G 0.25/0.25df, 25.0 m, temperature: 220/80 iso 450 min, 10/min, 220 iso 5 min, 0.60 bar H_2, sample size: 0.2 µL): t_{R1} = 280.0 min (**4n**), t_{R2} = 287.6 min (ent-**4n**), t_{R3} = 321.8 min (**2n**), t_{R4} = 346.6 min (ent-**2n**).
$[\alpha]_D^{25}$ = −71.9 (c = 0.72, $CHCl_3$).

(*S*)-1-ethyl-5,5,6-trimethylcyclohex-1-ene 2o

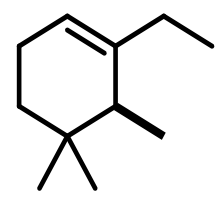

2o

At −50 °C for 3 d, 88% yield;
***R*$_F$** (pentane) = 0.9.
2o:**4o** = 72:28;
e.r. of **2o**: 96.5:3.5.
^{1}H NMR (501 MHz, $CDCl_3$): δ = 5.28–5.26 (m, 0.72H), 5.11–5.07 (m, 0.28 H), 2.03–1.90 (m, 4H), 1.60–1.59 (m, 0.84H), 1.50–1.43 (m, 1.44H), 1.29–1.25 (m, 0.28H), 1.16–1.11 (m, 0.72H), 0.98–0.72 (m, 11.72H)
^{13}C NMR (126 MHz, $CDCl_3$): δ = 143.5, 142.4, 118.0, 114.7, 48.6, 42.2, 38.9, 31.8, 31.3, 29.4, 28.6, 27.3, 26.9, 26.7, 23.4, 23.3, 23.2, 15.3, 13.5, 13.1, 13.0.
HRMS *m/z* (GC-EI): calculated for $C_{11}H_{20}^+$ ($[M]^+$): 152.15595; found: 152.15594.
Chiral GC (Ivadex-1 0.25/0.25df, 25.0 m, temperature: 220/60 iso 30 min, 5/min, 220 iso 5 min, 0.60 bar H_2, sample size: 0.2 µL): t_{R1} = 8.7 min (ent-**2o**), t_{R2} = 9.0 min (**2o**).
$[\alpha]_D^{25}$ = −45.0 (c = 0.44, $CHCl_3$).

(*S*)-2,3,3-trimethyl-2,3,4,5-tetrahydro-1,1'-biphenyl 2p

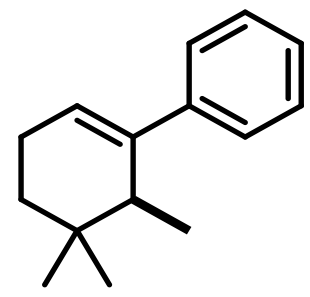

2p

At −50 °C for 3 d, 56% yield; r.r. >20:1;
***R*$_F$** (pentane) = 0.7.
e.r. of **2p**: 84:16.
^{1}H NMR (501 MHz, $CDCl_3$): δ = 7.23–7.09 (m, 5H), 5.72 (t, J = 3.7 Hz, 1H), 2.27–2.24 (m, 1H), 2.11–2.08 (m, 2H), 1.52–1.49 (m, 1H), 1.18–1.13 (m, 1H), 0.94 (s, 3H), 0.89 (s, 3H), 0.78 (d, J = 7.5 Hz, 3H).
^{13}C NMR (126 MHz, $CDCl_3$): δ = 128.3, 126.5, 126.4, 123.7, 41.8, 32.0, 30.3, 27.7, 27.1, 23.8, 16.1.
HRMS *m/z* (GC-EI): calculated for $C_{15}H_{20}^+$ ($[M]^+$): 200.15595; found: 200.15586.
Chiral GC (BGB 176 0.25/0.25df, 30.0 m, temperature: 220/150 iso 60 min, 5/min, 220 iso 5 min, 0.60 bar H_2, sample size: 0.2 µL): t_{R1} = 14.9 min (ent-**2p**), t_{R2} = 15.7 min (**2p**).
$[\alpha]_D^{25}$ = −58.3 (c = 0.69, $CHCl_3$).

(*S*)-cyclohomogeranates 2r + 4r

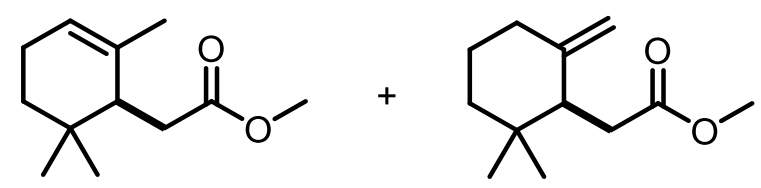

2r 4r

At −50 °C for 15 d. 78% yield;

2r:**4r** = 25:75.

e.r. of **2r**: 76:24.

e.r. of **4r**: 99:1.

(**2r**:**4r** = 50:50) **^{1}H NMR** (501 MHz, $CDCl_3$): δ = 5.35–5.34 (m, 1H), 4.75 (s, 1H), 4.55 (s, 1H), 3.68 (s, 3H), 3.63 (s, 3H), 2.52–2.39 (m, 4H), 2.23–2.19 (m, 3H), 2.08–1.99 (m, 4H), 1.65–1.64 (m, 3H), 1.61–1.53 (m, 2H), 1.37–1.19 (m, 4H), 0.96 (s, 3H), 0.91 (s, 3H), 0.83 (s, 3H), 0.79 (s, 3H).

^{13}C NMR (126 MHz, $CDCl_3$): δ = 175.1, 174.3, 149.1, 135.3, 121.6, 108.6, 51.8, 51.7, 49.9, 45.8, 38.4, 35.8, 35.0, 34.2, 33.4, 32.3, 31.4, 28.9, 28.1, 27.0, 26.3, 23.9, 23.7, 23.0, 22.8.

Chiral GC (BGB 177 0.25/0.25df, 30.0 m, temperature: 220/ 50 iso 900 min, 5/min, 220 iso 5 min, 0.60 bar H_2, sample size: 0.2 μL): t_{R1} = 676.9 min (**2r**), t_{R2} = 725.4 min (**4r**), t_{R3} = 743.0 min (ent-**4r**), t_{R4} = 954.8 min (ent-**2r**).

(*S*)-ionone 2s

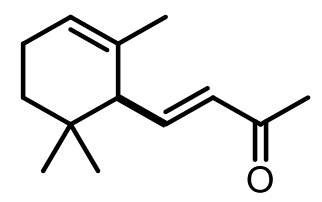

2s

At 0 °C for 4 d, then at 40 °C for 2d. 80% yield;

R_F (pentane/Et_2O 10:1) = 0.7.

2s:**4s** = 94:6;

e.r. of **2s**: 70.5:29.5.

e.r. of **4s**: 89.5:10.5.

^{1}H NMR (501 MHz, $CDCl_3$): δ =6.64 (dd, *J* = 15.8, 9.7 Hz, 1H), 6.06 (d, *J* = 15.8 Hz, 1H), 5.51–5.49 (m, 1H), 2.29–2.27 (m, 1H), 2.25 (s, 3H), 2.07–2.03 (m, 2H), 1.56 (s, 3H), 1.49–1.43 (m, 1H), 1.25–1.20 (m, 1H), 0.93 (s, 3H), 0.85 (s, 3H).

^{13}C NMR (126 MHz, $CDCl_3$): δ = 198.7, 149.3, 132.5, 132.1, 122.8, 54.4, 32.7, 31.3, 28.0, 27.1, 26.9, 23.2, 22.9.

Chiral GC (BGB 176 0.25/0.25df, 30.0 m, temperature: 220/ 100 iso 30 min, 5/min, 220 iso 5 min, 0.60 bar H_2, sample size: 0.2 μL): t_{R1} = 40.59 min (**2s**), t_{R2} = 41.2 min (ent-**2s**), t_{R3} = 41.3 min (ent-**4s**), t_{R4} = 41.5 min (**4s**).

5 Catalytic asymmetric polyene cyclization to polycyclic terpenoid

General Procedure: A 5 mL vial equipped with a magnetic stir bar was charged with IDPi **5d** (2 mol%) under an argon atmosphere. TFE (112 μL) was added, and the vial was cooled to −40 °C. Substrate **7** (0.14 mmol, 1.0 eq.) was added dropwise, and the reaction mixture was stirred at −40 °C until complete consumption of the starting material

was confirmed by TLC. The reaction afforded the monocyclized product **8**, which was used directly in the subsequent cyclization step without further purification to furnish the fully cyclized product **9**.

Second cyclization (Method A): The reaction mixture was allowed to warm to room temperature and stirred for 24 h. The reaction was then quenched with triethylamine (0.28 mmol, 2 eq.) at the reaction temperature, followed by the addition of CH_2Br_2 (0.14 mmol, 1.0 eq.) as an internal standard. An aliquot of the crude mixture was taken to determine the yield and regioisomeric ratio (r.r.) by ^{1}H NMR spectroscopy in $CDCl_3$. The product was subsequently purified by silica gel column chromatography, and the enantiomeric ratio (e.r.) was determined by chiral GC or HPLC.

Second cyclization (Method B): The solvent was removed under reduced pressure, and the resulting mixture was dissolved in *i*-$PrNO_2$ (0.26 M). TFA (10 eq.) and a solution of $SnCl_4$ (2 eq.) in hexane were then added at –70 °C. The reaction mixture was stirred at –70 °C for 24 h. Upon completion, the reaction was quenched with saturated aqueous $NaHCO_3$ and extracted with Et_2O (5 mL × 3). The combined organic layers were dried over Na_2SO_4, filtered, and concentrated under reduced pressure. The residue was purified by column chromatography on silica gel using pentane as the eluent. The product was then analyzed by ^{1}H NMR spectroscopy and chiral GC or HPLC to determine the regioisomeric ratio (r.r.) and enantiomeric ratio (e.r.).

(4a*S*,10a*S*)-1,1,4a-trimethyl-1,2,3,4,4a,9,10,10a-octahydrophenanthrene 9a

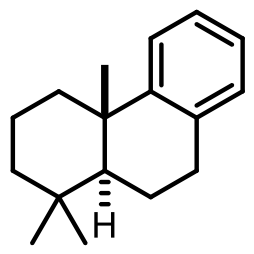

The second cyclization was performed according to **Method A**, 80% yield;

R

e.r.: 95.0:5.0.

^{1}H NMR (501 MHz, $CDCl_3$): δ = 7,.27–7.26 (m, 1H), 7.14–7.11 (m, 1H), 7.09–7.03 (m, 2H), 2.98–2.83 (m, 2H), 2.32–2.29 (m, 1H), 1.92–1.87 (m, 1H), 1.78–1.68 (m, 2H), 1.64–1.60 (m, 1H), 1.51–1.47 (m, 1H), 1.44–1.38 (m, 1H), 1.37–1.34 (m, 1H), 1.26–1.23 (m, 1H), 1.20 (s, 3H), 0.96 (s, 3H), 0.94 (s, 3H).

^{13}C NMR (126 MHz, $CDCl_3$): δ = 150.3, 135.4, 129.1, 125.7, 125.3, 124.5, 50.5, 41.9, 39.0, 38.0, 33.6, 33.5, 30.5, 25.0, 21.8, 19.5, 19.2.

HRMS *m*/*z* (GC-EI): calculated for $C_{17}H_{24}^+$ ($[M]^+$): 228.18725; found: 228.18717.

Chiral GC (Ivadex-1 0.25/0.25df, 25.0 m, temperature: 220/ 150 iso 20 min, 1/min, 220 iso 5 min, 0.60 bar H_2, sample size: 0.2 μL): t_{R1} = 28.5 min (ent-**9a**), t_{R2} = 28.83 min (**9a**).

$[\alpha]_D^{25}$ = +24.0 (*c* = 0.70, $CHCl_3$). Literature data for **9a** enantiomer:[7] (91% ee) $[\alpha]_D^{25}$ = +16.5 (c = 1.00, $CHCl_3$).

(4a*S*,10a*S*)-1,1,4a,6-tetramethyl-1,2,3,4,4a,9,10,10a-octahydrophenanthrene 9b

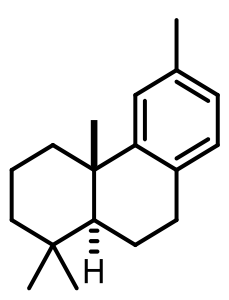

The second cyclization was performed according to **Method A**, 68% yield;

$\boldsymbol{R_F}$ (pentane) = 0.7.

e.r.: 97:3.

^{1}H NMR (501 MHz, $CDCl_3$): δ = 7.07 (s, 1H), 6.95 (d, *J* = 7.7 Hz, 1H), 6.91 (d, *J* = 7.7 Hz, 1H), 2.94–2.79 (m, 2H), 2.32–2.28 (m, 4H), 1.89–1.85 (m, 1H), 1.78–1.67 (m, 2H), 1.63–1.59 (m, 1H), 1.50–1.47 (m, 1H), 1.43 (td, *J* = 13.1, 3.8 Hz, 1H), 1.35 (d, *J* = 2.4 Hz, 0.5H), 1.33 (d, *J* = 2.4 Hz, 0.5H), 1.25–1.22 (m, 1H), 1.19 (s, 3H), 0.95 (s, 3H), 0.94 (s, 3H).

^{13}C NMR (126 MHz, $CDCl_3$): δ = 150.1, 134.9, 132.2, 129.1, 126.2, 125.1, 50.5, 41.9, 39.0, 37.9, 33.6, 33.5, 30.2, 25.0, 21.8, 21.4, 19.5, 19.2.

HRMS *m/z* (GC-EI): calculated for $C_{18}H_{26}^+$ ($[M]^+$): 242.20290; found: 242.20293.

Chiral HPLC (150 mm, OJ-3R, 4.6 mm; MeCN/H_2O = 70:30; Flow rate = 1 mL/min; λ = 220 nm): t_{R1} = 9.5 min (**9b**), t_{R2} = 16.8 min (ent-**9b**).

$[\alpha]_D^{25}$ = +36.5 (*c* = 0.70, $CHCl_3$).

(4a*S*,10a*S*)-6-isopropyl-1,1,4a-trimethyl-1,2,3,4,4a,9,10,10a-octahydrophenanthrene 9c

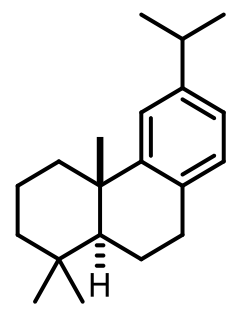

The second cyclization was performed according to **Method A**, 80% yield;

R_F (pentane) = 0.7.

e.r.: 91.5:8.5.

^{1}H NMR (501 MHz, $CDCl_3$): δ = 7.14 (s, 1H), 6.99-6.98 (m, 2H), 2.92–2.85 (m, 1H), 2.35-2.32 (m, 1H), 1.91–1.86 (m, 1H), 1.80-1.61 (m, 1H), 1.51–1.48 (m, 1H), 1.46–1.40 (m, 1H), 1.38–1.36 (m, 1H), 1.34–1.30 (m, 1H), 1.26 (d, *J* = 7.0 Hz, 6H), 1.22 (s, 3H), 0.97 (s, 3H), 0.95 (s, 3H).

^{13}C NMR (126 MHz, $CDCl_3$): δ = 150.1, 146.2, 132.8, 129.0, 123.3, 122.6, 50.6, 41.9, 39.0, 38.1, 34.2, 33.6, 33.5, 30.2, 25.0, 24.3, 21.8, 19.5, 19.2.

HRMS *m/z* (GC-EI): calculated for $C_{20}H_{30}^+$ ($[M]^+$): 270.23420; found: 270.23411.

Chiral HPLC (150 mm, OJ-3R, 4.6 mm; MeCN/H_2O = 70:30; Flow rate = 1 mL/min; λ = 220 nm): t_{R1} = 8.7 min (**9c**), t_{R2} = 9.0 min (ent-**9c**).

$[\alpha]_D^{25}$ = +31.8 (*c* = 0.85, $CHCl_3$).

(4a*S*,10a*S*)-6-(tert-butyl)-1,1,4a-trimethyl-1,2,3,4,4a,9,10,10a-octahydrophenanthrene 9d

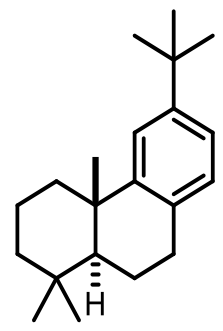

The second cyclization was performed according to **Method A**, 76% yield;

R_F (pentane) = 0.7.

e.r.: 91:9.

¹H NMR (501 MHz, $CDCl_3$): δ = 7.34 (d, *J* = 2.1 Hz, 1H), 7.14 (dd, *J* = 7.9, 2.1 Hz, 1H), 7.00 (d, *J* = 7.9 Hz, 1H), 2.95–2.80 (m, 2H), 2.36–2.33 (m, 1H), 1.90–1.86 (m, 1H), 1.80–1.68 (m, 2H), 1.65–1.61 (m, 1H), 1.52–1.47 (m, 1H), 1.46–1.40 (m, 1H), 1.38–1.35 (m, 1H), 1.31 (s, 9H), 1.26–1.23 (m, 1H), 1.22 (s, 3H), 0.96 (s, 3H), 0.95 (s, 3H).
¹³C NMR (126 MHz, $CDCl_3$): δ = 149.7, 148.4, 132.4, 128.7, 122.4, 121.3, 50.6, 41.9, 39.0, 38.2, 34.7, 33.6, 33.5, 31.7, 30.1, 25.1, 21.8, 19.5, 19.2.
HRMS *m*/*z* (GC-EI): calculated for $C_{21}H_{32}^+$ ($[M]^+$): 284.24985; found: 284.24977.
Chiral HPLC (150 mm, OJ-3R, 4.6 mm; MeCN/H_2O = 70:30; Flow rate = 0.5 mL/min; λ = 220 nm): t_{R1} = 41.7 min (ent-**9d**), t_{R2} = 42.9 min (**9d**).
$[\alpha]_D^{25}$ = +46.3 (*c* = 0.85, $CHCl_3$).

(4a*S*,10a*S*)-6-methoxy-1,1,4a-trimethyl-1,2,3,4,4a,9,10,10a-octahydrophenanthrene 9e

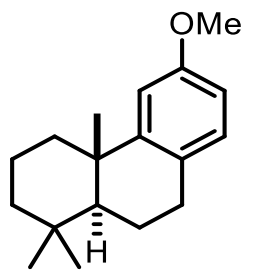

The second cyclization was performed according to **Method A**, 82% yield;
***R**$_F$* (pentane/Et_2O 10:1) = 0.7.
e.r.: 82:18.
¹H NMR (501 MHz, $CDCl_3$): δ = 6.97 (d, *J* = 8.4 Hz, 1H), 6.81 (d, *J* = 2.8 Hz, 1H), 6.67 (dd, *J* = 8.4, 2.8 Hz, 1H), 3.78 (s, 3H), 2.91–2.75 (m, 2H), 2.27–2.22 (m, 1H), 1.89–1.85 (m, 1H), 1.76–1.66 (m, 3H), 1.49–1.46 (m, 1H), 1.41–1.31 (m, 2H), 1.26–1.25 (m, 1H), 1.19 (s, 3H), 0.95 (s, 3H), 0.93 (s, 3H).
¹³C NMR (126 MHz, $CDCl_3$): δ = 157.8, 151.6, 129.9, 127.6, 110.8, 110.3, 55.4, 50.5, 41.8, 39.0, 38.2, 33.6, 33.5, 29.7, 24.9, 21.8, 19.5, 19.3.
HRMS *m*/*z* (GC-EI): calculated for $C_{18}H_{26}O^+$ ($[M]^+$): 258.19782; found: 258.19781.
Chiral GC (Ivadex-1 0.25/0.25df, 25.0 m, temperature: 220/150 iso 20 min, 0.2/min 160, 20/min, 220 iso 5 min, 0.60 bar H_2, sample size: 0.2 µL): t_{R1} = 100.5 min (ent-**9e**), t_{R2} = 103.6 min (**9e**).
$[\alpha]_D^{25}$ = +32.3 (*c* = 0.70, $CHCl_3$).

(4a*S*,10a*S*)-6-fluoro-1,1,4a-trimethyl-1,2,3,4,4a,9,10,10a-octahydrophenanthrene 9f

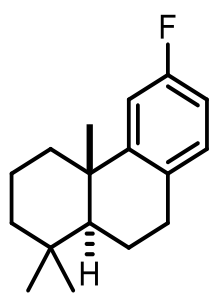

The second cyclization was performed according to **Method B**, 54% yield;
***R**$_F$* (pentane) = 0.7.
e.r.: 82:18.
¹H NMR (501 MHz, $CDCl_3$): δ = 6.99–6.92 (m, 2H), 6.78–6.74 (m, 1H), 2.93–2.76 (m, 2H), 2.21–2.18 (m, 1H), 1.90–1.86 (m, 1H), 1.76–1.60 (m, 3H), 1.50–1.44 (m, 1H), 1.42–1.29 (m, 2H), 1.22–1.19 (m, 1H), 1.18 (s, 3H), 0.96 (s, 3H), 0.93 (s, 3H).

^{13}**C NMR** (126 MHz, $CDCl_3$): δ = 162.4 (d, *J* = 214.7 Hz), 152.4 (d, *J* = 5.7 Hz), 130.8, (d, *J* = 2.9 Hz), 130.3 (d, *J* = 7.7 Hz), 112.4 (d, *J* = 21.2 Hz), 111.1 (d, *J* = 22.2 Hz), 50.2, 41.7, 38.9, 33.6, 33.4, 29.8, 24.8, 21.8, 19.4, 19.1.

^{19}**F NMR** (471 MHz, $CDCl_3$) δ = −117.9.

HRMS *m/z* (GC-EI): calculated for $C_{17}H_{23}F^+$ ($[M]^+$): 246.17783; found: 246.17776.

Chiral GC (Ivadex-1 0.25/0.25df, 25.0 m, temperature: 220/ 150 iso 20 min, 0.2/min 160, 20/min, 220 iso 5 min, 0.60 bar H_2, sample size: 0.2 µL): t_{R1} = 29.9 min (ent-**9f**), t_{R2} = 30.5 min (**9f**).

$[\alpha]_D^{25}$ = +28.6 (*c* = 0.68, $CHCl_3$).

(4a*S*,10a*S*)-6-chloro-1,1,4a-trimethyl-1,2,3,4,4a,9,10,10a-octahydrophenanthrene 9g

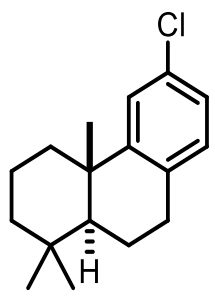

The second cyclization was performed according to **Method B**, 63% yield;

$\boldsymbol{R_F}$ (pentane) = 0.7.

e.r.: 87:13.

^{1}**H NMR** (501 MHz, $CDCl_3$): δ = 7.22 (d, *J* = 2.2 Hz, 1H), 7.04 (dd, *J* = 8.2, 2.2 Hz, 1H), 6.97 (d, *J* = 8.2 Hz, 1H), 2.93–2.88 (m, 1H), 2.84–2.76 (m, 1H), 2.25–2.21 (m, 1H), 1.91–1.86 (m, 1H), 1.76–1.61 (m, 3H), 1.51–1.47 (m, 1H), 1.42–1.38 (m, 1H), 1.31–1.28 (m, 1H), 1.25–1.22 (m, 1H), 1.18 (s, 3H), 0.96 (s, 3H), 0.93 (s, 3H).

^{13}**C NMR** (126 MHz, $CDCl_3$): δ = 152.1, 133.8, 131.3, 130.5, 125.4, 124.7, 50.1, 41.7, 38.9, 38.1, 33.6, 33.4, 29.9, 24.9, 21.8, 19.3, 19.0.

HRMS *m/z* (GC-EI): calculated for $C_{17}H_{23}Cl^+$ ($[M]^+$): 262.14828; found: 262.14823.

Chiral HPLC (150 mm, OJ-3R, 4.6 mm; MeCN/H_2O = 70:30; Flow rate = 1 mL/min; λ = 220 nm): t_{R1} = 9.90 min (**9g**), t_{R2} = 12.9 min (ent-**9g**).

$[\alpha]_D^{25}$ = +12.7 (*c* = 0.85, $CHCl_3$).

(4a*S*,10a*S*)-6-bromo-1,1,4a-trimethyl-1,2,3,4,4a,9,10,10a-octahydrophenanthrene 9h

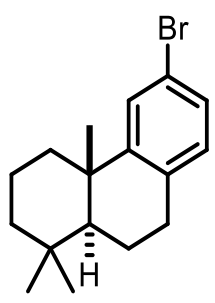

The second cyclization was performed according to **Method B**, 69% yield;

$\boldsymbol{R_F}$ (pentane) = 0.7.

e.r.: 86:14.

^{1}**H NMR** (501 MHz, $CDCl_3$): δ = 7.37 (d, *J* = 2.2 Hz, 1H), 7.18 (dd, *J* = 8.5, 2.2 Hz, 1H), 6.91 (d, *J* = 8.2 Hz, 1H), 2.91–2.86 (m, 1H), 2.81–2.74 (m, 1H), 2.24–2.21 (m, 1H), 1.91–1.86 (m, 1H), 1.76–1.60 (m, 3H), 1.51–1.47 (m, 1H), 1.42–1.38 (m, 1H), 1.31–1.28 (m, 1H), 1.25–1.21 (m, 1H), 1.18 (s, 3H), 0.95 (s, 3H), 0.93 (s, 3H).

^{13}**C NMR** (126 MHz, $CDCl_3$): δ = 152.6, 134.3, 130.9, 128.3, 127.7, 119.5, 50.1, 41.7, 38.9, 38.2, 33.6, 33.4, 29.9, 24.9, 21.7, 19.3, 18.9.

HRMS *m*/*z* (GC-EI): calculated for $C_{17}H_{23}Br^{+}$ ($[M]^{+}$): 306.09777; found: 306.09772.

Chiral HPLC (150 mm, OJ-3R, 4.6 mm; MeCN/H_2O = 70:30; Flow rate = 0.5 mL/min; λ = 220 nm): t_{R1} = 21.6 min (**9h**), t_{R2} = 27.3 min (ent-**9h**).

$[\alpha]_D^{25}$ = +19.6 (*c* = 0.85, $CHCl_3$).

6. Cyclization of polyenes with multiple olefinic sites

General Procedure: A 5 ml vial was charged with IDPi **5d** (2.7 μmol, 2 mol%) and a magnetic stir bar under an atmosphere of argon. Dry toluene (0.107 mL) and PFTB (0.275 mmol, 2 eq.) added. The vial was cooled to −50 ºC. The substrate **10** (0.137 mmol, 1.0 eq.) was added dropwise and the reaction was stirred at desired temperature. Afterwards, the reaction mixture was treated with triethylamine (0.257 mmol, 3.8 μL, 0.2 eq.). After the mixture was stirred for 10 min, the solvent of the reaction mixture was directly removed under reduced pressure. The product was purified by column chromatography on silica gel (pentane), then analyzed by ^{1}H NMR and chiral GC or chiral HPLC to determine regioisomeric ratio (r.r.) and the enantiomeric ratio (e.r.).

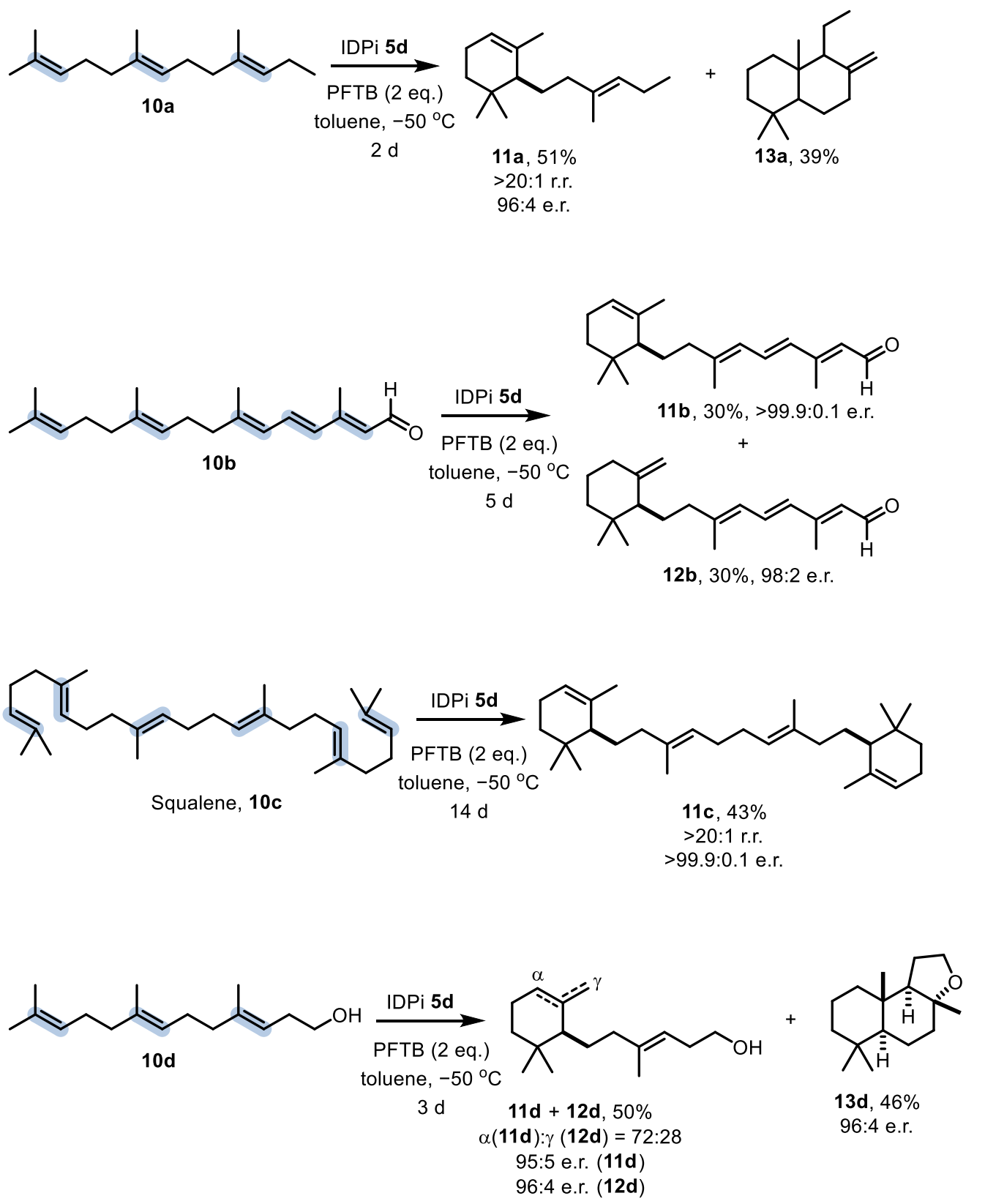

(*S*,*E*)-1,5,5-trimethyl-6-(3-methylhex-3-en-1-yl)cyclohex-1-ene 11a

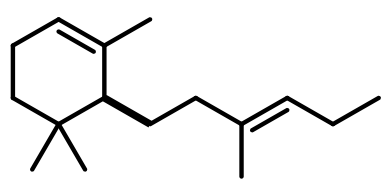

11a

At −50 °C for 2 d, 51% yield;

RF (pentane) = 0.7.

r.r. >20:1;

e.r. of **11a**: 96:4.

^{1}H NMR (501 MHz, $CDCl_3$): δ = 5.28–5.27 (m, 1H), 5.14–5.11, (m, 1H), 2.01–1.94 (m, 6H), 1.68–1.67 (m, 3H), 1.60 (s, 3H), 1.49–1.35 (m, 4H), 1.14–1.09 (m, 1H), 0.95–0.92 (m, 6H), 0.86 (s, 3H).

^{13}C NMR (126 MHz, $CDCl_3$): δ = 137.1, 135.2, 126.4, 119.9, 49.1, 40.7, 32.7, 31.8, 30.0, 27.7, 27.6, 23.6, 23.2, 21.3, 16.1, 14.6.

HRMS *m*/*z* (GC-EI): calculated for $C_{16}H_{28}^+$ ($[M]^+$) 220.21855; found: 220.21841.

Chiral GC (BGB 176 0.25/0.25df, 30.0 m, temperature: 220/120 iso 60 min, 5/min, 220 iso 5 min, 0.60 bar H_2, sample size: 0.2 µL): t_{R1} = 27.6 min (**11a**), t_{R2} = 28.6 min (ent-**11a**).

$[\alpha]_D^{25}$ = −25.0 (*c* = 0.87, $CHCl_3$).

5-ethyl-1,1,4a-trimethyl-6-methylenedecahydronaphthalene 13a

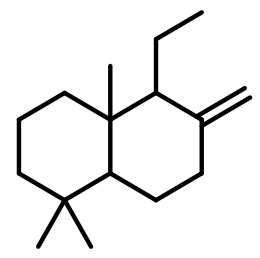

At −50 °C for 2

d, 39% yield;

R_F (pentane) = 0.8.

^{1}H NMR (501 MHz, $CDCl_3$): δ = 4.82 (d, *J* = 1.7 Hz, 1H), 4.50 (d, *J* = 1.7 Hz, 1H), 2.40–2.36 (m, 1H), 2.01–1.95 (m, 1H), 1.76–1.69 (m, 2H), 1.55 (s, 3H), 1.52–1.45 (m, 3H), 1.37–1.28 (m, 3H), 1.21–1.14 (m, 1H), 1.10–1.02 (m, 3H), 0.87 (s, 6H), 0.80 (s, 3H), 0.66 (s, 3H).

^{13}C NMR (126 MHz, $CDCl_3$): δ = 148.7, 106.2, 59.3, 55.7, 42.4, 39.2, 38.6, 33.8, 24.7, 21.9, 19.6, 16.4, 14.6, 13.7.

HRMS *m*/*z* (GC-CI): calculated for $C_{16}H_{32}N^+$ ($[M+NH_4]^+$) 238.252924; found: 238.25309.

(*S*,2*E*,4*E*,6*E*)-cyclodidehydrogeranylgeranial 11b

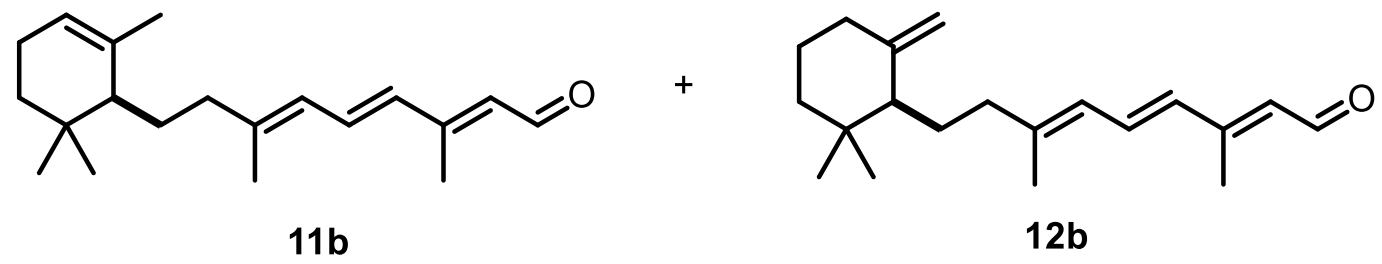

Condition 1: at −50 °C for 5 d. 60% yield; a:γ = 50:50; e.r. of a-product **11b**: >99.0:1.0, e.r. of γ-product **12b**: 97.5:2.5.

Condition 2: at −50 °C for 5 d, then 40 °C

for 2d. 35% yield; >20:1 r.r.; e.r. of a-product: 95:5.

(*S*,2*E*,4*E*,6*E*)-a-cyclodidehydrogeranylgeranial 11b

^{1}H NMR (600 MHz, C_6D_6): δ = 10.00 (d, *J* = 8.0 Hz, 1H), 6.72 (dd, *J* = 15.3, 11.0 Hz, 1H), 5.97 (dq, *J* = 7.7, 1.1 Hz, 1H), 5.96 (d, *J* = 15.3 Hz, 1H), 5.88 (dm, *J* = 11.2 Hz, 1H), 5.38–5.33 (m, 1H), 2.10-2.05 (m, 2H), 1.99–1.94 (m, 2H), 1.74 (d, *J* = 1.1 Hz, 3H), 1.73–1.67 (m, 3H), 1.60 (d, *J* = 1.3 Hz, 3H), 1.54 (ddd, *J* = 10.0, 5.0, 1.5 Hz, 1H), 1.47–1.44 (m, 1H), 1.44–1.42 (m, 1H), 1.43–1.40 (m, 1H), 1.10–1.08 (m, 1H), 0.93 (s, 3H), 0.92 (s, 3H).

^{13}C NMR (151 MHz, C_6D_6): δ = 190.0, 153.6, 145.4, 136.3, 133.8, 131.9, 129.4, 125.5, 120.9, 49.3, 41.2, 32.8, 31.9, 29.9, 27.73, 27.69, 23.7, 23.4, 17.1, 12.5.

2D-HPLC: 1D (1st dimension, achiral stationary phase): 50 mm Nucleodur Gravity SB 1.8 µm, 4.6 mm i.D., MeOH/H_2O Gradient: 70% to 95% MeOH, 1.0 mL/min, 37.5 MPa, 308 K, UV: λ = 220 nm): t_R = 9.63-9.67 min (sampling range). 2D (2 nd dimension, chiral stationary phase): (150 mm Chiralpak IG-3, 4.6 mm i.D., MeCN/ H_2O Gradient: 70% to 95% MeCN, 1.0 mL/min, 14.3 MPa, 298 K, UV: λ = 220 nm): t_{R1} = 8.6 min (>99.0%), t_{R2} = 9.5 min (<1%).

HRMS m/z (ESI^+): calculated for $C_{20}H_{30}O^+$ ($[M]^+$): 286.22912, found: 286.22927;

(*S*,2*E*,4*E*,6*E*)-γ-cyclodidehydrogeranylgeranial 12b

^{1}H NMR (600 MHz, C_6D_6): characteristic spectroscopic signals: δ = 4.83 (app q, *J* = 1.9 Hz, 1H), 4.49 (app q, *J* = 1.9 Hz, 1H).

2D-HPLC: 1D (1st dimension, achiral stationary phase): 50 mm Nucleodur Gravity SB 1.8 µm, 4.6 mm i.D., MeOH/H_2O Gradient: 70% to 95% MeOH, 1.0 mL/min, 37.5 MPa, 308 K, UV: λ = 220 nm): t_R = 9.74–9.78 min (sampling range). 2D (2 nd dimension, chiral stationary phase): (150 mm Chiralpak IG-3, 4.6 mm i.D., MeCN/H_2O Gradient: 70% to 95% MeCN, 1.0 mL/min, 14.3 MPa, 298 K, UV: λ = 220 nm): t_{R1} = 7.6 min (2.5%), t_{R2} = 9.4 min (97.5%).

(*S*)-monocyclized squalene 11c

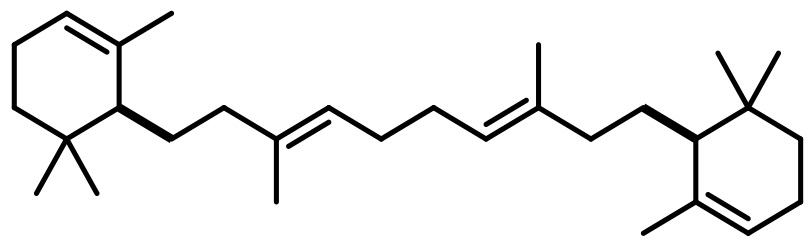

11c

At −50 °C for 14 d, 43% yield;

***R**$_F$* (pentane) = 0.7.

r.r. >20:1;

e.r. of **11c**: >99.9:0.1. (The reaction was conducted in separate experiments using (*S*,*S*)- and (*R*,*R*)-IDPi catalysts, and the enantiomeric composition of the products was analyzed by 2D HPLC)

^{1}H NMR (501 MHz, $CDCl_3$): δ = 5.28 (s, 2H), 5.14 (s, 2H), 2.02–1.96 (m, 12H), 1.67 (s, 6H), 1.60 (s, 6H), 1.43–1.41 (m, 4H), 1.13–1.10 (m, 2H), 0.92 (s, 6H), 0.86 (s, 6H).

^{13}C NMR (151 MHz, $CDCl_3$): δ = 137.1, 136.1, 124.3, 119.9, 49.1, 40.8, 32.7, 31.8, 30.0, 28.4, 27.7, 27.6, 23.6, 23.2, 16.3.

HRMS *m*/*z* (GC-EI): calculated for $C_{30}H_{50}^+$ ($[M]^+$) 410.39070; found: 410.39083.

2D-HPLC: 1D (1st dimension, achiral stationary phase): 50 mm Nucleodur C18 Gravity SB, 4.6 mm i.D., MeOH/H_2O 95:5 *v*/*v*, 1.0 mL/min, 22.3 MPa, 308 K, UV: λ = 220 nm): t_R = 18.85–18.89 min (sampling range). 2D (2 nd dimension, chiral stationary phase): (150 mm Chiralpak IG-3, 4.6 mm i.D., MeCN/ H_2O 80:20 *v*/*v*, 1.0 mL/min, 13.5 MPa, 298 K, UV: λ = 220 nm): t_{R1} = 11.7 min (*R*-product), t_{R2} = 14.1 min (*S*-product).

$[\alpha]_D^{25}$ = −46.2 (*c* = 0.55, $CHCl_3$).

(*S*,*E*)-4-methyl-6-(2,6,6-trimethylcyclohex-2-en-1-yl)hex-3-en-1-ol 11d[8]

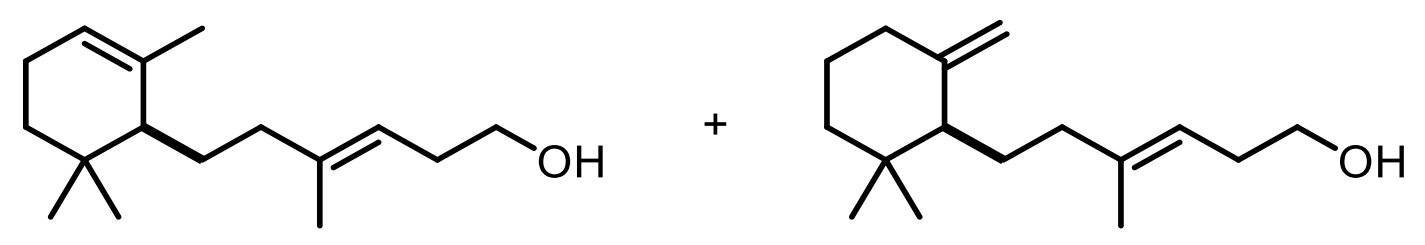

11d 72：28 **12d**

At −50 °C for 3 d, 50% yield;

R_F (pentane/EtOAc 5:1) = 0.6.

α:γ = 72:28

e.r. of **11d**: 95:5 e.r..

e.r. of **12d**: 96:4 e.r..

^{1}H NMR of **11d** (500 MHz, $CDCl_3$): δ = 5.31–5.25 (m, 1H), 5.13 (tq, *J* = 7.4, 1.4 Hz, 1H), 3.62 (t, *J* = 6.5 Hz, 2H), 2.32–2.25 (m, 2H), 2.08–2.02 (m, 2H), 1.97–1.93 (m, 2H), 1.67 (q, *J* = 1.7 Hz, 3H), 1.66–1.64 (m, 3H), 1.55–1.49 (m, 1H), 1.46–1.34 (m, 3H), 1.12 (ddd, *J* = 13.3, 6.0, 3.4 Hz, 1H), 0.92 (s, 3H), 0.86 (s, 3H).

^{13}C NMR of **11d** (151 MHz, $CDCl_3$): δ = 139.8, 136.9, 120.1, 119.7, 62.7, 49.2, 40.8, 32.7, 31.7, 31.7, 30.0, 27.7, 27.6, 23.6, 23.2, 16.5.

Analytical 2D-HPLC method for **11d**: 1D (1st dimension, achiral stationary phase, 100 mm VDSpher PUR 100 SIL, 4.6 mm i.D., *n*-heptane/propan-2-ol 95.5:0.5 v/v, 1.0 mL/min, 20.8 MPa, 288 K, UV: λ = 205 nm): t_R = 11.91–11.95 (sampling range). 2D (2nd dimension, chiral stationary phase, 150 mm Chiralpak IG-3, 4.6 mm i.D., n-heptane/propan-2-ol = 99.5:0.5 v/v, 1.0 mL/min, 9.7 MPa, 288 K, UV: λ = 220 nm): (*R*)-(*E*)-α-cyclohomofarnesol t_{R1} = 9.77 min, (*S*)-(*E*)-α-cyclohomofarnesol: t_{R2} = 11.62 min.

method for **12d**: (1st dimension, achiral stationary phase, 100 mm VDSpher PUR 100 SIL, 4.6 mm i.D., *n*-heptane/propan-2-ol 95.5:0.5 v/v, 1.0 mL/min, 20.8 MPa, 288 K, UV: λ = 205 nm): t_R = 11.73–11.77 (sampling range). 2D (2nd dimension, chiral stationary phase, 150 mm Chiralpak OD-3, 4.6 mm i.D., *n*-heptane/propan-2-ol = 99.5:0.5 v/v, 1.0 mL/min, 9.7 MPa, 288 K, UV: λ = 220 nm): t_{R1} = 24.78 min, t_{R2} = 26.34 min.

7. Synthesis of Imidodiphosphorimidate IDPi catalysts

The chiral imidodiphosphorimidate acids IDPis **5a-5d** were synthesized according to literature procedures.[8] IDPi **5d** was synthesized as follows:

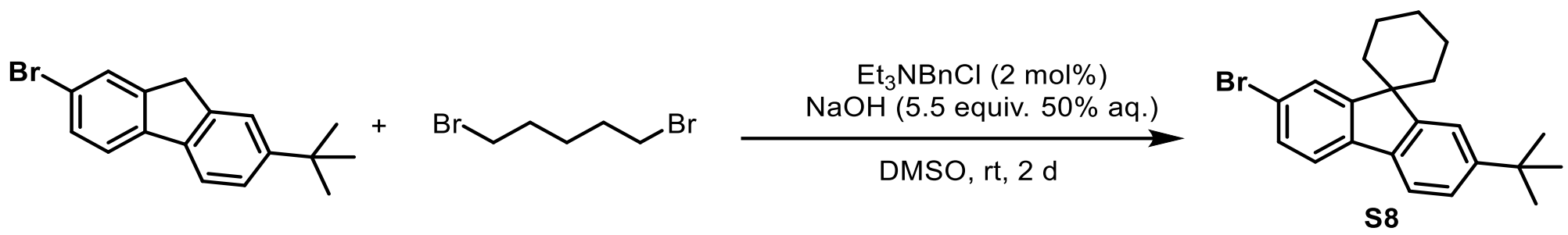

2'-bromo-7'-(*tert*-butyl)spiro[cyclohexane-1,9'-fluorene] S8: NaOH (50% aq., 110 mmol, 5.5 eq.) was added dropwise to the mixture of 2-bromo-7-*tert*-butylfluorene (20.0 mmol, 1.0 eq.) and benzyl-triethylammonium chloride (0.4 mmol, 2 mol%) in DMSO (20 mL) under argon at rt. After the subsequent dropwise addition of 1,5-dibromopentane (20.0 mmol, 1.0 eq.), the reaction was stirred for 2 d at rt. Then, water (100 mL) and toluene (100 mL) were added and the organic layer was separated, dried over $MgSO_4$ and concentrated under reduced pressure. The crude product was purified by column chromatography (hexane) to yield **S8** as a white solid (5.52 g, 75%). **^{1}H**

NMR (501 MHz, $CDCl_3$): δ = 7.78 (d, J = 1.9 Hz, 1H), 7.65 (d, J = 1.8 Hz, 1H), 7.62 (d, J = 8.0 Hz, 1H), 7.56 (d, J = 8.0 Hz, 1H), 7.46 (dd, J = 8.0, 1.8 Hz, 1H), 7.41 (dd, J = 8.0, 1.8 Hz, 1H), 1.94–1.89 (m, 4H), 1.83–1.69 (m, 6H), 1.39 (s, 9H).). **^{13}C NMR** (151 MHz, $CDCl_3$): δ = 155.4, 153.0, 150.7, 138.8, 136.1, 130.0, 127.9, 124.4, 121.4, 121.0, 120.2, 119.4, 50.6, 35.7, 35.2, 31.8, 25.6, 22.8. **HRMS** *m/z* (GC-EI): calculated for $C_{22}H_{25}Br^+$ ($[M]^+$) 368.11342; found: 368.11367.

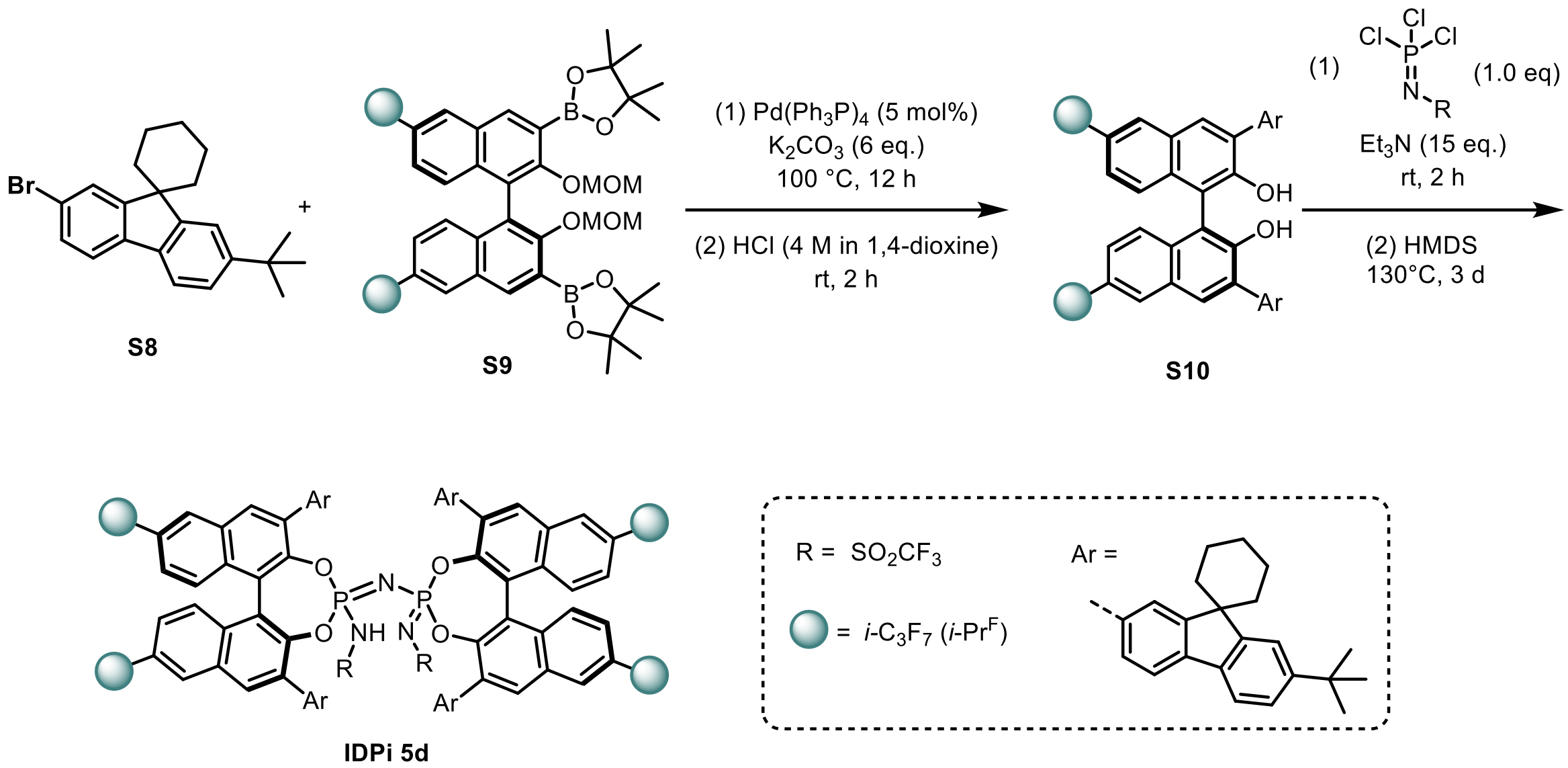

Diol S10 synthesis: To a two-neck round-bottom flask with a condenser was added **S9** (0.50 mmol, 1.0 eq.), the respective aryl bromide **S8** (Ar–Br, 1.00 mmol, 2.0 eq.), tetrakis(triphenylphosphine)palladium (25.0 µmol, 0.05 eq.) and K_2CO_3 (3.00 mmol, 6.0 eq.) under an atmosphere of argon. Degassed toluene (3 mL), ethanol (2 mL) and water (1 mL) were sequentially added. The mixture was then heated to 110 °C and stirred overnight. After cooling to room temperature, the organic layer was separated and the aqueous phase was extracted with ethyl acetate (3 × 10 mL). The organic phase was combined, filtered through a thin layer of silica gel using a Büchner funnel, and the silica gel layer was eluted with additional ethyl acetate. The solvent was removed under reduced pressure and the crude MOM-protected diol was obtained. Subsequently, the crude product was dissolved in a small amount of DCM (1 mL). A solution of HCl (4 M in 1,4-dioxane, 3 mL) was added at room temperature and the mixture was stirred for 3 h. The solvent was evaporated under reduced pressure and the crude product was purified by column chromatography to afford the corresponding diol **S9** in 67% yield as white solid. **^{1}H NMR** (501 MHz, $CDCl_3$): δ = 8.25 (s, 2H), 8.19 (s, 2H), 7.98 (s, 2H), 7.84 (d, J = 7.9 Hz, 2H), 7.77–7.67 (m, 6H), 7.52 (d, J = 9.1 Hz, 2H), 7.44–7.39 (m, 4H), 5.67 (s, 2H), 1.96–1.94 (m, 8H), 1.83–1.81 (m, 12H), 1.41 (s, 18H). **^{13}C NMR** (151 MHz, $CDCl_3$): δ = 154.2, 153.6, 151.9, 150.8, 140.3, 136.4, 134.2, 133.9, 132.9, 132.1, 128.7, 128.3, 125.8, 125.5, 124.4, 121.5, 120.0, 119.6, 112.9, 50.6, 35.9, 35.2, 25.7, 23.0. **^{19}F NMR (471 MHz, $CDCl_3$)**: δ = −75.33–−75.42 (m, 12F), −181.81 (hept, J = 7.3 Hz, 2F). **HRMS** *m/z* (ESI): calculated for $C_{70}H_{59}F_{14}O_2^-$ ($[M–H]^-$) 1197.4297; found: 1197.4305.

In a Schlenk tube under argon, a suspension of diol **S10** (0.21 mmol, 2.1 eq.) in toluene (0.8 mL) was treated with ((trifluormethyl)sulfonyl)phosphorimidoyl trichloride (0.21 mmol, 2.1 eq.) and triethyl amine (1.60 mmol, 16 eq.). The reaction mixture was stirred for 45 min at room temperature, then 4-dimethylaminopyridine (DMAP, 0.10 mmol, 1.0 eq.) and neat 1,1,1,3,3,3-hexamethyldisilazane (HMDS, 0.10 mmol, 1.0 eq.) were added. The reaction mixture was stirred for additional 15 min at room temperature, the Schlenk tube was subsequently sealed and heated to 130 °C for 3 d. After cooling to room temperature, aqueous HCl (10%) was added and the mixture was extracted with DCM.

The combined organic layers were washed with brine, dried over $MgSO_4$, and concentrated under reduced pressure. The crude material was purified by column chromatography on silica gel to afford the desired product as a salt. The corresponding IDPi Brønsted acids were obtained after acidification in DCM with aq. HCl (6 M) and evaporation of the solvent followed by drying under high vacuum as typically off-white solids. Acidification procedure: The salt was dissolved in DCM (5 mL), then aq. HCl (6 m, 8 mL) was added, and the mixture was stirred vigorously for 10 minutes. The organic layer was separated and the aqueous phase was extracted several times with DCM until no product could be detected by TLC in the last DCM extract. The organic layers were combined and the solvent was removed under reduced pressure. The obtained **IDPi 5d** (63% yield) solid was dried under high vacuum for 16 h at room temperature.

(*S*)- imidodiphosphorimidates (5d)

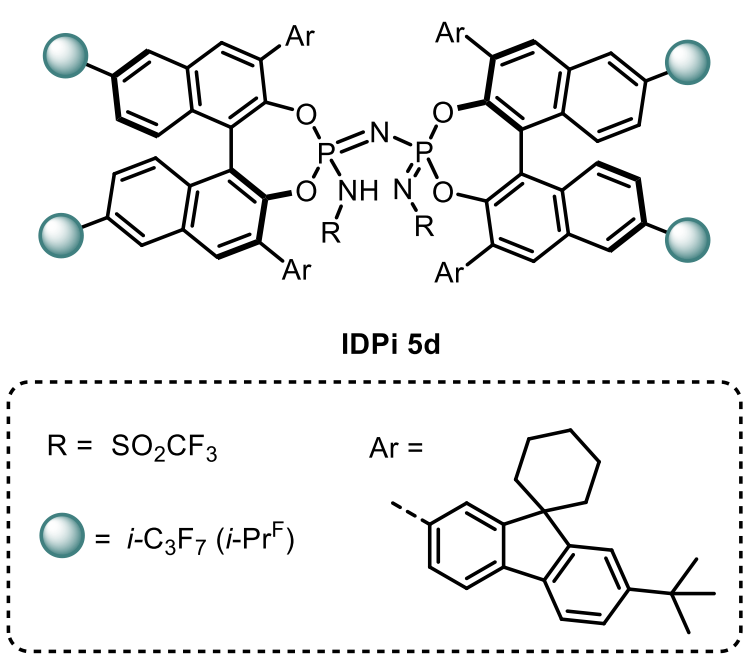

^{1}H NMR (501 MHz, $CDCl_3$): δ = 8.37 (s, 2H), 8.36 (s, 2H), 8.22 (s, 2H), 7.98 (d, *J* = 9.1 Hz, 2H), 7.89 (d, *J* = 9.1 Hz, 2H), 7.78 (s, 2H), 7.74 (s, 2H), 7.63-7.59 (m, 4H), 7.55–7.49 (m, 4H), 7.36 (d, *J* = 7.8 Hz, 2H), 7.29–7.24 (m, 8H), 7.15 (s, 2H), 6.65 (d, *J* = 7.9 Hz, 2H), 6.53 (d, *J* = 7.9 Hz, 2H), 6.10 (d, *J* = 7.9 Hz, 2H), 1.95-1.70 (m, 30H), 1.53–1.43 (m, 10H), 1.34 (s, 18H), 1.33 (s, 18H). **^{13}C NMR (126 MHz, $CDCl_3$)**: δ = 154.2, 154.1, 153.6, 153.2, 150.9, 150.1, 145.8, 144.8, 140.5, 139.8, 136.8, 136.4, 136.0, 133.1, 132.9, 132.4, 132.3, 132.1, 131.9, 131.5, 131.0, 128.8, 128.3, 128.2, 127.7, 125.3, 125.2, 124.5, 124.4, 123.8, 123.6, 123.3, 122.1, 121.6, 121.2, 119.8, 119.0, 118.6, 50.7, 50.5, 26.1, 35.8, 35.1, 35.0, 25.8, 25.3, 23.2, 22.7, 22.6. **^{19}F NMR (471 MHz, $CDCl_3$)**: δ = −75.07–−75.10 (m, 18F), −75.26–−75.30 (m, 6F), −78.19 (s, 6F), −181.91 (hept, *J* = 7.3 Hz, 2F), −182.09 (br, 2F). **^{31}P NMR (203 MHz, $CDCl_3$)**: δ = −16.14. **HRMS** *m*/*z* (ESI): calculated for $C_{142}H_{117}F_{34}N_3O_8P_2S_2^-$ ([M−H]$^-$) 2762.71362; found: 2762.71158.

8. Determination of the absolute configuration of products

The absolute configuration of product **2a** and **2f** were determined by X-ray crystallography after conversion into the corresponding osmate ester and all other compounds were assigned by analogy. Osmate esters were prepared according to a procedure developed by Rychnovsky et al.[9]

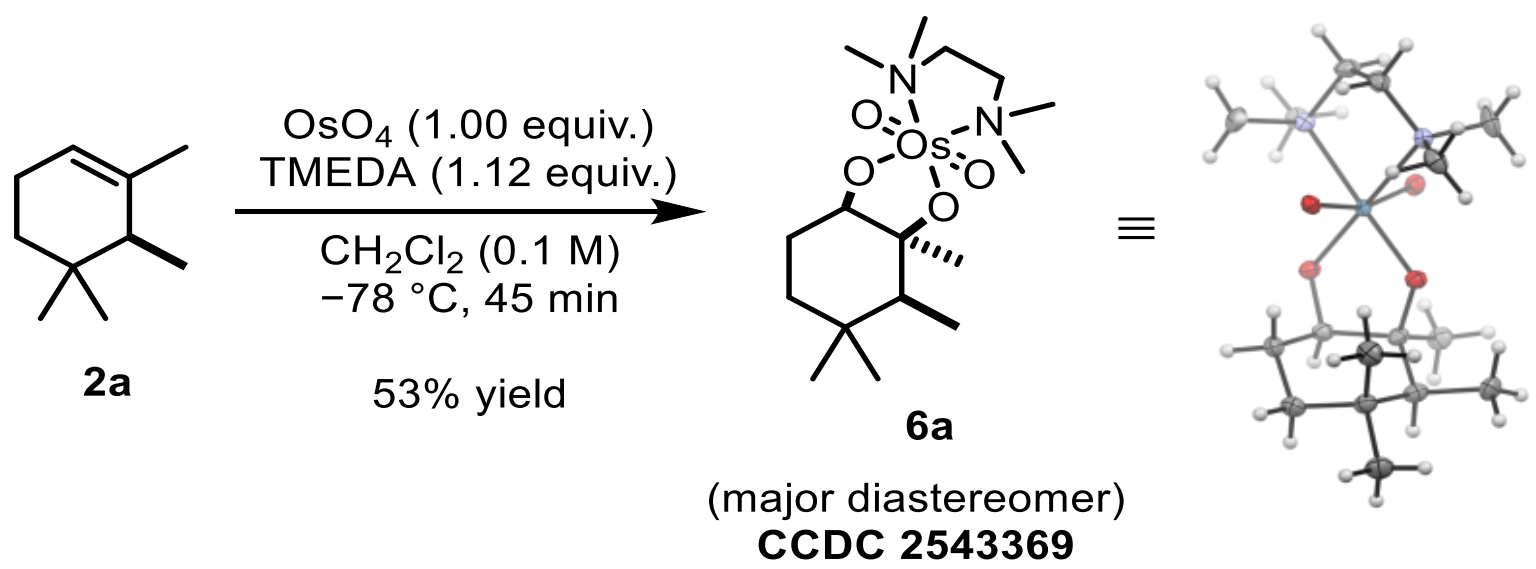

(*S*)-1,5,5,6-tetramethylcyclohex-1-ene osmate ester (6a): *N*,*N*,*N′*,*N′*-Tetramethylethylenediamine (TMEDA) (42.88 µL, 0.28 mmol, 1.1 eq.) was added to a stirred solution of compound **2a** (0.26 mmol, 1.0 eq.) in CH_2Cl_2 (5.0 mL) under argon. The resulting solution was cooled to –78 °C before a solution of osmium tetroxide (0.2 M in CH_2Cl_2,

1.30 mL, 0.26 mmol, 1.0 eq.) was added. The mixture was stirred for 30 min at –78 °C before it was allowed to reach rt. Following removal of the solvent under reduced pressure, a brown solid residue was directly purified by flash column chromatography on silica (eluent: 2–3% MeOH in CH_2Cl_2). Upon evaporation of the solvent under reduced pressure, the corresponding inseparable diastereomeric mixture of osmate ester **6a** and *epi*-**6a** was obtained as a brown solid (53% yield, d.r. > 20:1). Single crystals suitable for X-ray diffraction analysis were obtained by diffusing *n*-pentane into a saturated solution of diastereomeric mixture of osmate ester in THF at rt.

^{1}H NMR (501 MHz, $CDCl_3$): δ = 4.10 (t, *J* = 3.1 Hz, 1H), 3.15–3.04 (m, 2H), 2.98–2.97 (m, 2H), 2.87 (s, 3H), 2.85 (s, 3H), 2.82–2.75 (m, 9H), 2.15 (tt, *J* = 14.1, 3.5 Hz, 1H), 2.05–2.01 (m, 1H), 1.72 (td, *J* = 13.0, 4.0 Hz, 1H), 1.16 (dt, *J* = 12.9, 3.4 Hz, 1H), 0.90 (s, 3H), 0.88 (s, 3H), 0.85 (d, *J* = 7.2 Hz, 3H).

^{13}C NMR (126 MHz, $CDCl_3$): δ = 94.0, 88.0, 64.3, 64.1, 52.4, 51.4, 50.9, 50.5, 41.7, 35.6, 34.0, 32.6, 26.7, 21.0, 20.2, 8.3.

HRMS *m*/*z* (ESI^+): calculated for $C_{16}H_{34}N_2NaO_4Os^+$ $[M+Na]^+$: 533.20256, found:533.20253.

X-Ray: CCDC 2543369.

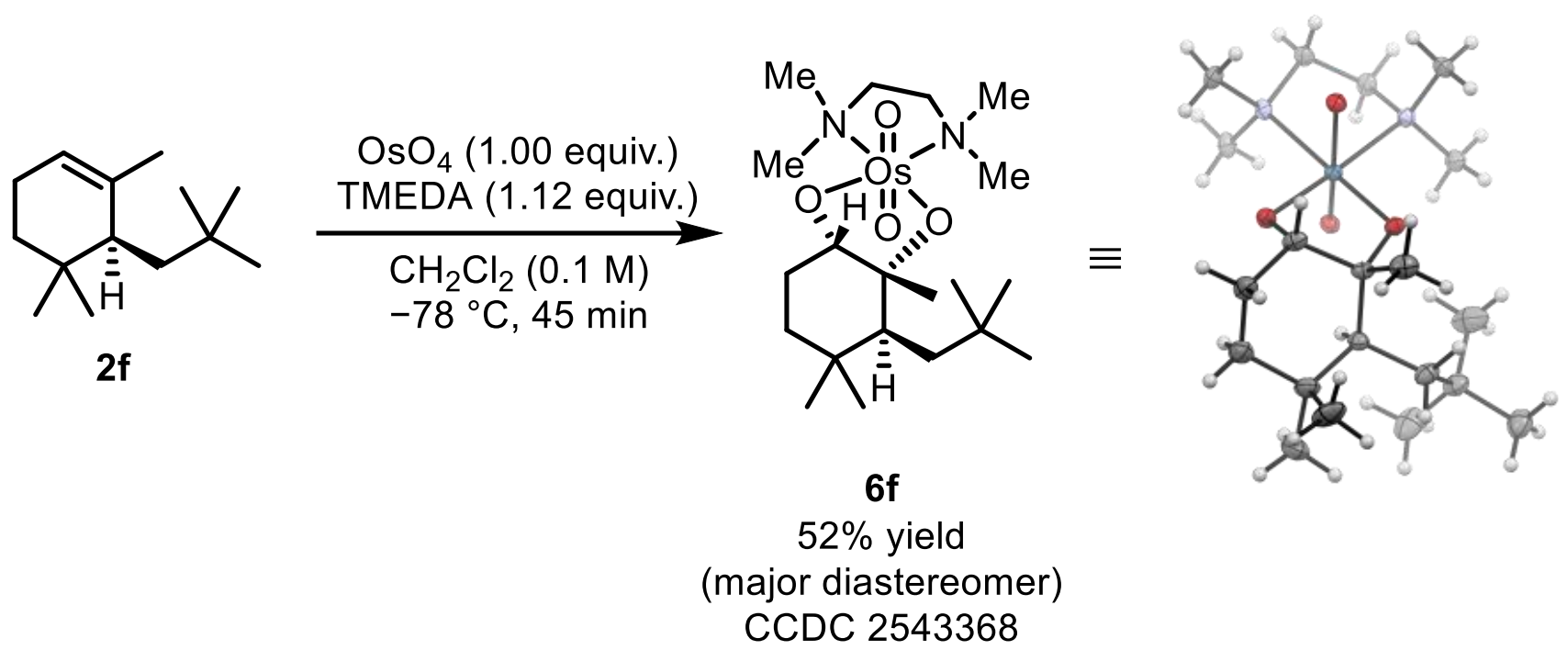

(*S*)-1,5,5-trimethyl-6-neopentylcyclohex-1-ene osmate ester (6f): Alkene **2f** (6.97 mg, 35.9 µmol, 1.00 eq.) was dissolved in dry CH_2Cl_2 (40.0 µL, 0.1 m) and *N*,*N*,*N′*,*N′*-tetramethylethylenediamine (TMEDA, 6.00 µL, 4.65 mg, 40.0 µmol, 1.12 eq.) was added. The resulting colorless solution was cooled to −78 °C and a stock solution of osmium tetroxide in dry CH_2Cl_2 (0.20 m, 0.18 mL, 36.0 µmol, 1.00 eq.) was added dropwise (the colorless solution became dark red and subsequently dark brown/black upon addition of OsO_4). TLC analysis (SiO_2, CH_2Cl_2/MeOH 19:1 *v*/*v*) after 45 min reaction time indicated full consumption of the starting material (R_F = 0.94) and formation of a more polar compound (R_F = 0.31). ^{1}H NMR spectroscopic analysis of the crude reaction mixture indicated the presence of two diastereomers in a ratio of 4:1. The dark brown-red reaction mixture was directly loaded onto a silica gel column equilibrated with CH_2Cl_2. Purification by flash column chromatography on silica gel dichloromethane/methanol (isocratic elution: 19:1 *v*/*v*) as eluent afforded the major diastereomer of title compound **6f** (10.6 mg, 18.8 µmol, 52% yield) as a beige-brown solid/foam. Single crystals suitable for X-ray diffraction were grown by vapor diffusion of *n*-pentane into a solution of osmate ester **6f** in $CDCl_3$/CH_2Cl_2/diethyl ether in a fridge at 4 °C.

Physical state: beige-brown foam.

TLC (SiO_2, CH_2Cl_2/MeOH 19:1 *v*/*v*): $\boldsymbol{R_F}$ = 0.31 (CAM stain).

^{1}H NMR(501 MHz, $CDCl_3$): δ (ppm) = 4.09 (t, *J* = 3.1 Hz, 1H), 3.20–3.08 (m, 2H), 2.96–2.86 (m, 2H), 2.85 (s, 3H), 2.81 (s, 3H), 2.75 (s, 3H), 2.71 (s, 3H), 2.17 (dq, *J* = 14.5, 3.6 Hz, 1H), 2.09 (tt, *J* = 14.3, 3.3 Hz, 1H), 1.82 (td, *J* =

13.3, 3.9 Hz, 1H), 1.77 (dd, J = 7.7, 2.1 Hz, 1H), 1.59 (dd, J = 15.0, 7.8 Hz, 1H), 1.43 (s, 3H), 1.33–1.24 (m, 1H), 1.12 (dt, J = 12.9, 3.4 Hz, 1H), 0.97 (s, 9H), 0.92 (s, 3H), 0.85 (s, 3H).

^{13}C NMR (126 MHz, $CDCl_3$): δ = 96.3, 88.4, 64.5, 63.9, 52.2, 51.8, 51.1, 50.4, 44.4, 40.3, 35.9, 35.6, 33.3, 31.6, 31.1, 26.7, 23.5, 21.3.

HRMS *m*/*z* (ESI$^+$): calculated for $C_{20}H_{43}N_2O_4Os^+$ [M+H]$^+$: 567.28321, found: 567.28290.

X-Ray: CCDC 2543368.

9. Mechanistic studies

9.1 Kinetic profiling

9.1.1 Reaction monitoring

To gain insight into the reaction pathway, the changes in each component were monitored using time-resolved ^{1}H NMR spectroscopy (Figure S1 and Figure S2), and the enantioselectivity of the products was analyzed by GC (Figure S3).

Sample Preparation An oven-dried (80 °C, overnight) 3 mm NMR tube was charged with IDPi **5d** (2 mol%). Dry toluene-d_8 (0.107 mL) and PFTB (0.275 mmol, 2 eq.) were added. The initial mixture was precooled to −78 °C in a dry ice/acetone bath. After addition of the substrate **1a** (0.137 mmol, 1.0 eq.), the NMR tube was quickly turned upside down, vortexed, and transferred to the precooled NMR probe at 223 K (−50 °C). After quick shimming, single-scan ^{1}H and ^{31}P NMR spectra were acquired every 5 min until completion of the reaction.

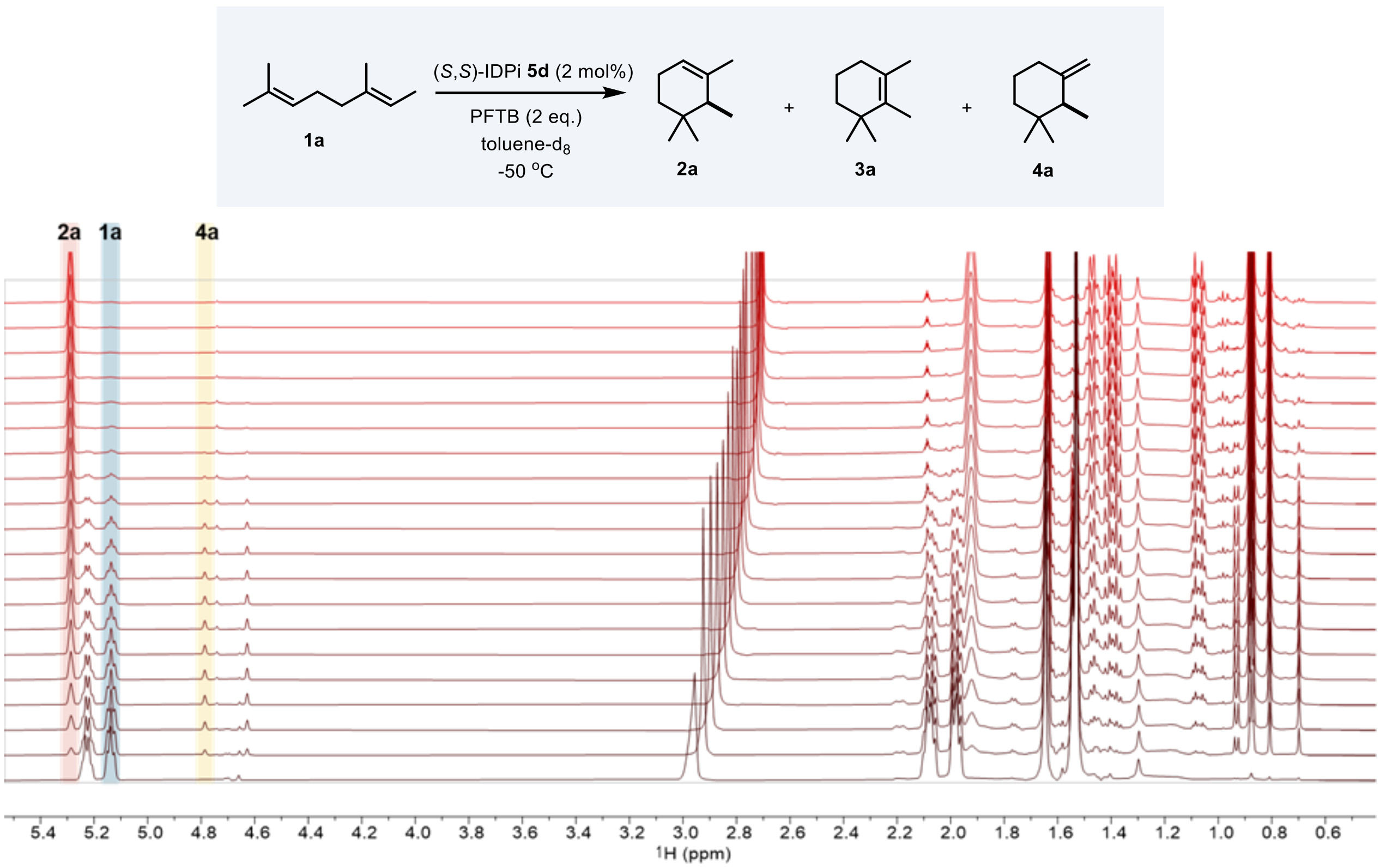

Figure S1. ^{1}H NMR spectra recorded at different time points at 223 K.

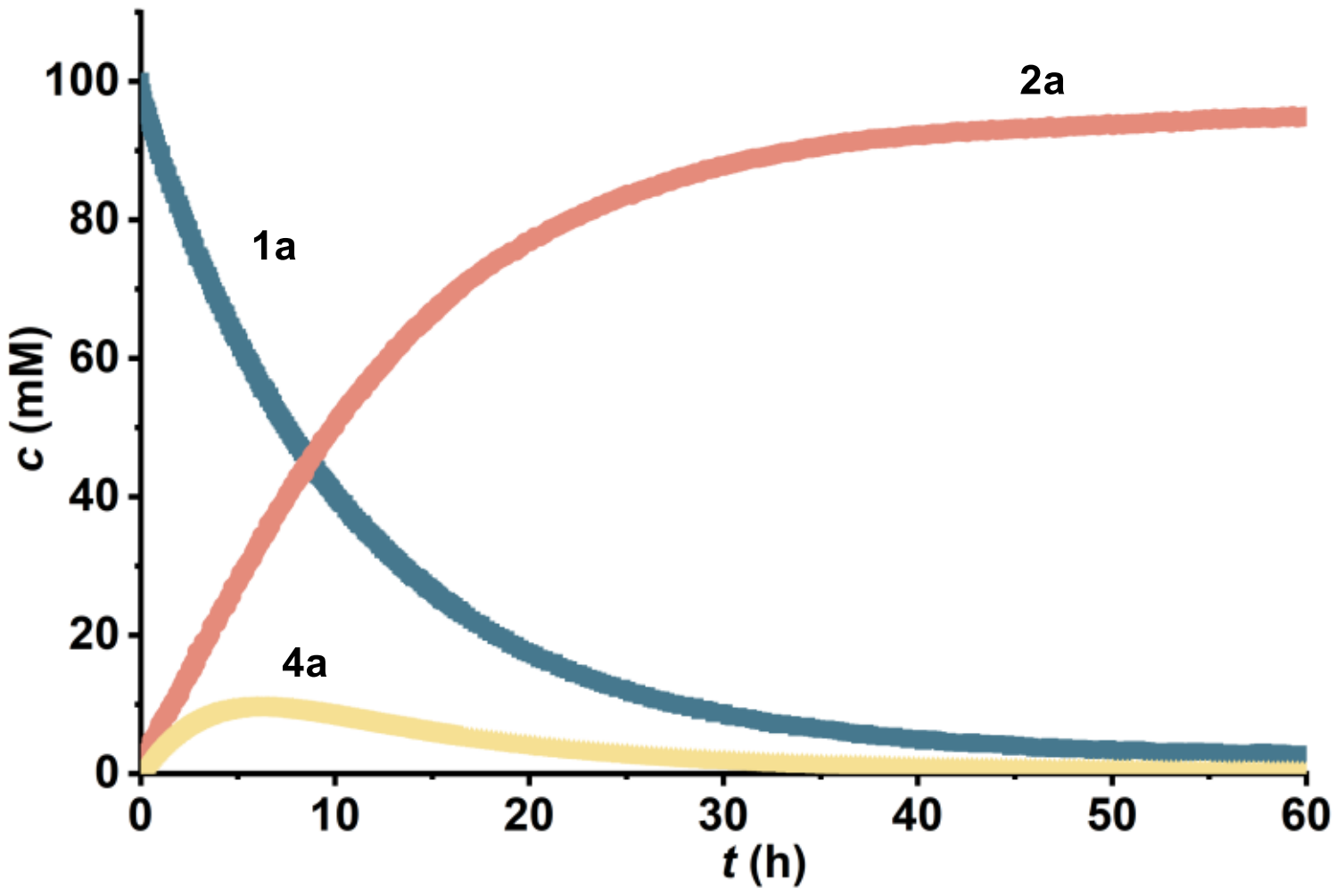

Figure S2. Concentration plots obtained from ^{1}H NMR spectroscopy reaction monitoring.

time	2a (ee)	4a (ee)
1 h	86.2%	92.4%
2 h	91.2%	90.6%
4 h	91.2%	86.3%
6 h	91.2%	82.0%
8 h	91.2%	78.8%
10 h	91.2%	78.0%

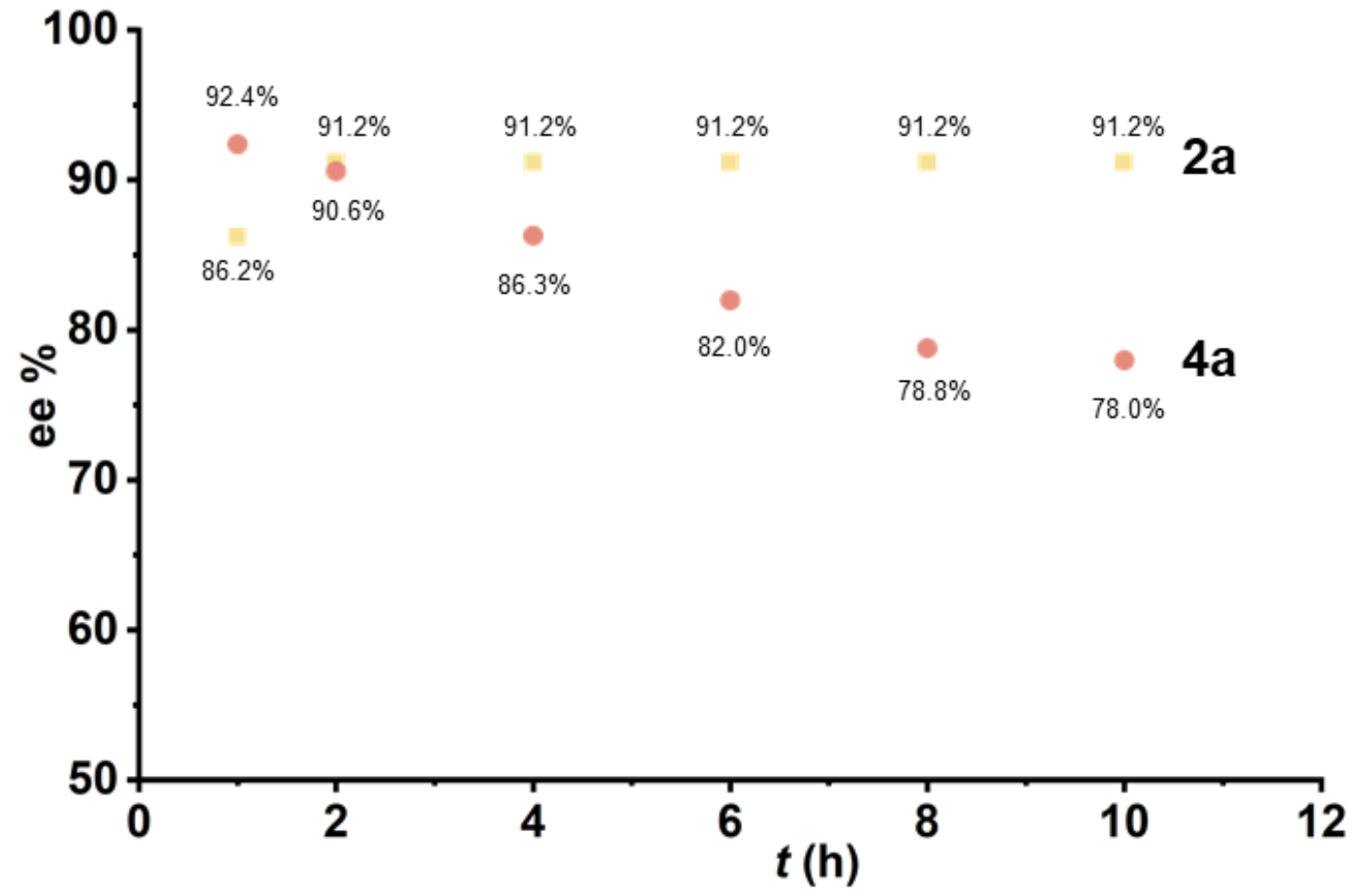

Figure S3. Time-dependent changes in the ee values of the α-product **2a** and γ-product **4a** during the initial reaction stage, monitored by GC.

9.1.2 Control experiments

Synthesis of (±)-γ-isomer 4a

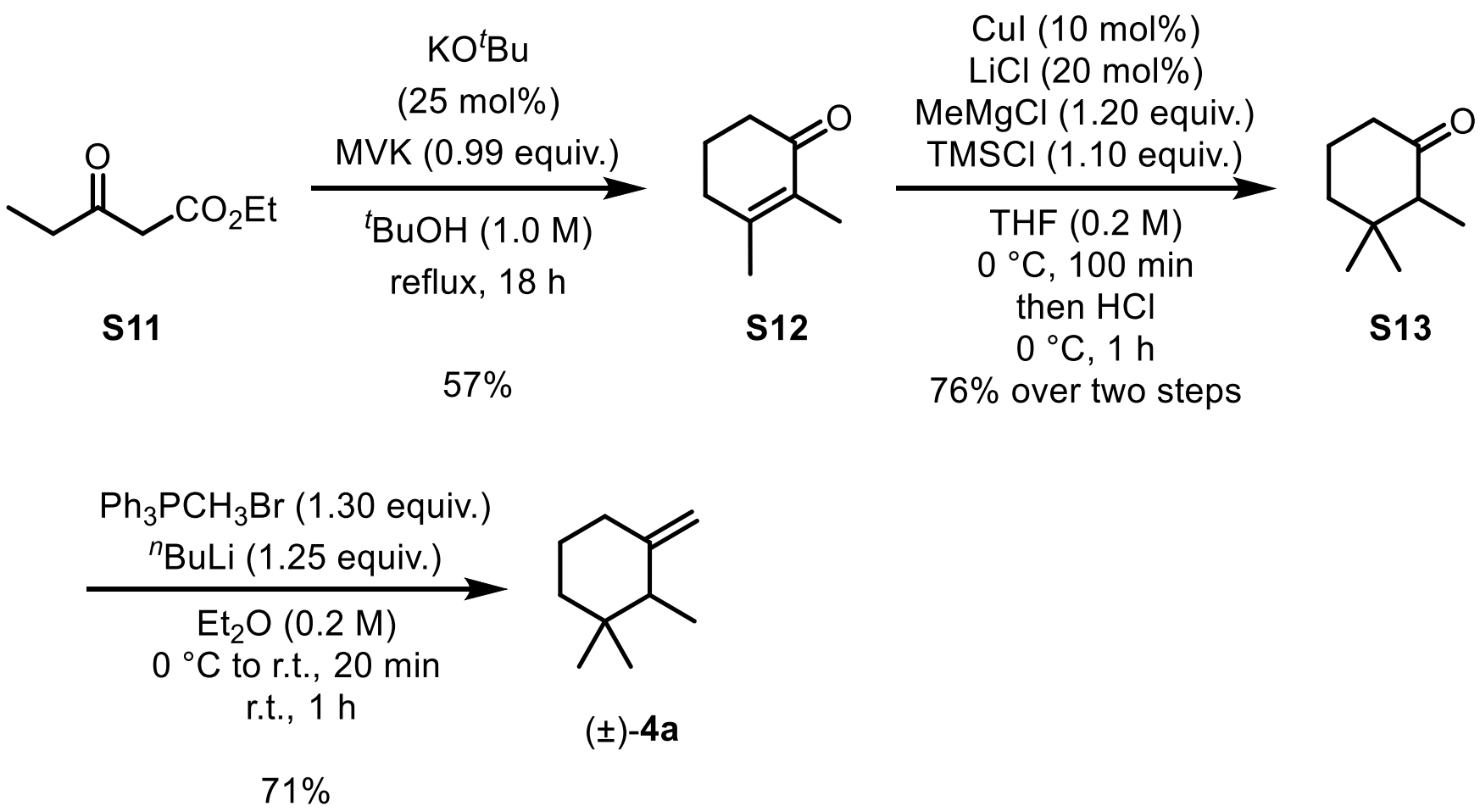

2,3-dimethyl-2-cyclohexen-1-one (S12)

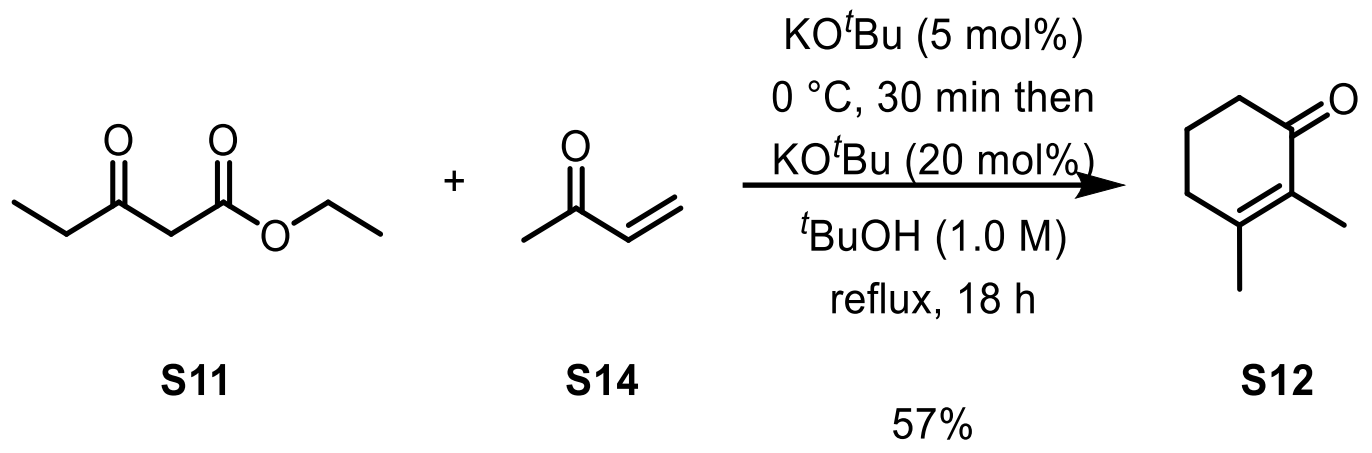

Ketone **S12** was prepared according to a reported literature procedure.[10] A catalytic amount of potassium *tert*-butoxide (236 mg, 2.10 mmol, 0.05 eq., 5 mol%) was added to a vigorously stirred solution of *β*-keto ester **S11** (6.00 mL, 6.06 g, 42.0 mmol, 1.00 eq.) and methyl vinyl ketone (MVK, 3.90 mL, 3.24 g, 41.6 mmol, 0.99 eq.) in dry *tert*-butanol (40 mL, 1.0 M with respect to **S14**) at 0 °C. The mixture was stirred at this temperature for 30 min. Subsequently, additional potassium *tert*-butoxide (943 mg, 8.40 mmol, 0.20 eq., 20 mol%) was added, the reaction vessel was fitted with a reflux condenser, and the mixture was heated at reflux overnight. After 18 h, the mixture was poured into ice cold aqueous hydrochloric acid (1 M, 50 mL) and diluted with diethyl ether (50 mL). The aqueous phase was extracted with diethyl ether (3 × 20 mL), and the combined organic layers were washed with aq. NaOH (1 M, 3 × 30 mL), and brine (2 × 30 mL), dried over Na_2SO_4, filtered, and concentrated under reduced pressure to afford the crude product as a yellow oil. Purification by flash column chromatography on silica gel using hexanes/ethyl acetate (gradient elution: 19:1→ 17:3 → 4:1 *v/v*) as eluent afforded the title compound **S12** as a colorless oil (2.99 g, 24.0 mmol, 57% yield over two steps).**Physical state:** colorless oil.

TLC (SiO_2, hexanes/ethyl acetate 4:1, *v/v*): $\boldsymbol{R_F}$ = 0.39 ($KMnO_4$ stain).

^{1}H NMR (501 MHz, $CDCl_3$): δ (ppm) = 2.36 (t, J = 6.8 Hz, 2H), 2.34–2.29 (m, 2H), 1.95–1.88 (m, 2H), 1.92–1.91 (m, 3H), 1.75 (q, J = 1.4 Hz, 3H).

^{13}C NMR (126 MHz, $CDCl_3$): δ (ppm) = 199.3, 155.3, 131.2, 37.7, 32.9, 22.4, 21.6, 10.9.

HRMS *m*/*z* (GC-EI): calculated for $C_8H_{12}O^+$ $[M]^+$: 124.08827, found: 124.08842.

The spectroscopic data are in agreement with the data reported in the literature**.**

2,3,3-trimethylcyclohexan-1-one (S13)

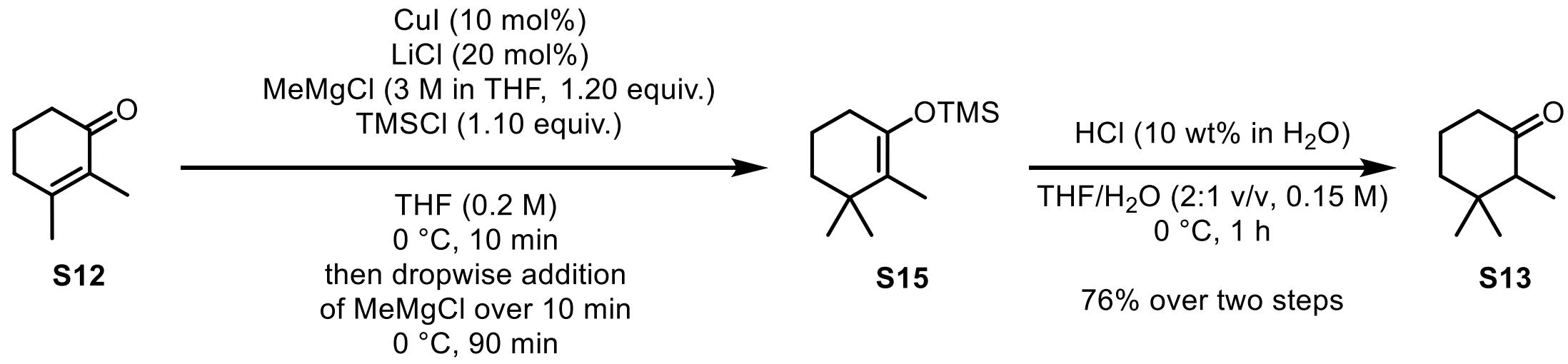

S13 was prepared according to procedures adapted from the literature.[11,12] A flame-dried 250 mL Schlenk flask under an atmosphere of argon was charged with LiCl (187 mg, 4.41 mmol, 0.20 eq.). The LiCl was dried with a heat-gun at 250 °C under reduced pressure for 1 min. After cooling to room temperature, CuI (420 mg, 2.21 mmol, 0.10 eq.) and dry THF (100 mL, 0.2 M with respect to the starting material **S12**) were transferred to the reaction vessel. Commercially available 2,3-dimethyl-2-cyclohexen-1-one (**S12**, 2.81 mL, 2.73 g, 22.0 mmol, 1.00 eq.) and trimethylsilylchloride (TMSCl, 3.10 mL, 2.64 g, 24.3 mmol, 1.10 eq.) were added and the resulting orange solution was stirred at 0 °C for 10 min. After the elapsed time, a solution of MeMgCl (3.0 M in THF, 8.80 mL, 26.4 mmol,

1.10 eq.) was added dropwise over 10 min. After the addition was complete, the reaction mixture was stirred for additional 90 min at 0 °C (*Note:* An aliquot of the reaction mixture was collected after 60 min reaction time and treated with either aq. sat. NH_4Cl solution or 10 wt% HCl in water. The individual samples were diluted with $CDCl_3$, filtered over Na_2SO_4, and the conversion was analyzed by ^1H NMR spectroscopy, indicating full consumption of the starting material. Treatment with NH_4Cl affords silyl enol ether **S15**, whereas treatment with aqueous HCl directly affords the desired ketone **S13**). After 90 min at 0 °C, aqueous hydrochloric acid (10 wt% in H_2O, 50 mL, 165 mmol, 7.48 eq.) was added and the resulting biphasic mixture was vigorously stirred for an additional hour at 0 °C. Then, the aqueous phase was separated, the organic phase was washed with a mixture of aq. sat. $NaHCO_3$ (20 mL) and aq. sat. NH_4Cl (100 mL) to remove copper ions as the intense blue $[Cu(NH_3)_4]$ complex. The aqueous phase was subsequently extracted with diethyl ether (4 × 50 mL), the combined organic layers were washed with water (50 mL), brine (50 mL), dried over Na_2SO_4, filtered, and concentrated under reduced pressure to afford the crude product as a pale yellow oil. Purification by flash column chromatography on silica gel using *n*-pentane/diethyl ether (gradient elution: 4:1→ 7:3 *v/v*) as eluent afforded the title compound **S13** as a colorless oil (2.35 g, 16.8 mmol,76% yield over two steps).

Physical state: colorless oil.
TLC (SiO_2, *n*-pentane/Et_2O 4:1, *v/v*): R_F = 0.50 ($KMnO_4$ stain).
^{1}H NMR (501 MHz, $CDCl_3$): δ (ppm) = 2.34 (dt, *J* = 13.5, 4.4 Hz, 1H), 2.27 (q, *J* = 6.8 Hz, 1H), 2.29–2.21 (m, 1H), 1.90 (ddt, *J* = 14.4, 6.6, 4.0 Hz, 1H), 1.85–1.75 (m, 1H), 1.67 (td, *J* = 12.6, 4.3 Hz, 1H), 1.59 (dtd, *J* = 13.5, 4.2, 1.4 Hz, 1H), 1.03 (s, 3H), 0.95 (d, *J* = 6.7 Hz, 3H), 0.75 (s, 3H).
^{13}C NMR (126 MHz, $CDCl_3$): δ (ppm) = 214.0, 54.8, 41.1, 39.8, 39.2, 29.8, 22.8, 21.0, 8.8.
HRMS *m/z* (GC-EI): calculated for $C_9H_{16}O^+$ $[M]^+$: 140.11957, found: 140.11958.

1,1,2-Trimethyl-3-methylenecyclohexane, (±)-4a

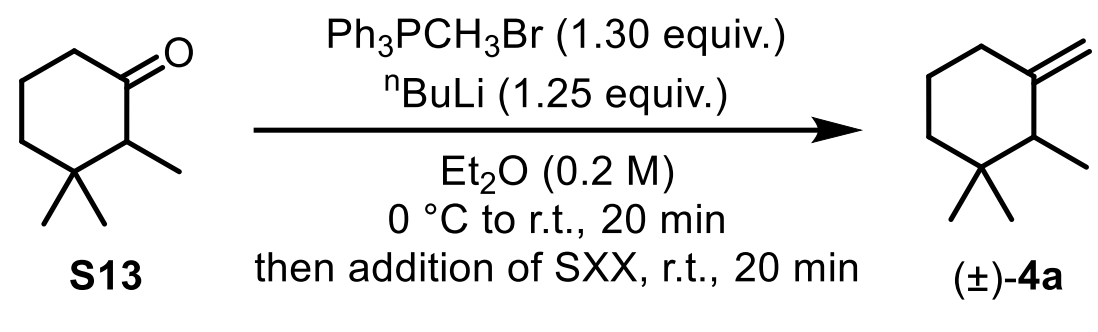

Methyltriphenylphosphonium bromide (3.72 g, 10.4 mmol, 1.30 eq.) was suspended in dry diethyl ether (40 mL, 0.2 M with respect to **S13**). The suspension was cooled to 0 °C and *n*-butyllithium (2.5 M solution in hexanes, 4.00 mL, 10.0 mmol, 1.25 eq.) was added (*Note:* The suspension changed from colorless to bright yellow upon addition.). The ice-bath was removed and the resulting orange suspension was stirred at room temperature for 20 min. After the elapsed time, ketone **S13** (1.12 g, 8.00 mmol, 1.00 eq.) was added dropwise (instantaneous precipitation of colorless Ph_3PO was observed upon addition). The reaction mixture was stirred at room temperature for an additional hour. Then, the reaction mixture was treated with water (5 mL) and filtered over a pad of silica gel which was subsequently eluted with *n*-pentane. The resulting colorless solution was carefully concentrated under reduced pressure (30 °C, 200 mbar) to afford the product as a colorless oil (^{1}H NMR analysis indicated presence of the desired γ-isomer).

Further purification by Kugelrohr distillation (oven temperature: T = 110–120 °C, p = 50 mbar) afforded alkene **(±)-4a** (780 mg, 5.64 mmol, 71% yield) as a colorless oil.

Physical state: colorless oil.
TLC (SiO_2, *n*-pentane): R_F = 0.80 ($KMnO_4$ stain).
^{1}H NMR (501 MHz, $CDCl_3$): δ (ppm) = 4.70 (dq, J = 2.4, 1.1 Hz, 1H), 4.55 (dt, J = 2.3, 1.1 Hz, 1H), 2.25 (dtt, J = 12.9, 4.9, 1.1 Hz, 1H), 1.99 (dddt, J = 12.8, 10.3, 5.2, 1.2 Hz, 1H), 1.92 (qt, J = 7.0, 1.3 Hz, 1H), 1.60–1.48 (m, 2H), 1.48–1.41 (m, 1H), 1.31 (ddd, J = 13.3, 10.2, 4.6 Hz, 1H), 0.97 (d, J = 6.9 Hz, 3H), 0.94 (s, 3H).
^{13}C NMR (126 MHz, $CDCl_3$): δ (ppm) = 152.5, 106.6, 47.2, 39.8, 35.5, 29.7, 24.1, 21.8, 12.9.
HRMS *m/z* (GC-EI): calculated for $C_{10}H_{18}^{+}$ $[M]^{+}$: 138.14030, found: 138.14037.

General Procedure of control experiments: A 5 ml vial was charged with IDPi **5d** (2.7 µmol, 2 mol%) and a magnetic stir bar under an atmosphere of argon. Dry toluene (0.107 mL) and PFTB (0.275 mmol, 2 eq.) added. The vial was cooled to −50 °C. The γ-product **4a** or the α-product **2a** (0.137 mmol, 1.0 eq.) was added dropwise and the reaction was stirred at desired temperature.
Afterwards, the reaction mixture was treated with triethylamine (0.257 mmol, 3.8 µL, 0.2 eq.). After the mixture was stirred for 10 min, the solvent of the reaction mixture was directly removed under reduced pressure. The product was purified by column chromatography on silica gel (pentane).

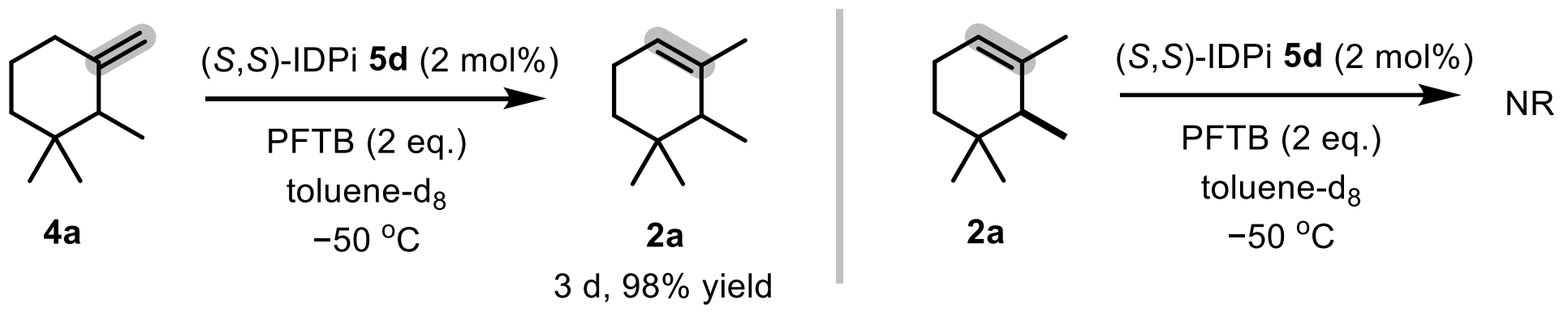

Figure S4. Control experiments performed using the γ-product **4a** and the α-product **2a** as the respective substrates.

Discussion of the results: Reaction monitoring revealed that both the α- and γ-cyclized products (**2a** and **4a**) were formed concurrently at an early stage, followed by the gradual and complete conversion of **4a** into the thermodynamically favored **2a**. This process was accompanied by a continuous increase in the enantioselectivity of **2a** and a corresponding decrease in that of **4a**, indicating an initial kinetic distribution followed by a selective isomerization. Independently resubjecting **2a** and **4a** to the catalytic conditions showed that only **4a** underwent complete conversion to **2a** (Figure S4), thereby identifying **4a** as a transient kinetic product.

9.2 PFTB effect

9.2.1 PFTB and IDPi 5d NMR titration

IDPi **5d** (32.8 mg, 0.0135 mmol, 1.00 eq.) was dissolved in 0.5 mL toluene-d_8 (final concentration 0.027 M) in an NMR tube. ^{1}H NMR, and ^{31}P NMR spectrums were measured at 223 K. Then, PFTB (1.35 M in toluene-d_8) was added in different equivalents. The mixture was vigorously mixed, and at every step, ^{1}H NMR, and ^{31}P NMR spectrum were measured at 223K. Based on the IDPi **5d** starting concentration [IDPi **5d**]$_0$, the relative [PFTB] was calculated.

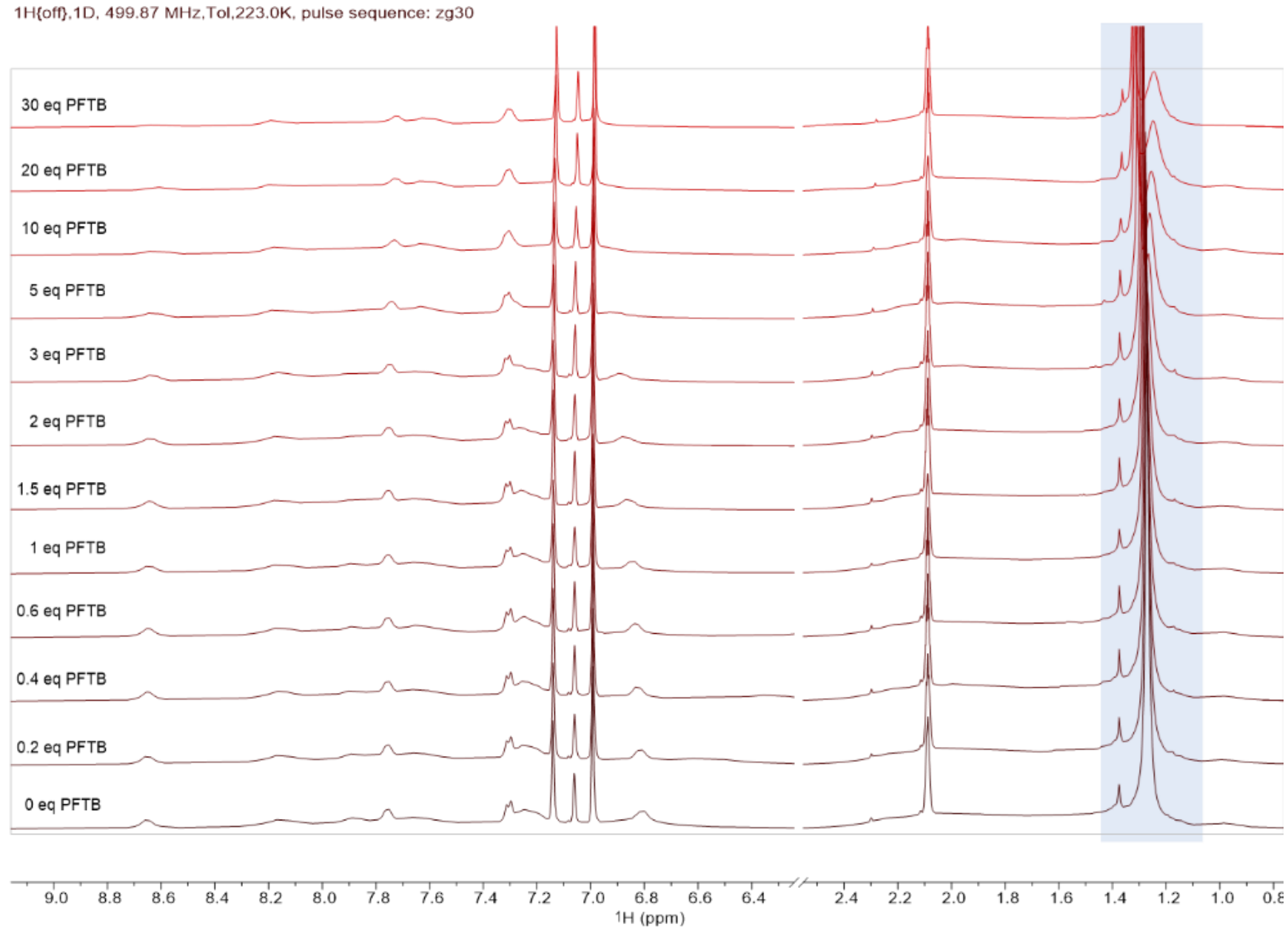

Figure S5. ^{1}H NMR titration between IDPi **5d** and PFTB ([IDPi **5d**] = 0.027 M, [PFTB] = 1.35 M, 223 K, 500 MHz, toluene-d_8).

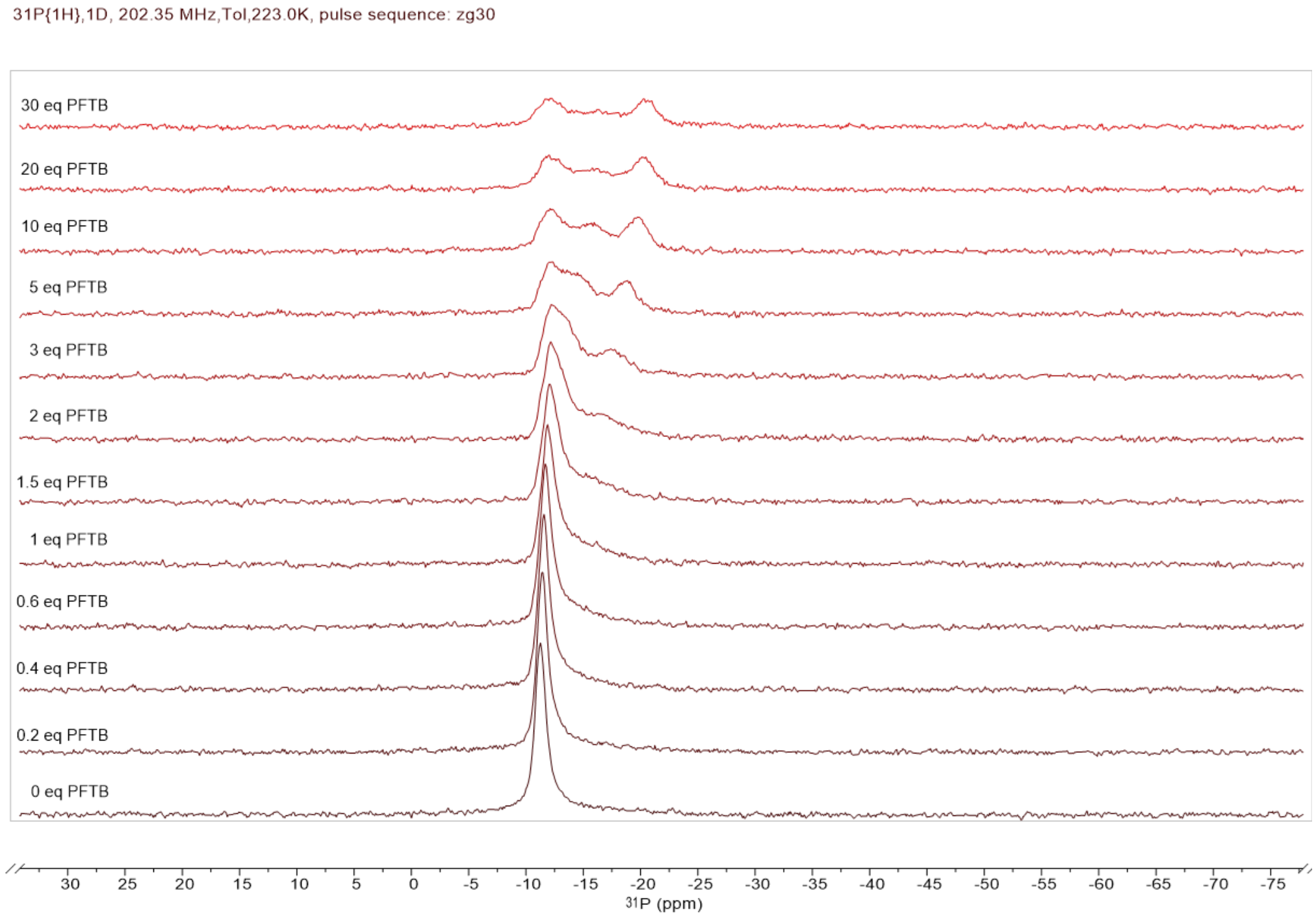

Figure S6. ^{31}P NMR titration between IDPi **5d** and PFTB ([IDPi **5d**] = 0.027 M, [PFTB] = 1.35 M, 223 K, 203 MHz, toluene-d_8).

9.2.2 Influence of PFTB equivalents on the reaction

To probe whether PFTB actively participates in the catalytic cycle, its reaction order was determined by variable time normalization analysis (VTNA).[13,14] Different equivalents of PFTB were applied to investigate their influence on reaction behavior in detail. The concentration changes of each component during the reaction were monitored by ^{1}H NMR spectroscopy, following the same procedure as described above for reaction monitoring, with the only variable being the equivalents of PFTB.

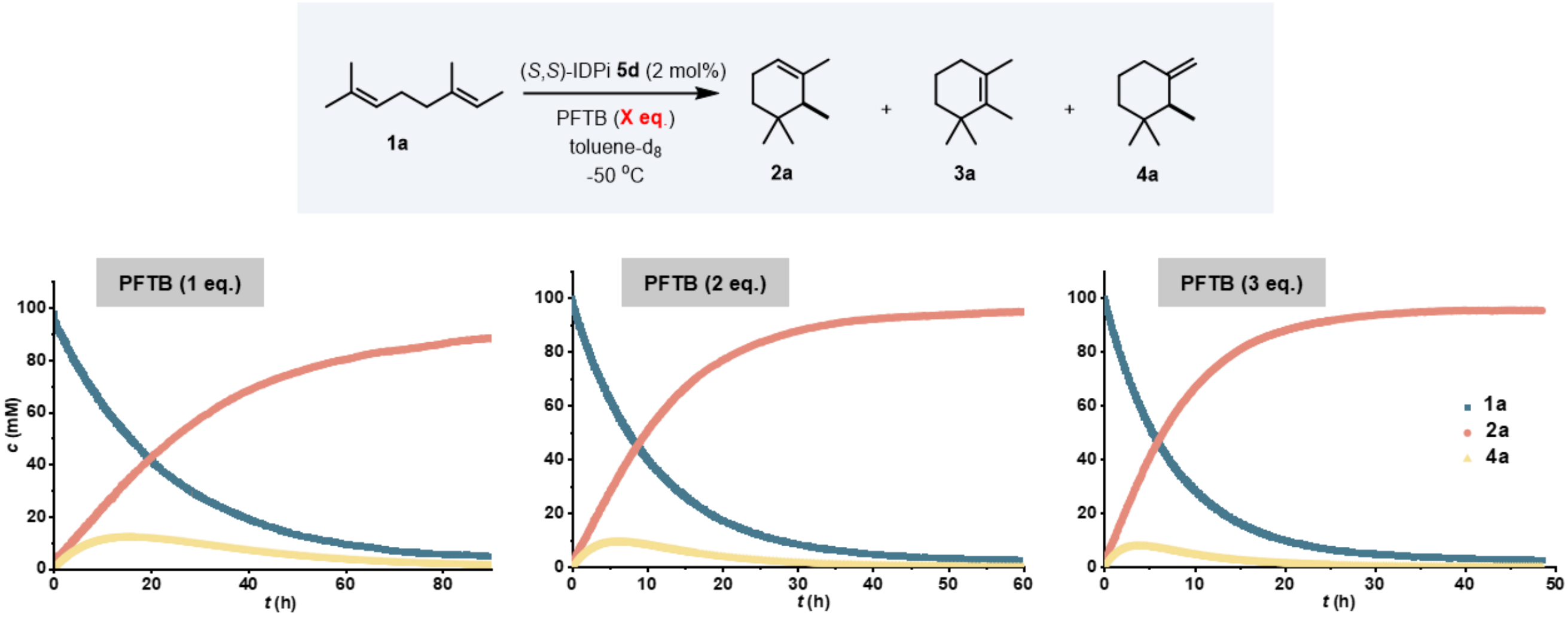

Figure S7. Concentration plots obtained from ^{1}H NMR spectroscopy reaction monitoring with different PFTB equivalents.

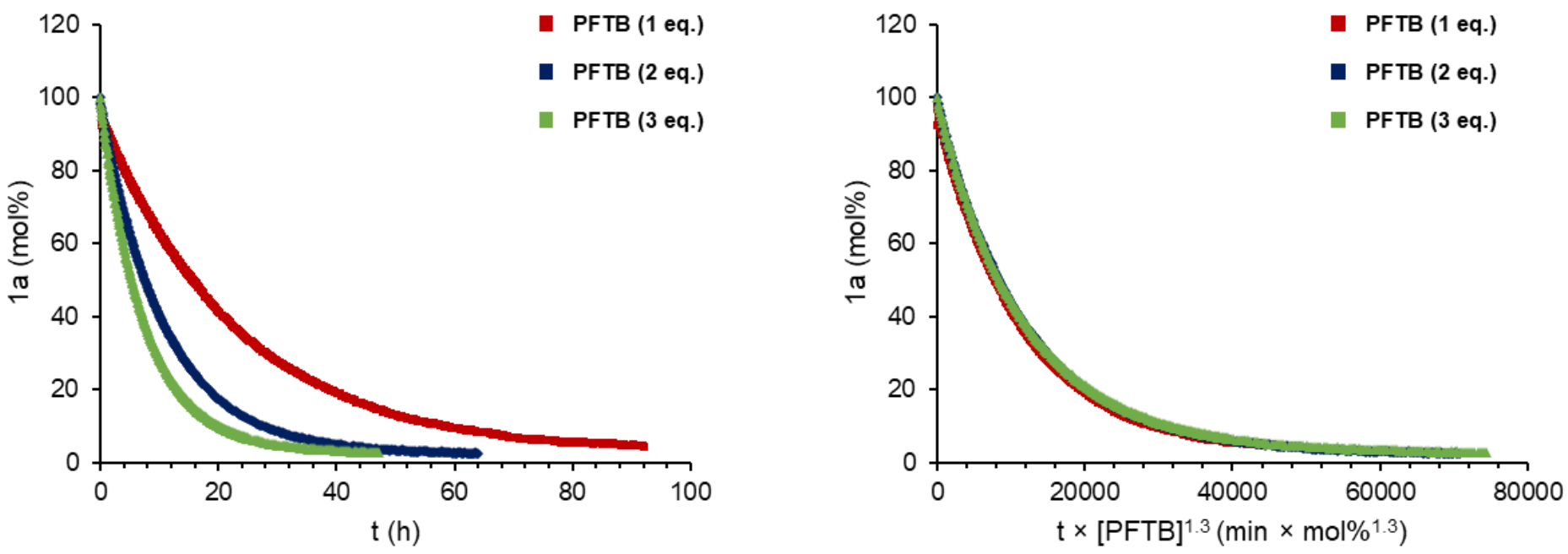

Figure S8. ^{1}H NMR concentration profiles in toluene-d_8 at 223 K in the presence of 1 eq., 2 eq., and 3 eq. of PFTB for **1a**, with time scales normalized to a 1.3[rd] order dependence on PFTB concentration.

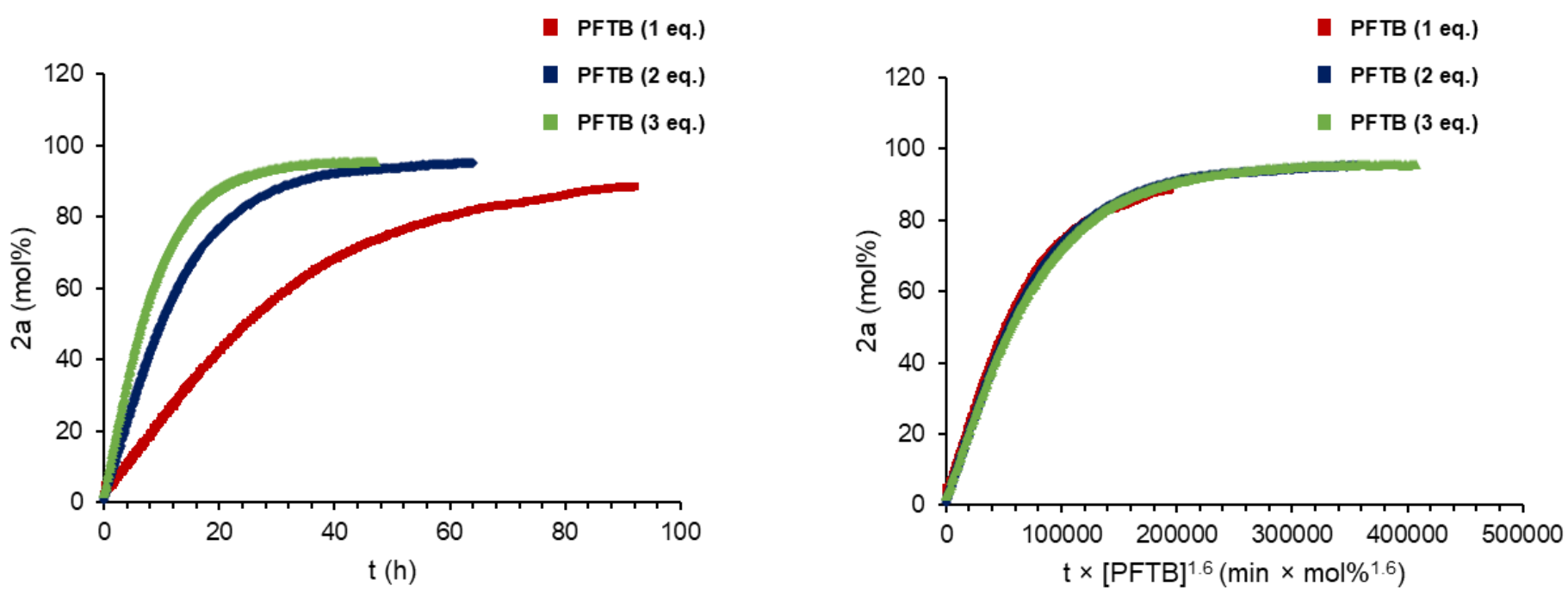

Figure S9. ^{1}H NMR concentration profiles in toluene-d_8 at 223 K in the presence of 1 eq., 2 eq., and 3 eq. of PFTB for **2a**, with time scales normalized to a 1.6[th] order dependence on PFTB concentration.

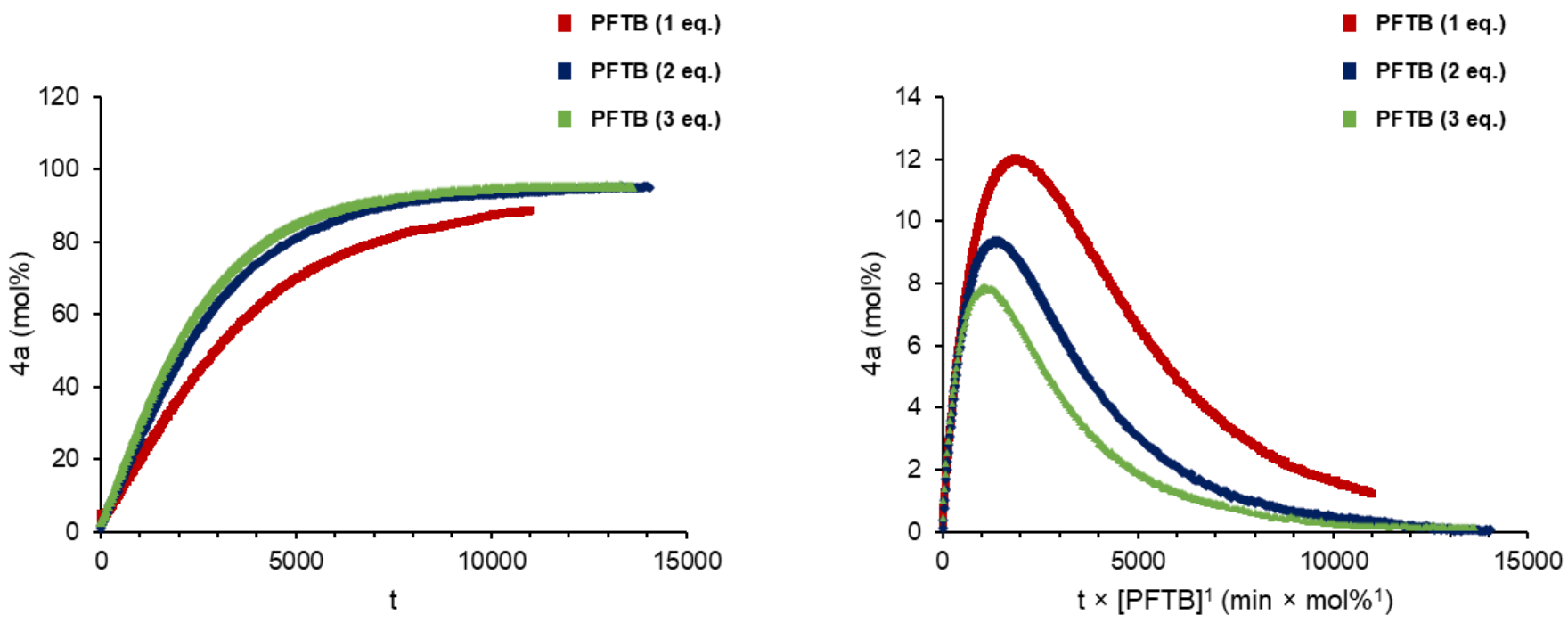

Figure S10. ¹H NMR concentration profiles in toluene-d_8 at 223 K in the presence of 1 eq., 2 eq., and 3 eq. of PFTB for **4a**, with time scales normalized to a first-order dependence on PFTB concentration.

Discussion of the results: Reaction order studies revealed that PFTB exhibits different apparent reaction orders with respect to various species, including substrate **1a** and products **2a** and **4a**. The reaction network involves not only the consumption of **1a** but also the isomerization of **4a** to **2a**, rendering the influence of PFTB on **2a** and **4a** complex and difficult to quantify. Therefore, only the reaction order of PFTB with respect to substrate **1a** is discussed. Notably, the reaction order of PFTB was determined to be 1.3, which suggests that more than one PFTB molecule may be involved in the rate-determining step, possibly through a cooperative effect.

9.2.3 Control Experiments

General Procedure: A 5 ml vial was charged with IDPi **5d** (2.7 µmol, 2 mol%) and a magnetic stir bar under an atmosphere of argon. Dry toluene (0.107 mL), PFTB (0.275 mmol, 2 eq.), and 1,4-dioxane (0.275 mmol, 2 eq.) added. The vial was cooled to −50 °C. The substrate **1** (0.137 mmol, 1.0 eq.) was added dropwise and the reaction was stirred at −50 °C for 3 days.

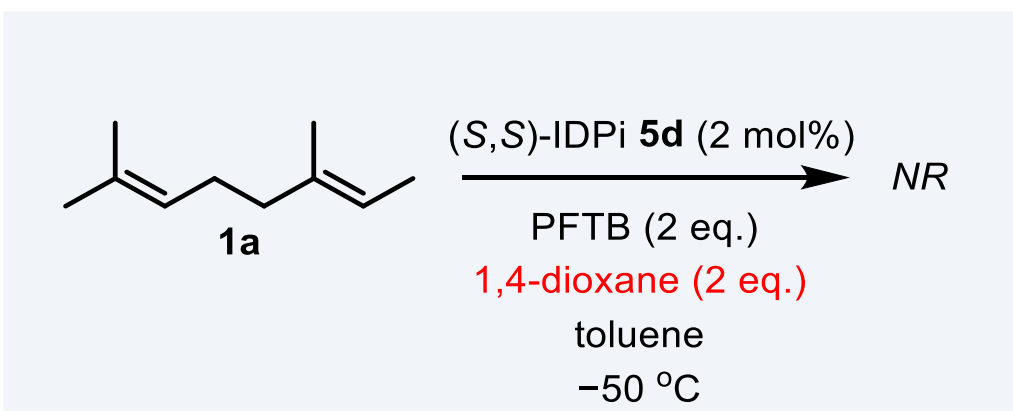

Consistent with our hypothesis (PFTB forms a hydrogen-bonding network with the IDPi catalyst), the addition of 1,4-dioxane (2 equiv.), a hydrogen-bonding disruptor, resulted in complete suppression of the reaction, suggesting that the catalytic system may be sensitive to disruption of hydrogen-bonding interactions.
A similar effect may also contribute to the reduced reactivity observed for substrates bearing carbonyl-containing functional groups (e.g., the ester and ketone functionalities in substrates **1r** and **1s**, respectively), although alternative explanations cannot be excluded.

9.2.4 Kinetic Isotope Effect (KIE)

Sample Preparation An oven-dried (80 °C, overnight) 3 mm NMR tube was charged with IDPi **5d** (2 mol%). Dry toluene-d_8 (0.107 mL) and PFTB-OH or PFTB-OD (0.275 mmol, 2 eq.) were added. The initial mixture was precooled to –78 °C in a dry ice/acetone bath. After addition of the substrate **1a** (0.137 mmol, 1.0 eq.), the NMR tube

was quickly turned upside down, vortexed, and transferred to the precooled NMR probe at 223 K (−50 °C). After quick shimming, single-scan ^{1}H and ^{31}P NMR spectra were acquired every 5 min until completion of the reaction.

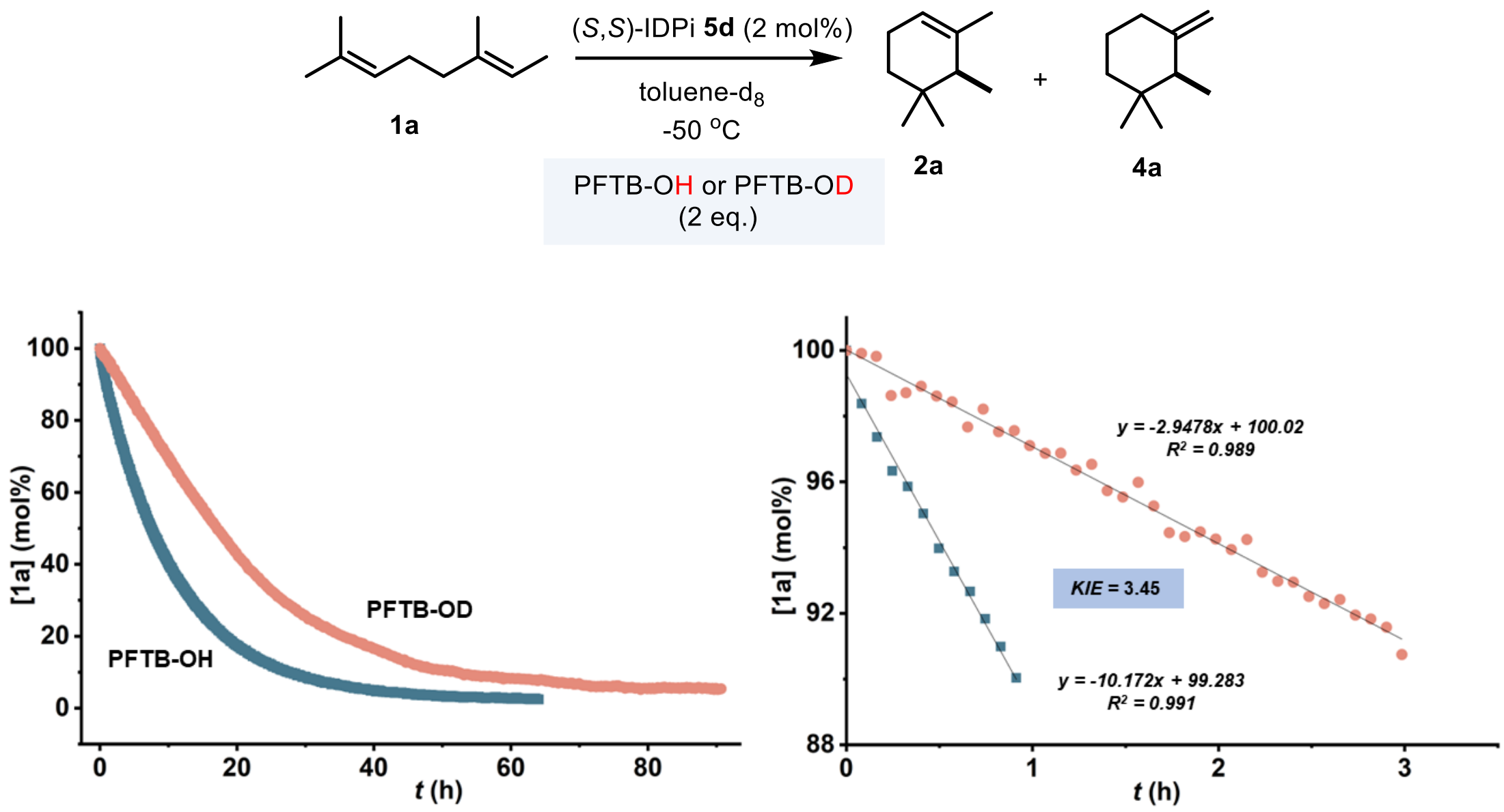

Figure S11. Left: Consumption comparison of starting material **1a** in PFTB-OH (blue) or PFTB-OD (orange) in the presence of 2 mol% IDPi **5d**. Right: slope for the initial 10% consumption of **1a**. The KIE value of 3.45 ± 0.02 for PFTB-OH (blue) and PFTB-OD (orange) was determined.

To further investigate the origin of the KIE, we performed additional calculations on different isotopic combinations (DFT details See Chapter 13). The calculated KIE values are as follows:

IDPi-D + PFTB-OH: KIE = 7.09

IDPi-H + PFTB-OD: KIE = 1.03

IDPi-D + PFTB-OD: KIE = 7.33

These results indicate that the large KIE primarily originates from deuteration at the IDPi catalyst rather than from deuteration of PFTB. Therefore, the computational results support the reviewer's suggestion that the observed isotope effect may largely arise from proton/deuteron substitution at the catalyst site, potentially as a consequence of H/D exchange processes in solution.

We also note that the experimentally observed initial-rate KIE (3.45) is significantly smaller than the calculated KIE associated with IDPi-D (7.09–7.33). A plausible explanation is that rapid H/D exchange between PFTB and IDPi generates a mixed population of IDPi-H and IDPi-D during the early stage of the reaction.

9.3 Deuterium Labeling study

Sample Preparation: An oven-dried (80 °C, overnight) 3 mm NMR tube was charged with IDPi **5d** (2 mol%). Dry toluene-d_8 (0.107 mL) and PFTB-OD (0.275 mmol, 2 eq.) were added. The initial mixture was precooled to –78 °C in a dry ice/acetone bath. After addition of the substrate **1a** (0.137 mmol, 1.0 eq.), the NMR tube was quickly turned upside down, vortexed, and transferred to the precooled NMR probe at 223 K (–50 °C). After quick shimming, single-scan ^{1}H and ^{31}P NMR spectra were acquired every 5 min until completion of the reaction.

After 3 days, the reaction mixture was treated with triethylamine (0.257 mmol, 3.8 µL, 0.2 eq.). After the mixture was stirred for 10 min, the solvent of the reaction mixture was directly removed under reduced pressure. The product was purified by column chromatography on silica gel (pentane), then analyzed by ^{1}H NMR and chiral GC to determine regioisomeric ratio (r.r.) and the enantiomeric ratio (e.r.). The isolated product **2a** was subsequently characterized by ^{1}H, ^{13}C, $^{2}H\{^{1}H\}$, and 2D-NMR spectroscopy.

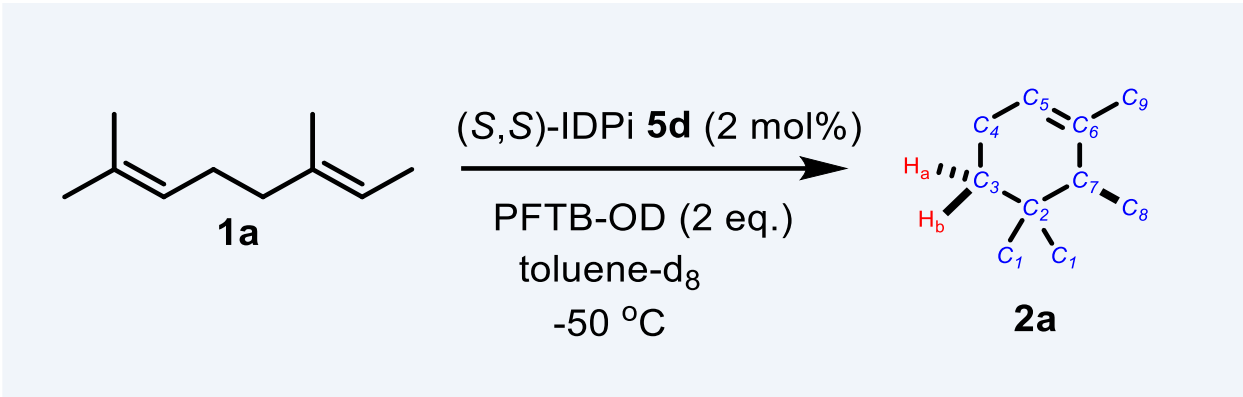

	Isotope distribution		Isotope distribution
$C_3D_aH_b$	0	C_9H_3	59%
$C_3D_aD_b$	0	C_9H_2D	16%
$C_3H_aH_b$	77%	C_9HD_2	17%
$C_3H_aD_b$	23%	C_9D_3	8%

Figure S12. Isotope distribution of **2a** based on the following NMR data.

Analysis of the products obtained from the reaction conducted with PFTB–OD by ^{1}H and $^{2}H\{^{1}H\}$ NMR revealed deuterium incorporation at the C_3-equatorial position (H_b). The observed stereochemical outcome can be rationalized in the context of a concerted (potentially asynchronous) cyclization model, as commonly proposed for acid-catalyzed terpene cyclizations proceeding through preorganized chair-like transition states. Moreover, the newly formed C_7 stereocenter exhibits a syn relationship between Hb and the methyl group, reflecting an anti-parallel addition of the electrophilic and nucleophilic termini across the olefin. In addition, significant deuterium enrichment was observed at the C_9 methyl group, whereas no deuterium incorporation was detected at C_5. This labeling pattern suggests that, following the initial formation of the γ-product, reversible protonation–deprotonation processes occur, gradually converting the γ-isomer into the thermodynamically favored α-isomer. Once formed, the α-product appears to be relatively stable under the reaction conditions, as further protonation would be expected to result in deuterium incorporation at C_5, which was not observed experimentally. This interpretation is consistent with the reaction-monitoring studies.

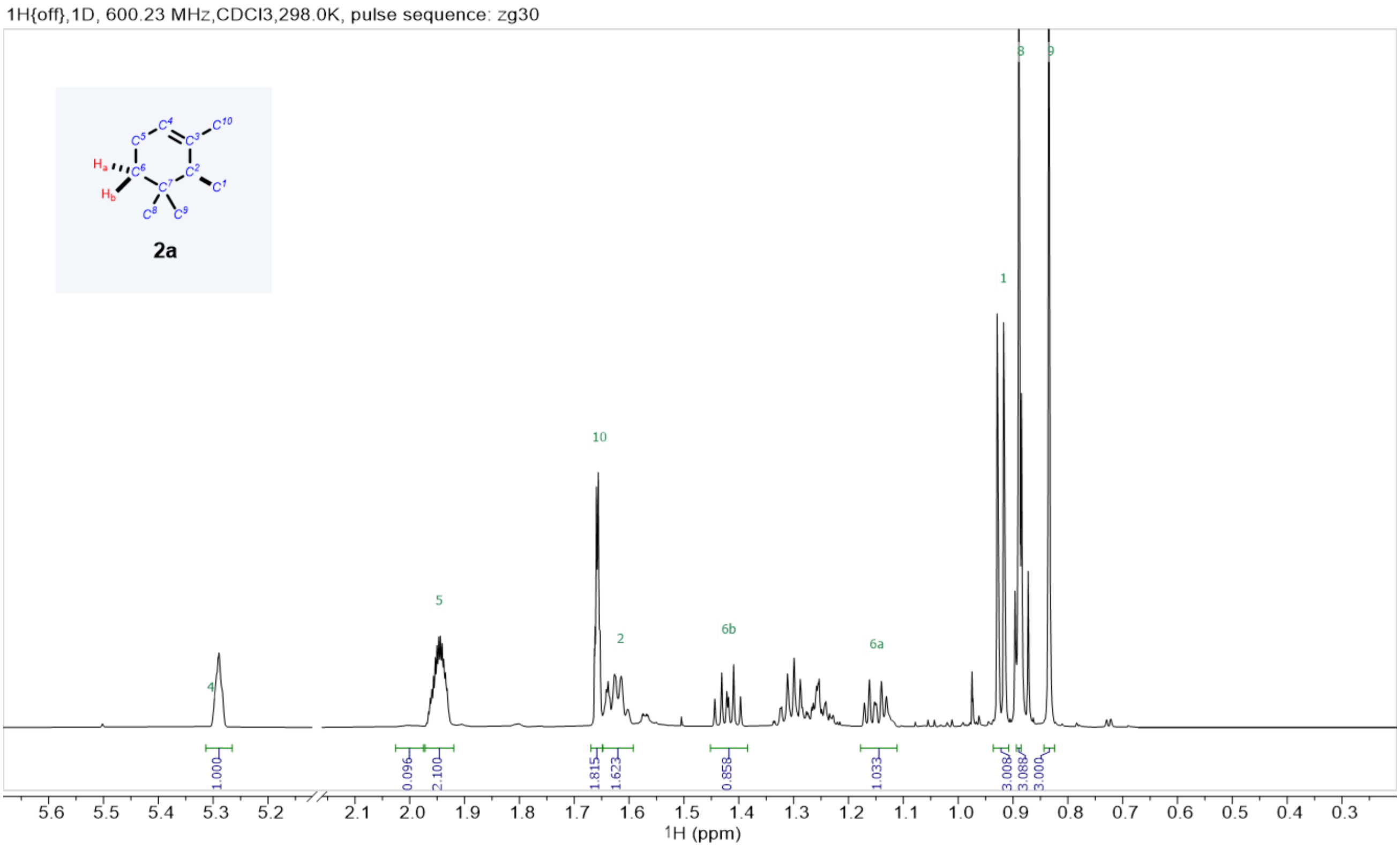

Figure S13. ^{1}H NMR spectrum of *a*-product **2a** and its proton signal assignments (600 MHz, $CDCl_3$, 298K).

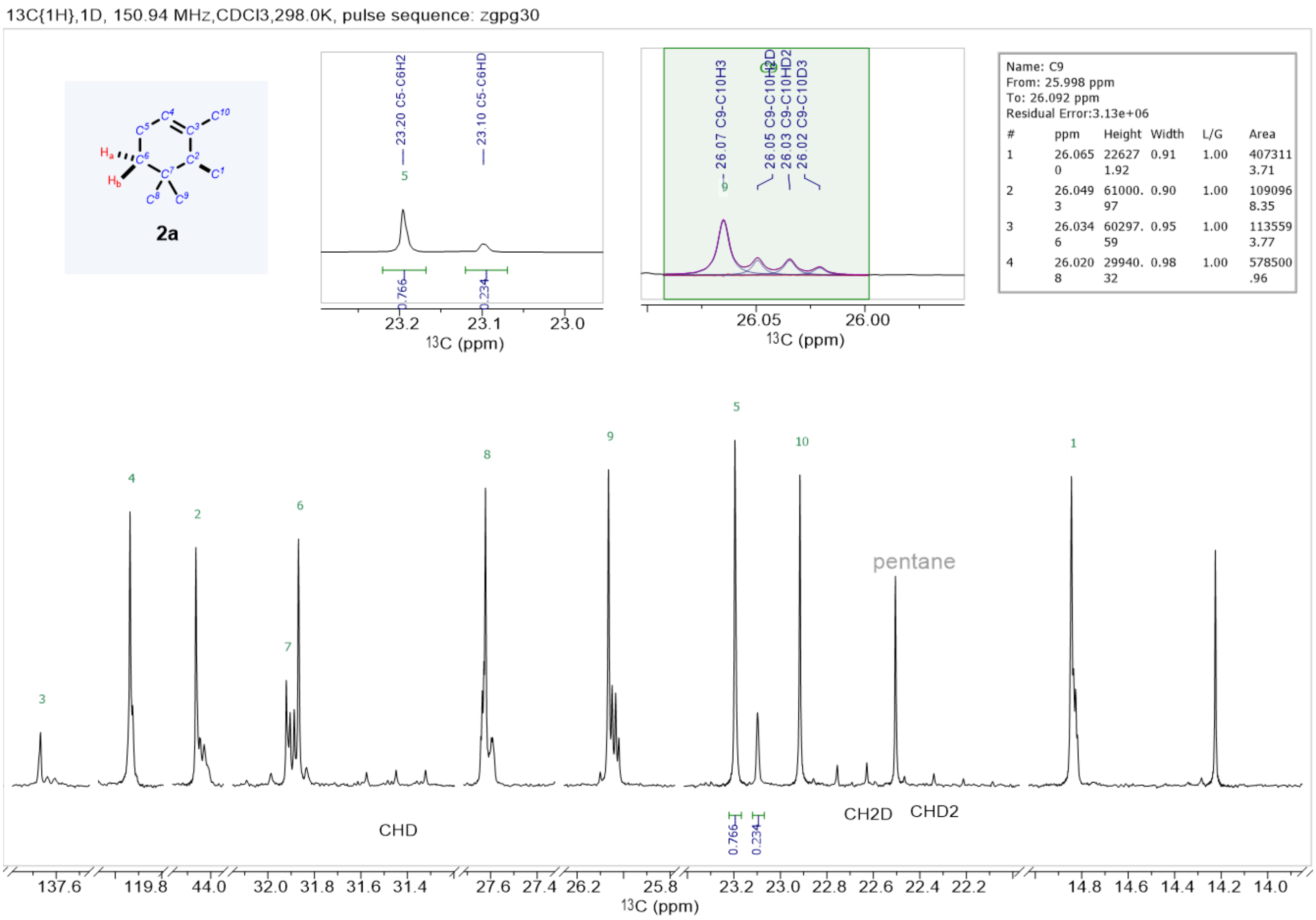

Figure S14. ^{13}C{^{1}H} NMR spectrum of *a*-product **2a** and its signal assignments (151 MHz, $CDCl_3$, 298K).

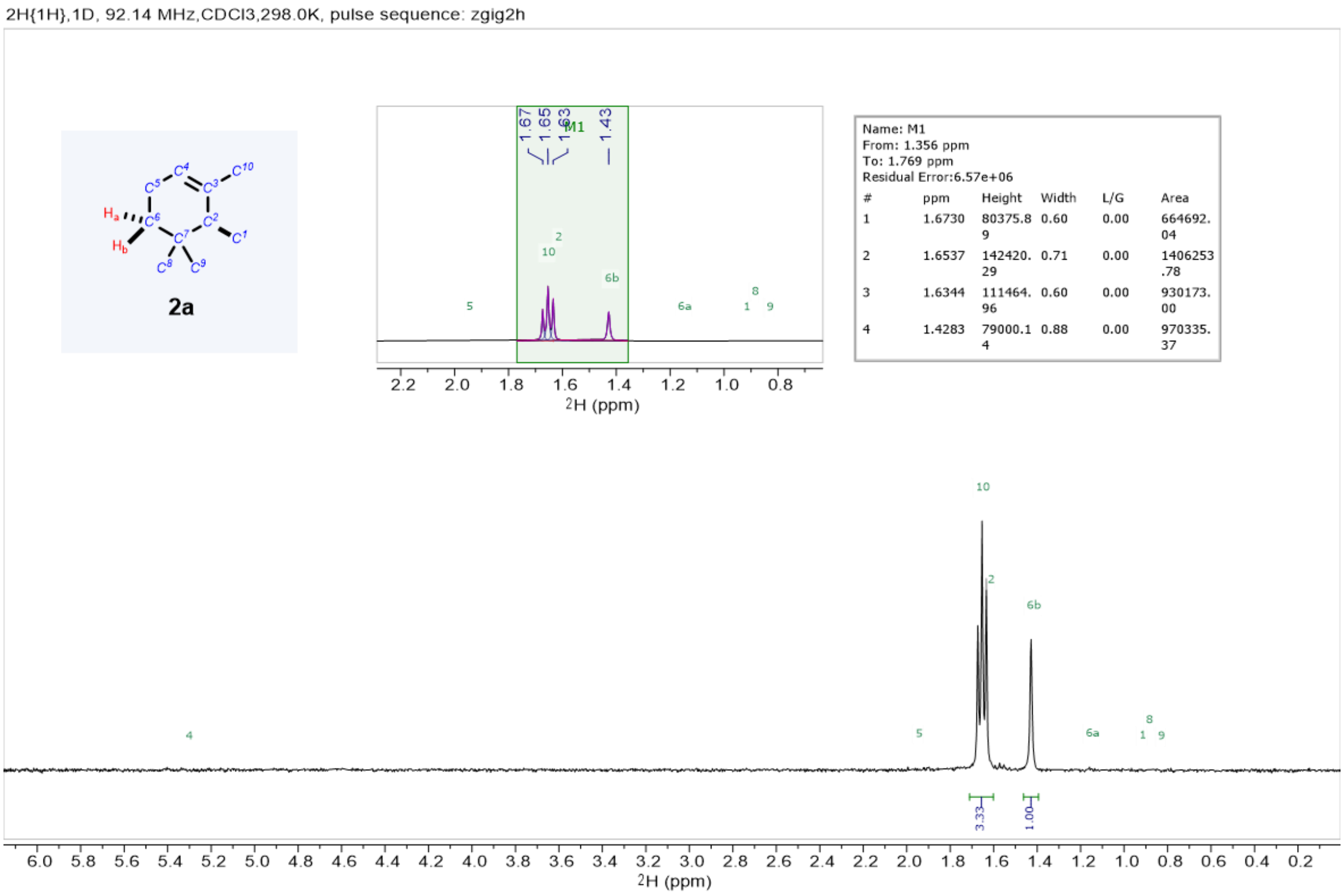

Figure S15. $^2H\{^1H\}$ NMR spectrum of *a*-product **2a** and its signal assignments (92 MHz, $CDCl_3$, 298K).

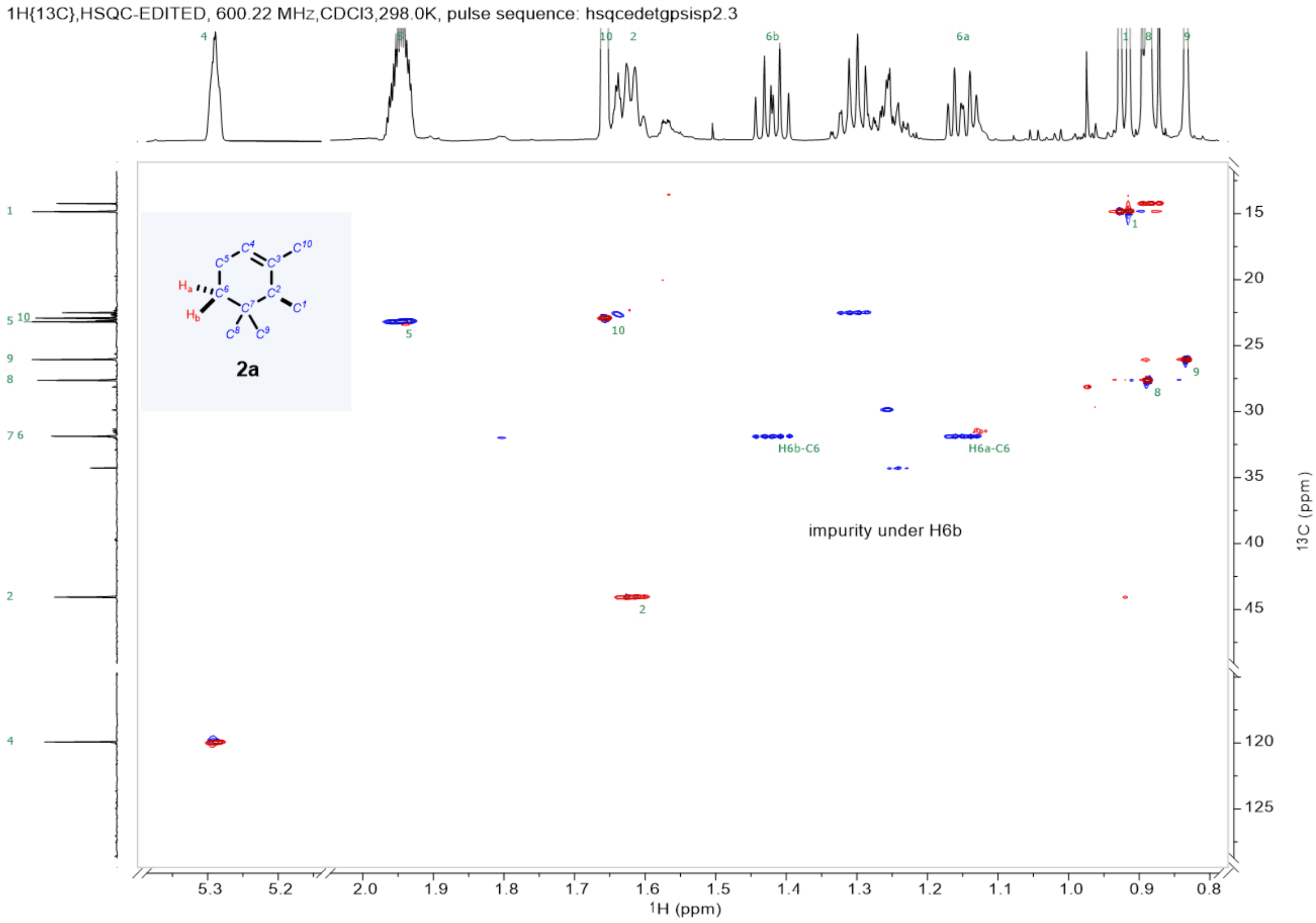

Figure S16. $^1H\{^{13}C\}$ HSQC NMR spectrum of *a*-product **2a** and its signal assignments (600 MHz, $CDCl_3$, 298K).

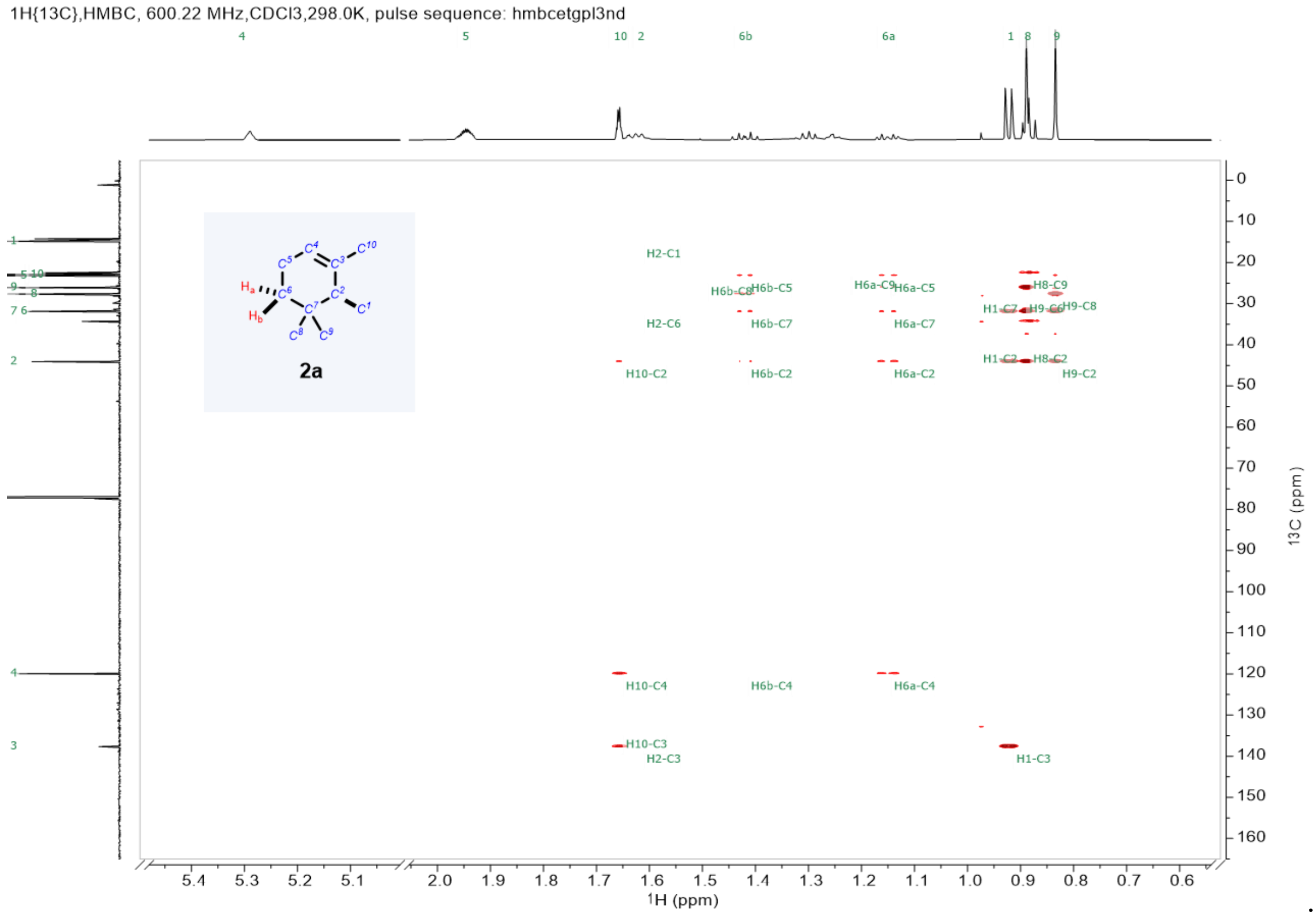

Figure S17. $^{1}H\{^{13}C\}$ HMBC NMR spectrum of *a*-product **2a** and its signal assignments (600 MHz, $CDCl_3$, 298K).

9.4 β-Secondary deuterium kinetic isotope effect (β-DKIE)

9.4.1 Synthesis of deuterated substrates 1a-d_1 and 1a-d_7

Synthesis of 3-methylbut-3-en-2-ol (S17)

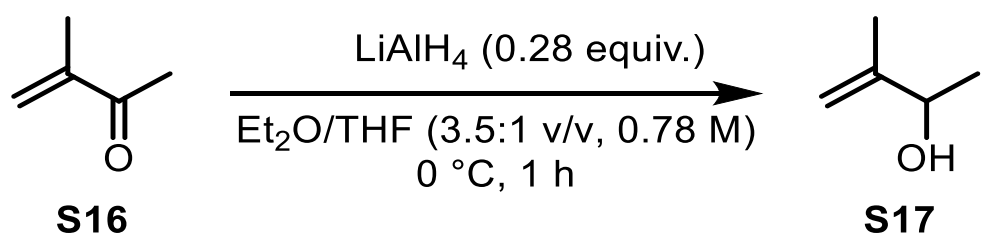

Compound **S17** was prepared according to an adapted literature procedure.[15,16] $LiAlH_4$ (1 M in THF, 17.0 mL, 17.0 mmol, 0.28 eq.; corresponding to 645 mg $LiAlH_4$) was added to a stirred solution of 3-methyl-3-buten-2-one (**S16**, 5.94 mL, 5.05 g, 60.0 mmol, 1.0 eq.) in dry diethyl ether (60 mL, 1 M) at 0 °C under an atmosphere of argon. ^{1}H NMR spectroscopic analysis of the crude reaction mixture indicated full consumption of starting material and formation of the desired product. The reaction mixture was cooled to 0 °C and diluted with diethyl ether (50 mL). A Fieser work-up was performed: water (0.65 mL) was added slowly, followed by aqueous sodium hydroxide (15 wt%, 0.65 mL), and another portion of water (1.95 mL). The mixture was warmed to room temperature and stirred for 15 min. Subsequently, anhydrous Na_2SO_4 (10 g) was added and the resulting colorless suspension was stirred for another 15 min. Then, the mixture was filtered over Celite® 545 and the filter cake was washed with diethyl ether (3 × 50 mL). The filtrate was concentrated under reduced pressure (300 mbar, 30 °C, *Caution*: product is volatile) to afford the crude product (**S17**, 5.16 g, 59.9 mmol) as a colorless oil, which was used in the next step without further purification.

Physical state: colorless oil.

TLC (SiO_2, hexanes/ethyl acetate 4:1, *v/v*): $\boldsymbol{R_F}$ = 0.42 ($KMnO_4$ stain).

^{1}H NMR (501 MHz, $CDCl_3$): δ (ppm) = 4.95 (dt, *J* = 2.0, 1.0 Hz, 1H), 4.79 (p, *J* = 1.6 Hz, 1H), 4.24 (q, *J* = 6.5 Hz, 1H), 1.74 (t, *J* = 1.1 Hz, 3H), 1.64 – 1.53 (m, 1H), 1.28 (d, *J* = 6.5 Hz, 3H).

^{13}C{^{1}H} NMR (126 MHz, $CDCl_3$): δ (ppm) = 149.1, 109.7, 71.7, 21.9, 18.0.

HRMS *m/z* (GC-EI): calculated for $C_5H_{10}O^+$ $[M]^+$: 86.07262, found: 86.07262.

The spectroscopic data are in agreement with the data reported in the literature.

Ethyl (*E*)-4-methylhex-4-enoate (S18)

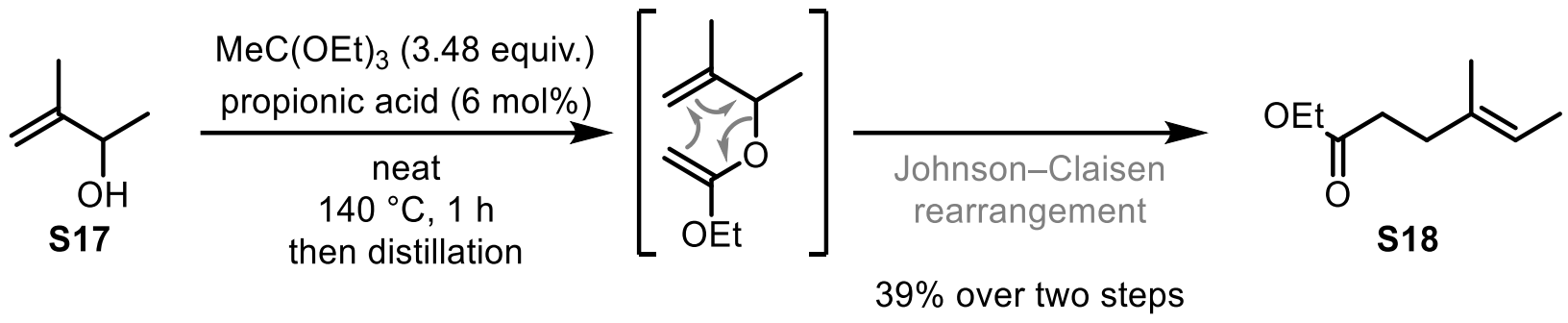

The reaction was performed according to procedures reported in the literature.[15,17] Allylic alcohol **S17** (3.65 g, 42.4 mmol, 1.00 eq.) and triethylorthoacetate (27.0 mL, 23.9 g, 147 mmol, 3.48 eq.) were transferred to a 100 mL round-bottom flask equipped with a PTFE-coated magnetic stir bar. The reaction vessel was closed with a rubber septum and flushed with argon. Propionic acid (0.19 mL, 189 mg, 2.55 mmol, 0.06 eq., 6 mol%) was added and the flask was fitted with a Vigreux column and a micro distillation apparatus. The reaction vessel was immersed in a preheated oil bath at 140 °C and heated at this temperature under distillative removal of ethanol. After 1 h reaction time, the product was purified by distillation under reduced pressure (b.p. 130 °C at 27 mbar) affording the title compound as a colorless oil (2.55 g, 16.3 mmol, 39% yield over two steps).

Physical state: colorless oil.

TLC (SiO_2, *n*-pentane/diethyl ether 1:1, *v/v*): $\boldsymbol{R_F}$ = 0.84 ($KMnO_4$ stain).

^{1}H NMR (501 MHz, $CDCl_3$): δ (ppm) = 5.24 (qq, *J* = 6.6, 1.4 Hz, 1H), 4.12 (q, *J* = 7.1 Hz, 2H), 2.41 – 2.35 (m, 2H), 2.32–2.27 (m, 2H), 1.61 (t, *J* = 1.3 Hz, 3H), 1.56 (dq, *J* = 6.6, 1.2 Hz, 3H), 1.25 (t, *J* = 7.1 Hz, 3H).

^{13}C{^{1}H} NMR (126 MHz, $CDCl_3$): δ (ppm) = 173.7, 134.2, 119.4, 60.4, 34.8, 33.4, 15.7, 14.4, 13.5.

HRMS *m/z* (GC-EI): calculated for $C_9H_{16}O_2^+$ $[M]^+$: 156.11448, found: 156.11464.

The spectroscopic data are in agreement with the data reported in the literature.

(*E*)-4-Methylhex-4-en-1,1-*d*$_2$-1-ol (S19)

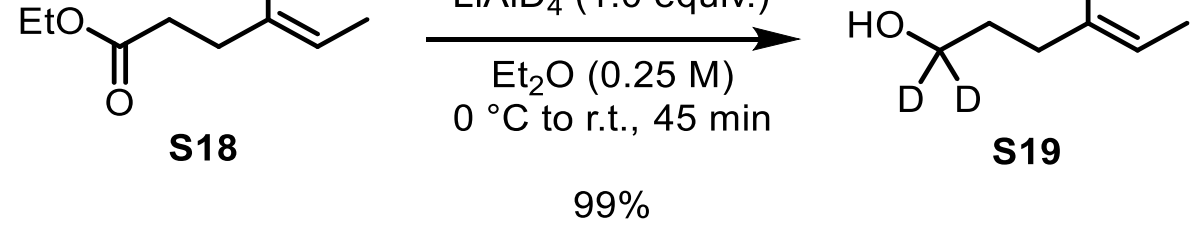

The reaction was performed according to a slightly modified literature procedure reported by Dickschat and coworkers:[18] Ester **S18** (2.50 g, 16.0 mmol, 1.00 eq.) was dissolved in dry diethyl ether (50 mL, 0.25 M) and the resulting colorless solution was cooled to 0 °C. Then, solid $LiAlD_4$ (672 mg) was added in one portion. The ice bath was removed and the resulting colorless solution was stirred at room temperature for 45 min. After the elapsed time, the reaction mixture was cooled to 0 °C and diluted with diethyl ether (20 mL). A Fieser work-up was performed: water (0.67 mL) was slowly added, followed by aqueous sodium hydroxide (15 wt%, 0.67 mL), and another portion

of water (2.0 mL). The mixture was warmed to room temperature and anhydrous $MgSO_4$ was added. The suspension was filtered over Celite® 545 and the filter cake was subsequently washed with diethyl ether (3 × 50 mL). The filtrate was concentrated under reduced pressure to afford crude alcohol **S19** (1.85 g, 15.9 mmol, 99%) as a colorless oil, which was used in the next step without any further purification.

Physical state: colorless oil.
TLC (SiO_2, *n*-pentane/diethyl ether 1:1, *v/v*): $\boldsymbol{R_F}$ = 0.27 ($KMnO_4$ stain).
^{1}H NMR (501 MHz, $CDCl_3$): δ (ppm) = 5.29–5.21 (m, 1H), 2.06 (tt, *J* = 7.4, 1.2 Hz, 2H), 1.65 (t, *J* = 7.5 Hz, 2H), 1.61 (t, *J* = 1.2 Hz, 3H), 1.57 (dq, *J* = 6.8, 1.2 Hz, 3H), 1.36 (br s, 1H, OH).
^{13}C{^{1}H} NMR (126 MHz, $CDCl_3$): δ (ppm) = 135.6, 119.0, 62.3 (p, $^1J_{C\text{–}D}$ = 21.6 Hz), 36.1, 30.7, 15.7, 13.5.
HRMS *m/z* (GC-EI): calculated for $C_7H_{12}OD_2^+$ $[M]^+$: 116.11647, found: 116.11656.

(*E*)-(4-Methylhex-4-en-1-yl-1,1-*d*$_2$)triphenylphosphonium iodide (S22)

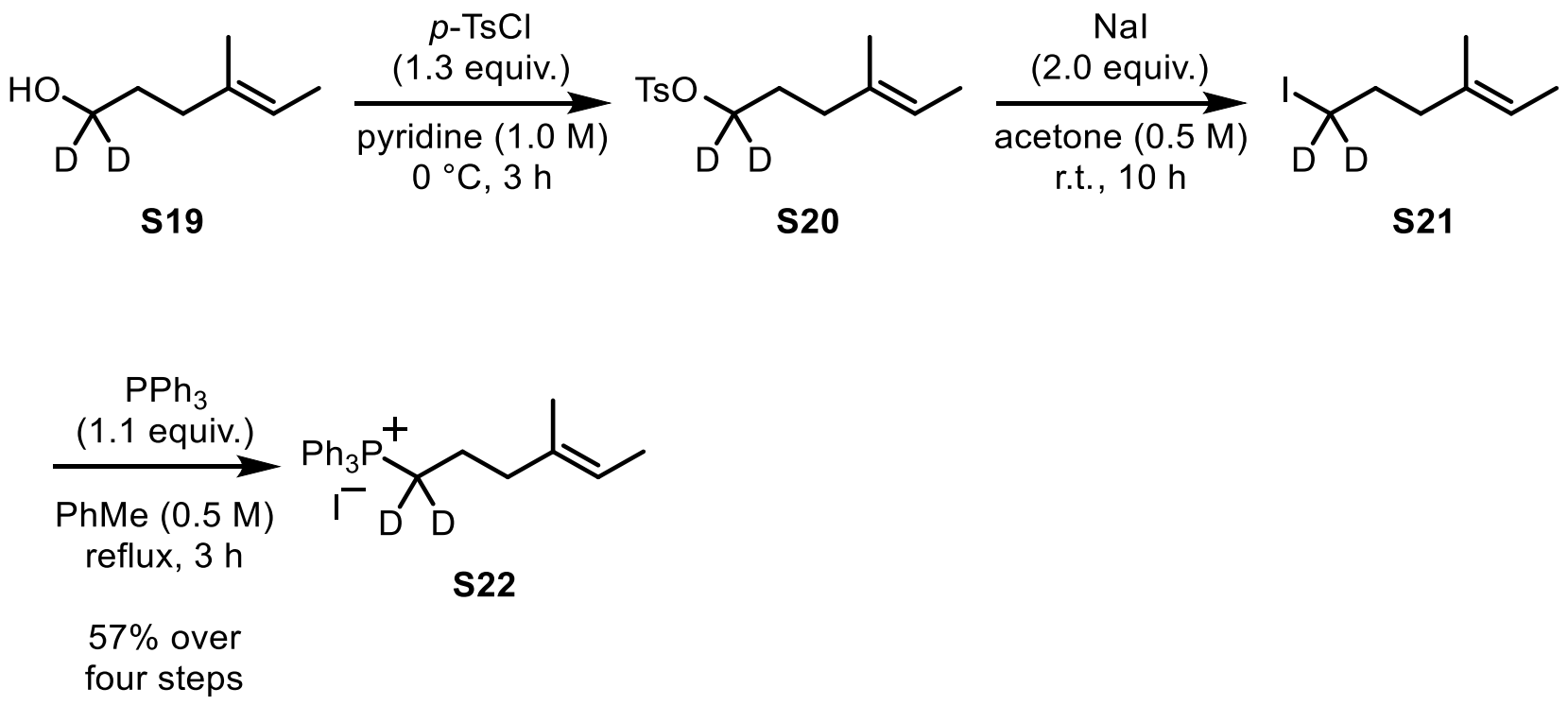

Compound **S22** was synthesized according to a sequence reported by Dickschat and coworkers for a similar substrate:[18] Crude alcohol **S19** (1.74 g, 15.0 mmol, 1.00 eq.) was dissolved in dry pyridine (15 mL, 1 M) and the resulting colorless solution was cooled to 0 °C. Then, *p*-toluenesulfonyl chloride (3.72 g, 19.5 mmol, 1.30 eq.) was added portion wise and the resulting turbid yellow reaction mixture was stirred at 0 °C for 3 h (a colorless precipitate, pyridinium hydrochloride, formed over the course of the reaction). After the elapsed time, the reaction mixture was diluted with diethyl ether (50 mL) and poured onto ice. The aqueous phase was extracted with diethyl ether (3 × 50 mL) and the combined organic layers were washed with aqueous hydrochloric acid (10%, 2 × 50 mL), water (50 mL) and brine (50 mL), dried over Na_2SO_4, filtered and concentrated under reduced pressure to afford tosylate **S20** as a pale yellow oil. **^{1}H NMR** (501 MHz, $CDCl_3$): δ (ppm) = 7.79 (app d, *J* = 8.3 Hz, 2H), 7.34 (app d, *J* = 8.0 Hz, 2H), 5.13 – 5.04 (m, 1H), 2.45 (s, 3H), 1.97 (app t, *J* = 7.3 Hz, 2H), 1.71 (app t, *J* = 7.5 Hz, 2H), 1.53 – 1.51 (m, 3H), 1.51 – 1.49 (m, 3H). **HRMS** *m/z* (ESI): calculated for $C_{14}H_{18}O_3SD_2Na^+$ $[M+Na]^+$: 293.11509, found: 293.11510.

Crude tosylate **S20** (4.06 g, 15.0 mmol, 1.00 eq.) was dissolved in dry acetone (30 mL) and sodium iodide (4.50 g, 30.0 mmol, 2.0 eq.) was added. The resulting yellow suspension was stirred at room temperature overnight. After 10 h reaction time, the orange-red reaction mixture was diluted with *n*-pentane (50 ml) and filtered over a pad of silica gel, eluting with *n*-pentane (3 × 50 mL). The filtrate was concentrated under reduced pressure to afford crude iodide **S21** (3.06 g, 13.5 mmol, 90% yield over two steps) as a pale red oil which was used in the subsequent step

without further purification. **^{1}H NMR** (501 MHz, $CDCl_3$): δ (ppm) = 5.34 – 5.19 (m, 1H), 2.07 (app t, J = 6.8 Hz, 2H), 1.90 (app t, J = 7.3 Hz, 2H), 1.60 – 1.58 (m, 3H), 1.58 – 1.56 (m, 3H).

Triphenylphosphine (4.25 g, 16.2 mmol, 1.20 eq.) was dissolved in toluene (30 mL, 0.45 M with respect to iodide **S21**) and crude iodide **S21** (3.06 g, 13.5 mmol, 1.00 eq.) was added. The resulting yellow reaction mixture was refluxed for 3 h and subsequently cooled to room temperature. The turbid reaction mixture (colorless gummy/gooey precipitate) was diluted with *n*-pentane and the resulting mixture was cooled to −78 °C and vigorously stirred which led to the formation of a colorless fine precipitate. The precipitate was filtered and washed with toluene and *n*-pentane. The resulting solid was collected and dried under reduced pressure to provide Wittig salt **S22** (3.77 g, 7.72 mmol, 57% yield over four steps from ester **S18**) as a colorless solid. **^{1}H NMR** (501 MHz, $CDCl_3$): δ (ppm) = 7.83 – 7.76 (m, 9H), 7.74 – 7.68 (m, 6H), 5.24 (q, J = 6.7 Hz, 1H), 2.29 (t, J = 7.2 Hz, 2H), 1.73 (q, J = 7.7 Hz, 2H), 1.52 (d, J = 6.7 Hz, 3H), 1.43 (s, 3H). **^{13}C{^{1}H} NMR** (126 MHz, $CDCl_3$): δ (ppm) = 135.3 (d, J = 3.2 Hz), 133.8 (d, J = 10.0 Hz), 133.2, 130.7 (d, J = 12.5 Hz), 121.3, 118.6, 117.9, 39.5 (d, J = 15.7 Hz), 20.4 (d, J = 4.2 Hz), 15.6, 13.5 (*Note:* The CD_2-group was not observed in the ^{13}C NMR spectrum). **^{31}P NMR** (203 MHz, $CDCl_3$): δ (ppm) = 24.47 (s, 1P). **HRMS** *m*/*z* (ESI): calculated for $C_{25}H_{26}D_2P^+$ $[M]^+$: 361.20487, found: 361.20492.

(*E*)-2,6-dimethylocta-2,6-diene-3-*d* (1a-*d*$_1$)

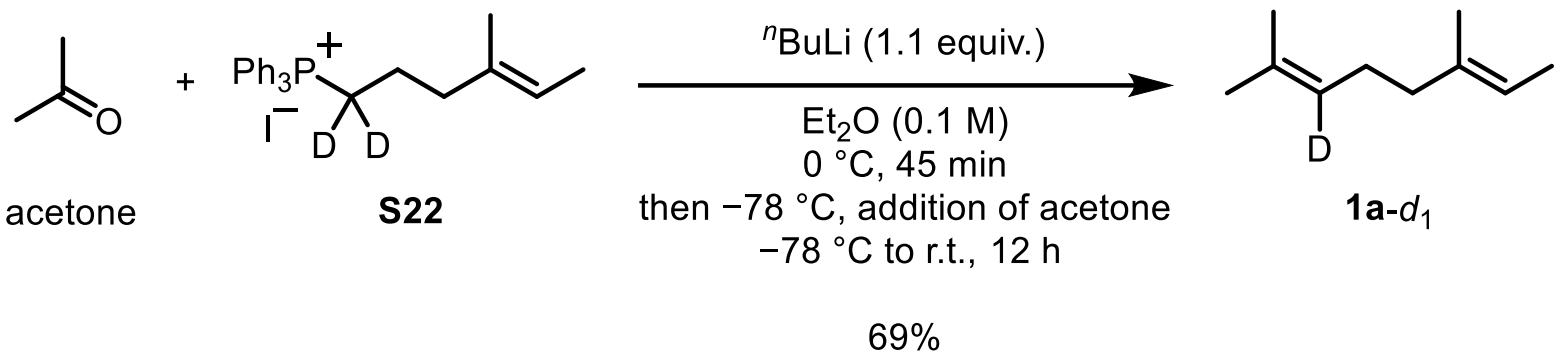

The Wittig reaction was performed by adapting a procedure reported by Dickschat et al. for a related substrate.[18] Wittig salt **S22** (2.21 g, 5.00 mmol, 1.00 eq.) was suspended in dry diethyl ether (50 mL, 0.1 M) and the resulting colorless suspension was cooled to 0 °C. Then, a solution of *n*-butyllithium (2.5 M in *n*-hexane, 2.20 mL, 5.50 mmol, 1.10 eq.) was added dropwise. The resulting orange suspension was stirred at 0 °C for 45 min. The bright red solution of the ylide was cooled to −78 °C and dry acetone (0.44 mL, 348 mg, 6.00 mmol, 1.20 eq.) was added dropwise (color change from a bright red solution → orange → yellow → beige suspension upon addition of acetone). The cooling bath was removed and the beige suspension was stirred at room temperature overnight. After 12 h, the beige suspension was diluted with *n*-pentane (20 mL) and filtered over a small pad of silica gel. The filtrate was carefully concentrated under reduced pressure. Purification by flash column chromatography on silica gel using *n*-pentane (isocratic elution) as eluent afforded 1,5-diene **1a-*d*$_1$** (478 mg, 3.43 mmol, 69% yield) as a colorless oil (evaporation of *n*-pentane at 10 °C and 200 mbar). Purification and removal of *n*-pentane can also be accomplished by Kugelrohr distillation (oven temperature T = 105–110 °C, p = 35 mbar). MS and NMR spectroscopic analysis indicated >98% D-incorporation at the desired position.

Physical state: colorless oil.
TLC (SiO_2, *n*-pentane): $\boldsymbol{R_F}$ = 0.72 ($KMnO_4$ or CAM stain).

^{1}H NMR (600 MHz, $CDCl_3$): δ (ppm) = 5.21 (qh, J = 6.7, 1.3 Hz, 1H), 2.09 – 2.03 (m, 2H), 2.00 – 1.96 (m, 2H), 1.68 (m, 3H), 1.62 – 1.59 (m, 6H), 1.57 (dh, J = 6.7, 1.1 Hz, 3H).

^{13}C{^{1}H} NMR (151 MHz, $CDCl_3$): δ (ppm) = 135.95, 131.37, 124.18 (t, $^1J_{C–D}$ = 23.0 Hz), 118.37, 39.86, 26.78, 25.79 (t, $^3J_{C–D}$ = 0.9 Hz), 17.79 (t, $^3J_{C–D}$ = 1.2 Hz), 15.81, 13.50.

^{2}H NMR (92 MHz, $CDCl_3$): δ (ppm) = 5.15 (s, 1D).

HRMS *m*/*z* (GC-EI): calculated for $C_{10}H_{17}D^+$ $[M]^+$: 139.14658, found: 139.14657.

(*E*)-6-methyl-2-(methyl-*d*$_3$)octa-2,6-diene-1,1,1,3-*d*$_4$ (1a-*d*$_7$)

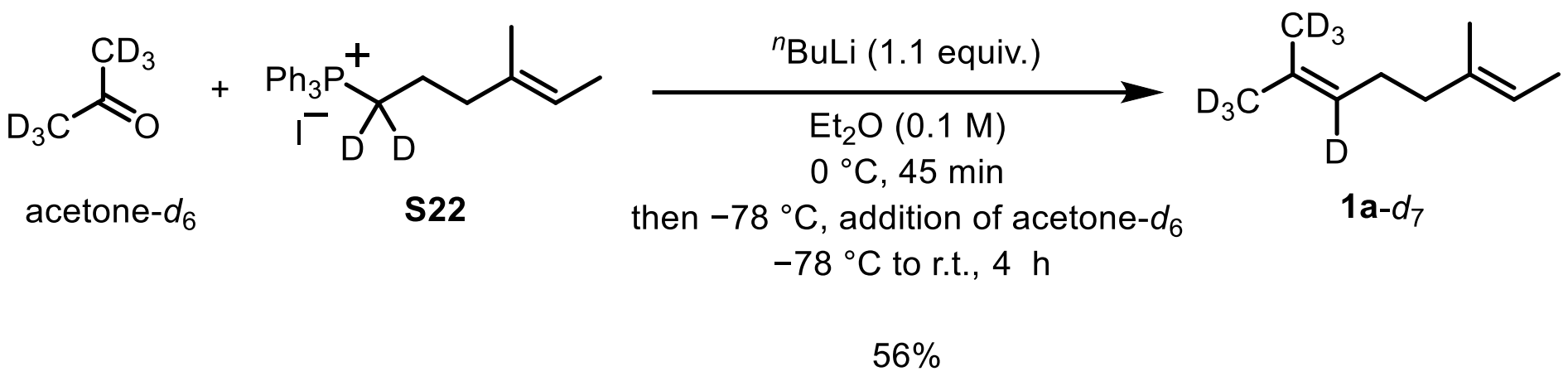

The Wittig reaction was performed by adapting a procedure reported by Dickschat et al. for a related substrate.[14] Wittig salt **S22** (964 g, 2.18 mmol, 1.0 eq.) was suspended in dry diethyl ether (22 mL, 0.1 M) and the resulting colorless suspension was cooled to 0 °C. Then, a solution of *n*-butyllithium (2.5 M in *n*-hexane, 0.96 mL, 2.40 mmol, 1.10 eq.) was added dropwise. The resulting orange suspension was stirred at 0 °C for 45 min. The bright red solution of the ylide was cooled to −78 °C and dry acetone-*d*$_6$ (0.20 mL, 174 mg, 2.72 mmol, 1.25 eq.) was added dropwise (color change from a bright red solution → orange → yellow → beige suspension upon addition of acetone). The cooling bath was removed and the beige suspension was stirred at room temperature overnight. After 4 h, the beige suspension was diluted with *n*-pentane (20 mL) and filtered over a small pad of silica gel. The filtrate was carefully concentrated under reduced pressure. Purification by flash column chromatography on silica gel using *n*-pentane (isocratic elution) as eluent afforded 1,5-diene **1a-*d*$_7$** (177 mg, 1.22 mmol, 56% yield) as a colorless oil (evaporation of *n*-pentane at 10 °C and 200 mbar). Purification and removal of *n*-pentane can also be accomplished by Kugelrohr distillation (oven temperature T = 110 °C, p = 35 mbar). MS and NMR spectroscopic analysis indicated >98% D-incorporation at the desired positions.

Physical state: colorless oil.

TLC (SiO_2, *n*-pentane): R_F = 0.72 ($KMnO_4$ or CAM stain).

^{1}H NMR (501 MHz, $CDCl_3$): δ (ppm) = 5.25 – 5.18 (m, 1H), 2.09 – 2.03 (m, 2H), 2.01 – 1.95 (m, 2H), 1.60 (s, 3H), 1.57 (dt, J = 6.7, 1.3 Hz, 4H).

^{13}C{^{1}H} NMR (126 MHz, $CDCl_3$): δ (ppm) = 135.97, 131.19, 124.22 (t, $^1J_{C–D}$ = 23.1 Hz), 118.37, 39.88, 26.77, 24.85 (p, $^1J_{C–D}$ = 19.3 Hz), 16.91 (p, $^1J_{C–D}$ = 18.4 Hz), 15.81, 13.49.

HRMS *m*/*z* (GC-EI): calculated for $C_{10}H_{11}D_7^+$ $[M]^+$: 145.18424, found: 145.18430.

9.4.2 Measurement of the β-DKIE

Competitive β-DKIE of 1a and 1a-d_1:

Sample Preparation A mixture of **1a** (12.5 µL) and **1a**-d_1 (12.5 µL) was dissolved in toluene (25 µL), and CH_2Br_2 (10 µL) was added as an internal standard. The solution was cooled to −50 °C prior to use.

Separately, an oven-dried (80 °C, overnight) GC vial equipped with a magnetic stir bar was charged with IDPi 5d (2 mol%). Dry toluene (0.107 mL) and PFTB–OH (0.275 mmol, 2.0 equiv) were added at −50 °C, followed by the precooled substrate solution. The reaction mixture was maintained at −50 °C, and aliquots were withdrawn after 1, 2, and 3 h. The remaining amounts of **1a** and **1a**-d_1 as well as product formation were quantified by NMR spectroscopy using CH_2Br_2 as an internal standard. Each experiment was performed in triplicate. Data analysis was performed according to previously reported procedures.[19]

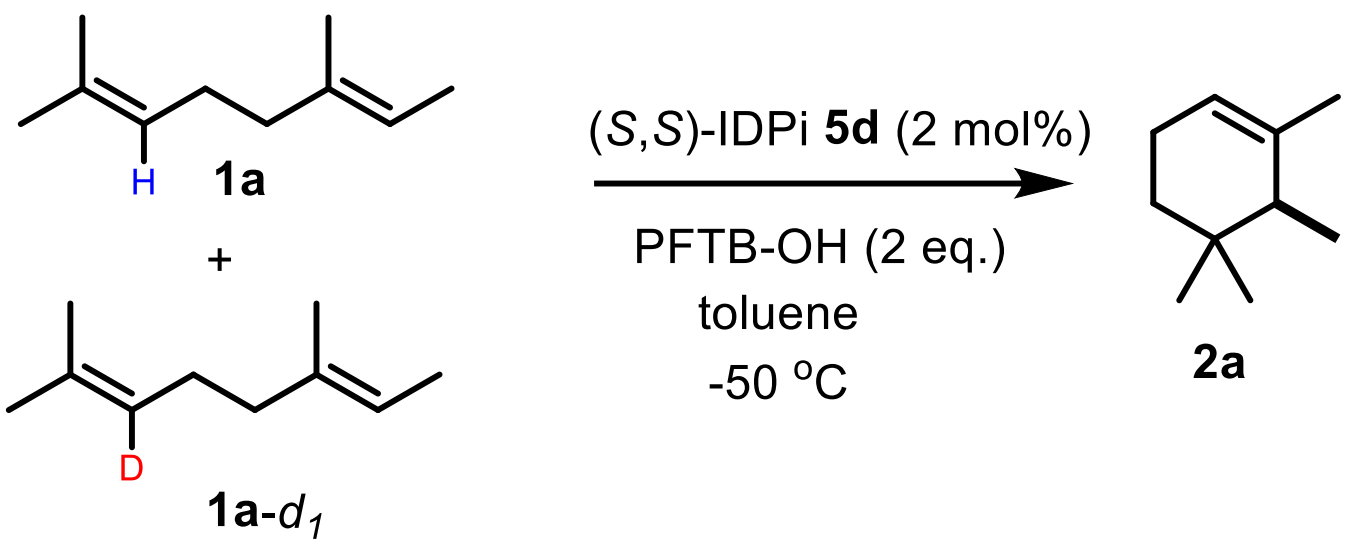

	Initial concentration 1a (mM)	Initial concentration 1a-d_1 (mM)	Reaction concentration 1a (mM)	Reaction concentration 1a-d_1 (mM)	KIE	Time
Repeat 1	76.083	69.933	66.0984	62.9296	1.333197	1 h
	76.083	69.933	62.3635	58.5105	1.115029	2 h
	76.083	69.933	56.5224	52.1886	1.015424	3 h
Repeat 2	76.083	69.933	64.7659	61.8161	1.305347	1 h
	76.083	69.933	60.1103	56.2337	1.080828	2 h
	76.083	69.933	55.4694	51.0356	1.003108	3 h
Repeat 3	76.083	69.933	64.7909	61.3921	1.23341	1 h
	76.083	69.933	60.307	55.867	1.034791	2 h
	76.083	69.933	54.7378	49.9332	0.977494	3 h

Table S8. Competitive β-DKIE of **1a** and **1a**-d_1.

Parallel β-DKIE of 1a and 1a-d_1 in PFTB-OD

Sample Preparation An oven-dried (80 °C, overnight) 3 mm NMR tube was charged with IDPi **5d** (2 mol%). Dry toluene-d_8 (0.107 mL) and PFTB-OD (0.275 mmol, 2 eq.) were added. The initial mixture was precooled to –78 °C in a dry ice/acetone bath. After addition of the substrate **1a** or **1a**-d_1 (0.137 mmol, 1.0 eq.), the NMR tube was quickly turned upside down, vortexed, and transferred to the precooled NMR probe at 223 K (–50 °C). After quick shimming, single-scan ^{1}H and ^{31}P NMR spectra were acquired every 5 min until completion of the reaction.

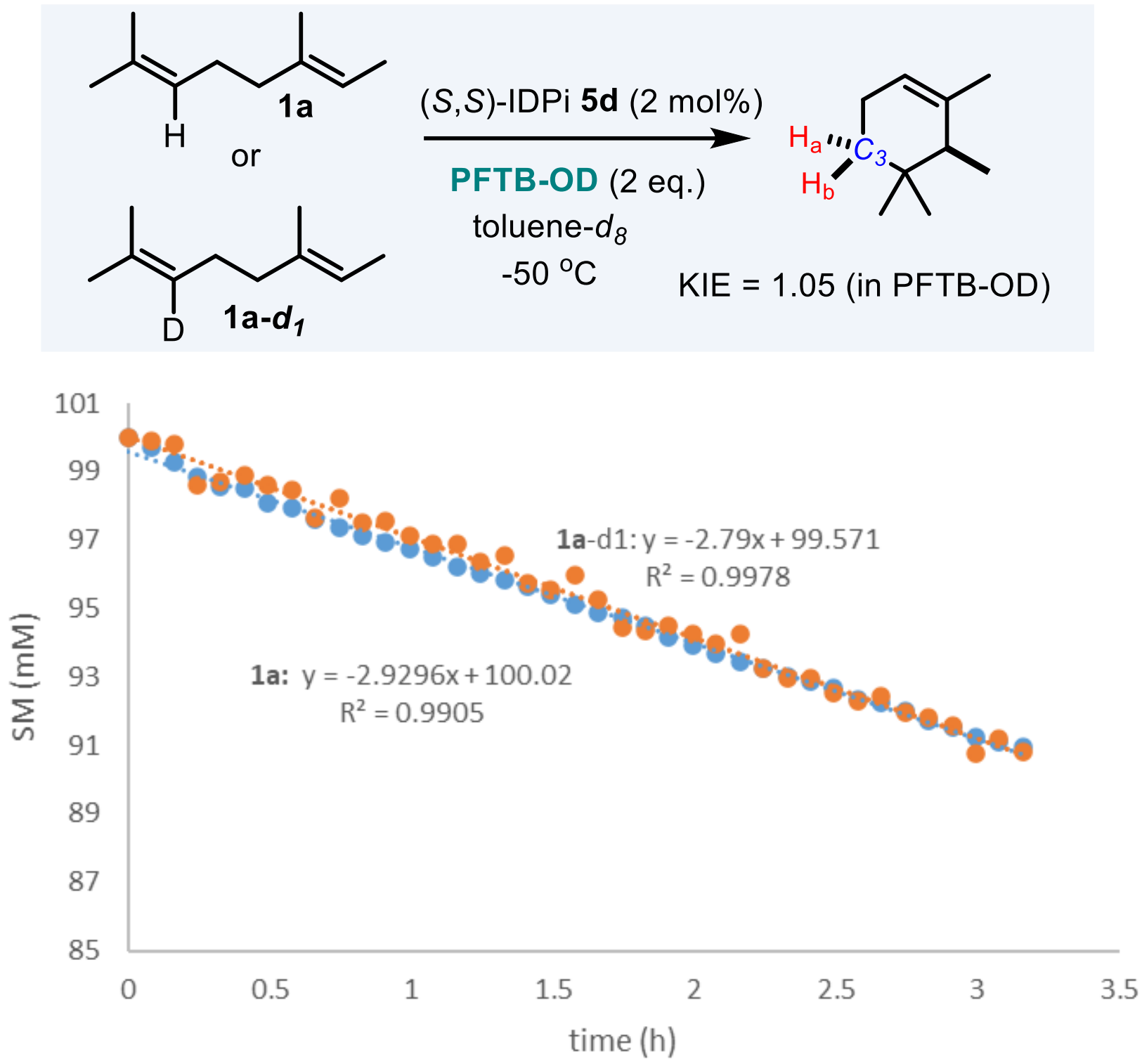

Figure S18. Slope for the initial 10% consumption of **1a** (orange) and **1a-*d*$_1$** (blue) in PFTB-OH.

Parallel β-DKIE of 1a and 1a-*d*$_7$ in PFTB-OD

Sample Preparation An oven-dried (80 °C, overnight) 3 mm NMR tube was charged with IDPi **5d** (2 mol%). Dry toluene-*d*$_8$ (0.107 mL) and PFTB-OD (0.275 mmol, 2 eq.) were added. The initial mixture was precooled to –78 °C in a dry ice/acetone bath. After addition of the substrate **1a** or **1a**-*d*$_7$ (0.137 mmol, 1.0 eq.), the NMR tube was quickly turned upside down, vortexed, and transferred to the precooled NMR probe at 223 K (–50 °C). After quick shimming, single-scan ^{1}H and ^{31}P NMR spectra were acquired every 5 min until completion of the reaction.

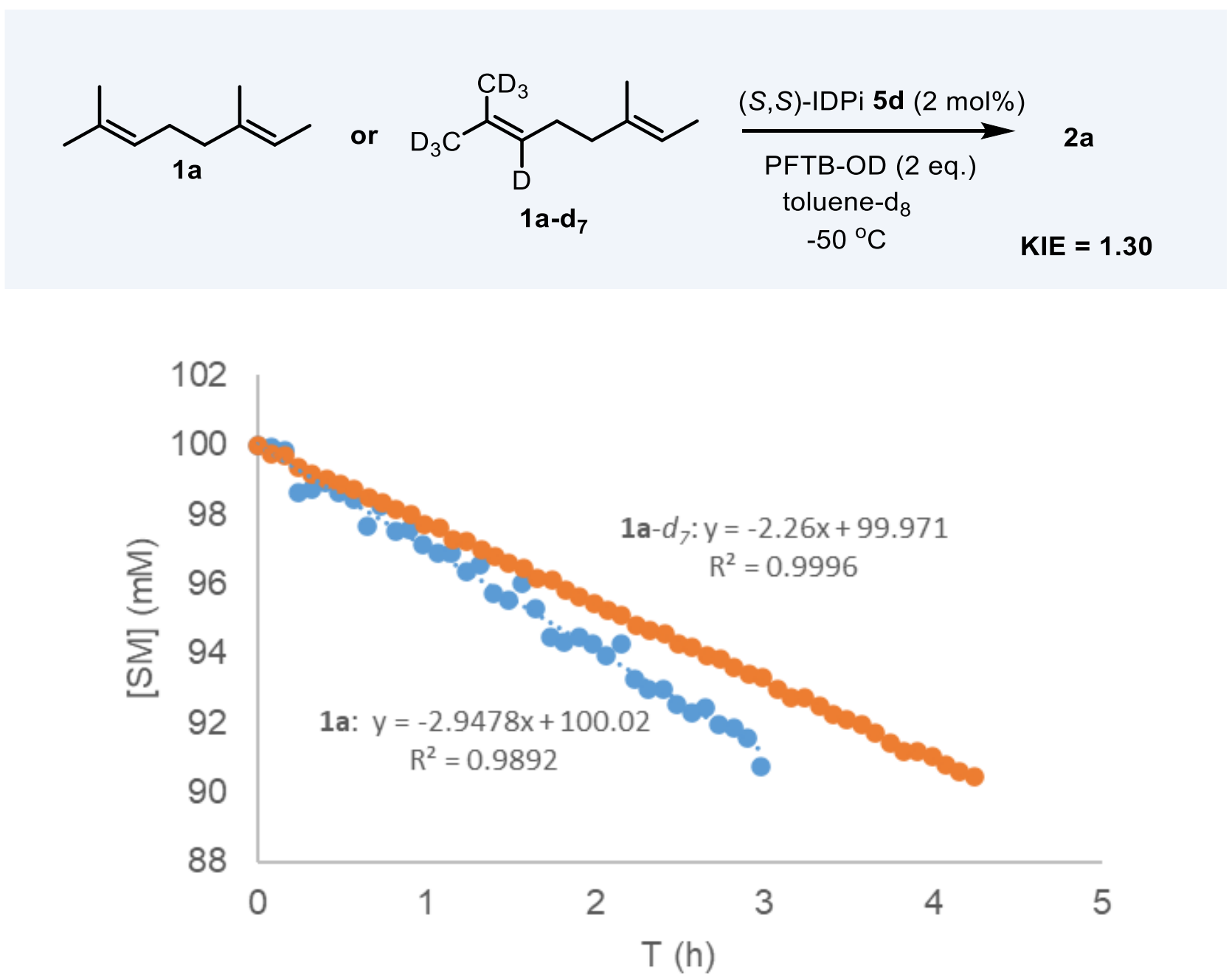

Figure S19. Slope for the initial 10% consumption of **1a** (orange) and **1a-*d*$_7$** (blue) in PFTB-OD.

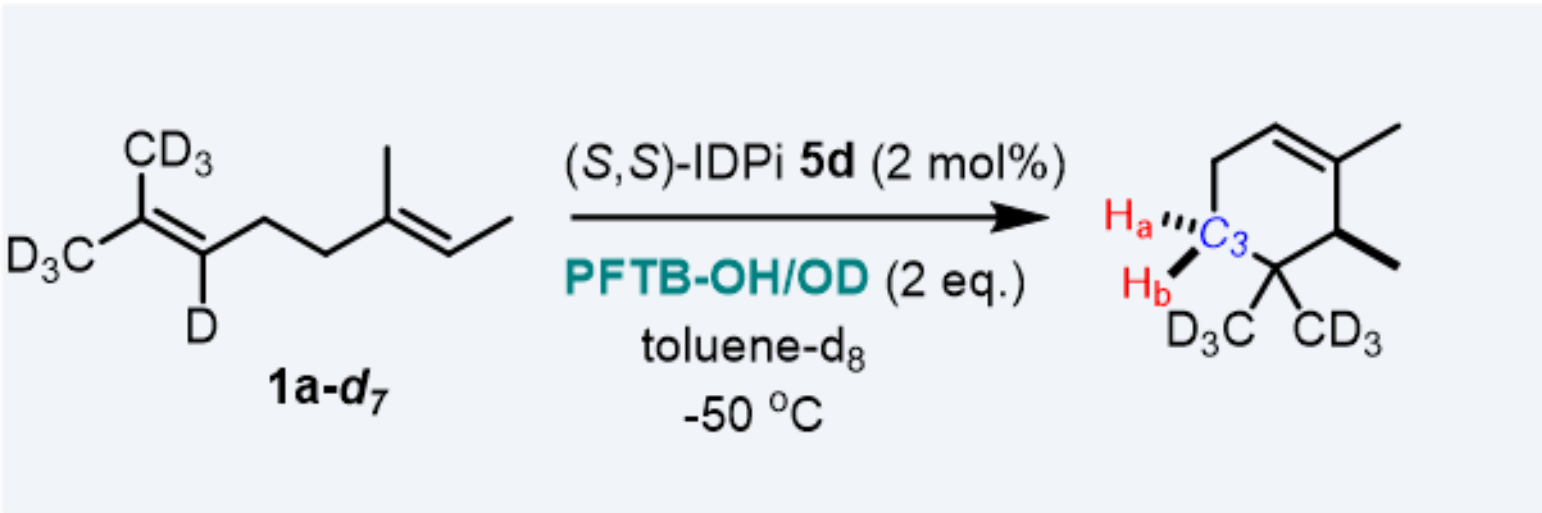

PFTB-OH	Isotope distribution	PFTB-OD	Isotope distribution
$C_3D_aH_b$	94.0%	$C_3D_aH_b$	73.6%
$C_3D_aD_b$	0	$C_3D_aD_b$	23.3%
$C_3H_aH_b$	2.6%	$C_3H_aH_b$	1.5%
$C_3H_aD_b$	3.4%	$C_3H_aD_b$	2%

Figure S20. Products Isotope distribution on C_3 position when using **1a**-*d*$_7$ as substrate.

	KIE experimental	*KIE (cal.) uncorrected*	*KIE (cal.) Wigner*	*KIE (cal.) Inverted parabola*
**1a**-*d*$_1$	1.05	0.95	0.96	1.10
**1a**-*d*$_7$	1.30	1.01	1.02	1.21

Table S9. Experimentally determined and calculated KIE values. KIE values are calculated using PyQuiver program.[20]

Conclusion:

We performed KIE measurements using the C_3-monodeuterated substrate **1a**-*d*$_1$ (Table S8). Interestingly, three independent experiments showed a consistent decrease in the apparent KIE value over the course of the reaction. Although we cannot fully exclude experimental uncertainty, the reproducibility of this trend suggests that it is unlikely to arise purely from random error. More importantly, the initial KIE values were consistently normal ($k_H/k_D > 1$), rather than the inverse secondary KIE expected for a simple sp^2 to sp^3 rehybridization event at C_3.

To further investigate this unexpected result, we compared the experimental values with calculated KIEs (DFT details see Chapter 13, Table S9). While calculations without tunneling corrections predict the expected inverse KIE, inclusion of a Bell inverted-parabola tunneling correction qualitatively reproduces the experimentally observed sign reversal. Such tunneling corrections have been described in the literature to improve agreement between calculated and experimental KIEs.[21] Furthermore, it is noteworthy that the experimentally observed KIE for the **1a**-*d*$_7$ (1.30) is in reasonably good agreement with the value calculated when tunneling effects are included (1.21). Although this correspondence does not constitute definitive proof and alternative mechanistic scenarios cannot be excluded, it suggests that barrier width and tunneling effects may contribute to the observed isotope effect under our reaction conditions.

In addition, reversible protonation/deprotonation events may further complicate interpretation of the experimental KIEs. Protonation from non-productive conformations could generate carbocationic species that revert to starting material through deprotonation, thereby allowing isotopic scrambling between IDPi–H and IDPi–D. Consistent with

this possibility, isotopic analysis of products derived from **1a**-d_7 consistently revealed a small amount of proton incorporation at the C_3Ha position, where deuterium retention would otherwise be expected (Figure S20).

When the experiment was performed in PFTB–OD, where H/D scrambling is expected to be reduced, 1a-d1 exhibited a KIE of 1.05 (Figure S18), substantially smaller than the value of 1.30 observed for **1a**-d_7 (Figure S19). On this basis, an approximate KIE of 1.24 can be estimated for the corresponding d_6 substrate (**1a**-d_6). Although this estimate should be regarded as qualitative, its magnitude is consistent with a significant hyperconjugative secondary isotope effect and therefore suggests appreciable positive charge development during the rate-relevant step. Notably, this estimated value is in good agreement with those reported for zeolite-catalyzed cyclizations of related d_6-geranyl acetate derivatives, where concerted cyclization pathways accompanied by substantial charge buildup in the transition state have been proposed.[22]

Accordingly, while the present KIE data do not allow an unambiguous separation of rehybridization and hyperconjugative contributions, they support the involvement of both C_2 and C_3 in the rate-relevant step and are consistent with a transition state featuring significant charge development during cyclization.

10. GC and HPLC traces of the products

10.1. Copies of GC traces

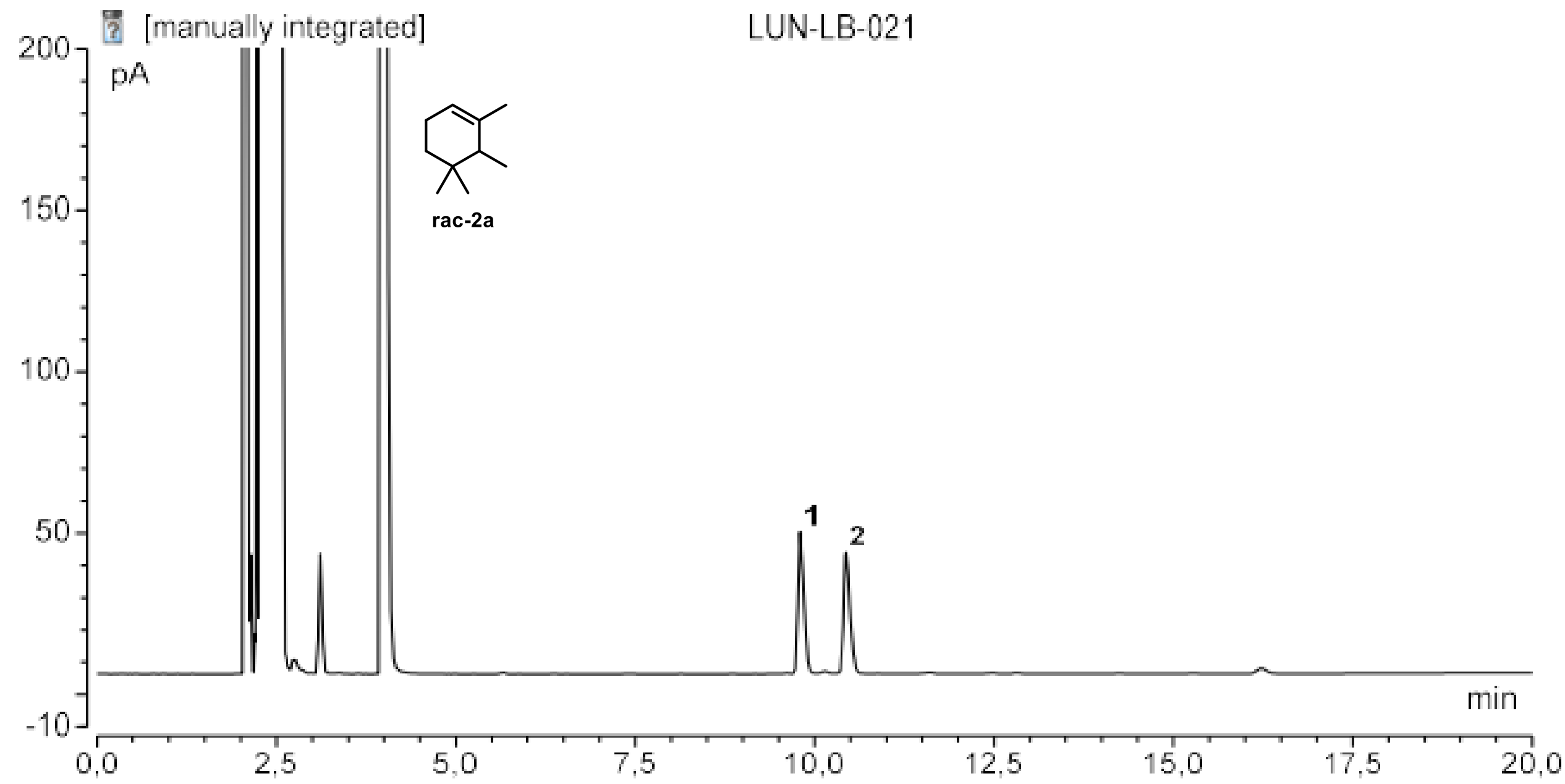

Peak#	Ret. Time (min)	Area%
1	9.80	50.88
2	10.44	49.12
Total		100.00

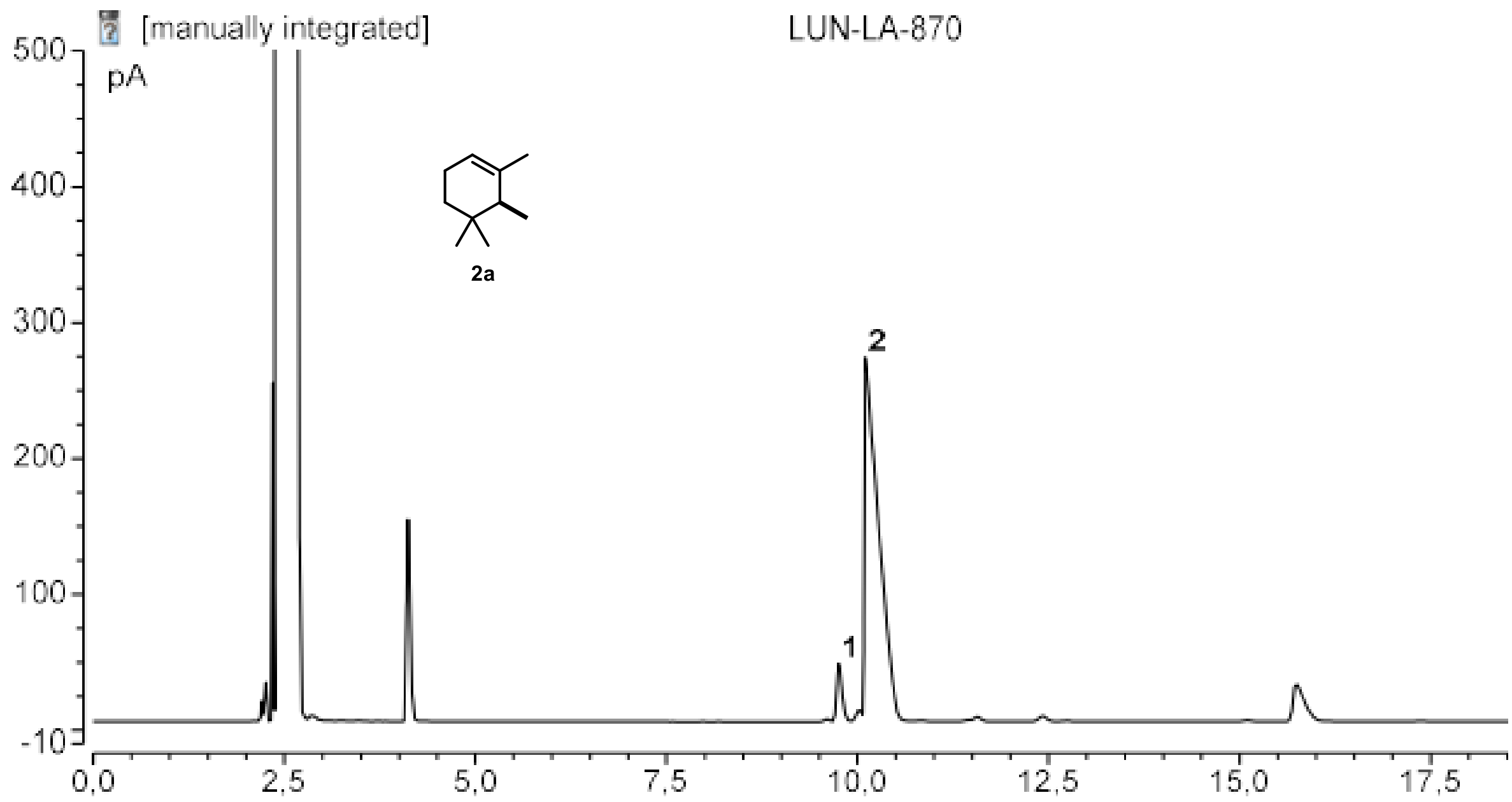

Peak#	Ret. Time (min)	Area%
1	9.75	5.22
2	10.10	94.78
Total		100.00

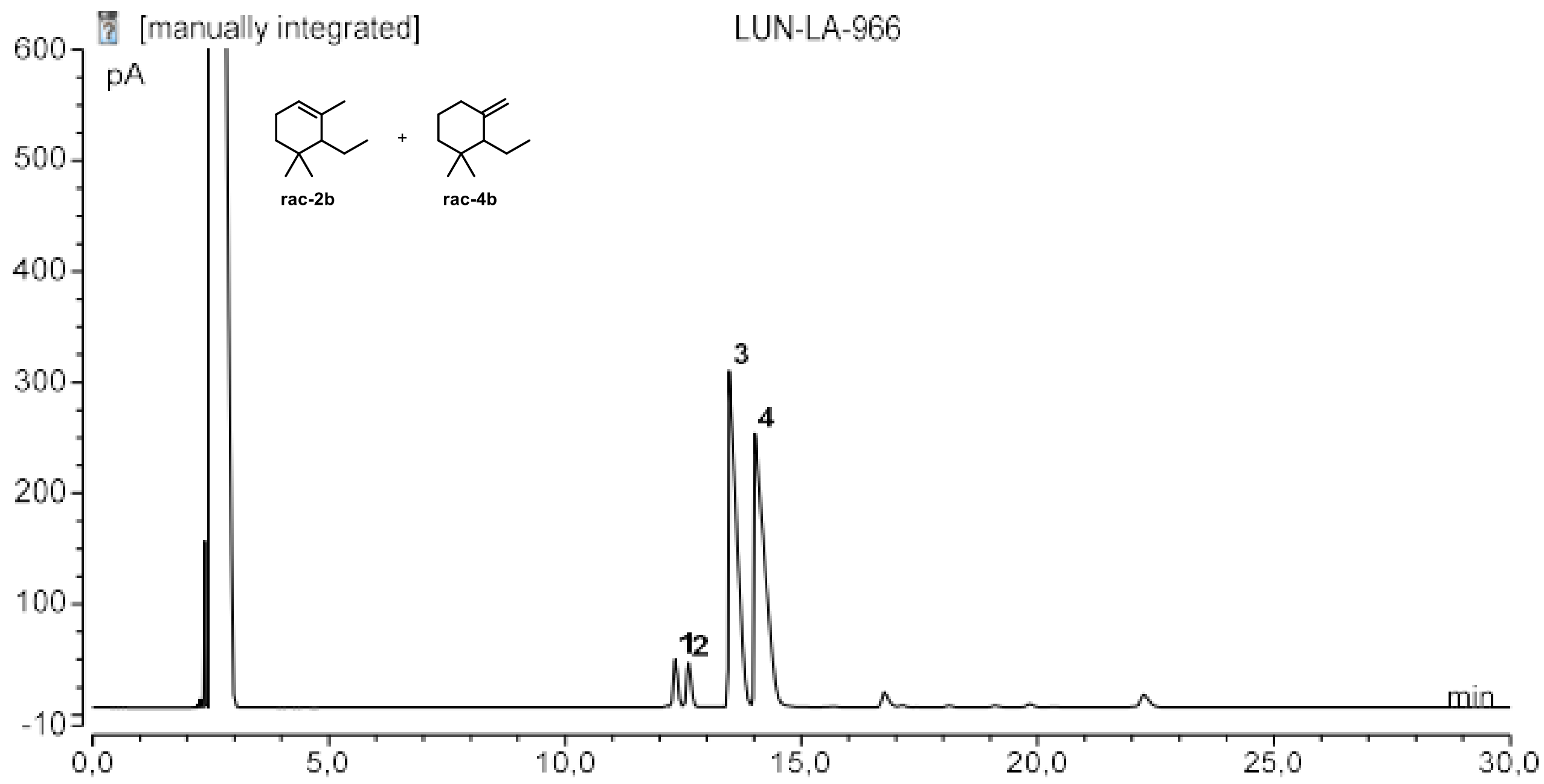

Peak#	Ret. Time (min)	Area%
1	12.34	3.13
2	12.61	2.99
3	13.47	46.26
4	14.02	47.62
Total		100.00

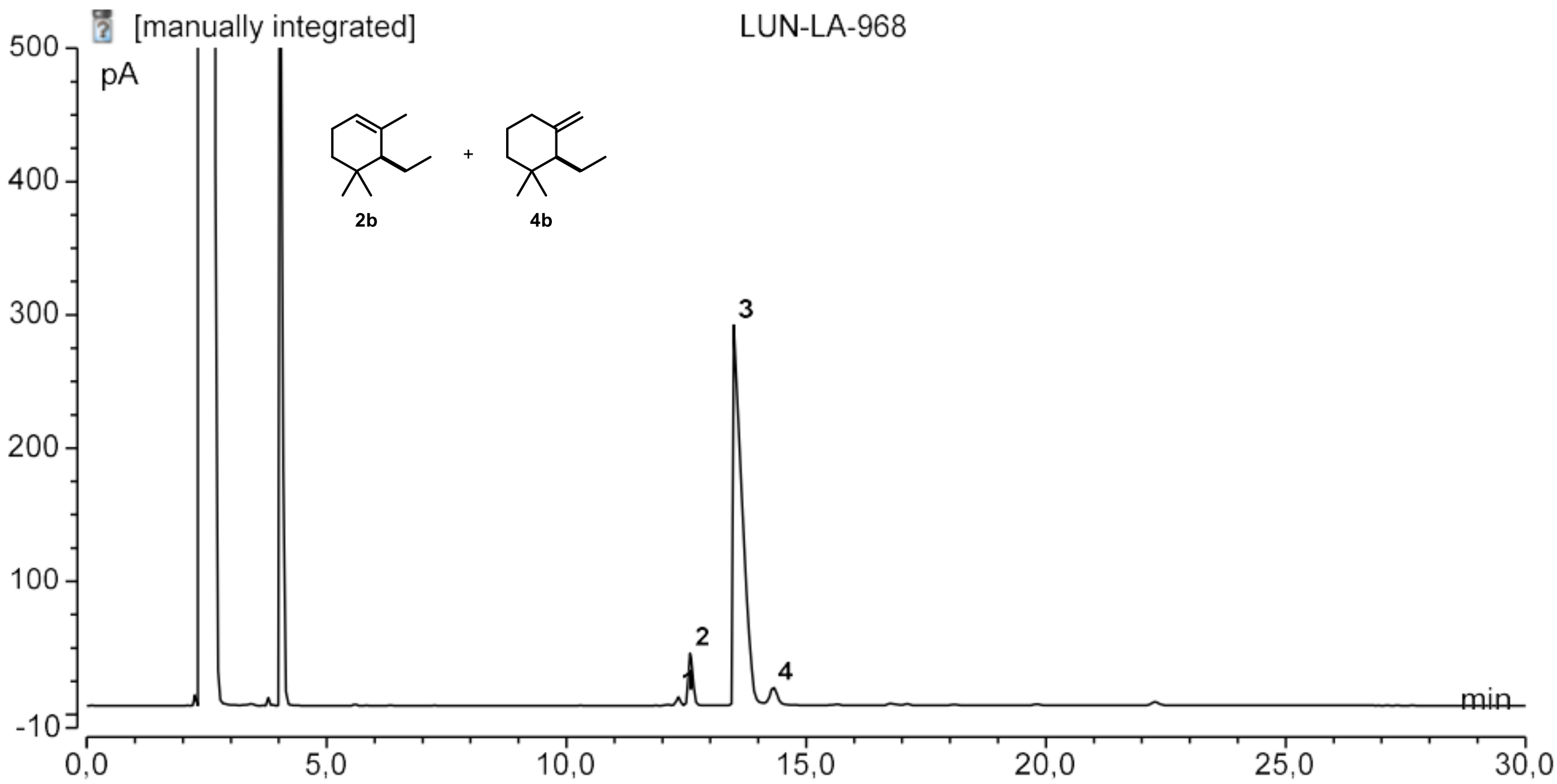

Peak#	Ret. Time (min)	Area%
1	12.33	0.90
2	12.58	6.12
3	13.49	90.50
4	14.32	2.48
Total		100.00

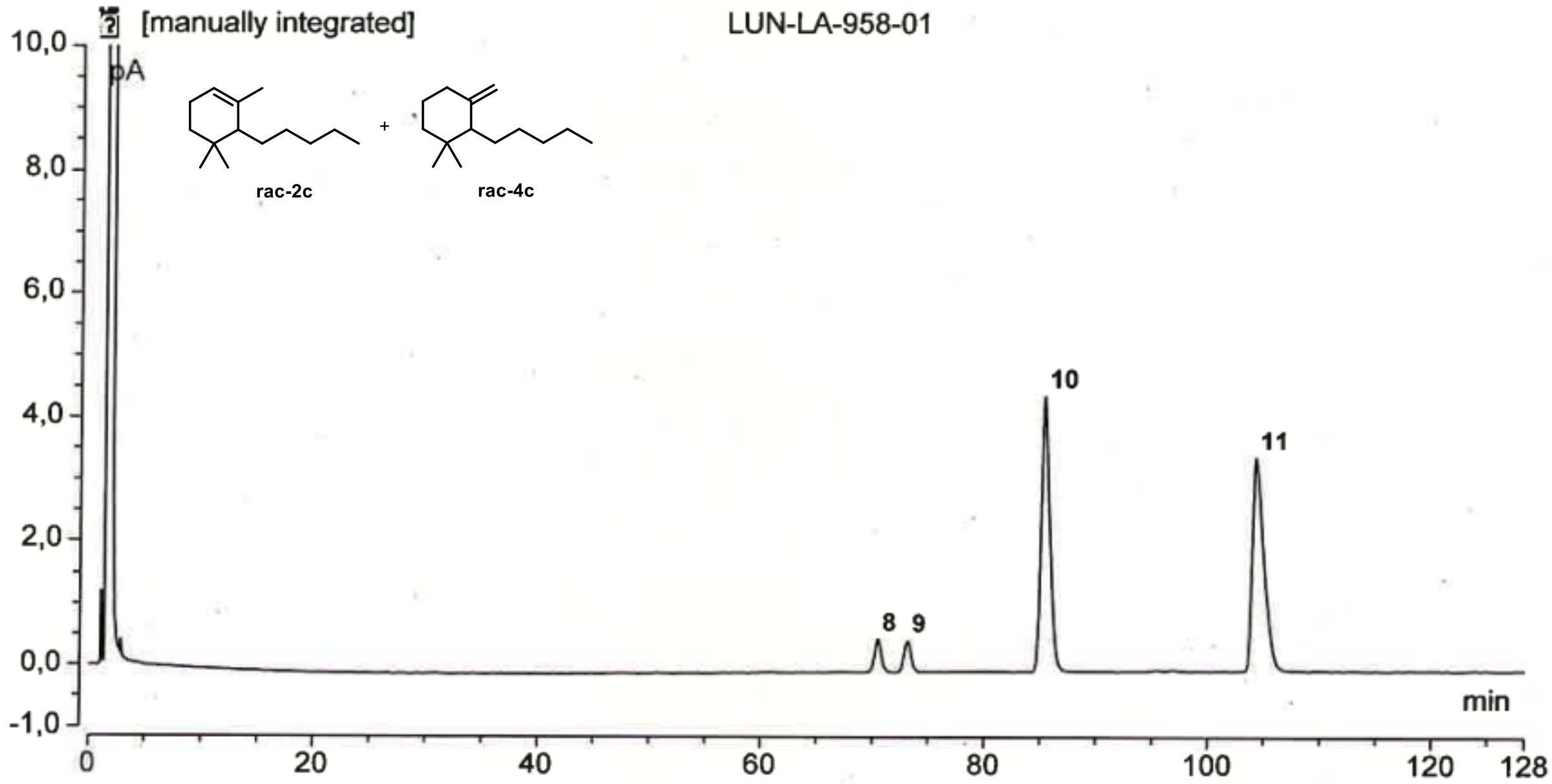

Peak#	Ret. Time (min)	Area%
8	70.43	4.25
9	73.10	4.26
10	85.24	45.22
11	104.16	46.27
Total		100.00

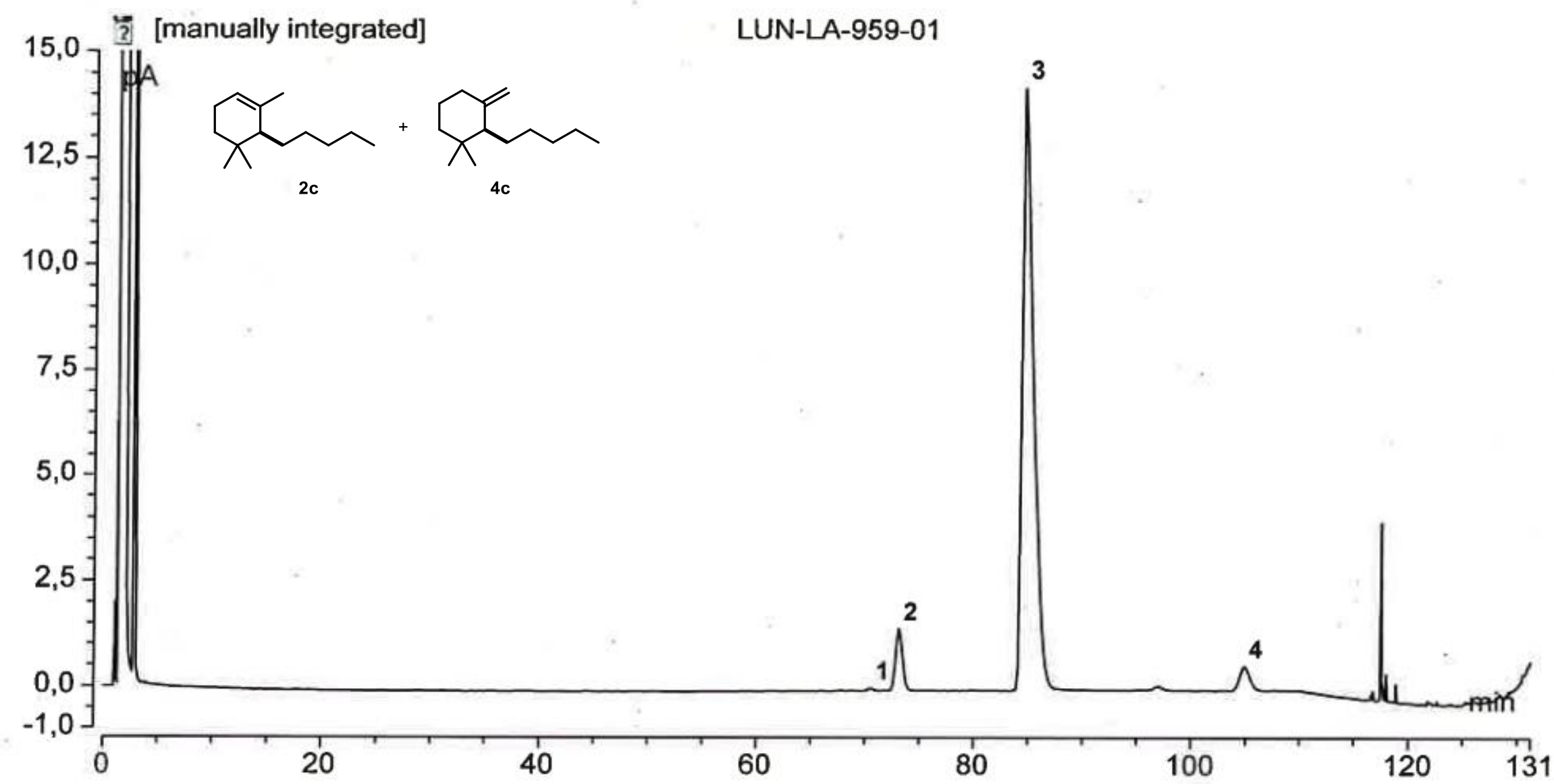

Peak#	Ret. Time (min)	Area%
1	70.58	0.22
2	73.11	5.81
3	84.43	90.69
4	104.89	3.29
Total		100.00

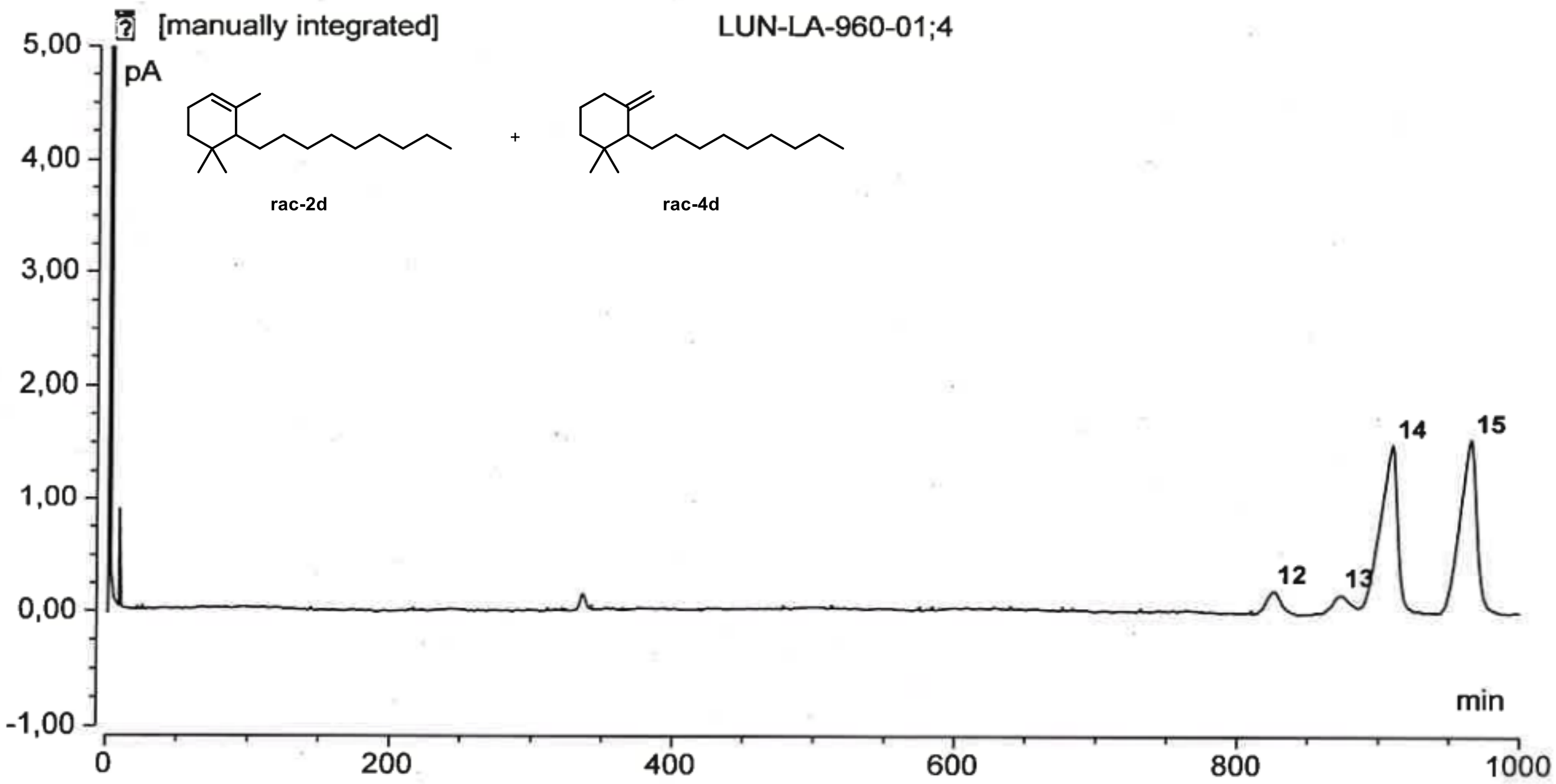

Peak#	Ret. Time (min)	Area%
12	824.60	5.43
13	872.15	5.23
14	908.70	44.77
15	964.41	44.57
Total		100.00

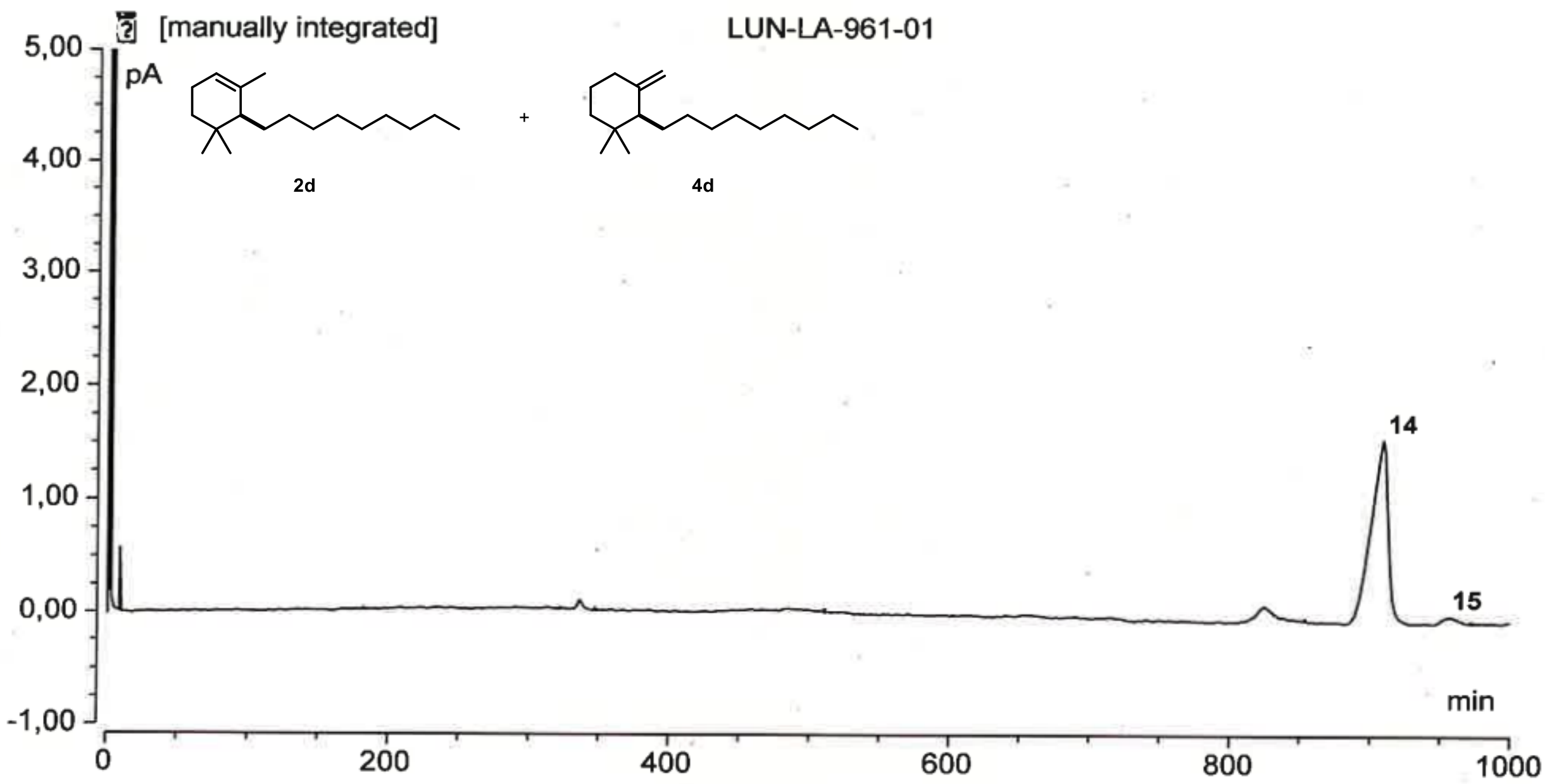

Peak#	Ret. Time (min)	Area%
14	908.31	95.74
15	954.97	4.2
Total		100.00

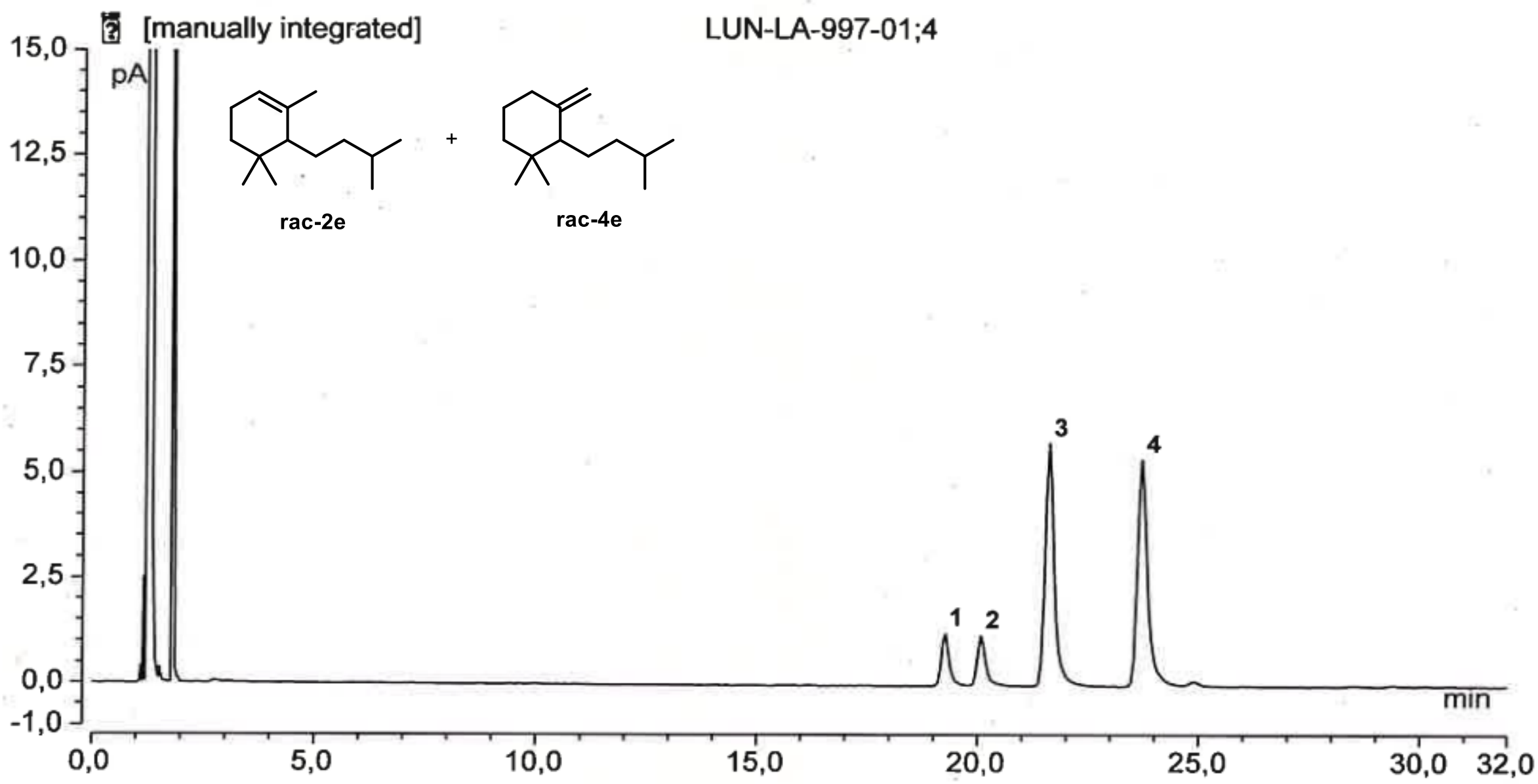

Peak#	Ret. Time (min)	Area%
1	19.26	7.90
2	20.06	7.77
3	21.59	41.07
4	23.68	43.26
Total		100.00

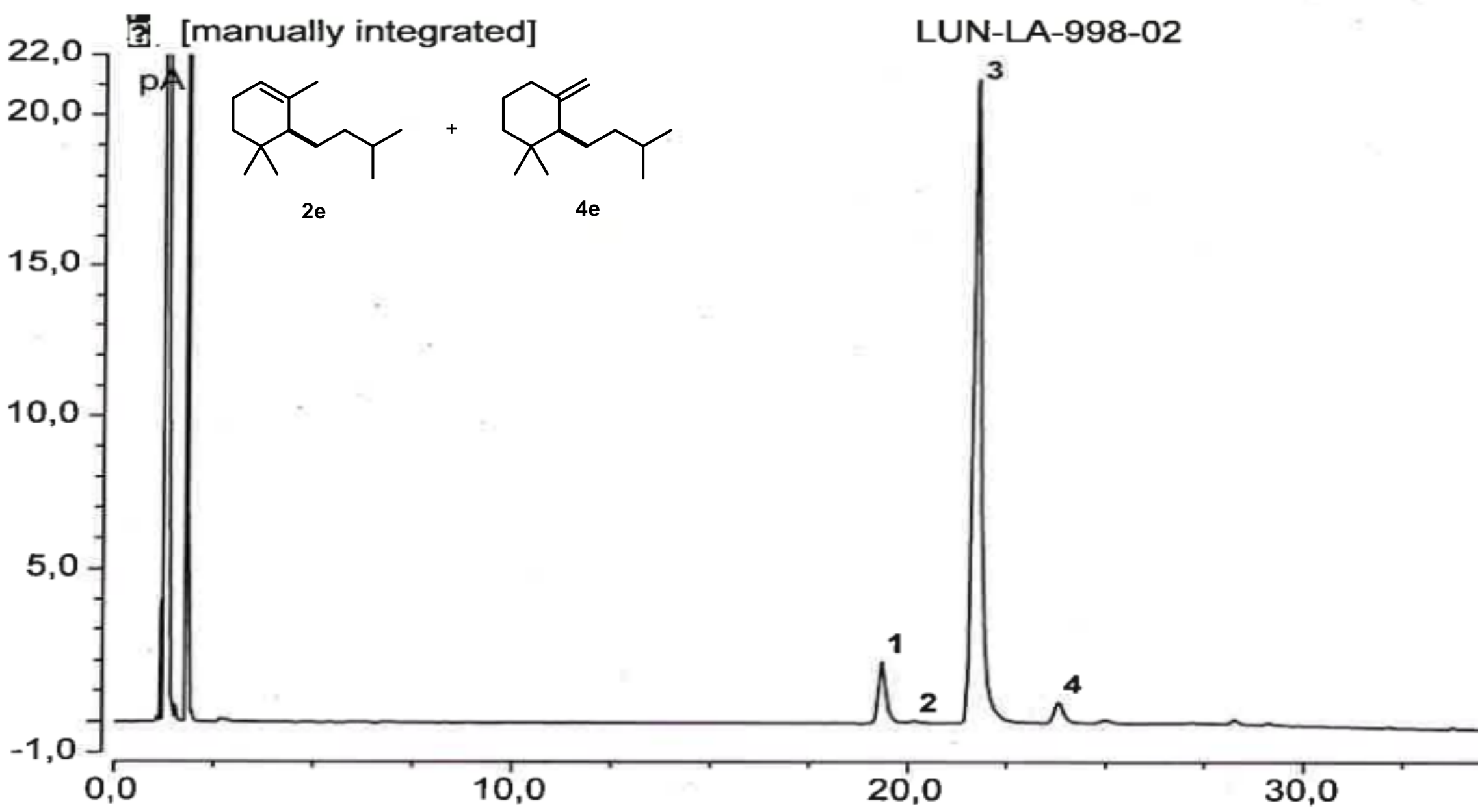

Peak#	Ret. Time (min)	Area%
1	19.32	7.52
2	20.15	0.22
3	21.69	88.99
4	23.80	3.27
Total		100.00

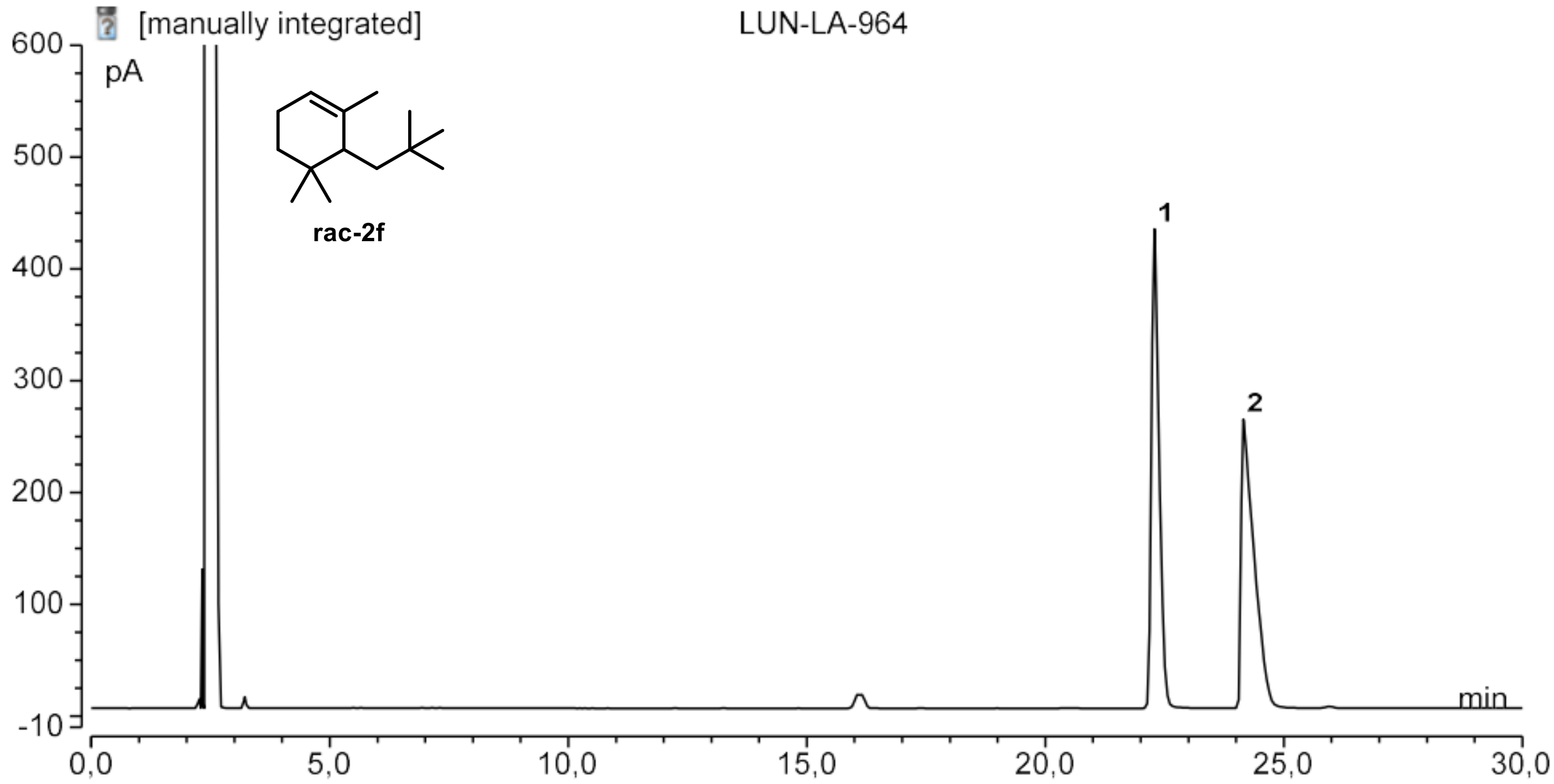

Peak#	Ret. Time (min)	Area%
1	22.29	49.33
2	24.15	50.67
Total		100.00

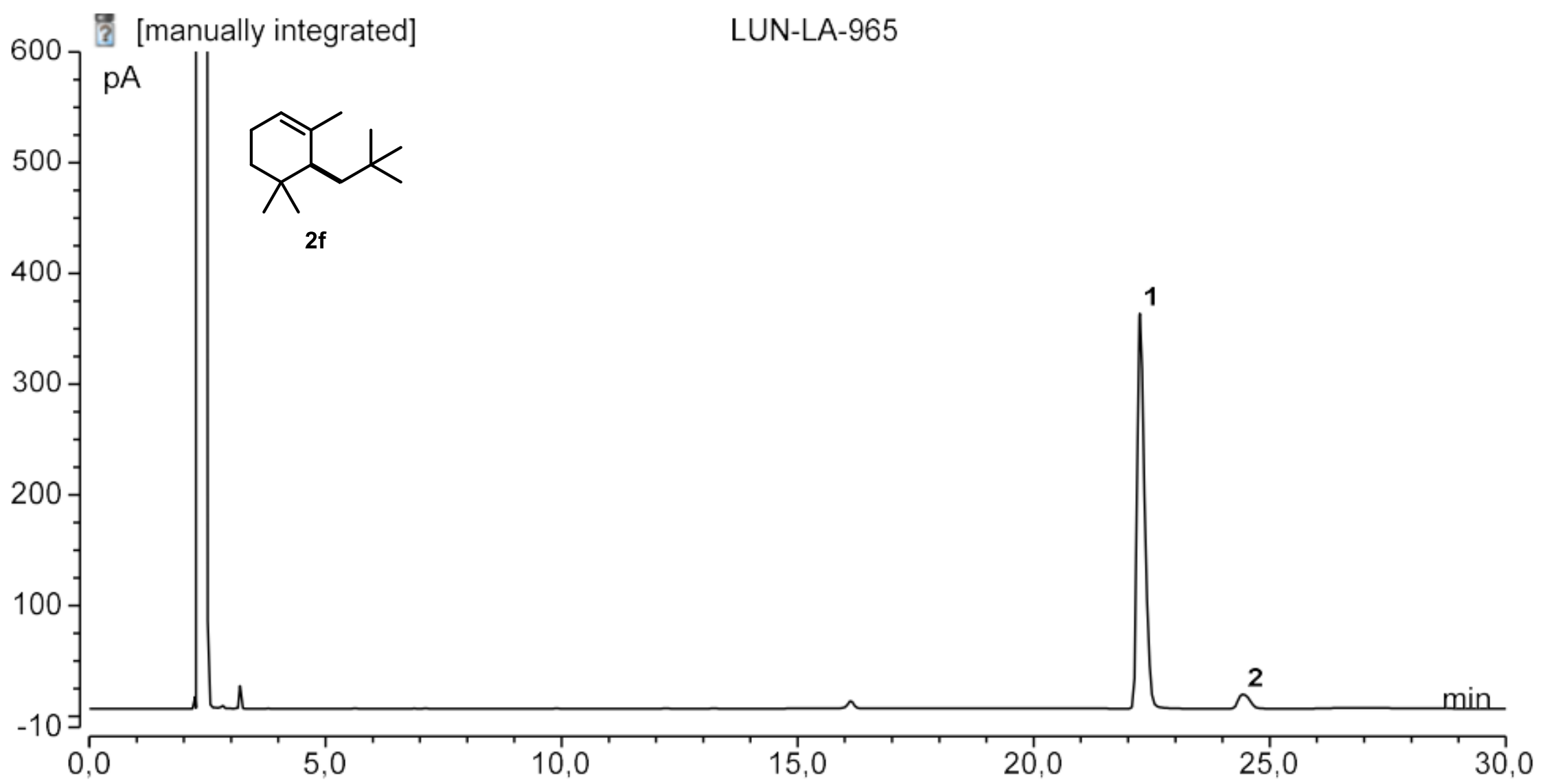

Peak#	Ret. Time (min)	Area%
1	22.25	95.07
2	24.44	4.93
Total		100.00

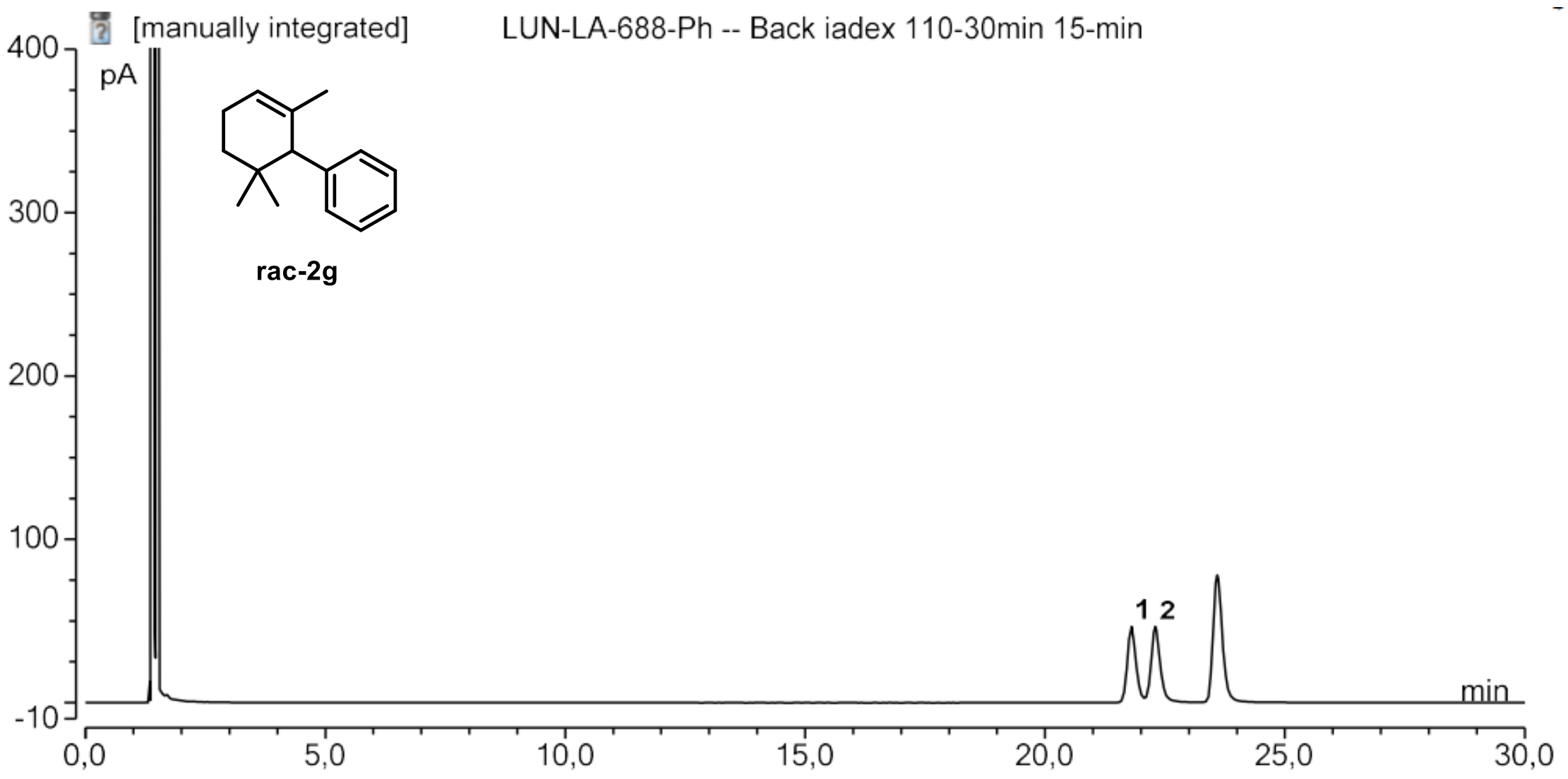

Peak#	Ret. Time (min)	Area%
1	21.81	49.77
2	22.30	50.23
Total		100.00

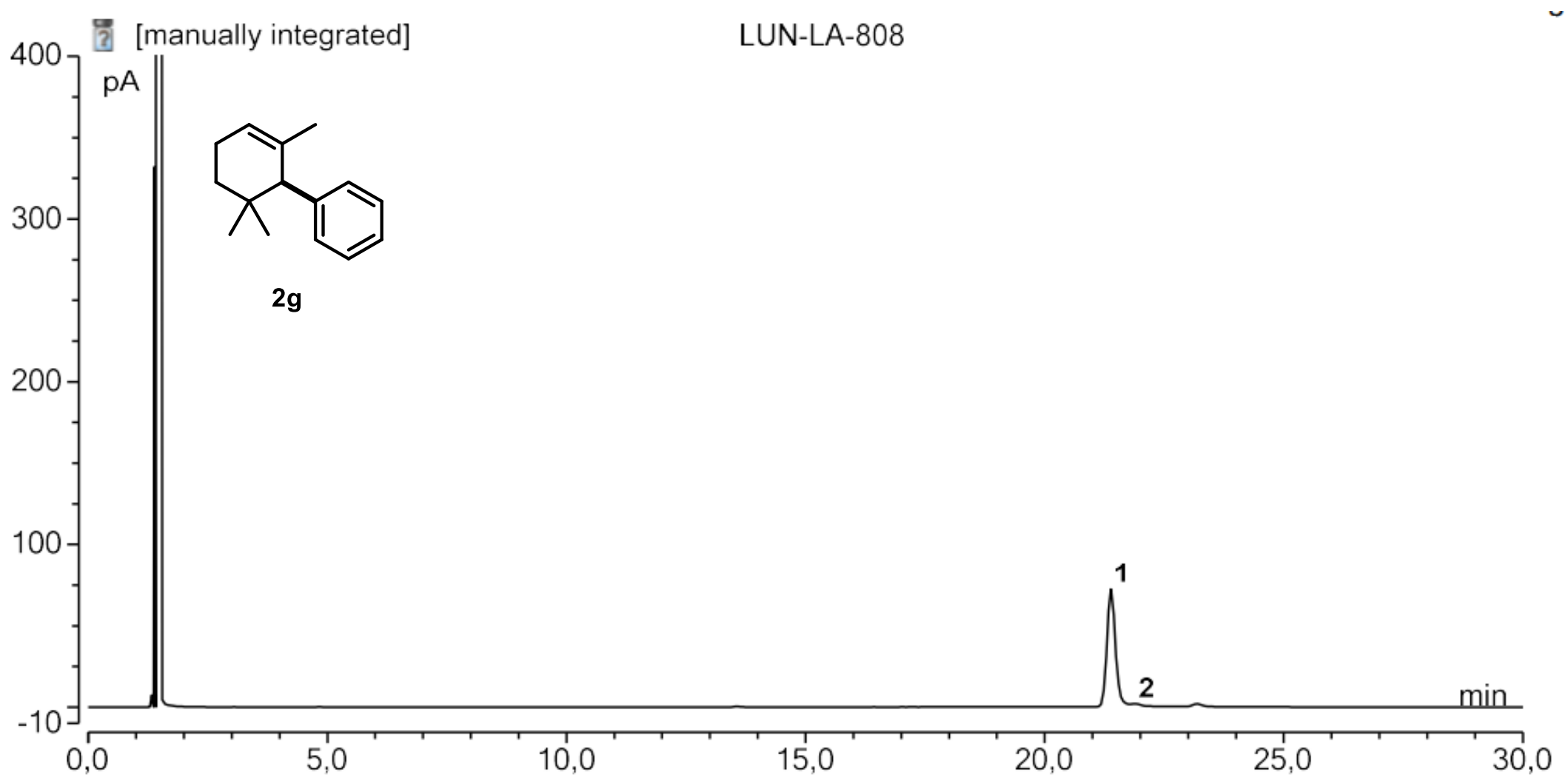

Peak#	Ret. Time (min)	Area%
1	21.38	97.53
2	21.89	2.47
Total		100.00

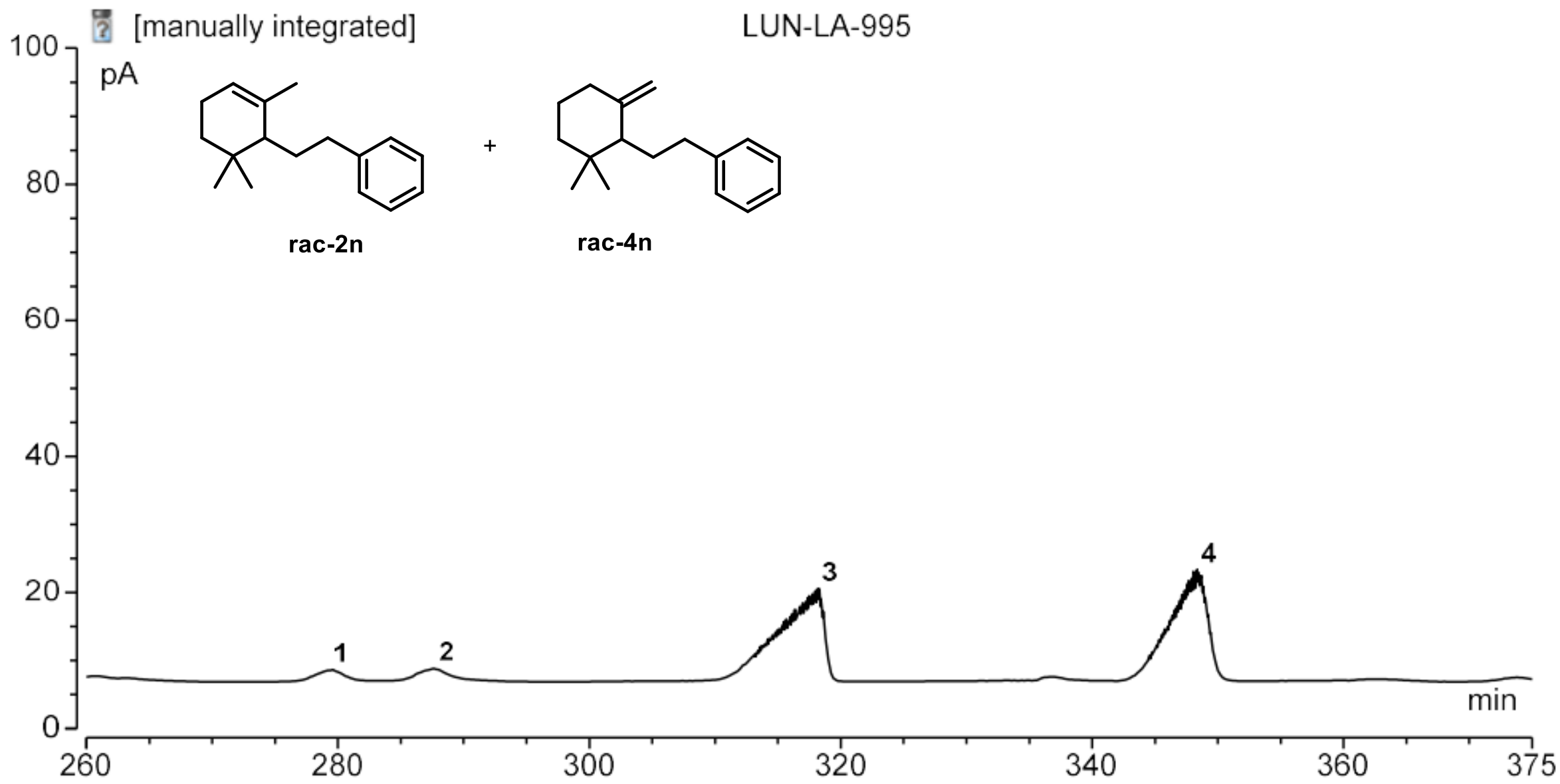

Peak#	Ret. Time (min)	Area%
1	279.35	4.24
2	287.69	5.82
3	318.18	45.57
4	348.34	44.36
Total		100.00

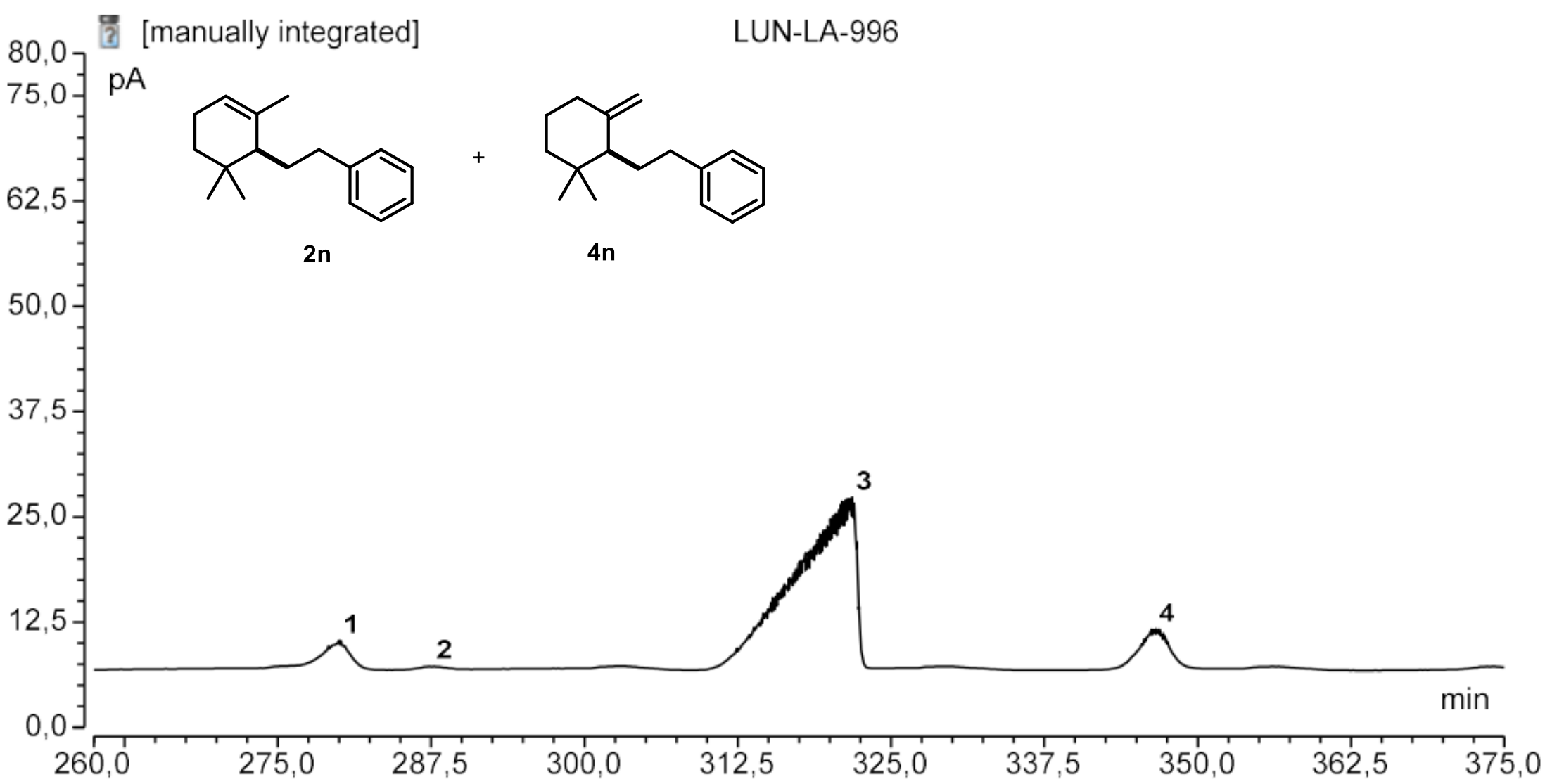

Peak#	Ret. Time (min)	Area%
1	280.03	6.84
2	287.61	0.70
3	321.83	82.78
4	346.59	9.68
Total		100.00

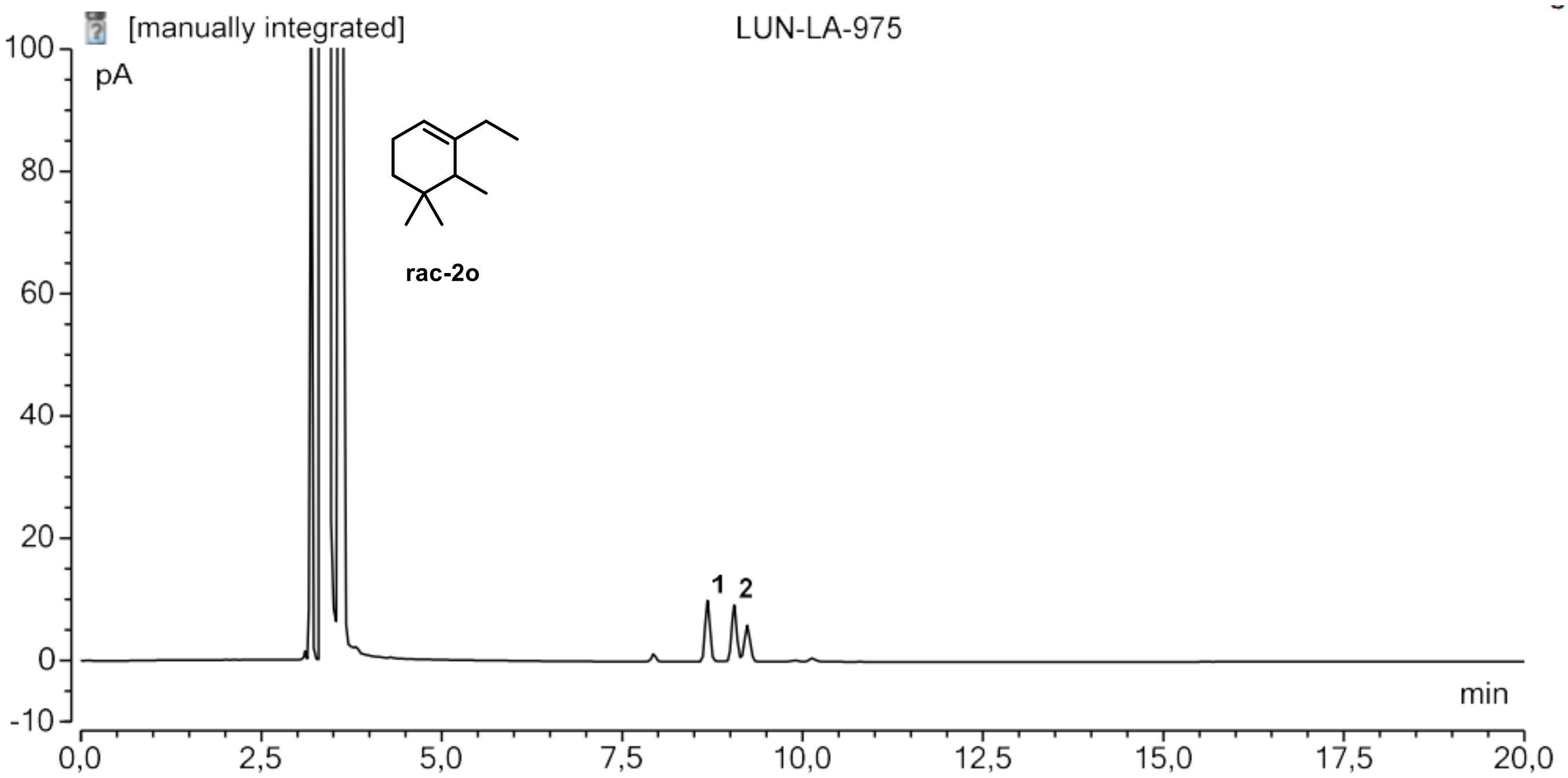

Peak#	Ret. Time (min)	Area%
1	8.68	52.92
2	9.05	47.08
Total		100.00

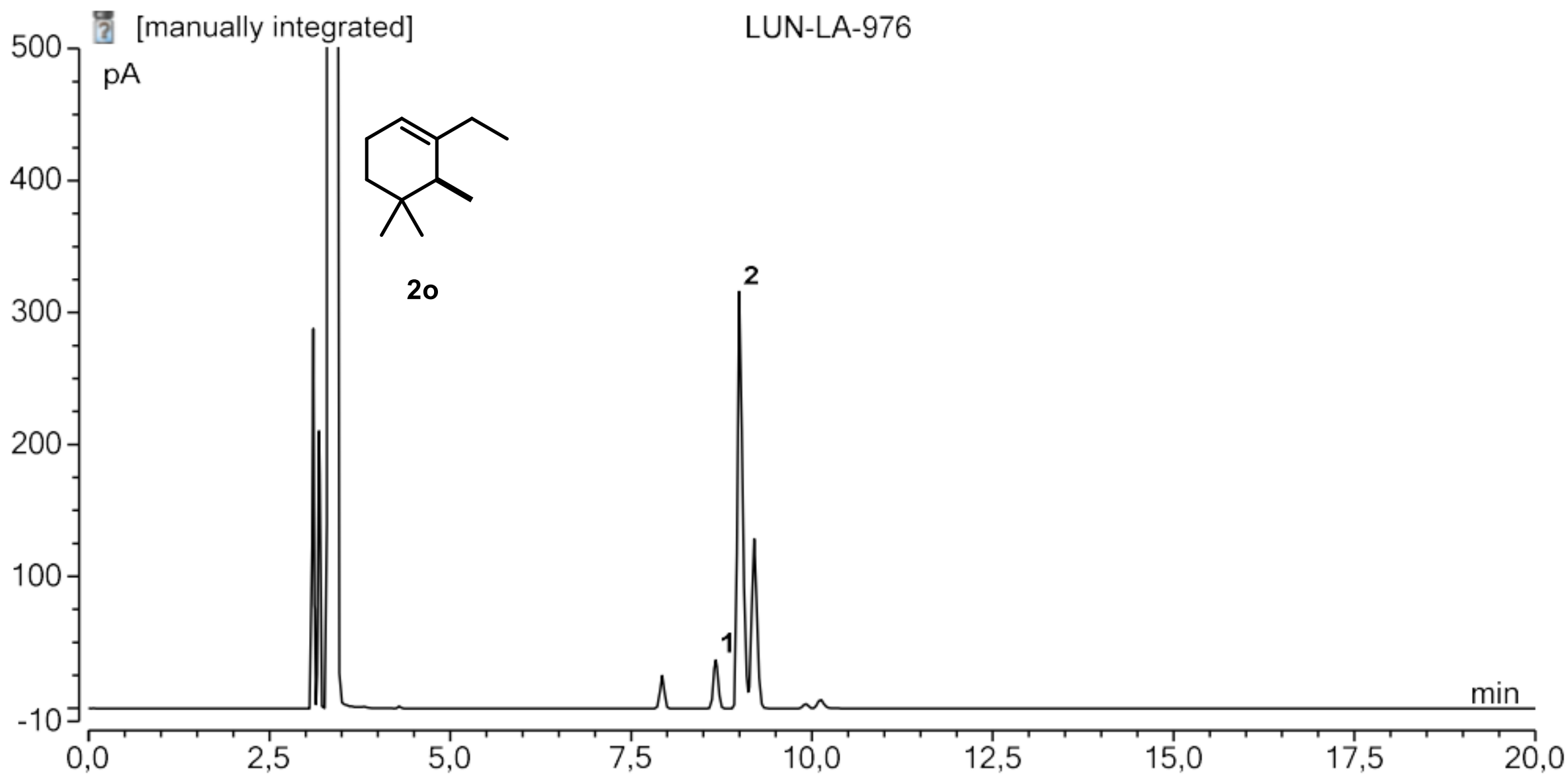

Peak#	Ret. Time (min)	Area%
1	8.67	9.59
2	8.99	90.41
Total		100.00

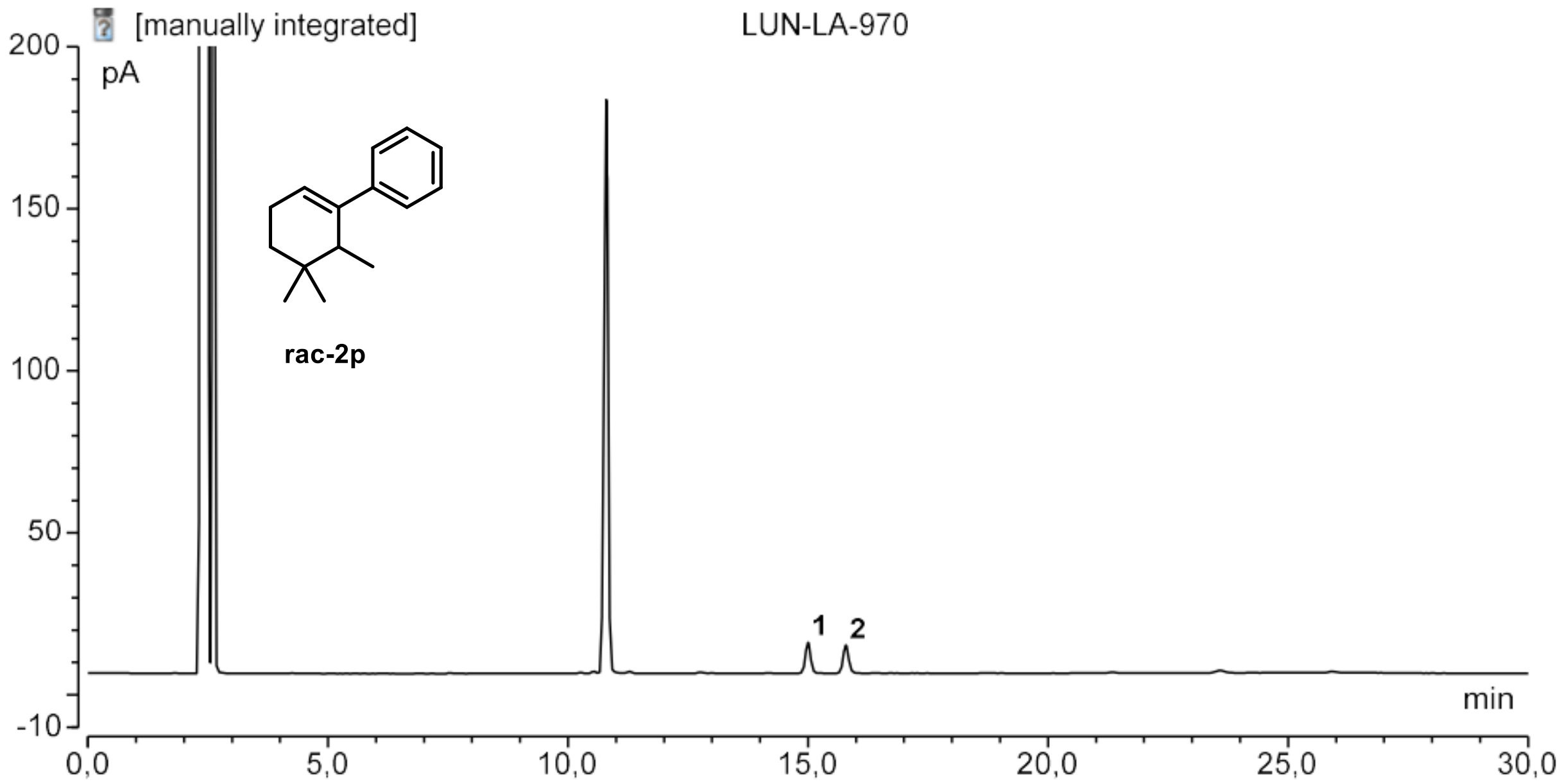

Peak#	Ret. Time (min)	Area%
1	15.00	50.29
2	15.79	49.71
Total		100.00

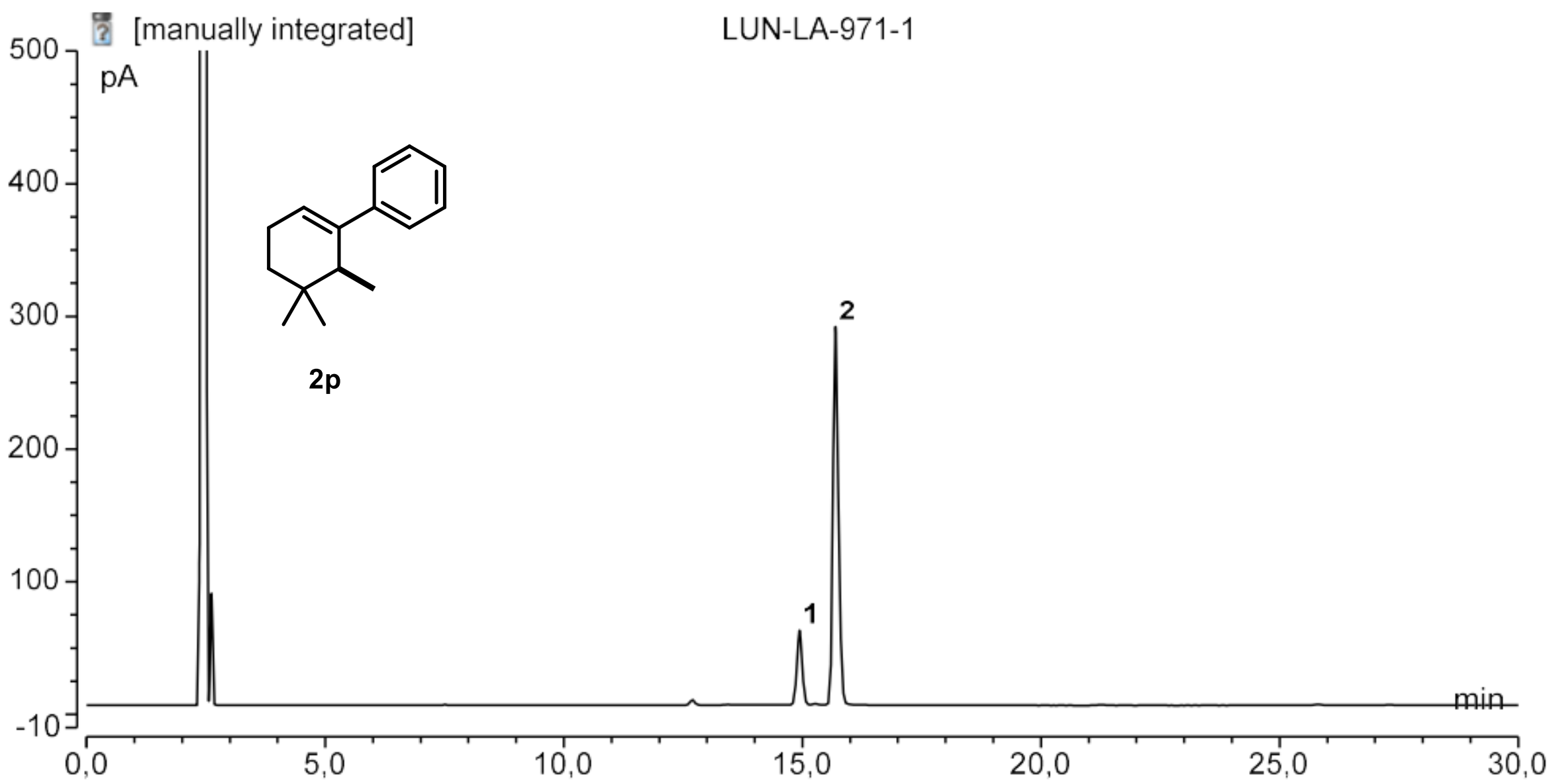

Peak#	Ret. Time (min)	Area%
1	14.94	15.76
2	15.69	84.24
Total		100.00

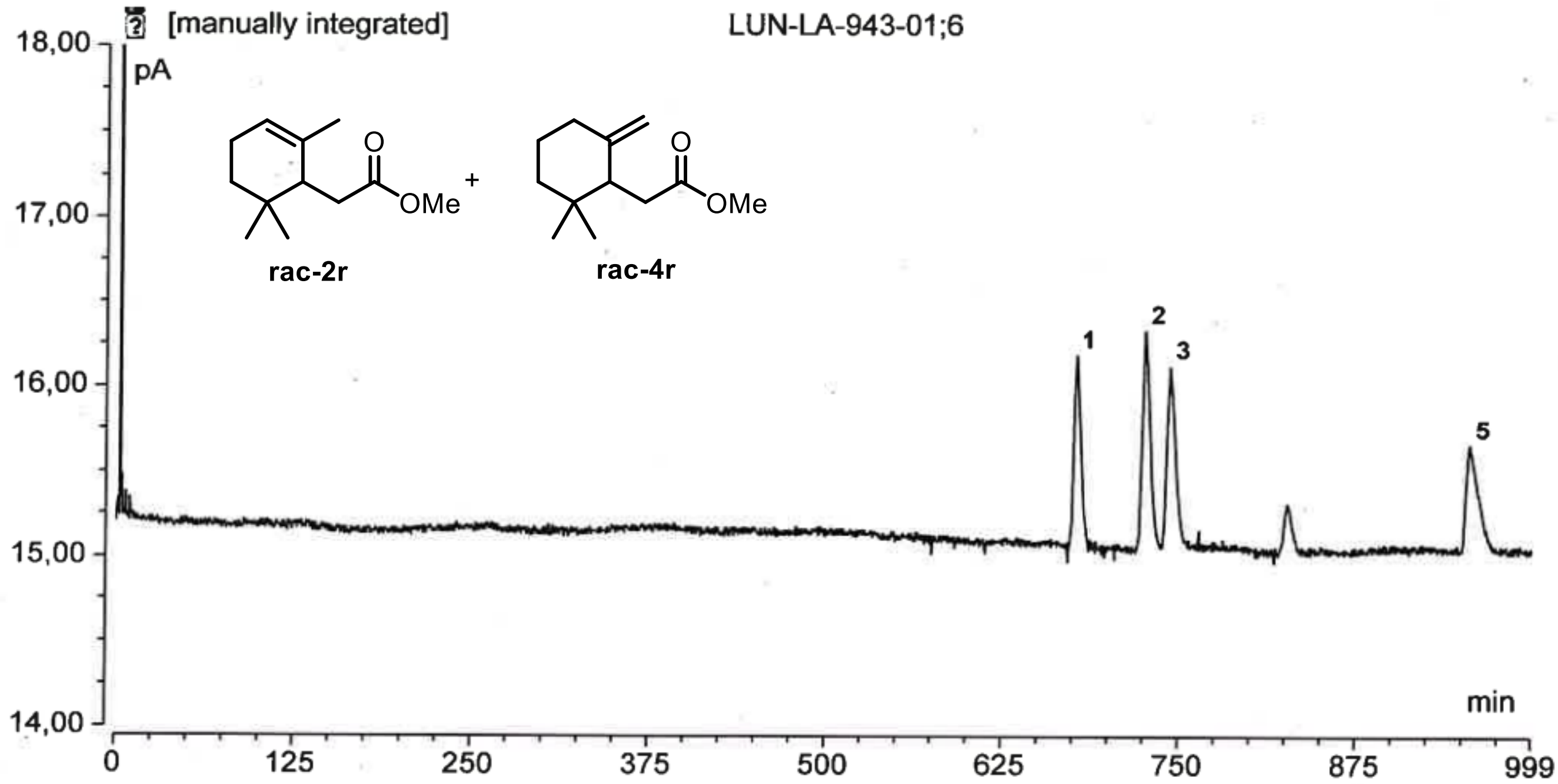

Peak#	Ret. Time (min)	Area%
1	676.91	22.59
2	725.37	28.41
3	743.00	27.05
5	954.83	21.94
Total		100.00

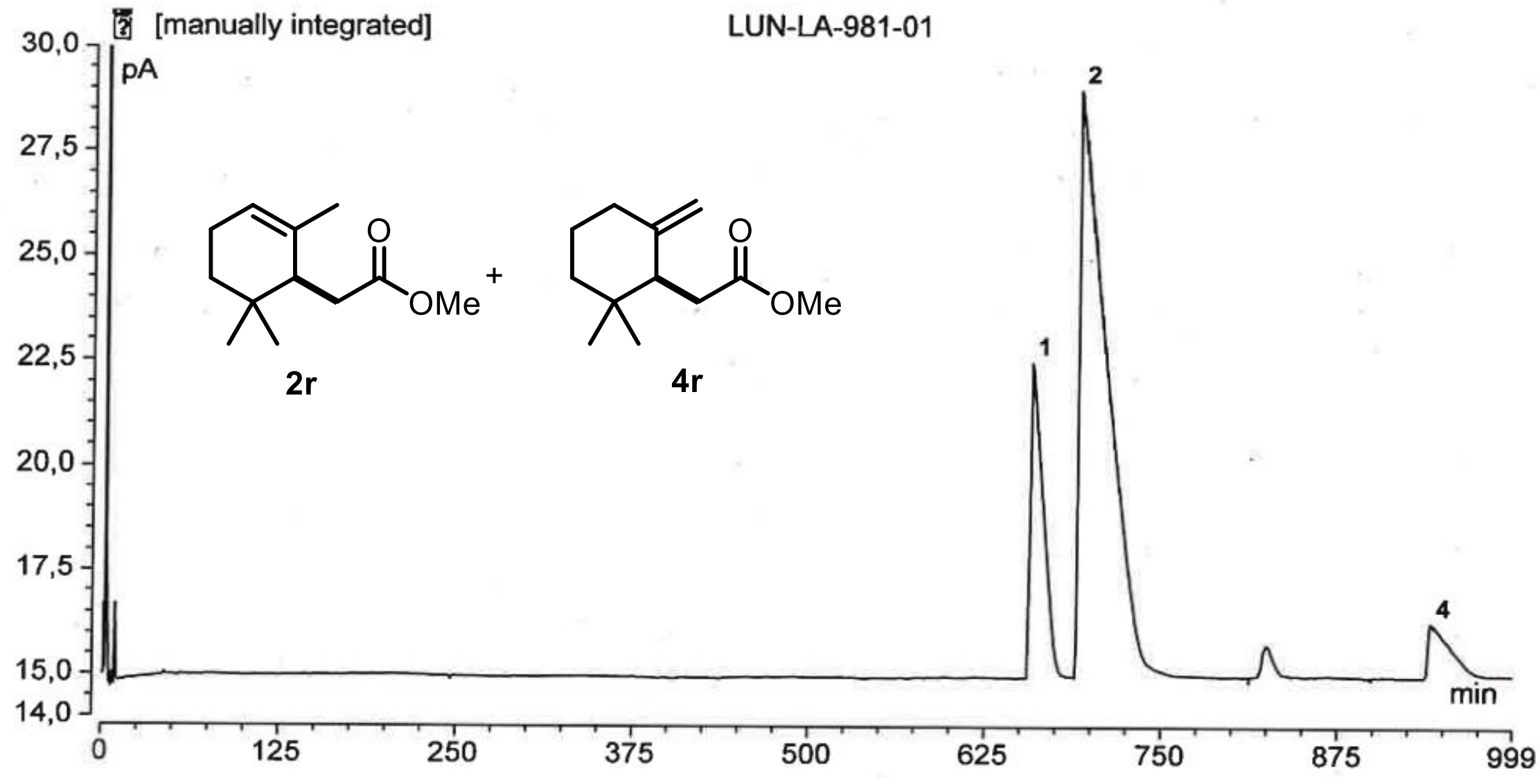

Peak#	Ret. Time (min)	Area%
1	657.52	18.44
2	690.65	75.76
4	940.81	5.80
Total		100.00

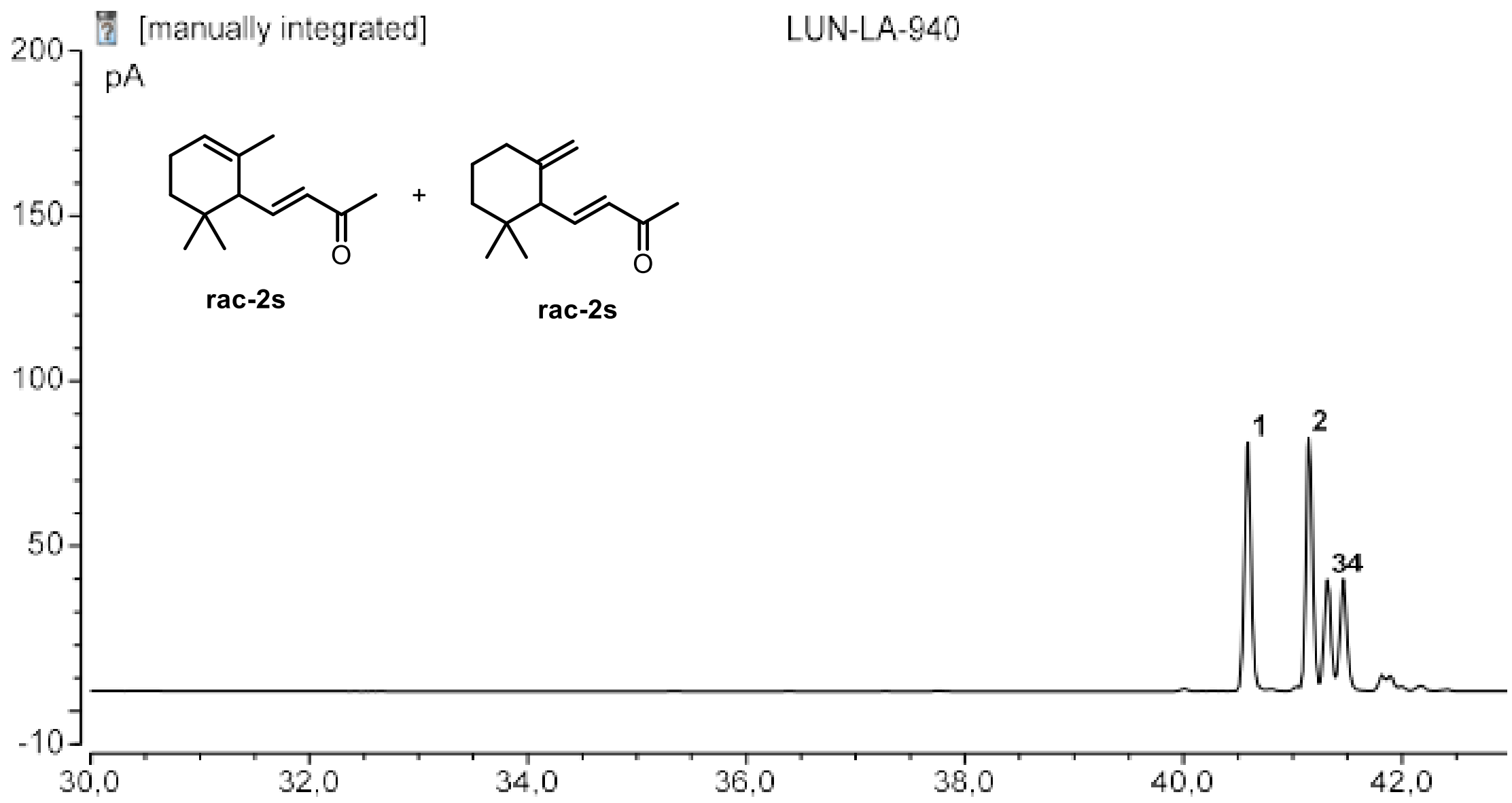

Peak#	Ret. Time (min)	Area%
1	40.59	35.69
2	41.15	34.10
3	41.32	14.60
5	41.46	15.61
Total		100.00

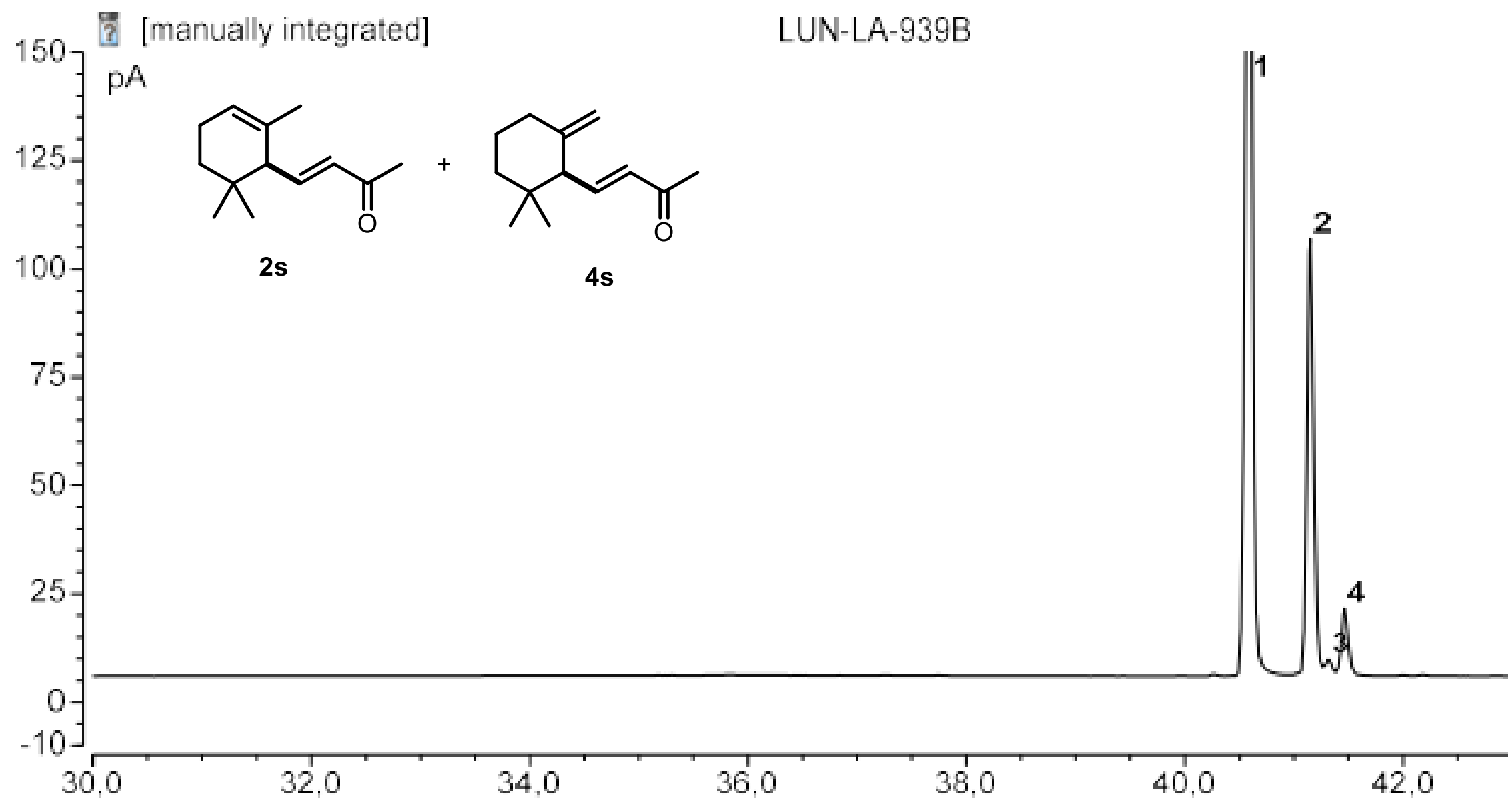

Peak#	Ret. Time (min)	Area%
1	40.59	67.32
2	41.15	28.16
3	41.31	0.47
5	41.46	4.04
Total		100.00

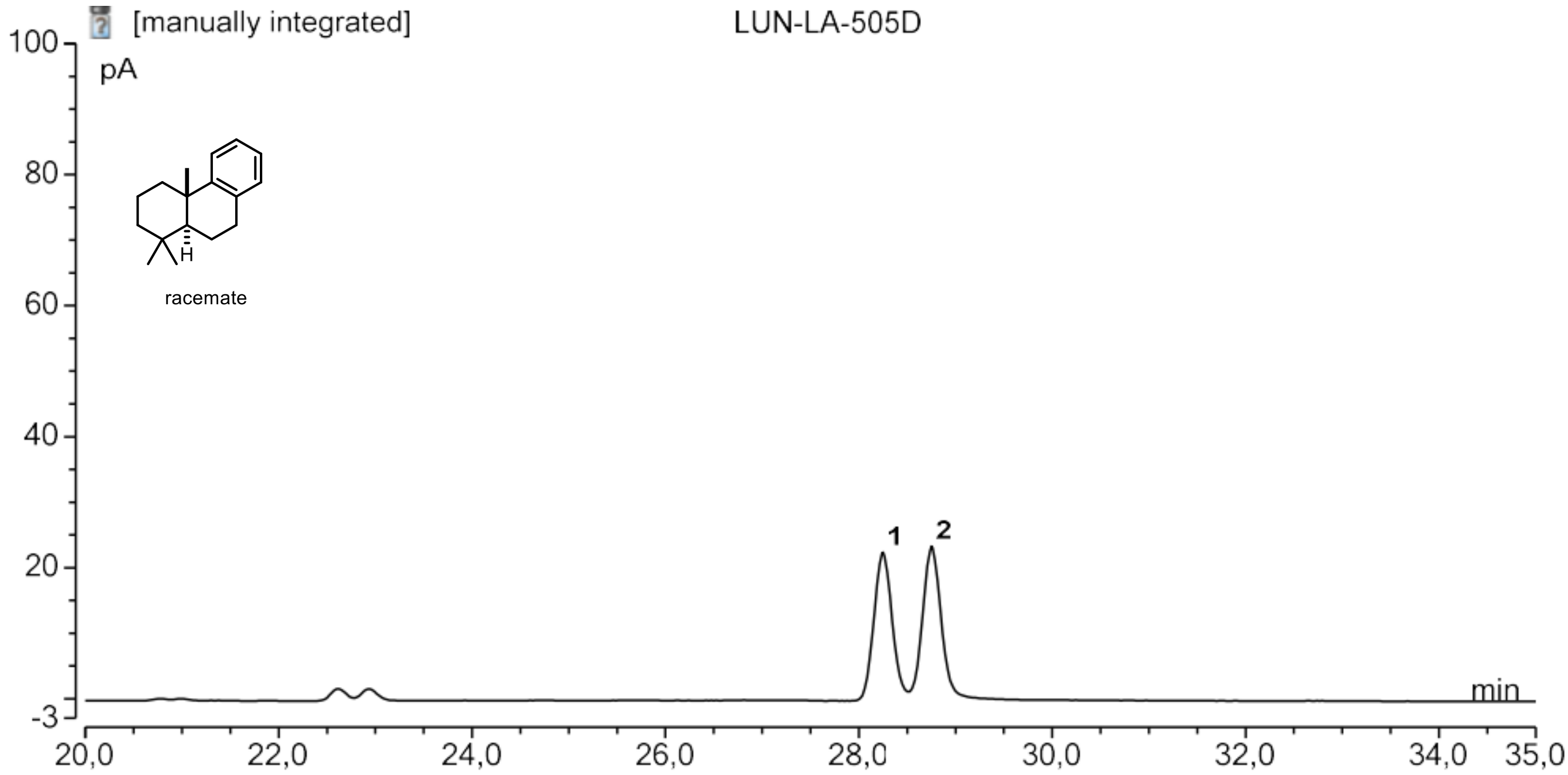

Peak#	Ret. Time (min)	Area%
1	28.25	49.26
2	28.75	50.74
Total		100.00

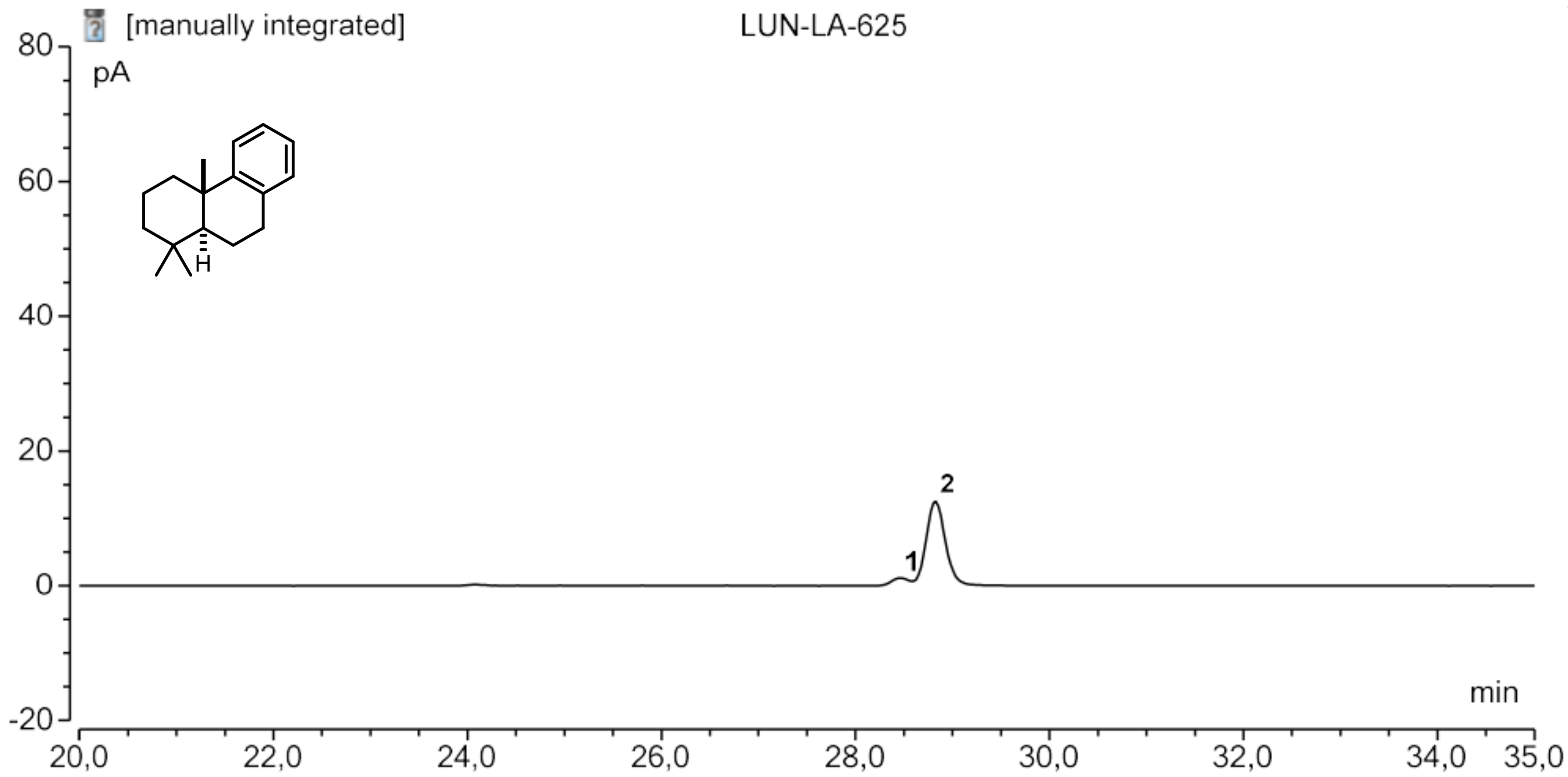

Peak#	Ret. Time (min)	Area%
1	28.46	4.95
2	28.83	95.05
Total		100.00

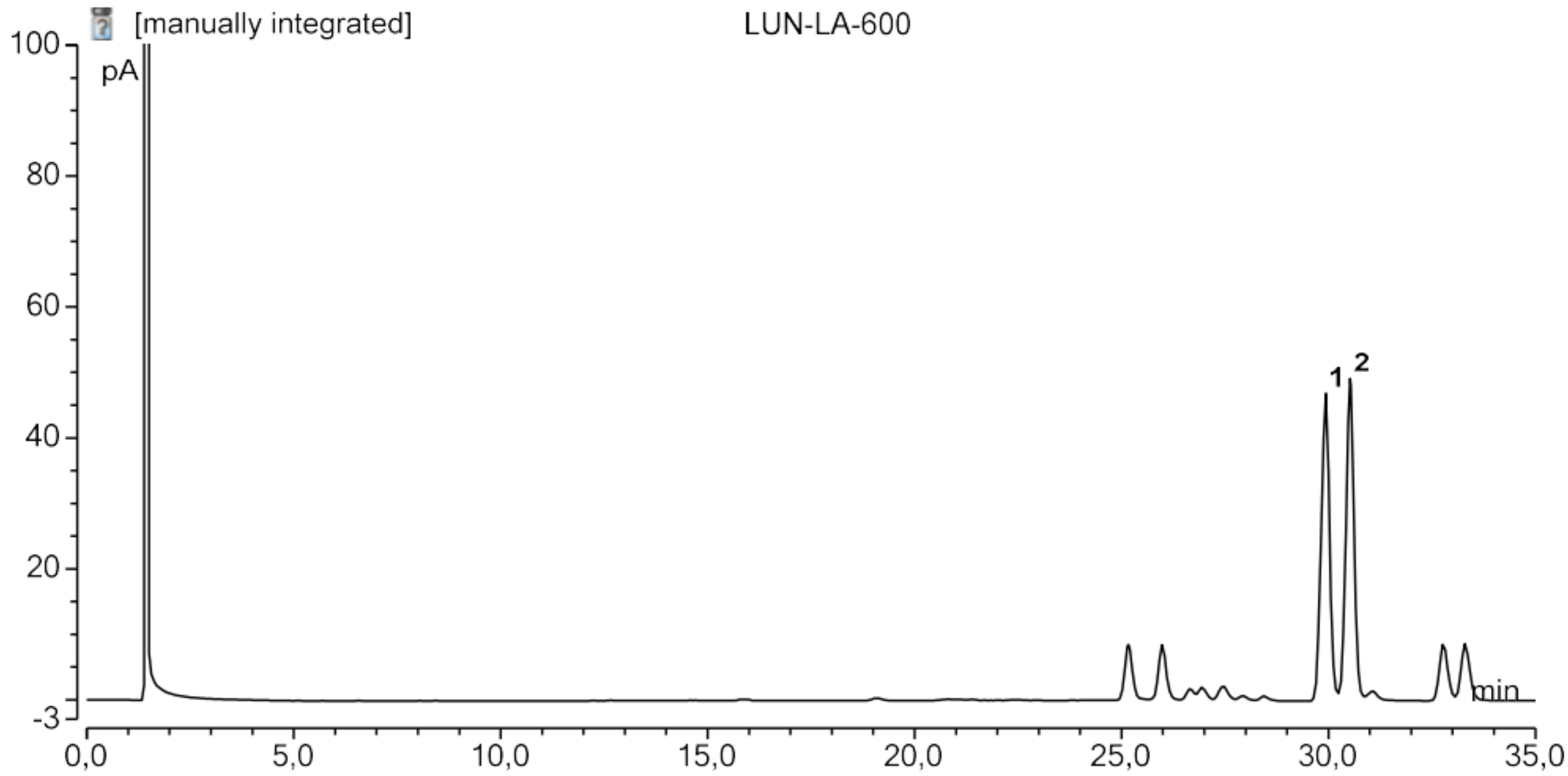

Peak#	Ret. Time (min)	Area%
1	29.94	50.16
2	49.84	49.84
Total		100.00

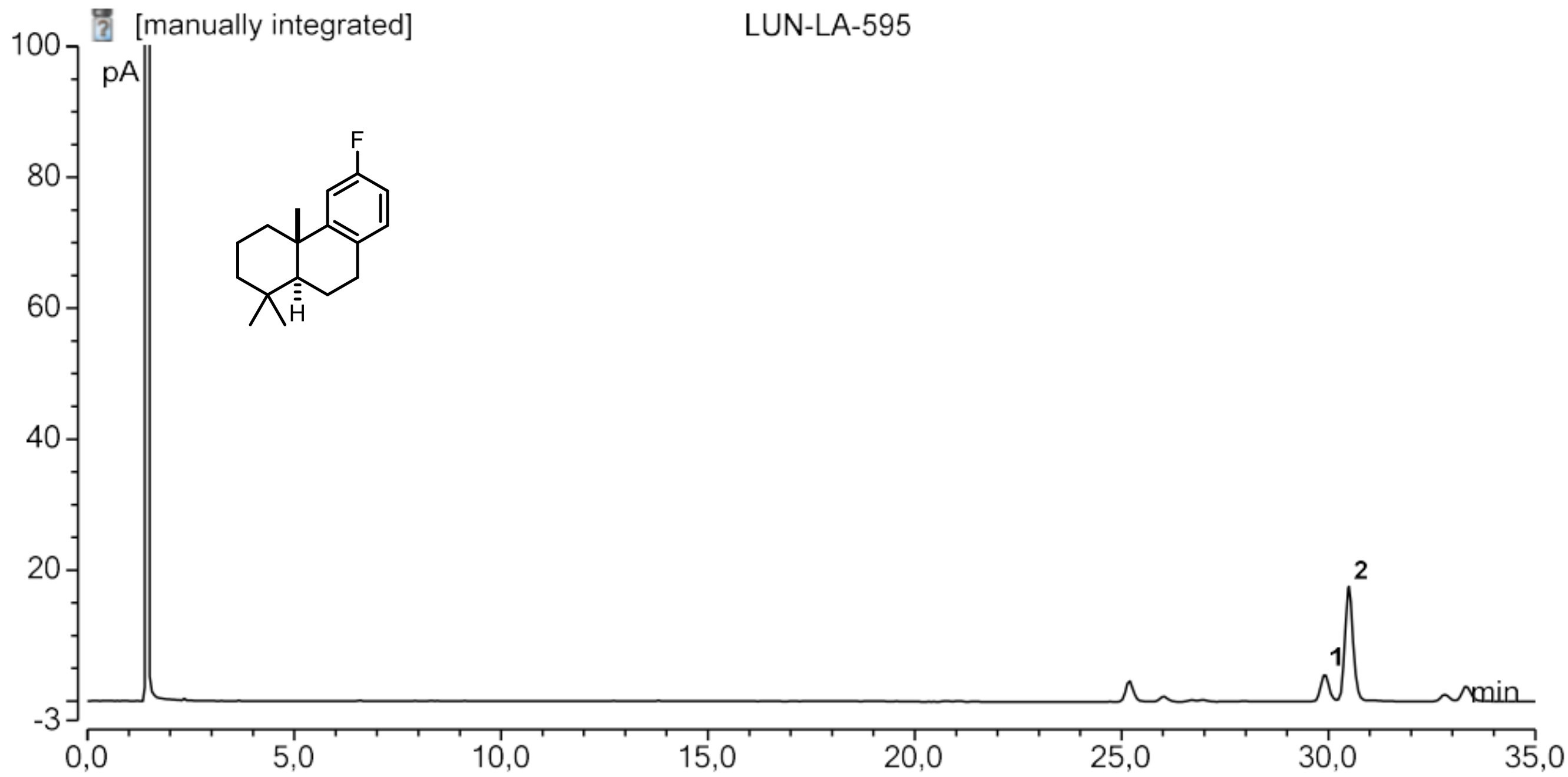

Peak#	Ret. Time (min)	Area%
1	29.91	17.95
2	30.50	82.05
Total		100.00

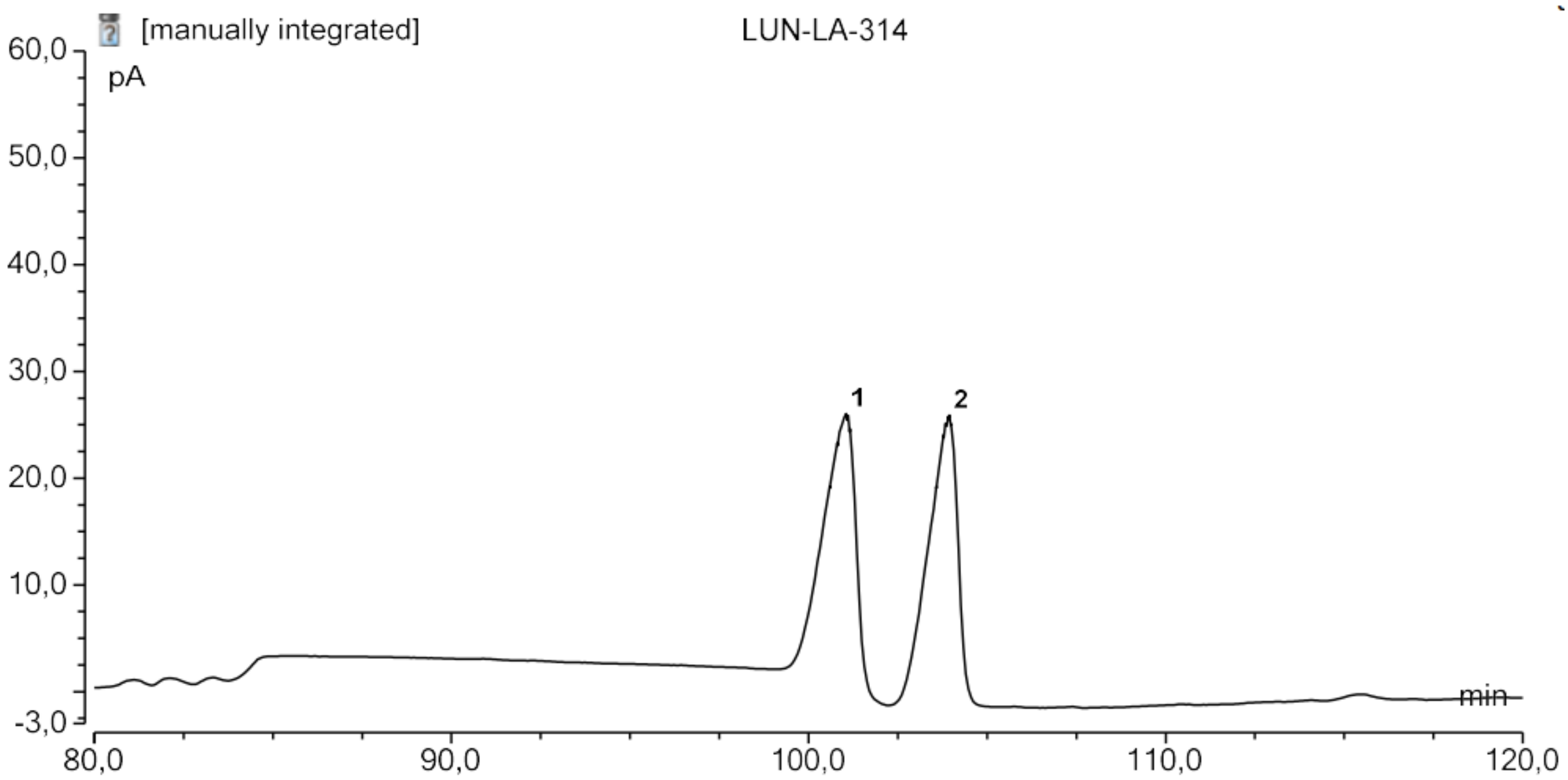

Peak#	Ret. Time (min)	Area%
1	101.05	49.78
2	103.94	50.22
Total		100.00

8

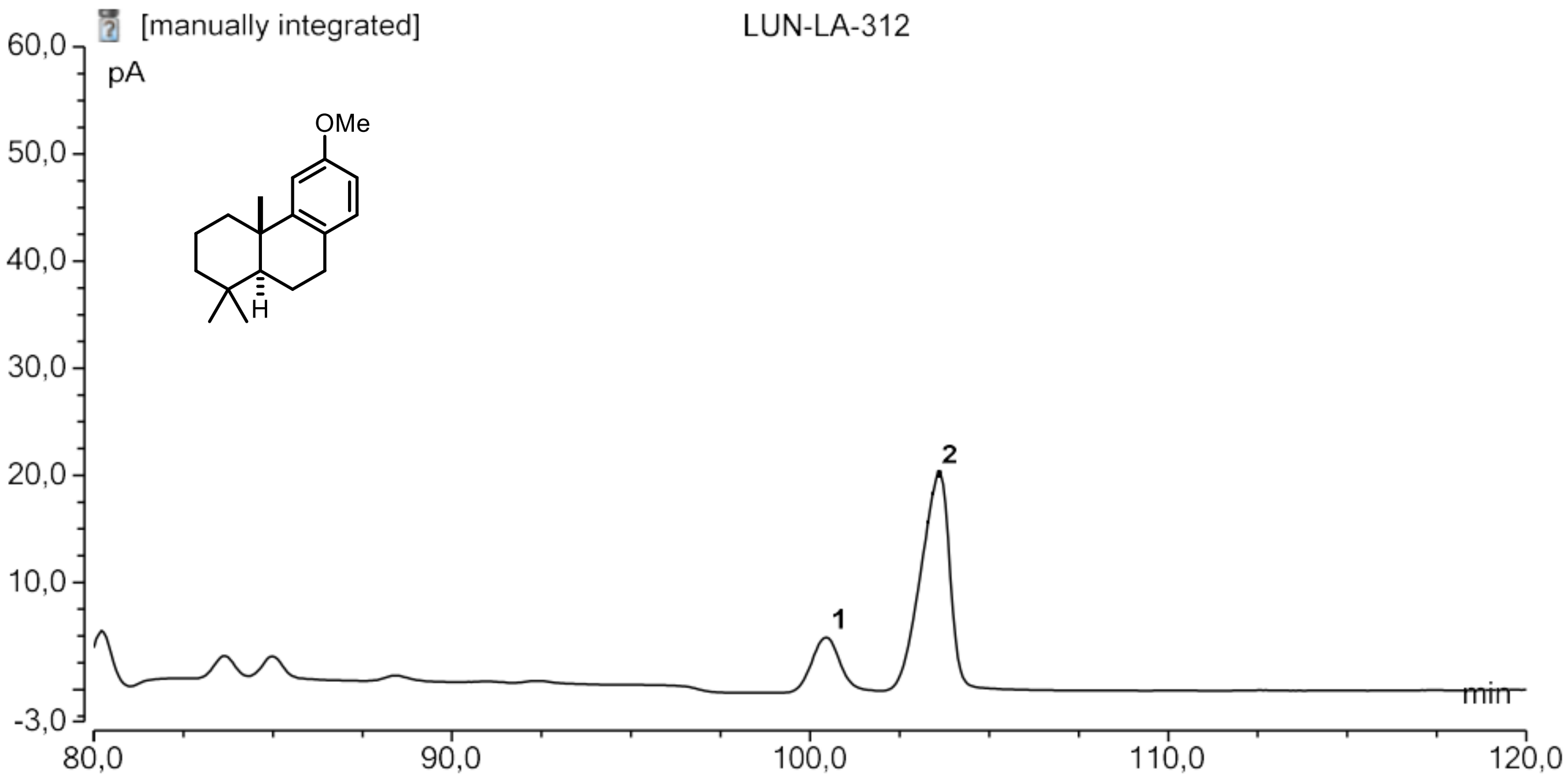

Peak#	Ret. Time (min)	Area%
1	100.48	18.04
2	103.57	81.96
Total		100.00

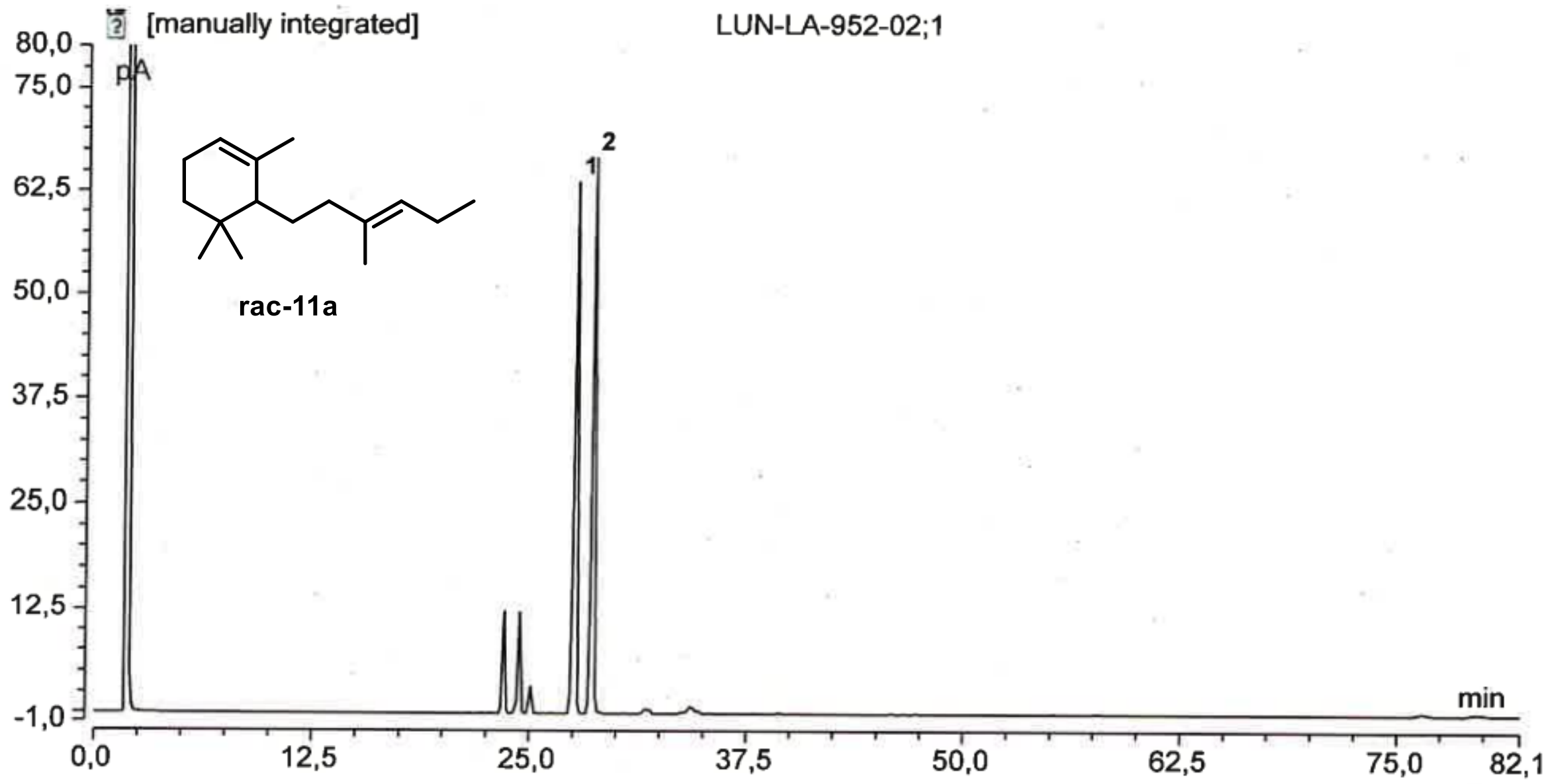

Peak#	Ret. Time (min)	Area%
1	27.60	49.40
2	28.61	50.60
Total		100.00

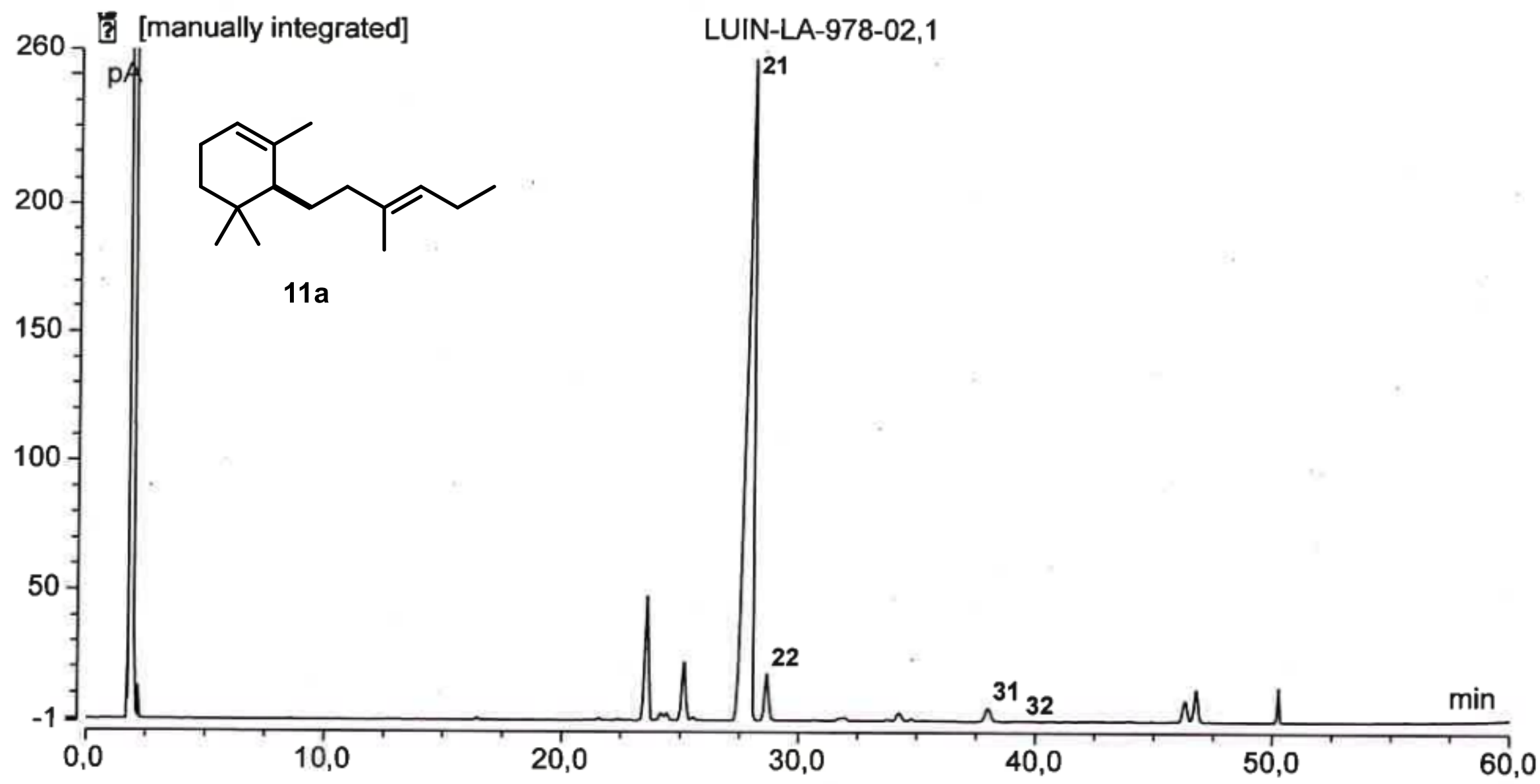

Peak#	Ret. Time (min)	Area%
21	28.00	96.14
22	28.65	3.86
Total		100.00

10.2. Copies of HPLC traces

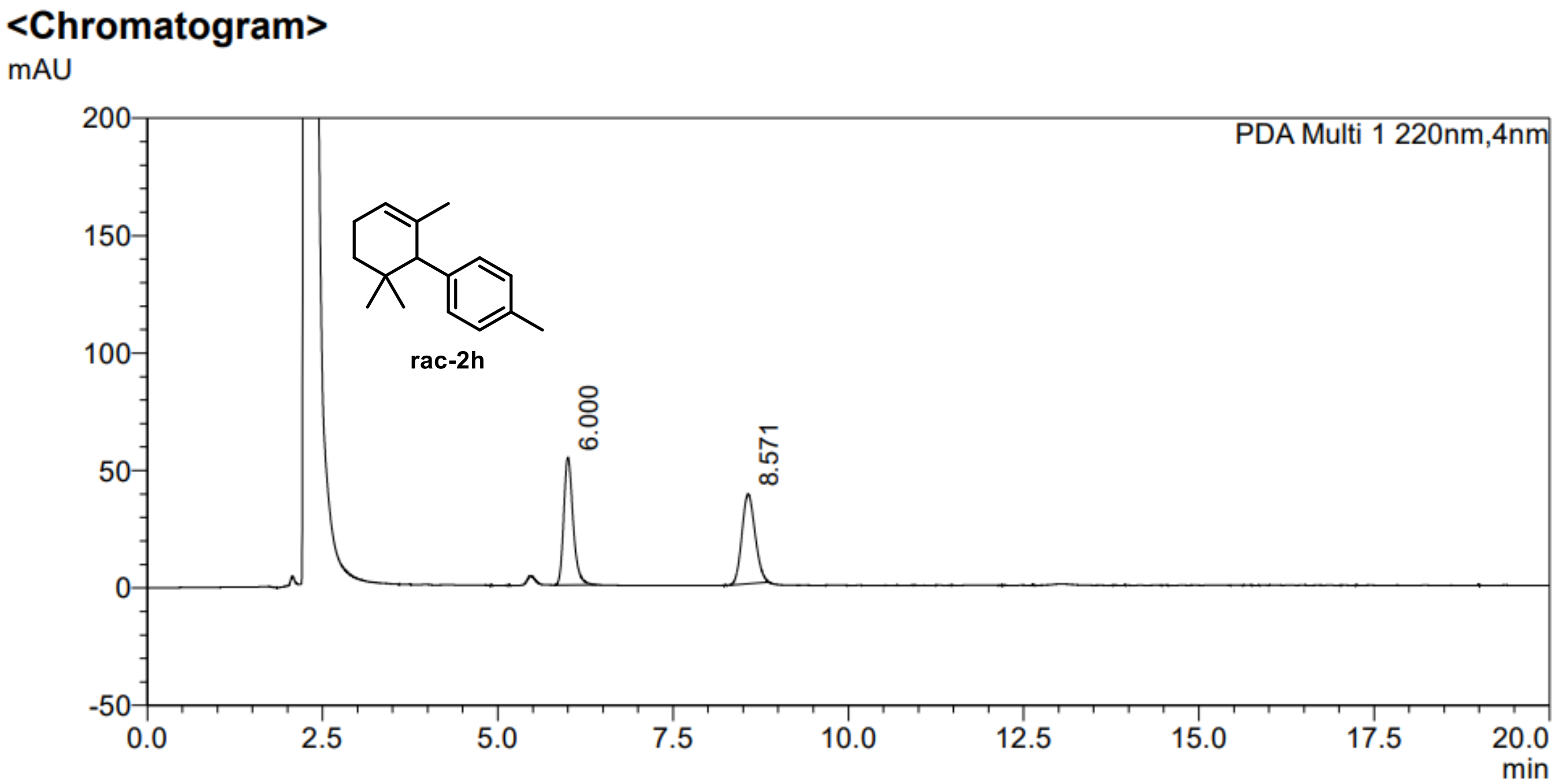

Peak#	Ret. Time (min)	Area%
1	6.00	50.16
2	8.57	49.84
Total		100.00

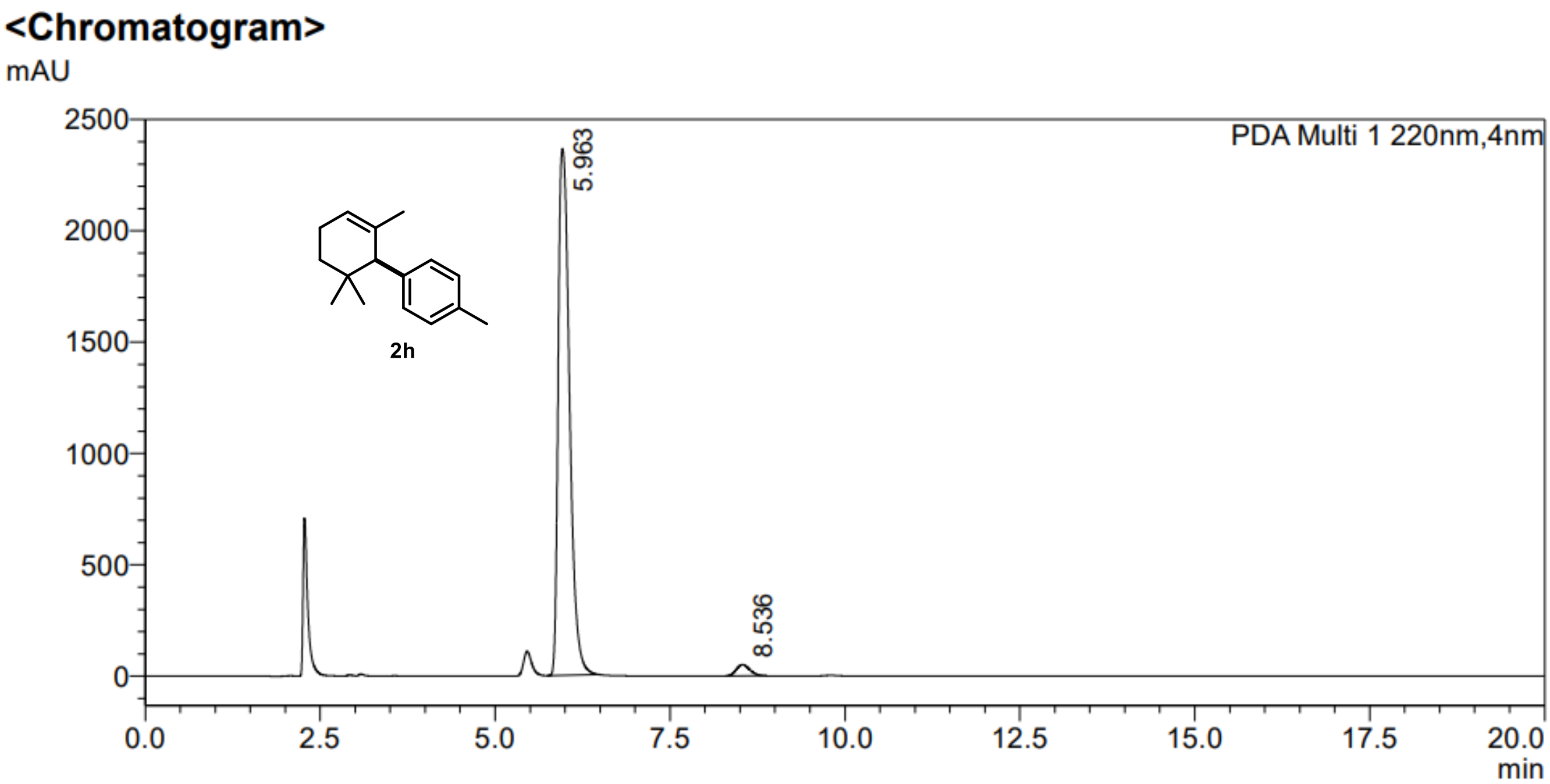

Peak#	Ret. Time (min)	Area%
1	5.96	97.50
2	8.54	2.50
Total		100.00

<Chromatogram>

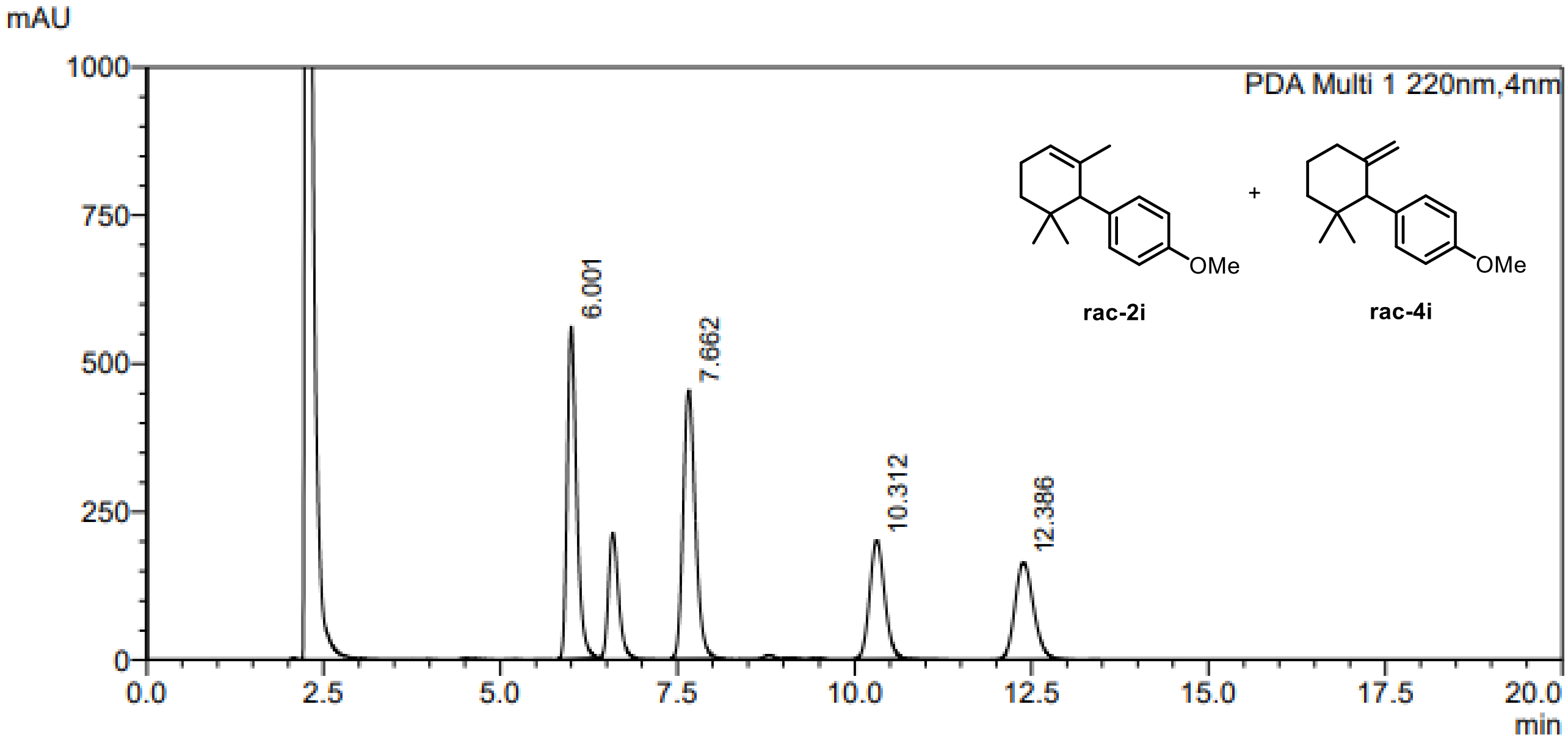

Peak#	Ret. Time (min)	Area%
1	6.00	31.17
2	7.66	32.78
3	10.31	17.89
4	12.39	18.16
Total		100.00

<Chromatogram>

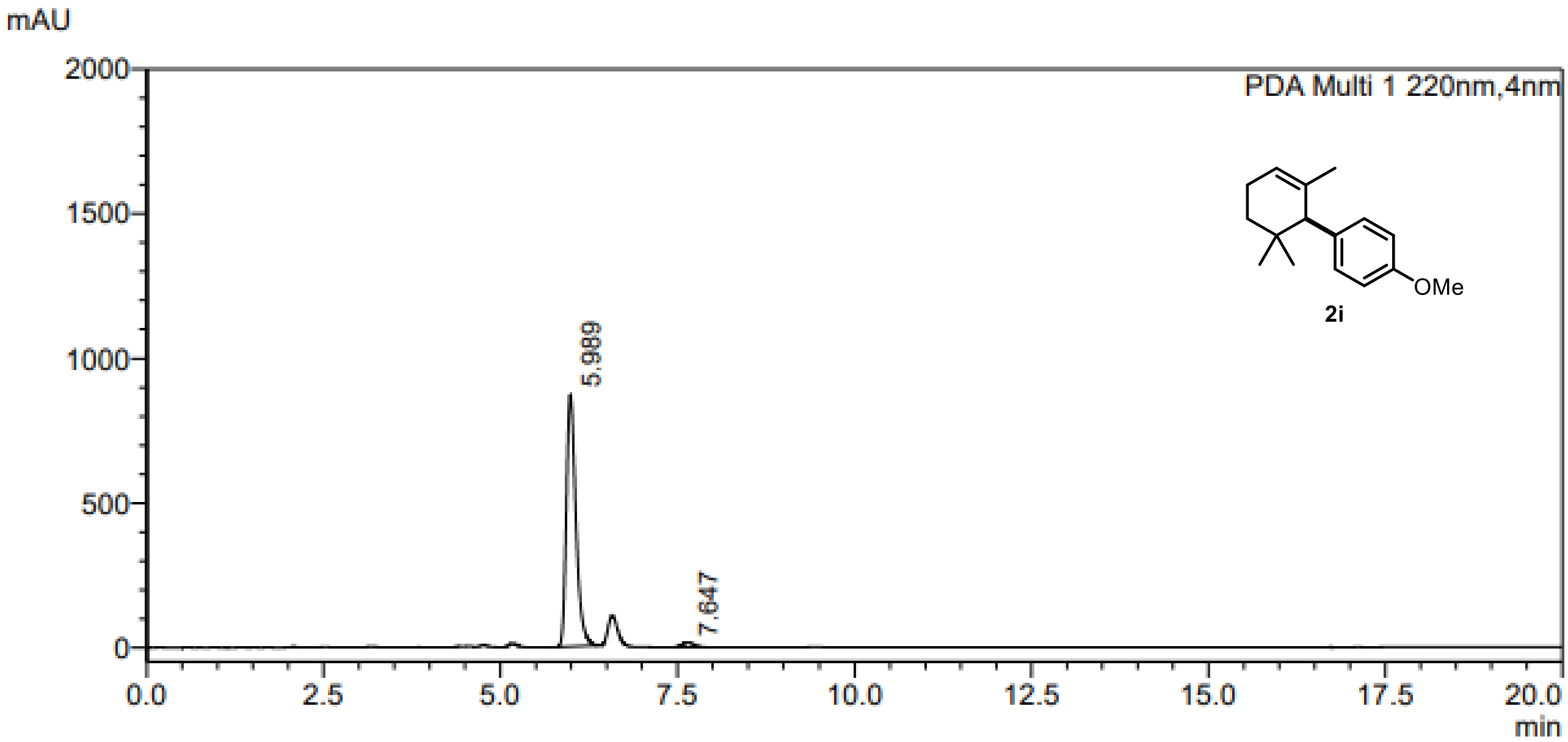

Peak#	Ret. Time (min)	Area%
1	5.99	97.78
2	7.65	2.22
Total		100.00

<Chromatogram>

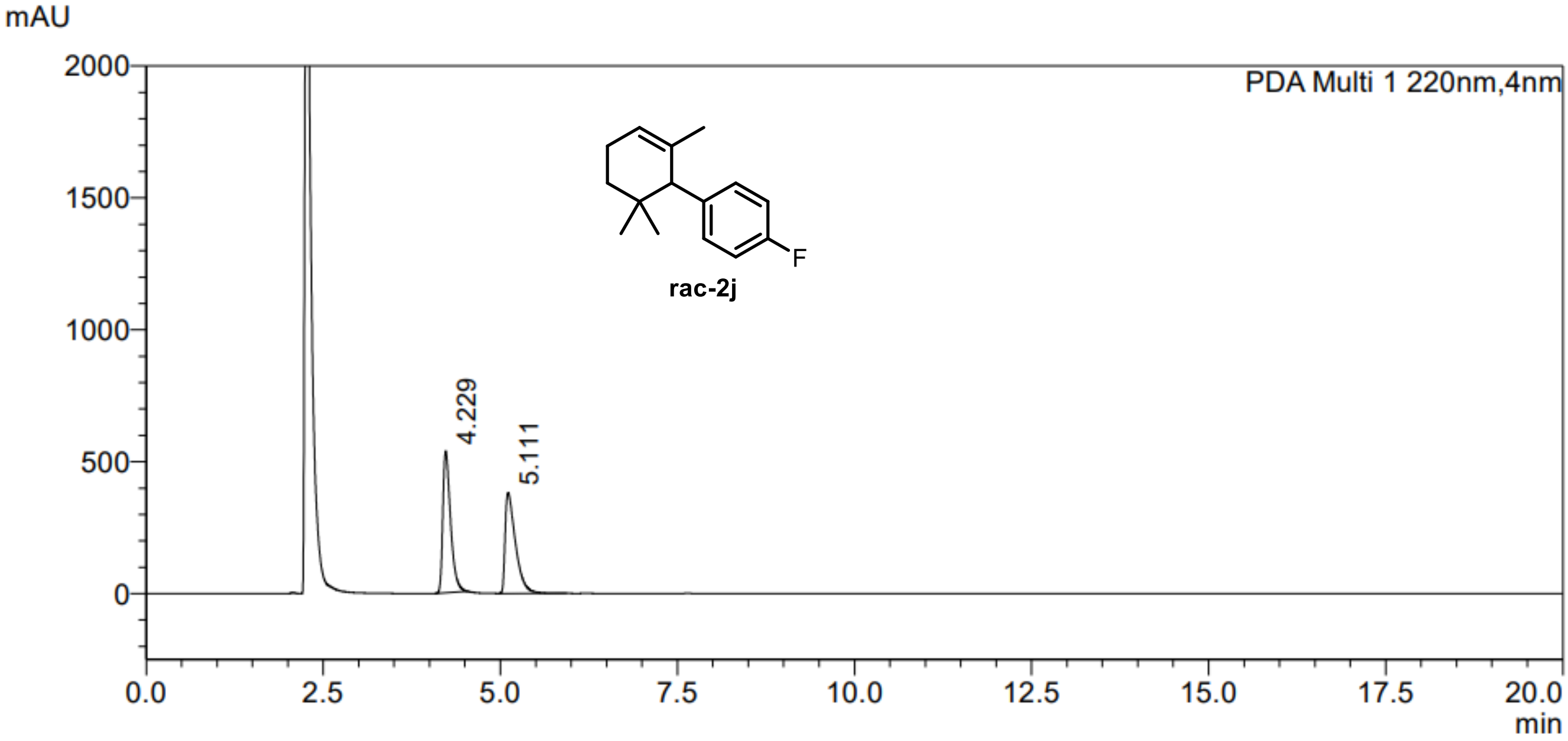

Peak#	Ret. Time (min)	Area%
1	4.23	51.32
2	5.11	48.68
Total		100.00

<Chromatogram>

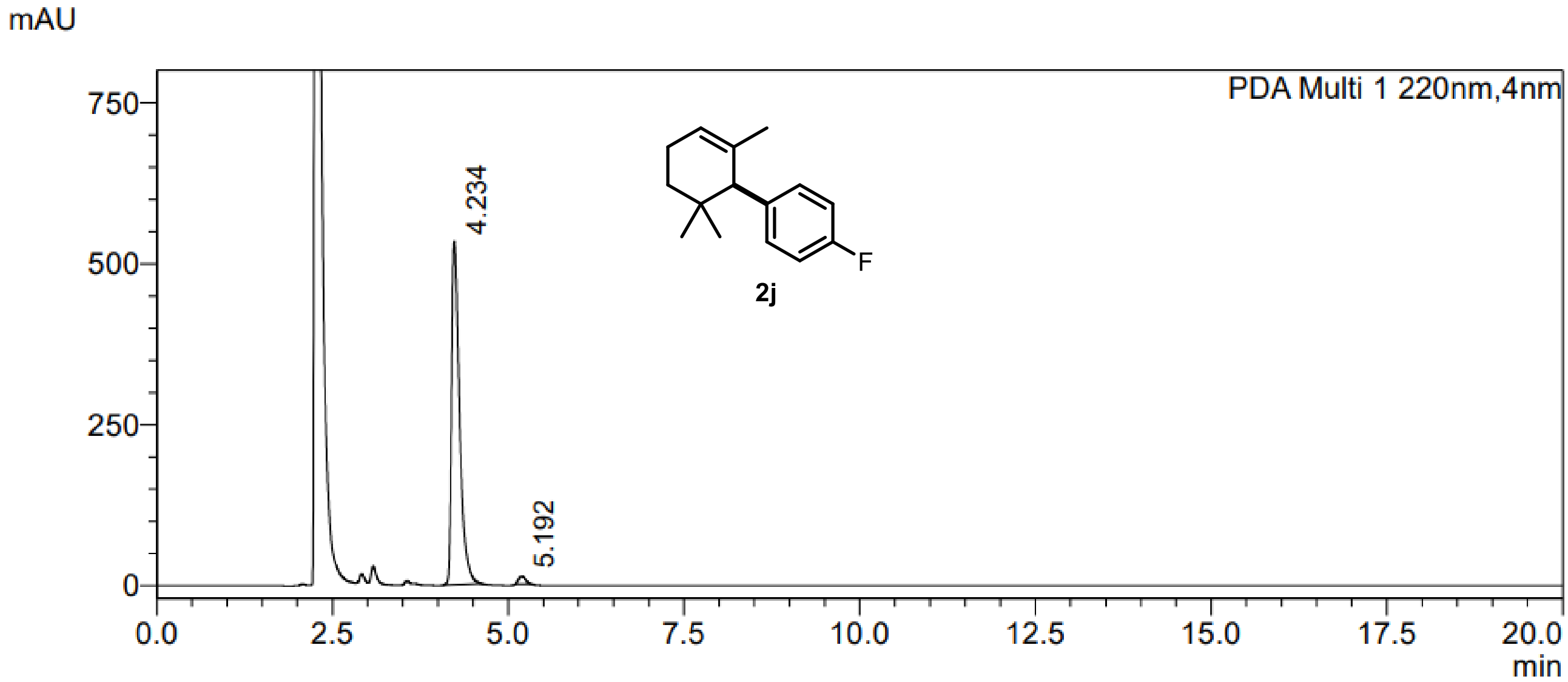

Peak#	Ret. Time (min)	Area%
1	4.23	98.04
2	5.19	1.96
Total		100.00

<Chromatogram>

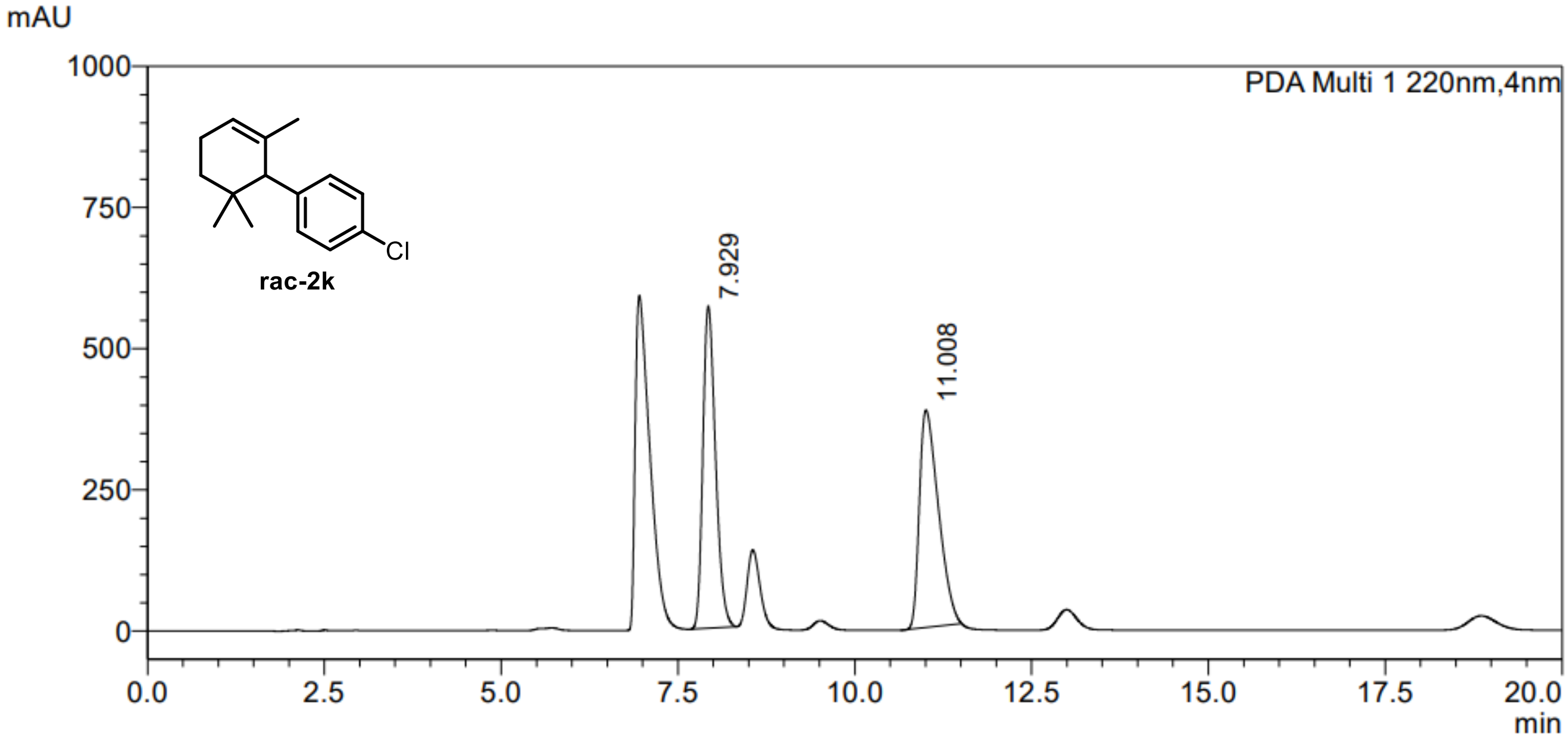

Peak#	Ret. Time (min)	Area%
1	7.93	49.60
2	11.01	50.40
Total		100.00

<Chromatogram>

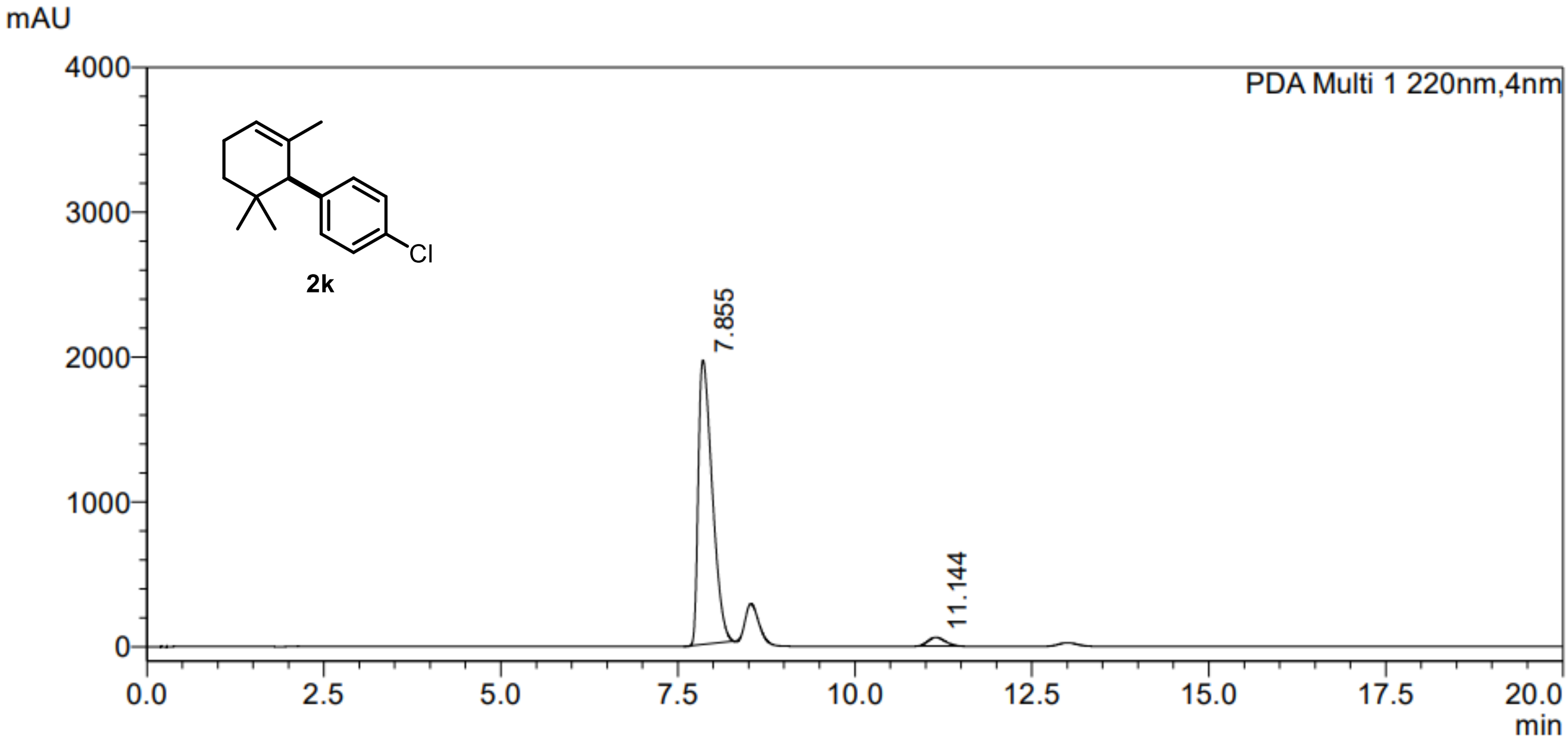

Peak#	Ret. Time (min)	Area%
1	7.86	96.44
2	11.14	3.56
Total		100.00

<Chromatogram>

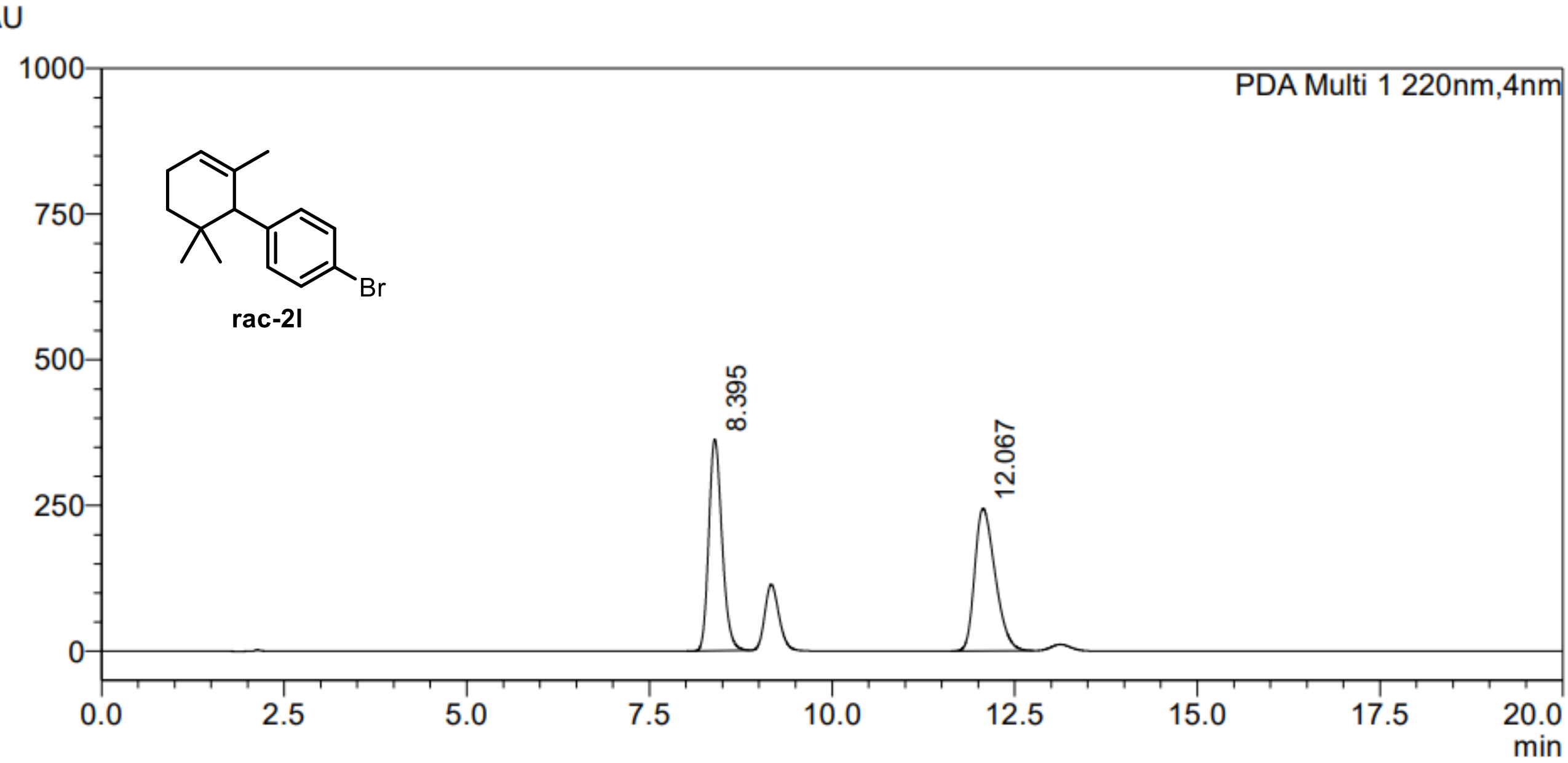

Peak#	Ret. Time (min)	Area%
1	8.40	49.03
2	12.07	50.97
Total		100.00

<Chromatogram>

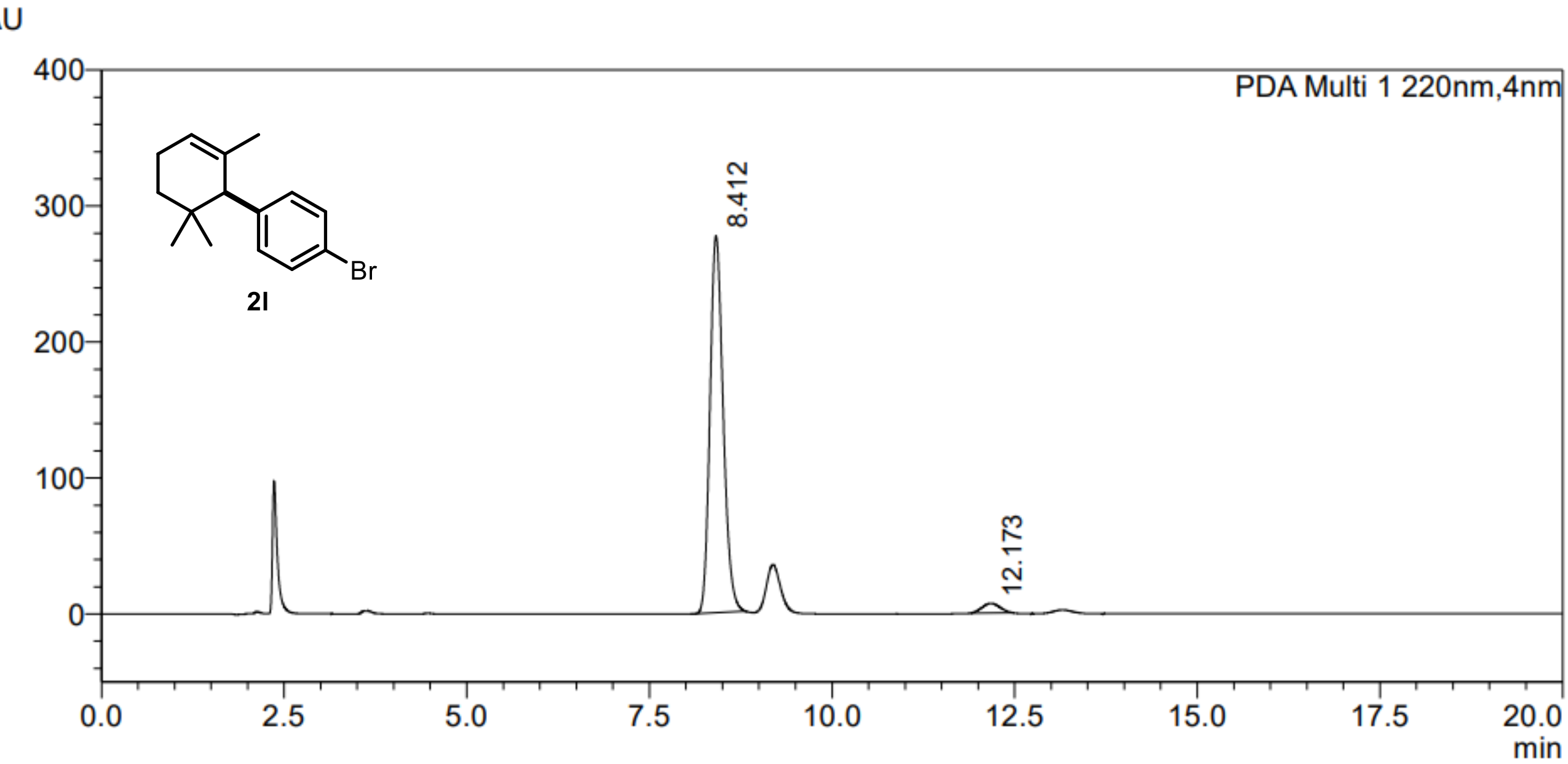

Peak#	Ret. Time (min)	Area%
1	8.41	96.70
2	12.17	3.30
Total		100.00

<Chromatogram>

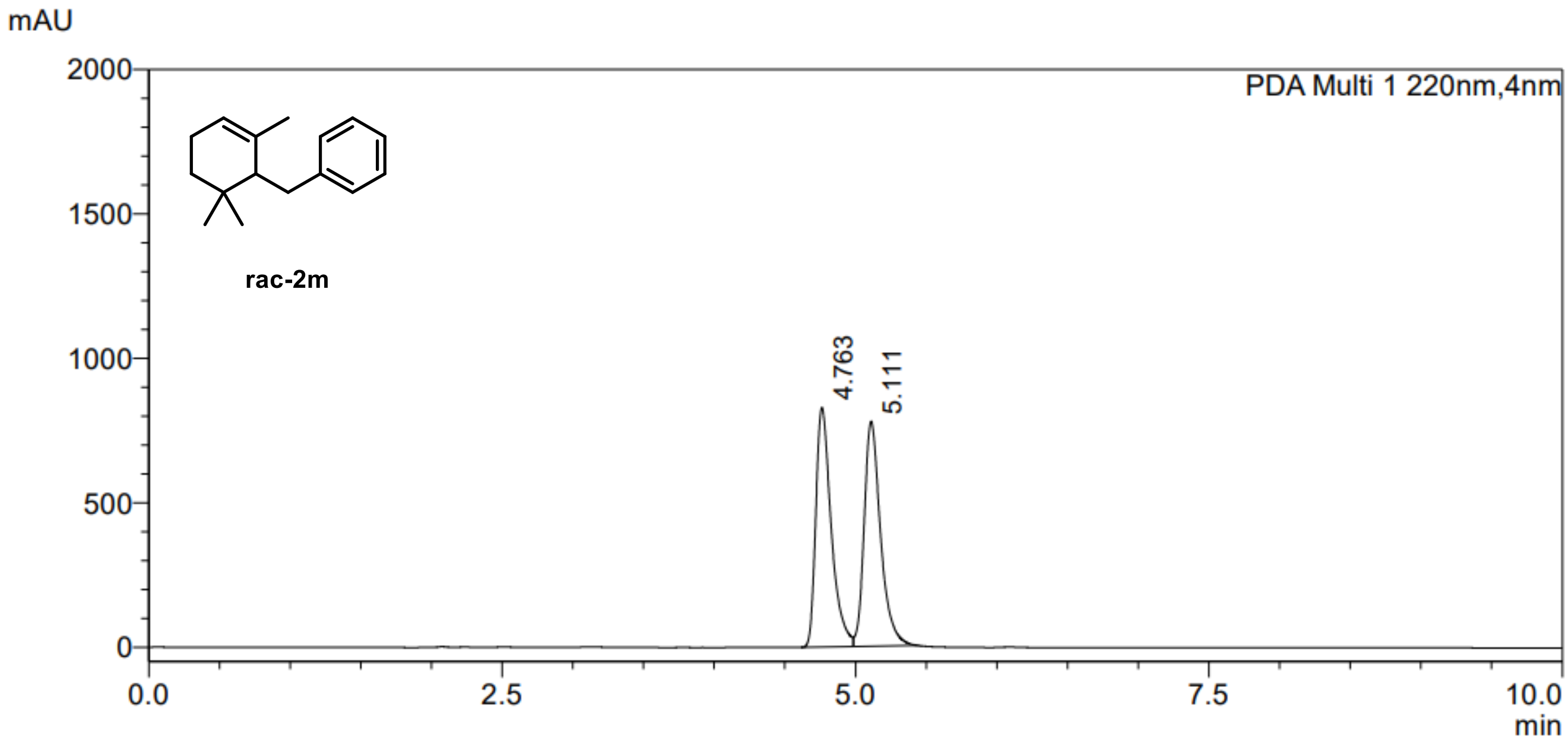

Peak#	Ret. Time (min)	Area%
1	4.76	50.00
2	5.11	50.00
Total		100.00

<Chromatogram>

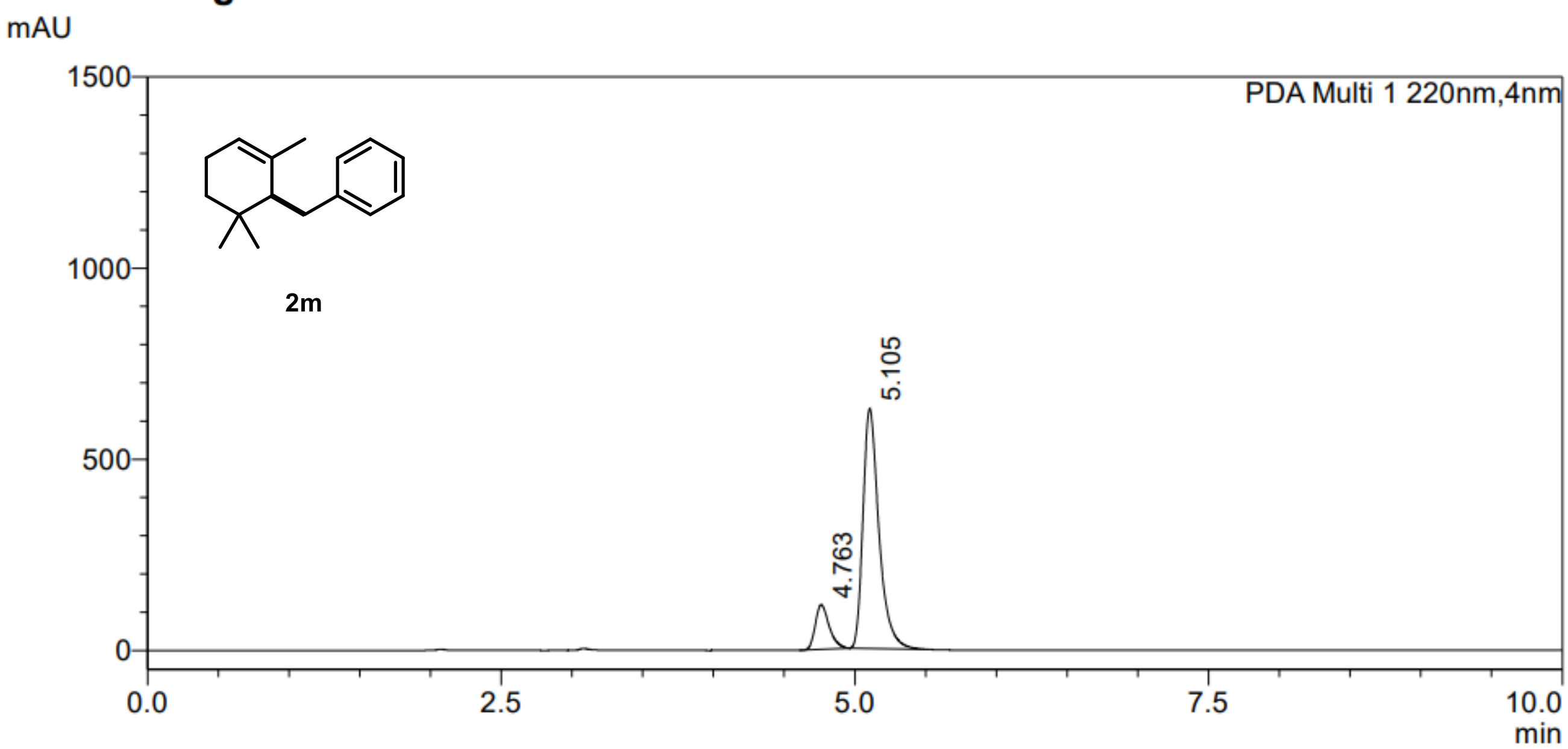

Peak#	Ret. Time (min)	Area%
1	4.76	13.93
2	5.11	86.07
Total		100.00

<Chromatogram>

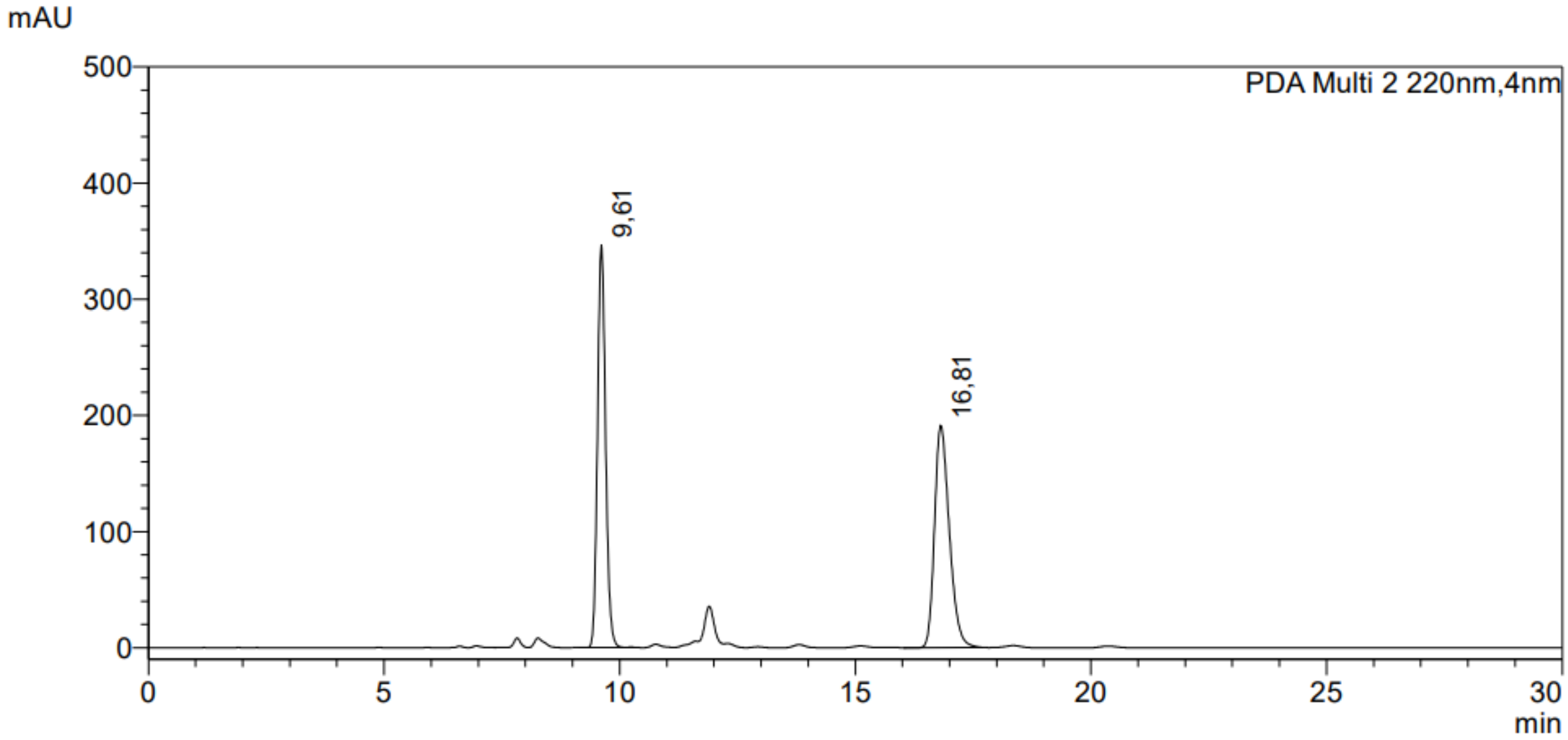

Peak#	Ret. Time (min)	Area%
1	9.61	49.82
2	16.81	50.18
Total		100.00

<Chromatogram>

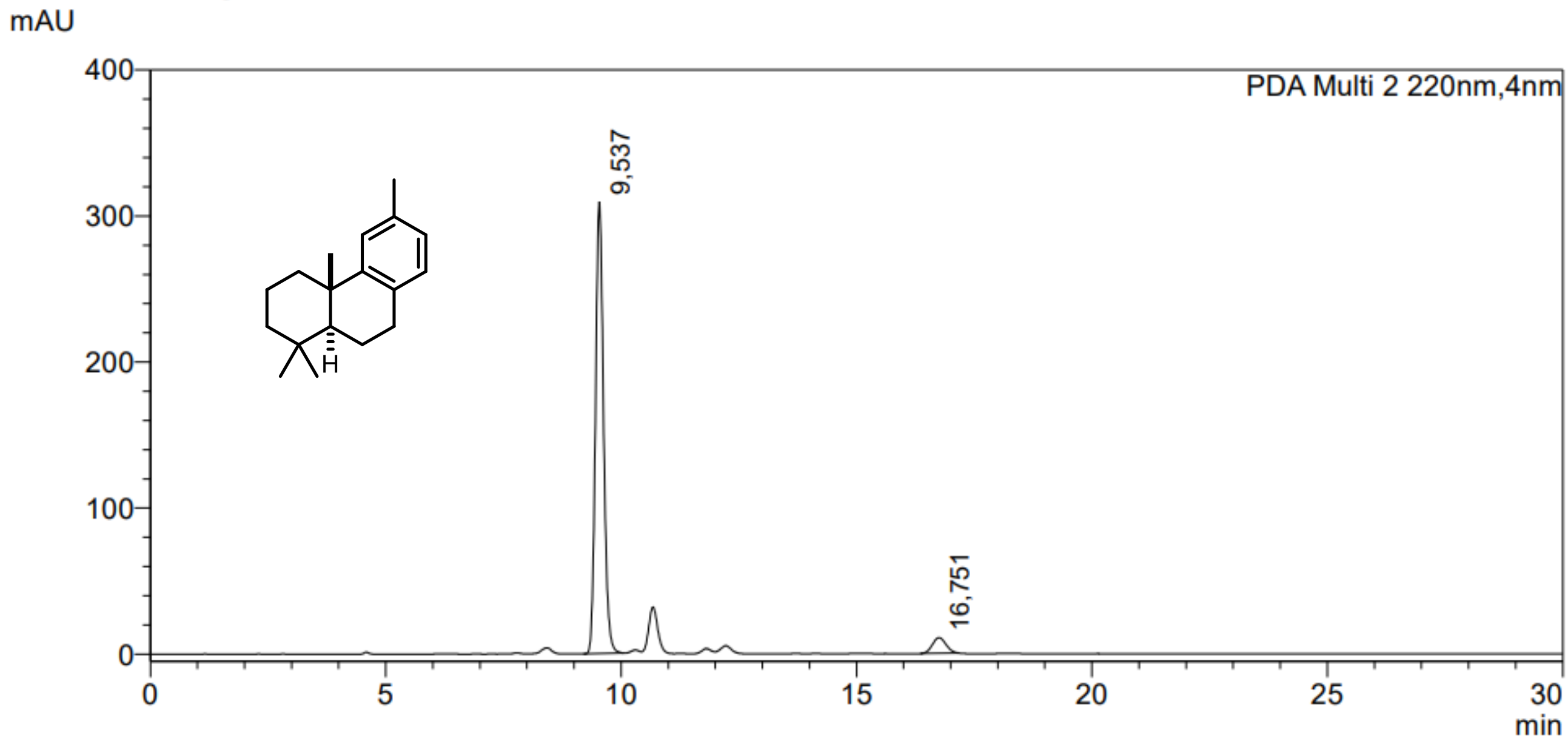

Peak#	Ret. Time (min)	Area%
1	9.54	96.66
2	16.75	3.34
Total		100.00

<Chromatogram>

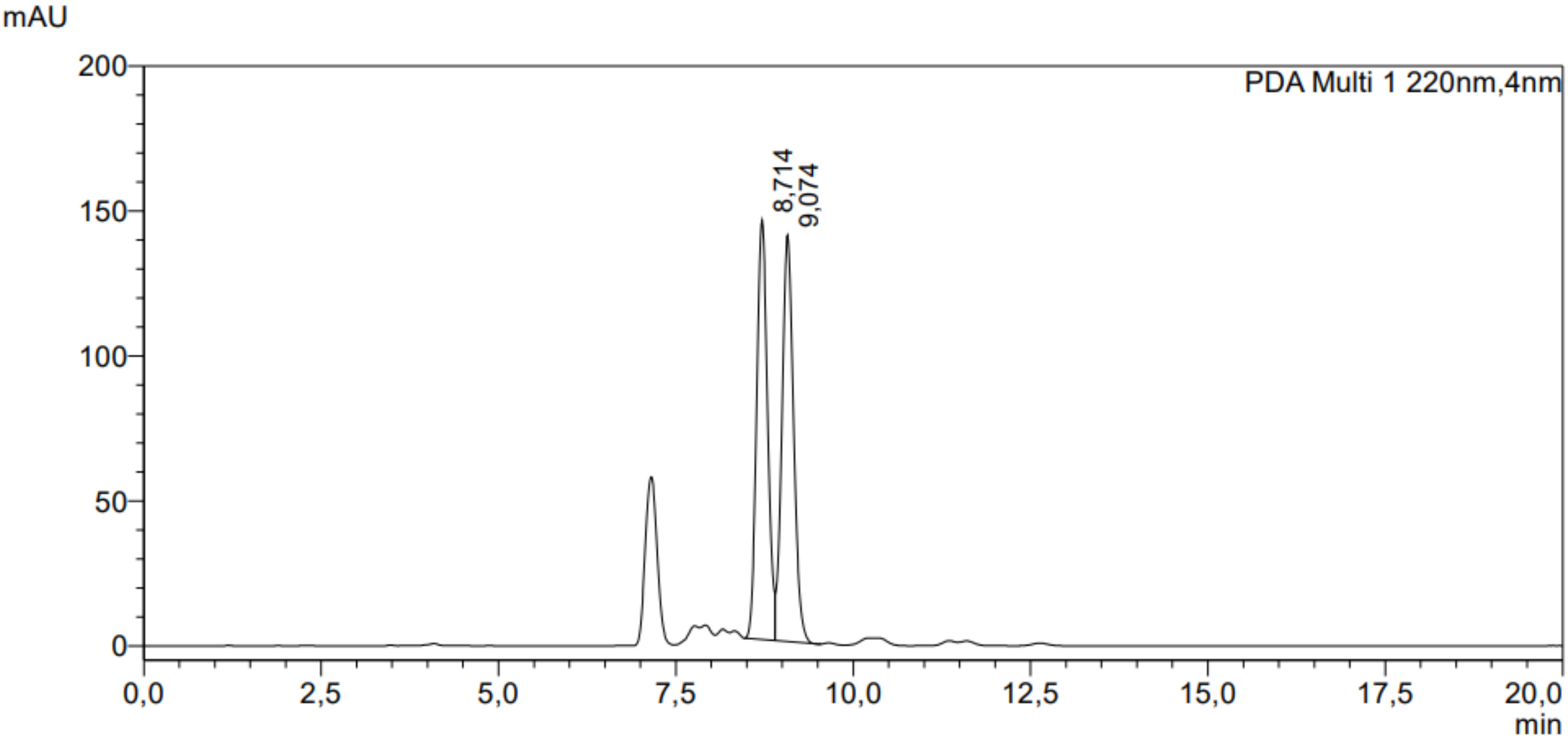

Peak#	Ret. Time (min)	Area%
1	8.71	49.13
2	9.07	50.87
Total		100.00

<Chromatogram>

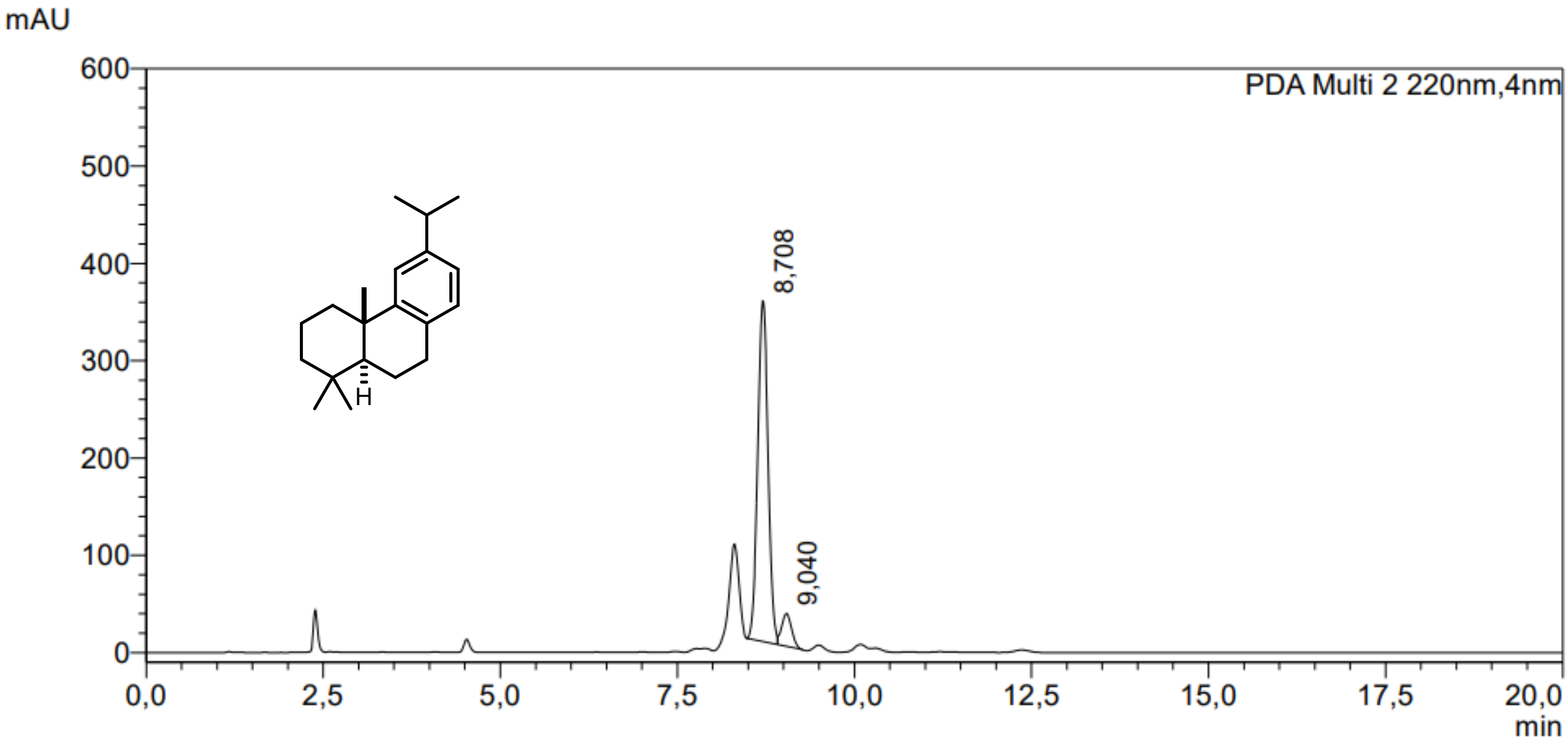

Peak#	Ret. Time (min)	Area%
1	8.71	91.61
2	9.04	8.39
Total		100.00

<Chromatogram>

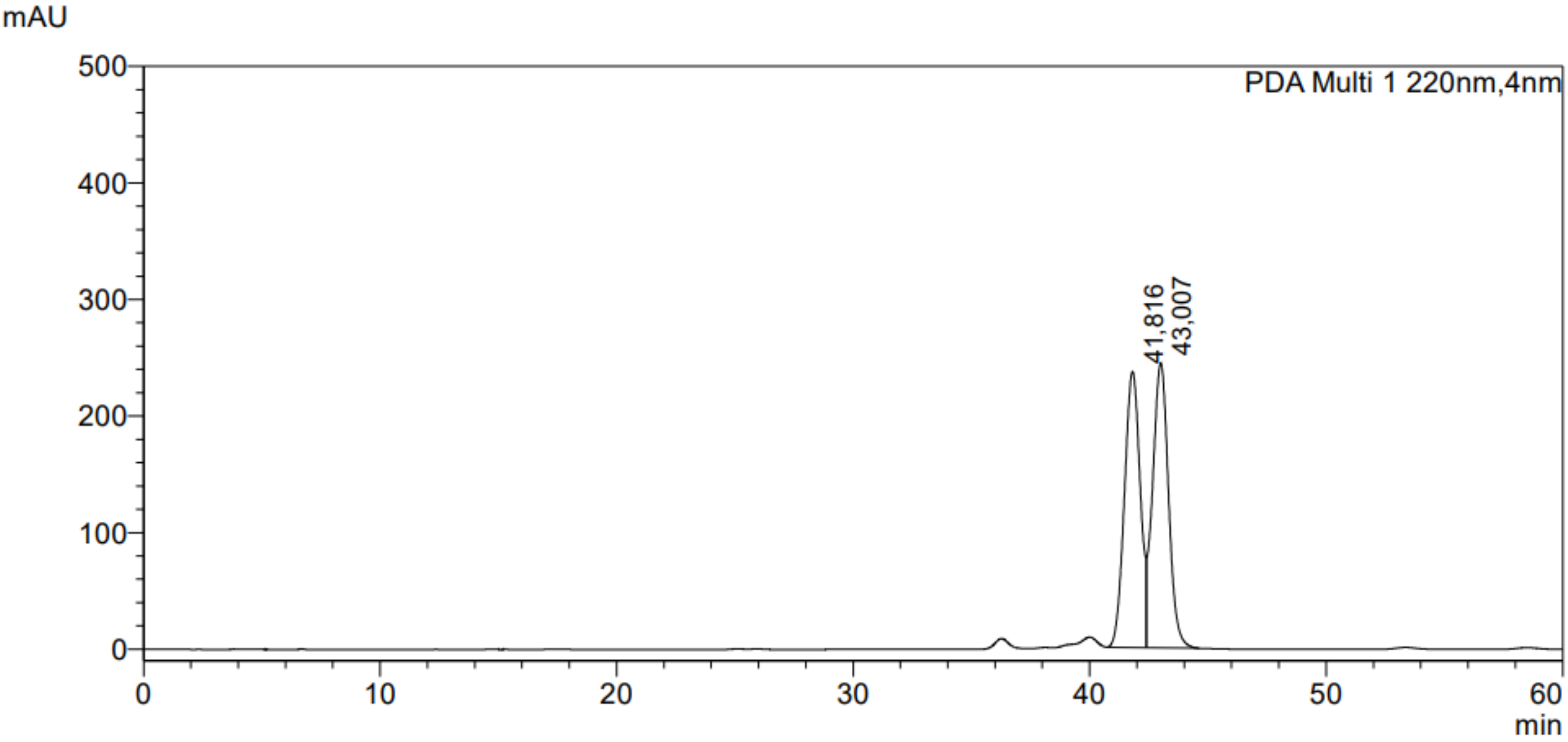

Peak#	Ret. Time (min)	Area%
1	41.82	48.95
2	43.01	51.05
Total		100.00

<Chromatogram>

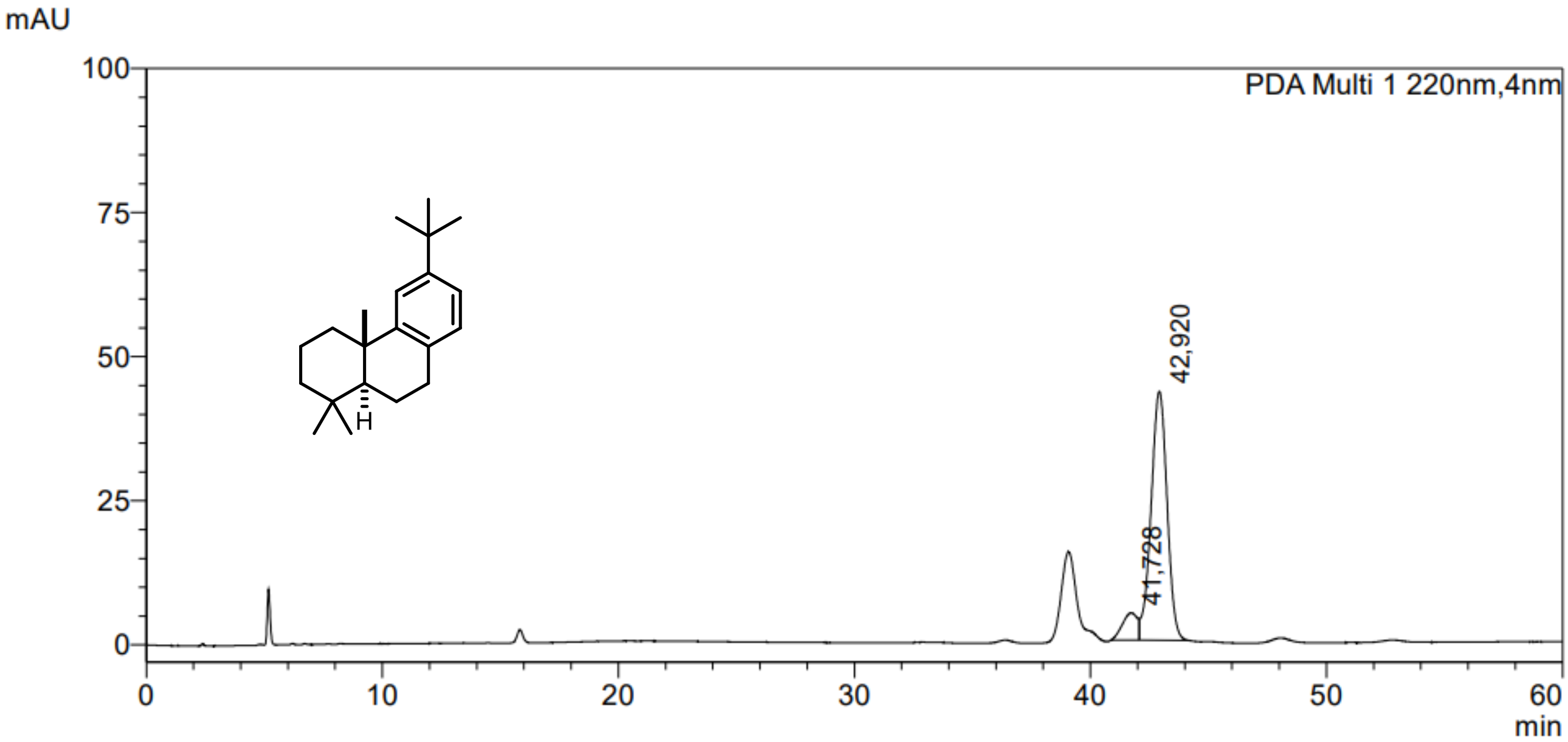

Peak#	Ret. Time (min)	Area%
1	41.73	9.31
2	42.92	90.69
Total		100.00

<Chromatogram>

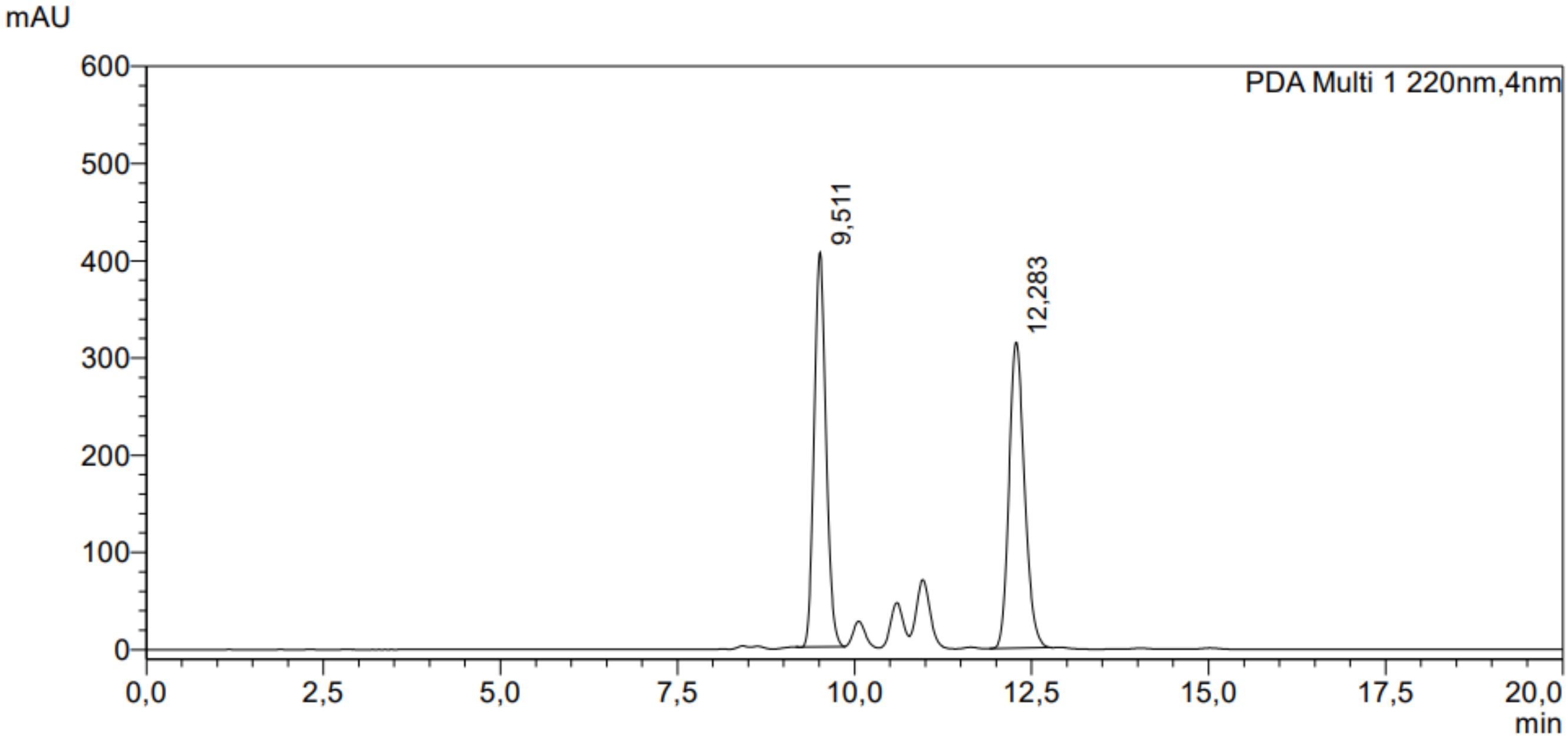

Peak#	Ret. Time (min)	Area%
1	9.51	49.63
2	12.28	50.37
Total		100.00

<Chromatogram>

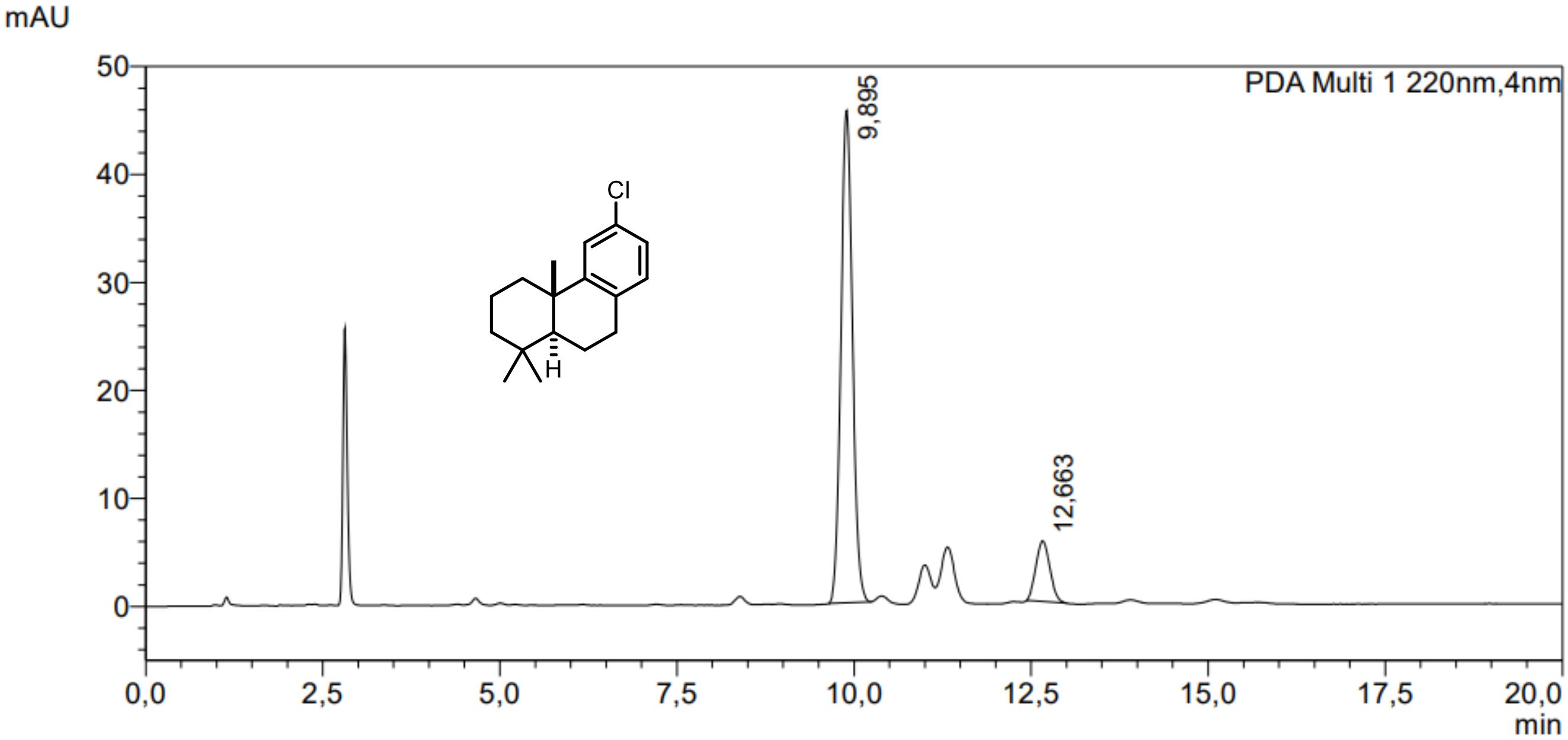

Peak#	Ret. Time (min)	Area%
1	9.90	87.05
2	12.66	12.95
Total		100.00

<Chromatogram>

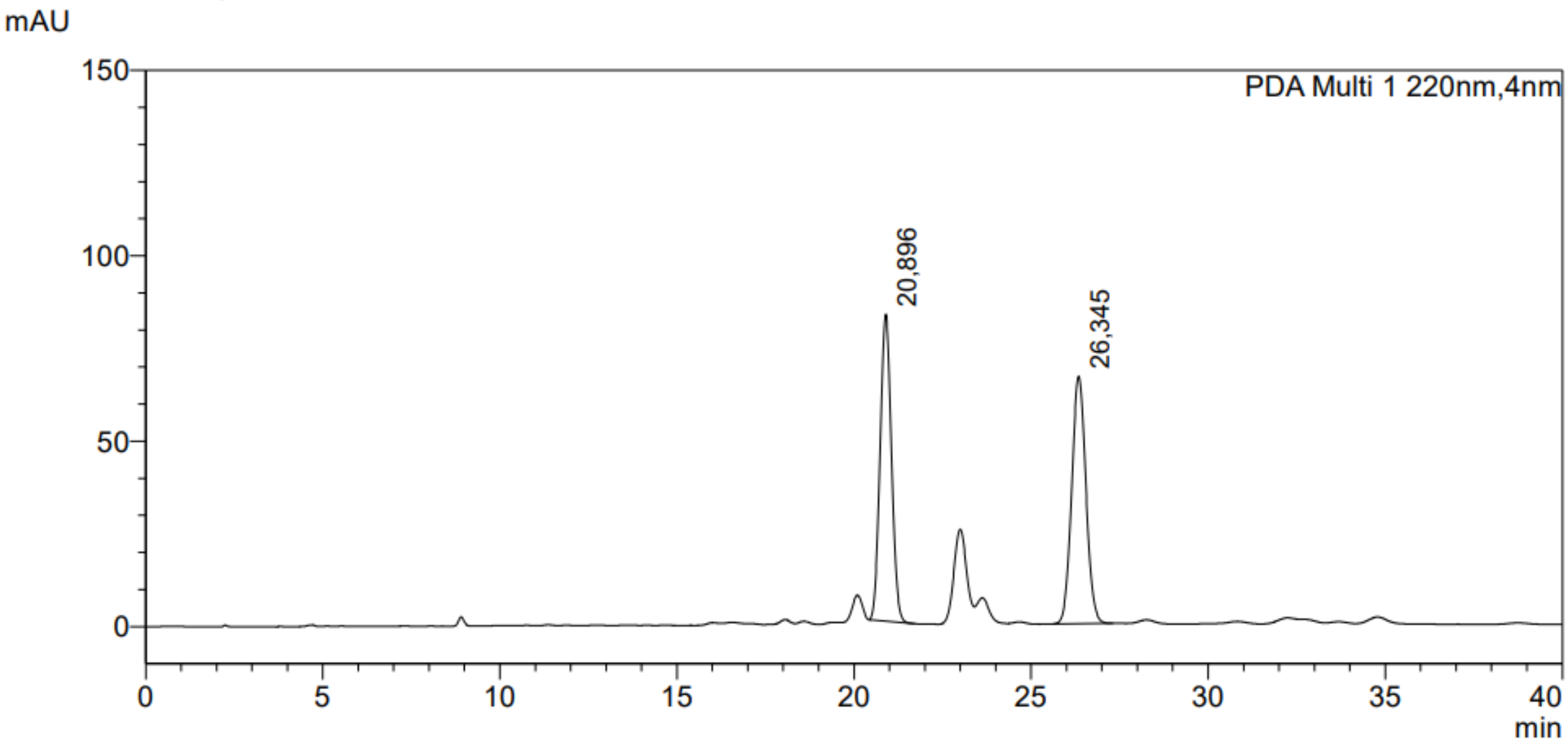

Peak#	Ret. Time (min)	Area%
1	20.90	49.60
2	26.35	50.40
Total		100.00

<Chromatogram>

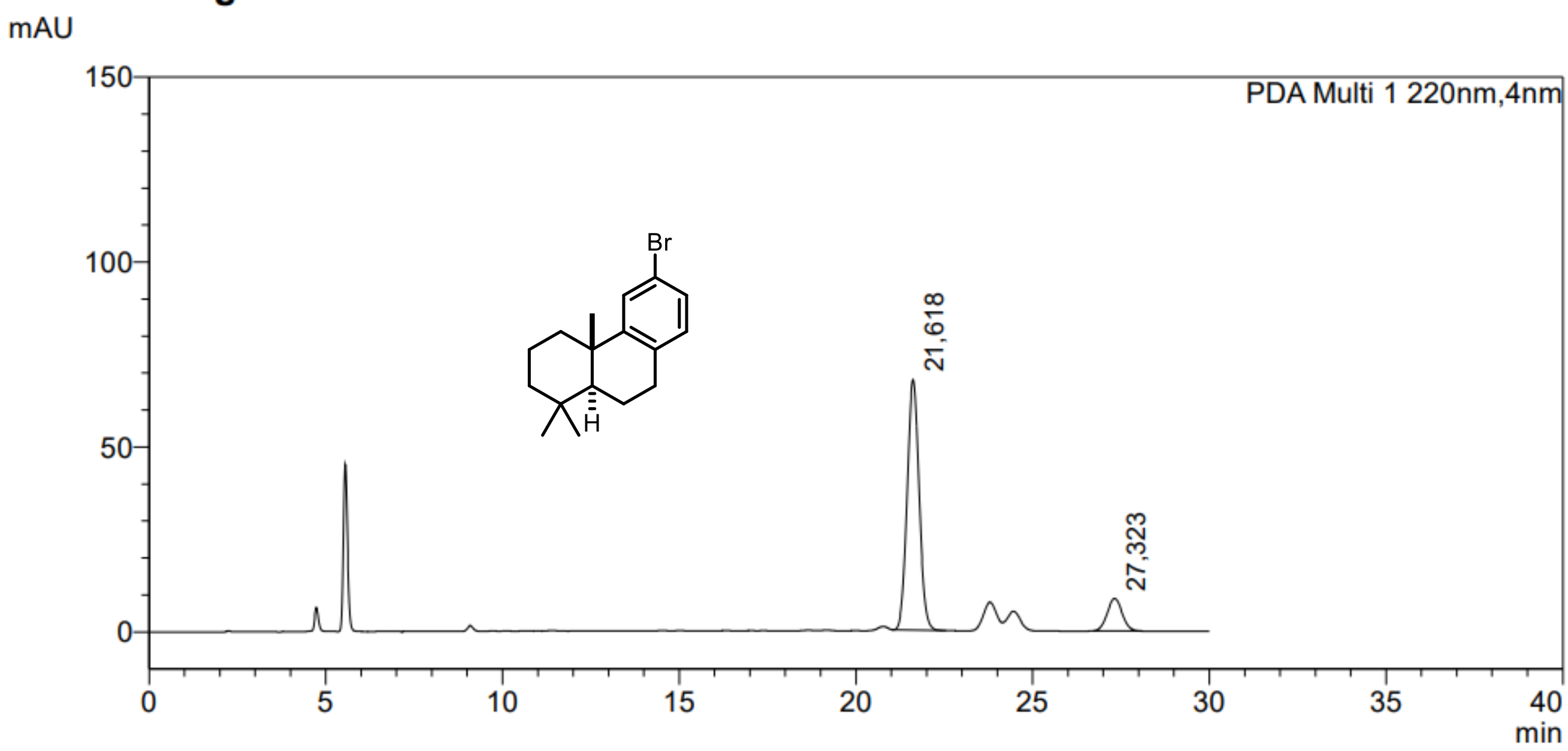

Peak#	Ret. Time (min)	Area%
1	21.62	86.12
2	27.32	13.88
Total		100.00

2D-HPLC separation of rac-11b and 12b

Stacked HPLC chromatograms (achiral stationary phase) of SM **10b**, rac-**11b** and rac-**12b:**

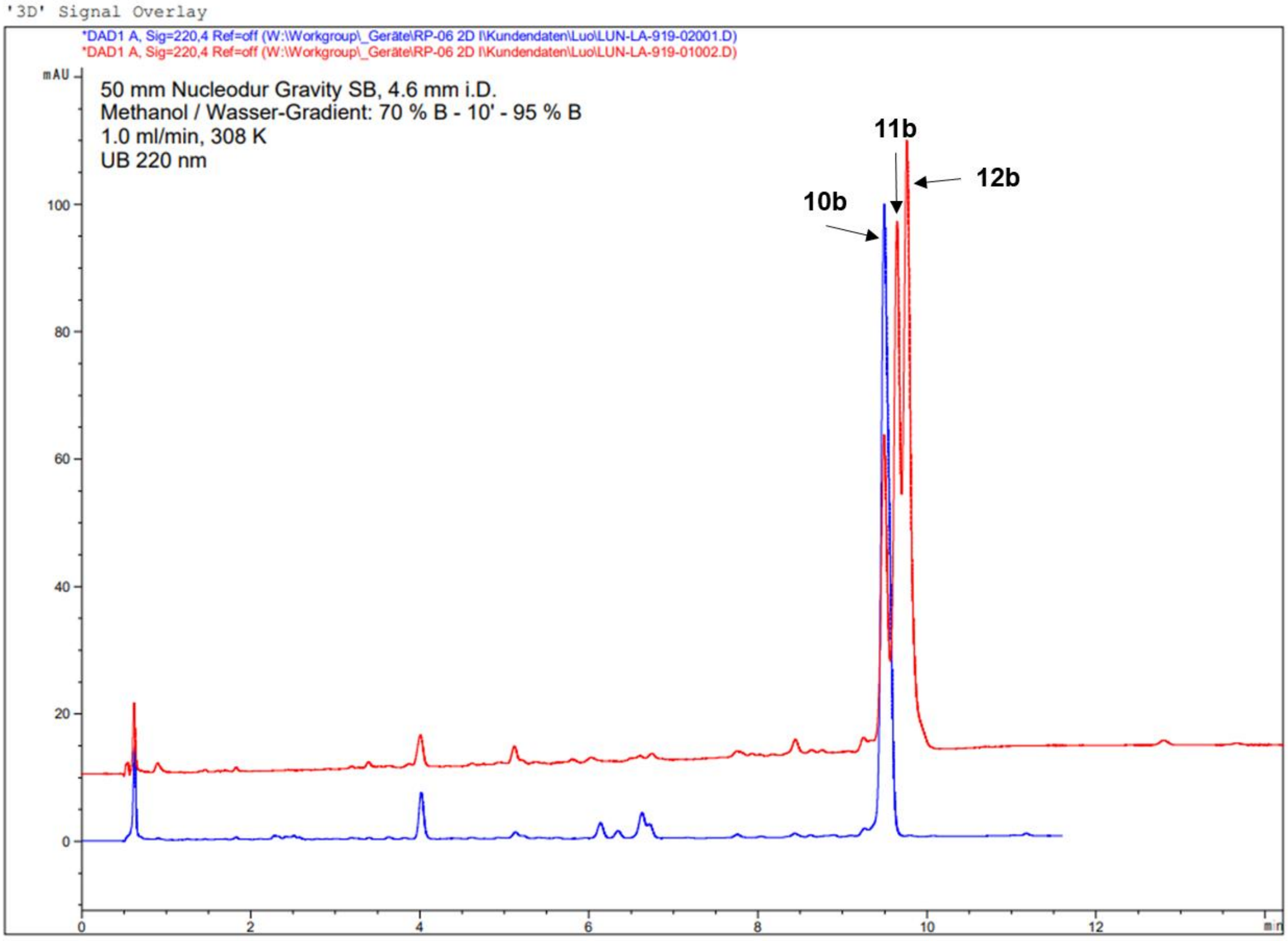

2D-HPLC separation:

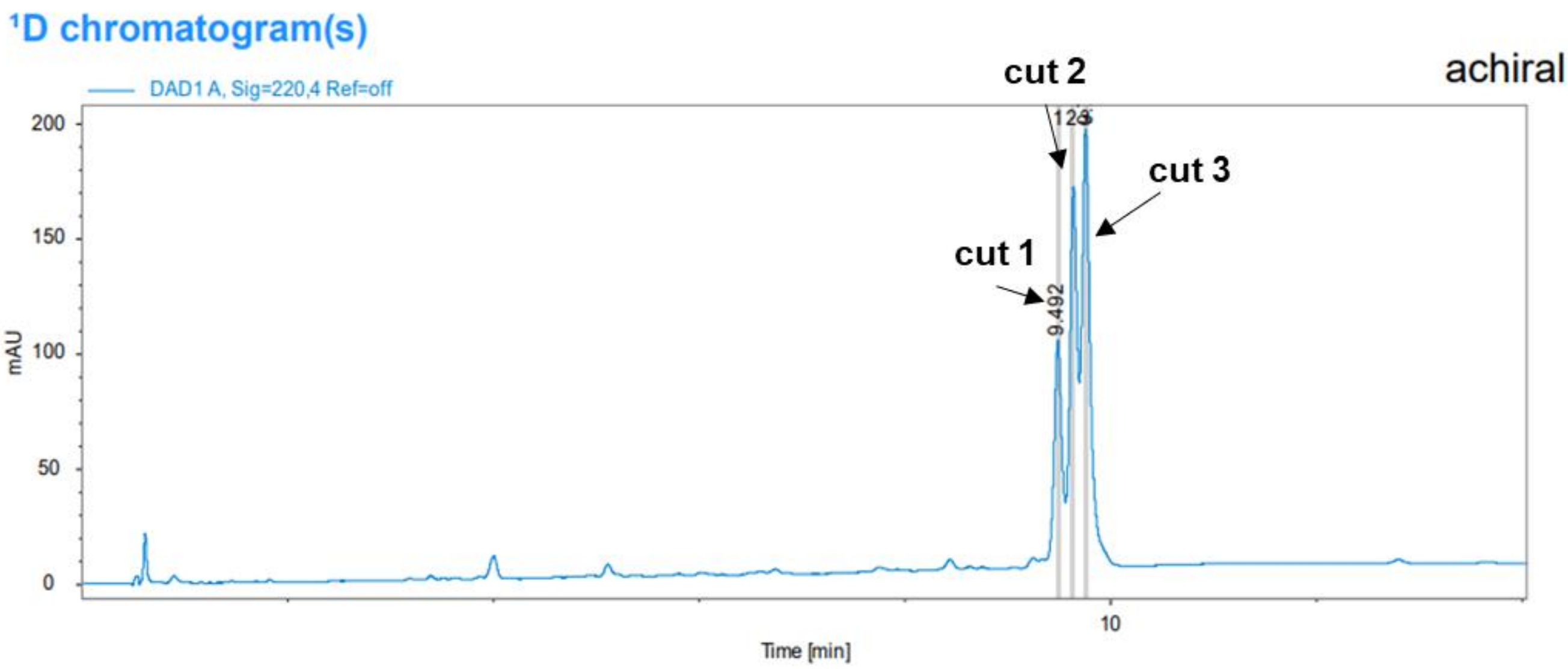

Cut# : 2

alpha isomer

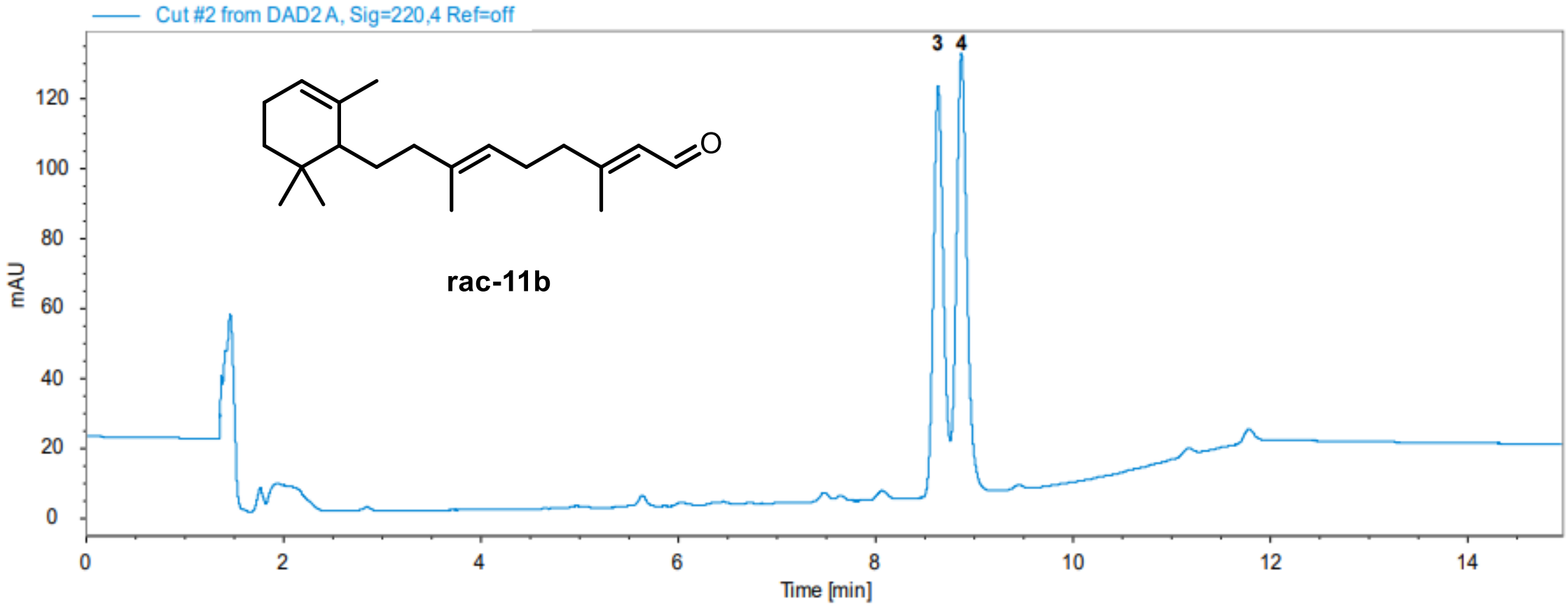

Peak#	Ret. Time (min)	Area%
3	8.64	50.00
4	8.88	50.00
Total		100.00

Cut# : 3

gamma isomer

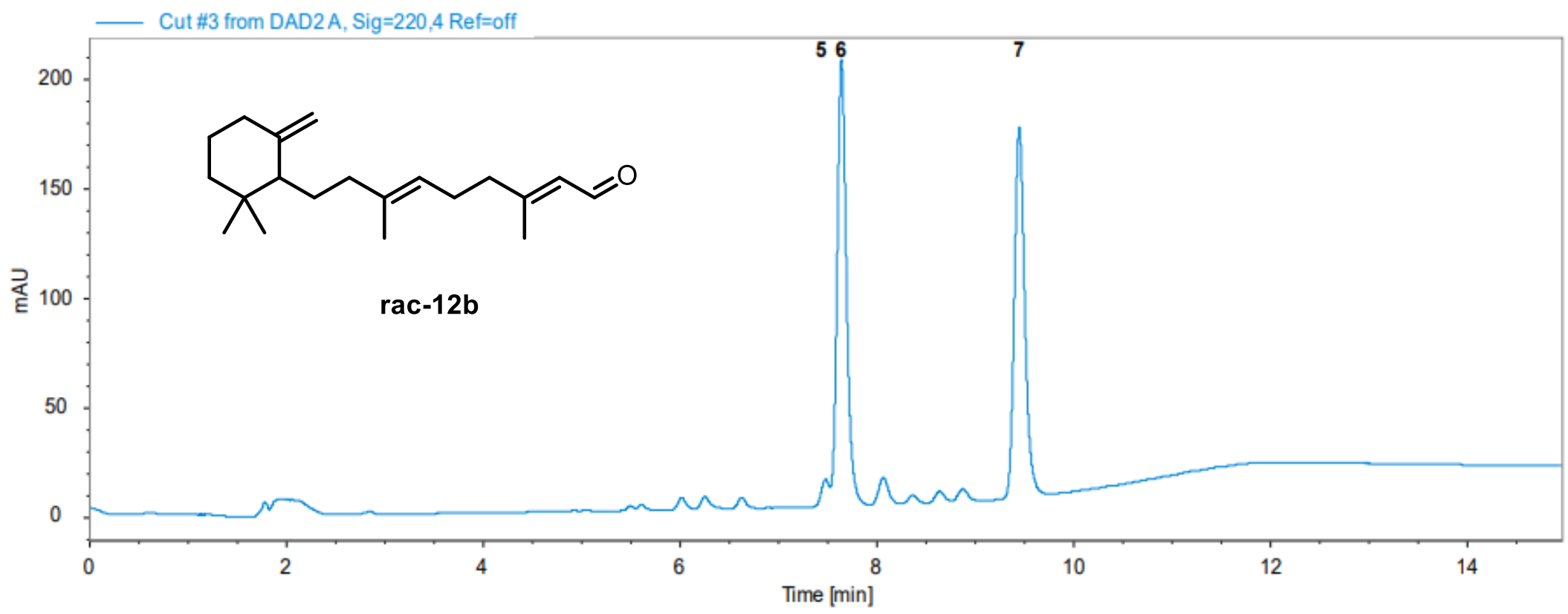

Peak#	Ret. Time (min)	Area%
6	7.65	50.00
7	9.45	50.00
Total		100.00

2D-HPLC separation of 11b

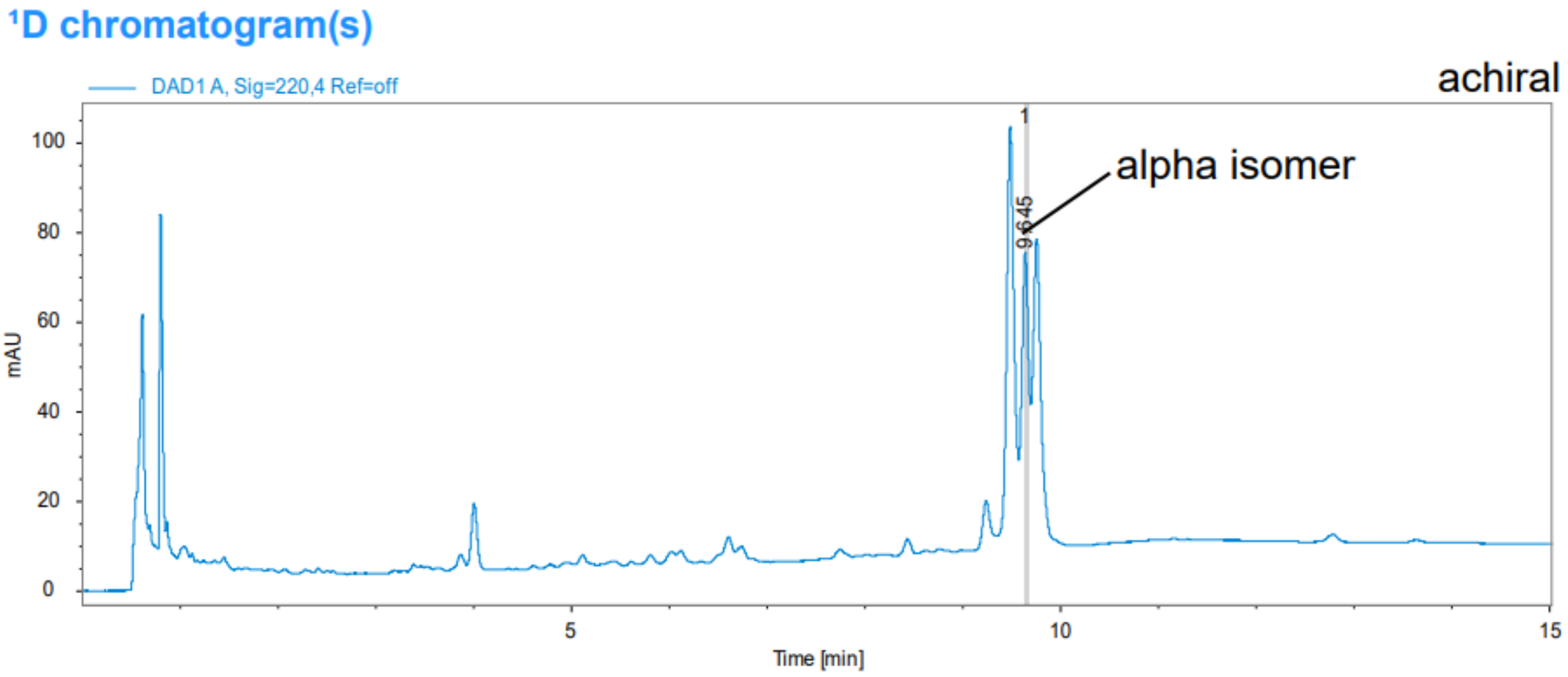

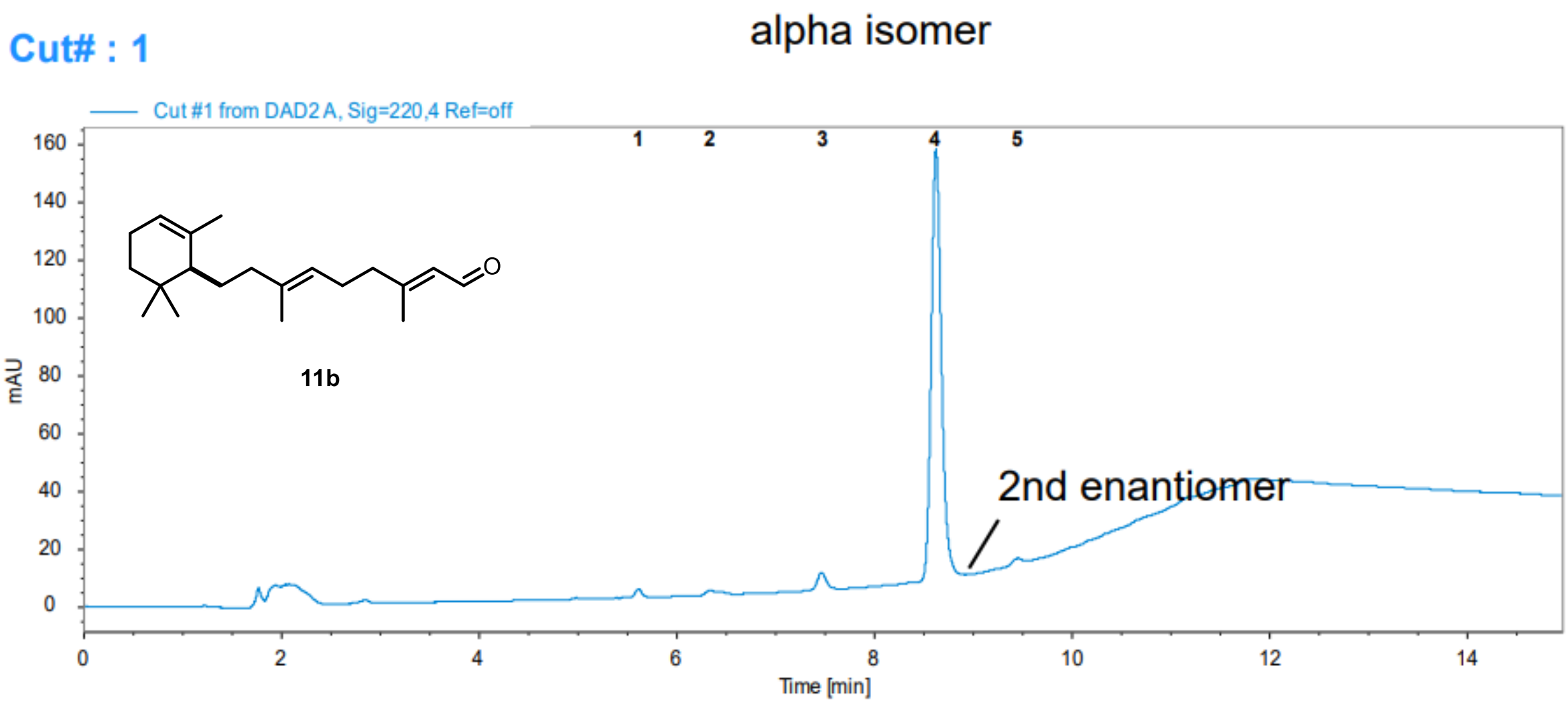

Peak#	Ret. Time (min)	Area%
4	8.63	>99.00
Total		100.00

^{1}D chromatogram(s)

achiral

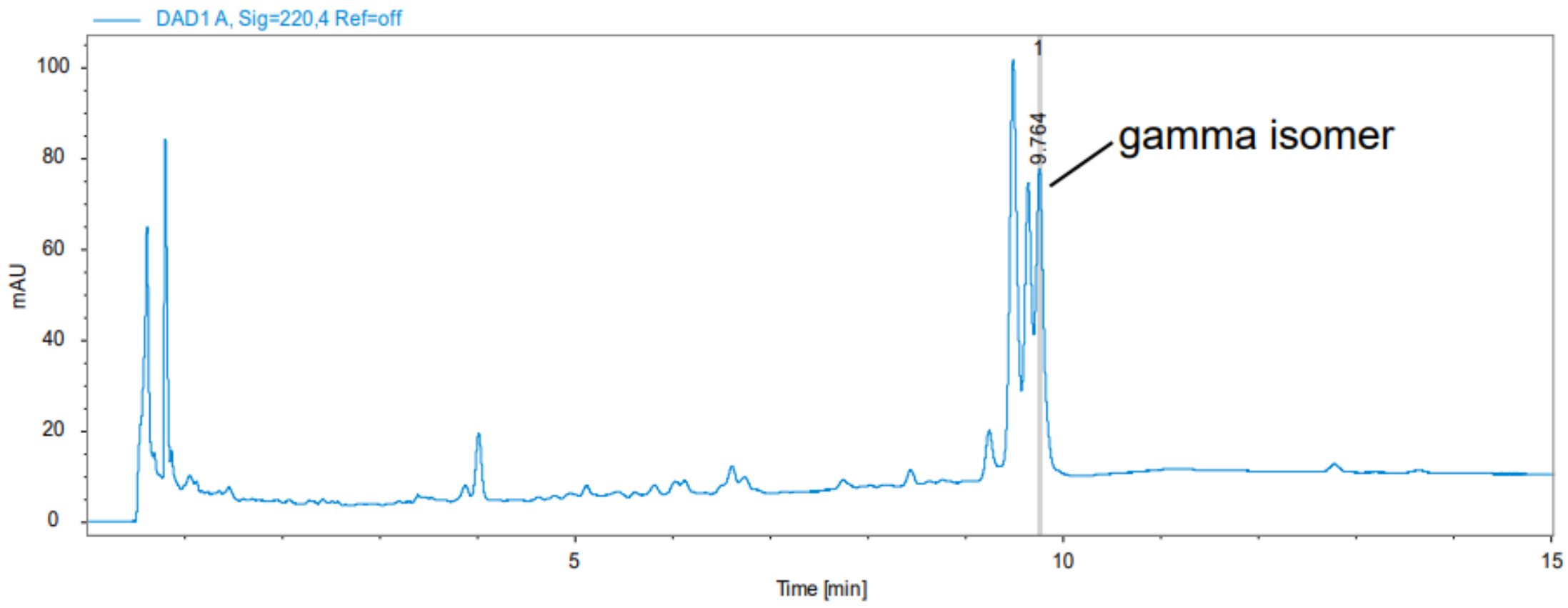

Cut# : 1

gamma isomer

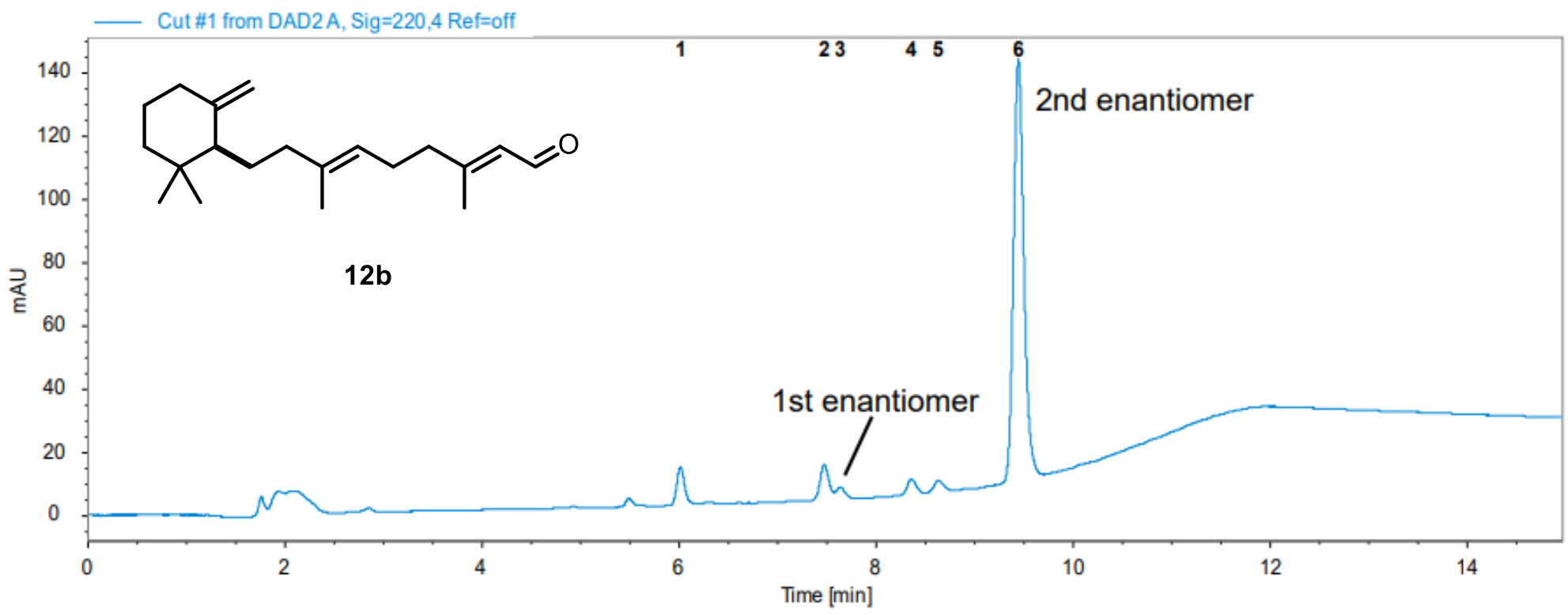

Peak#	Ret. Time (min)	Area%
3	7.64	2.40
6	9.45	97.60
Total		100.00

2D-HPLC separation of *S*-11c (product obtained using (*S*,*S*)-IDPi 5d as the catalyst.)

[1]D chromatogram(s)

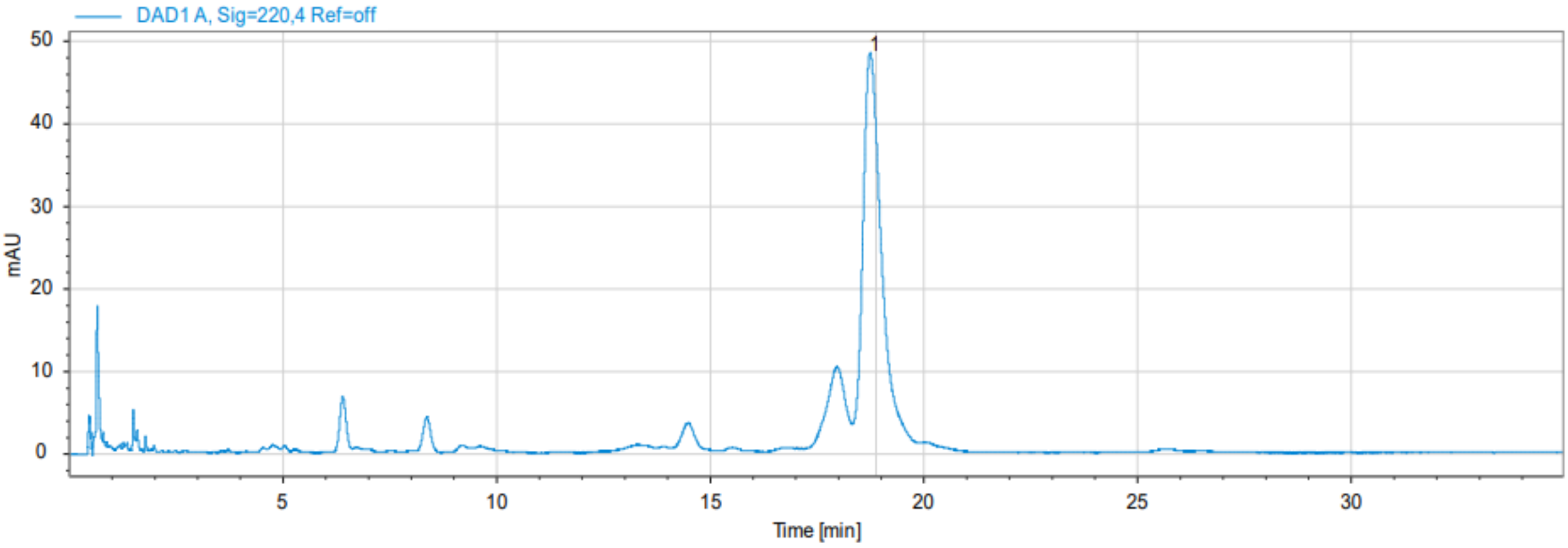

Cut# : 1

chiral

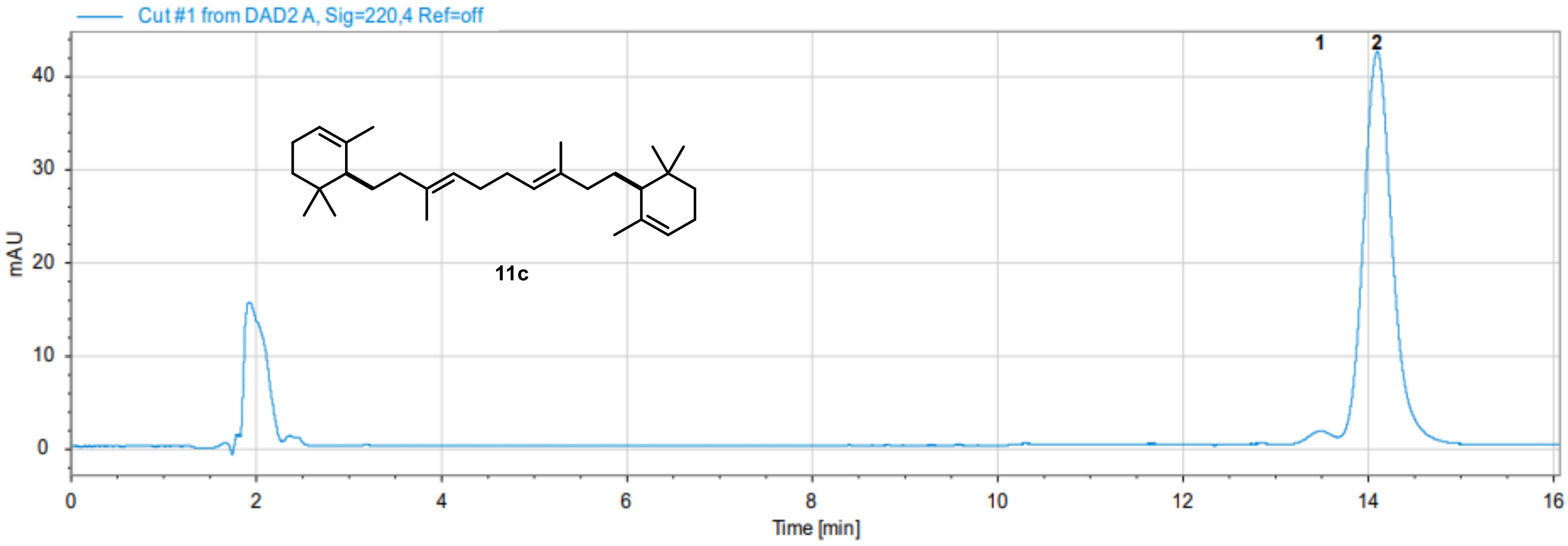

Peak#	Ret. Time (min)	Area%
1	13.49	2.65
2	14.10	97.35 (*S*-product)
Total		100.00

2D-HPLC separation of *R*-11c (product obtained using (*R*,*R*)-IDPi 5d as the catalyst.)

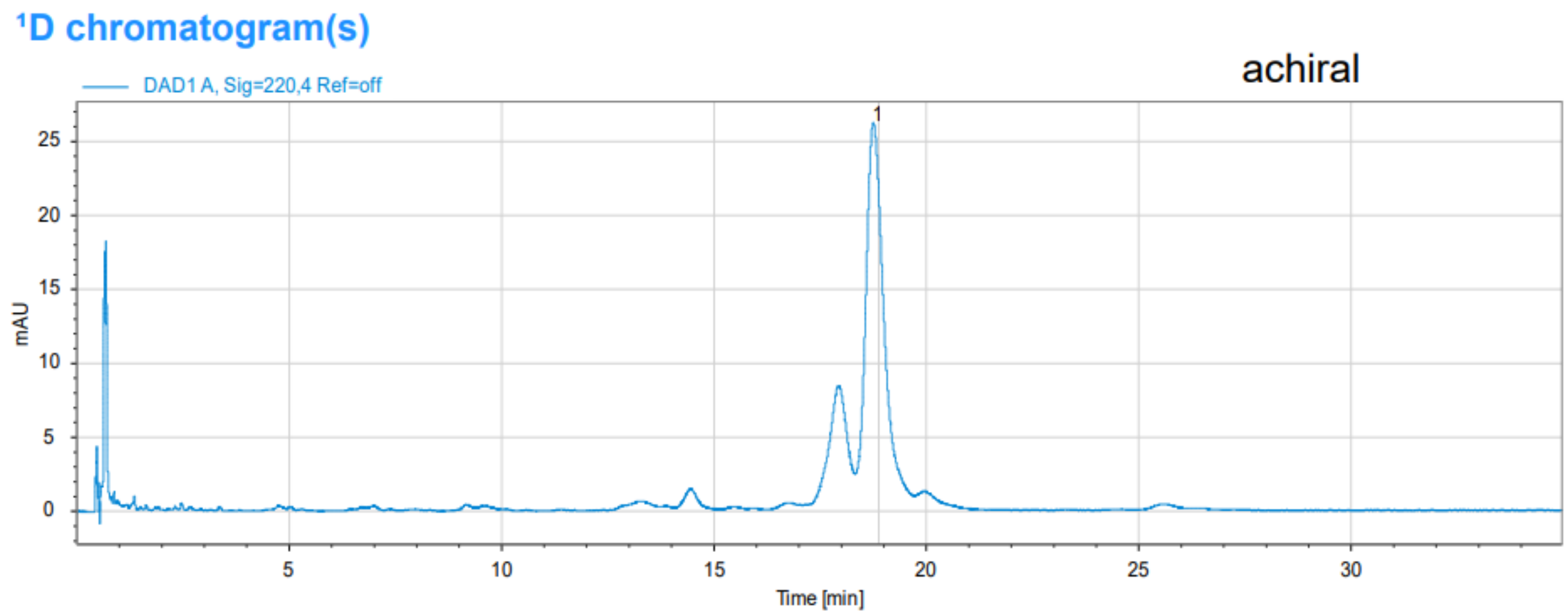

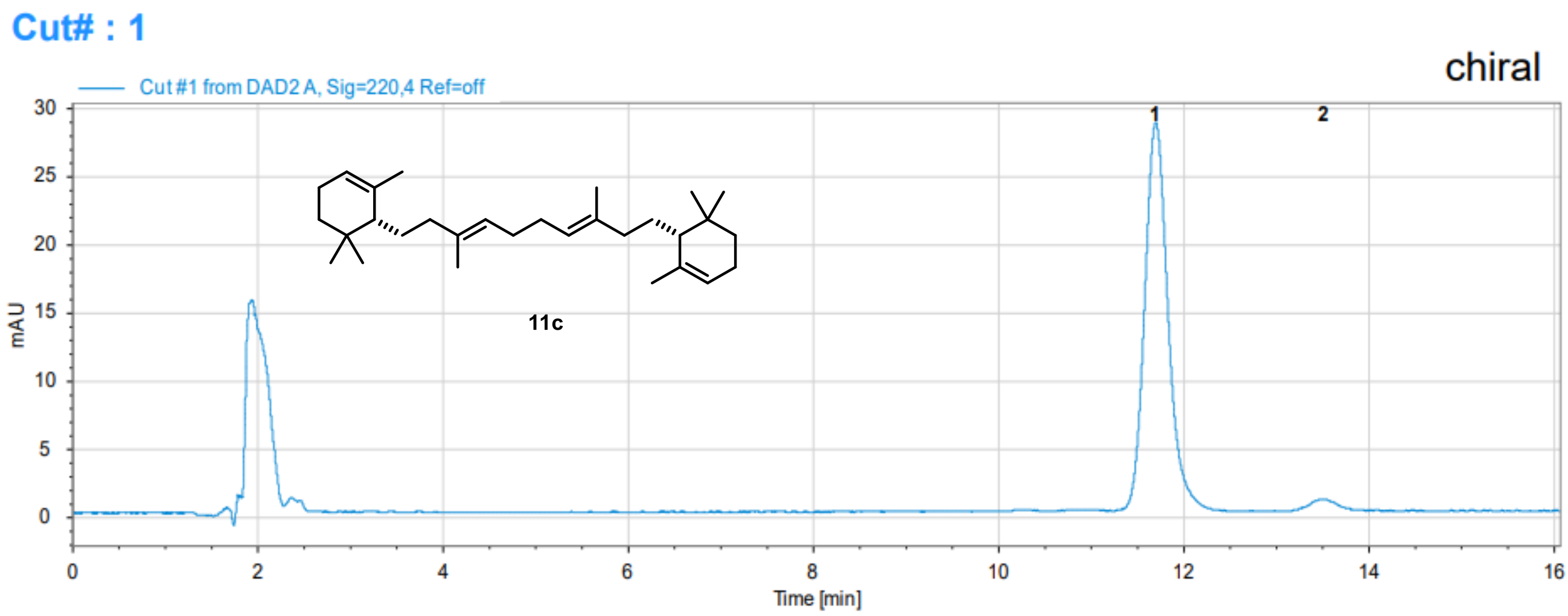

Peak#	Ret. Time (min)	Area%
1	11.70	96.56 (*R*-product)
2	13.50	3.44
Total		100.00

Overlap of HPLC traces of the *S*-11c (from (*S*,*S*)-IDPi 5d) and the *R*-11c (from (*R*,*R*)-IDPi 5d).

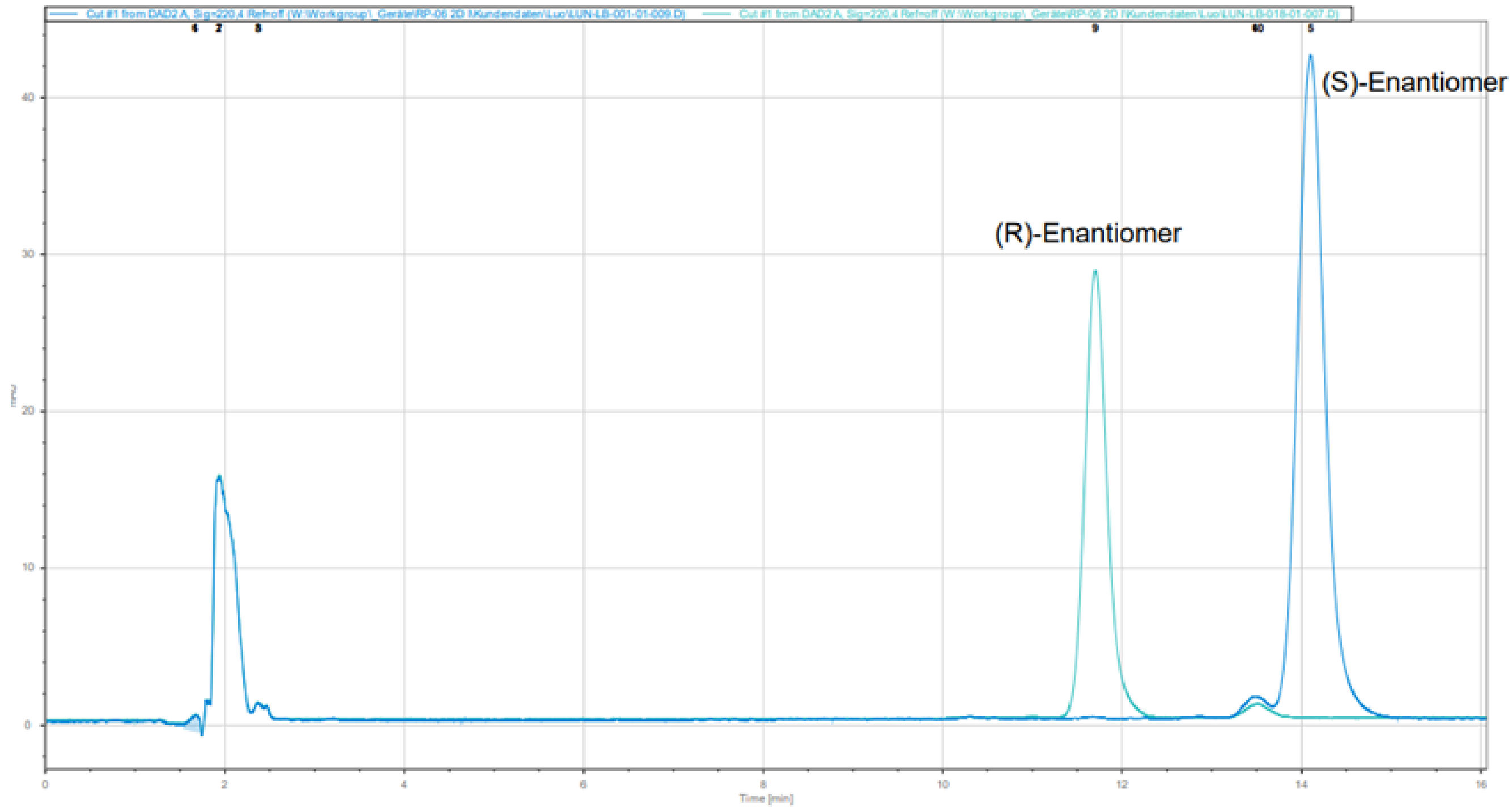

2D-HPLC separation of *Rac*-11d

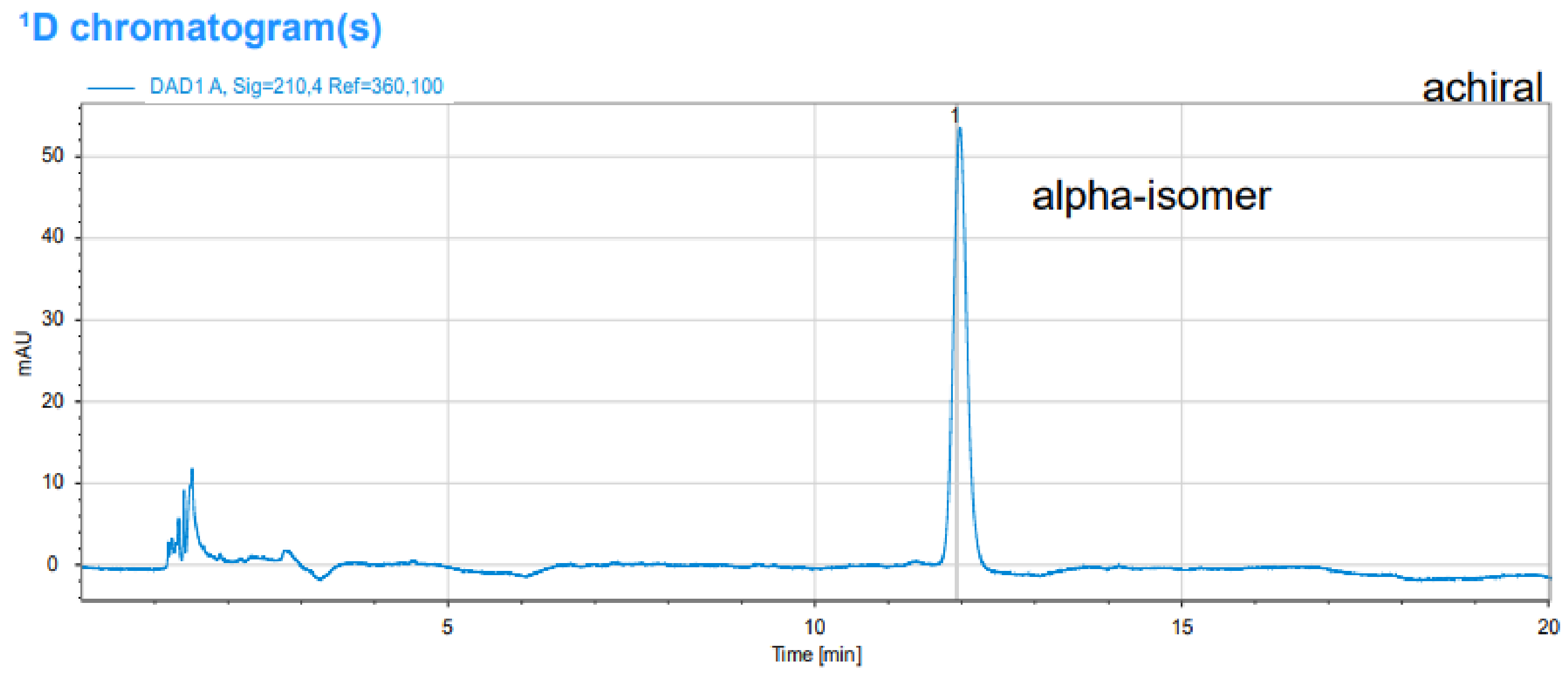

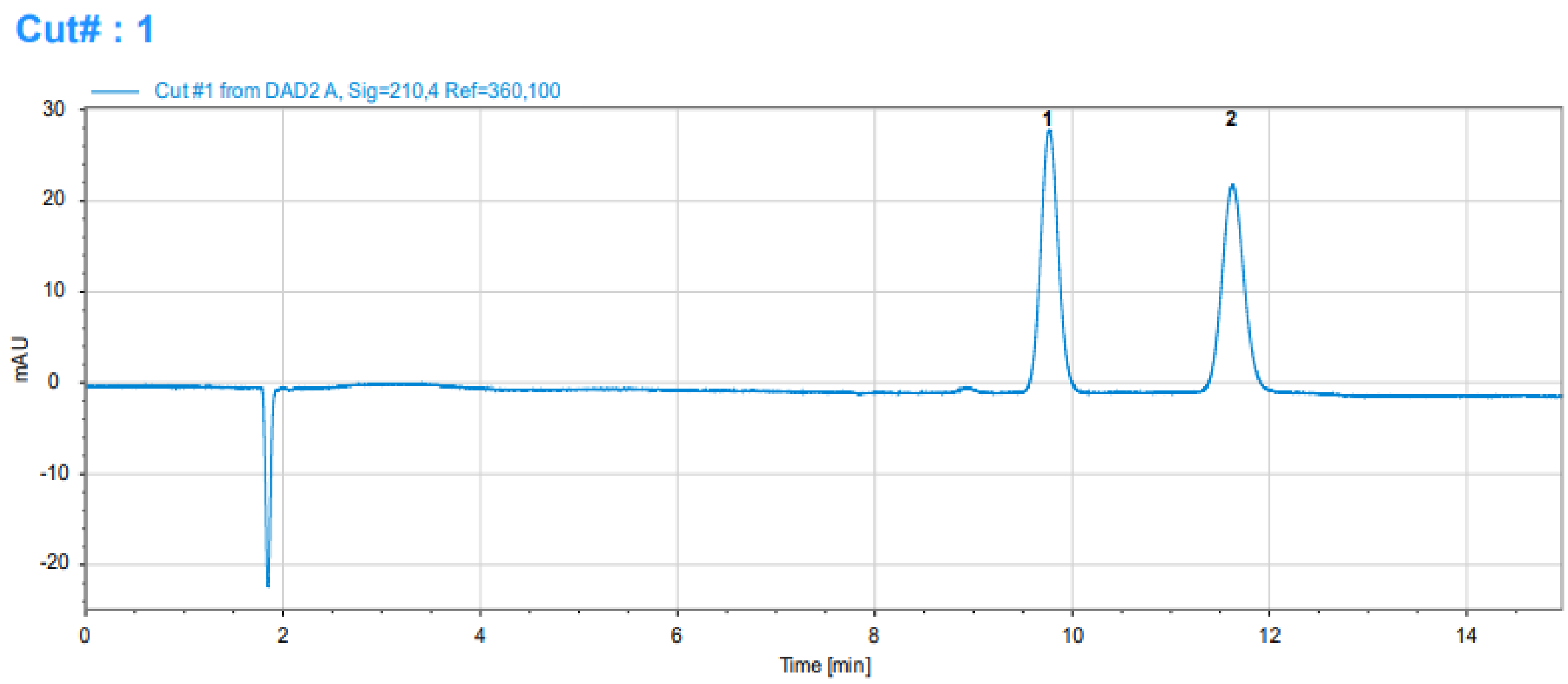

Peak#	Ret. Time (min)	Area%
1	9.77	50.00
2	11.62	50.00
Total		100.00

2D-HPLC separation of *S*-11d

¹D chromatogram(s)

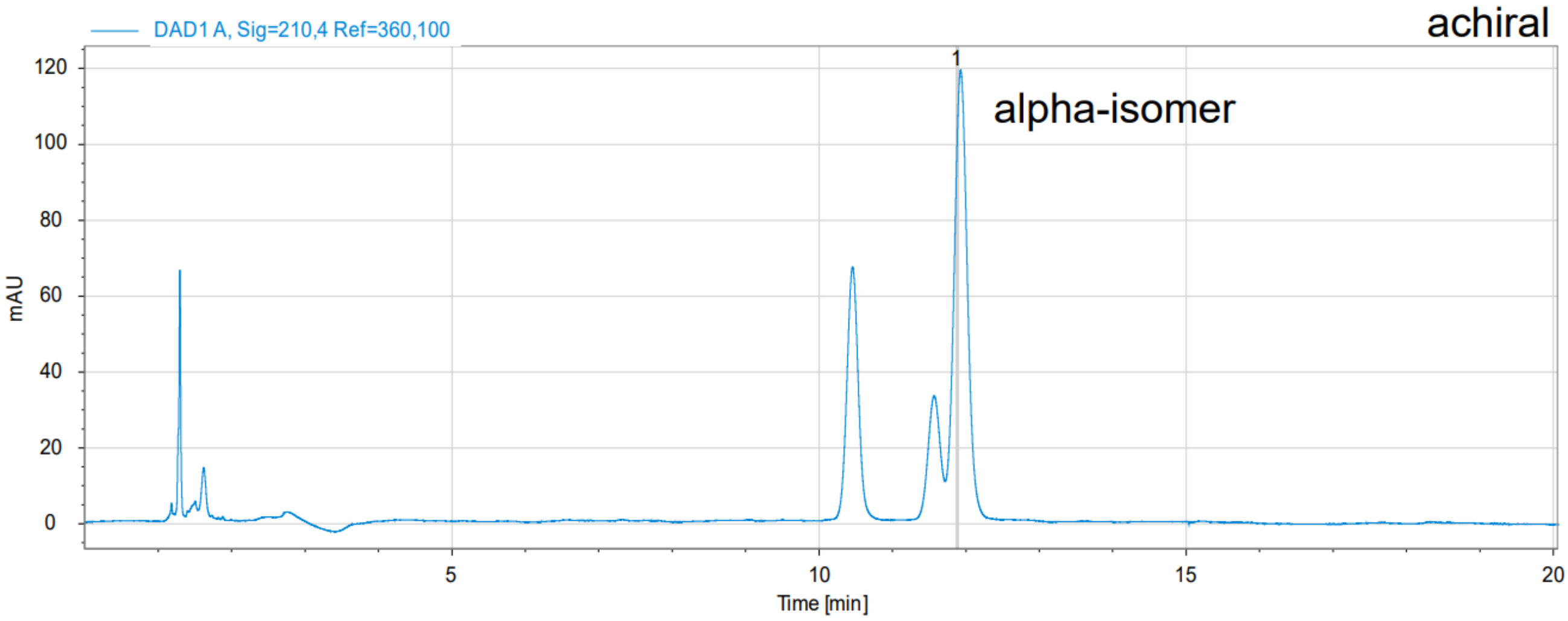

Cut# : 1

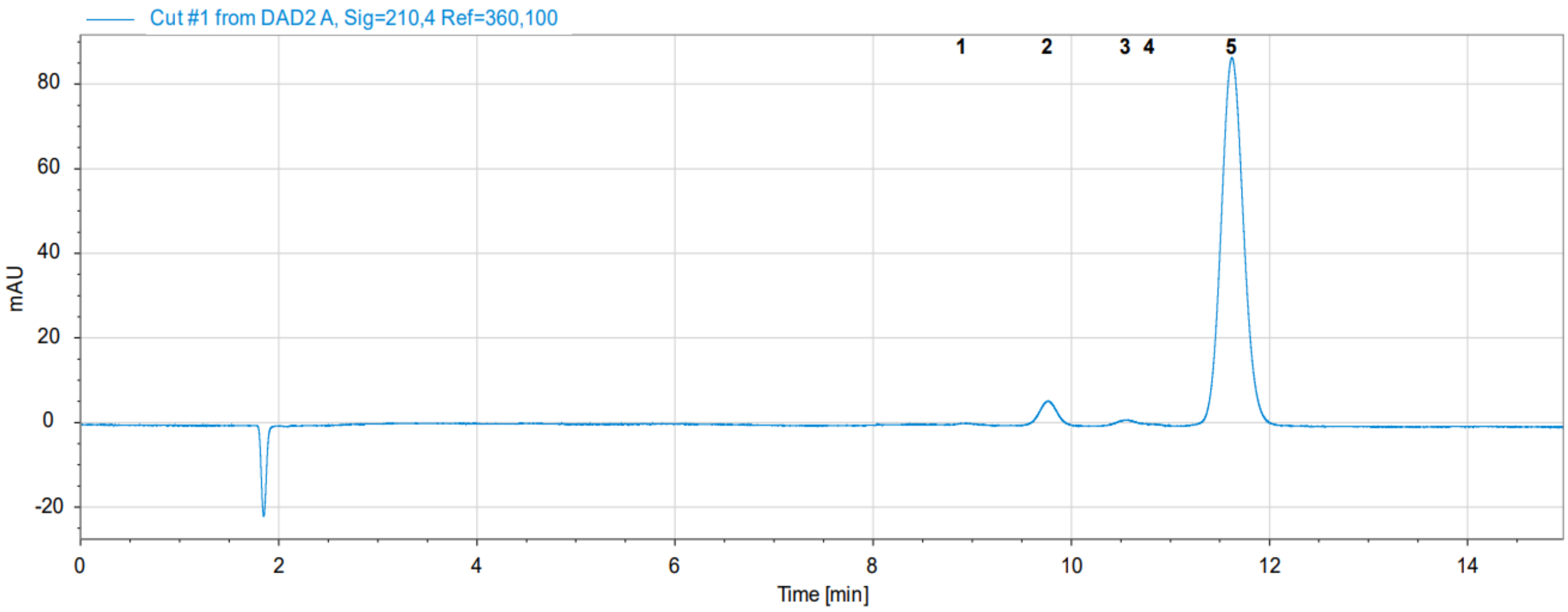

Peak#	Ret. Time (min)	Area%
2	9.77	5.10
5	11.62	94.90
Total		100.00

2D-HPLC separation of *Rac*-12d

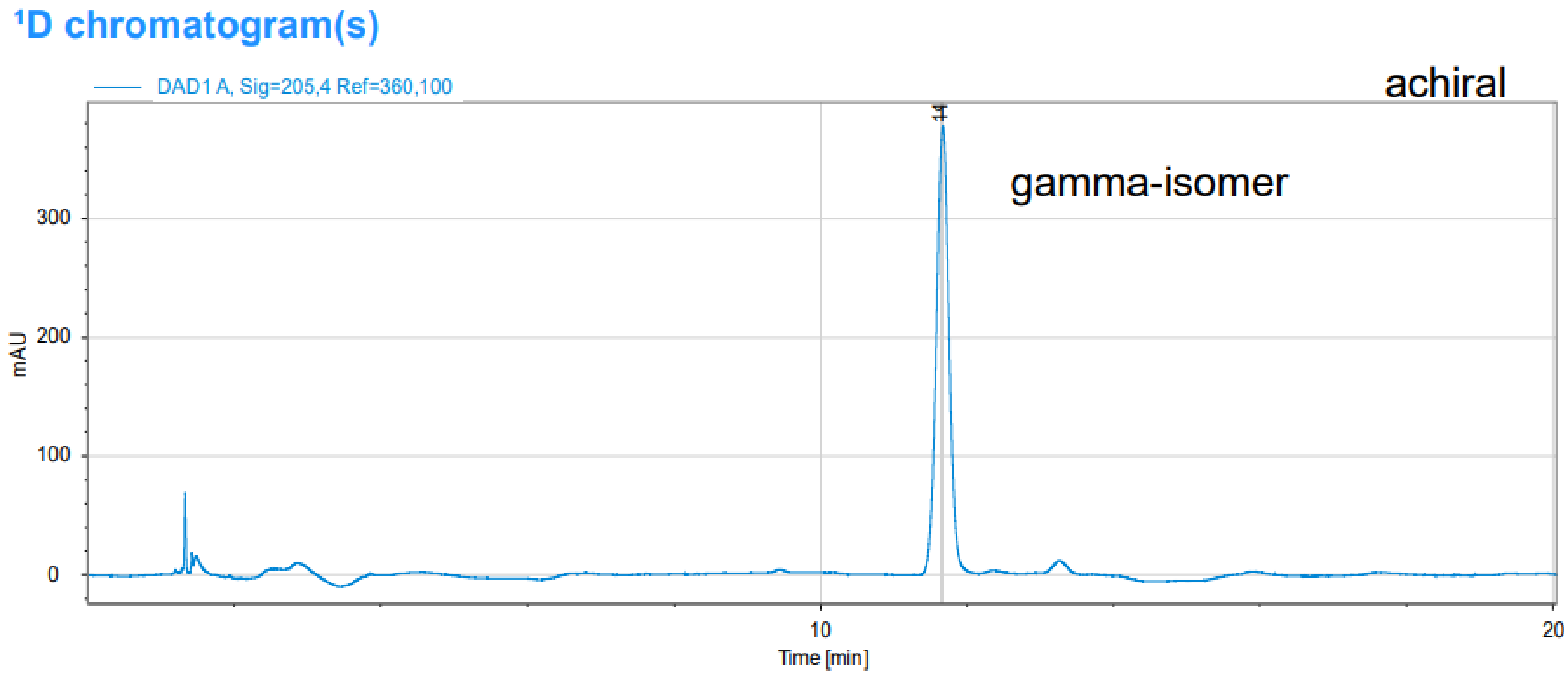

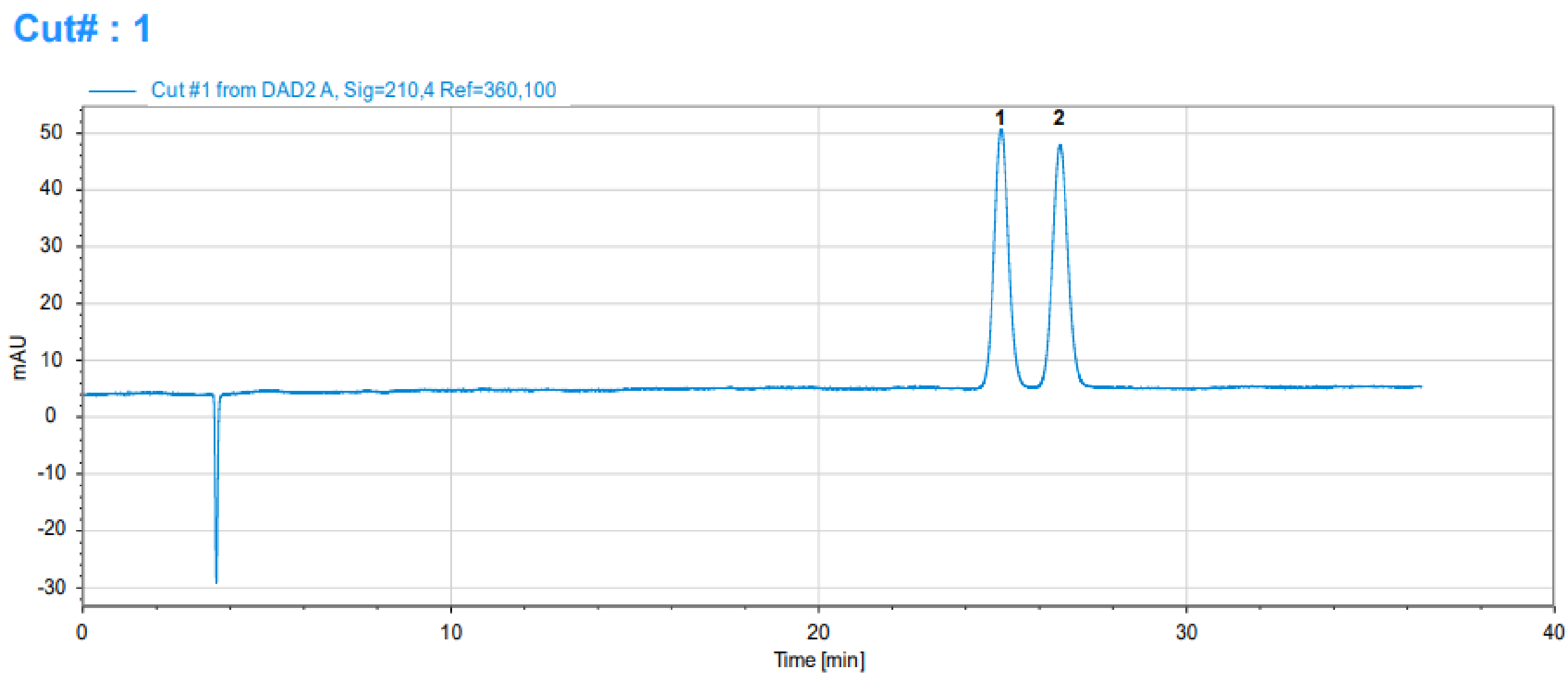

Peak#	Ret. Time (min)	Area%
1	24.96	50.00
2	26.56	50.00
Total		100.00

2D-HPLC separation of *S*-12d

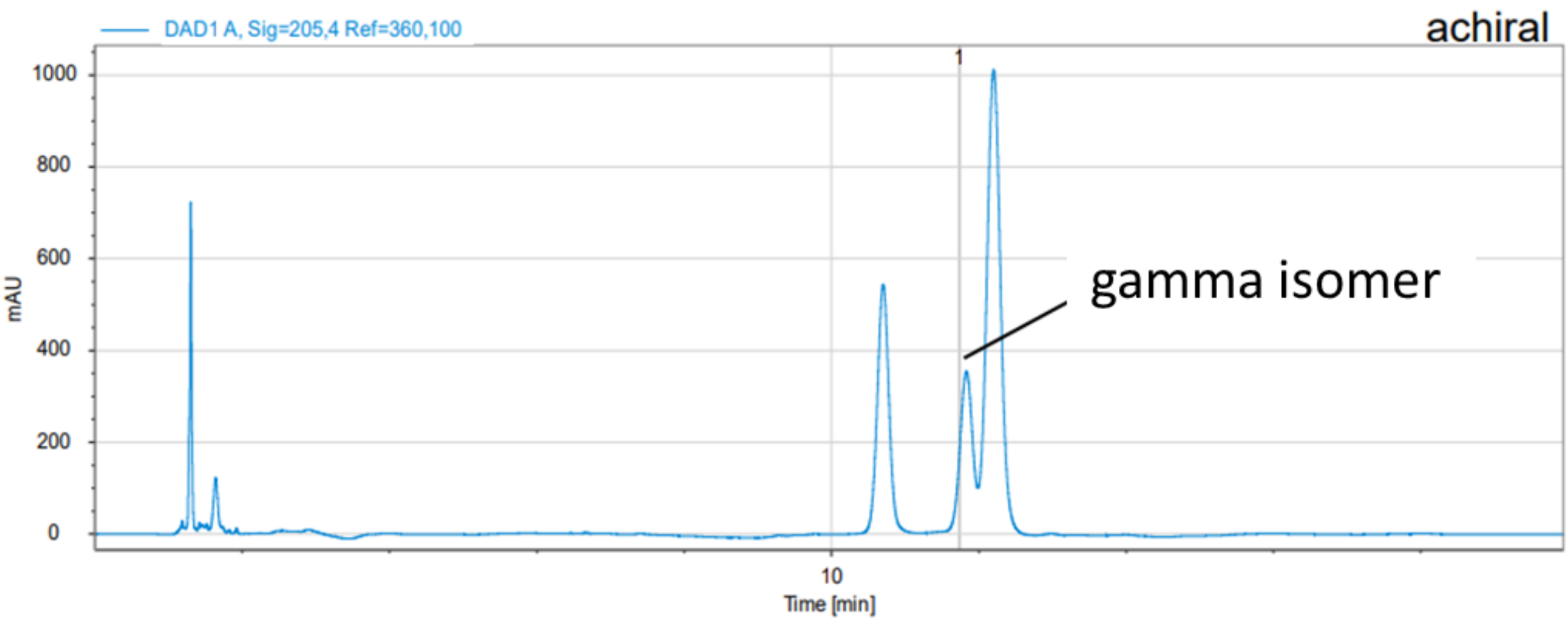

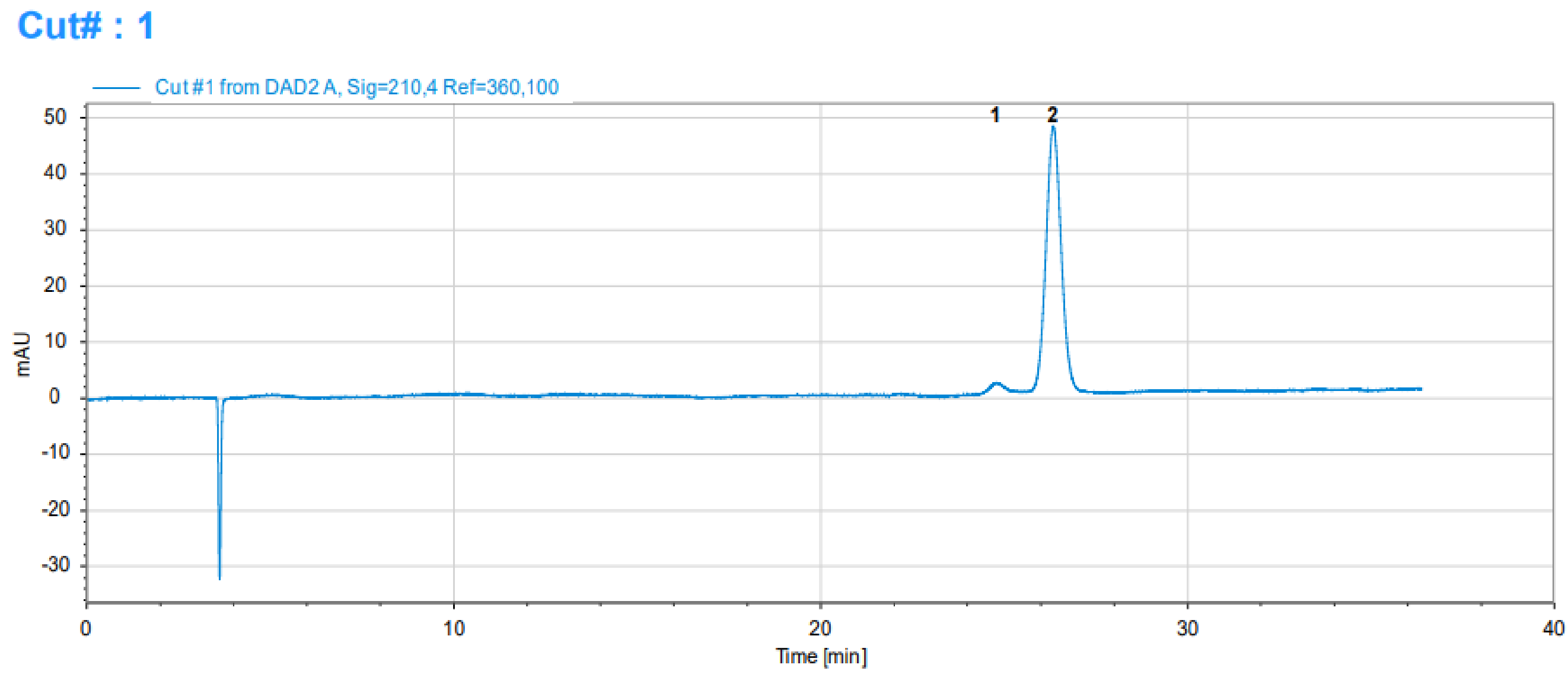

Peak#	Ret. Time (min)	Area%
1	24.78	3.73
2	26.34	96.27
Total		100.00

11. ^{1}H, ^{13}C, ^{31}P and ^{19}F NMR spectra of substrates and products

^{1}H NMR spectrum of **1a**

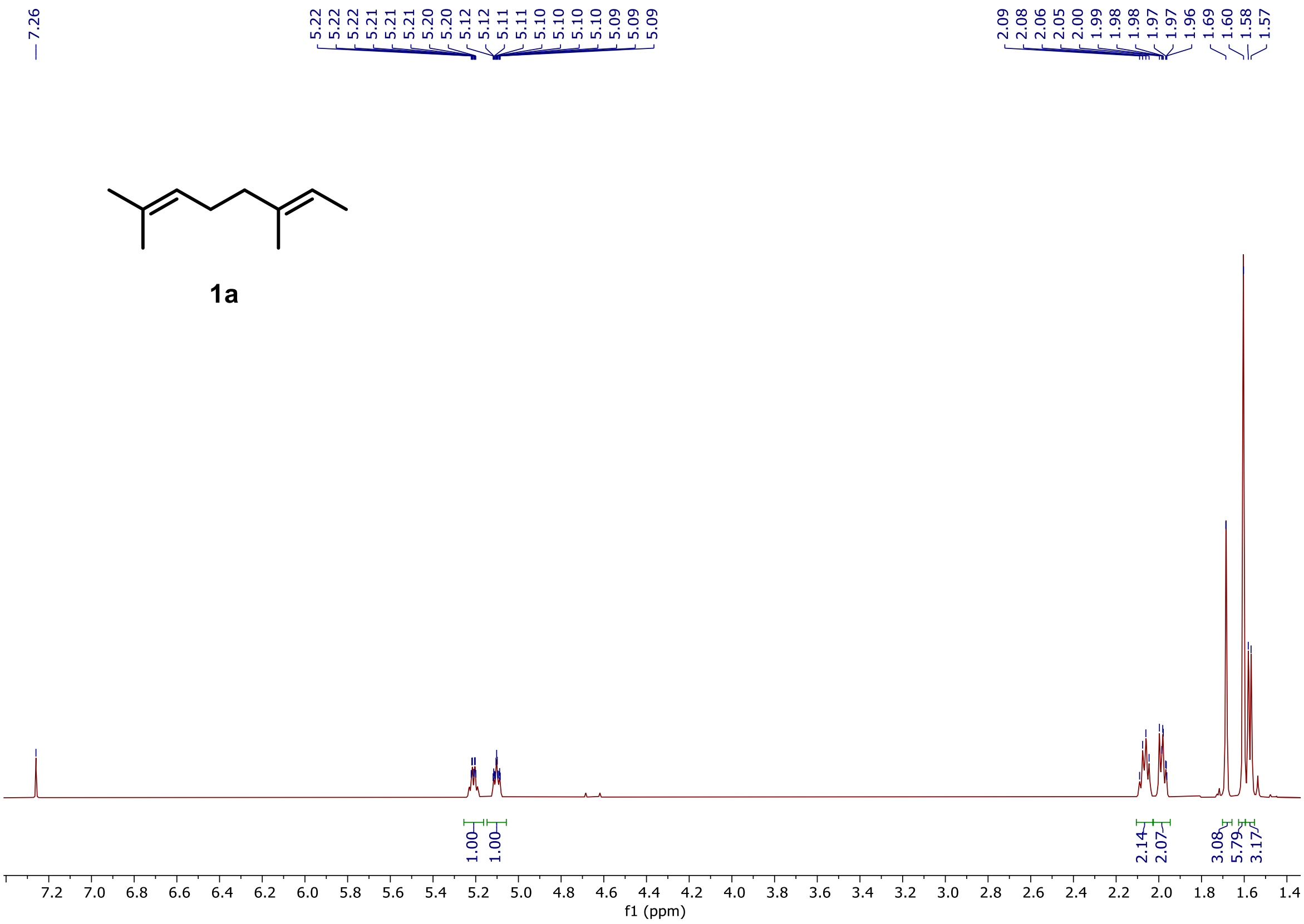

^{13}C NMR spectrum of **1a**

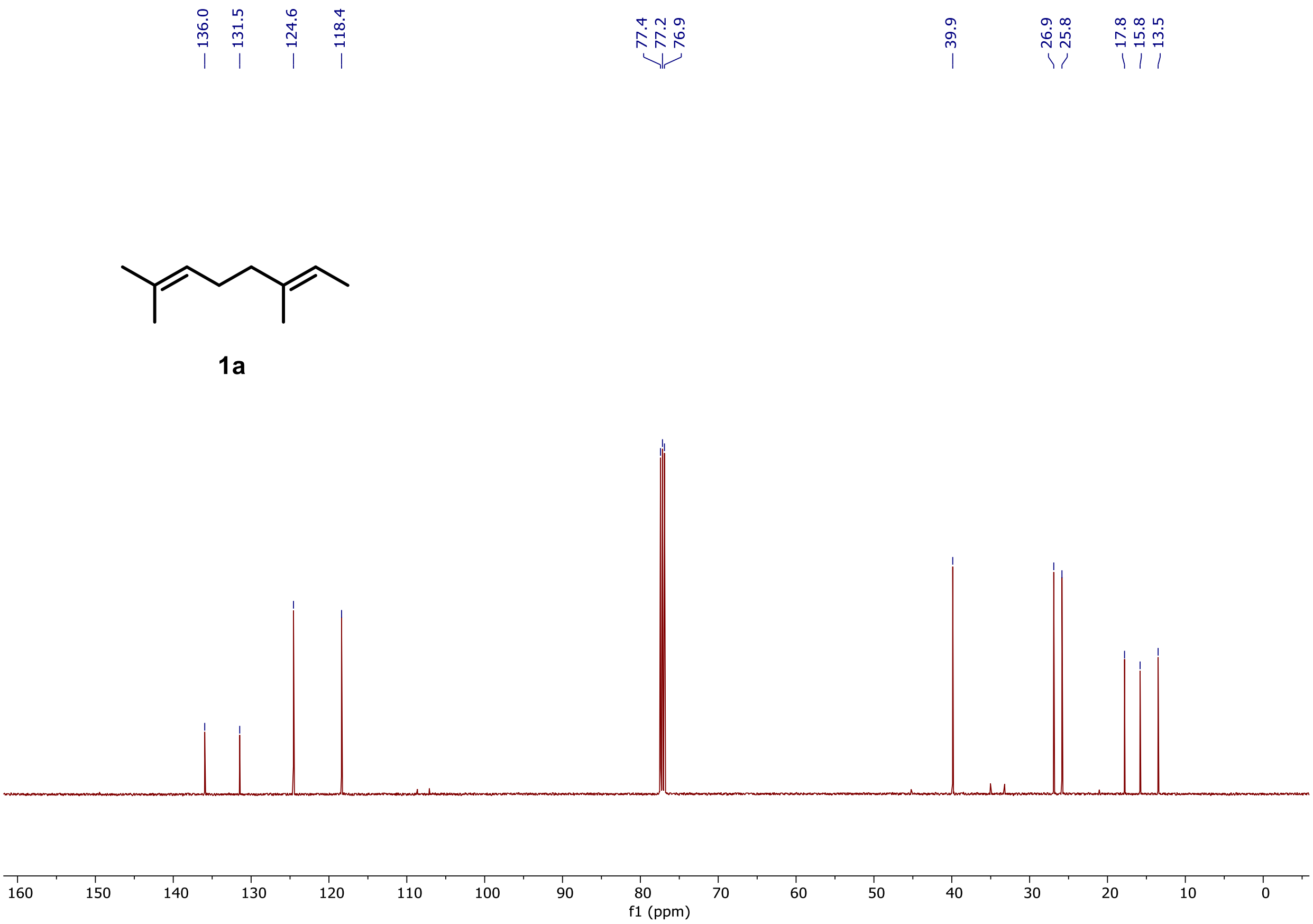

^{1}H NMR spectrum of **1b**

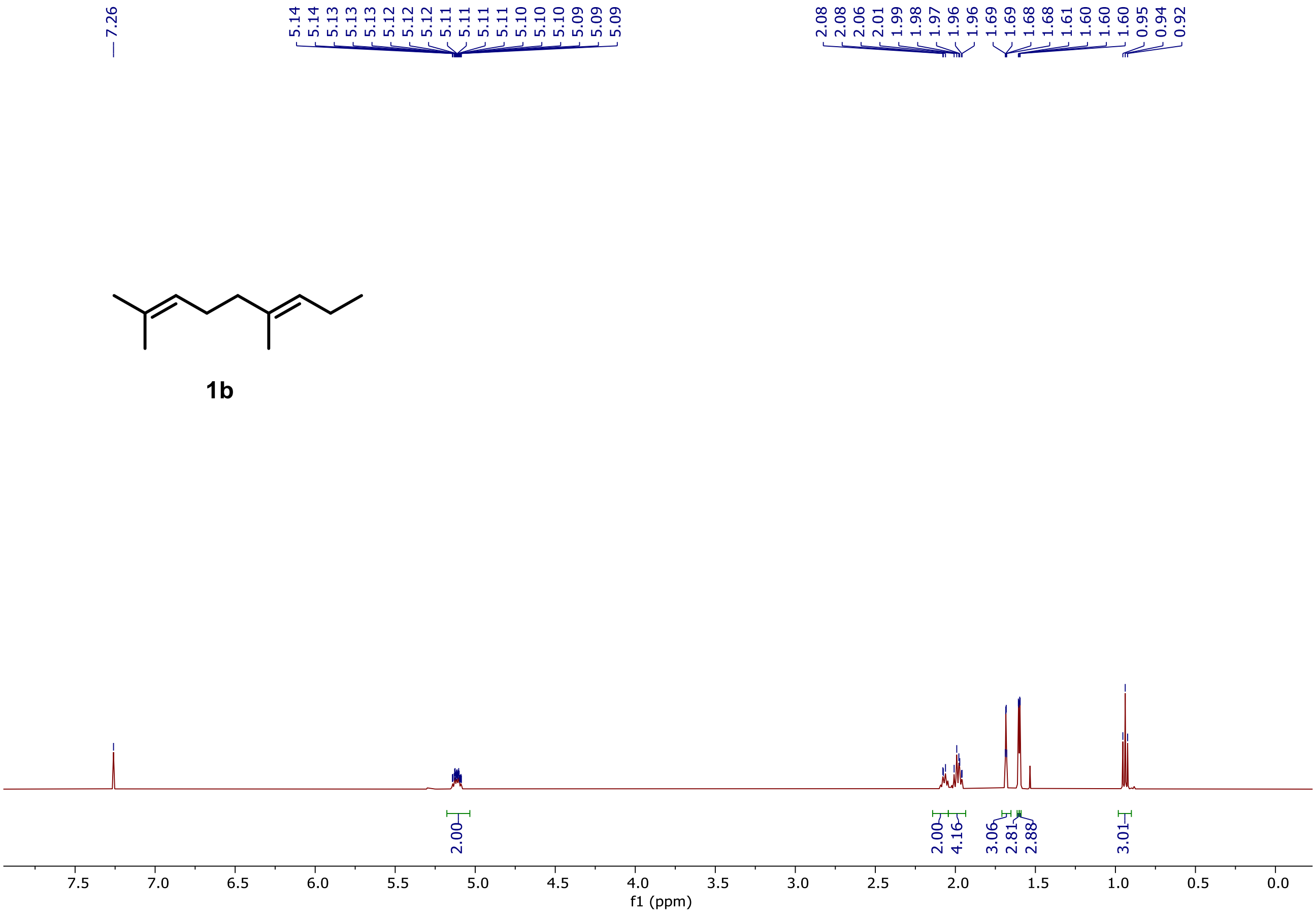

1b

^{13}C NMR spectrum of **1b**

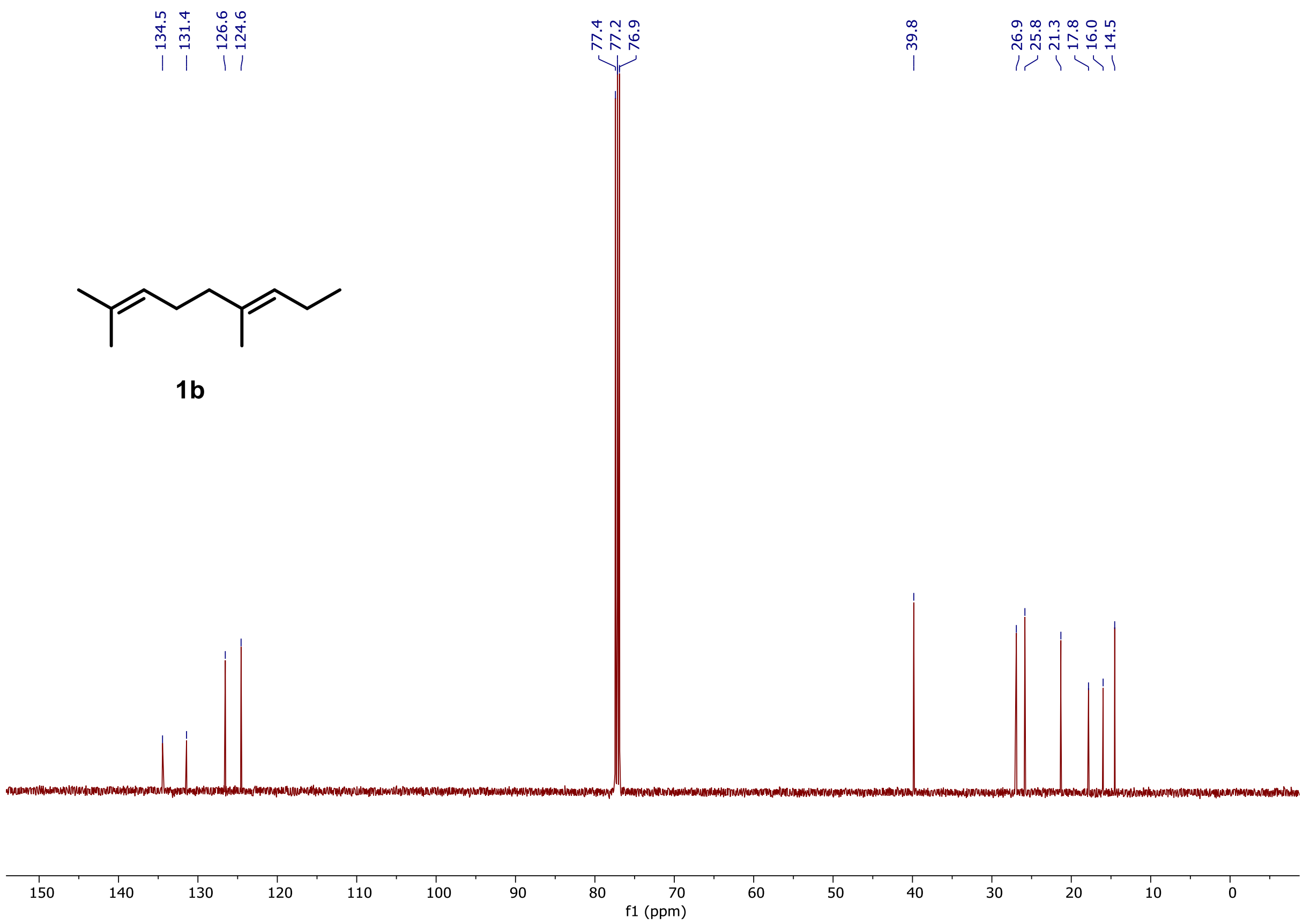

1b

^{1}H NMR spectrum of **1c**

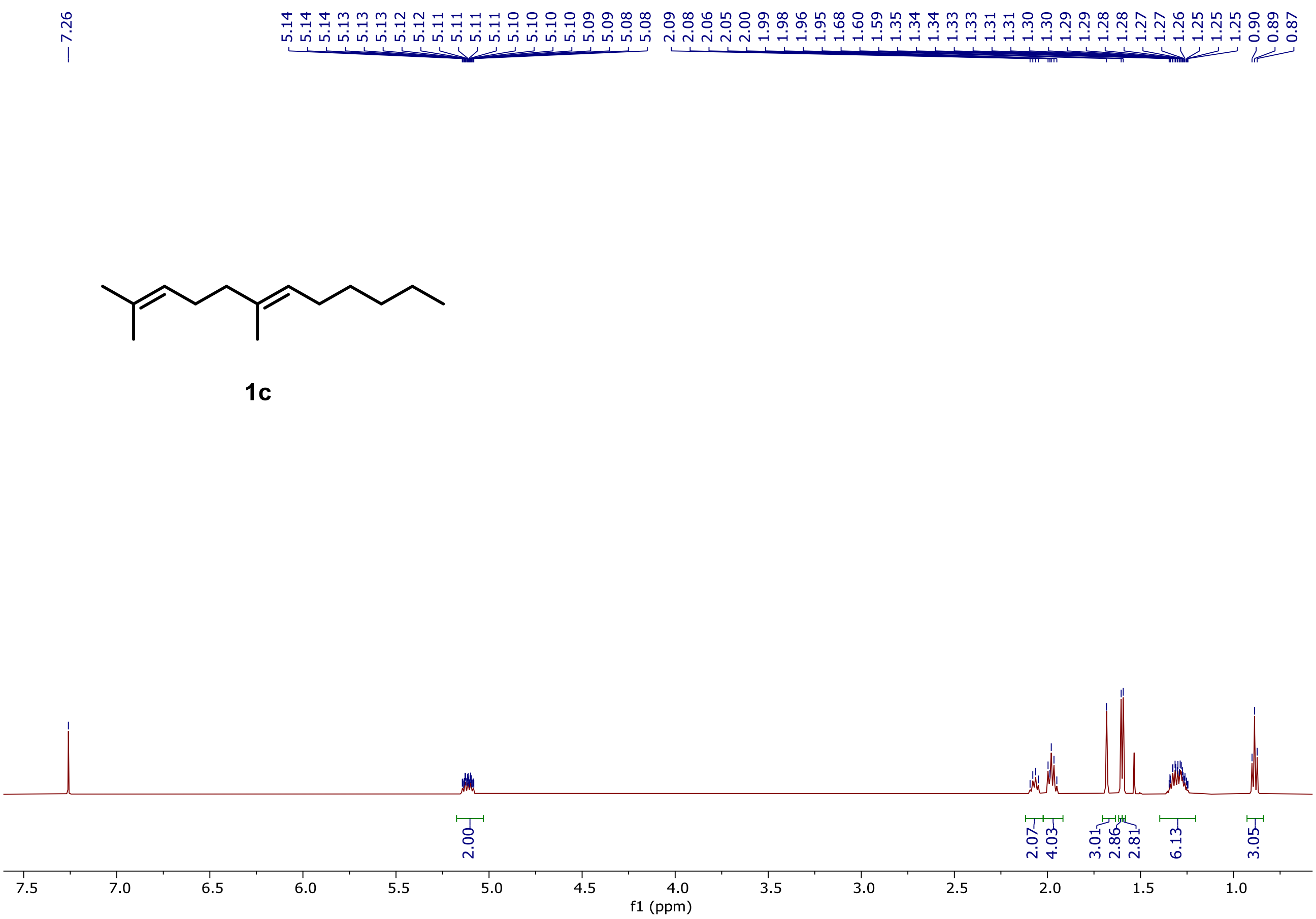

^{13}C NMR spectrum of **1c**

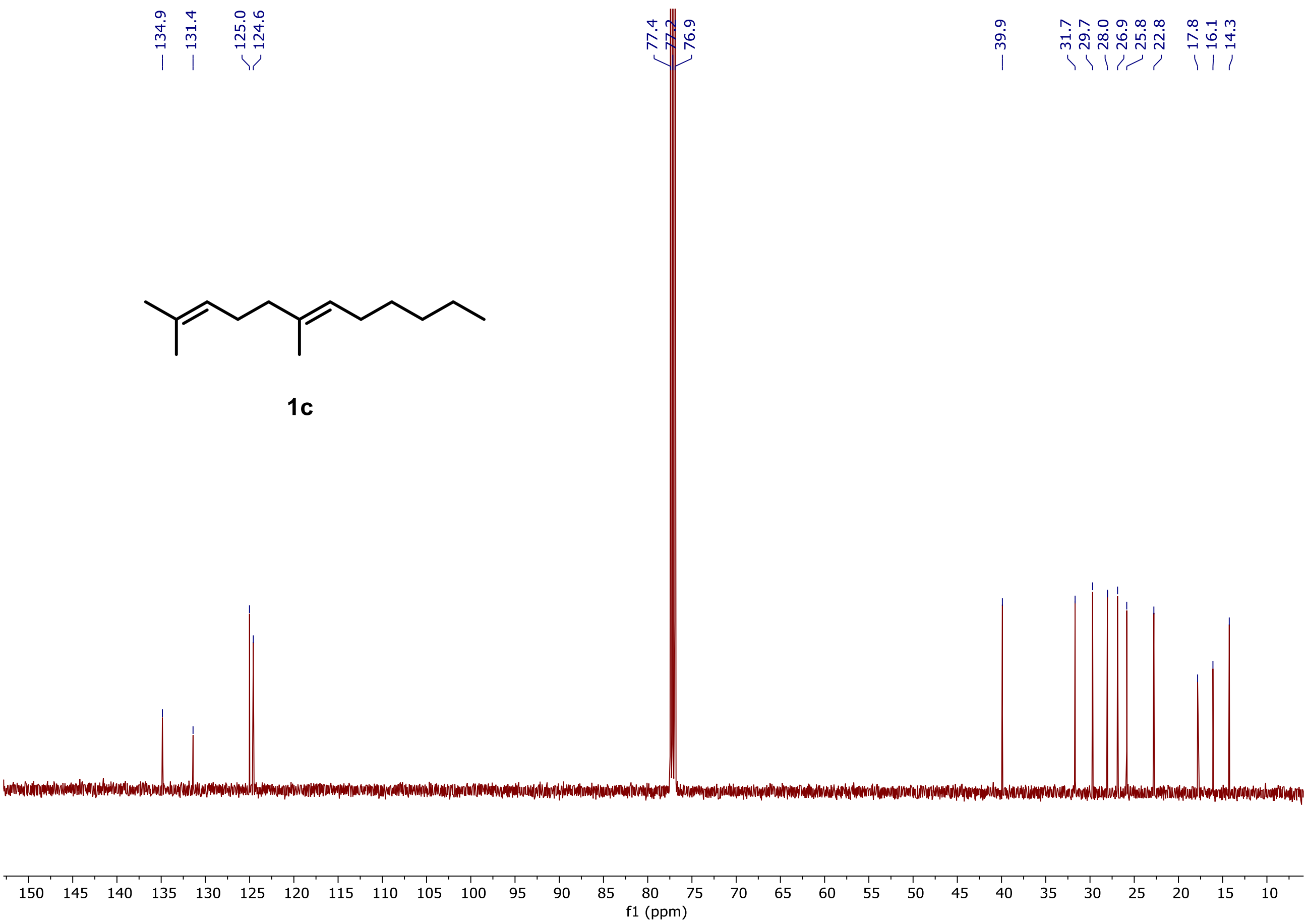

^{1}H NMR spectrum of **1d**

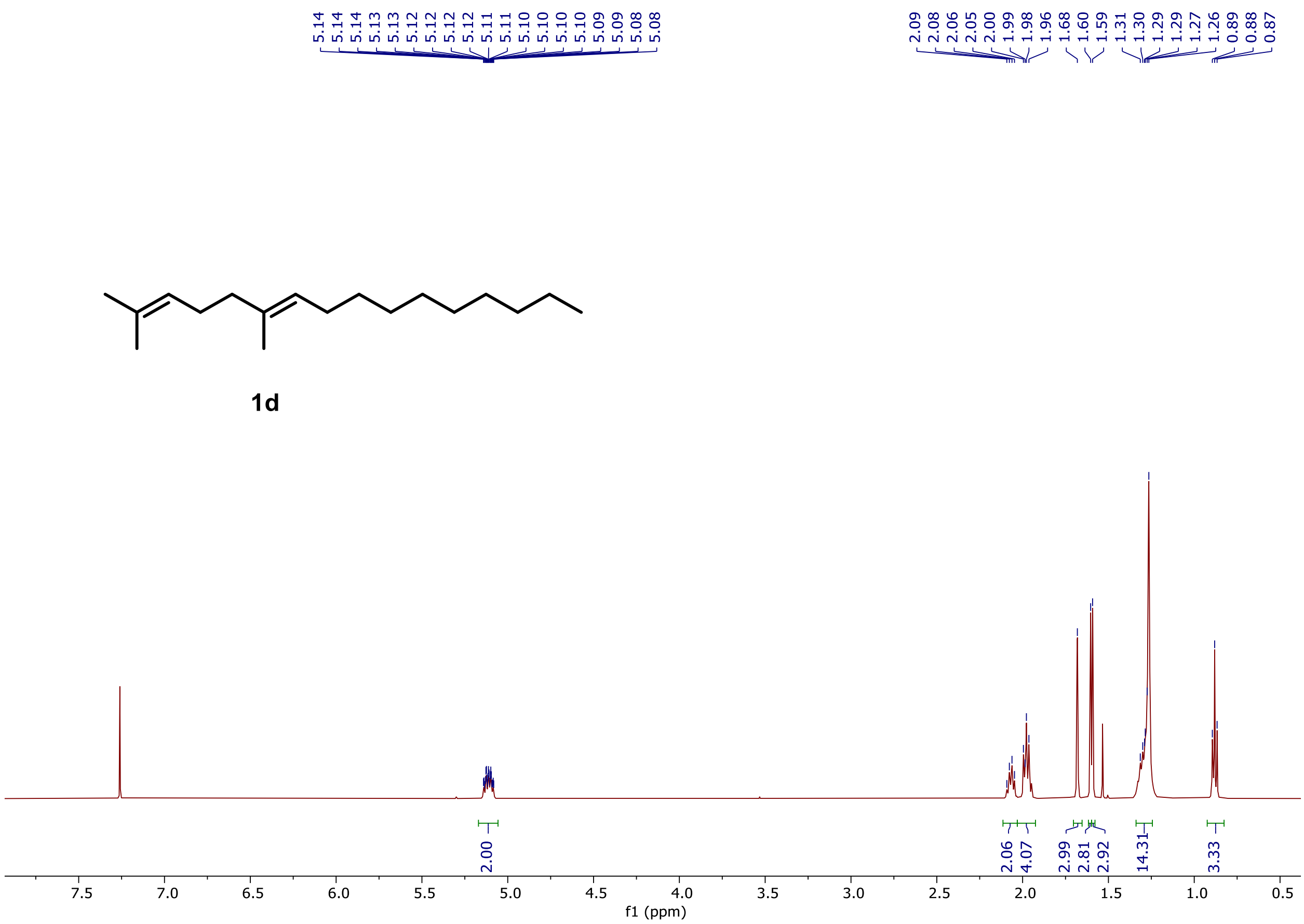

^{13}C NMR spectrum of **1d**

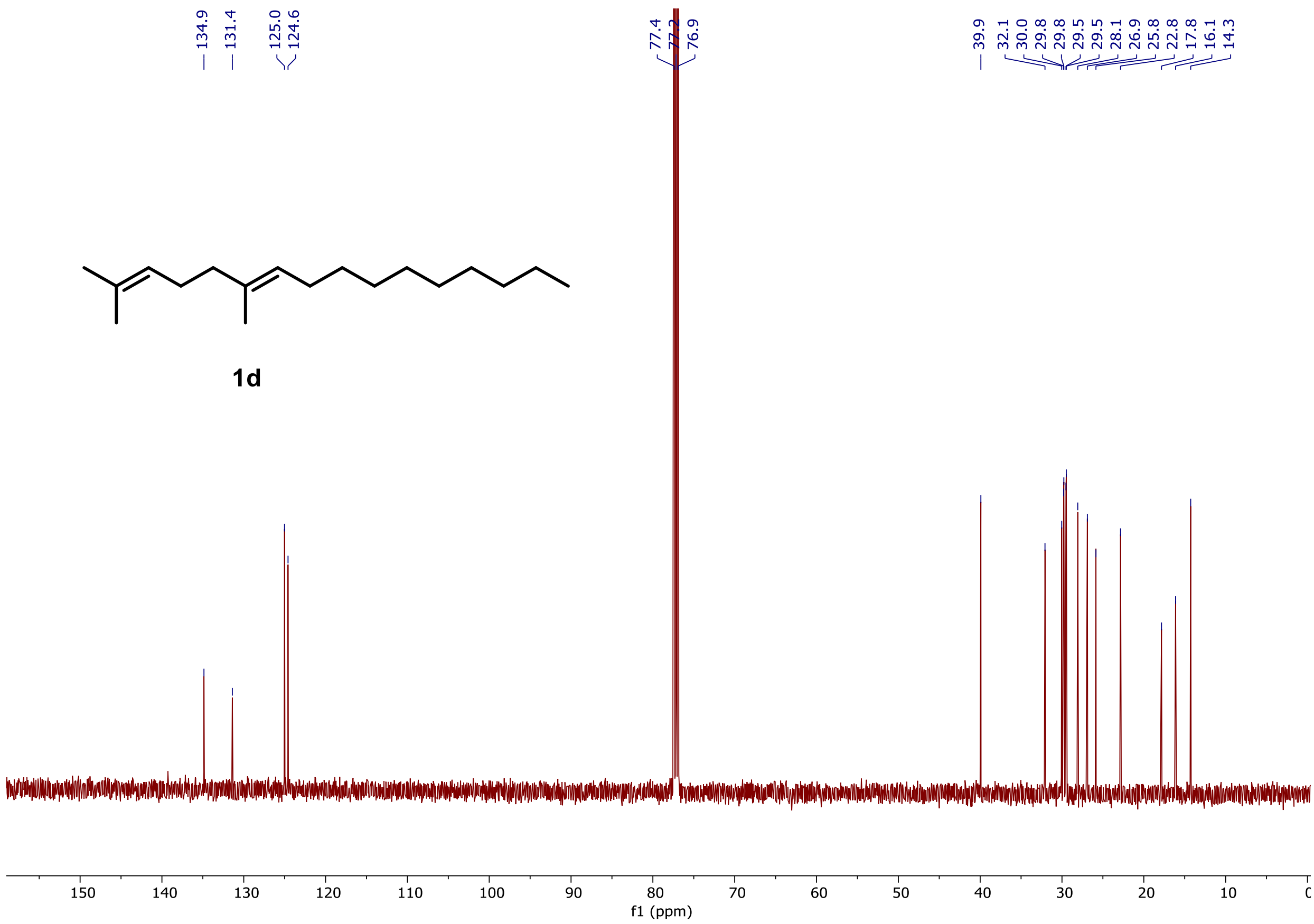

^{1}H NMR spectrum of **1e**

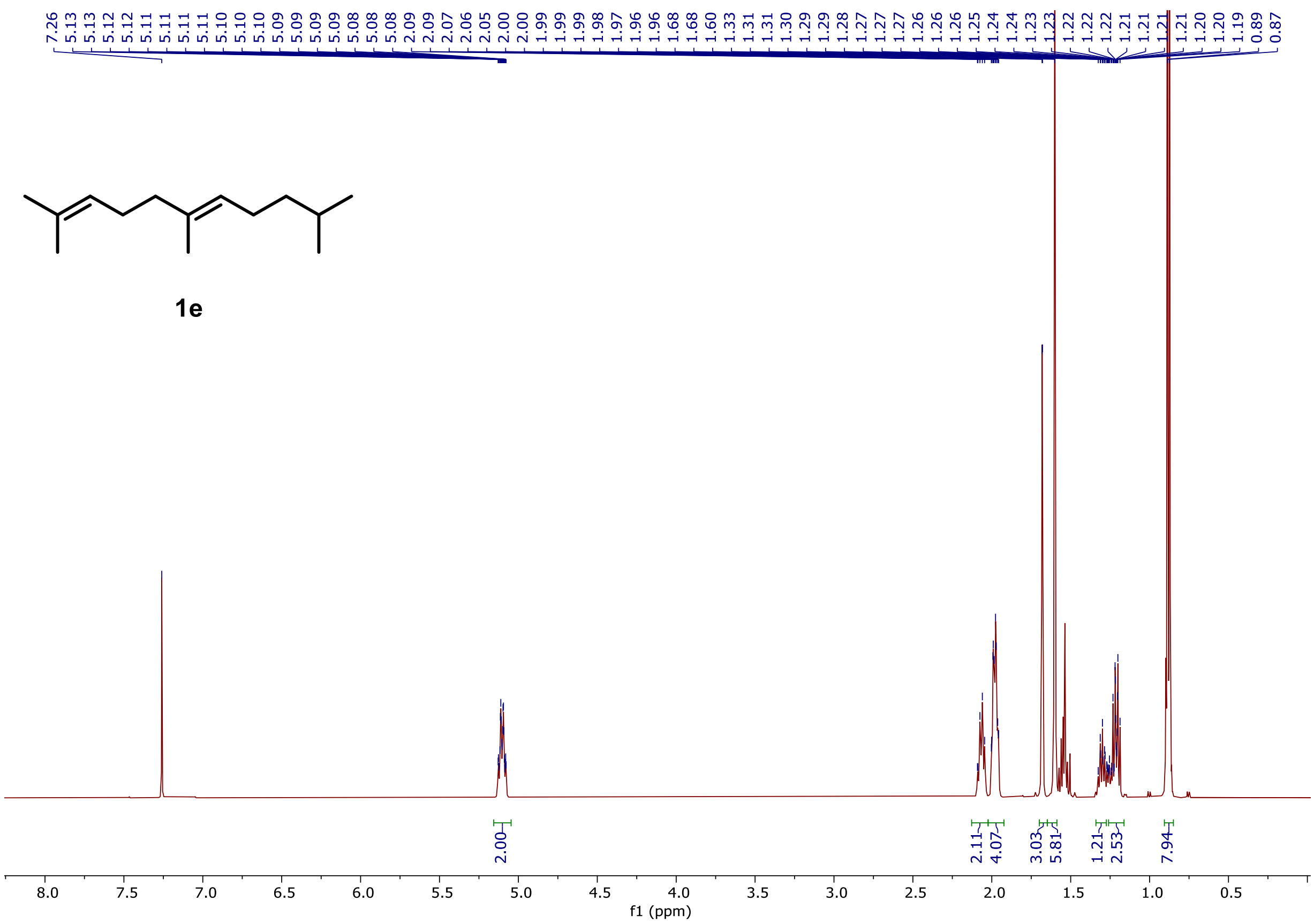

^{13}C NMR spectrum of **1e**

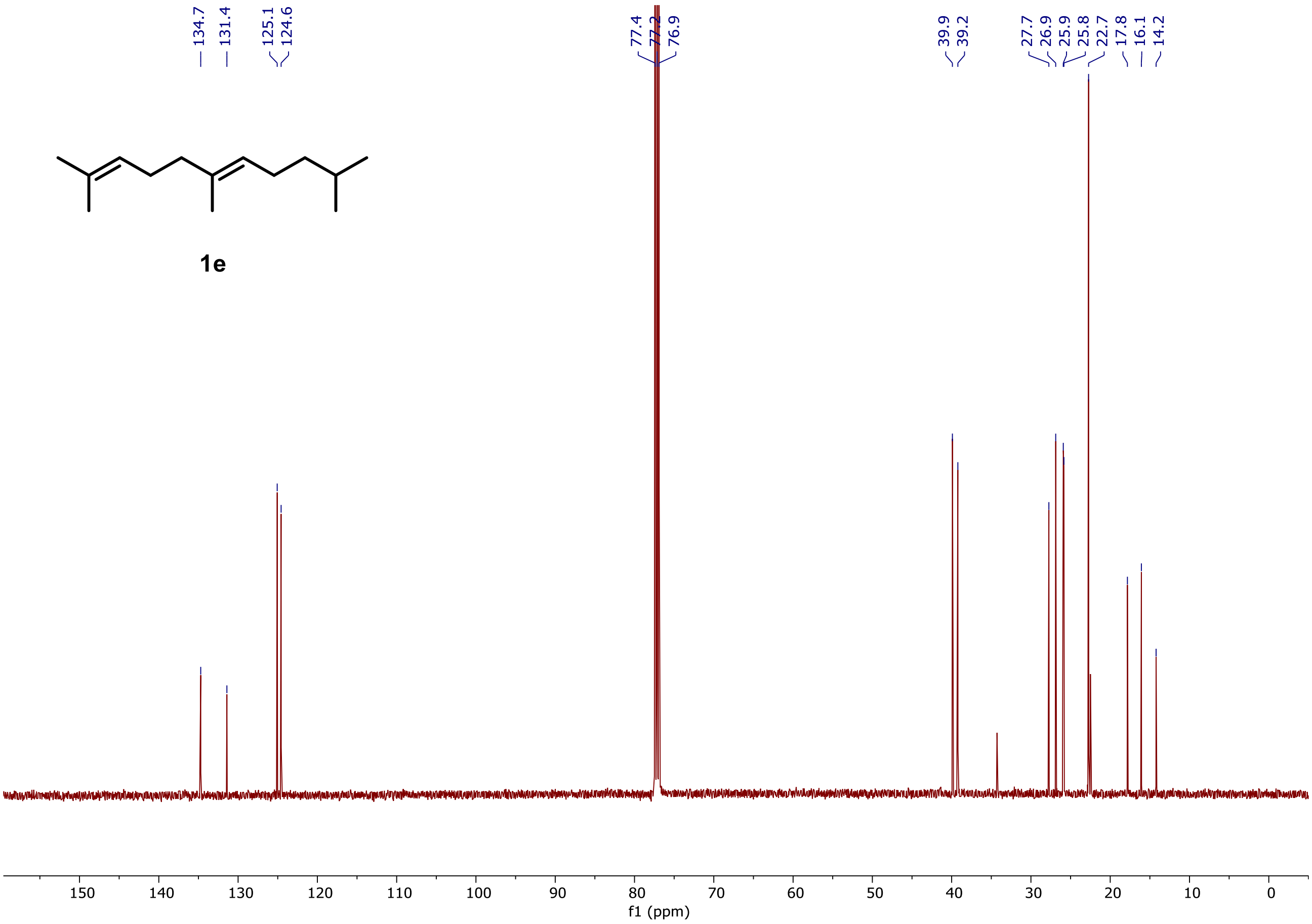

^{1}H NMR spectrum of **1f**

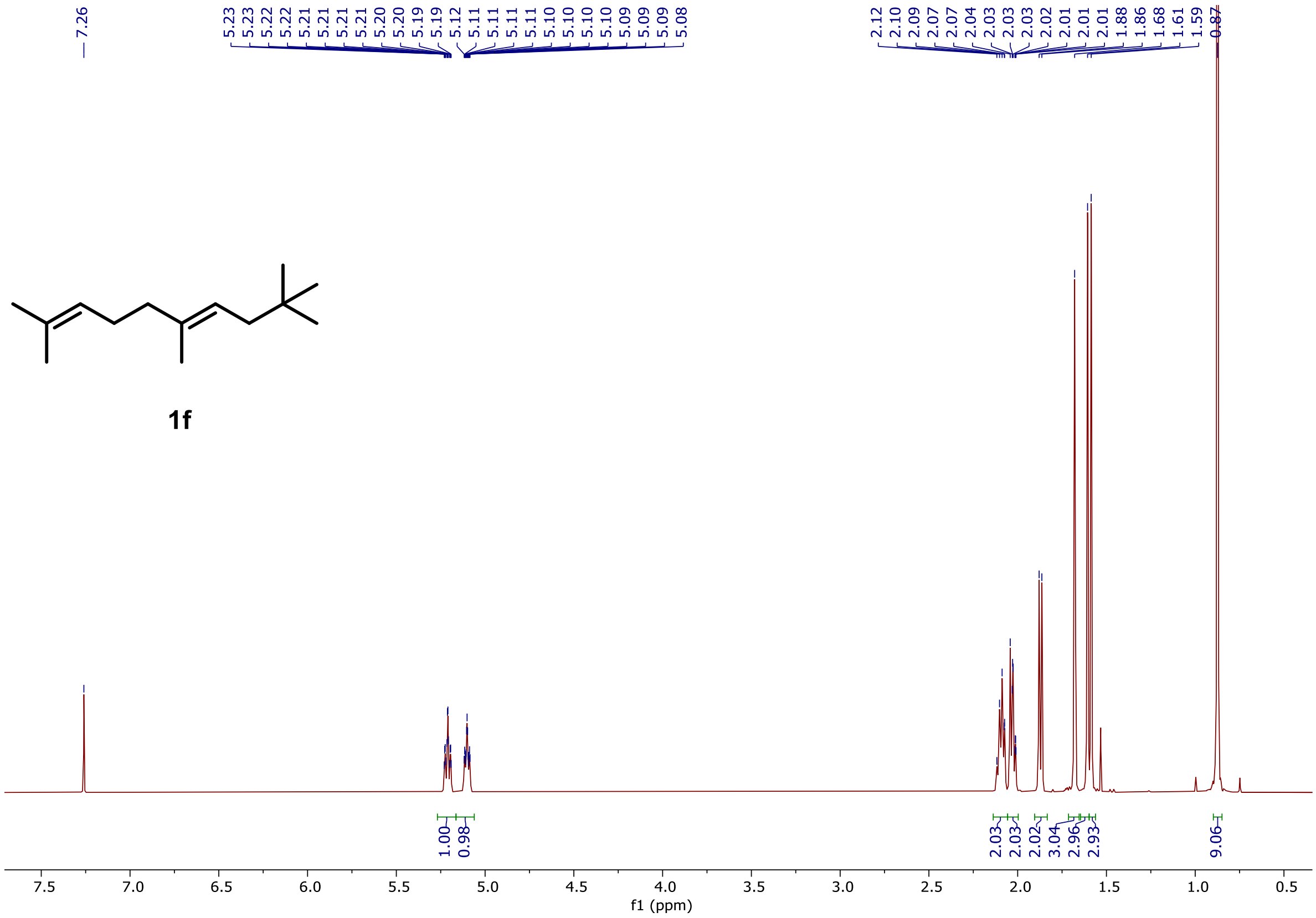

1f

^{13}C NMR spectrum of **1f**

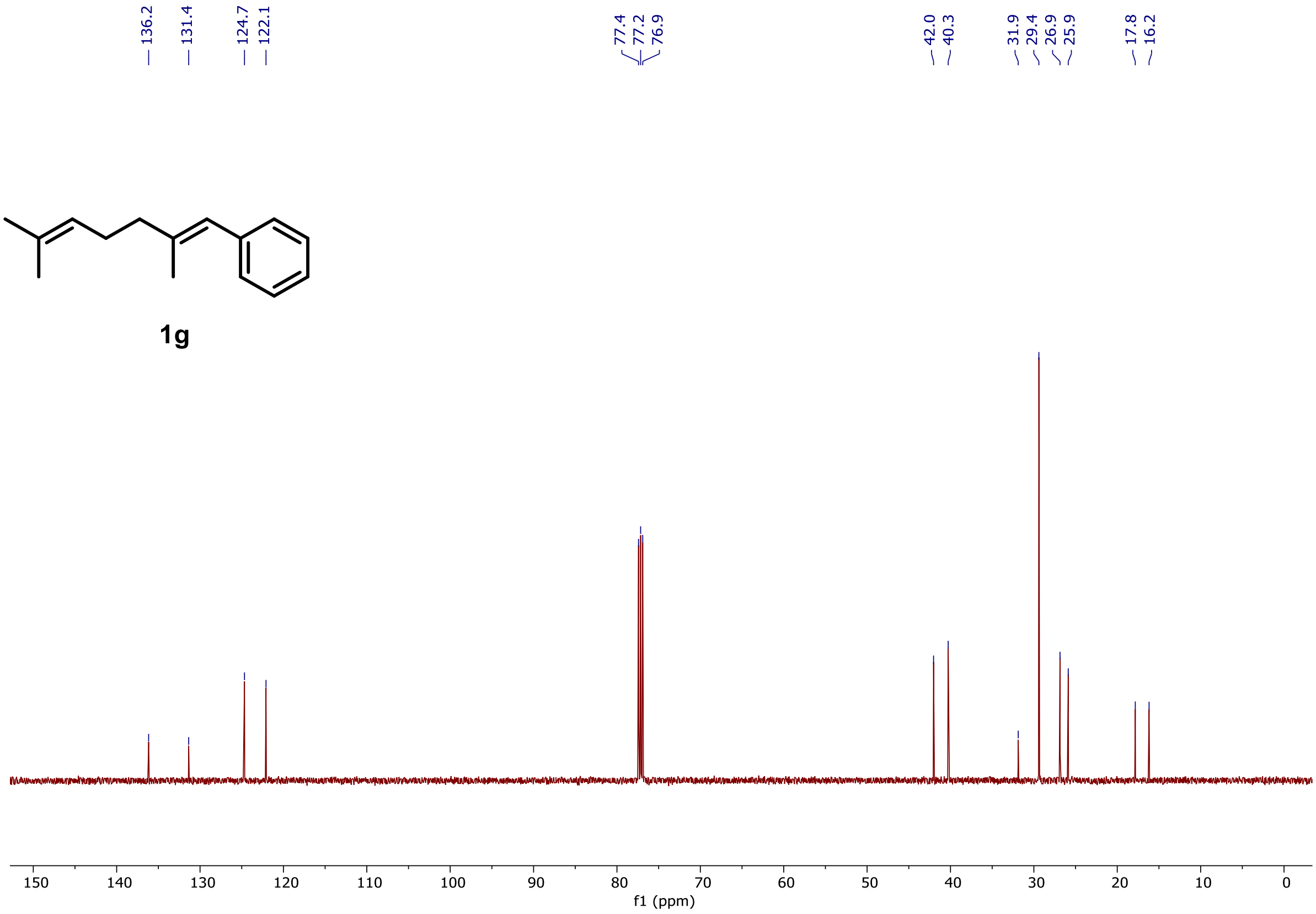

1g

^{1}H NMR spectrum of **1g**

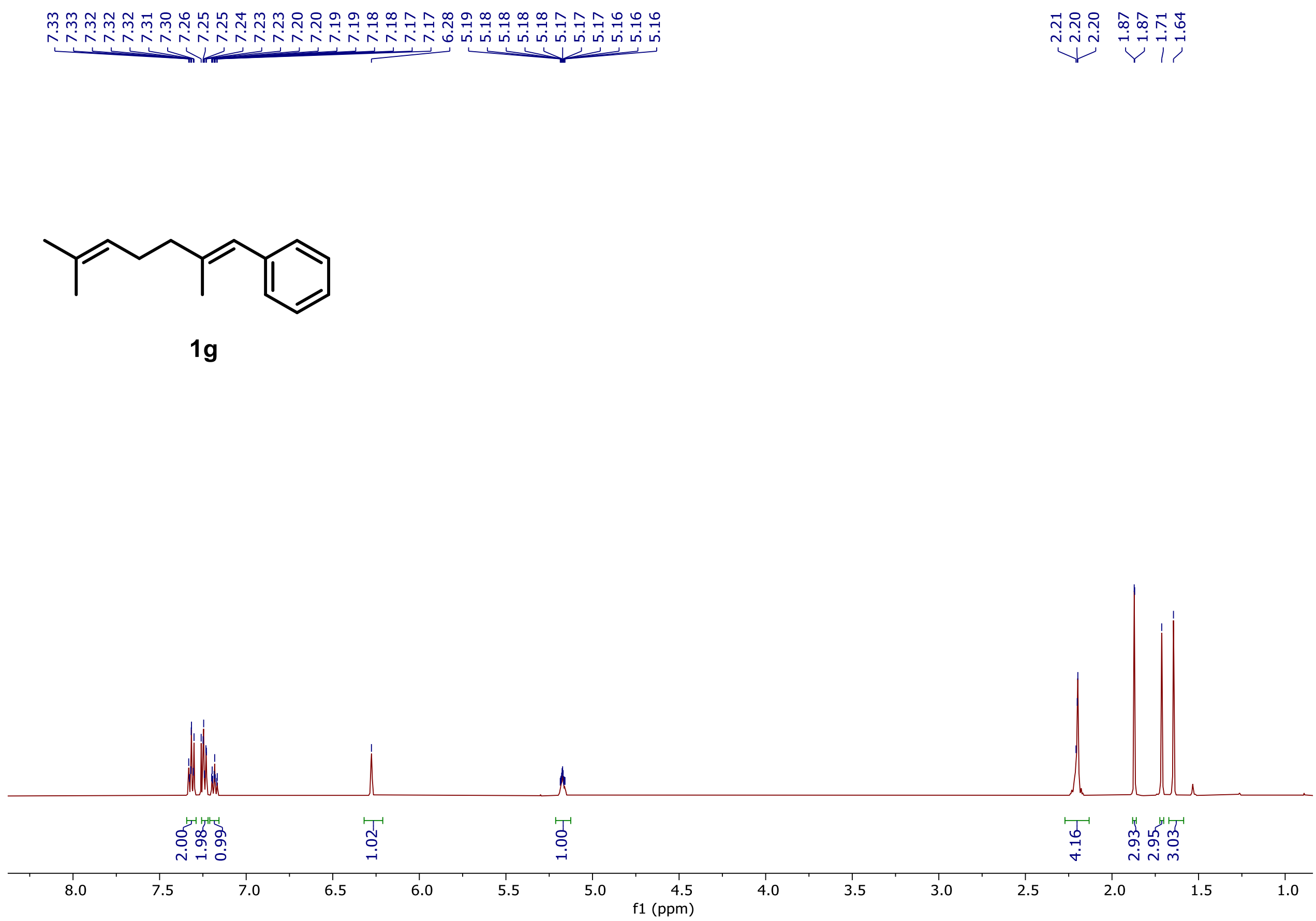

^{13}C NMR spectrum of **1g**

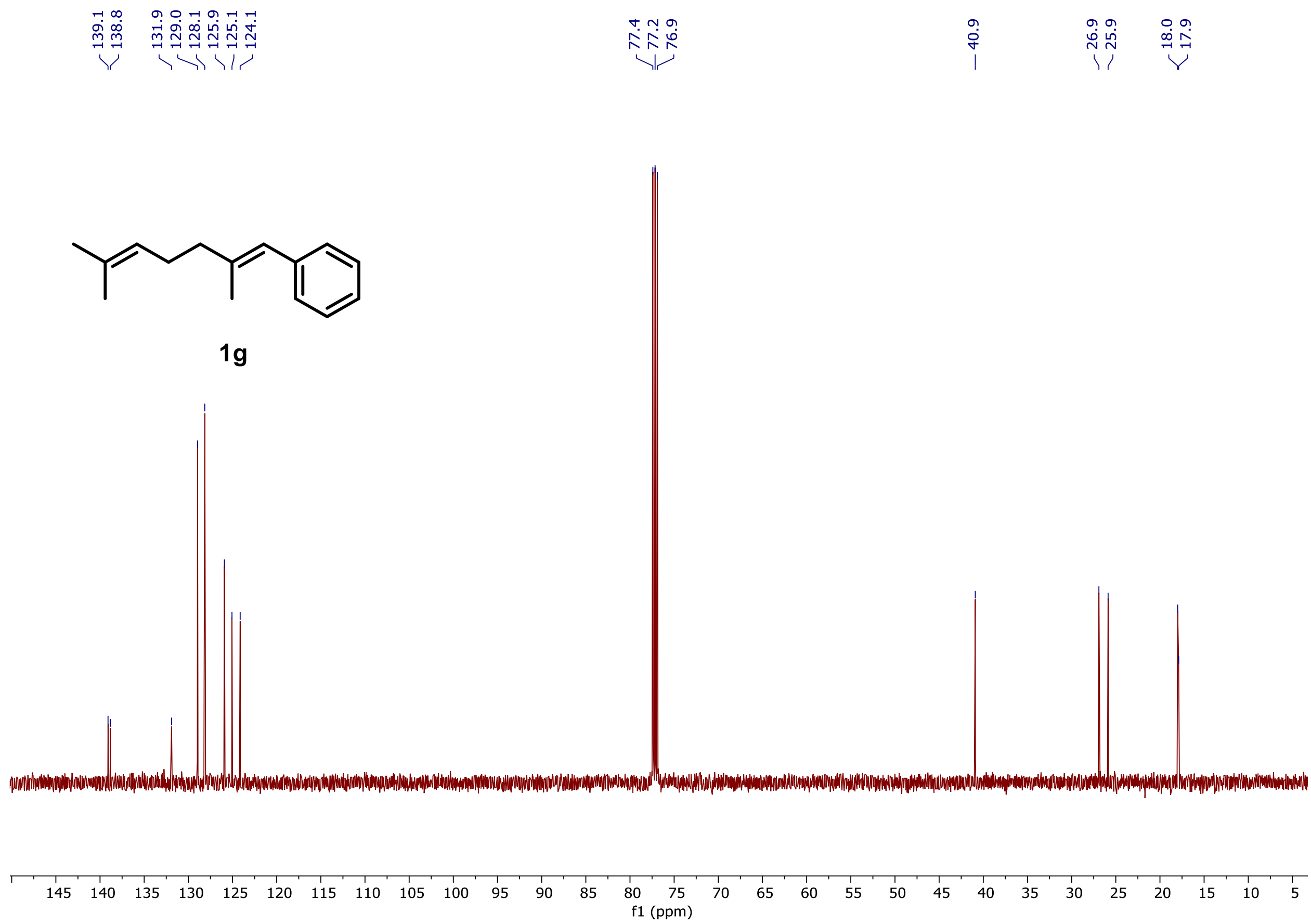

^{1}H NMR spectrum of **1h**

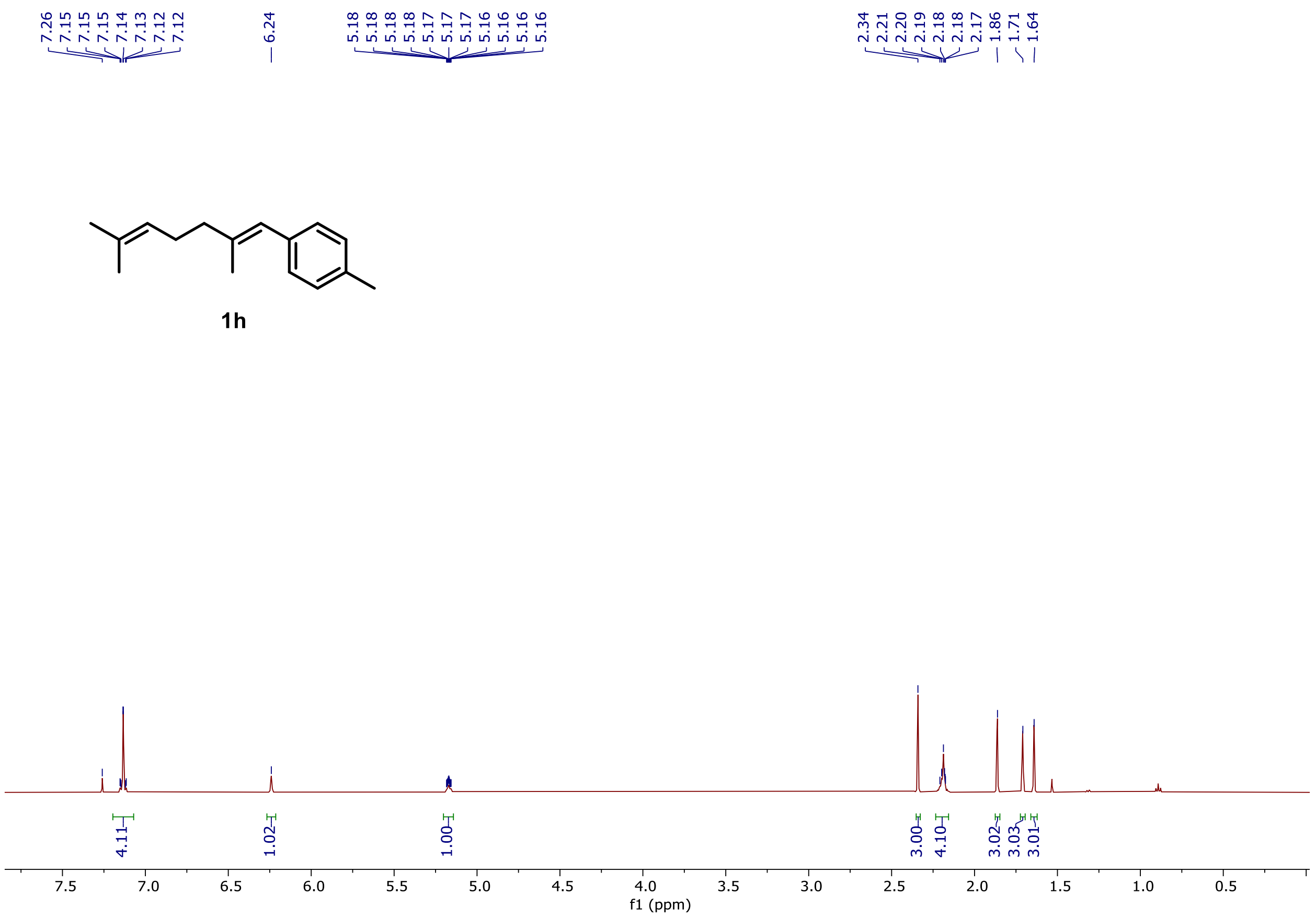

^{13}C NMR spectrum of **1h**

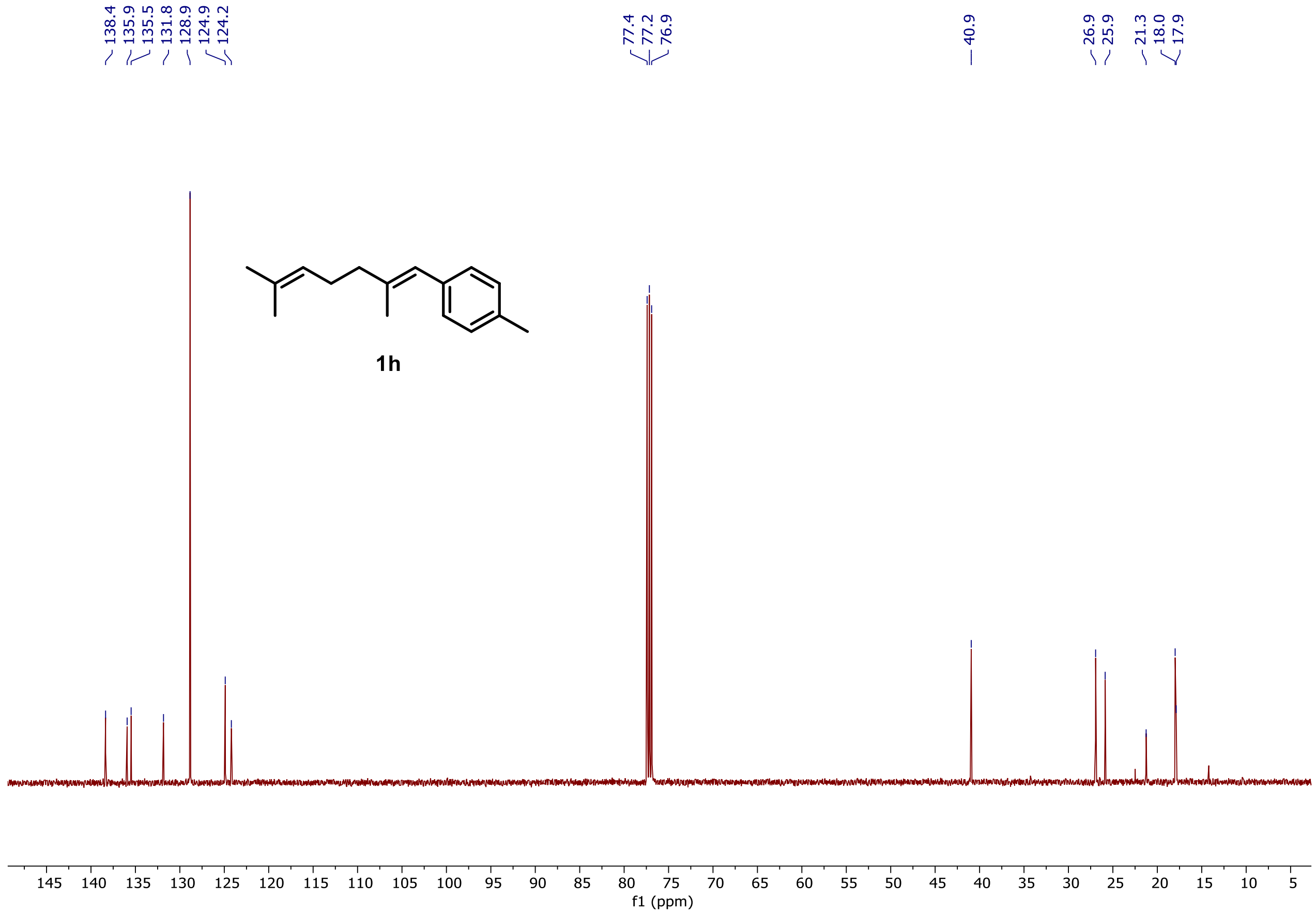

^{1}H NMR spectrum of **1i**

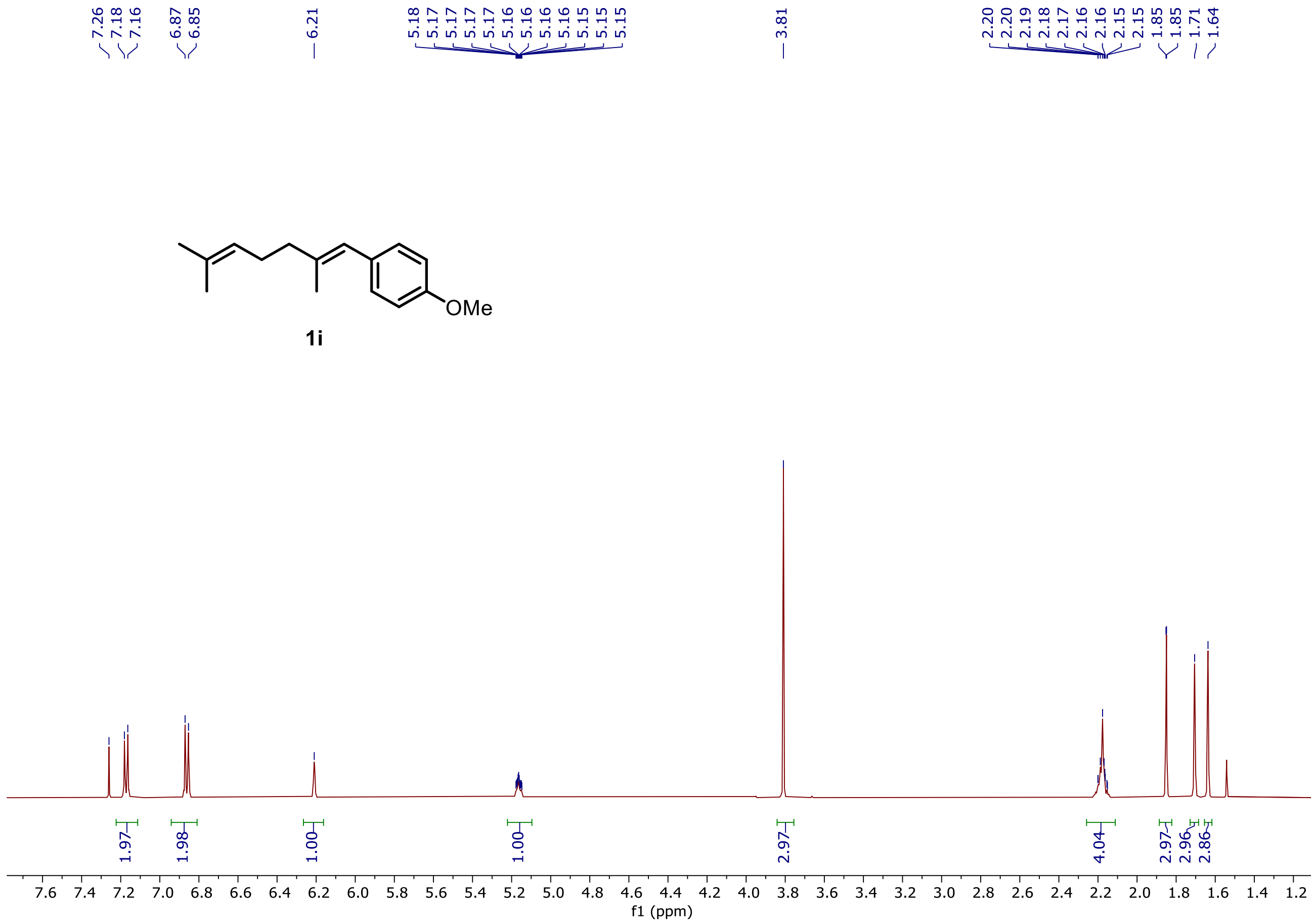

^{13}C NMR spectrum of **1i**

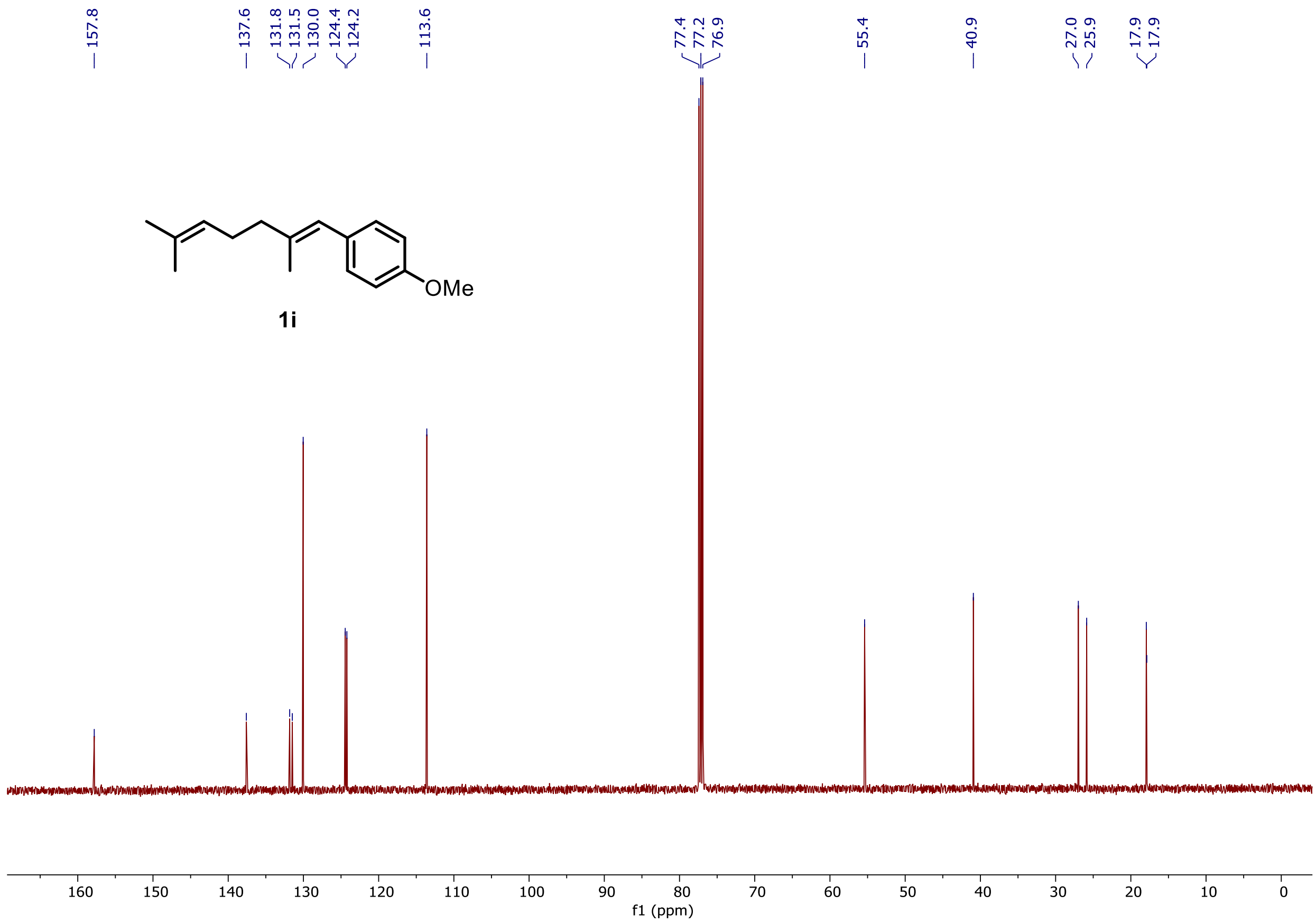

^{1}H NMR spectrum of **1j**

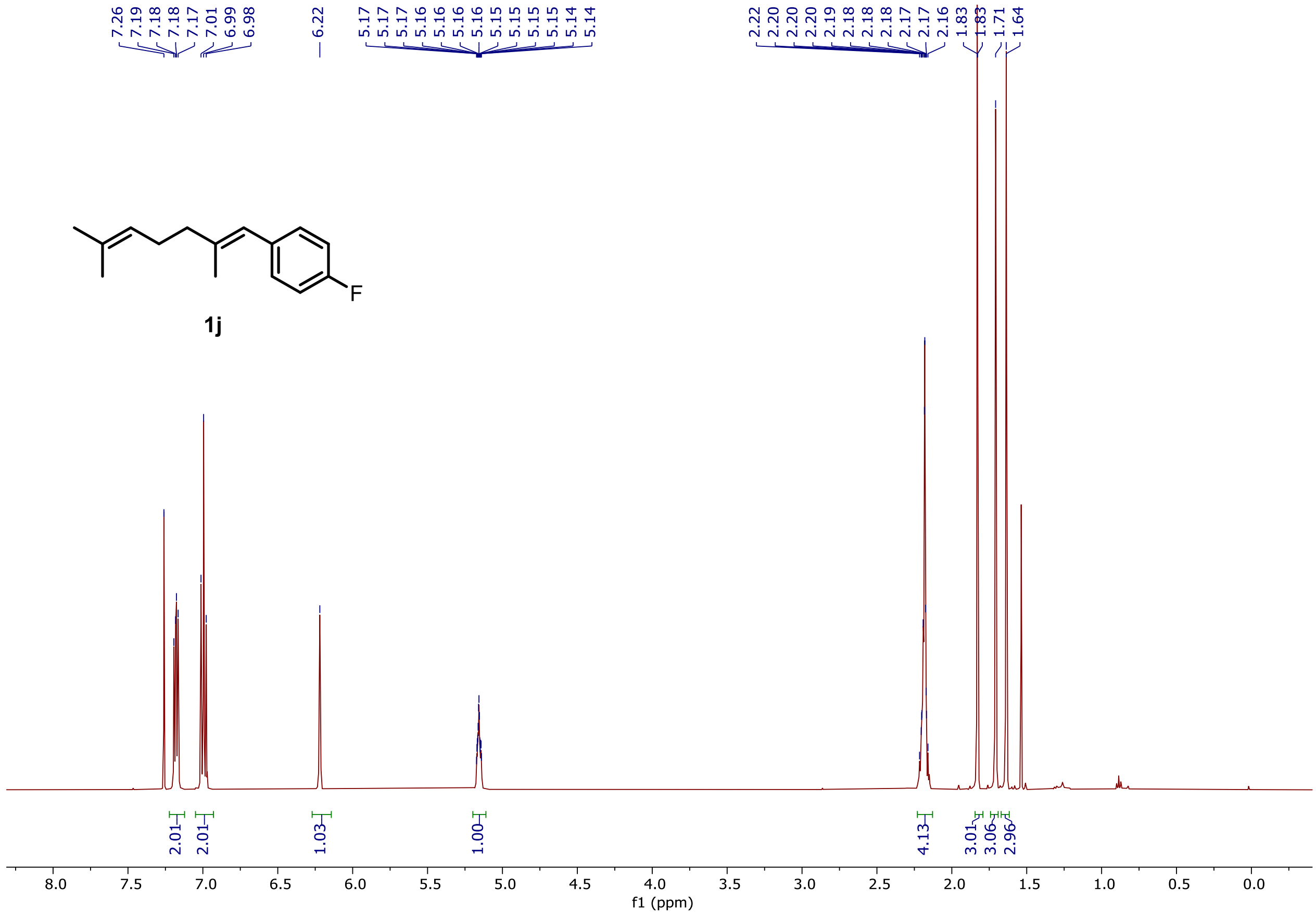

^{13}C NMR spectrum of **1j**

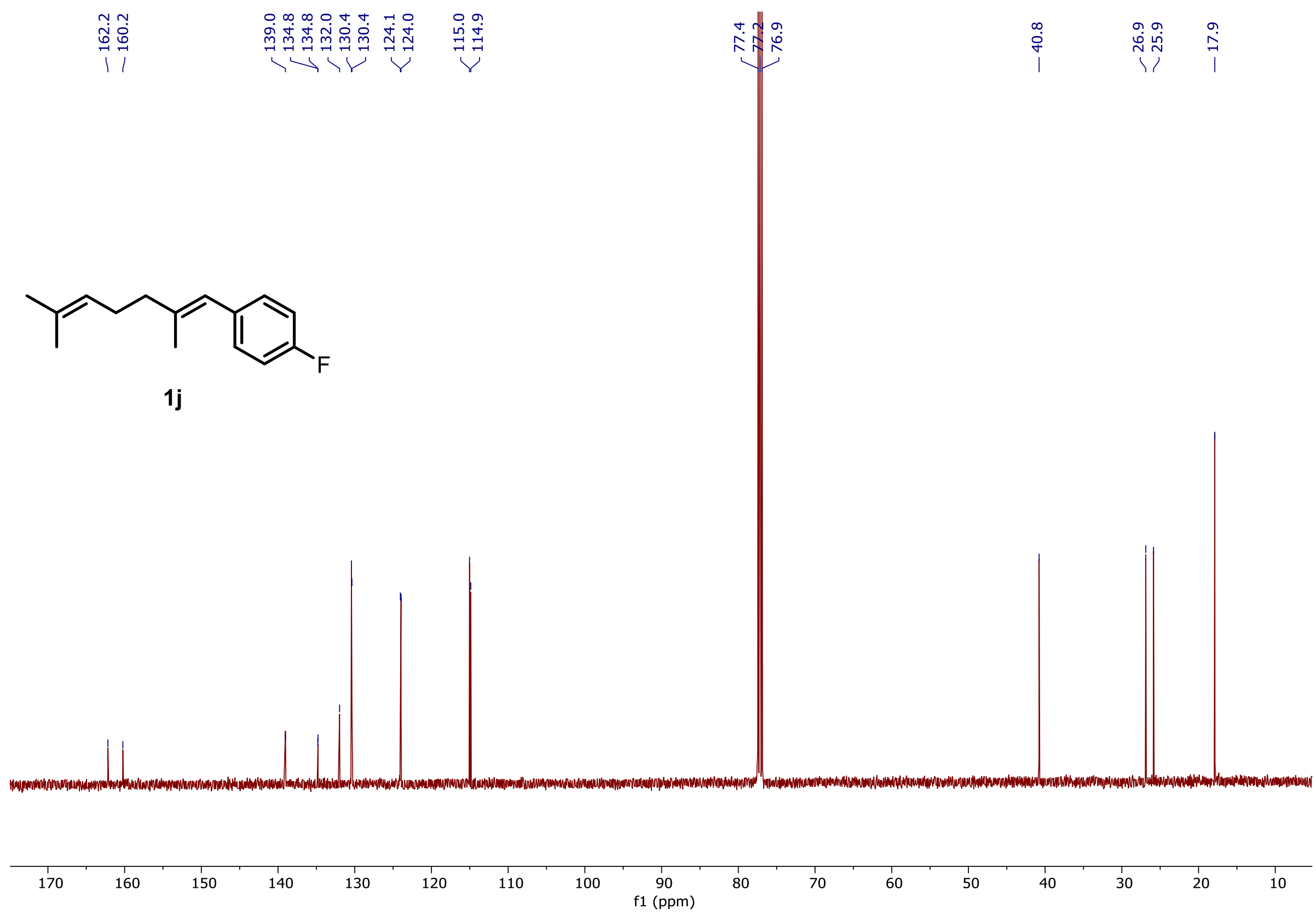

^{19}F NMR spectrum of **1j**

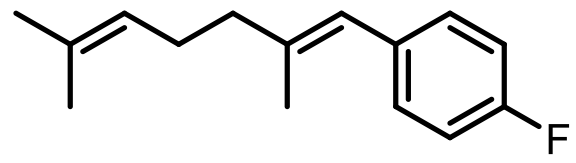

1j

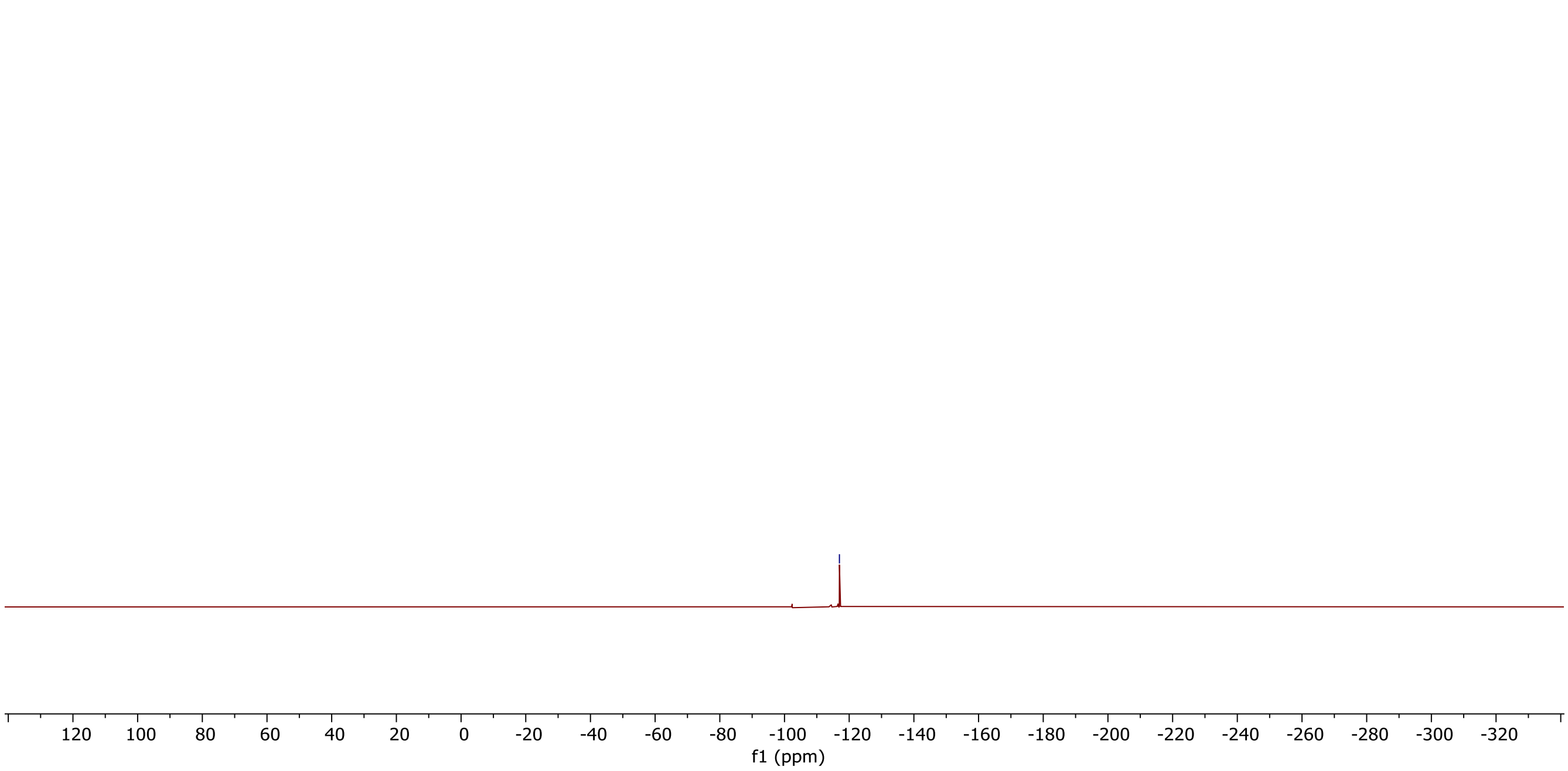

^{1}H NMR spectrum of **1k**

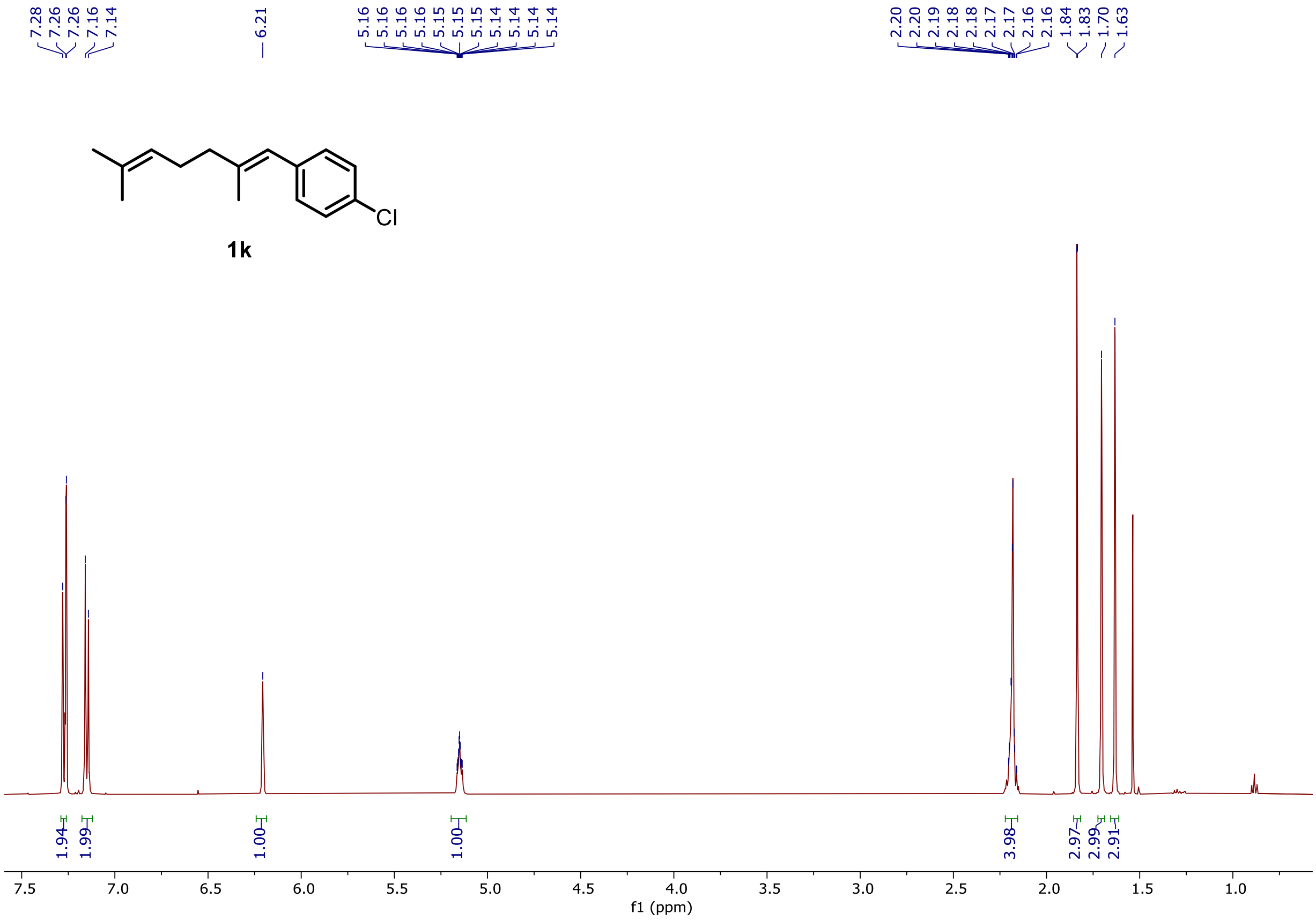

^{13}C NMR spectrum of **1k**

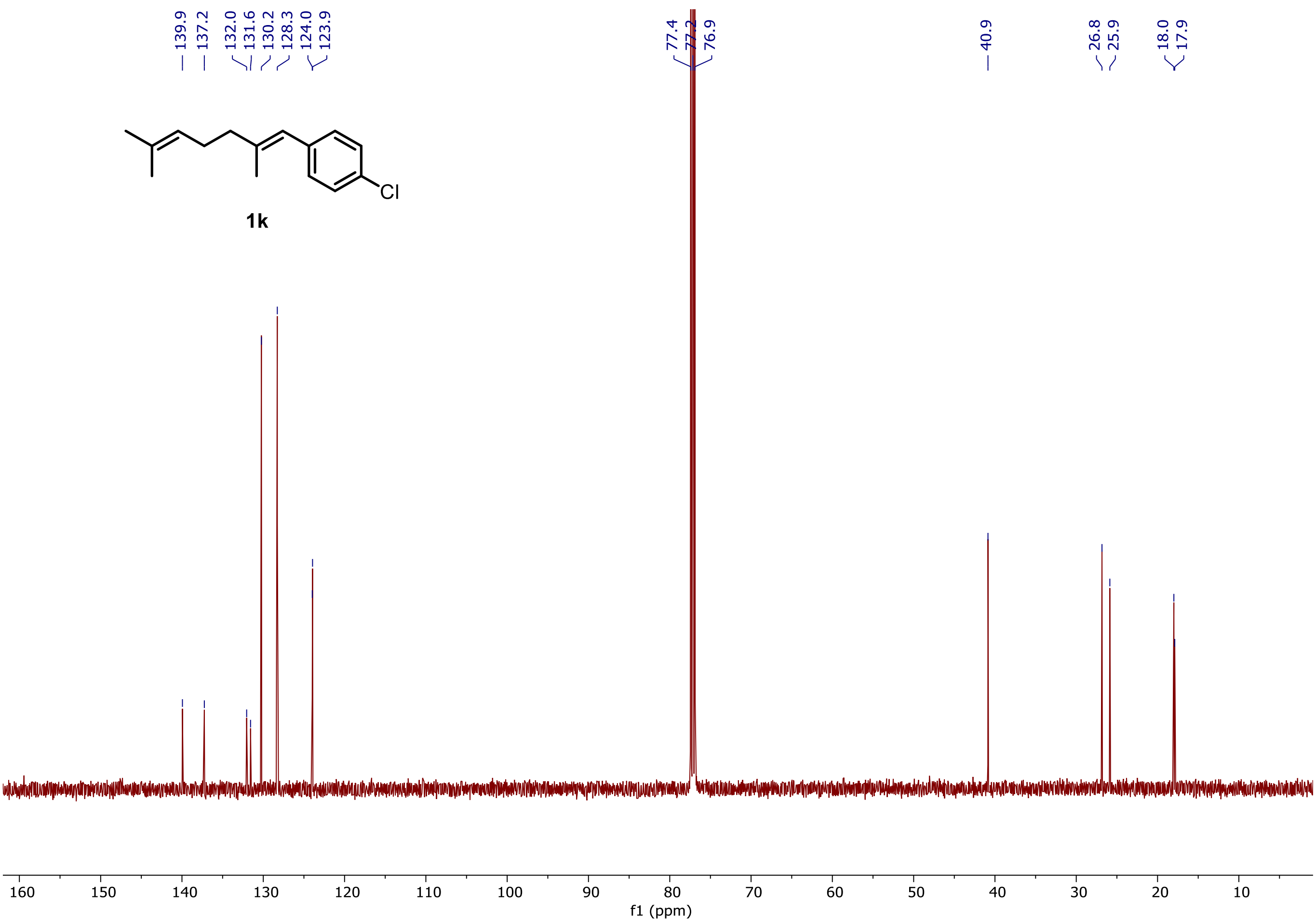

^{1}H NMR spectrum of **1l**

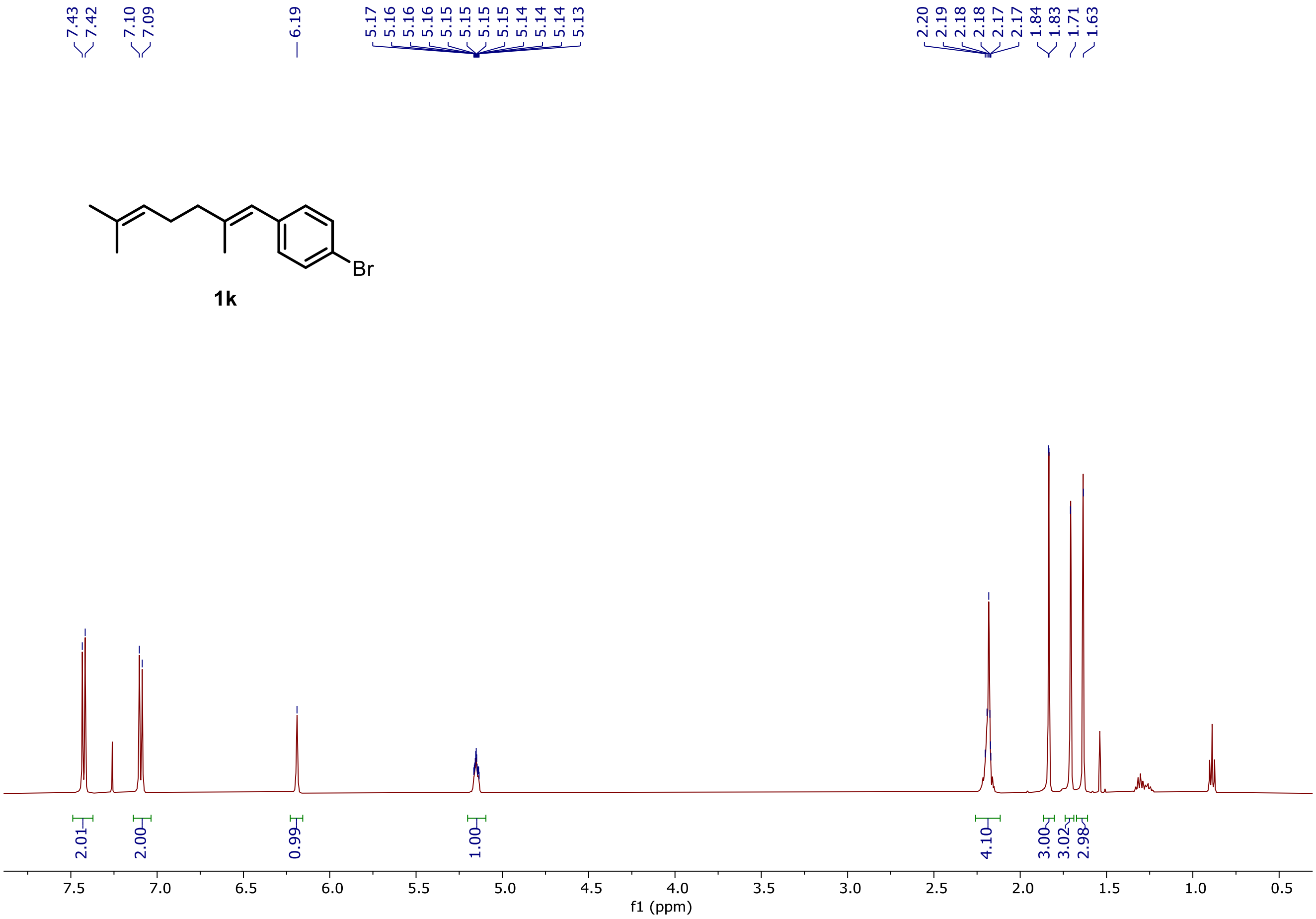

^{13}C NMR spectrum of **1l**

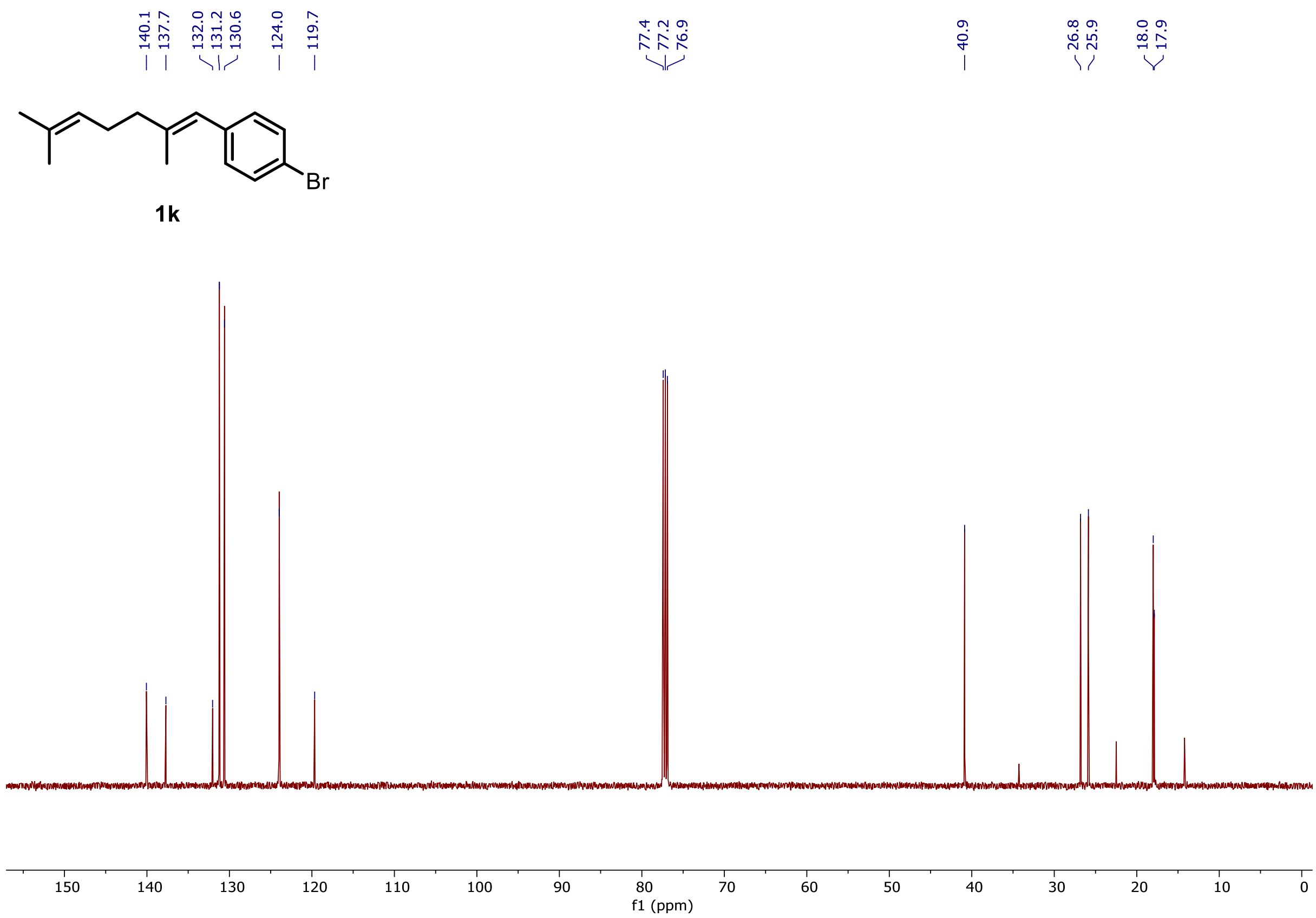

^{1}H NMR spectrum of **1m**

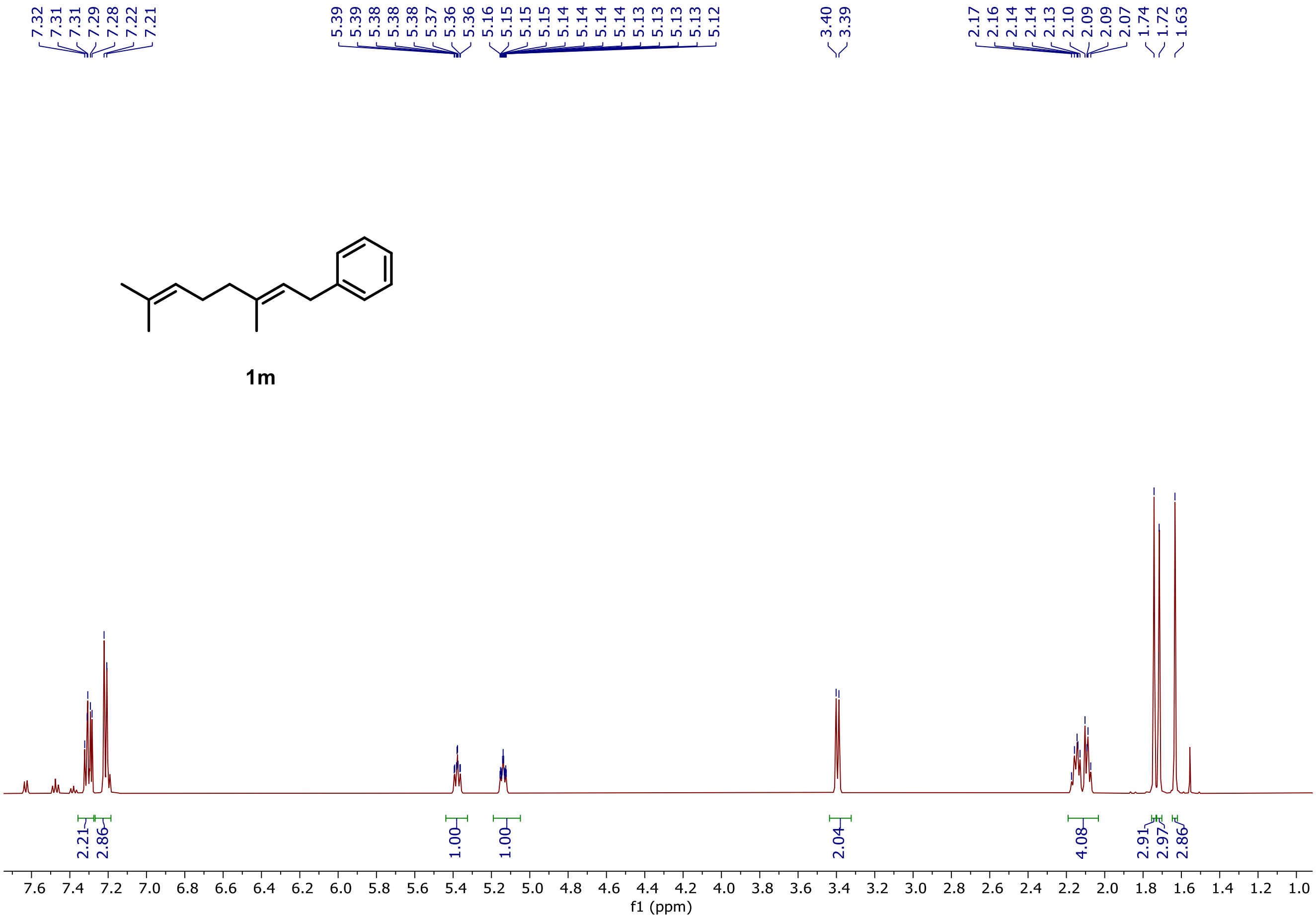

^{13}C NMR spectrum of **1m**

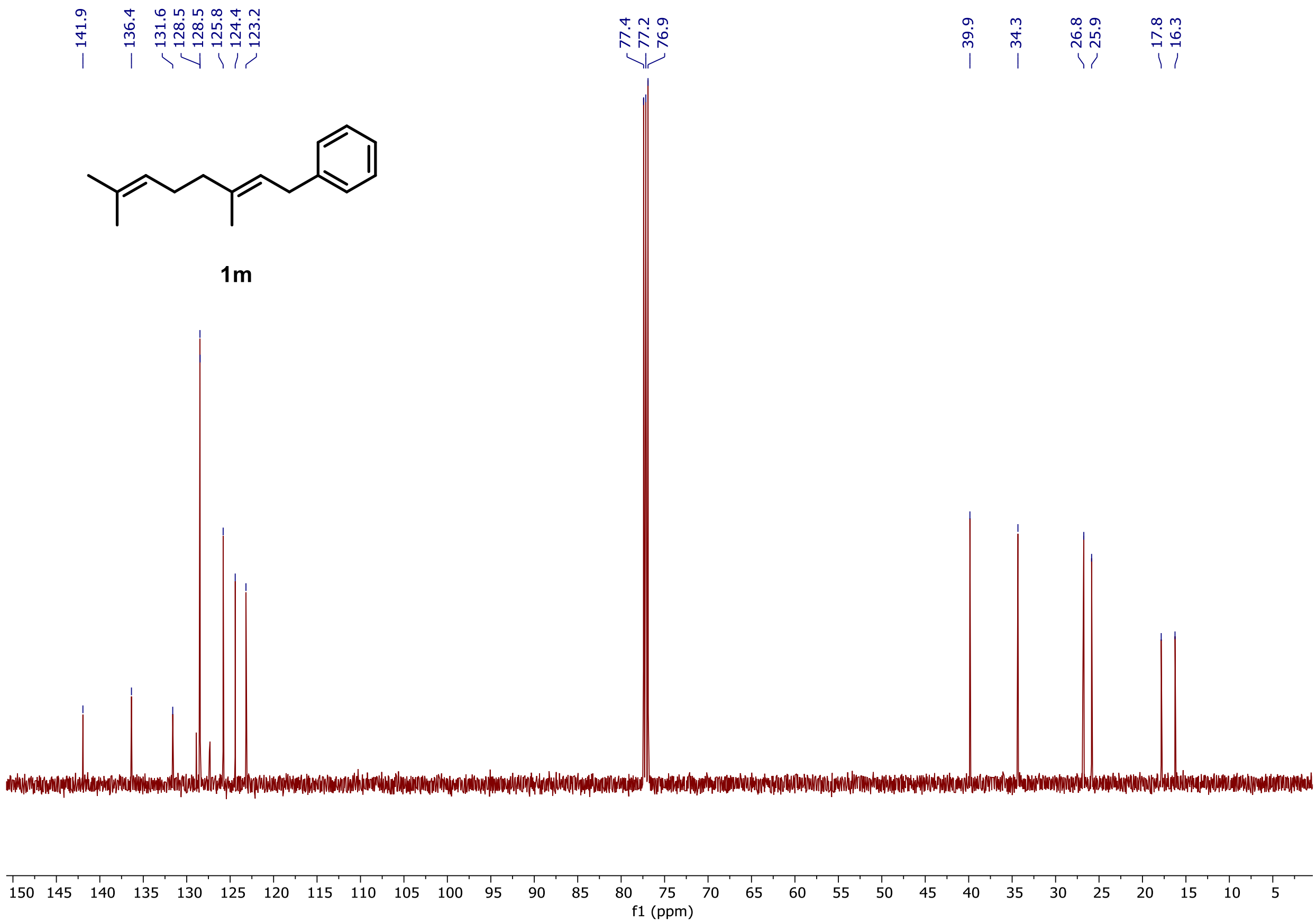

^{1}H NMR spectrum of **1n**

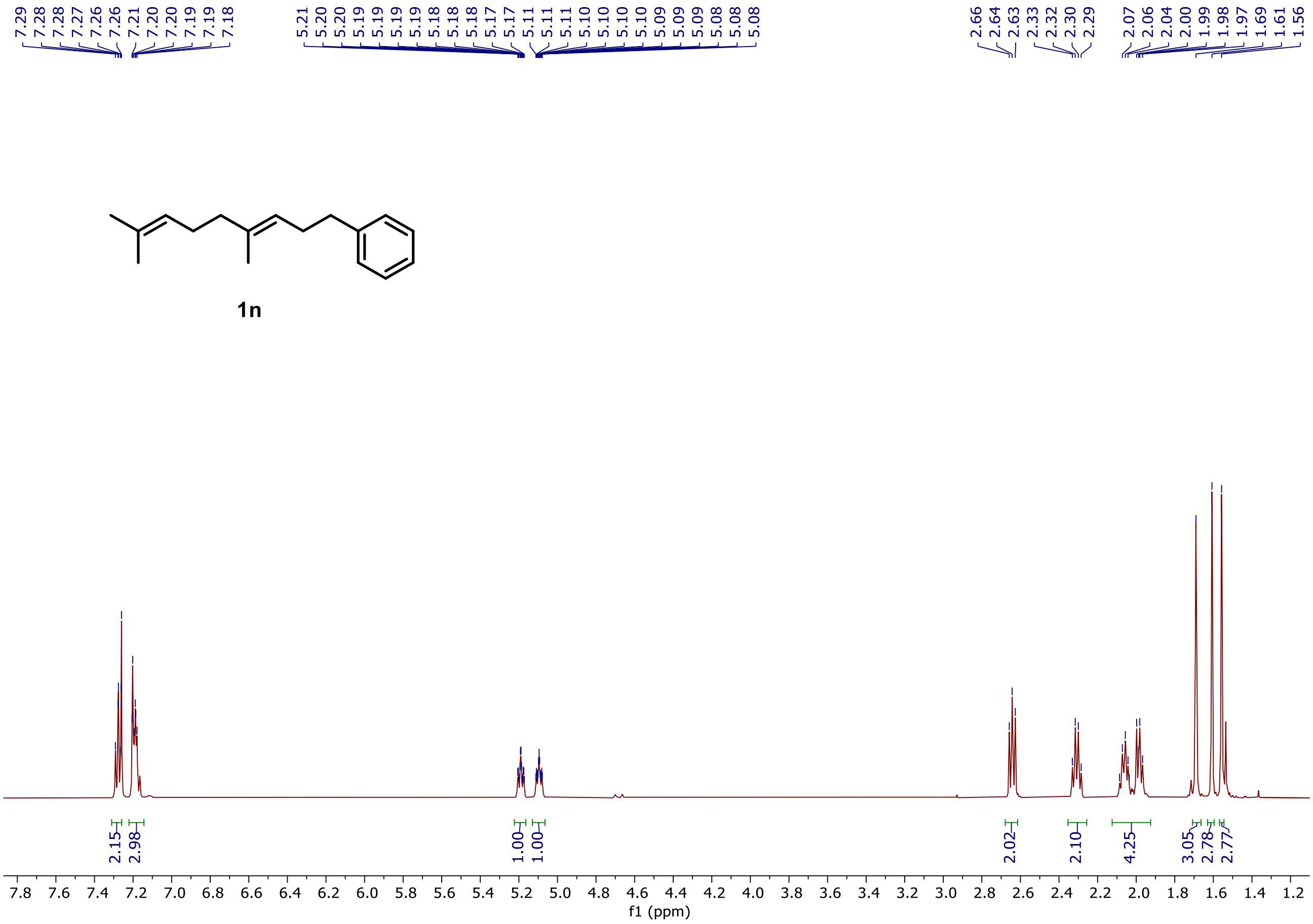

^{13}C NMR spectrum of **1n**

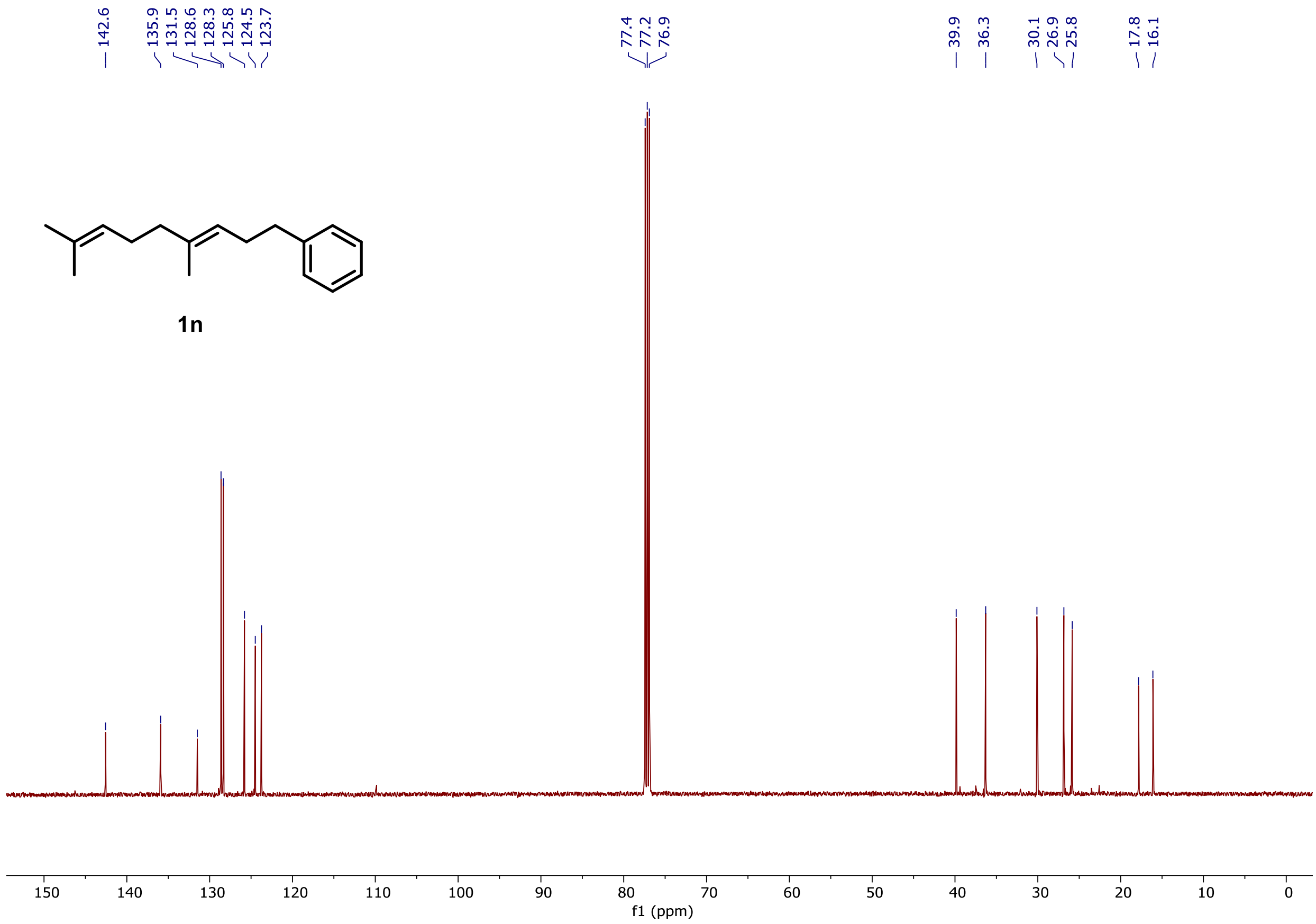

^{1}H NMR spectrum of **1o**

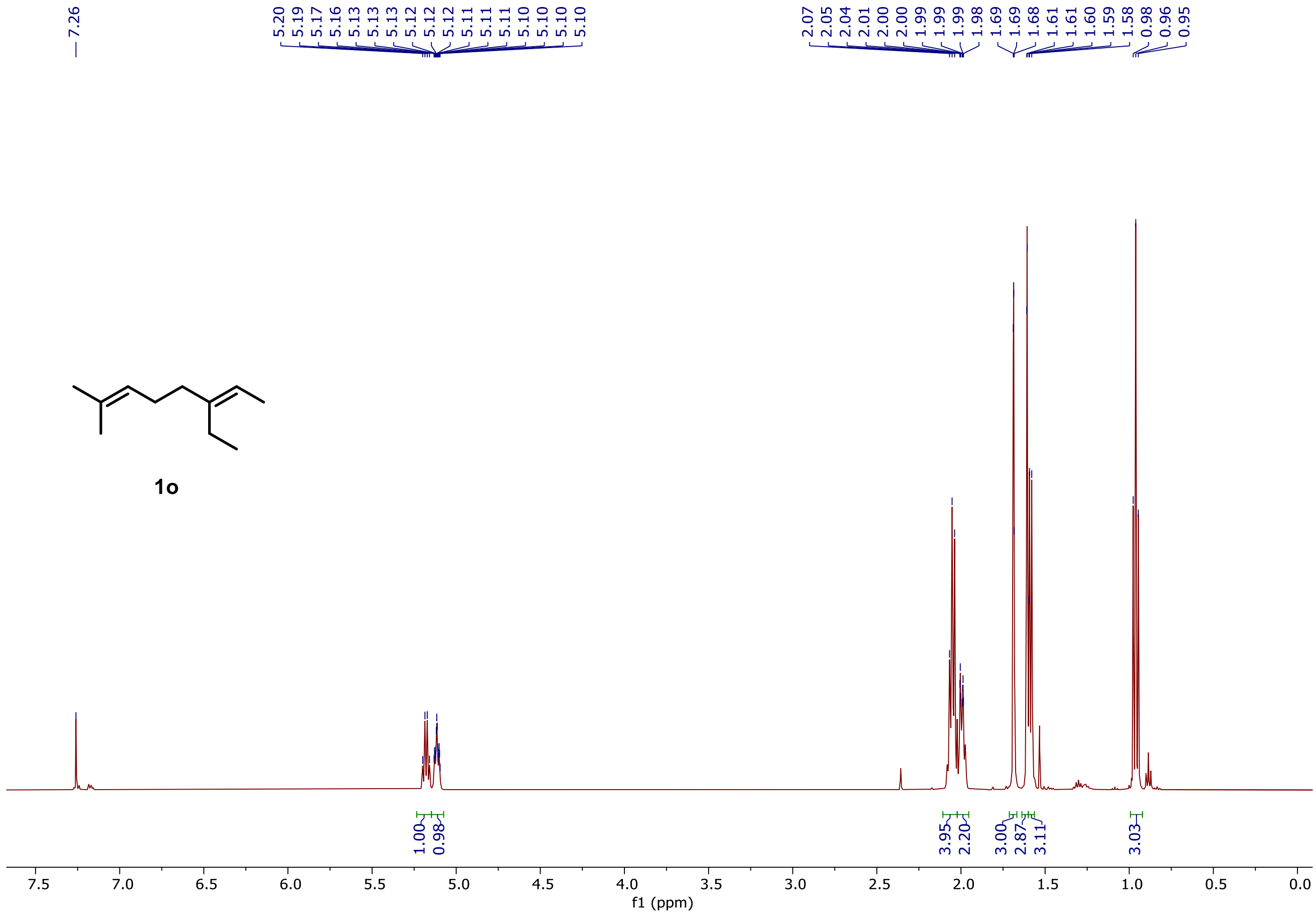

^{13}C NMR spectrum of **1o**

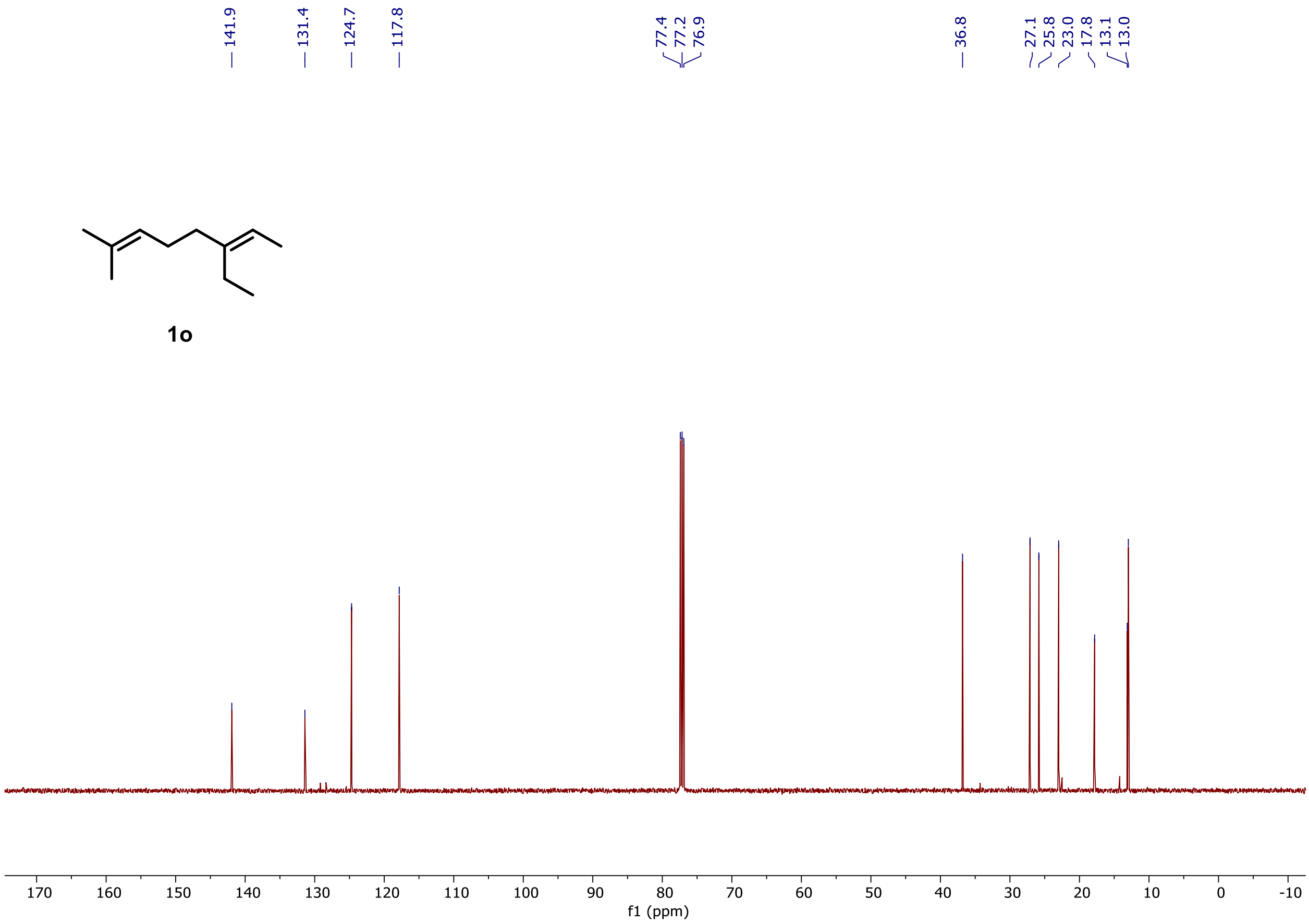

^{1}H NMR spectrum of **1p**

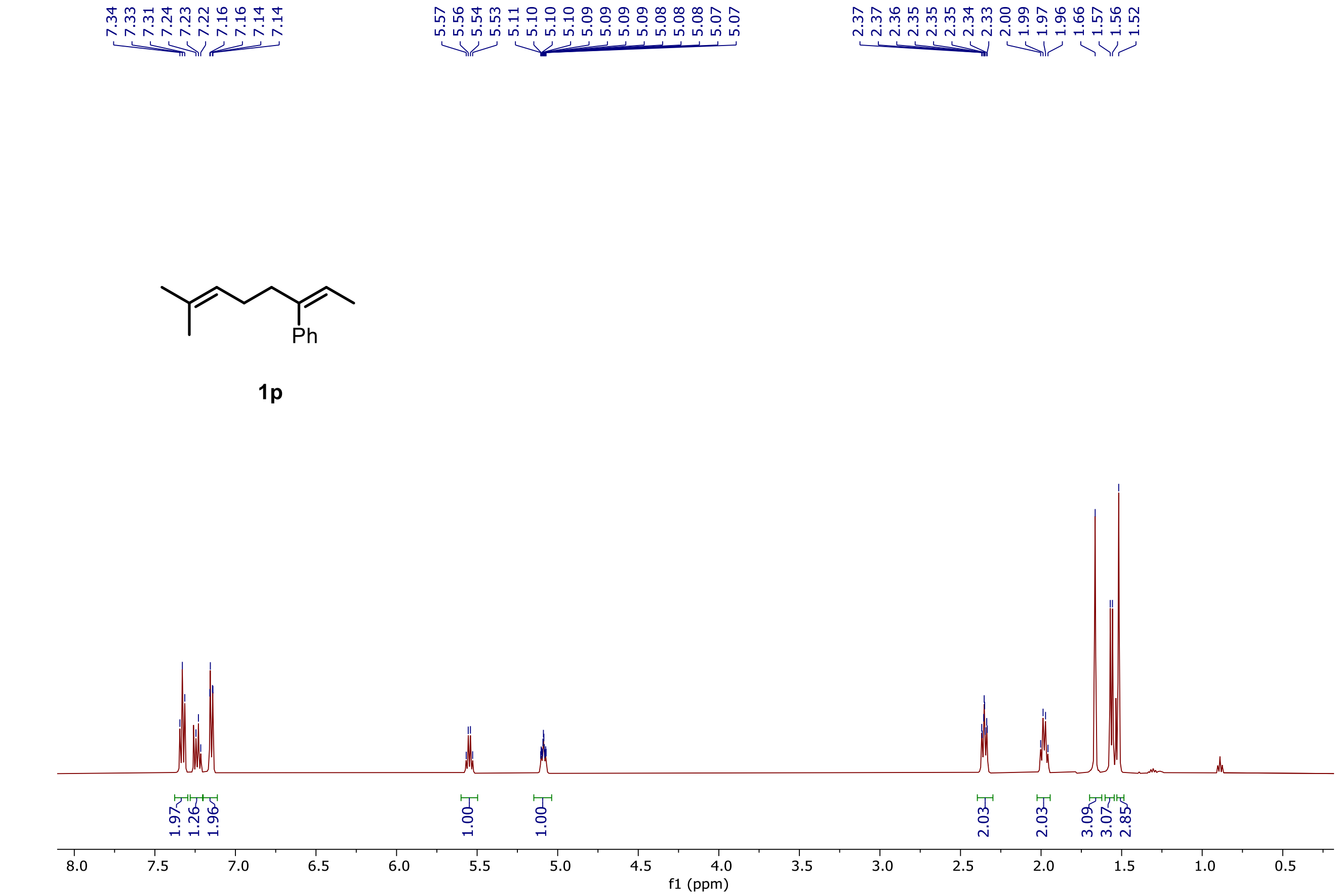

^{13}C NMR spectrum of **1p**

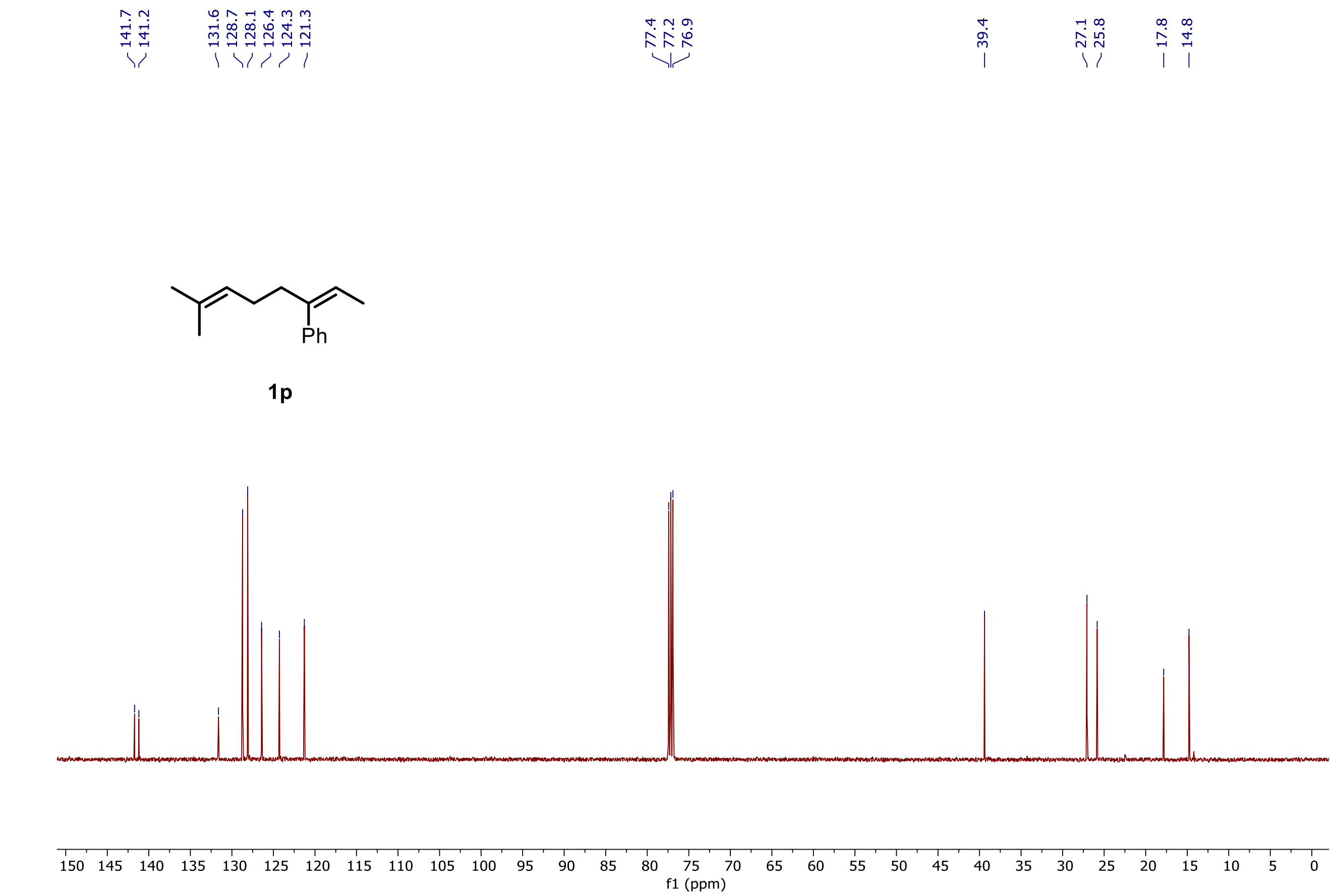

^{1}H NMR spectrum of **1q**

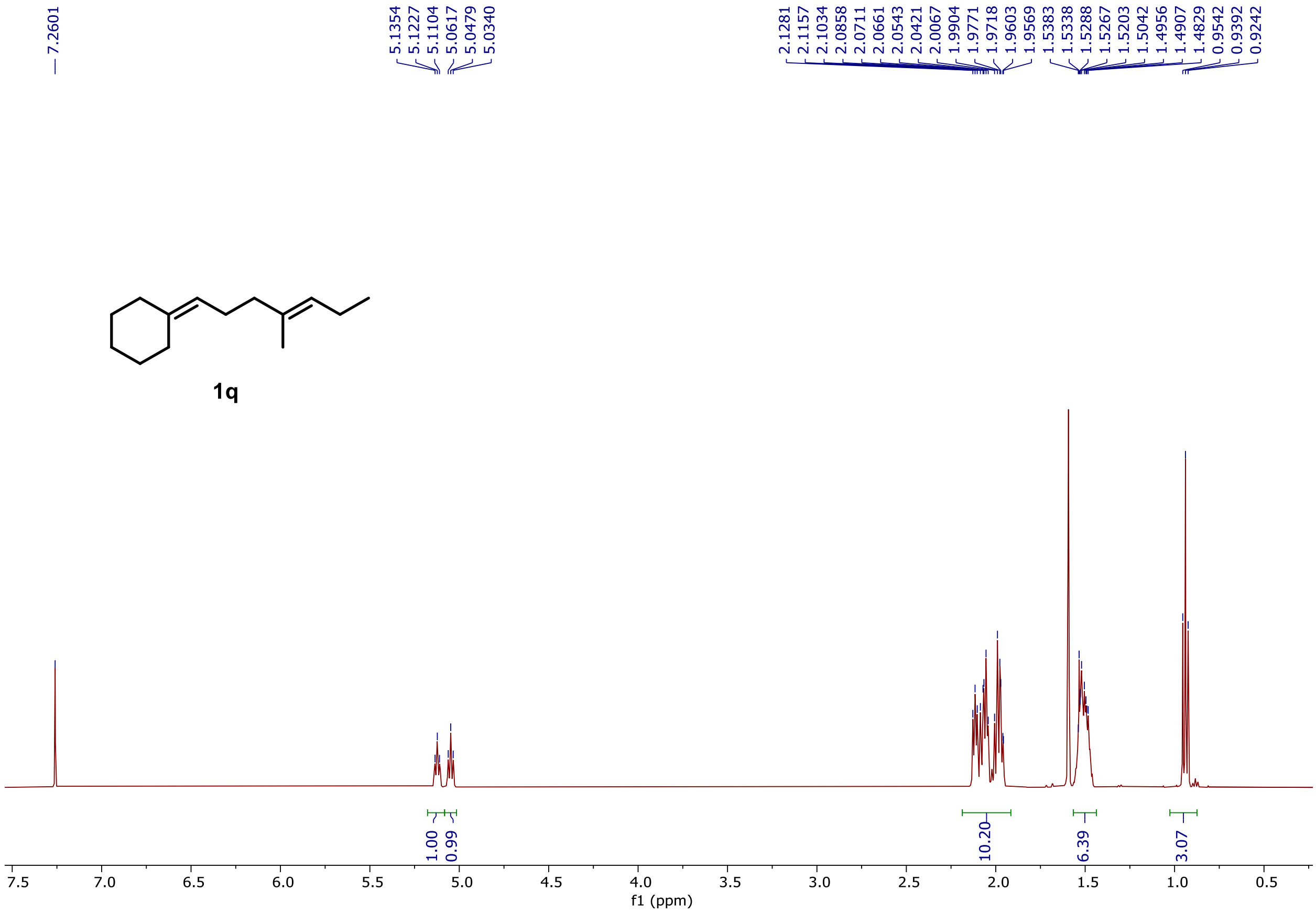

^{13}C NMR spectrum of **1q**

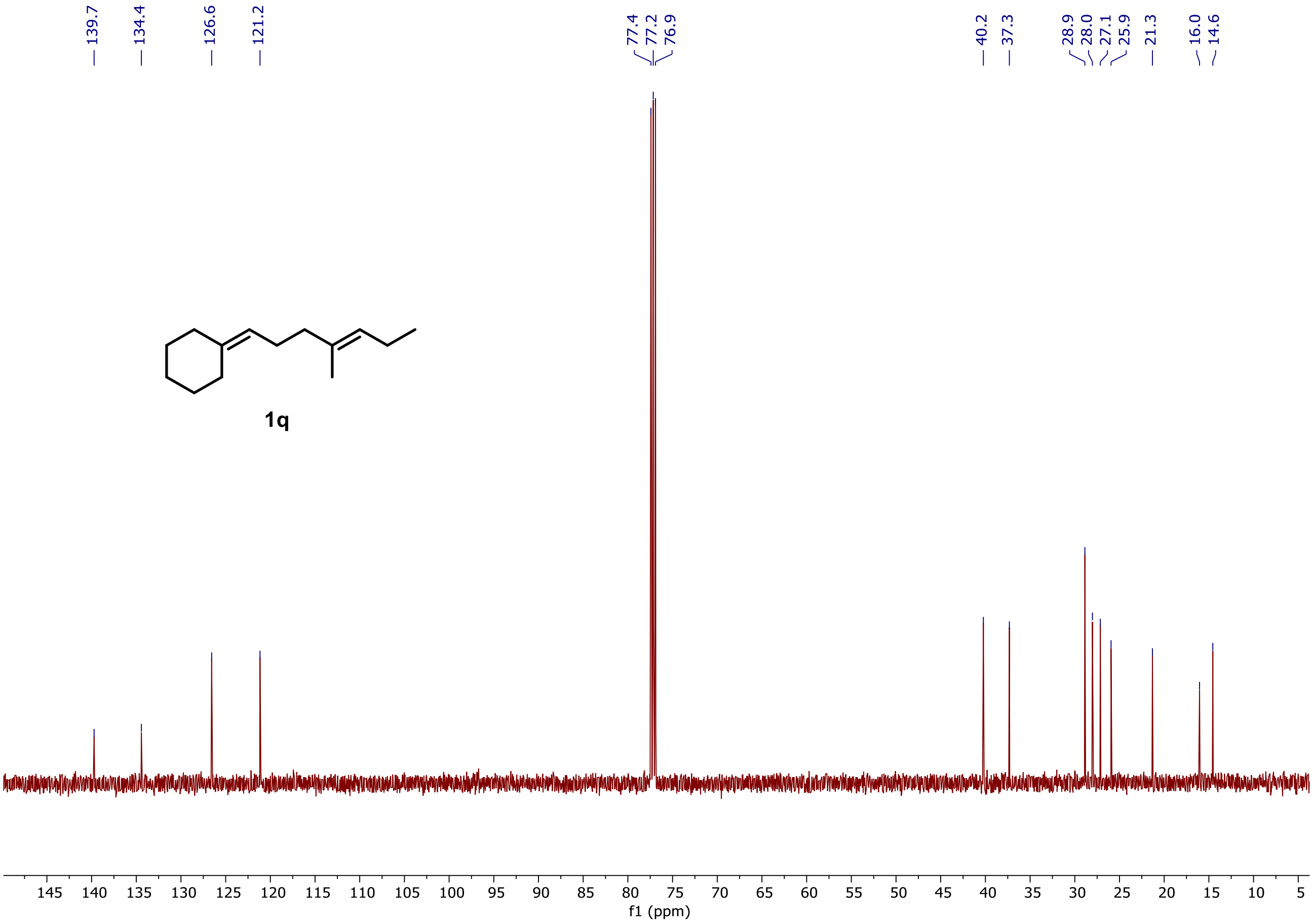

^{1}H NMR spectrum of **2a**

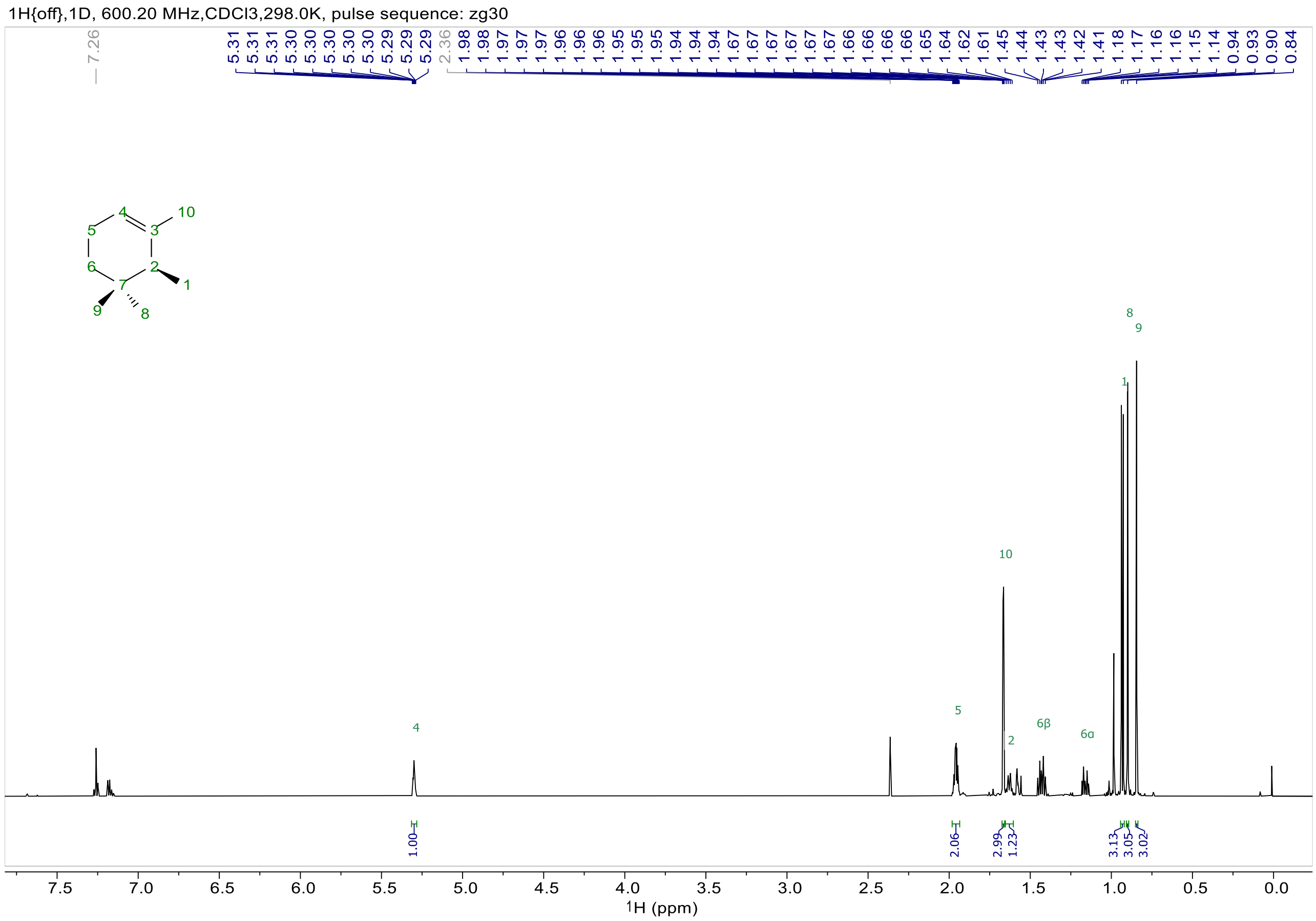

^{13}C NMR spectrum of **2a**

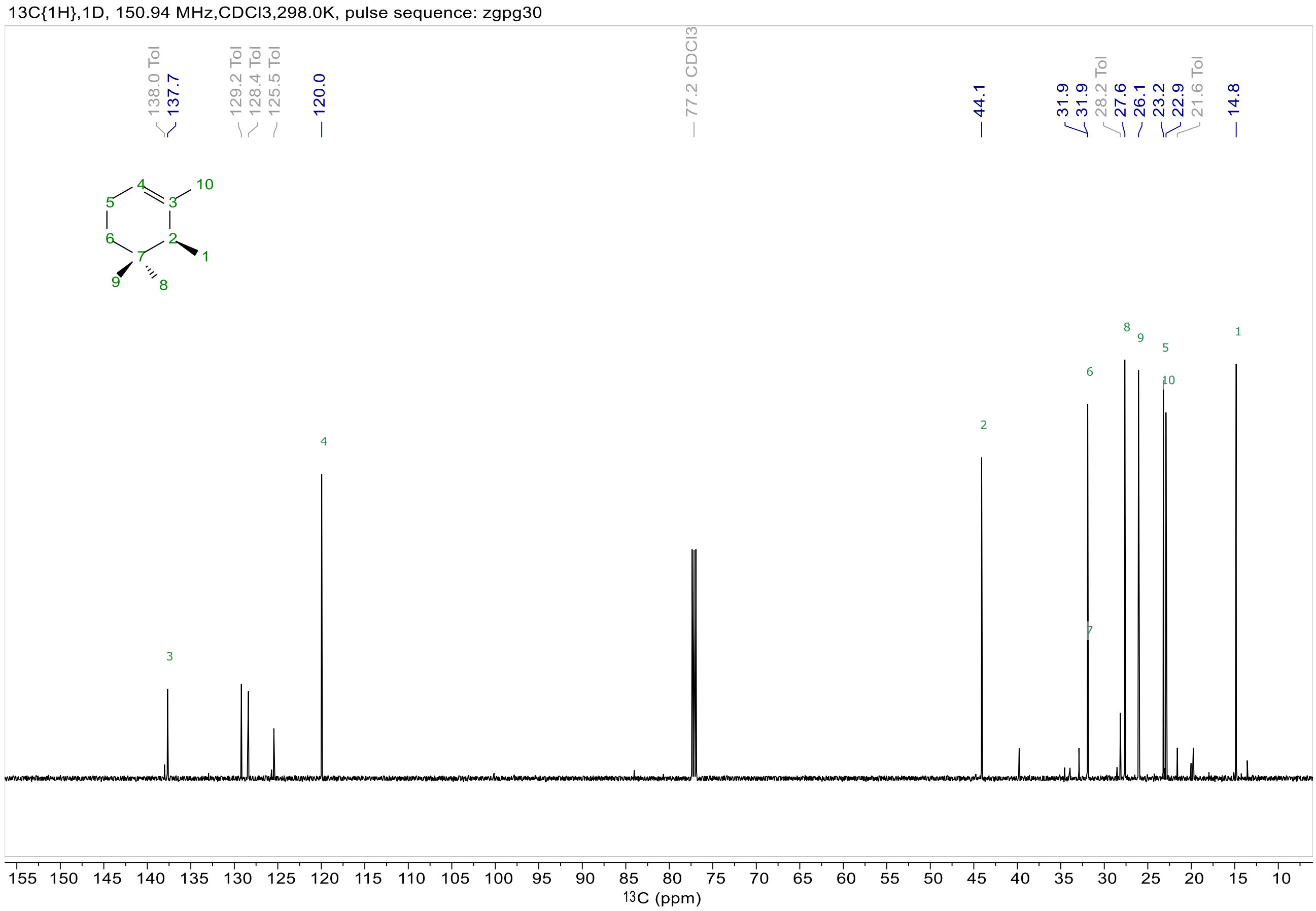

^{1}H NMR spectrum of **2b**

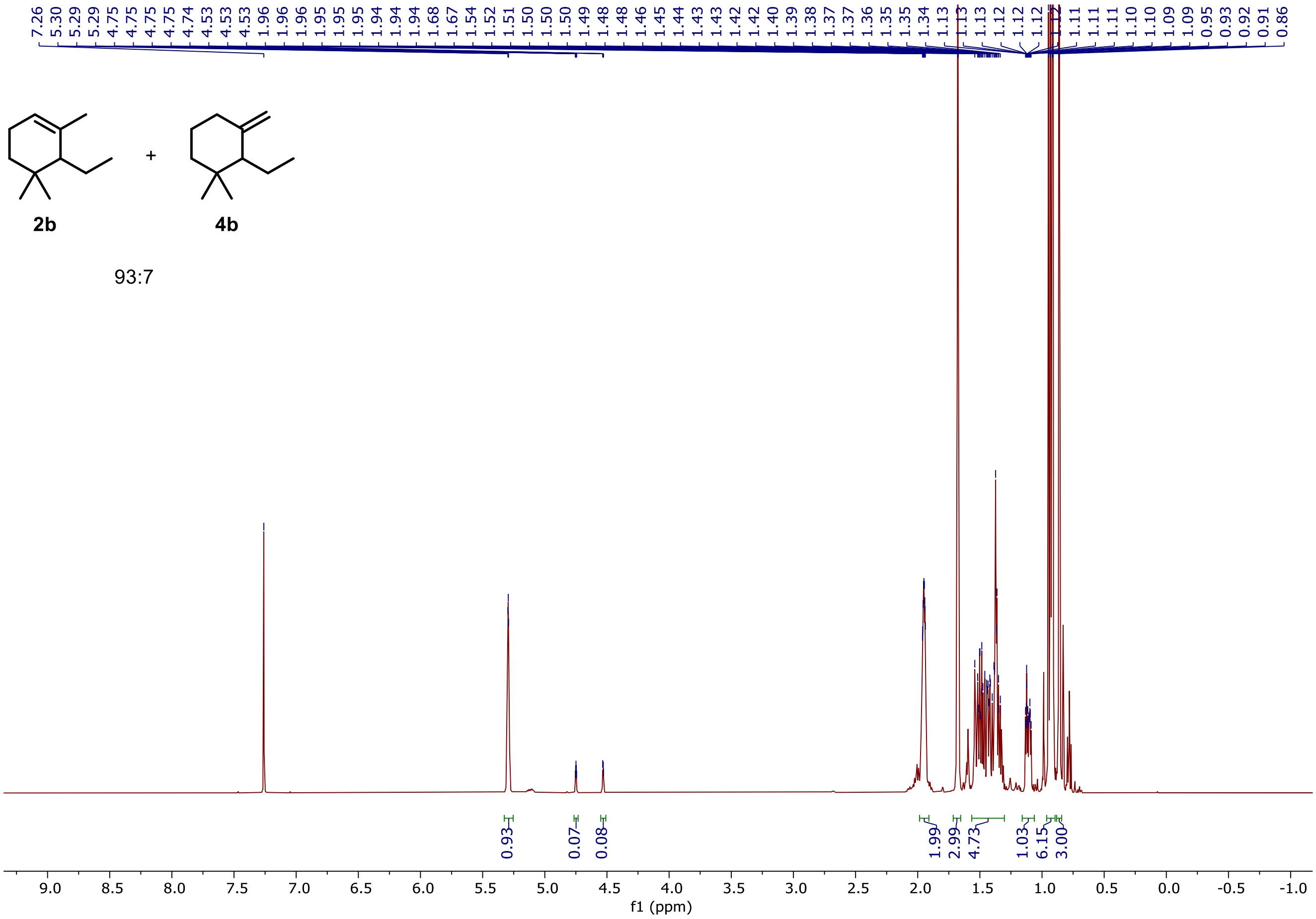

^{13}C NMR spectrum of **2b**

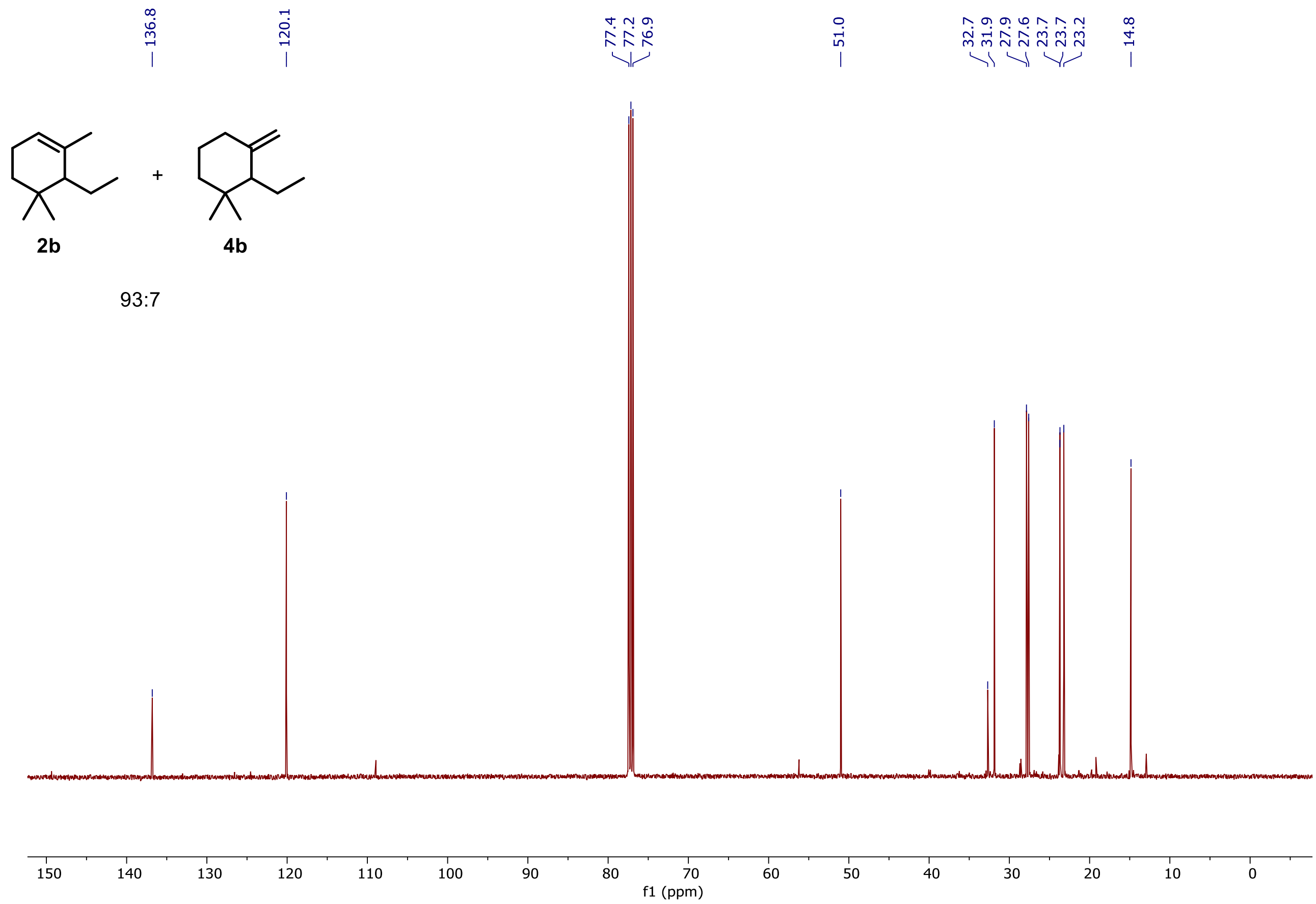

^{1}H NMR spectrum of **2c**

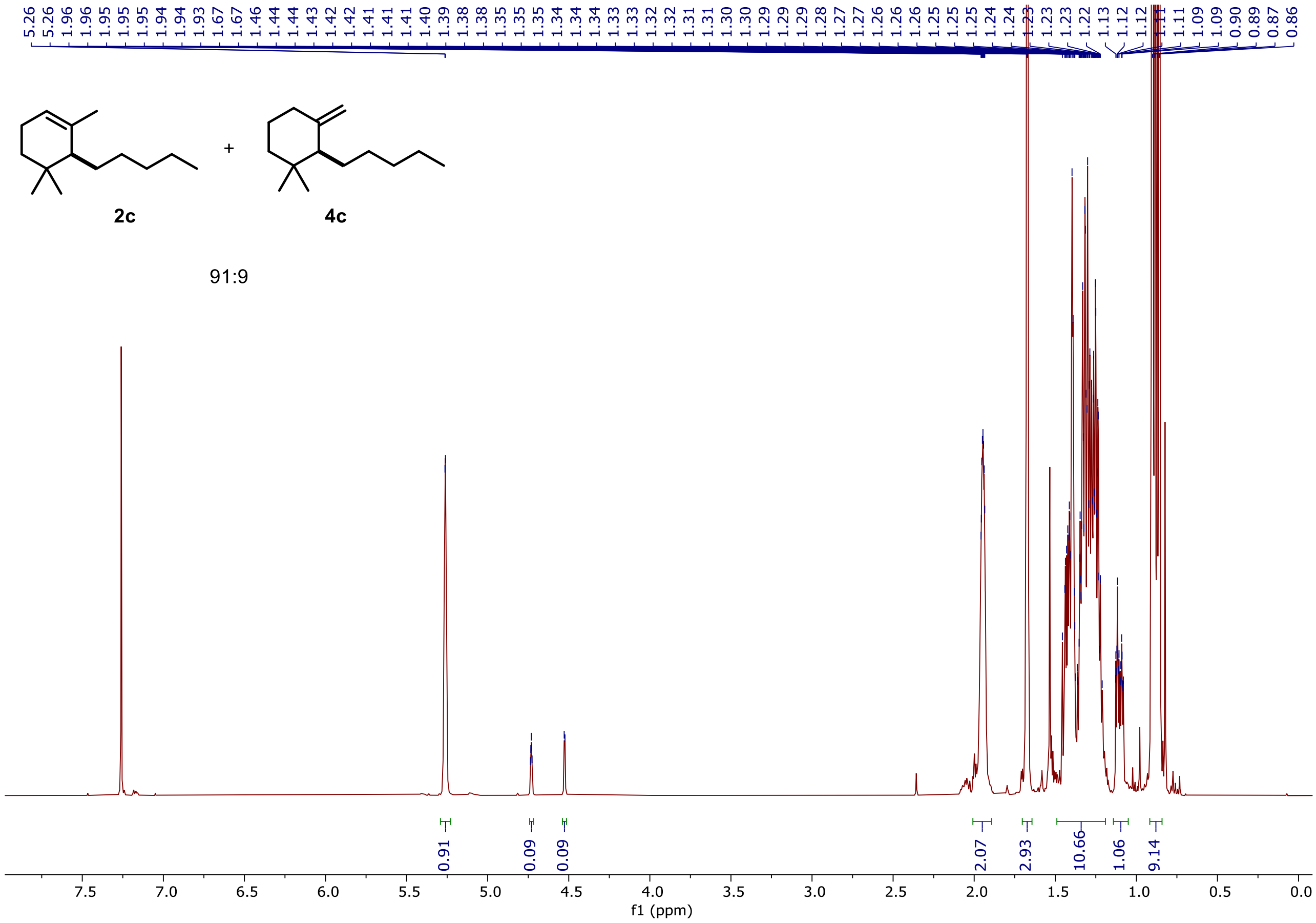

^{13}C NMR spectrum of **2c**

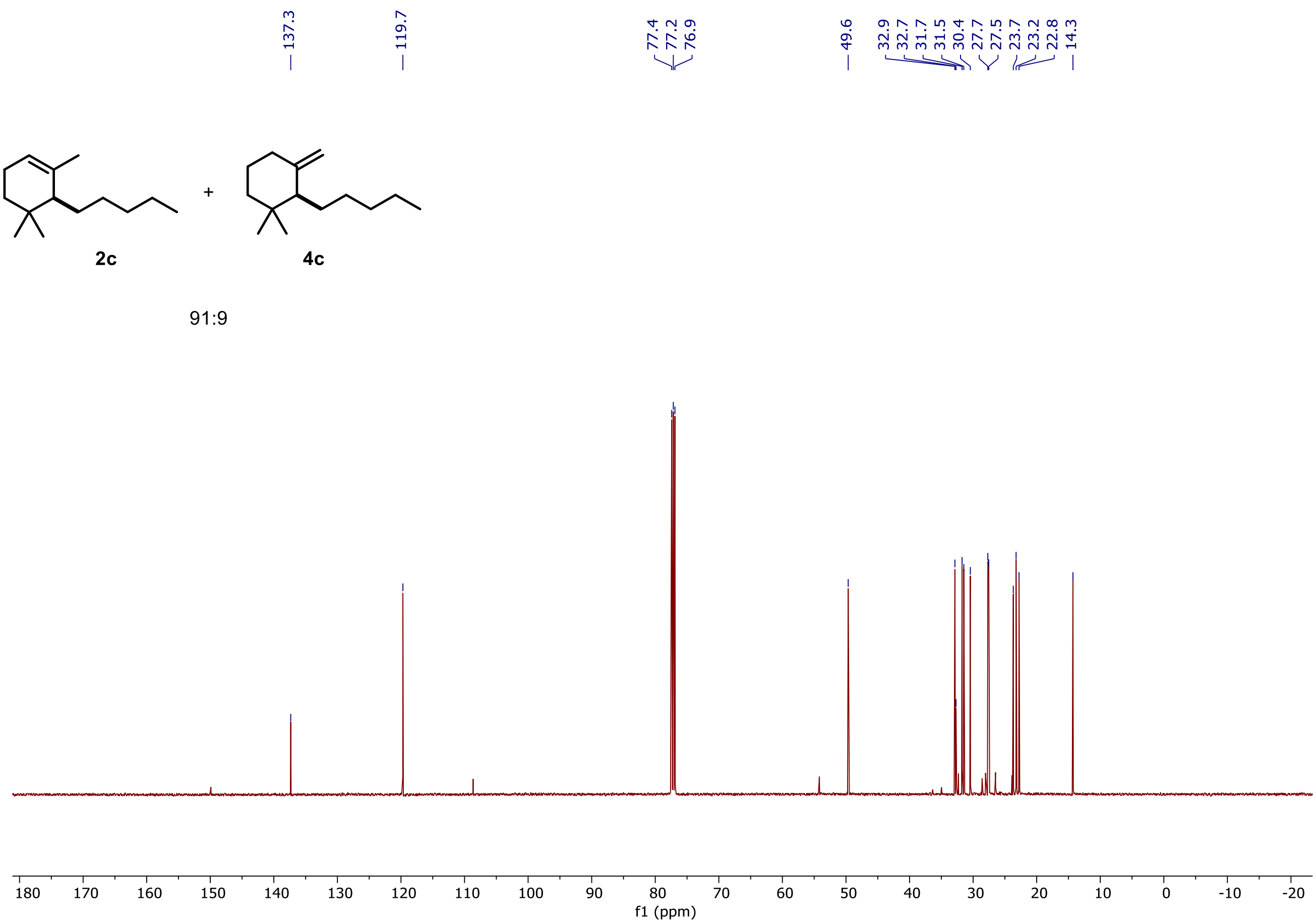

^{1}H NMR spectrum of **2d**

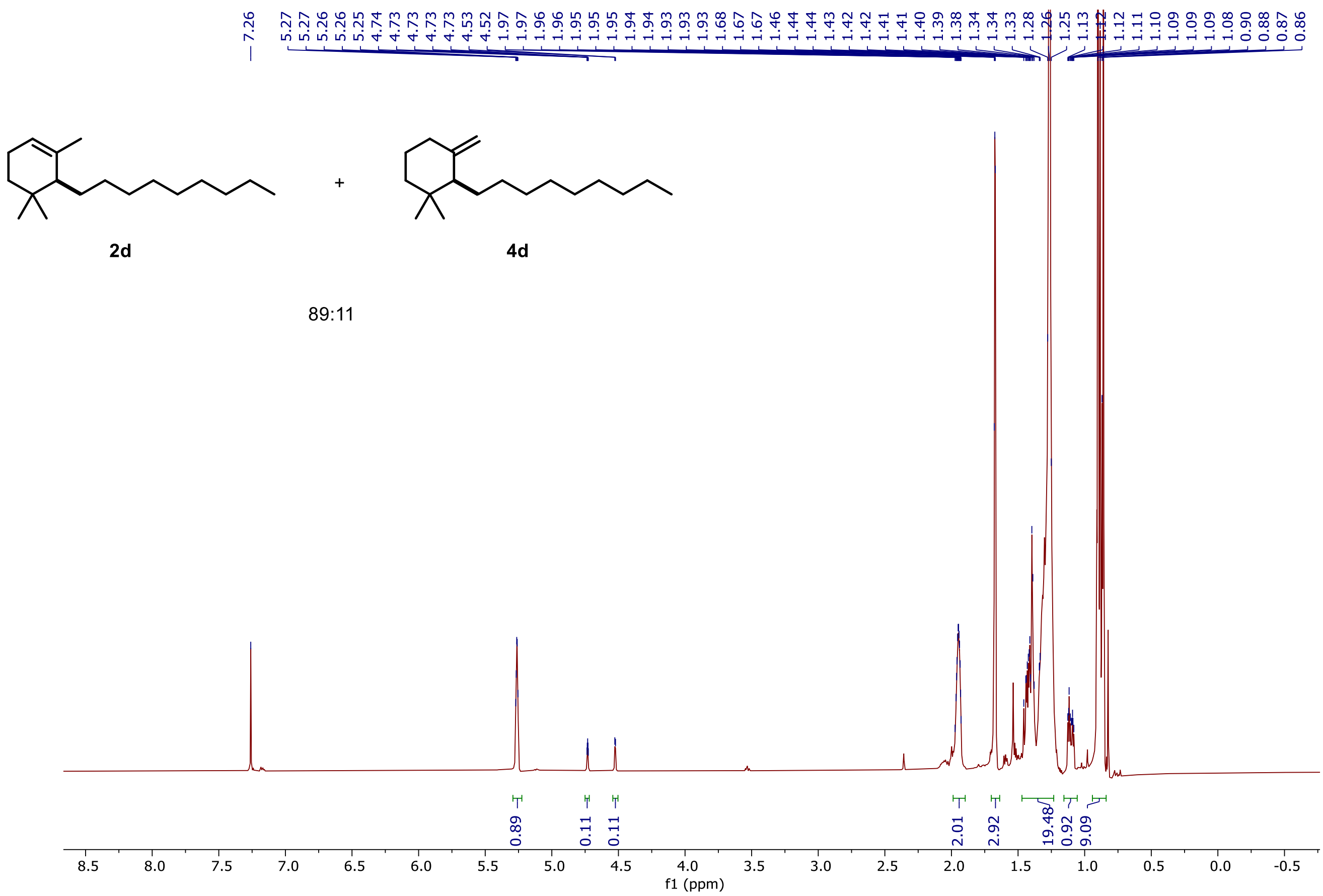

^{13}C NMR spectrum of **2d**

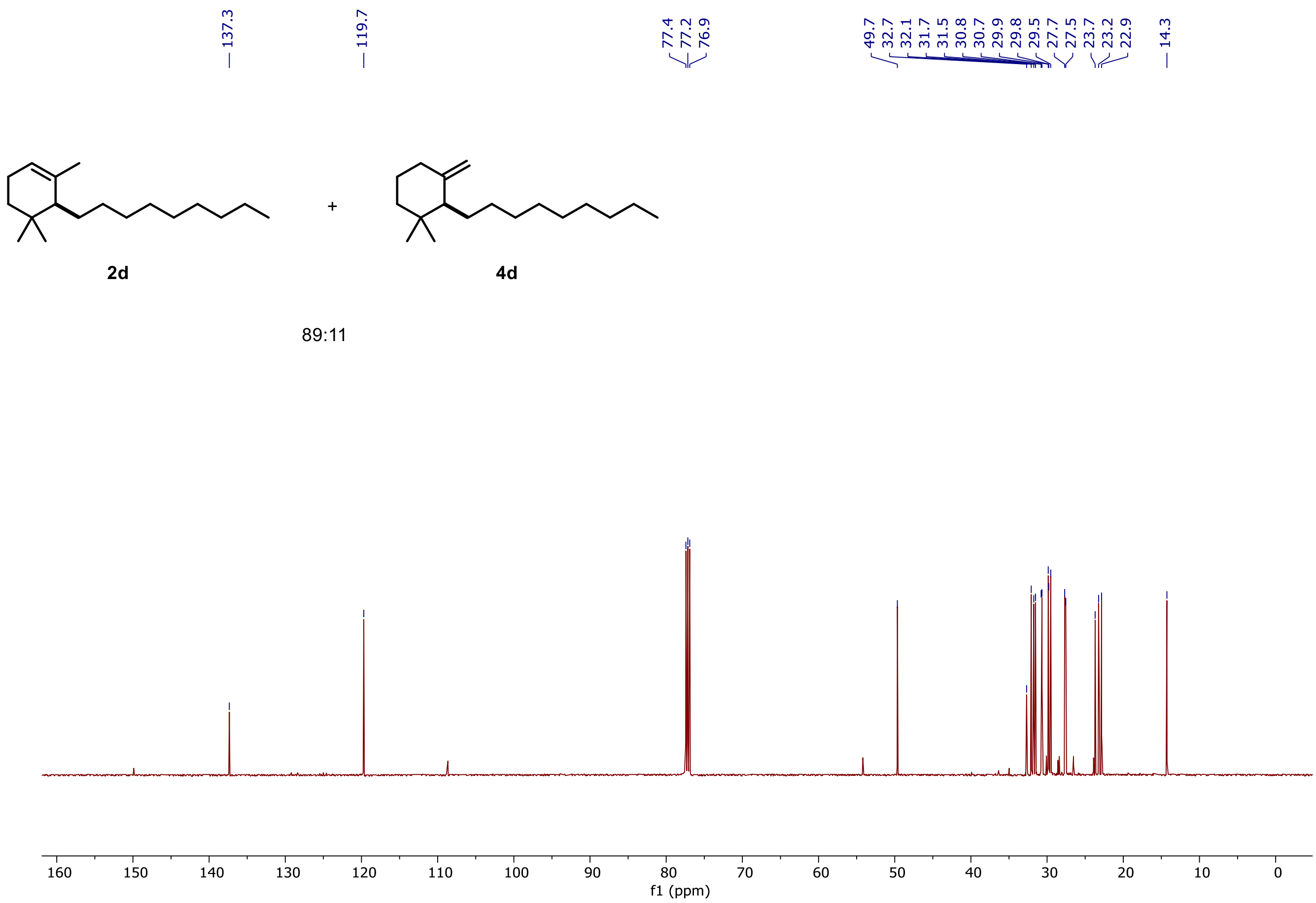

^{1}H NMR spectrum of **2e**

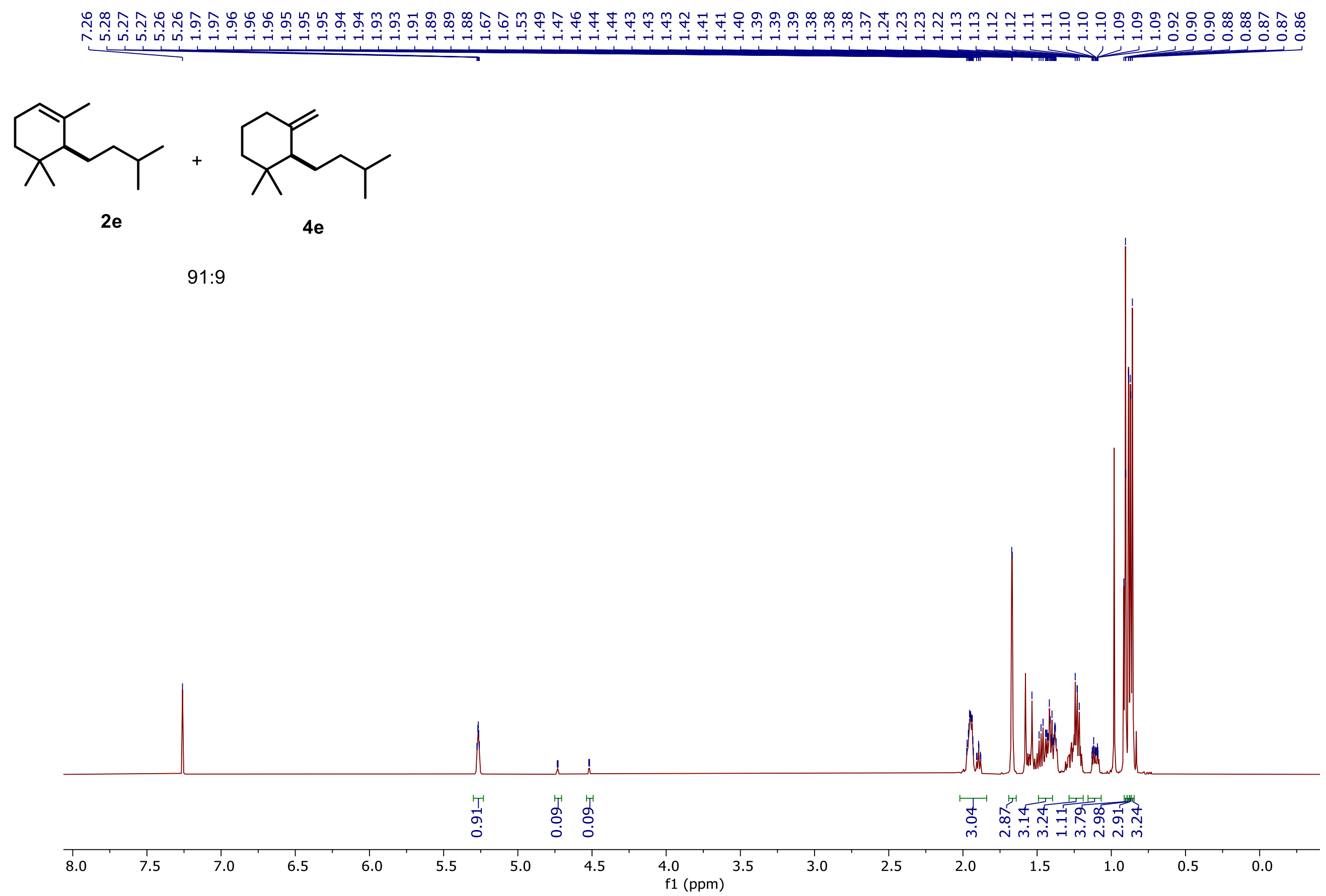

^{13}C NMR spectrum of **2e**

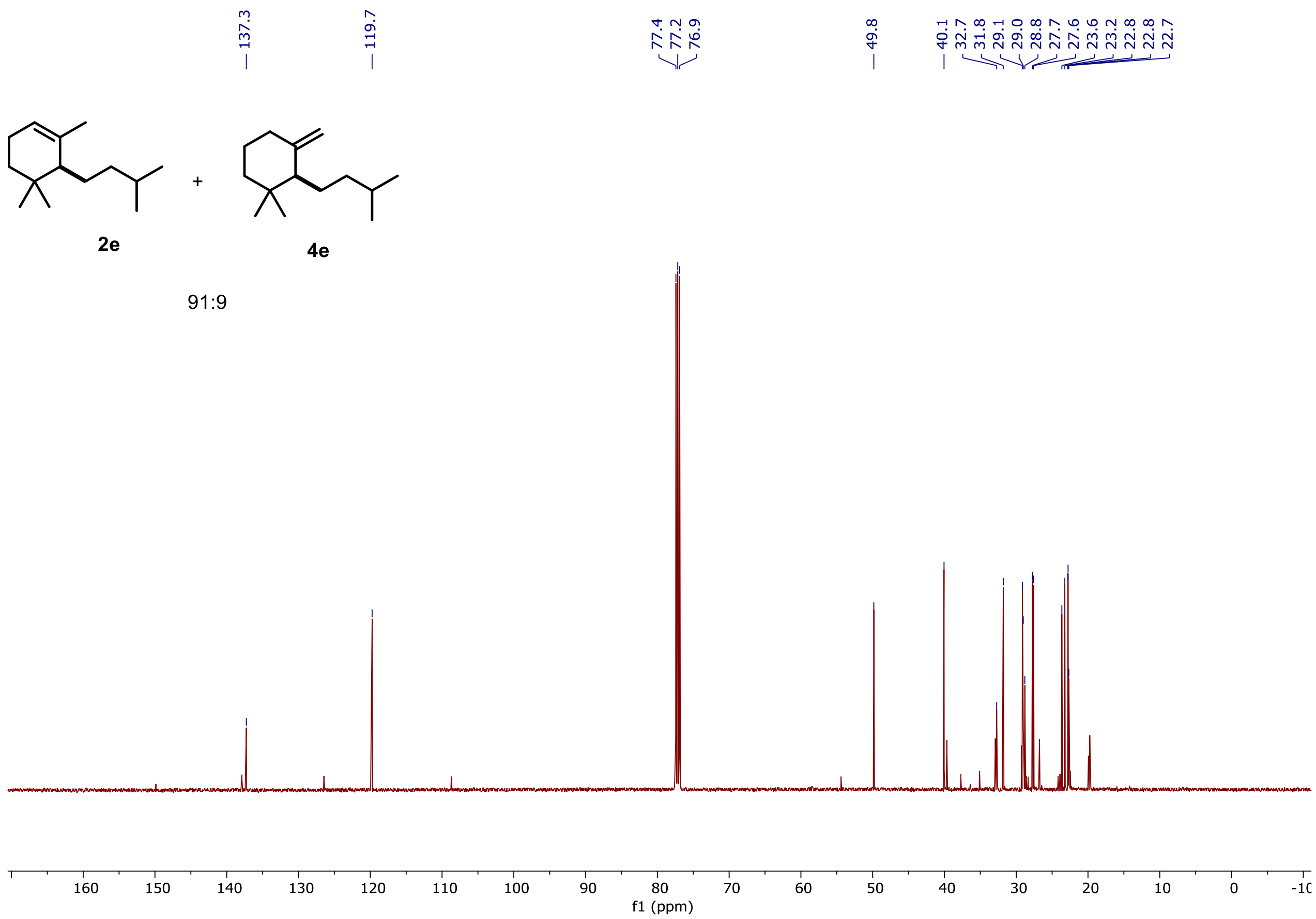

^{1}H NMR spectrum of **2f**

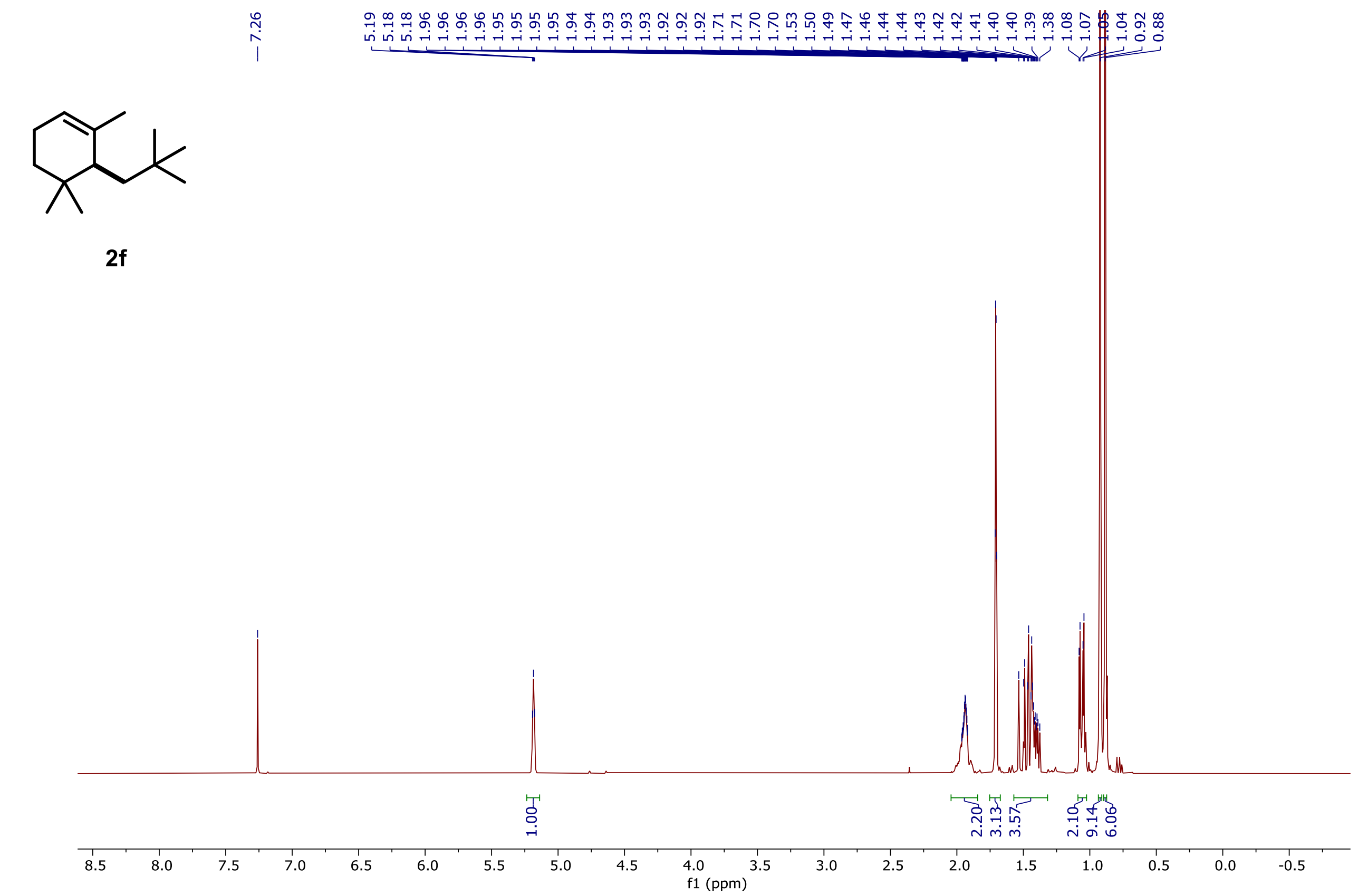

^{13}C NMR spectrum of **2f**

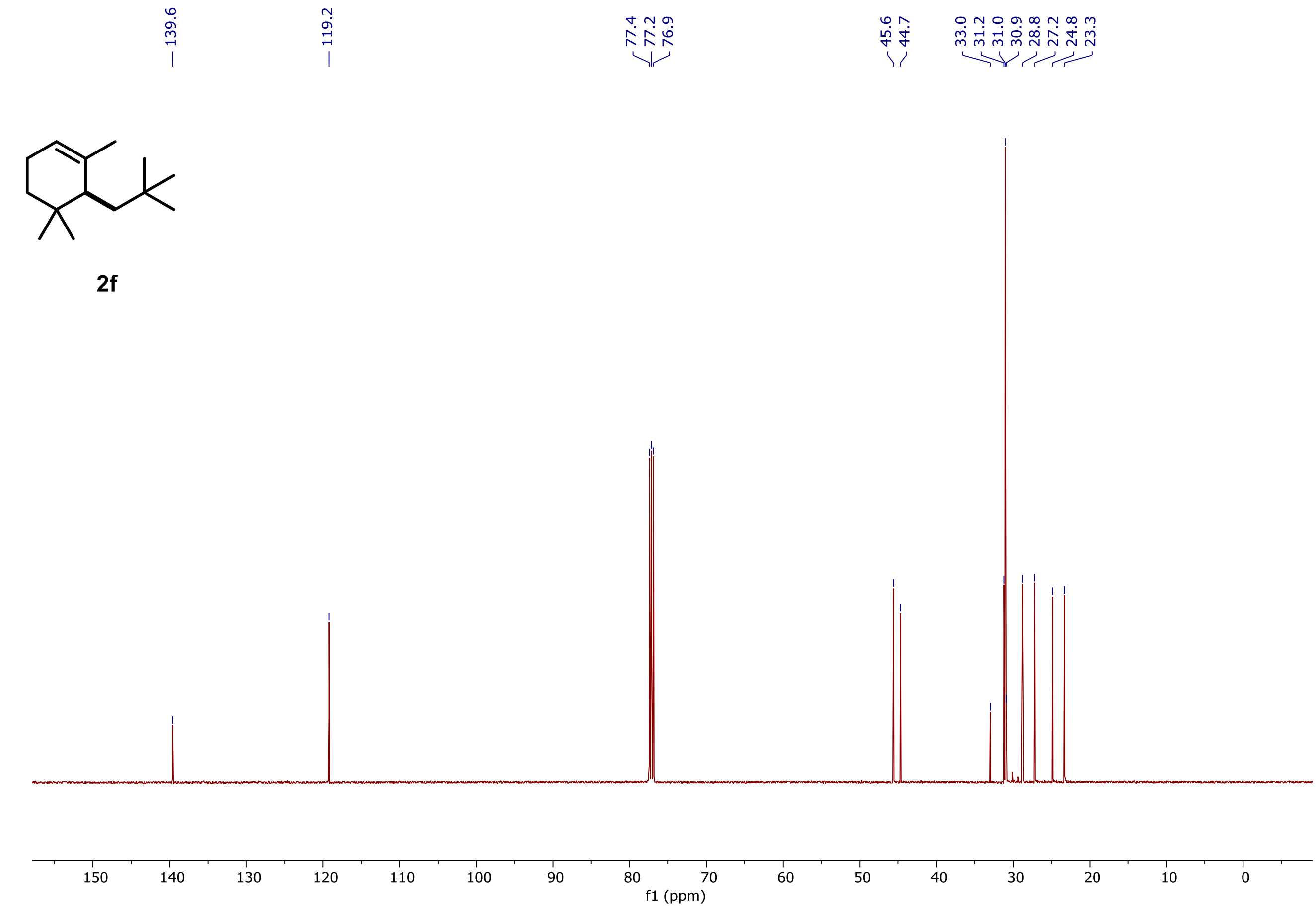

^{1}H NMR spectrum of **2g**

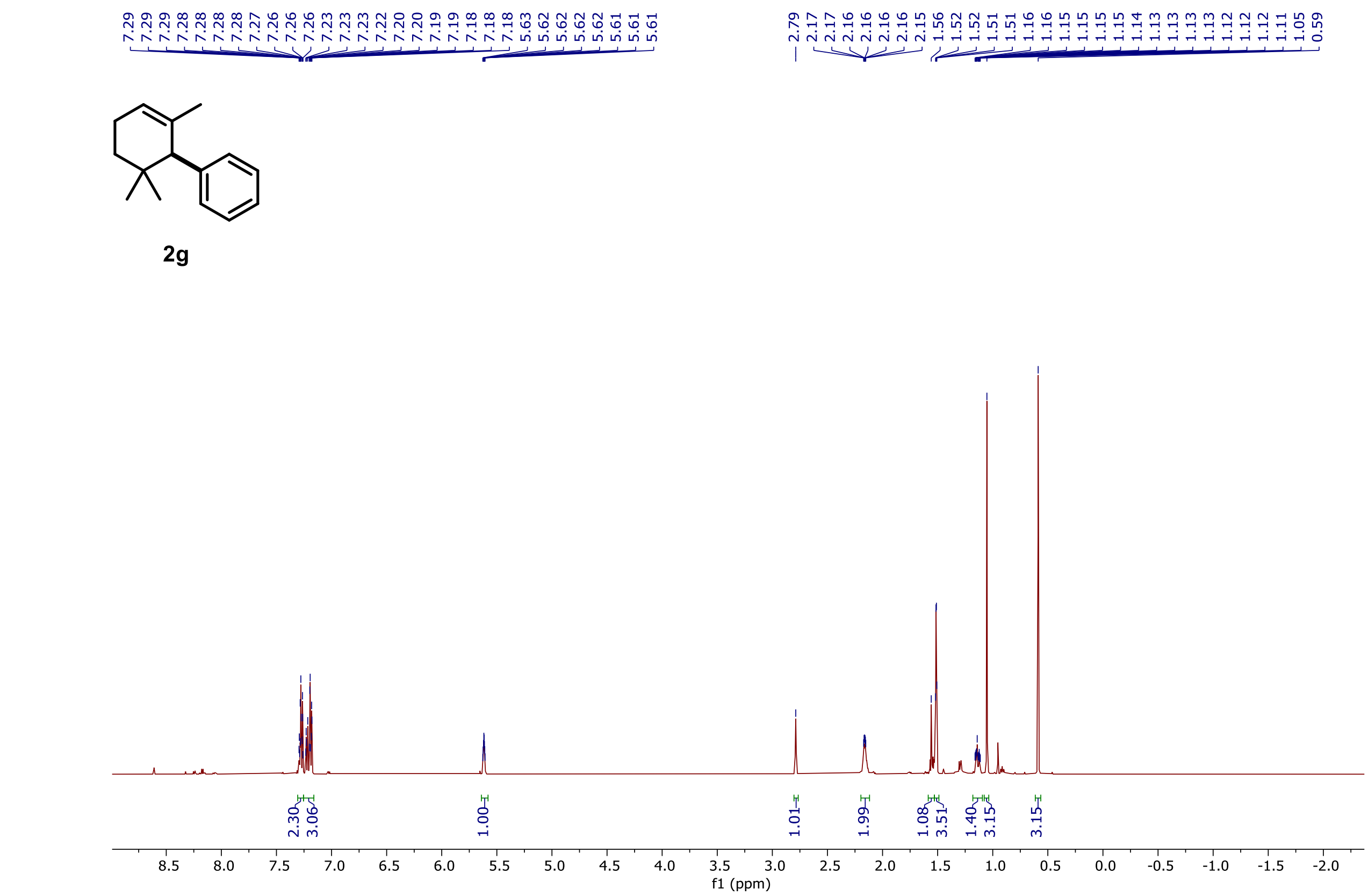

^{13}C NMR spectrum of **2g**

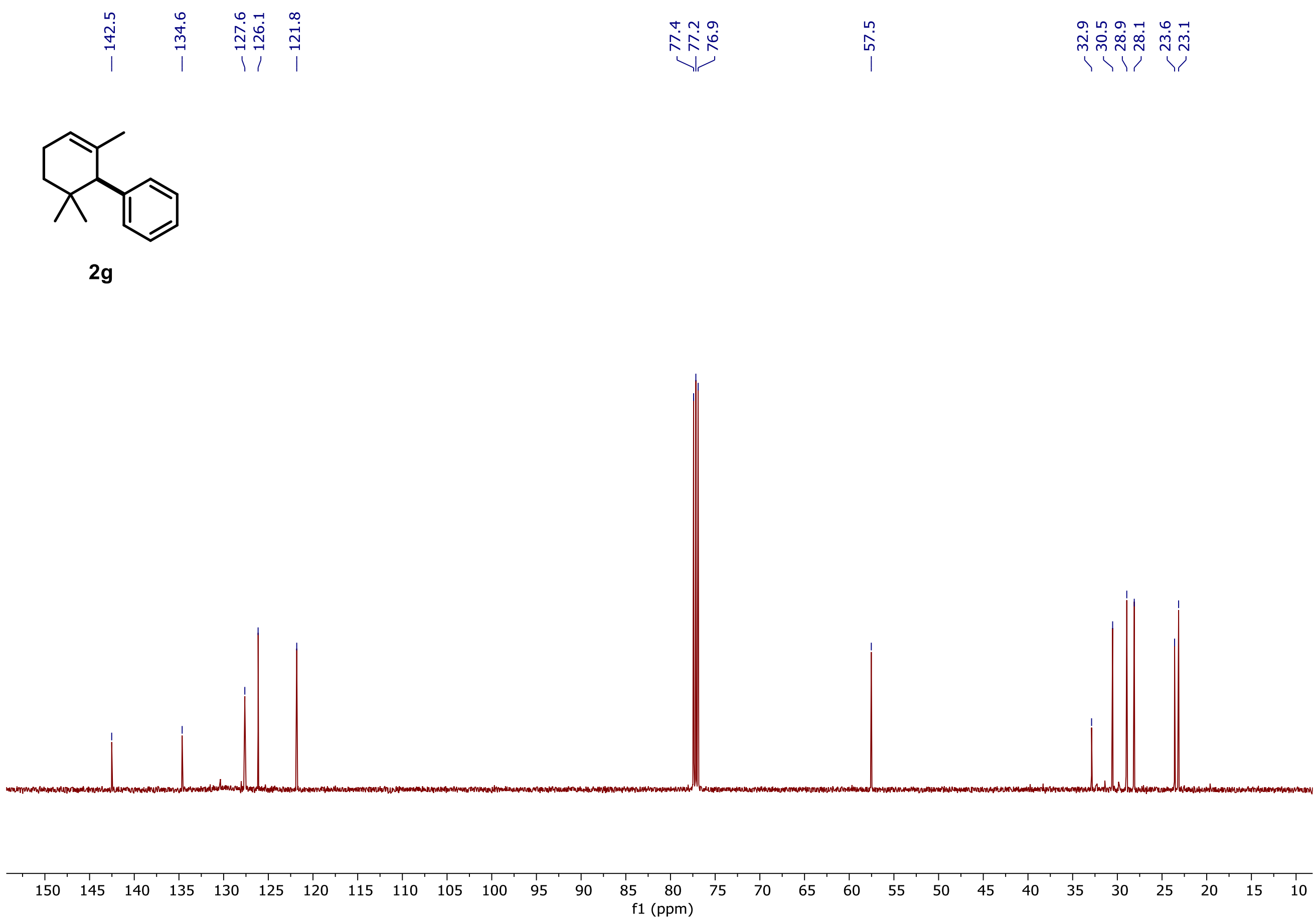

^{1}H NMR spectrum of **2h**

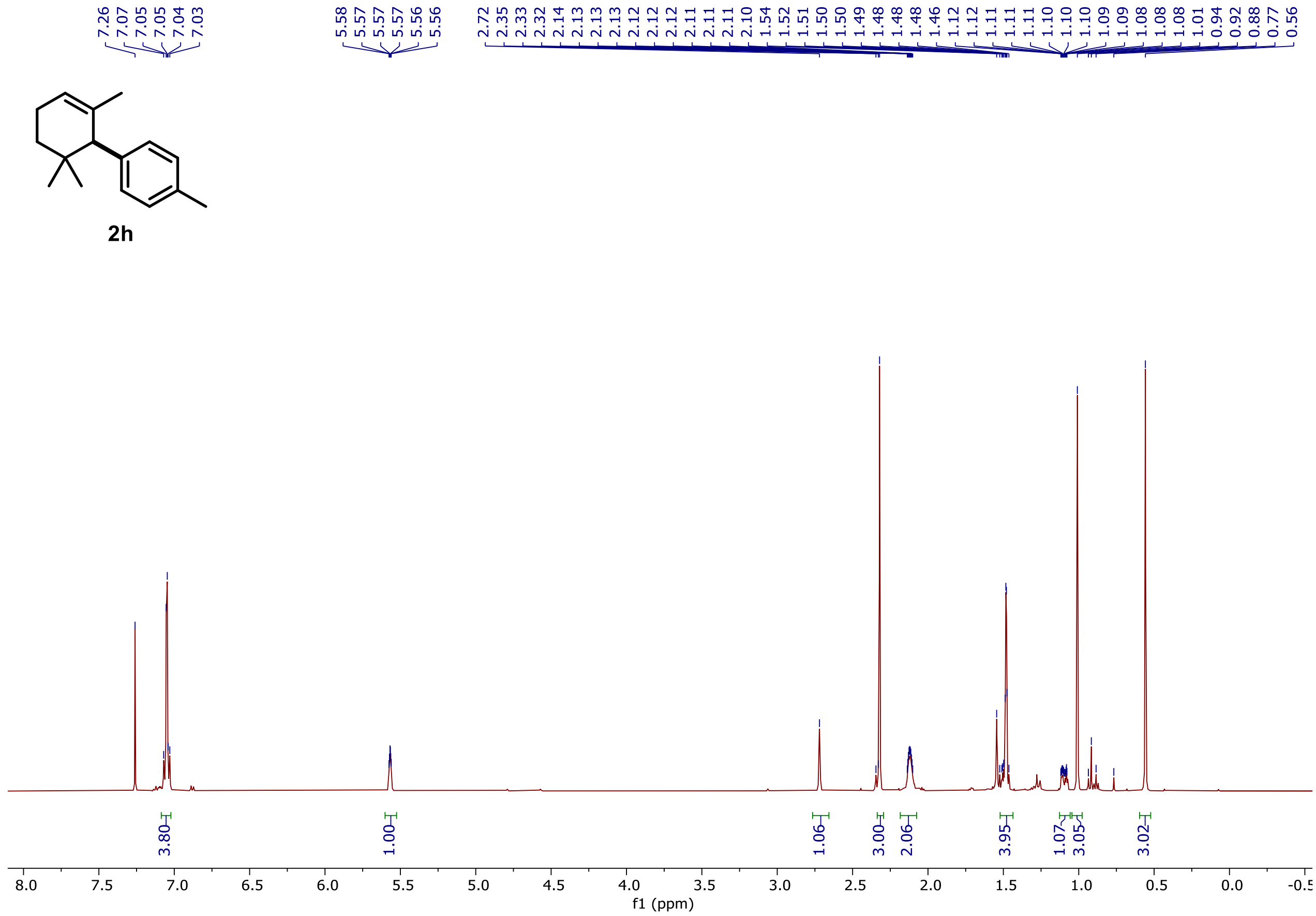

^{13}C NMR spectrum of **2h**

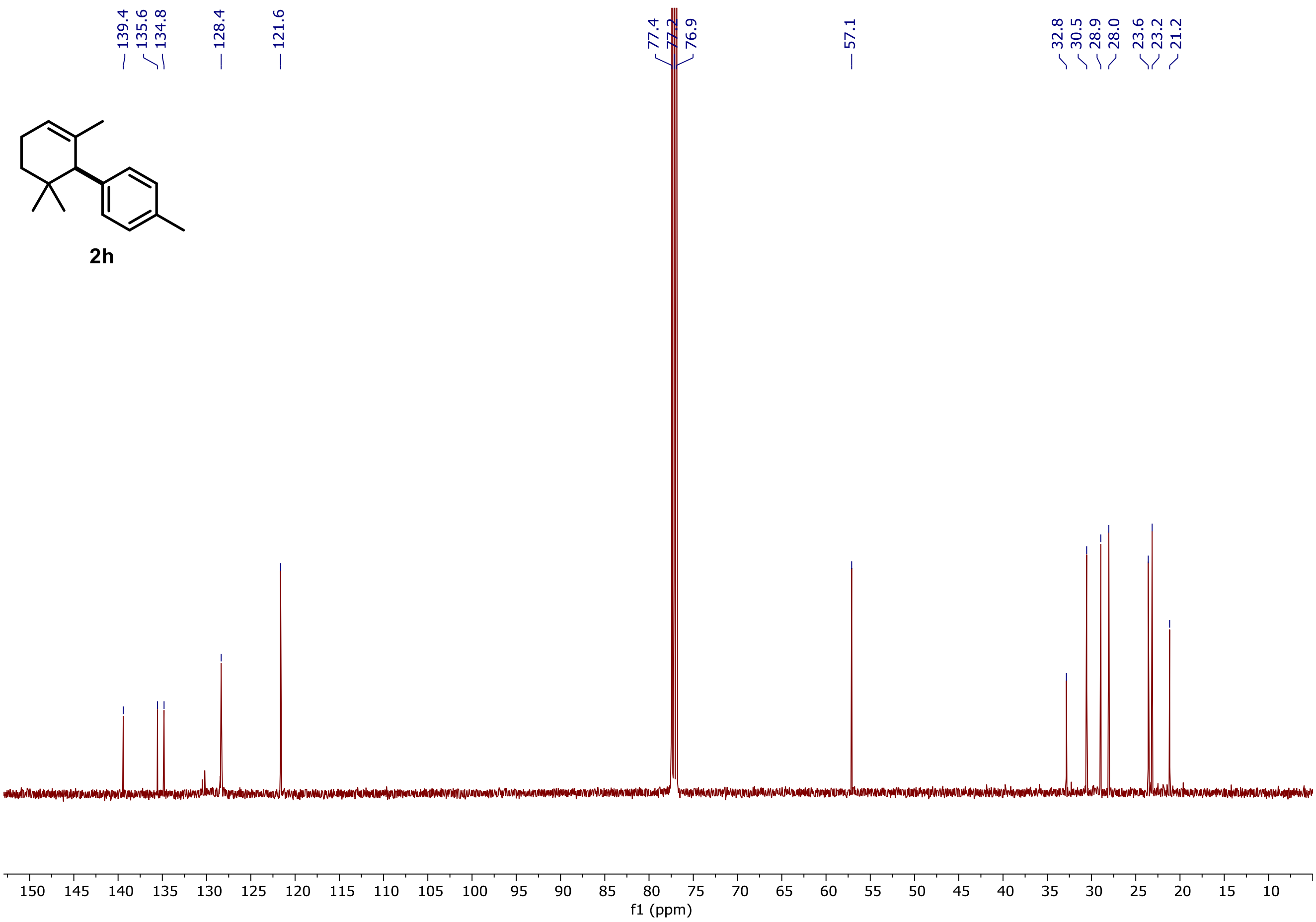

^{1}H NMR spectrum of **2i**

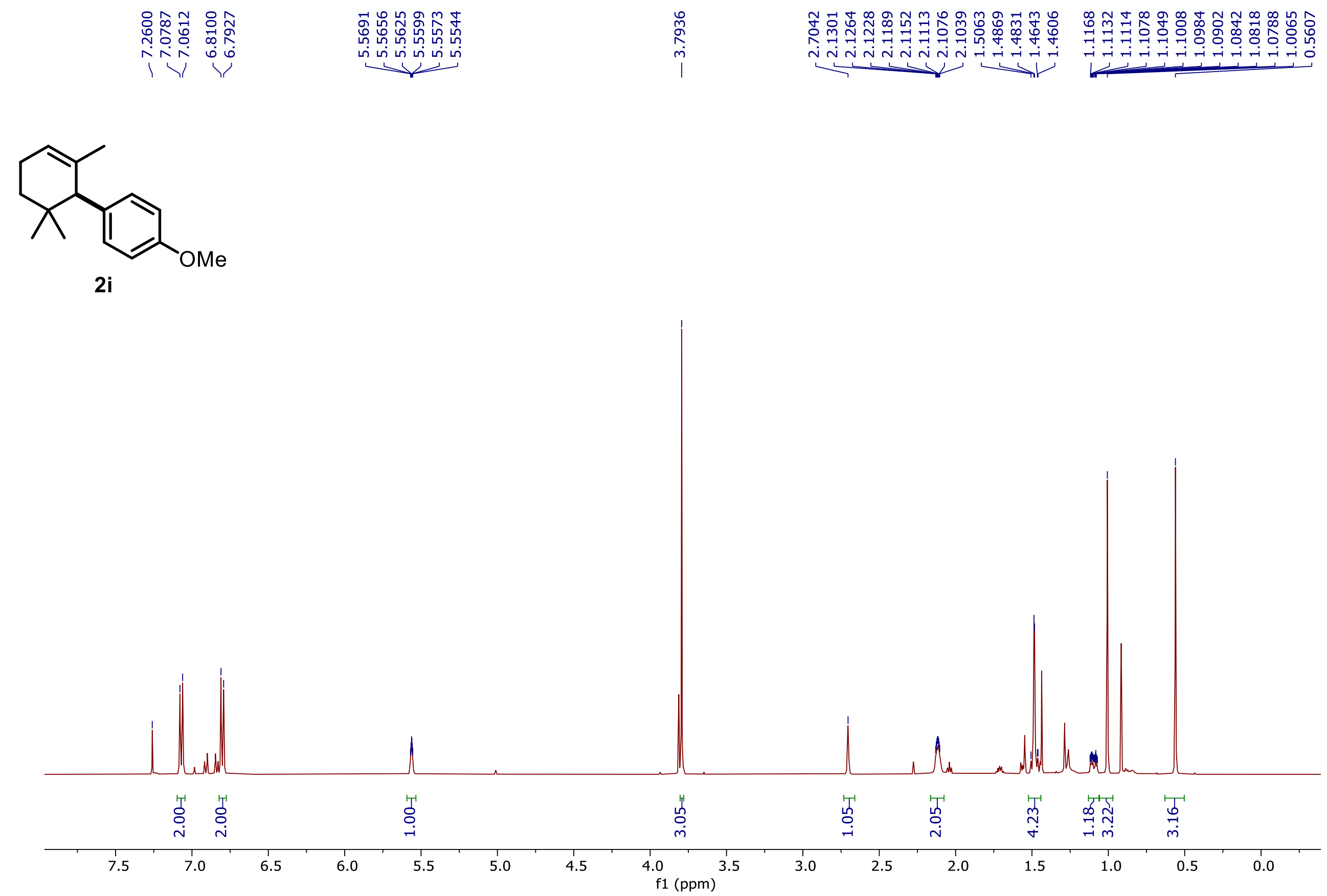

^{13}C NMR spectrum of **2i**

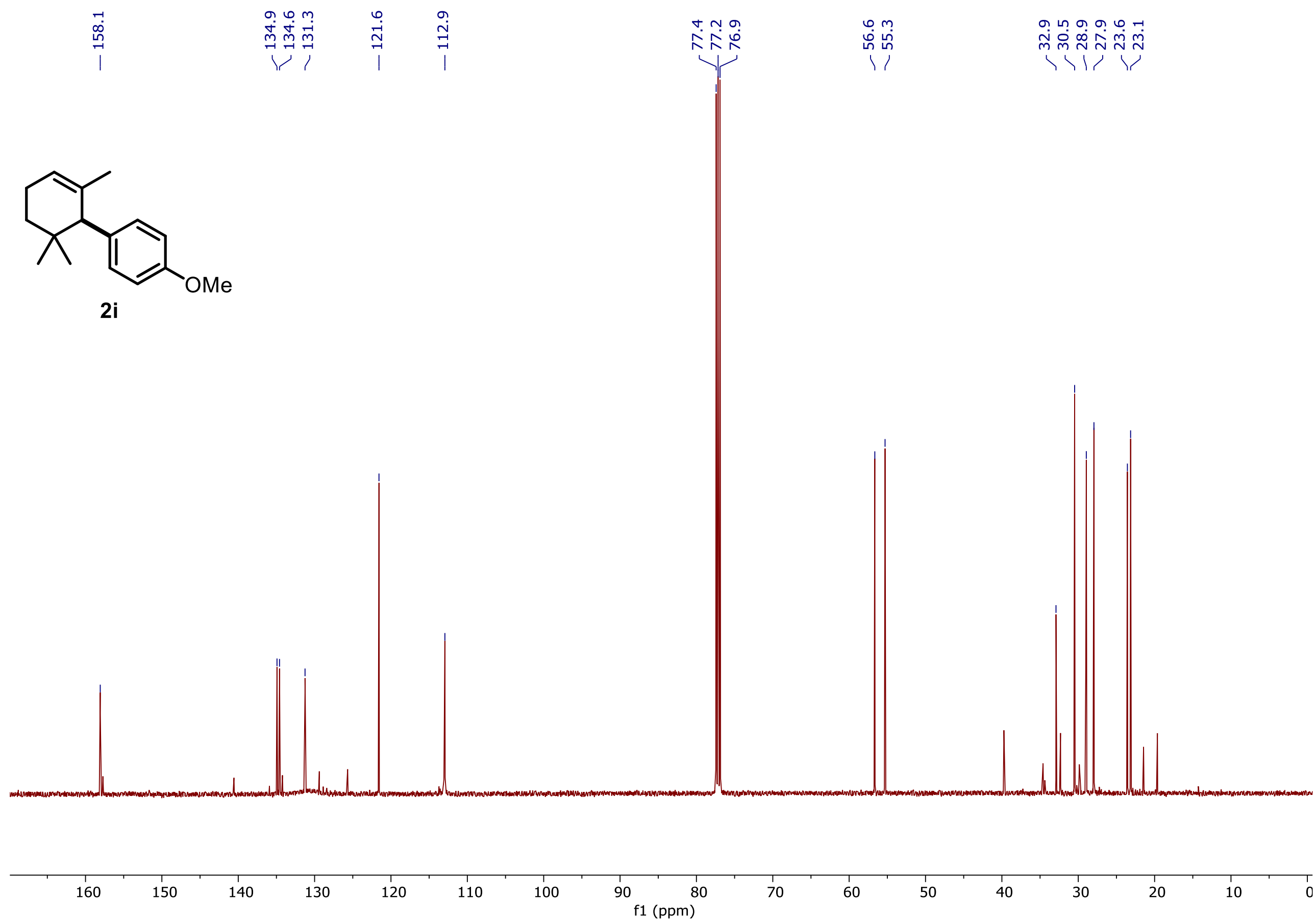

^{1}H NMR spectrum of **2j**

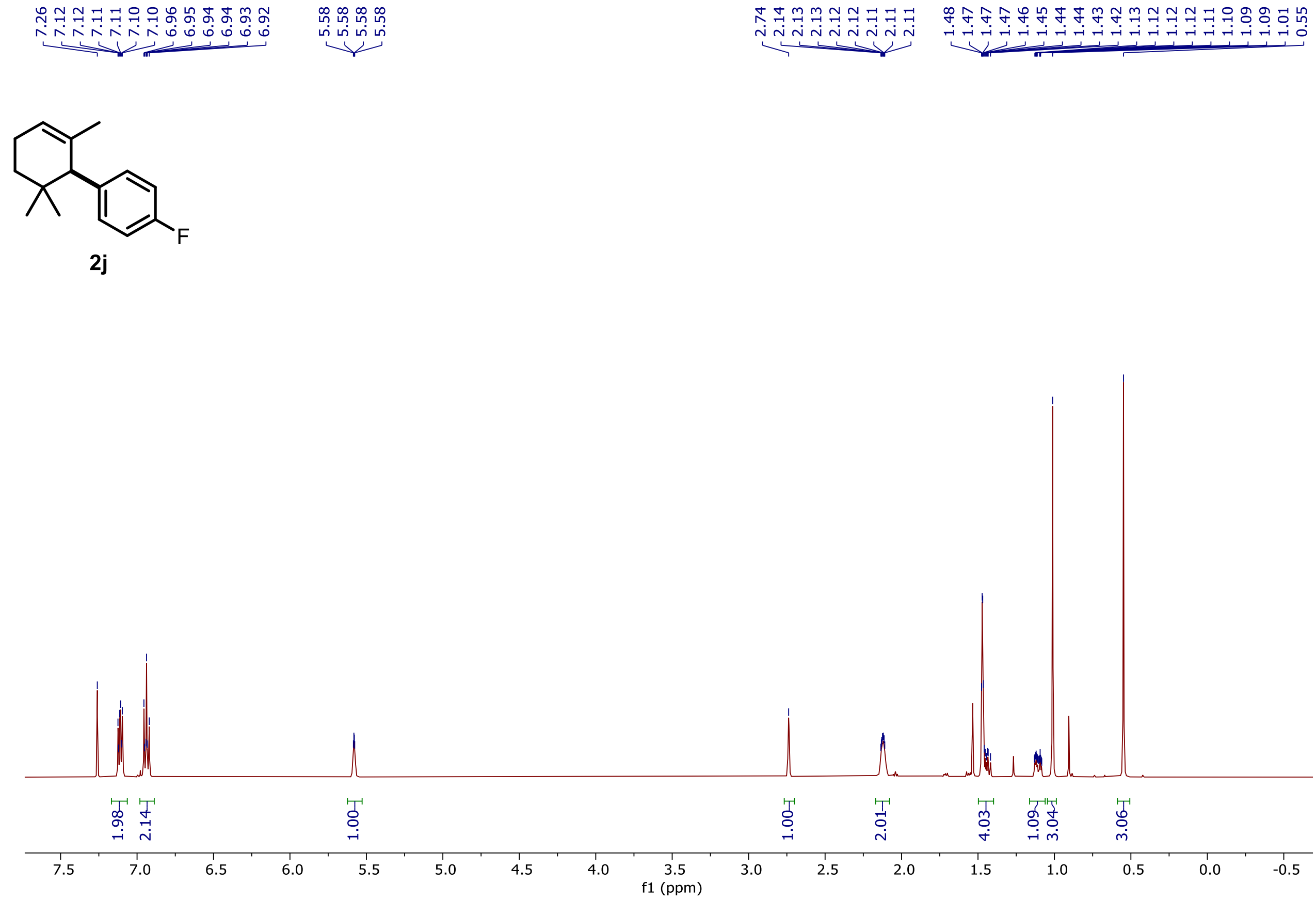

^{13}C NMR spectrum of **2j**

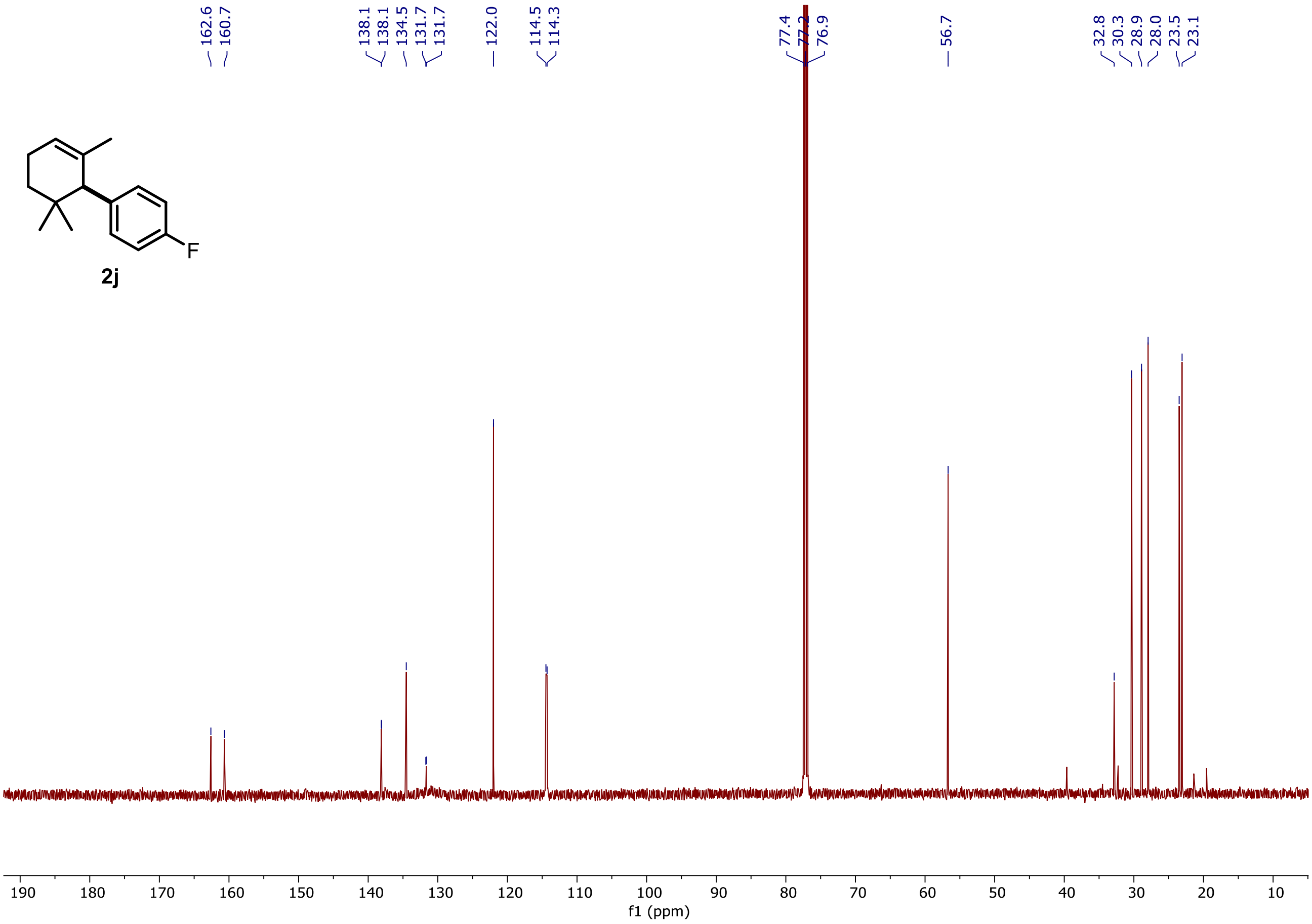

^{19}F NMR spectrum of **2j**

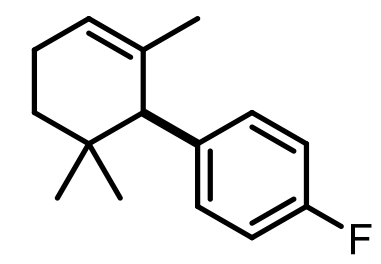

2j

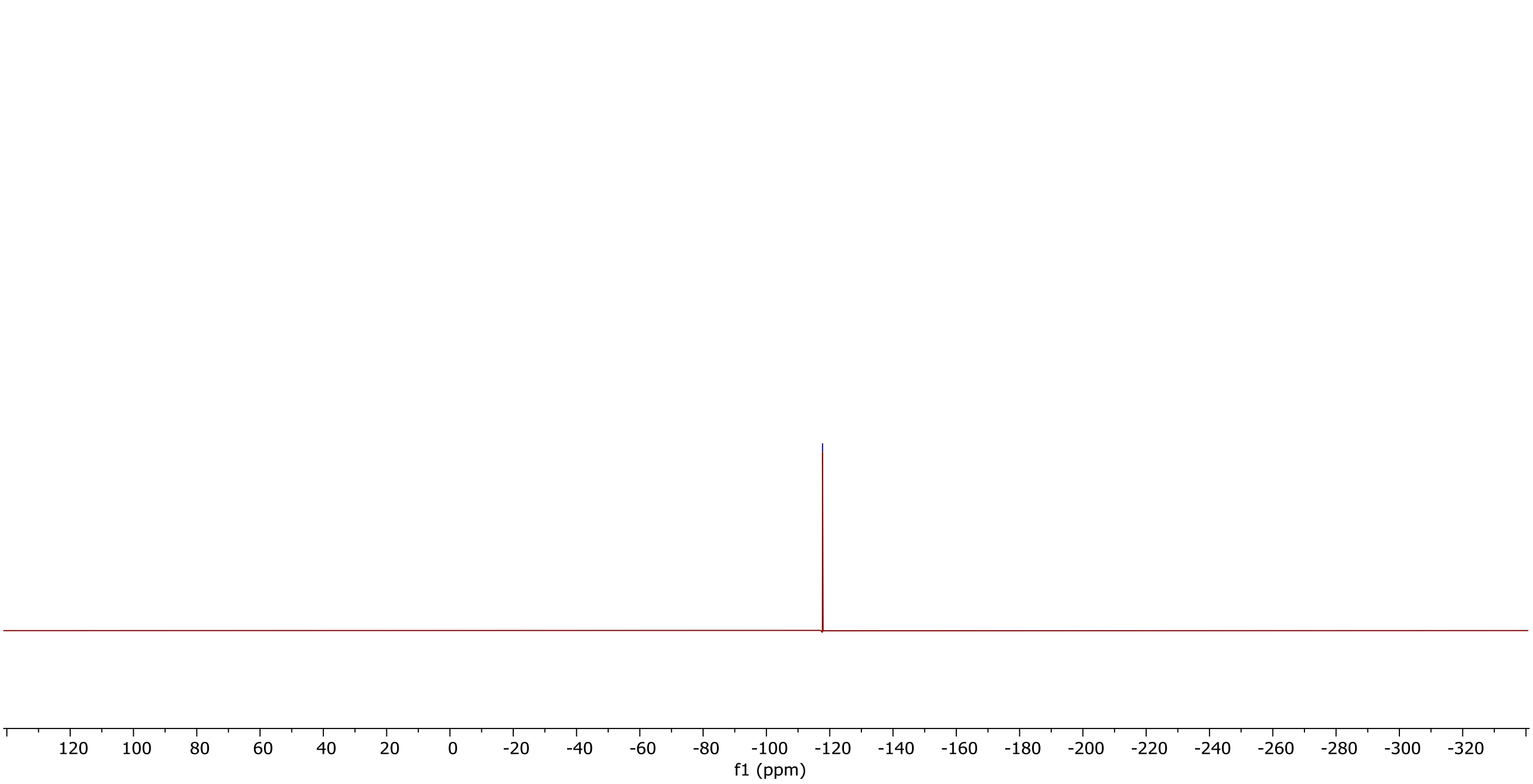

^{1}H NMR spectrum of **2k**

2k

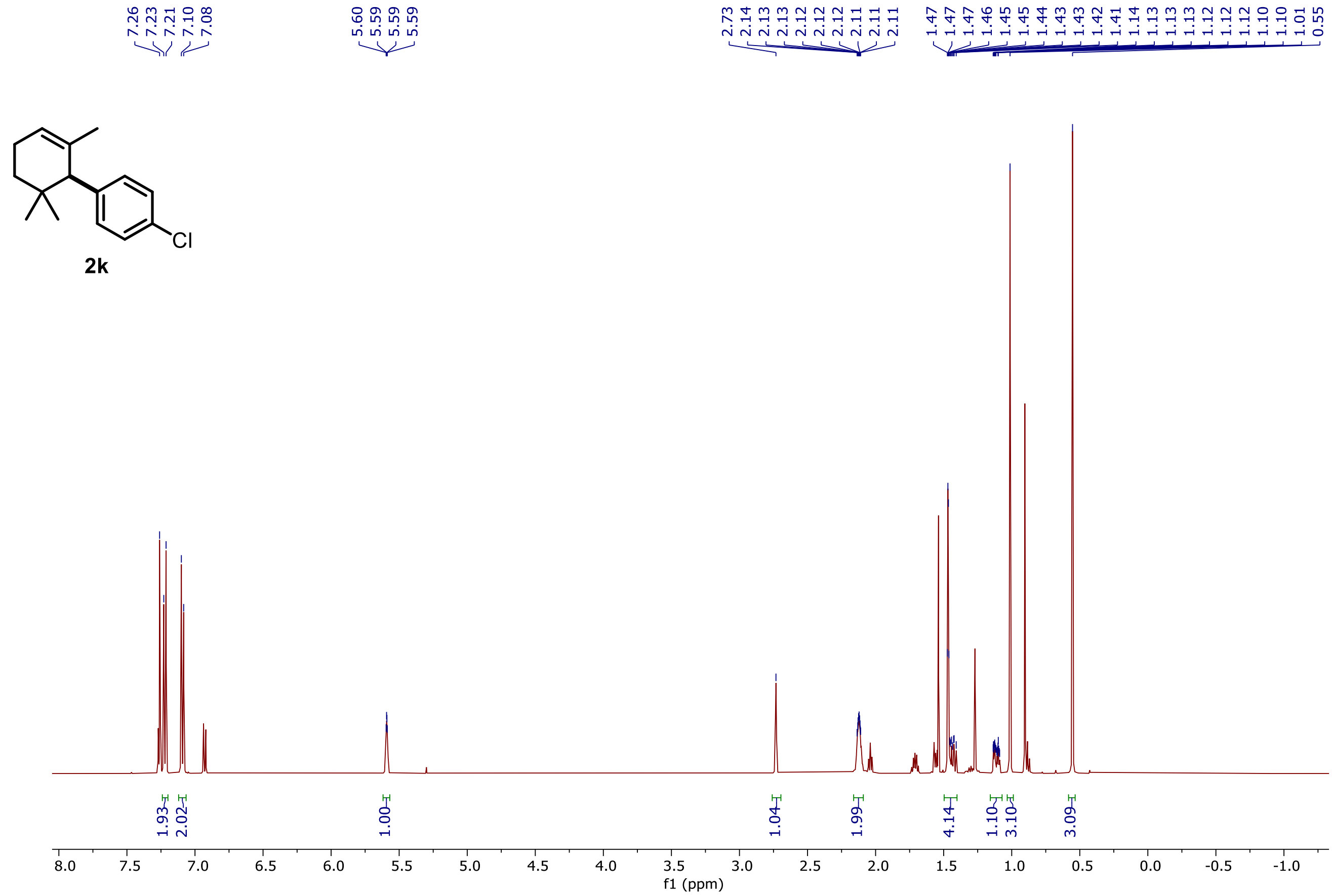

^{13}C NMR spectrum of **2k**

2k

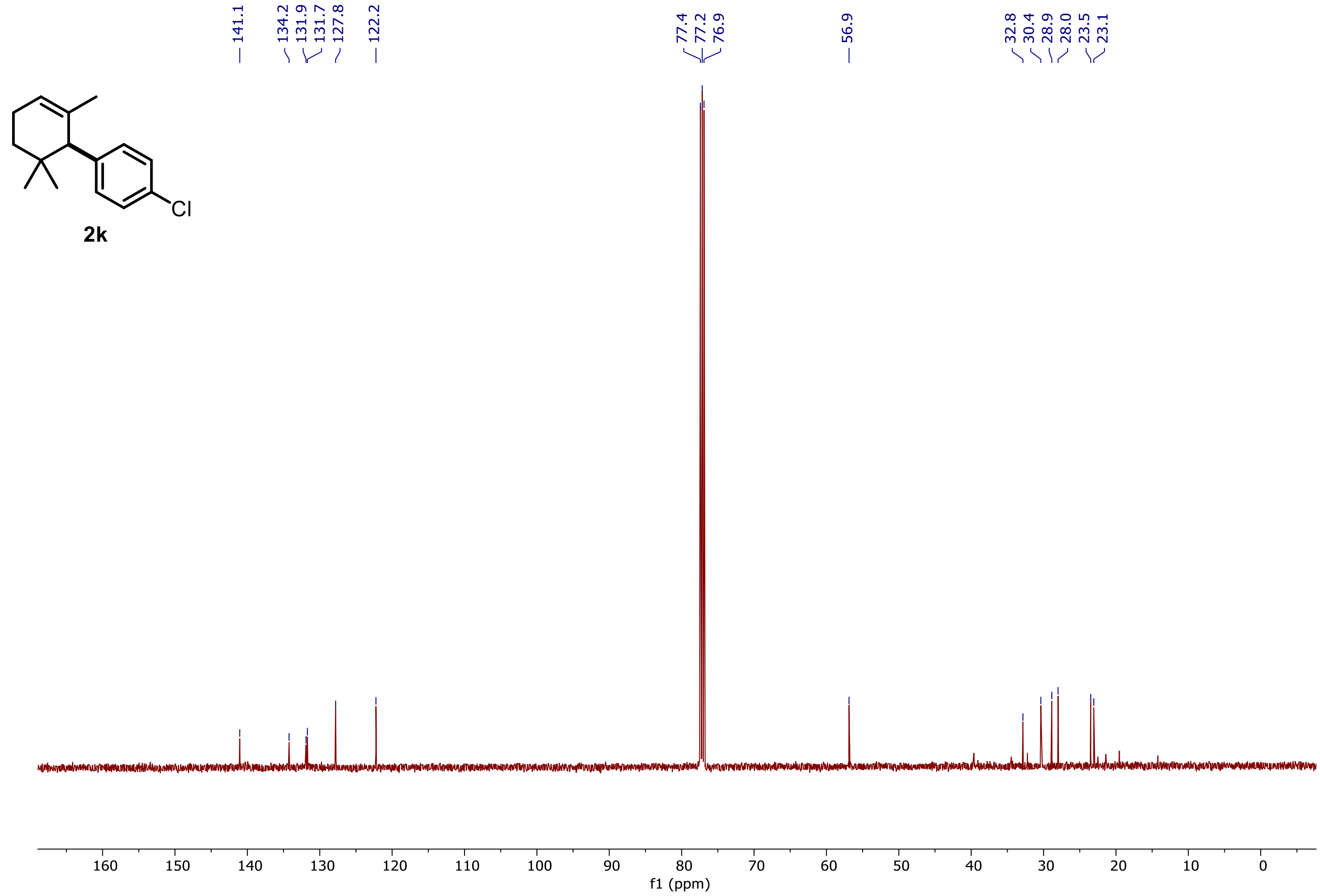

^{1}H NMR spectrum of **2l**

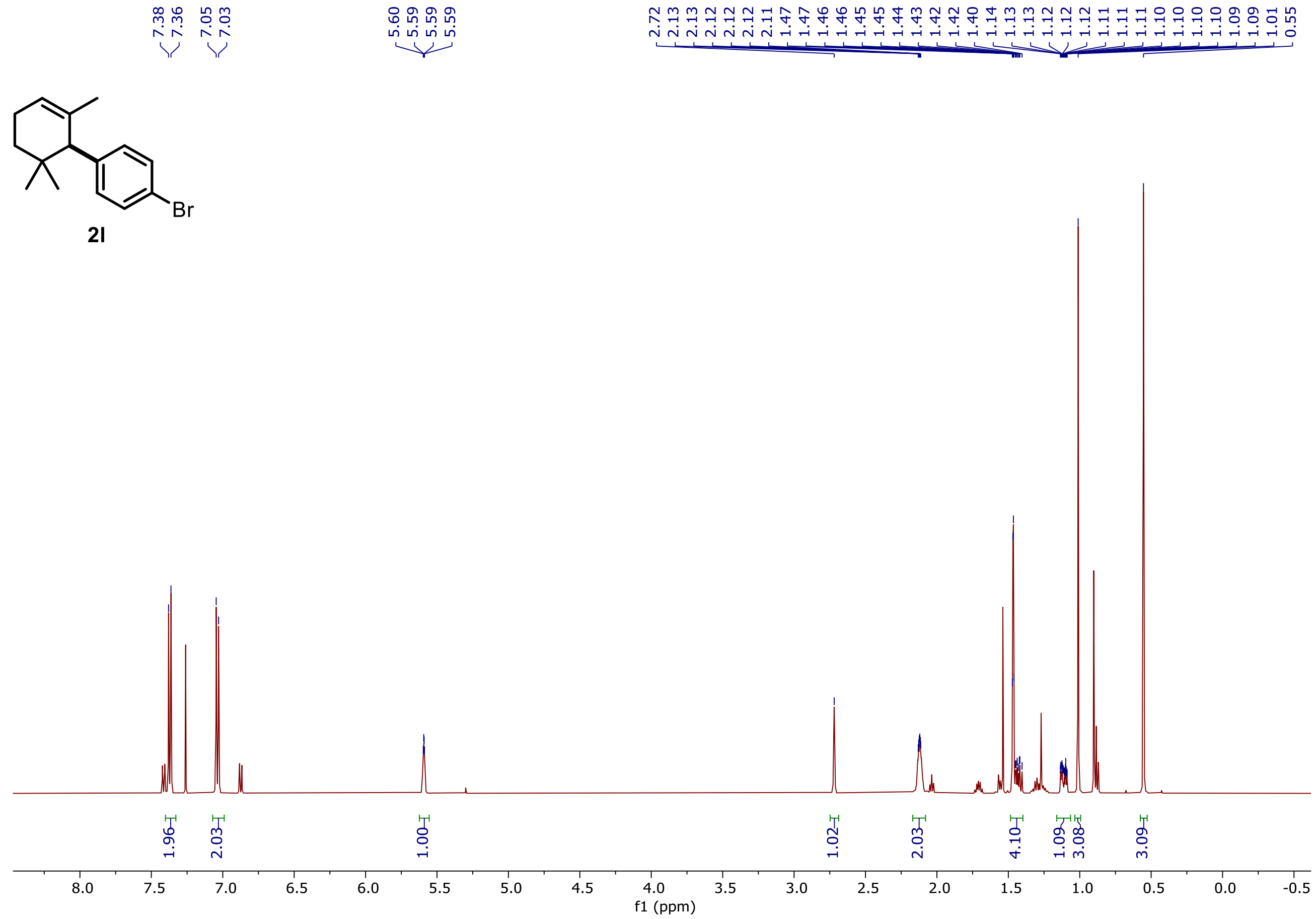

^{13}C NMR spectrum of **2l**

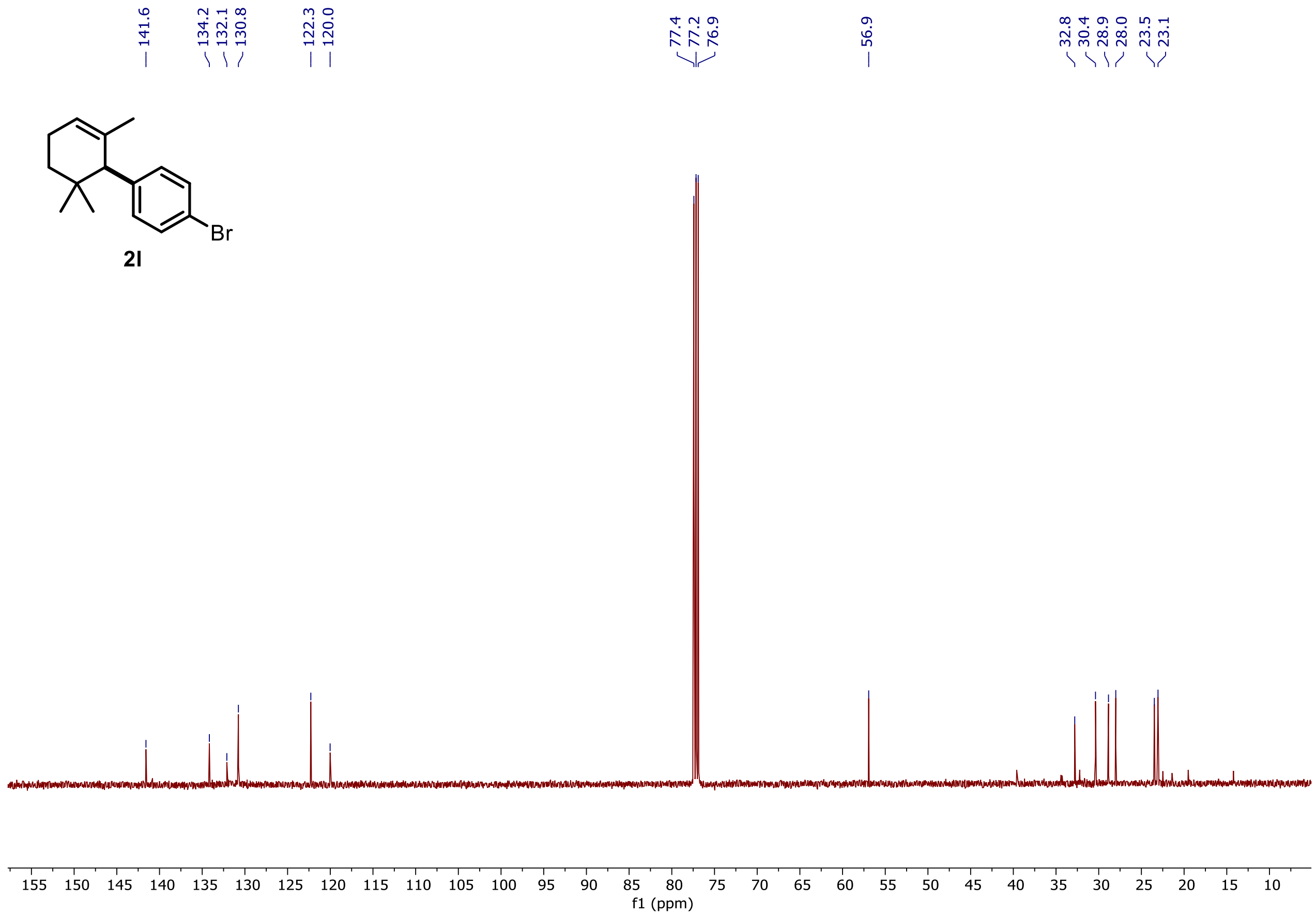

^{1}H NMR spectrum of **2m**

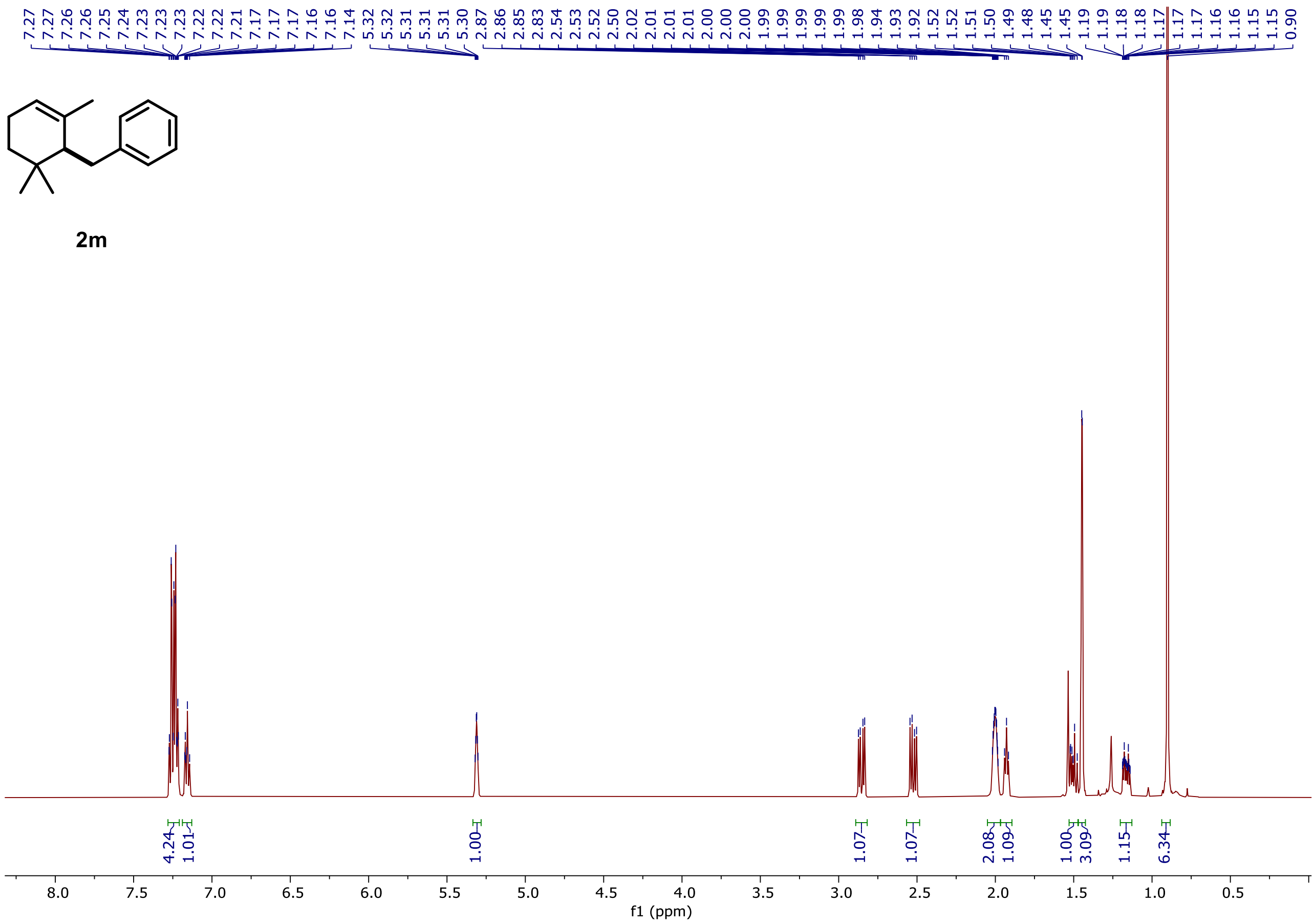

^{13}C NMR spectrum of **2m**

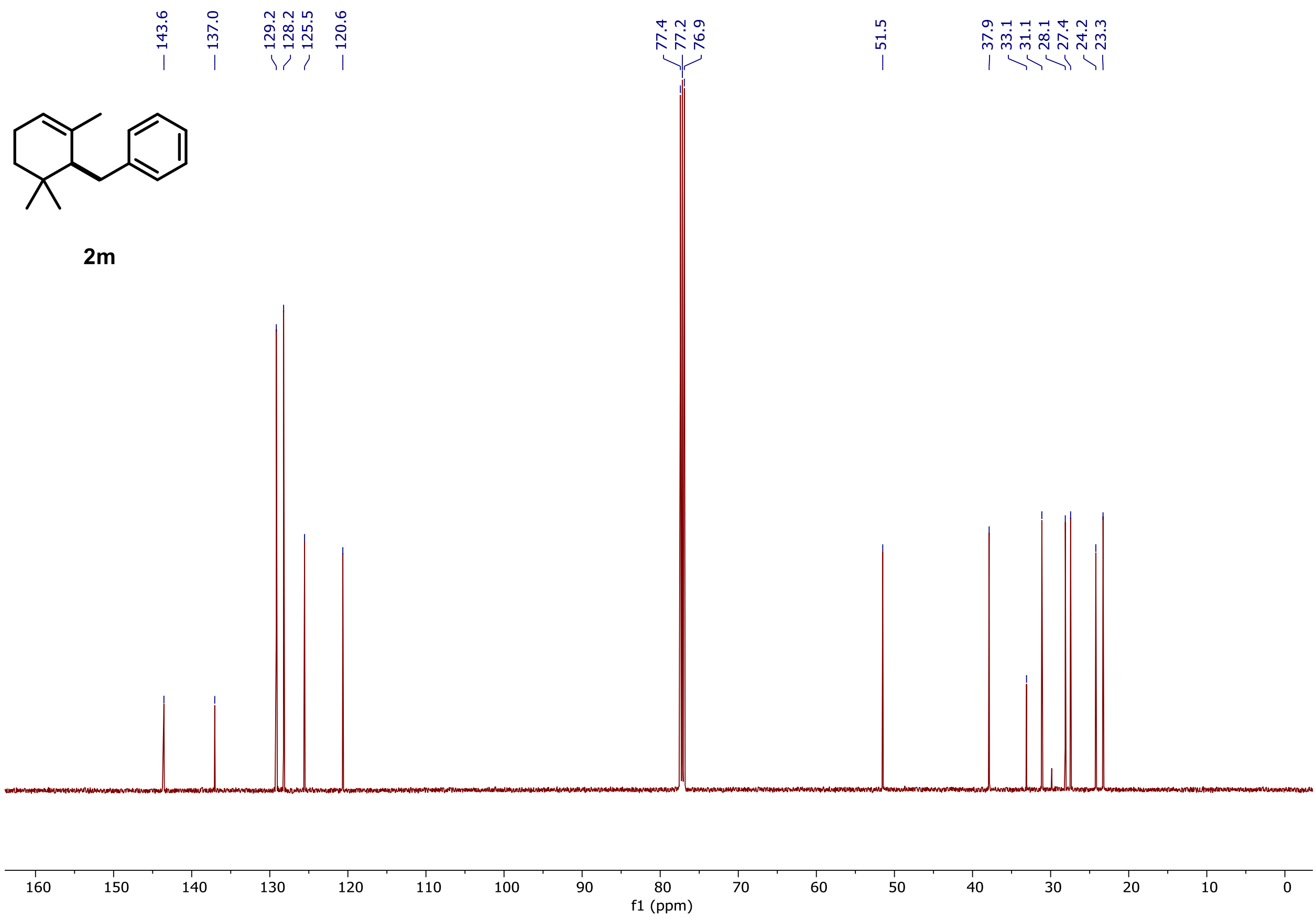

^{1}H NMR spectrum of **2n**

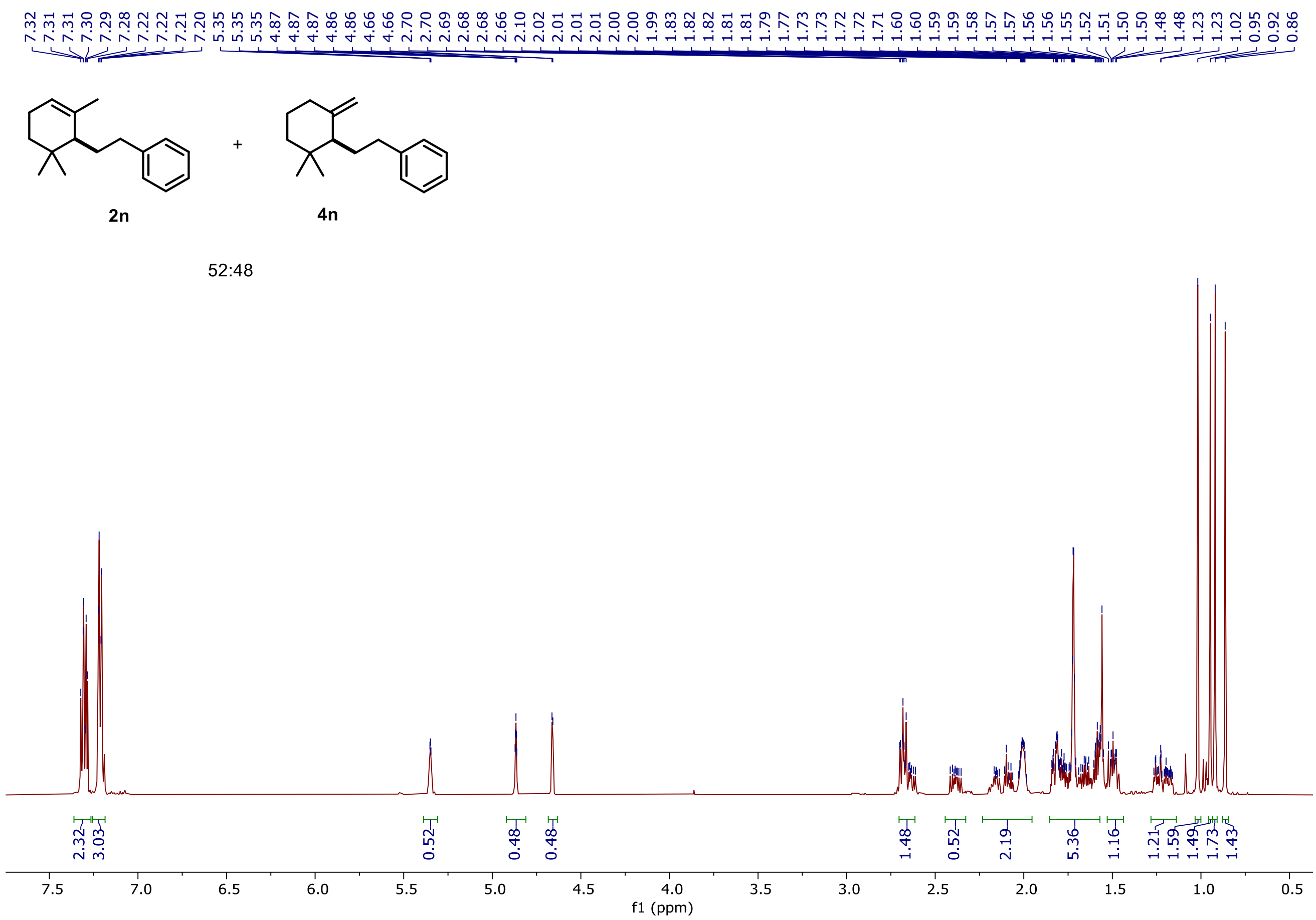

^{13}C NMR spectrum of **2n**

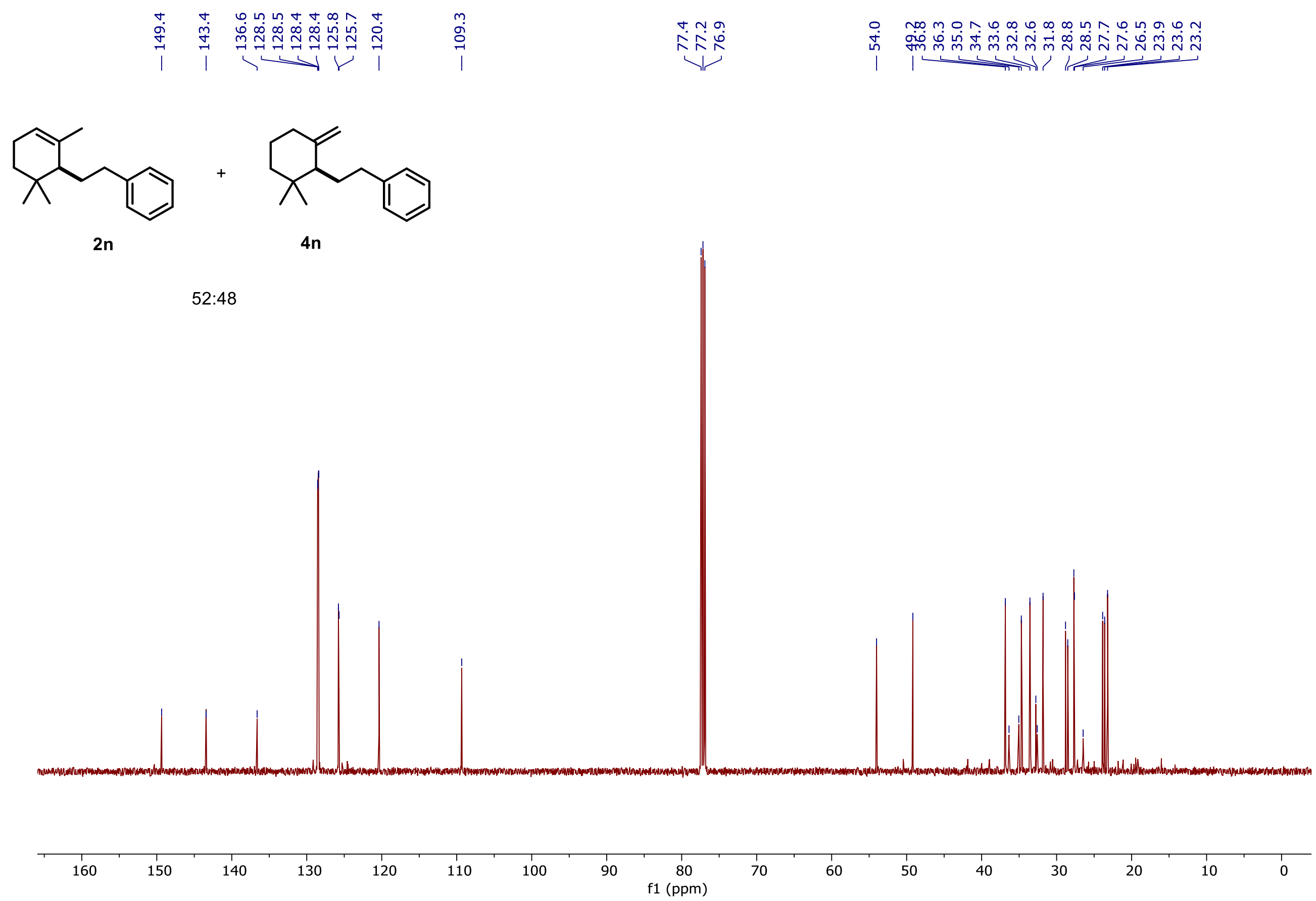

^{1}H NMR spectrum of **2o**

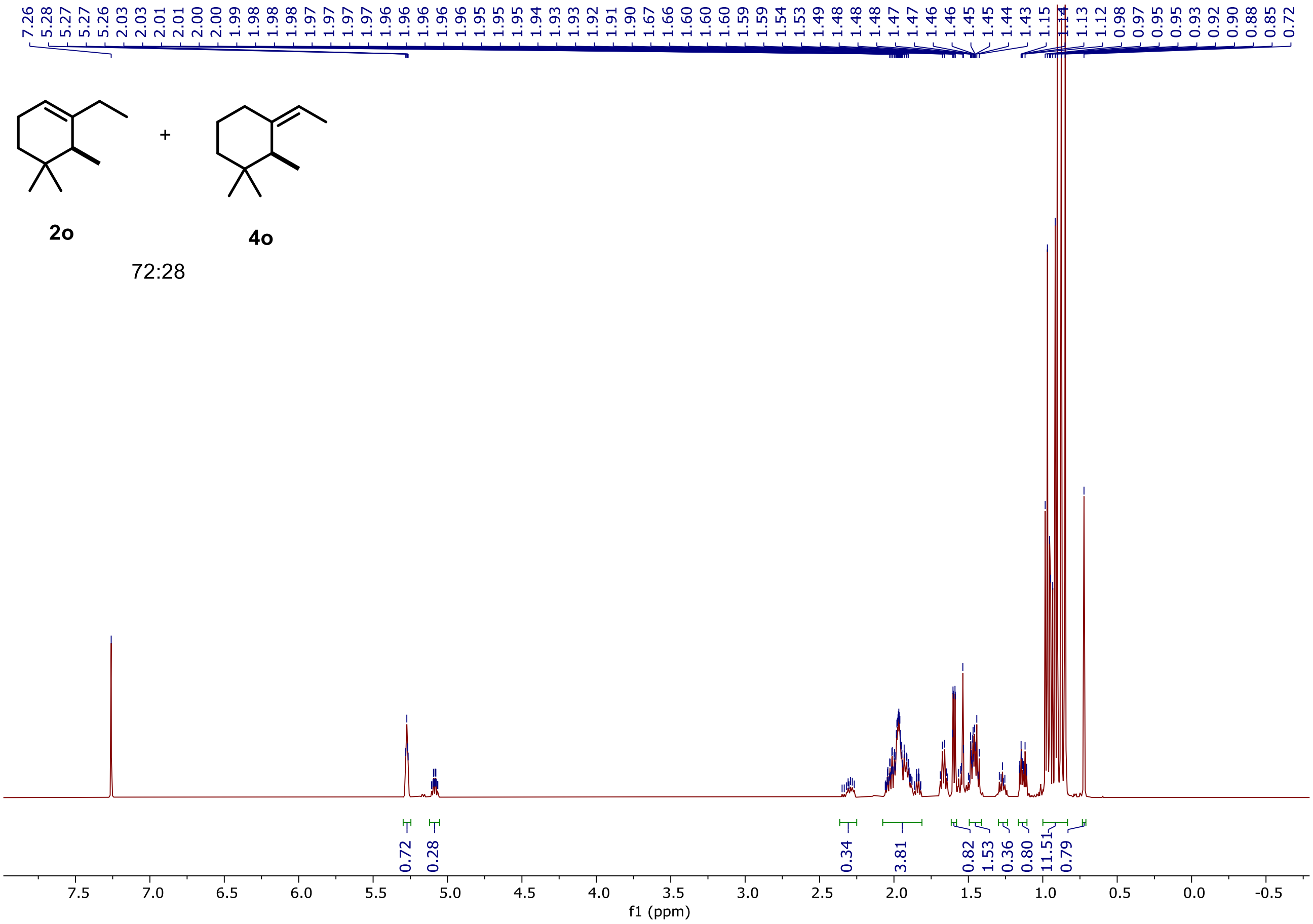

^{13}C NMR spectrum of **2o**

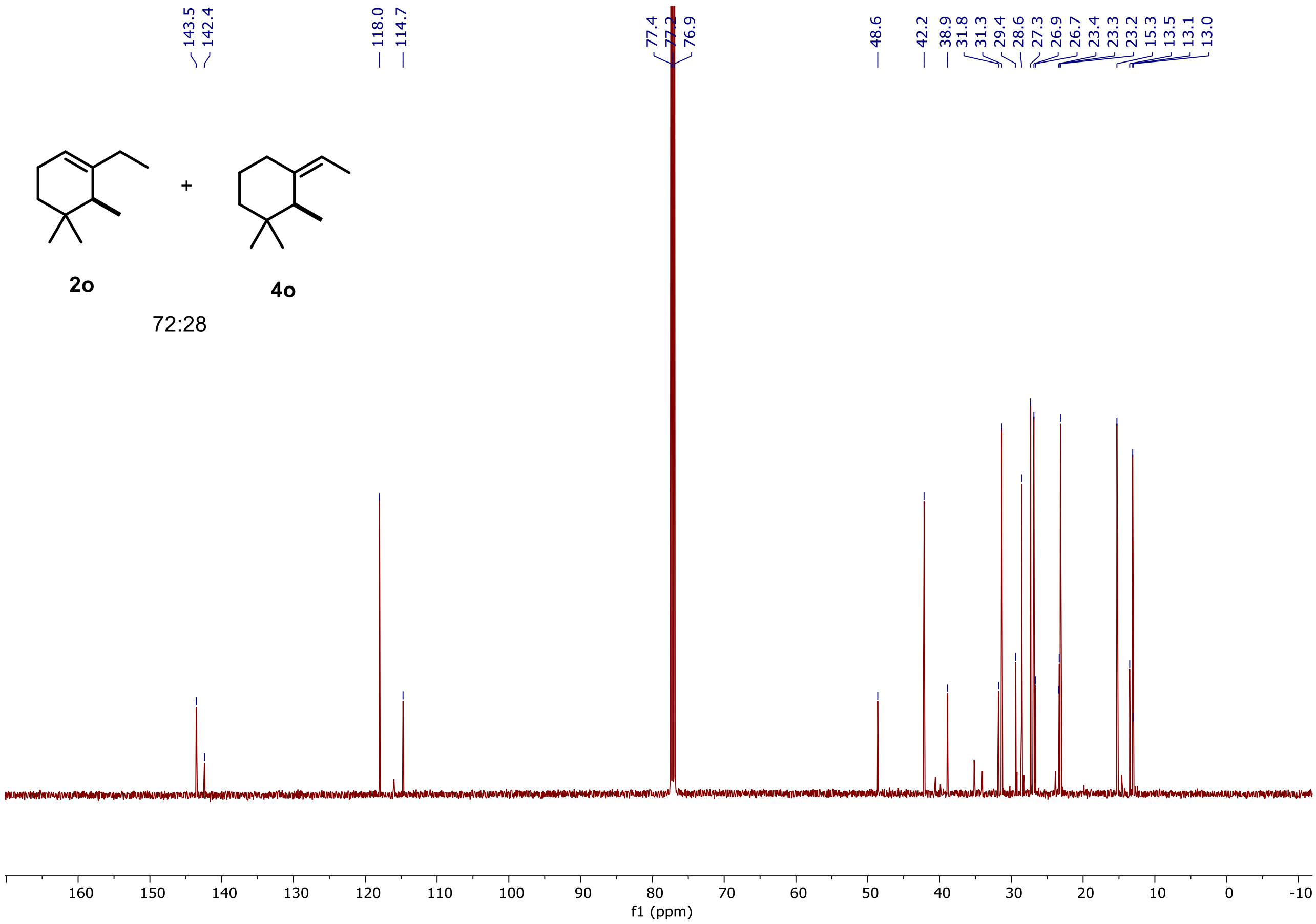

^{1}H NMR spectrum of **2p**

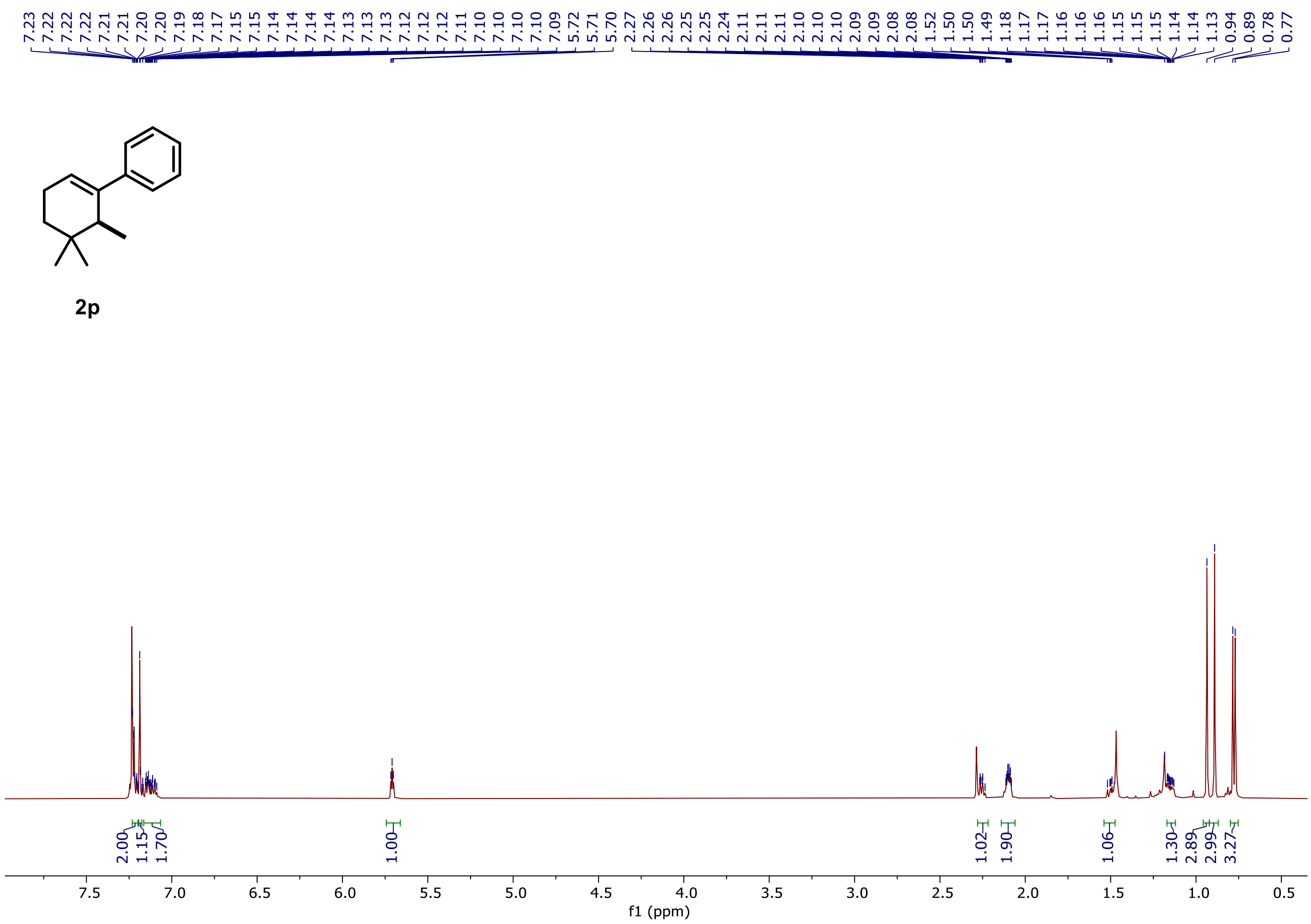

^{13}C NMR spectrum of **2p**

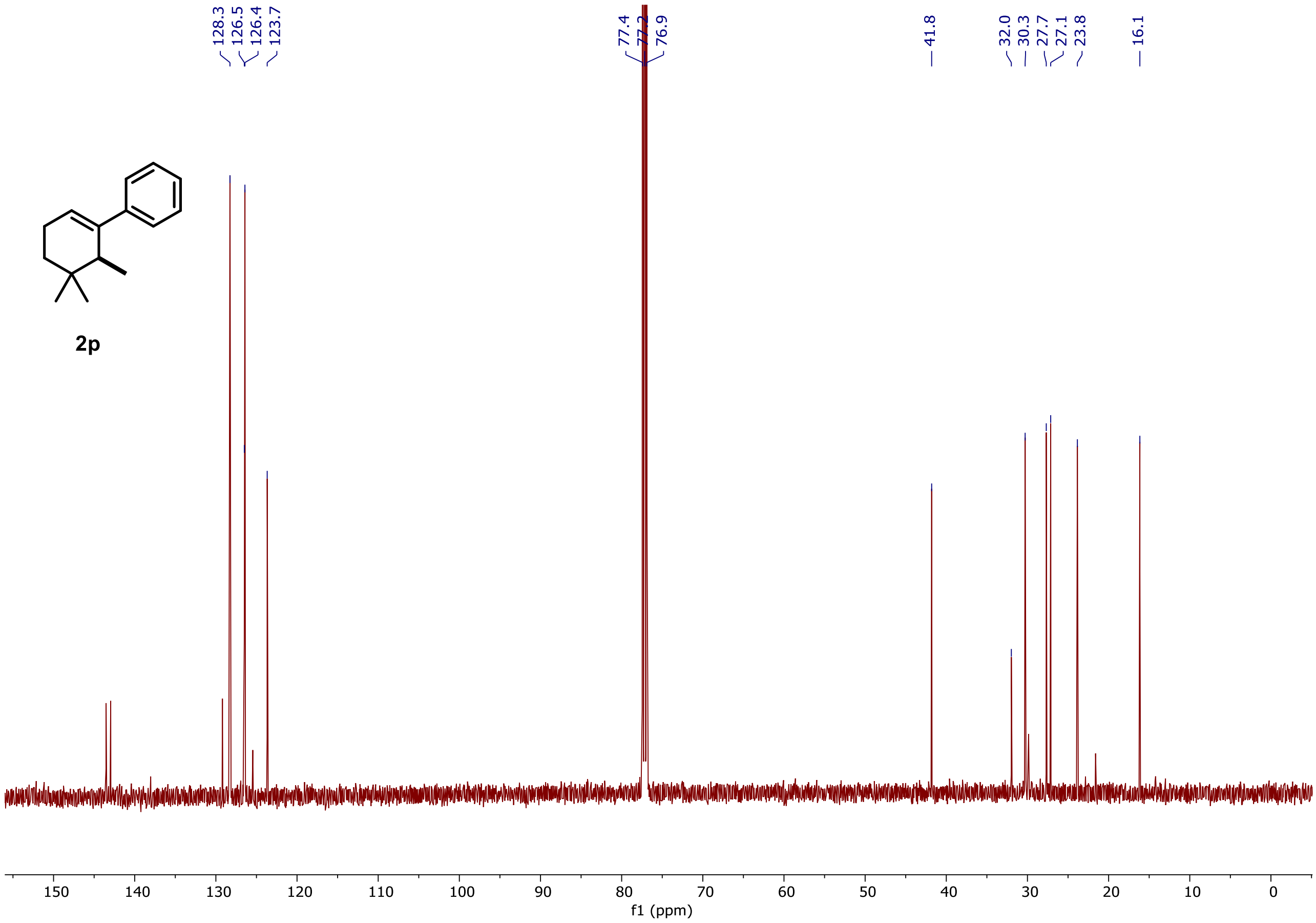

^{1}H NMR spectrum of **2r**

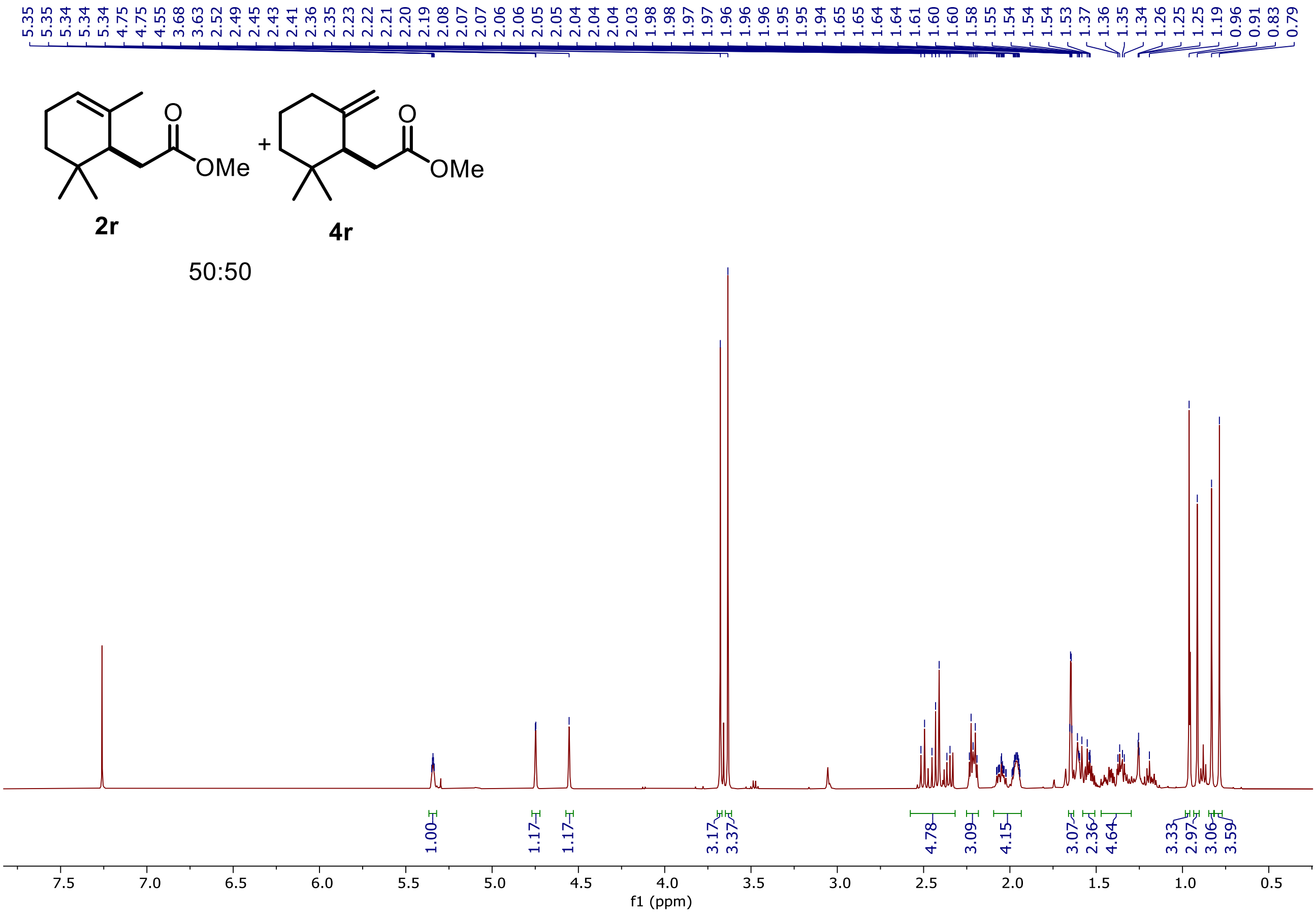

^{13}C NMR spectrum of 2r

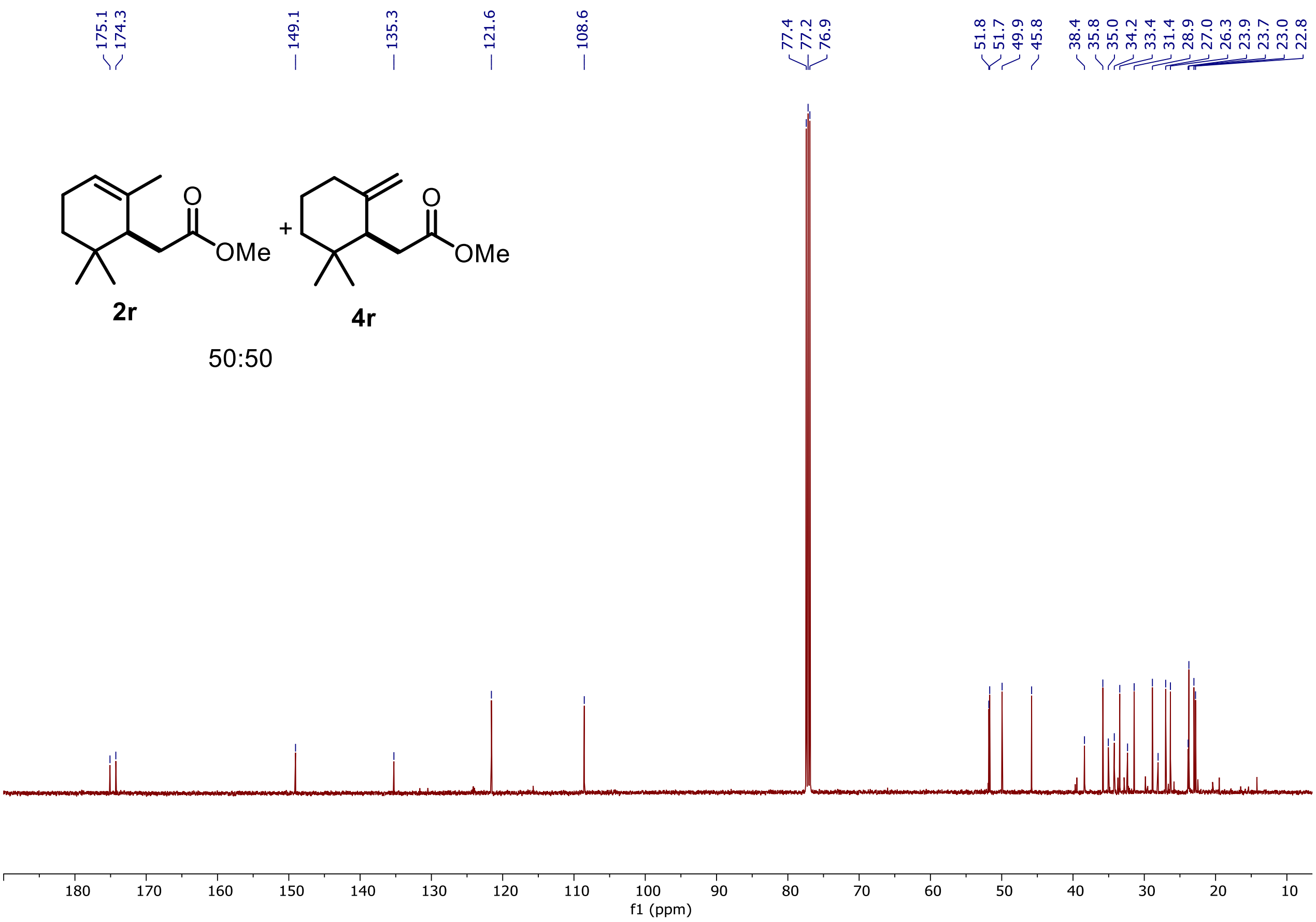

^{1}H NMR spectrum of **2s**

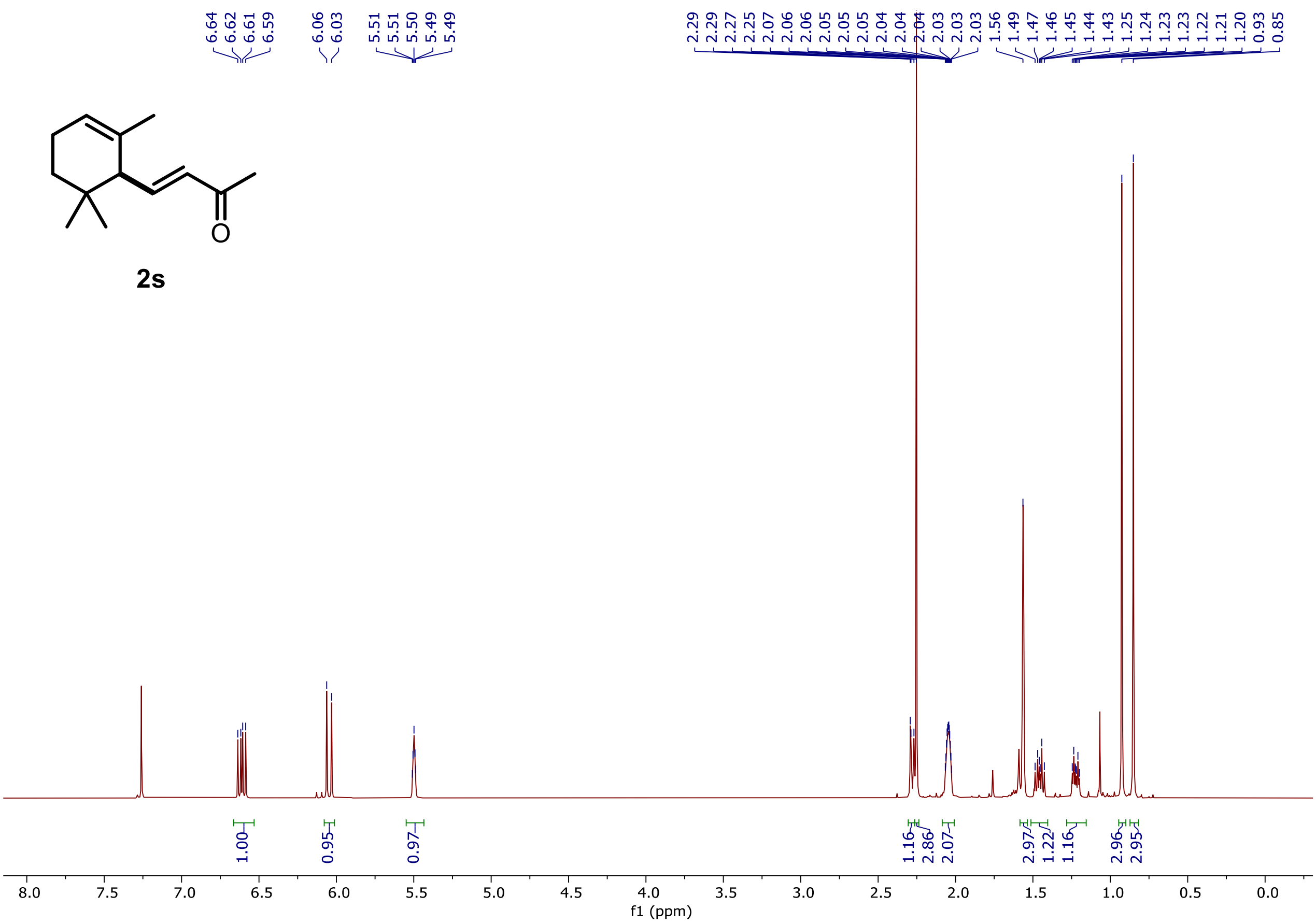

^{13}C NMR spectrum of **2s**

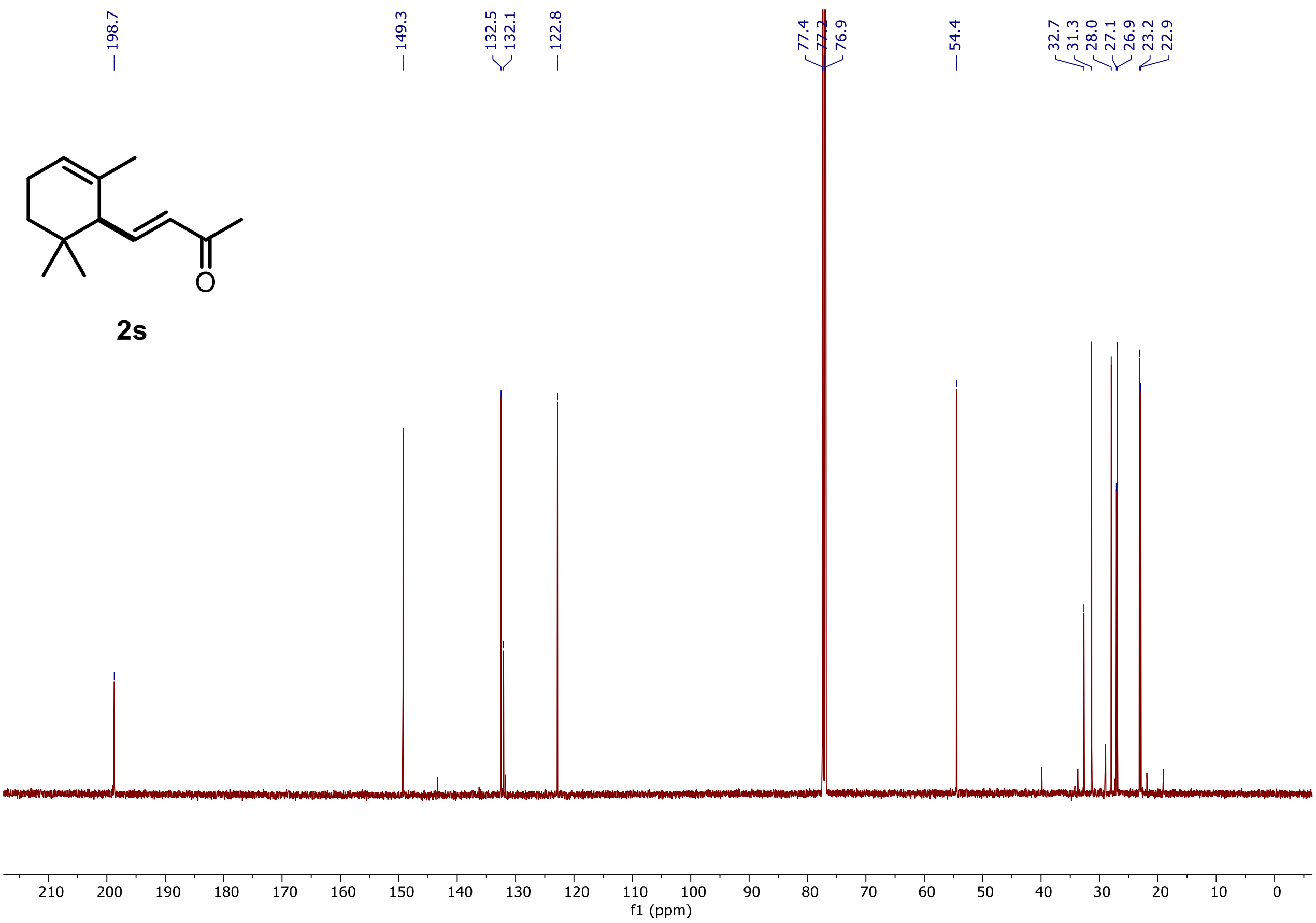

^{1}H NMR spectrum of **(±)-4a**

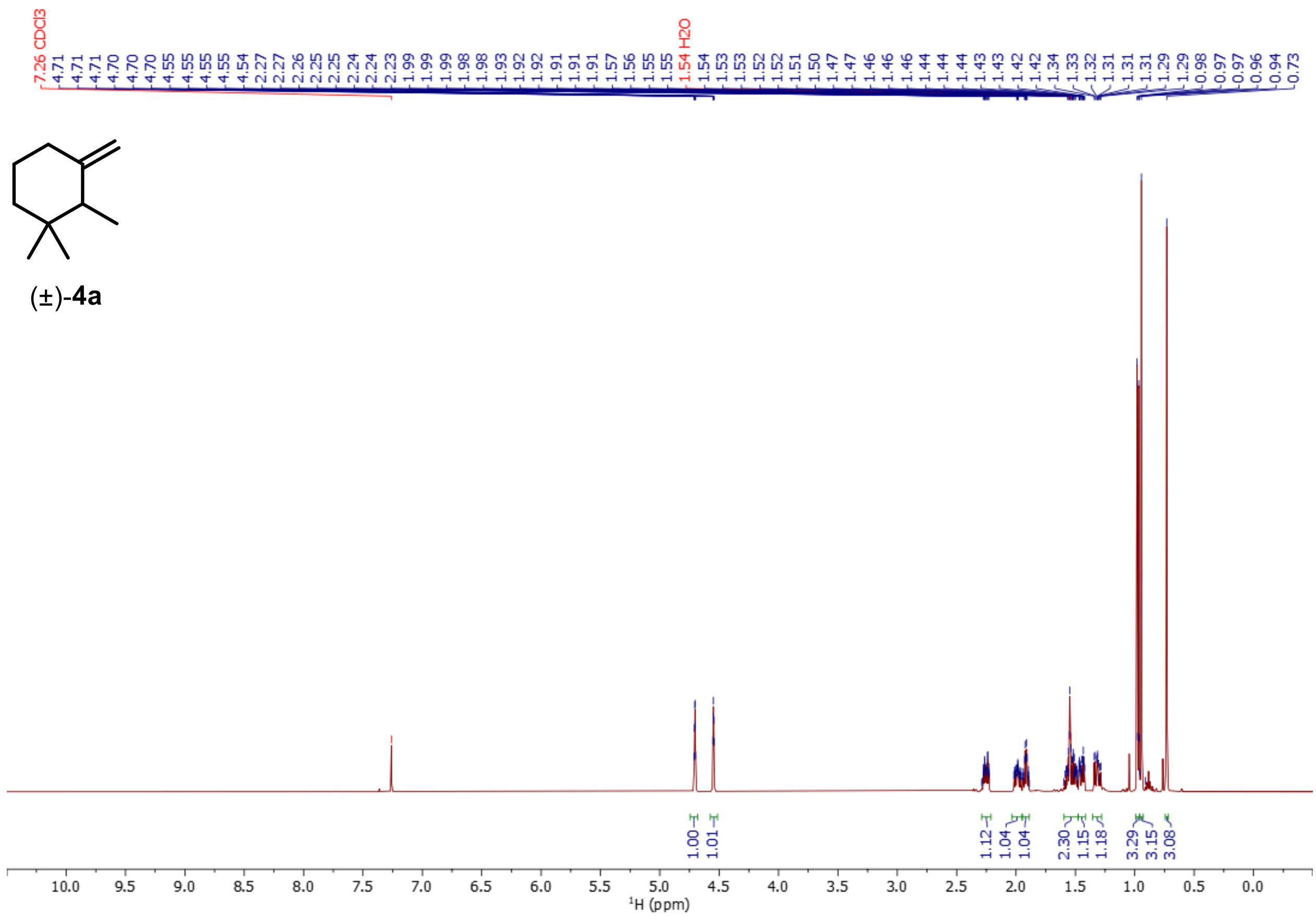

^{13}C NMR spectrum of **(±)-4a**

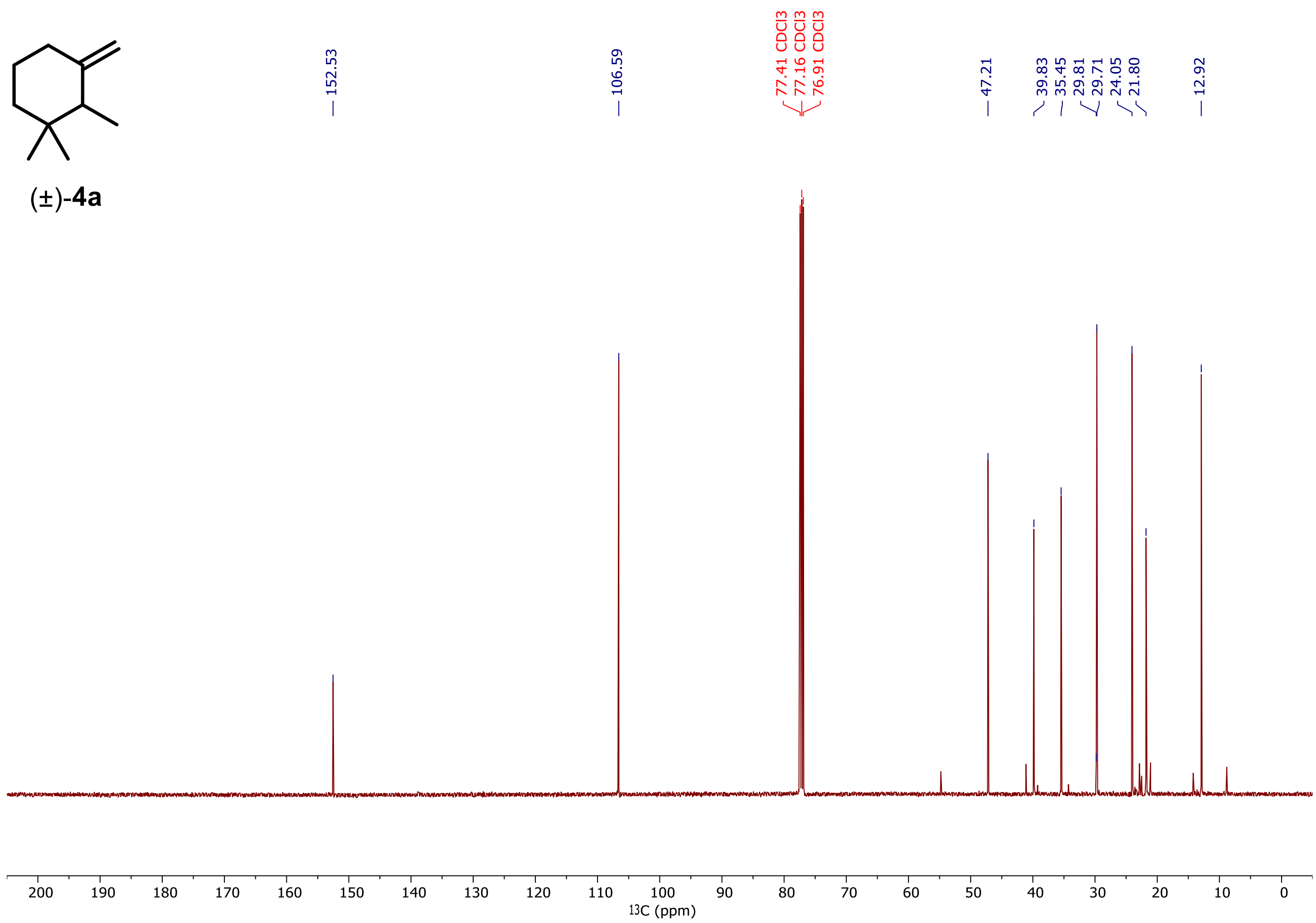

^{1}H NMR spectrum of **IDPi 5d**

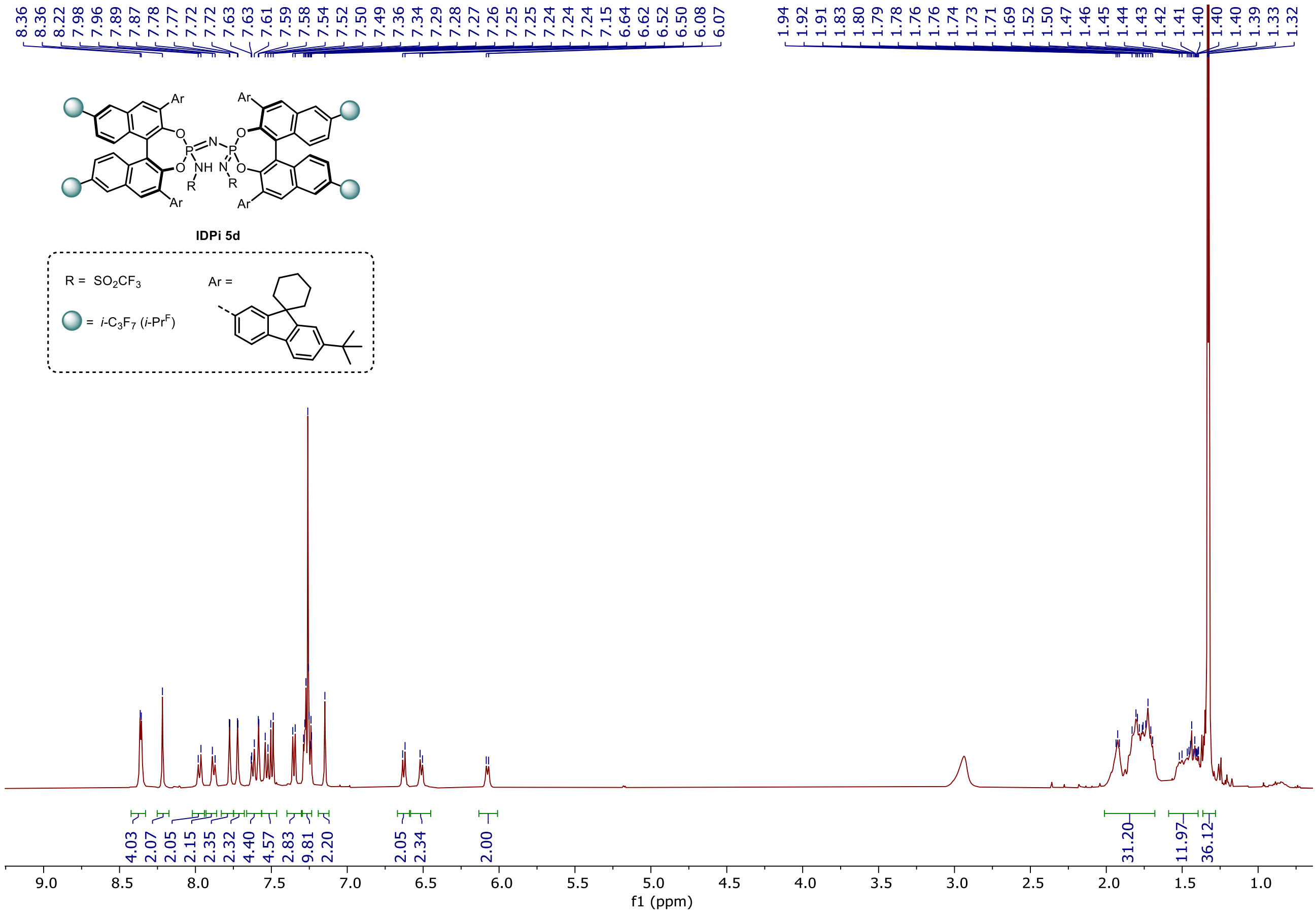

^{13}C NMR spectrum of **IDPi 5d**

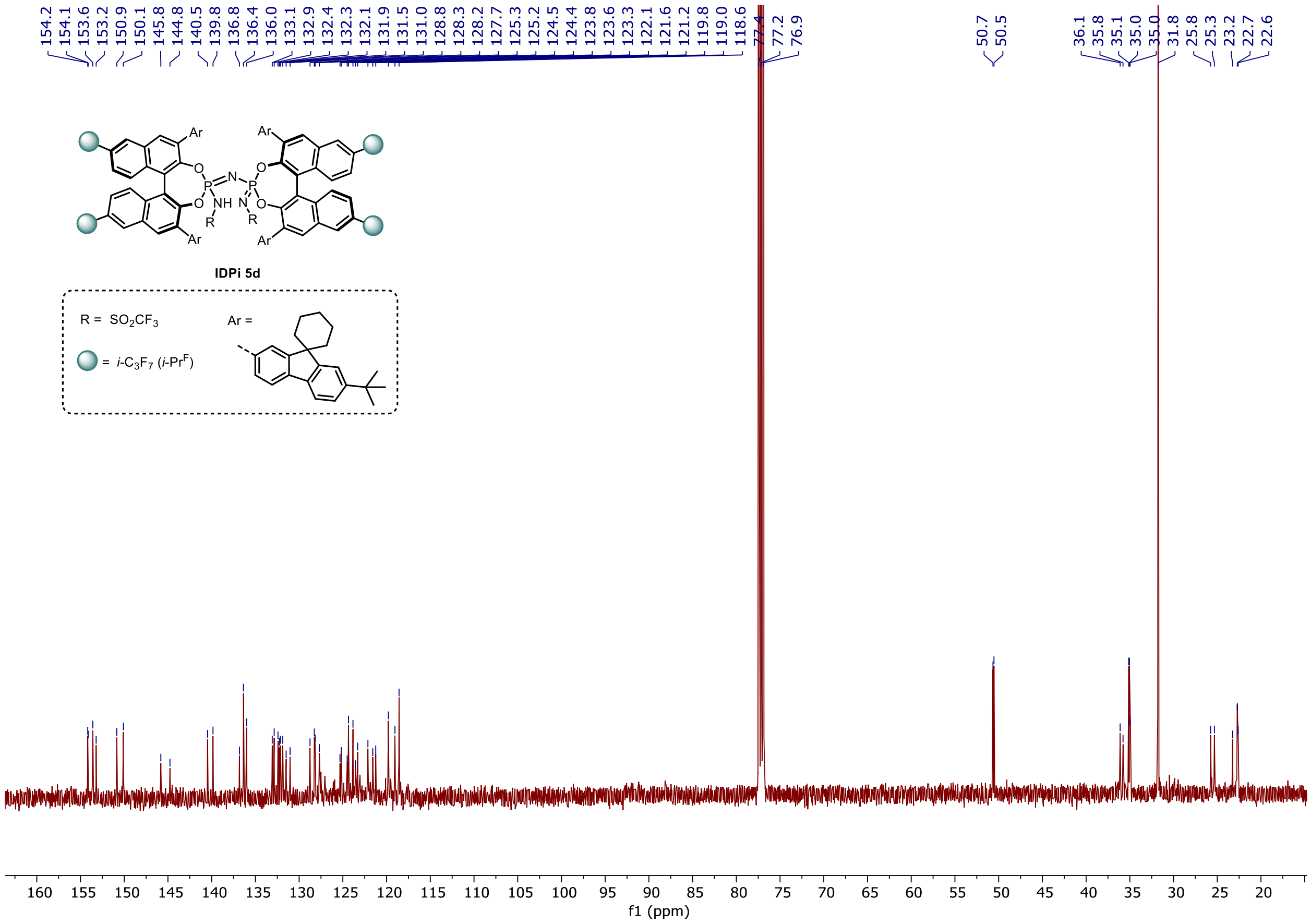

^{19}F NMR spectrum of **IDPi 5d**

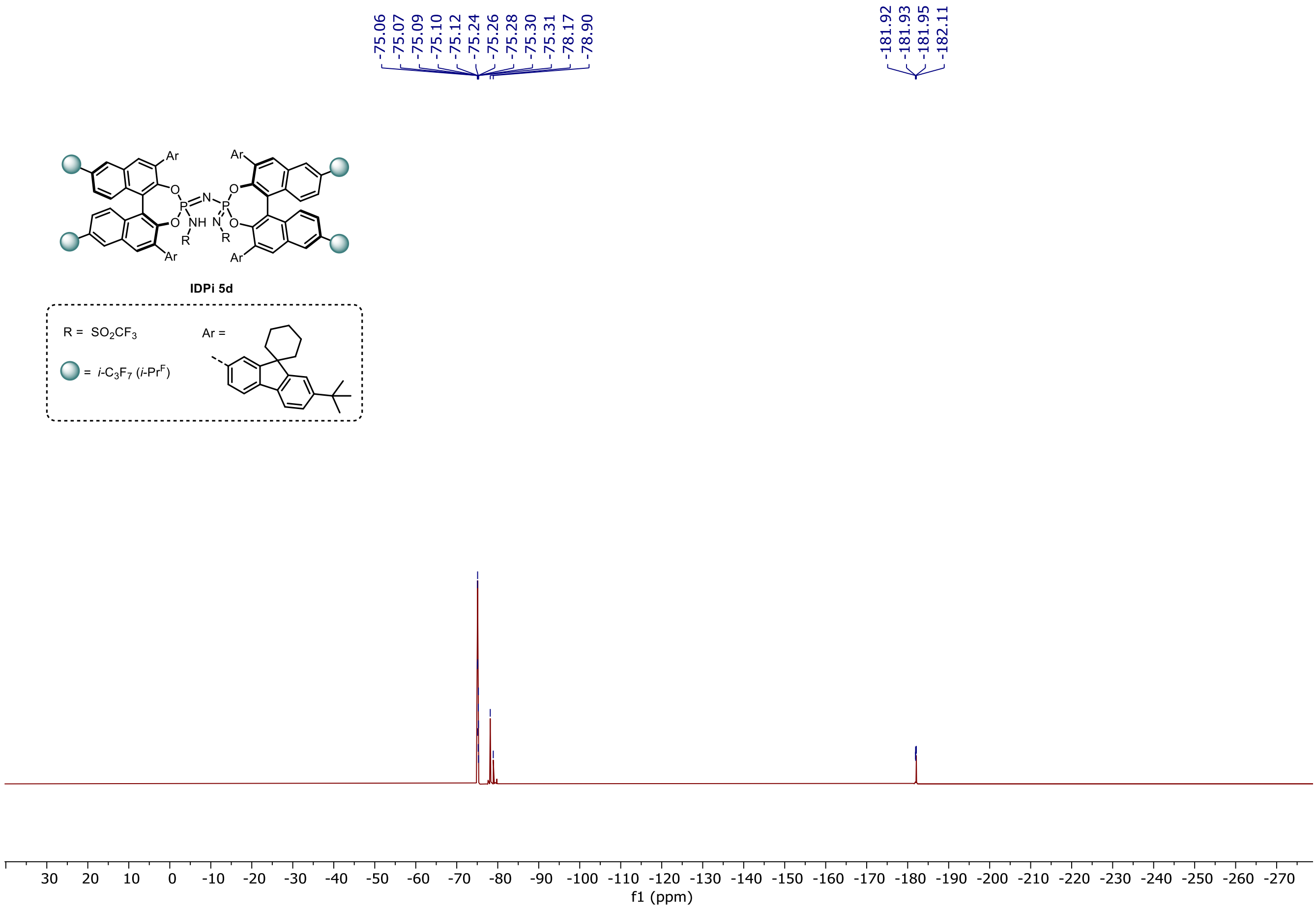

^{31}P NMR spectrum of **IDPi 5d**

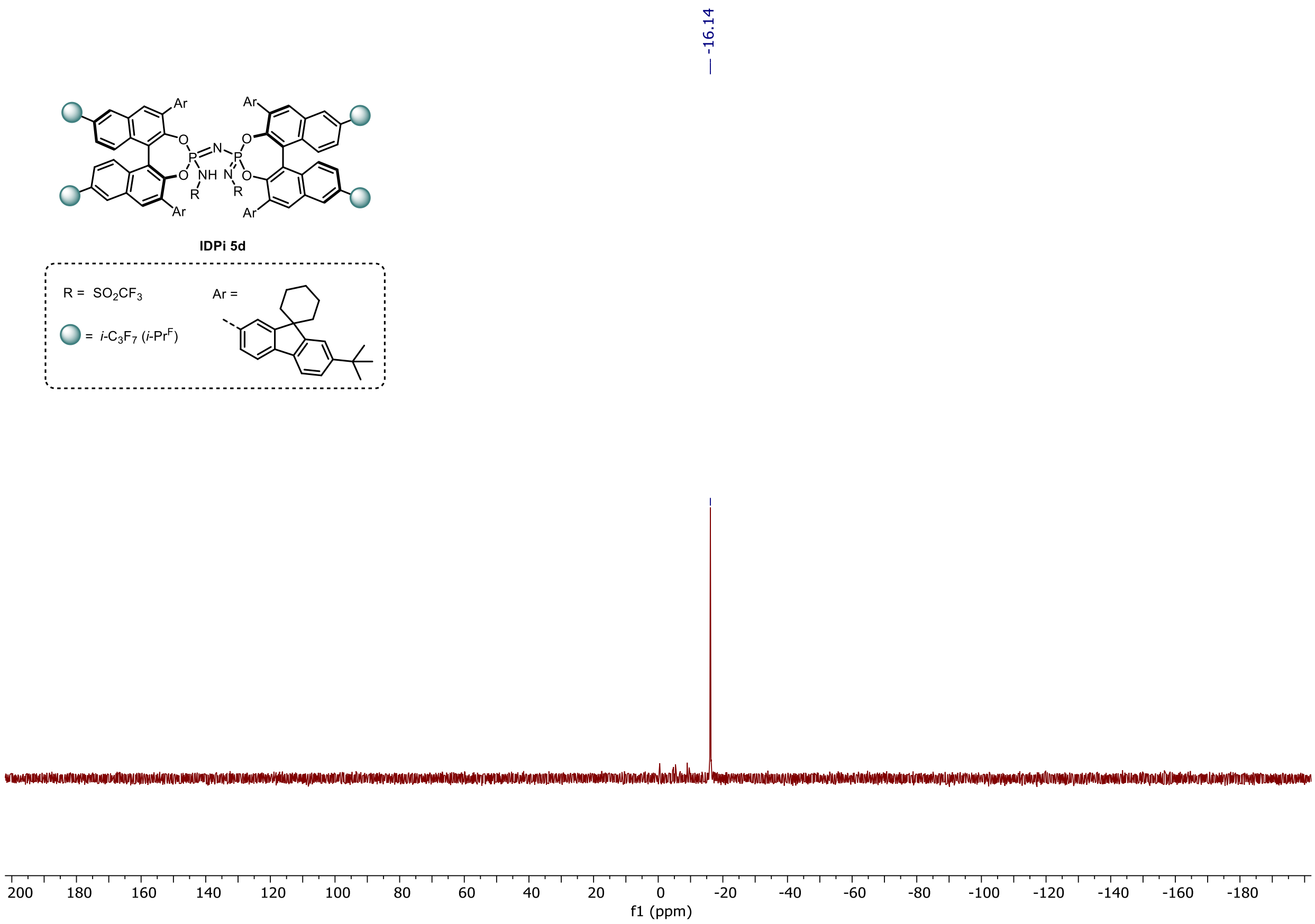

^{1}H NMR spectrum of **6a**

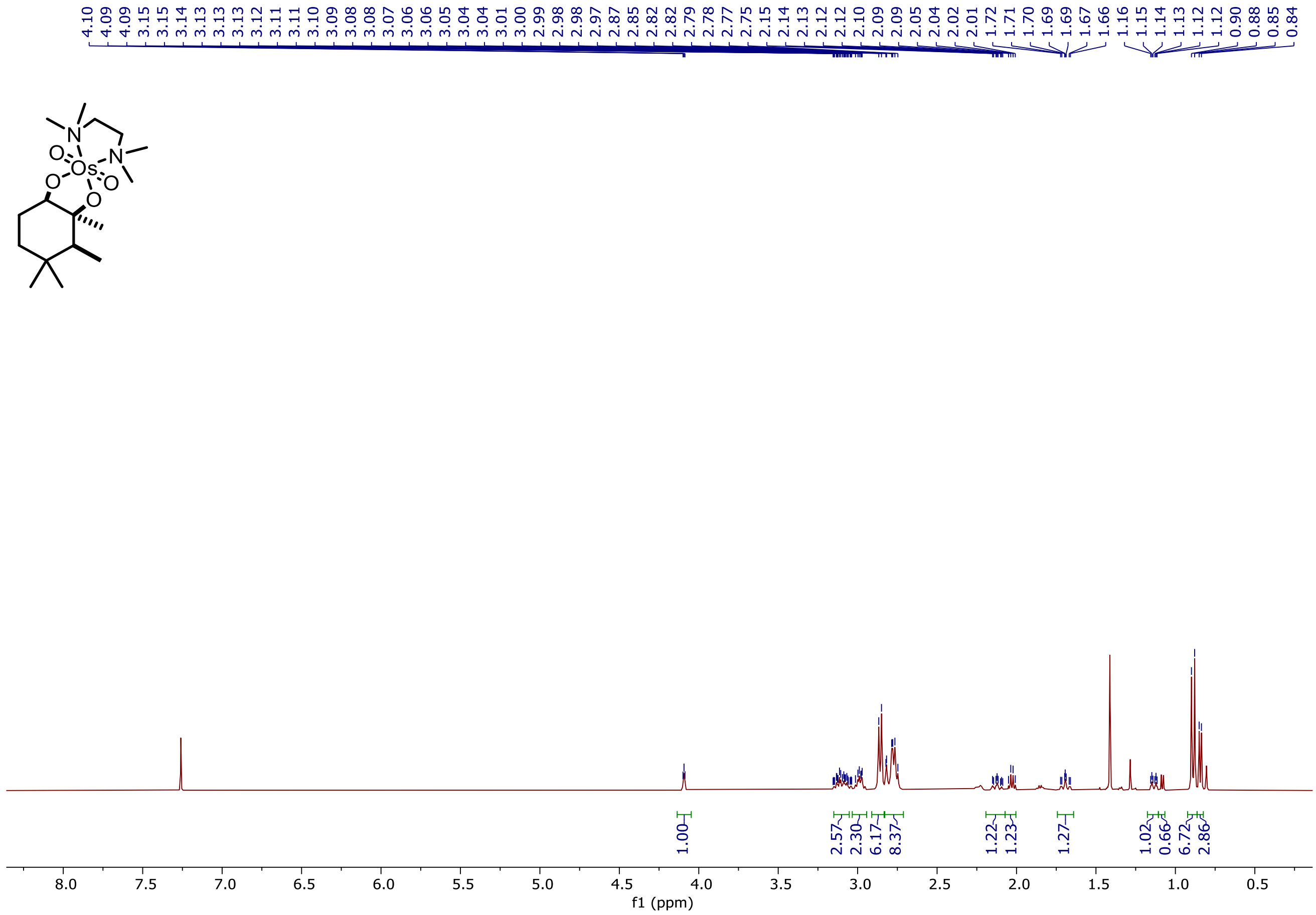

^{13}C NMR spectrum of **6a**

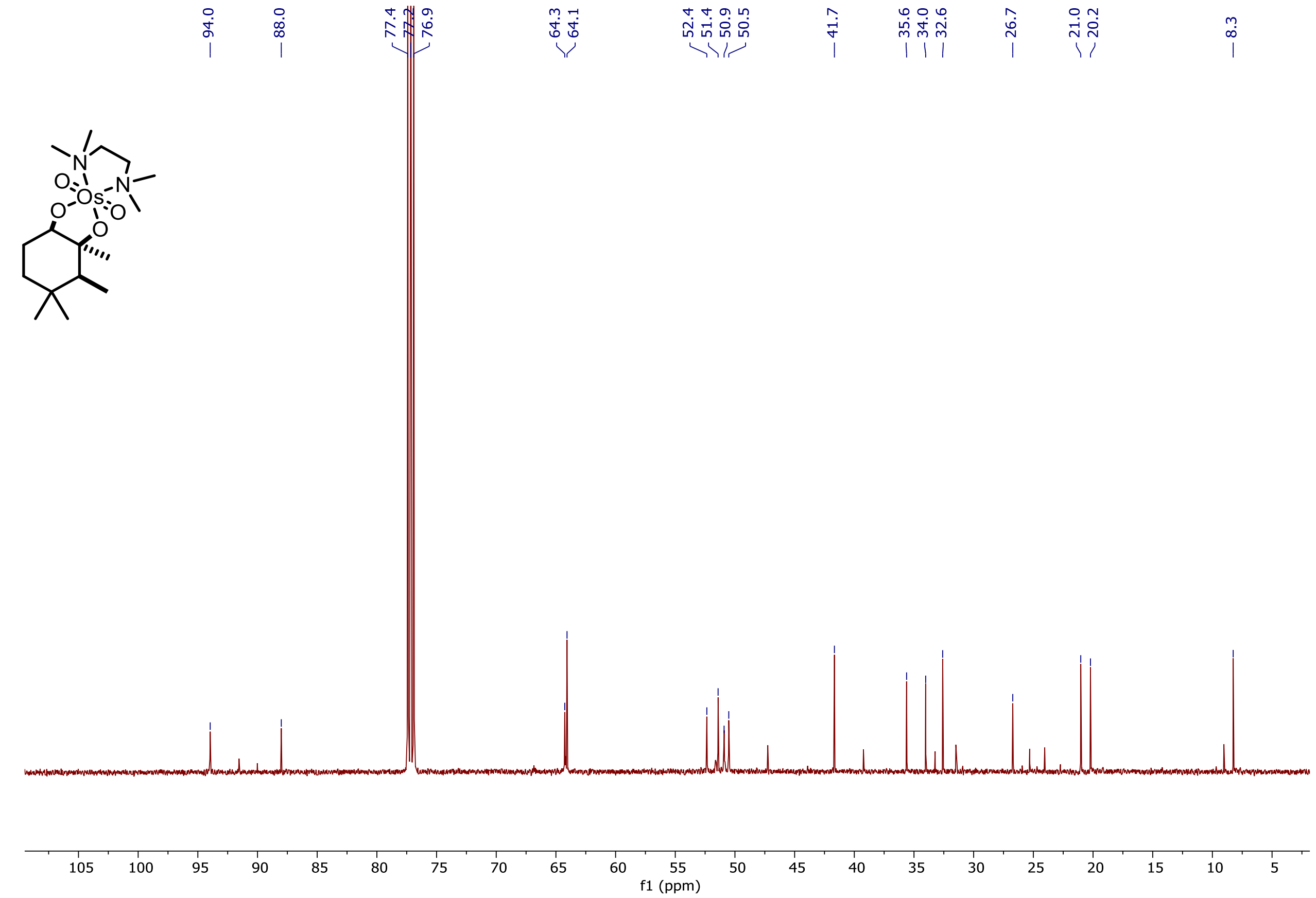

^{1}H NMR spectrum of osmate ester **6f**.

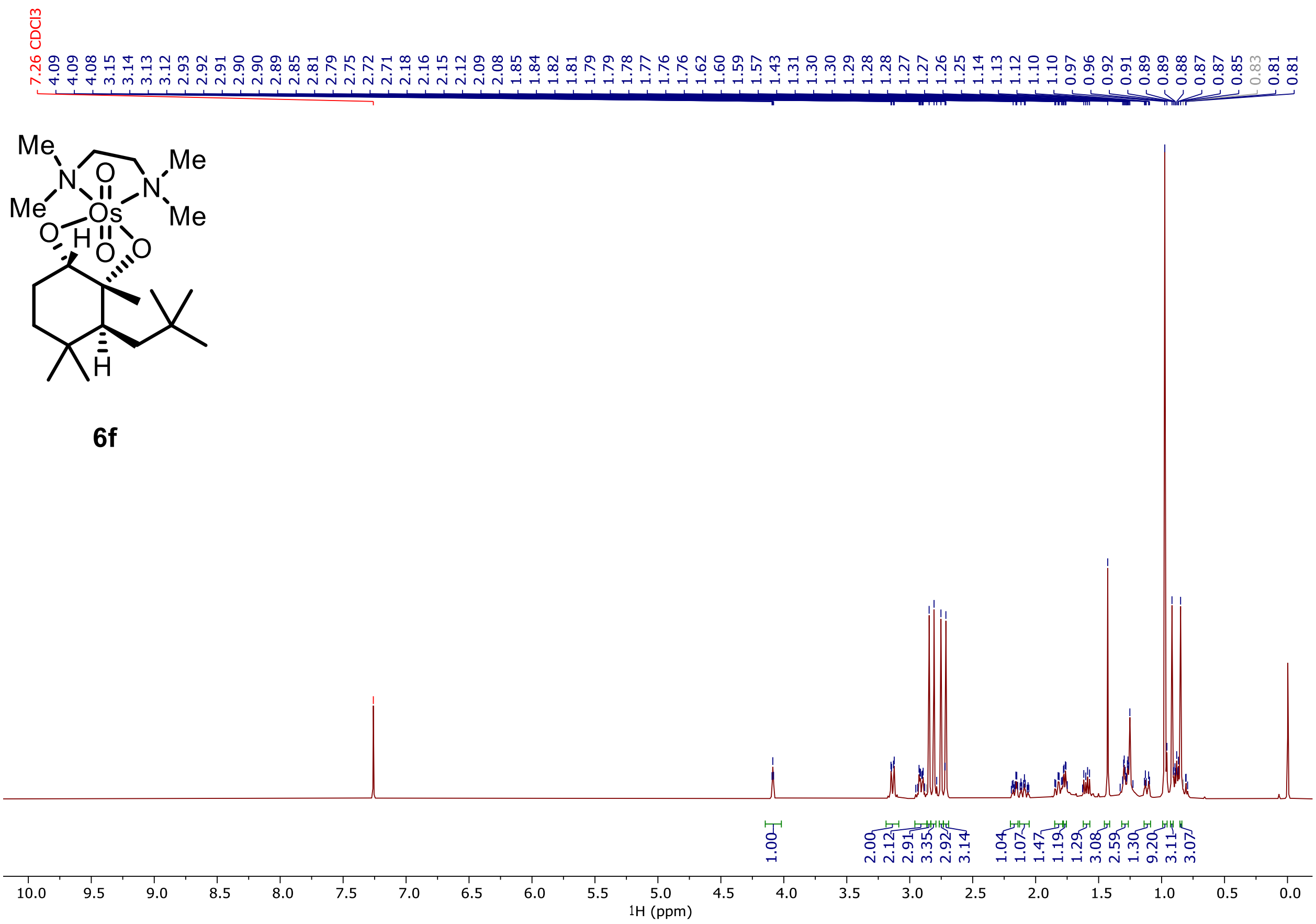

^{13}C NMR spectrum of osmate ester **6f**.

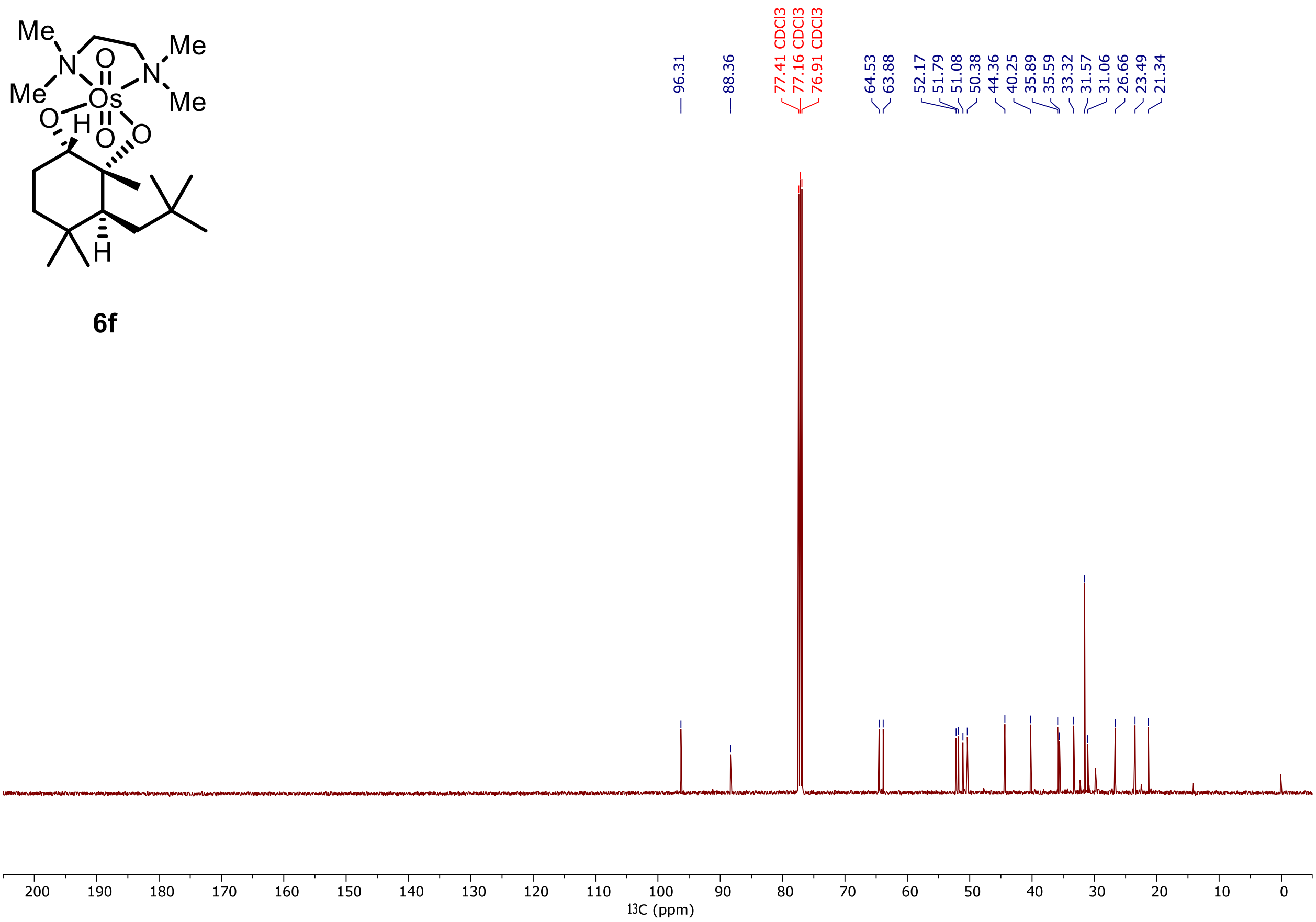

^{1}H NMR spectrum of **7b**

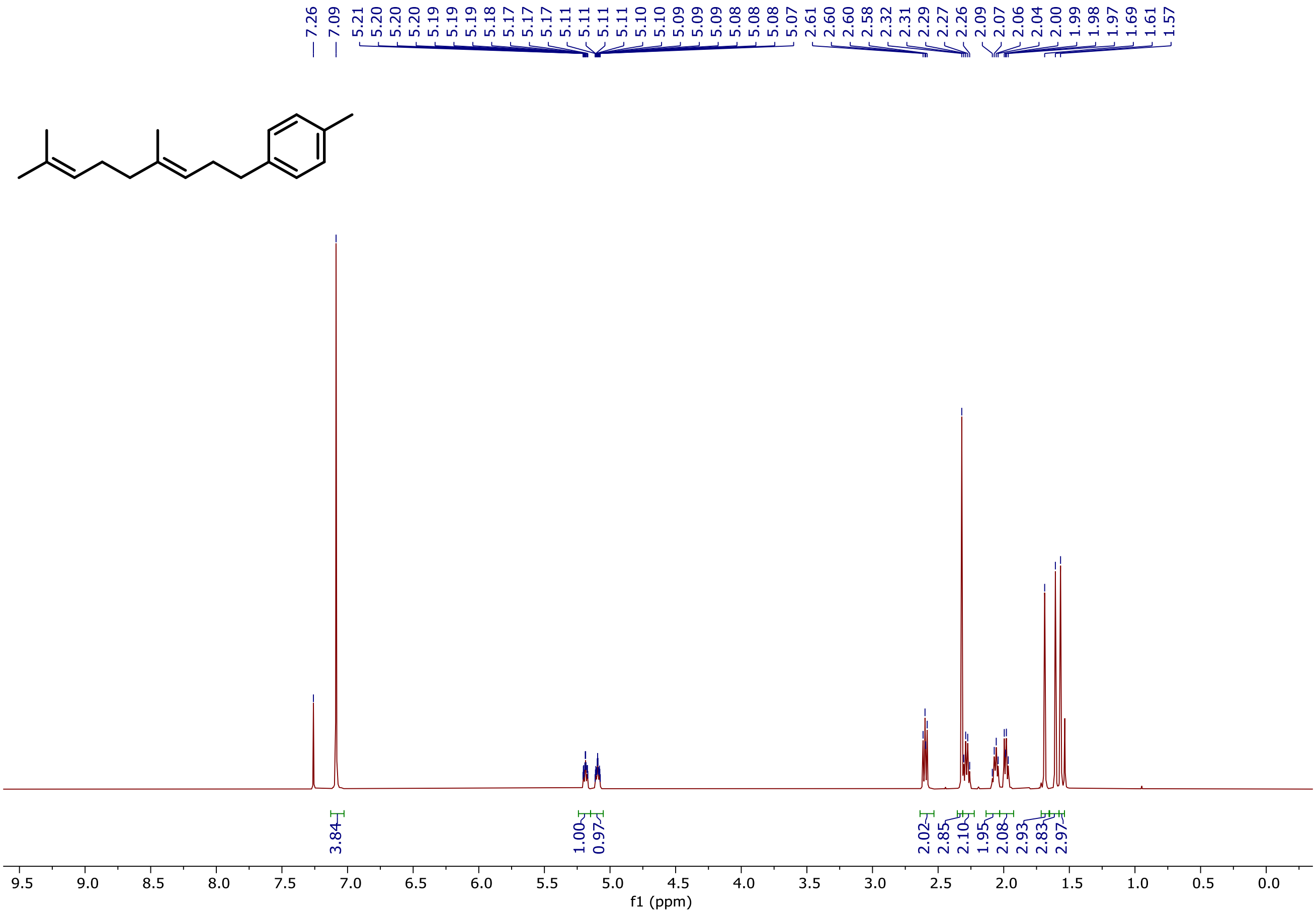

^{13}C NMR spectrum of **7b**

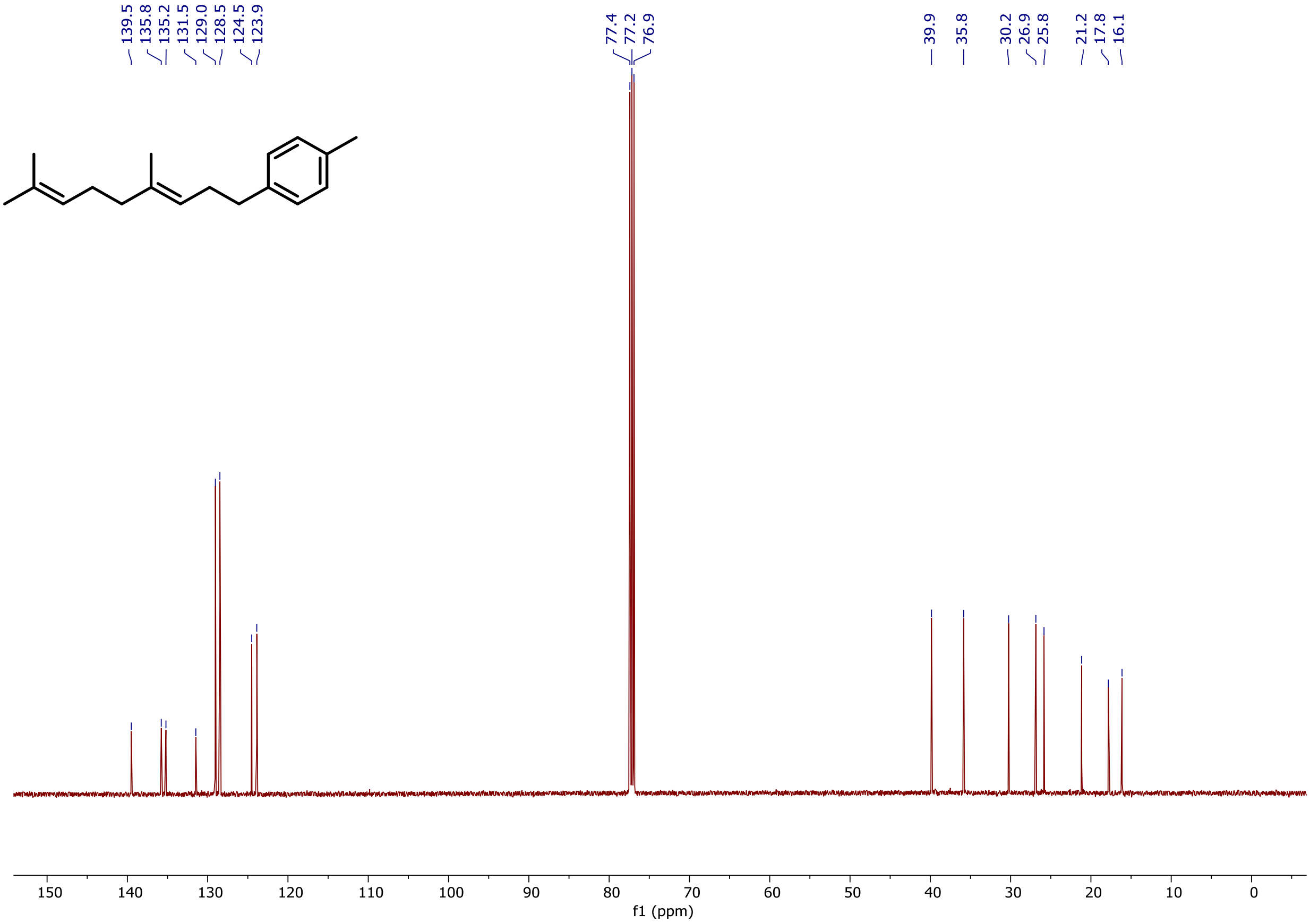

^{1}H NMR spectrum of **7c**

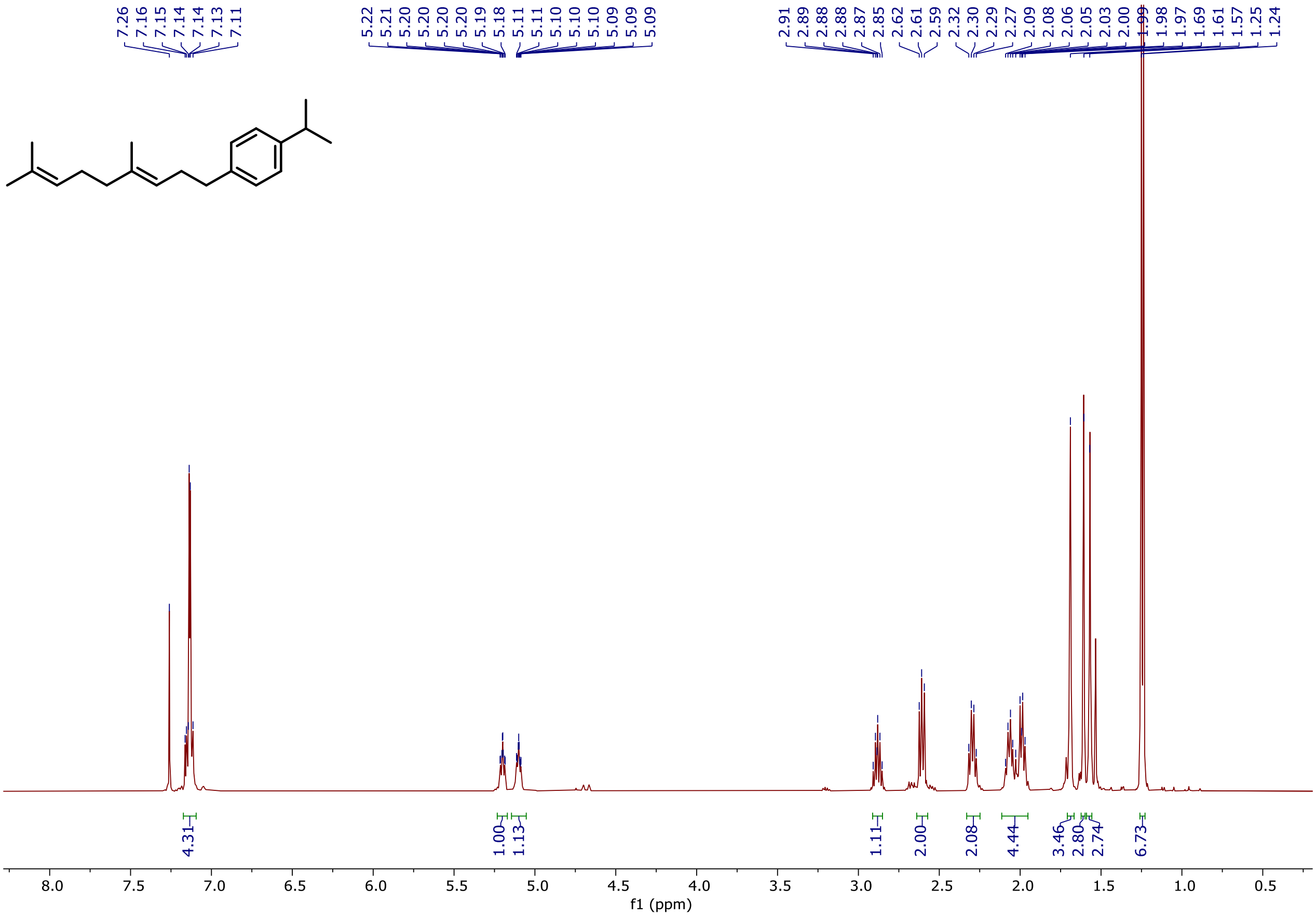

^{13}C NMR spectrum of **7c**

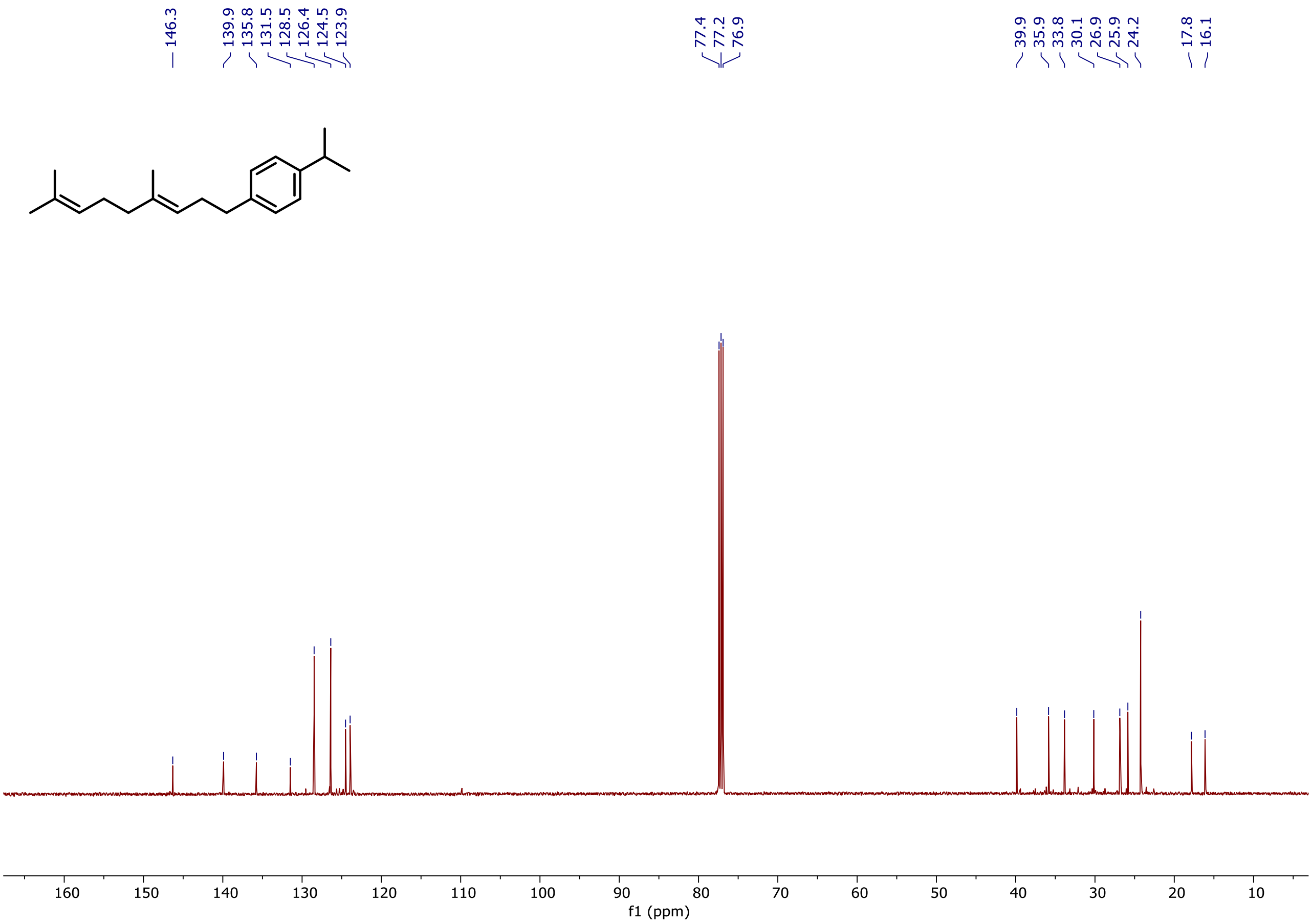

^{1}H NMR spectrum of **7d**

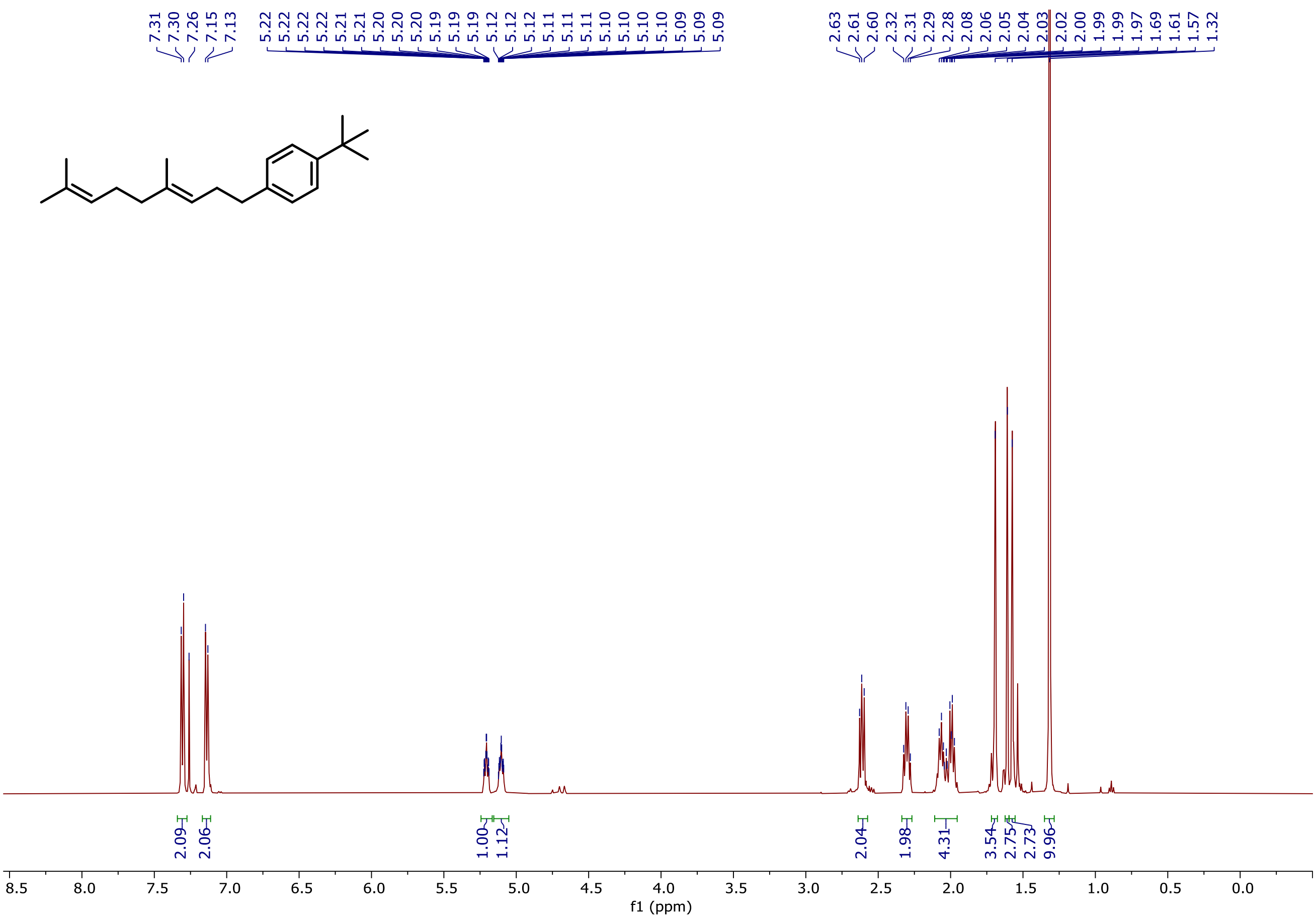

^{13}C NMR spectrum of **7d**

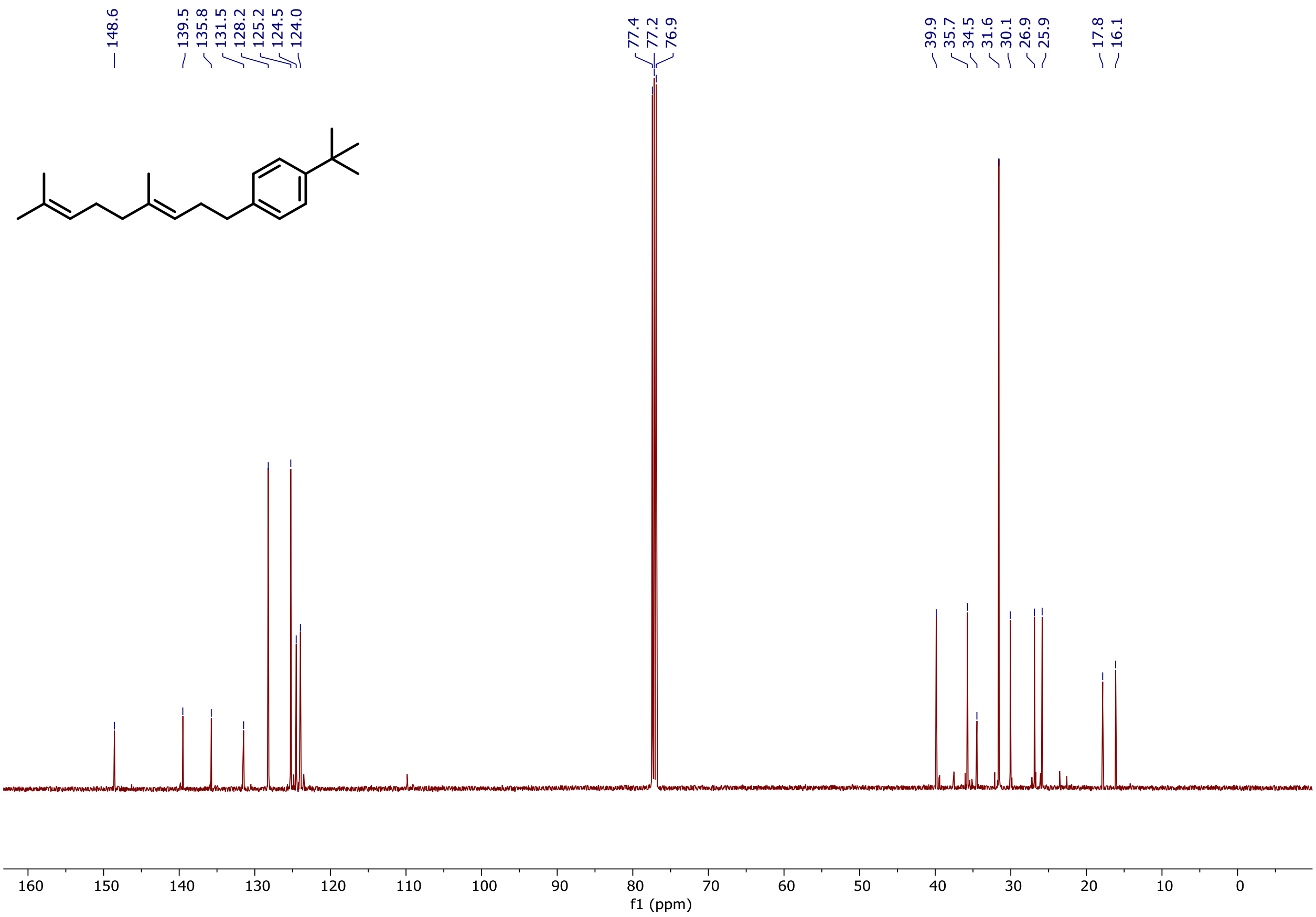

^{1}H NMR spectrum of **7e**

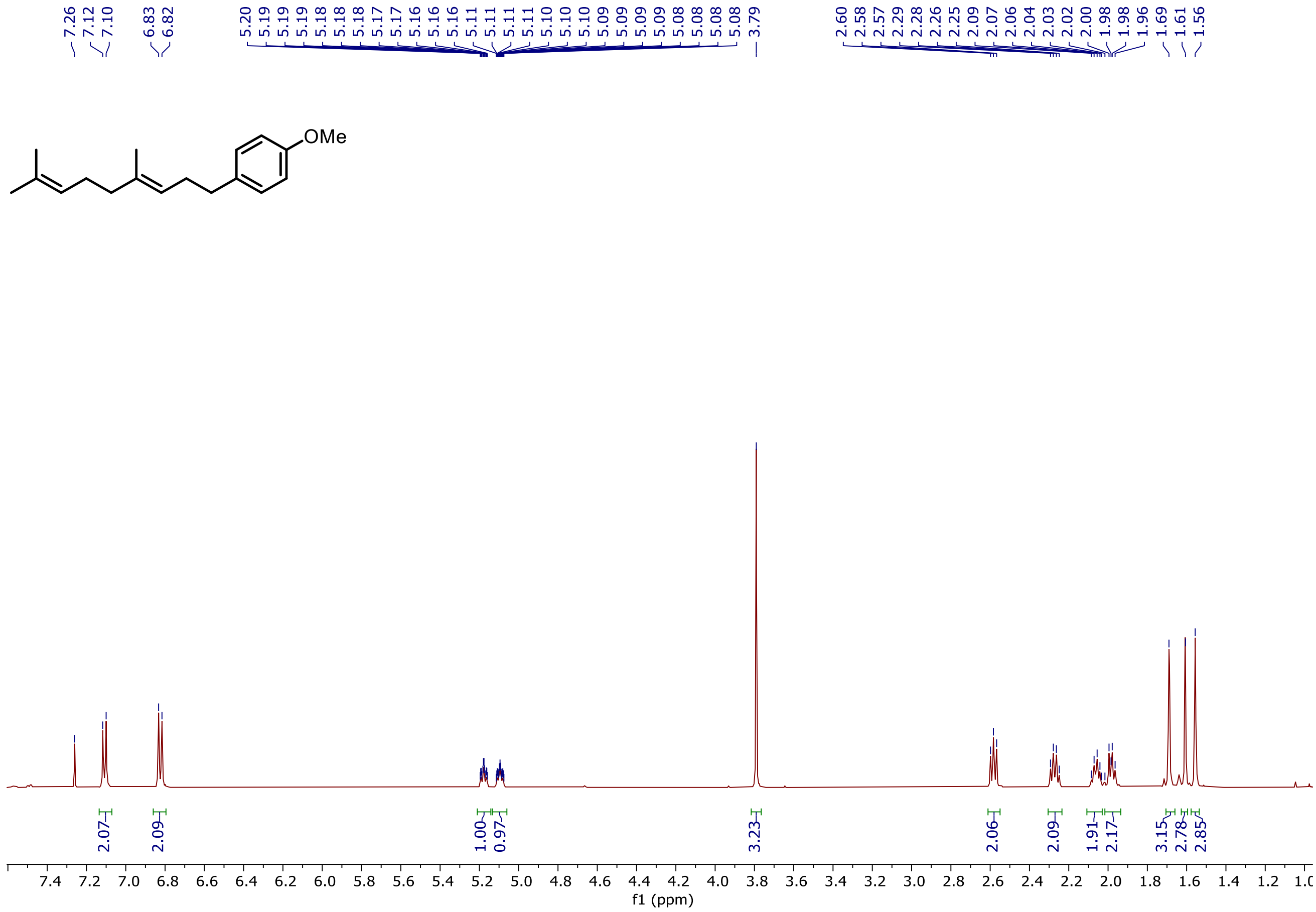

^{13}C NMR spectrum of **7e**

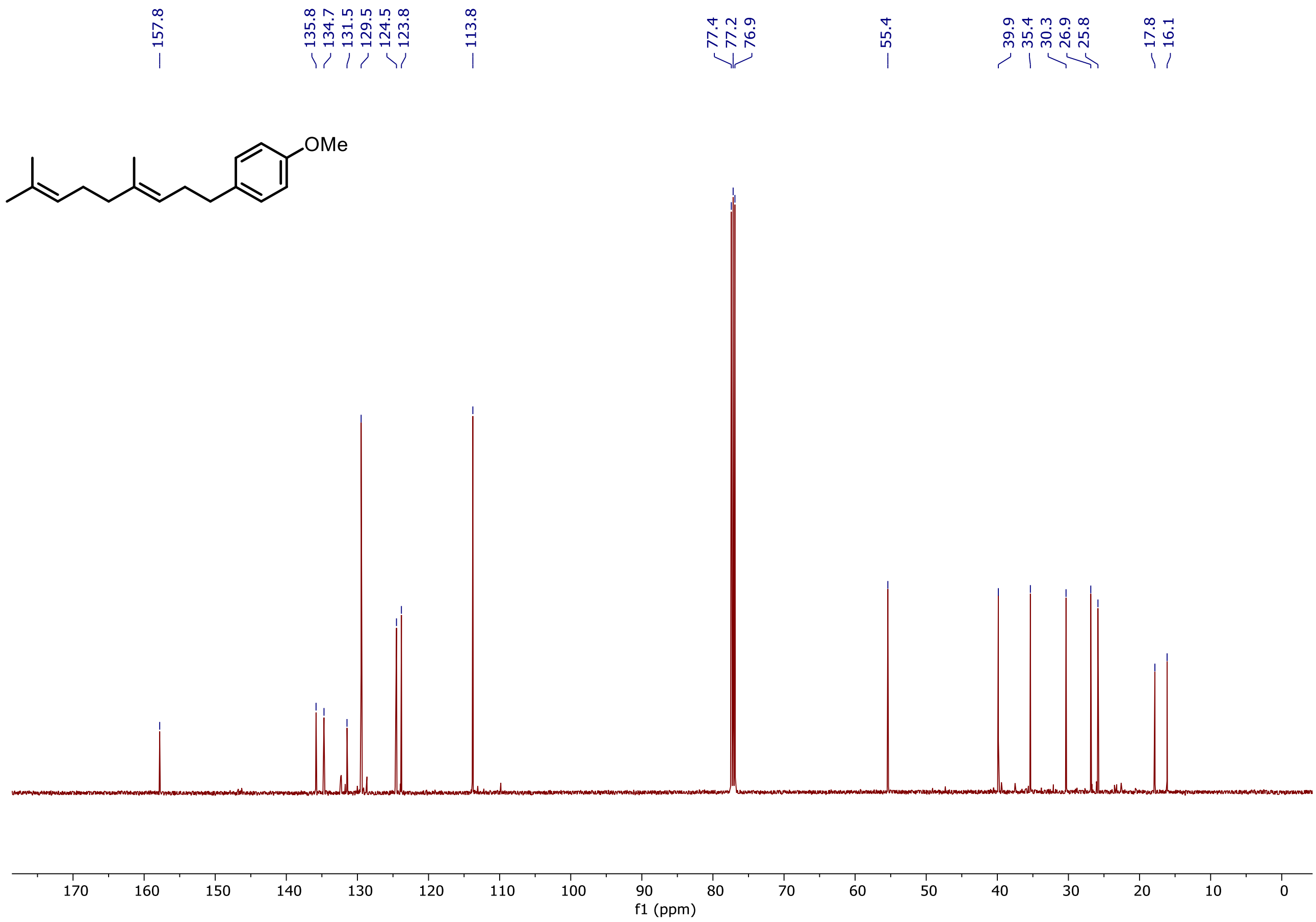

^{1}H NMR spectrum of **7f**

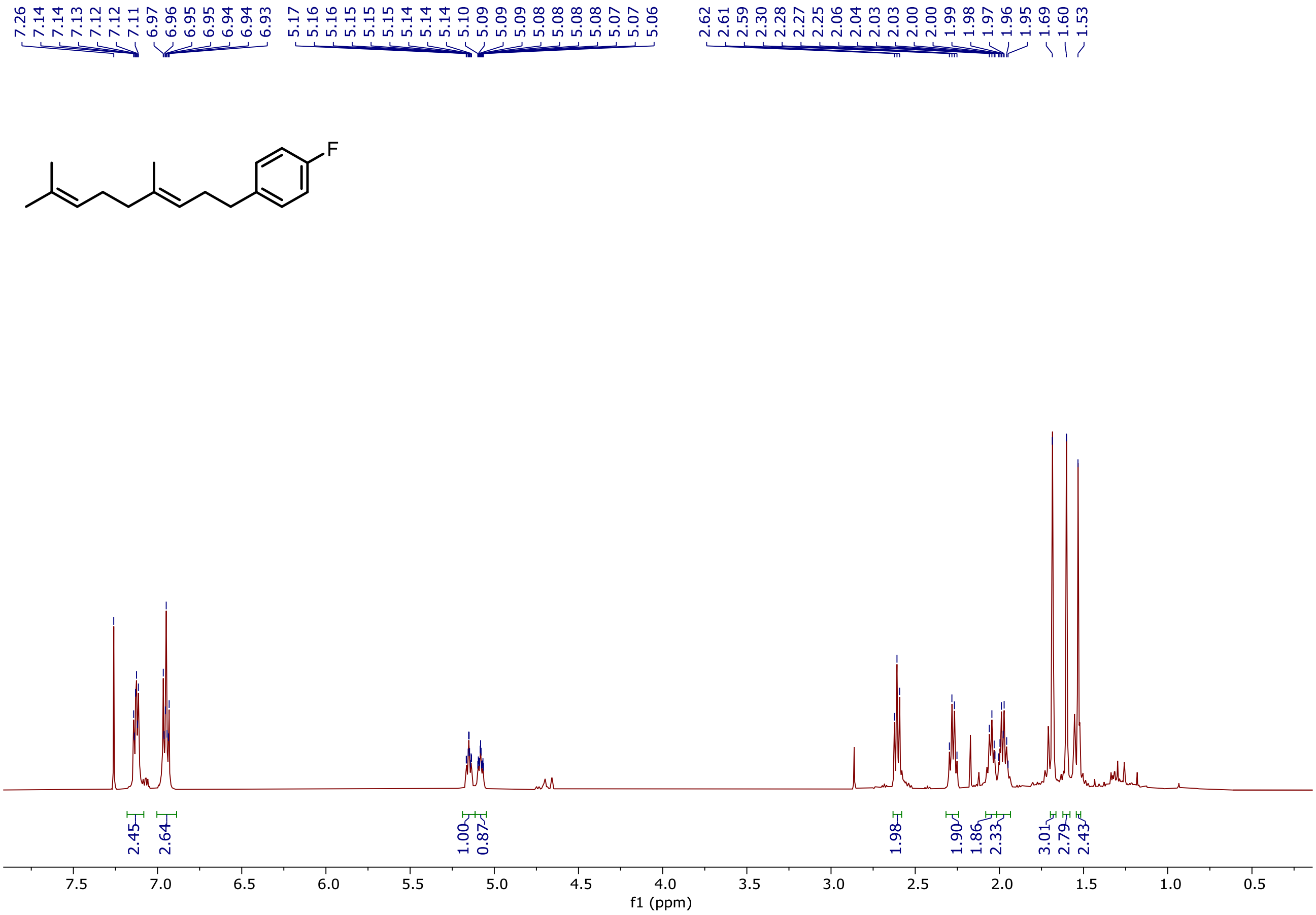

^{13}C NMR spectrum of **7f**

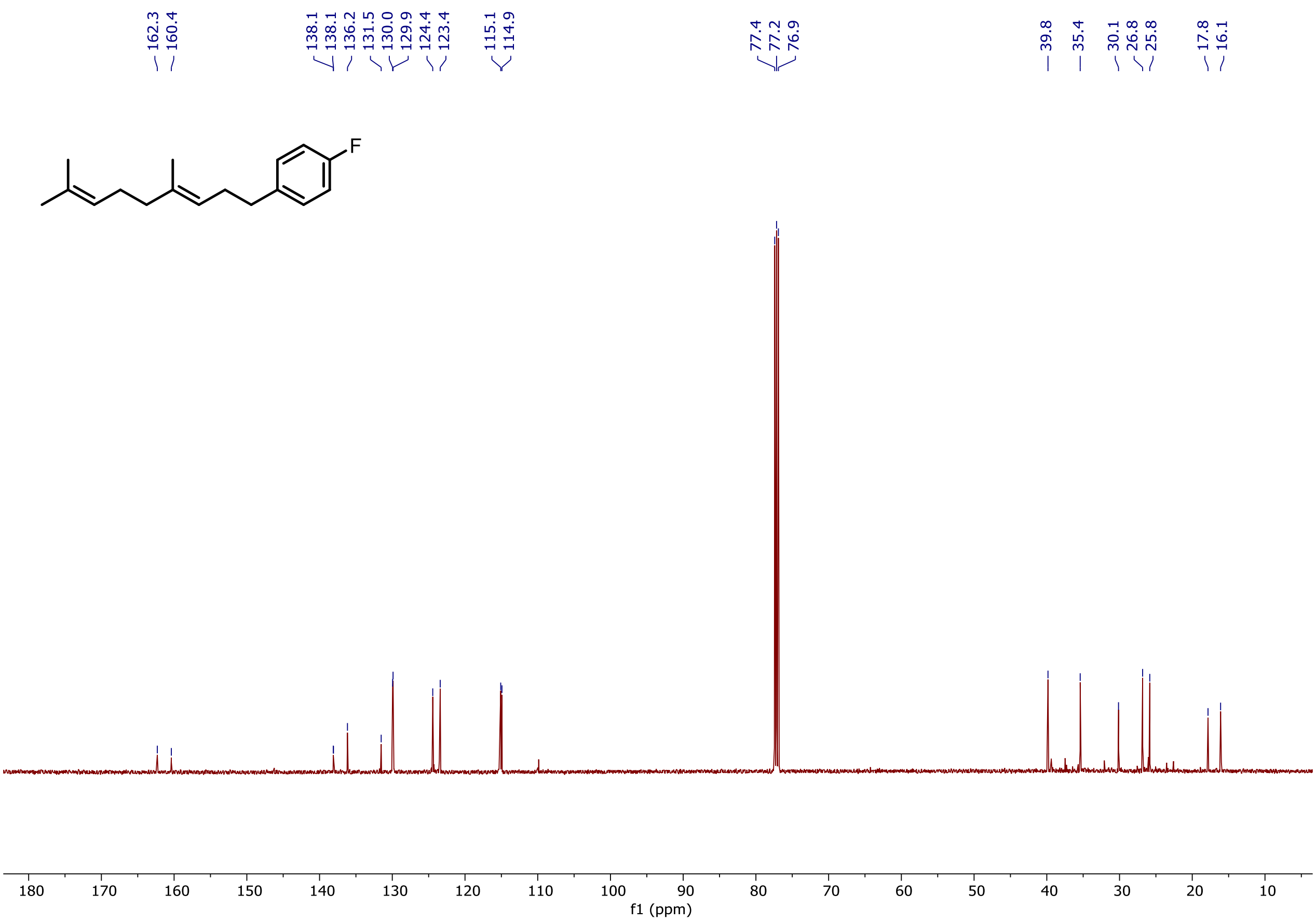

^{19}F NMR spectrum of **7f**

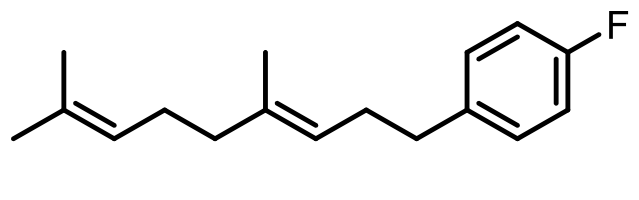

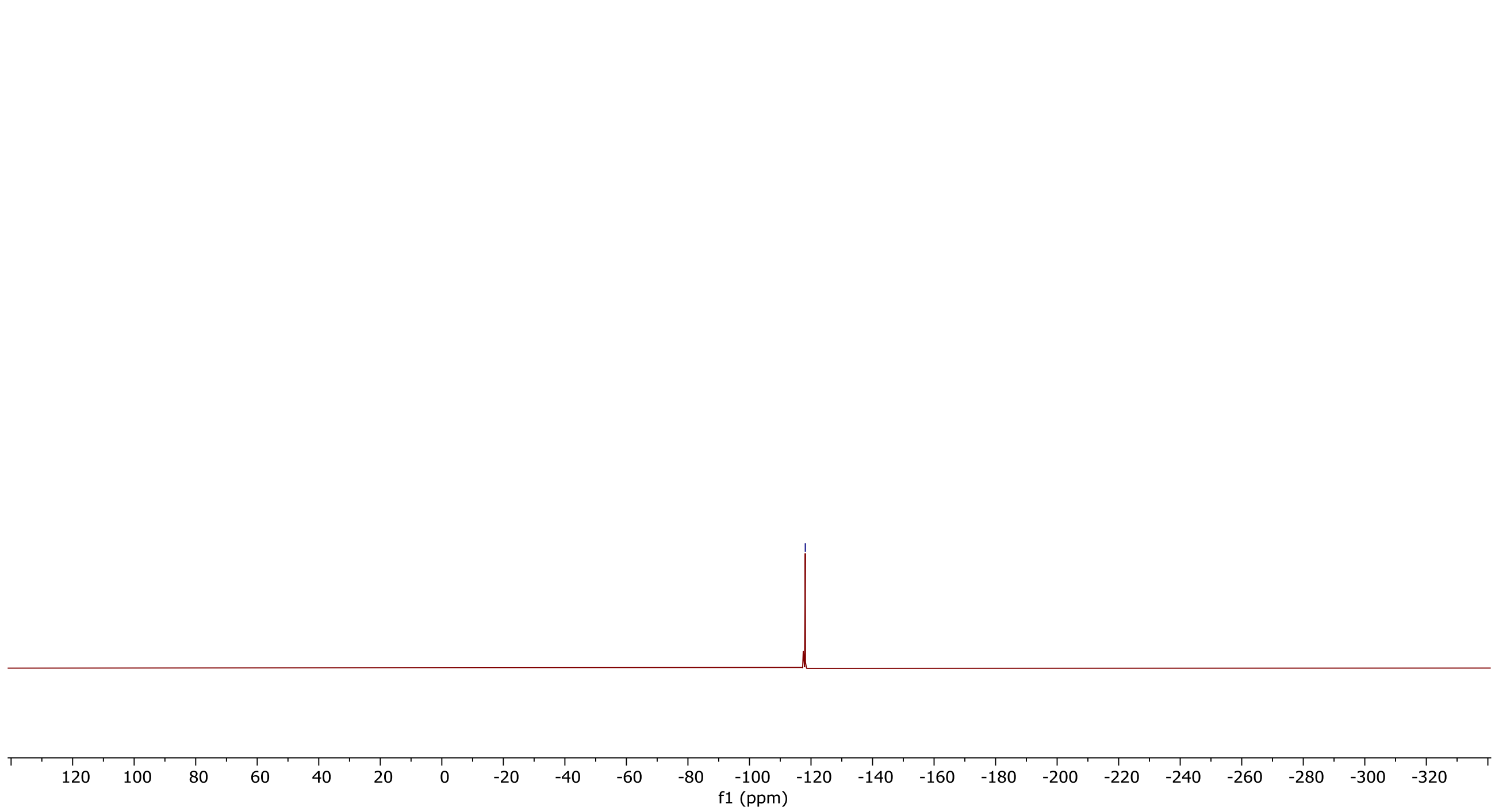

^{1}H NMR spectrum of **7g**

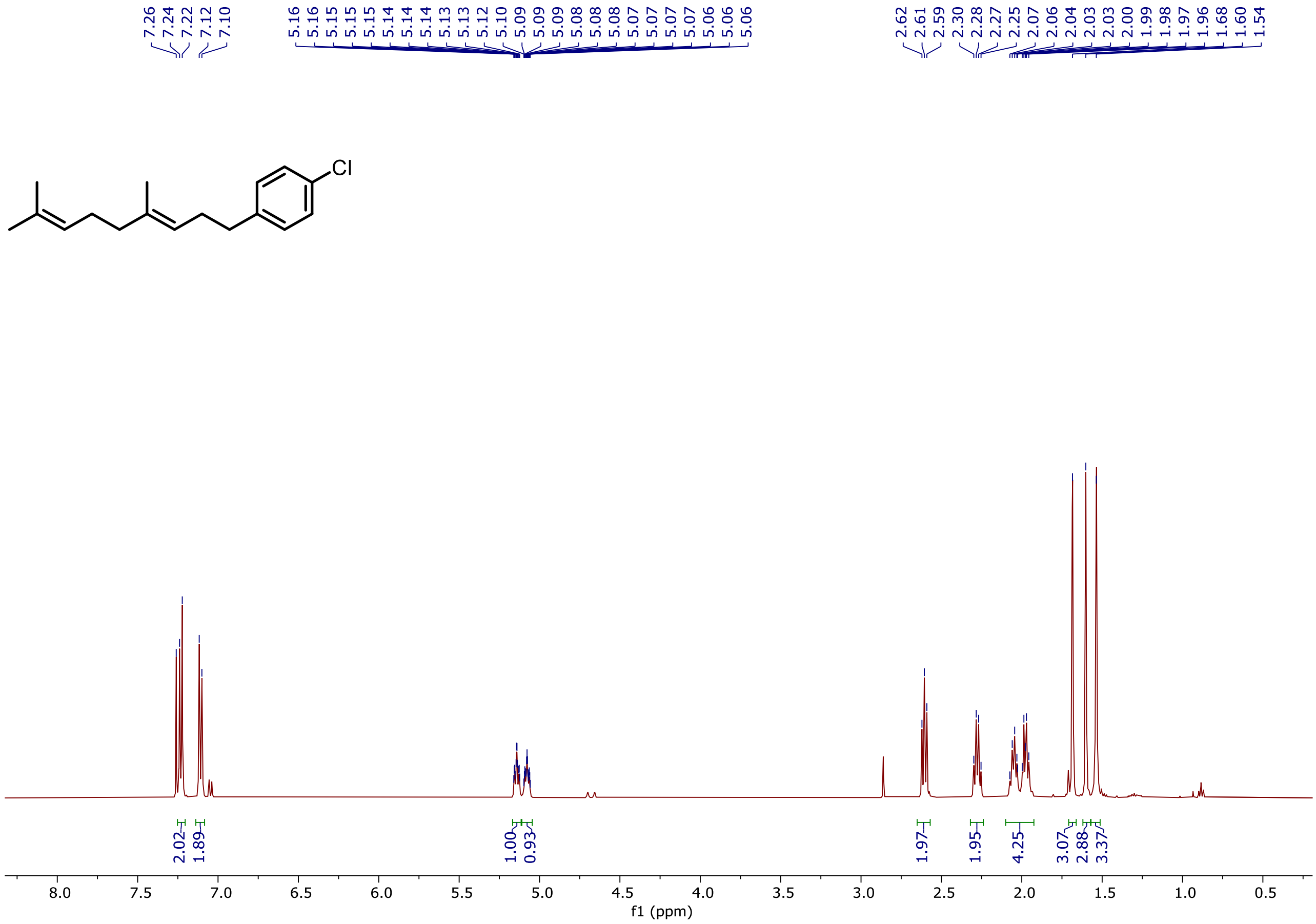

^{13}C NMR spectrum of **7g**

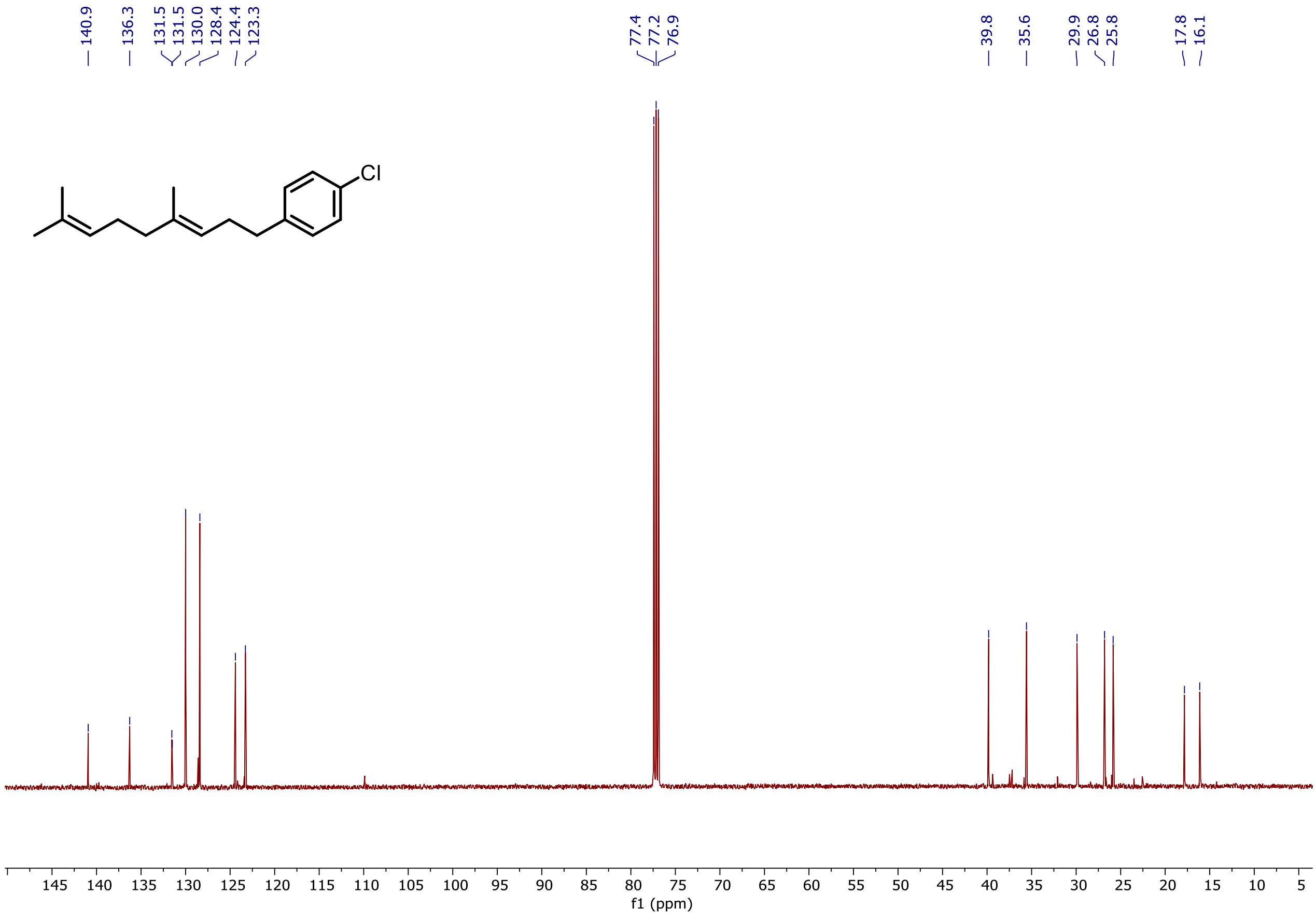

^{1}H NMR spectrum of **7h**

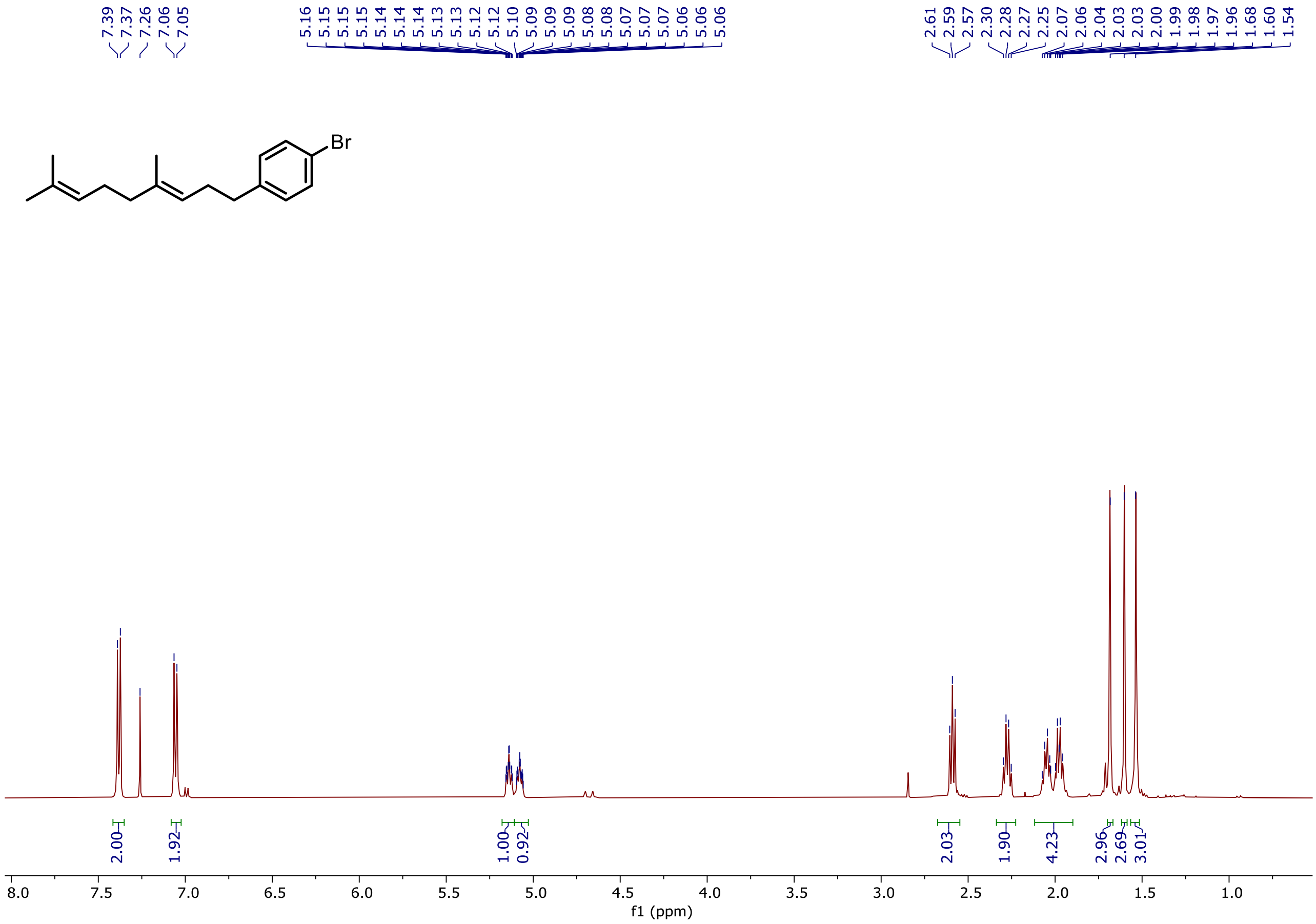

^{13}C NMR spectrum of **7h**

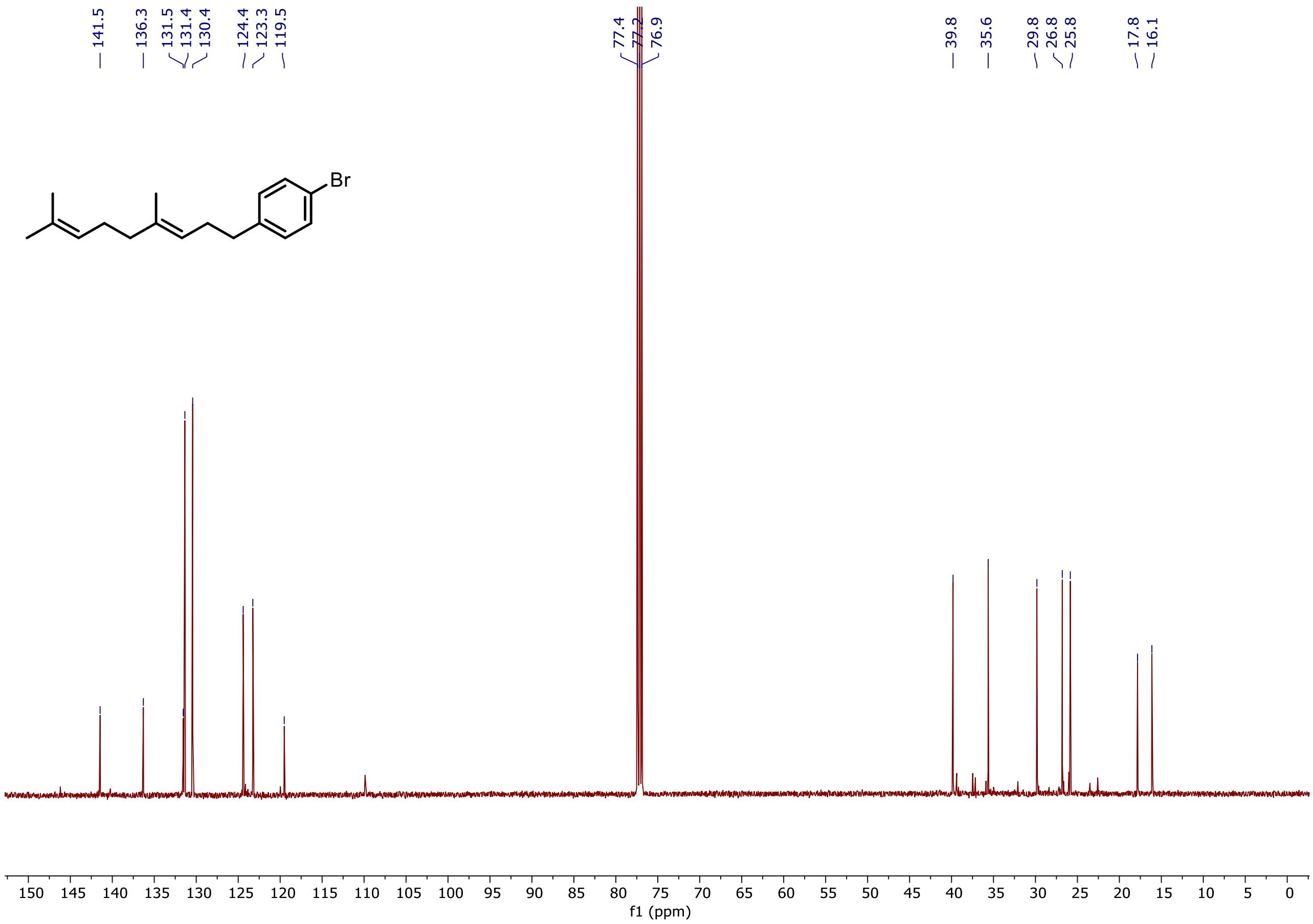

^{1}H NMR spectrum of **9a**

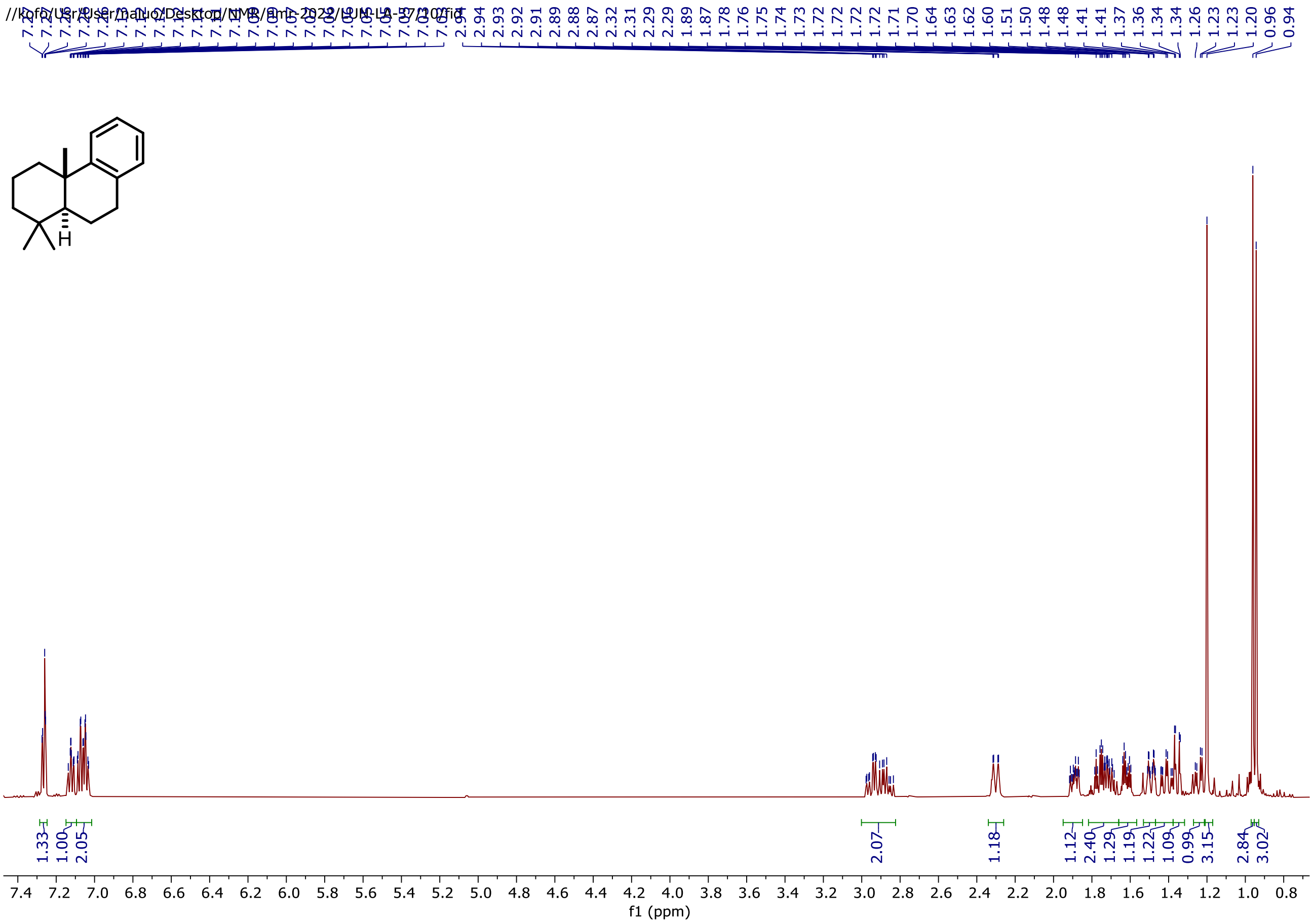

^{13}C NMR spectrum of **9a**

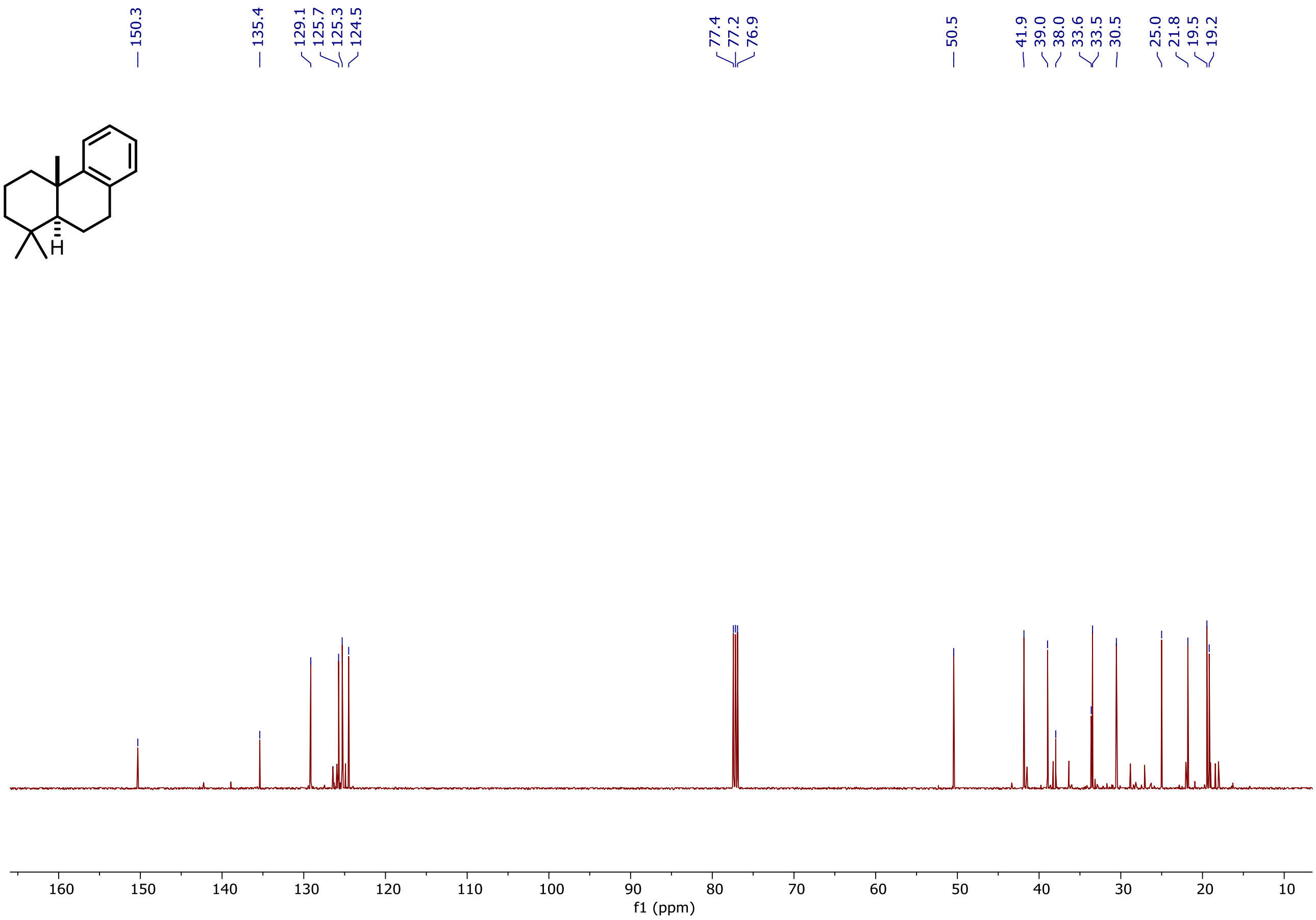

^{1}H NMR spectrum of **9b**

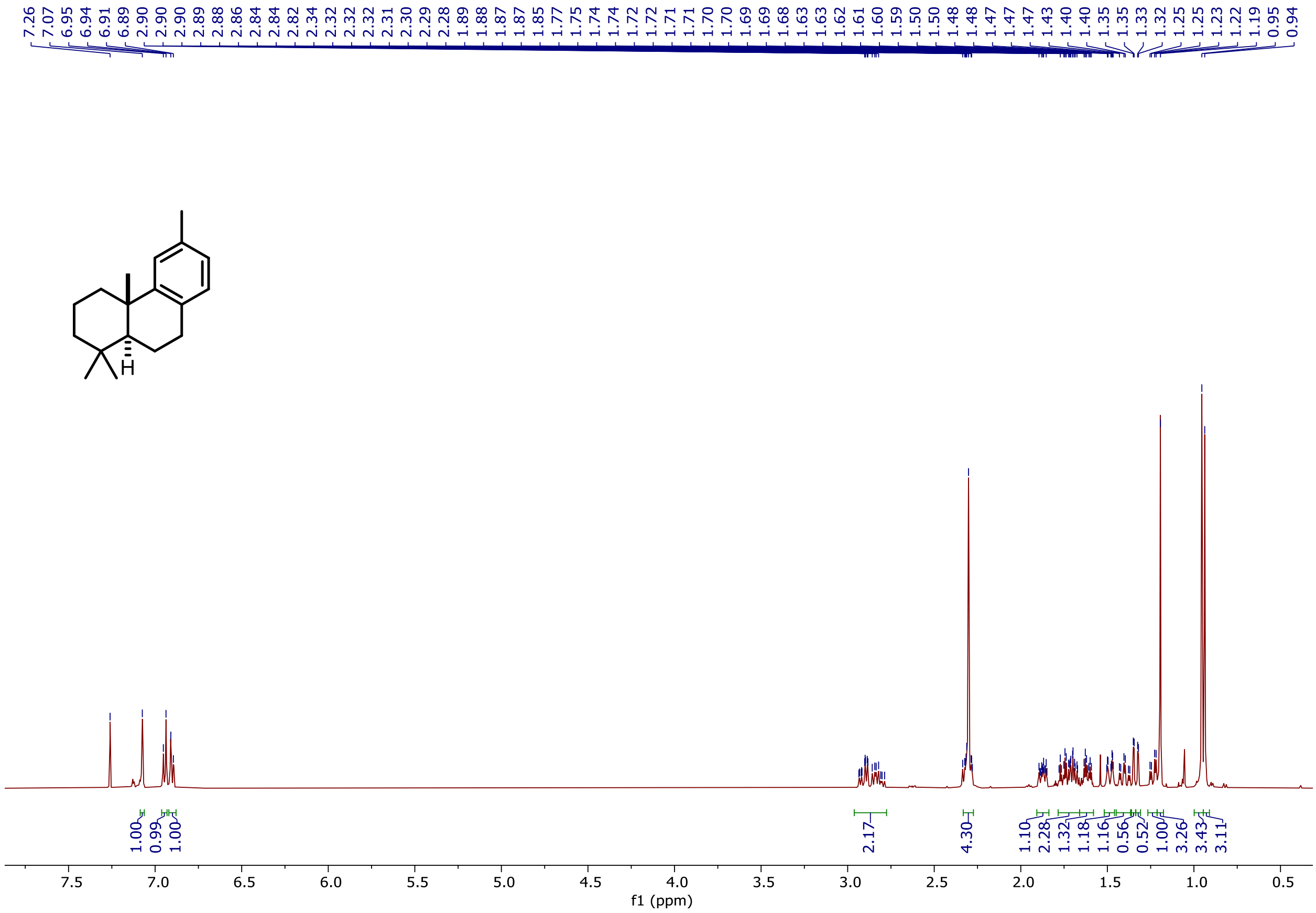

^{13}C NMR spectrum of **9b**

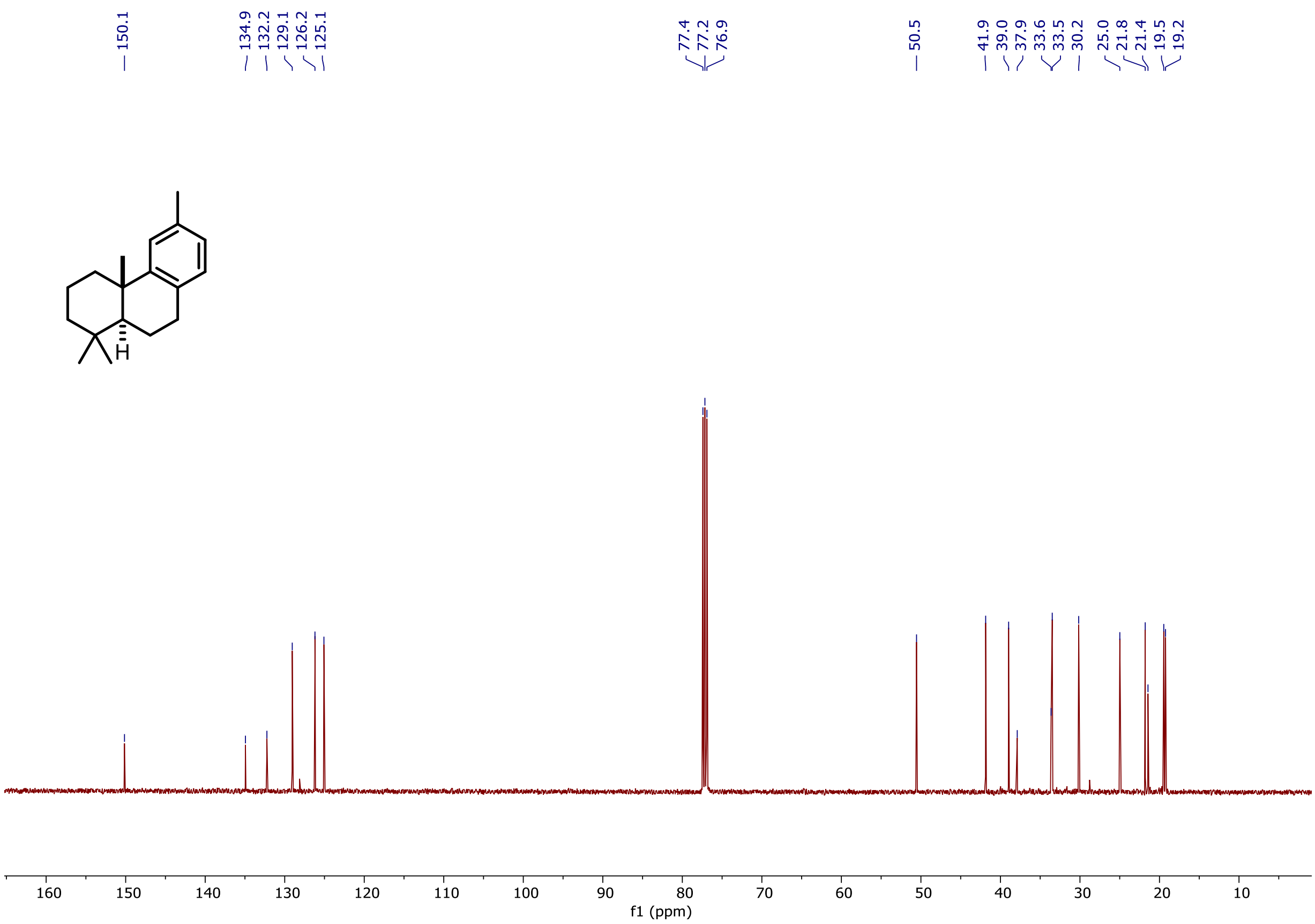

^{1}H NMR spectrum of **9c**

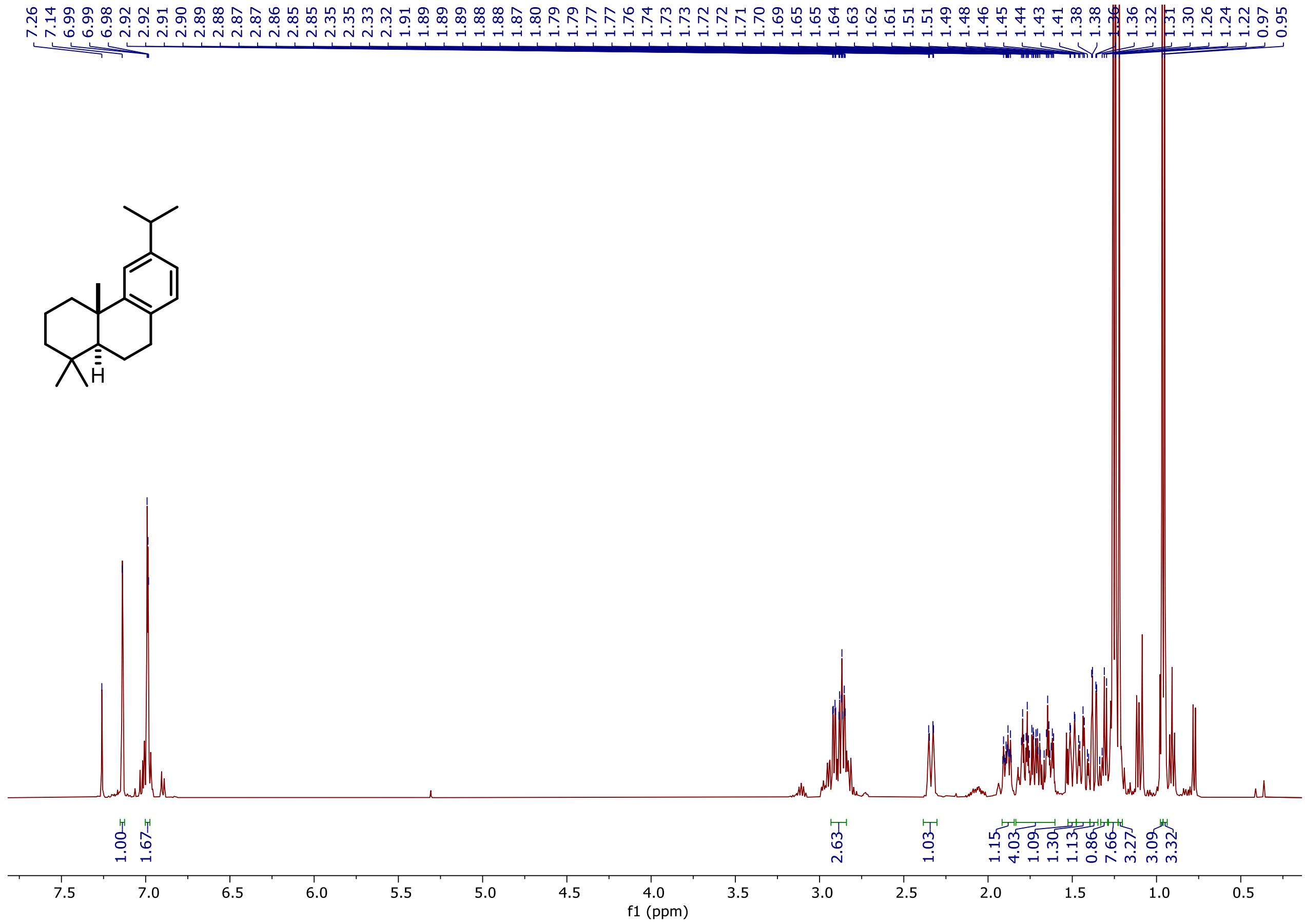

^{13}C NMR spectrum of **9c**

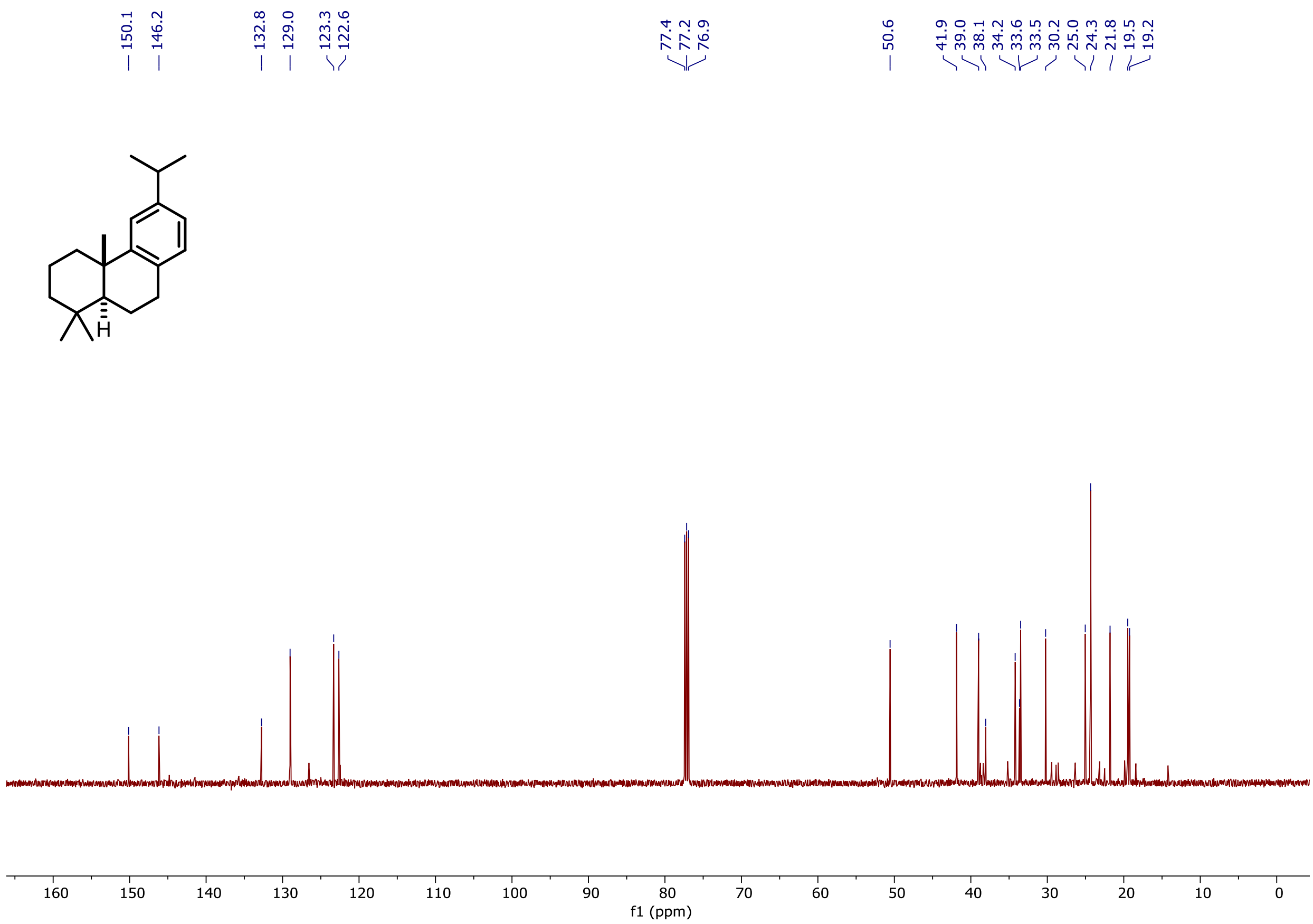

^{1}H NMR spectrum of **9d**

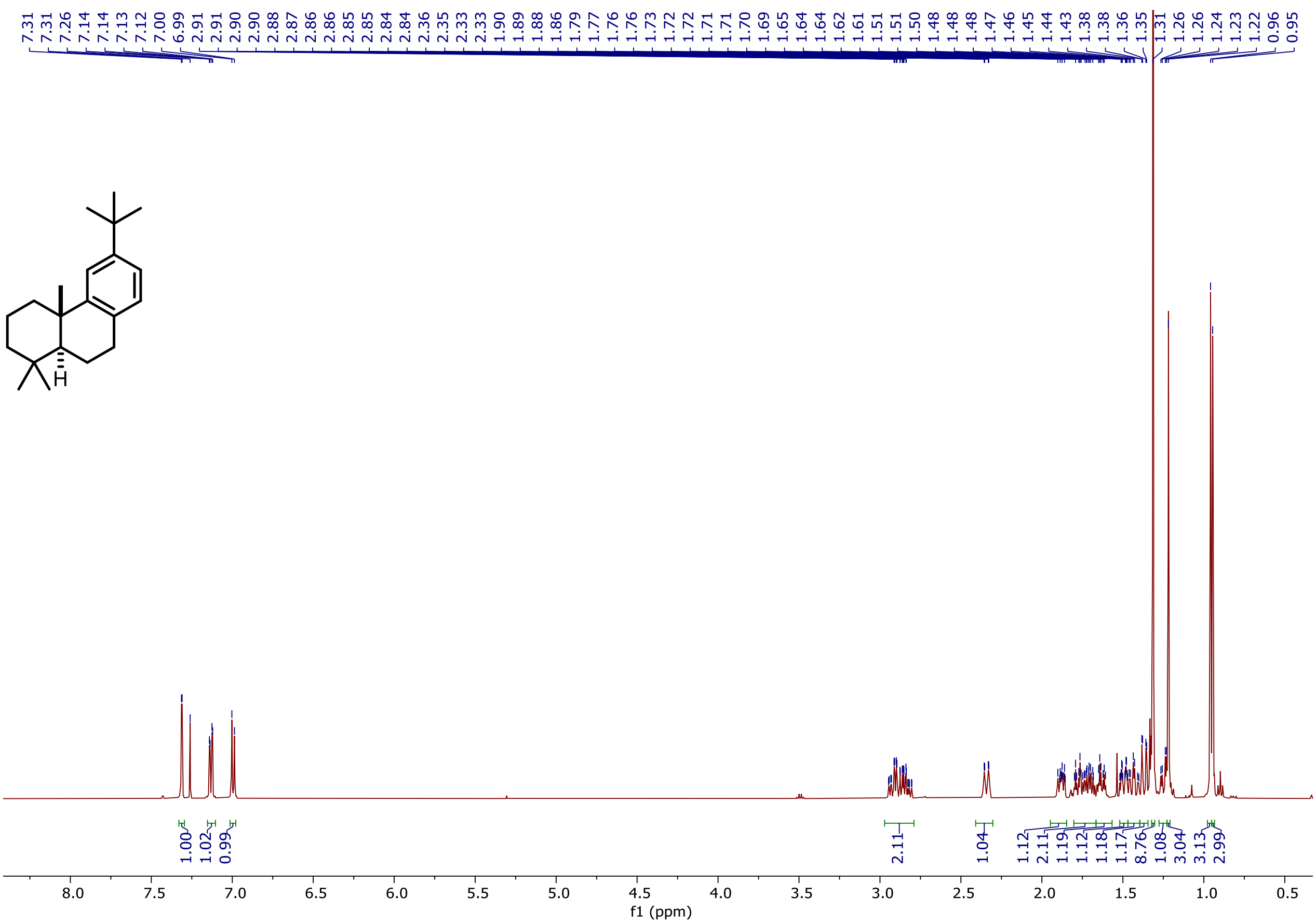

^{13}C NMR spectrum of **9d**

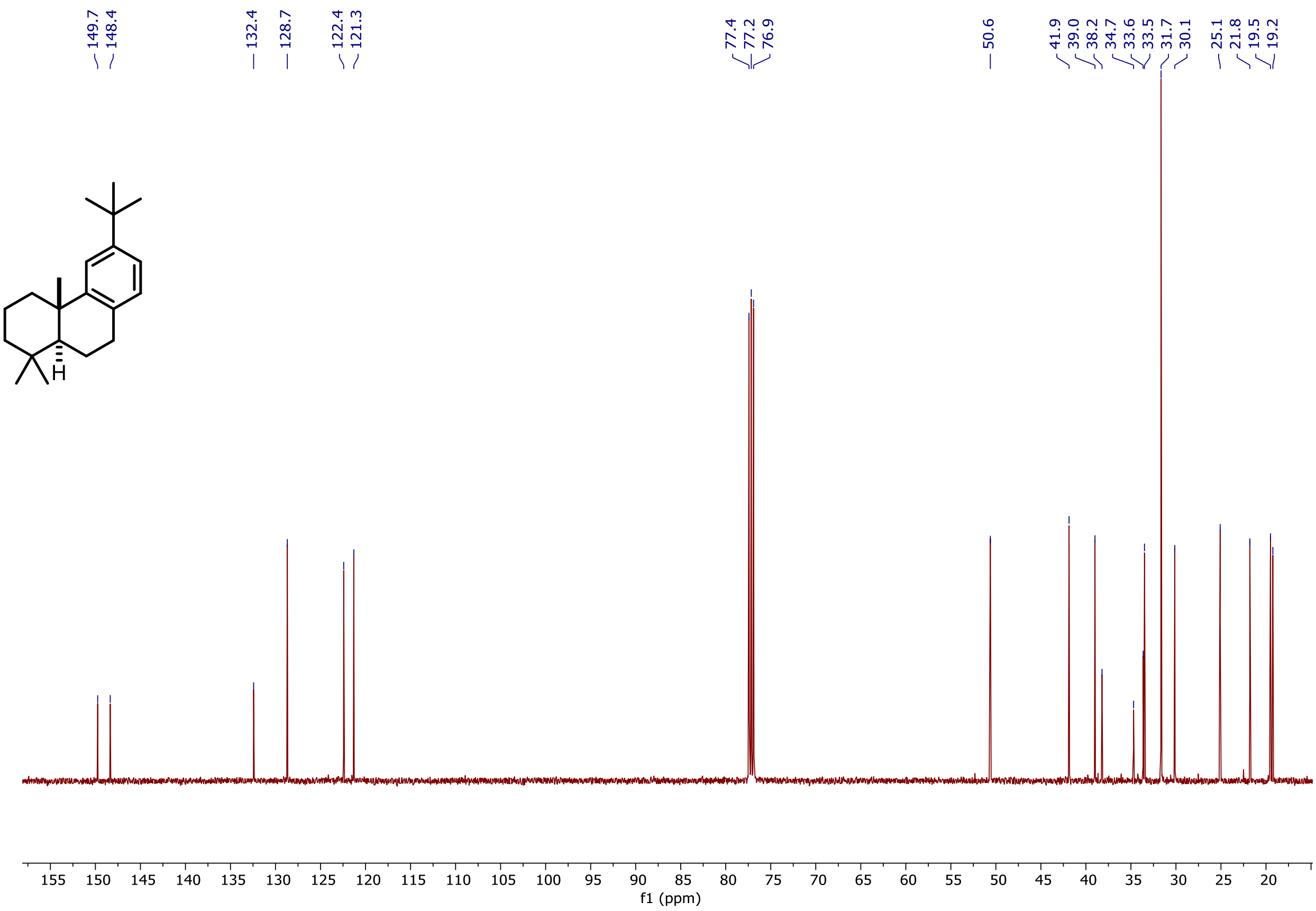

^{1}H NMR spectrum of **9e**

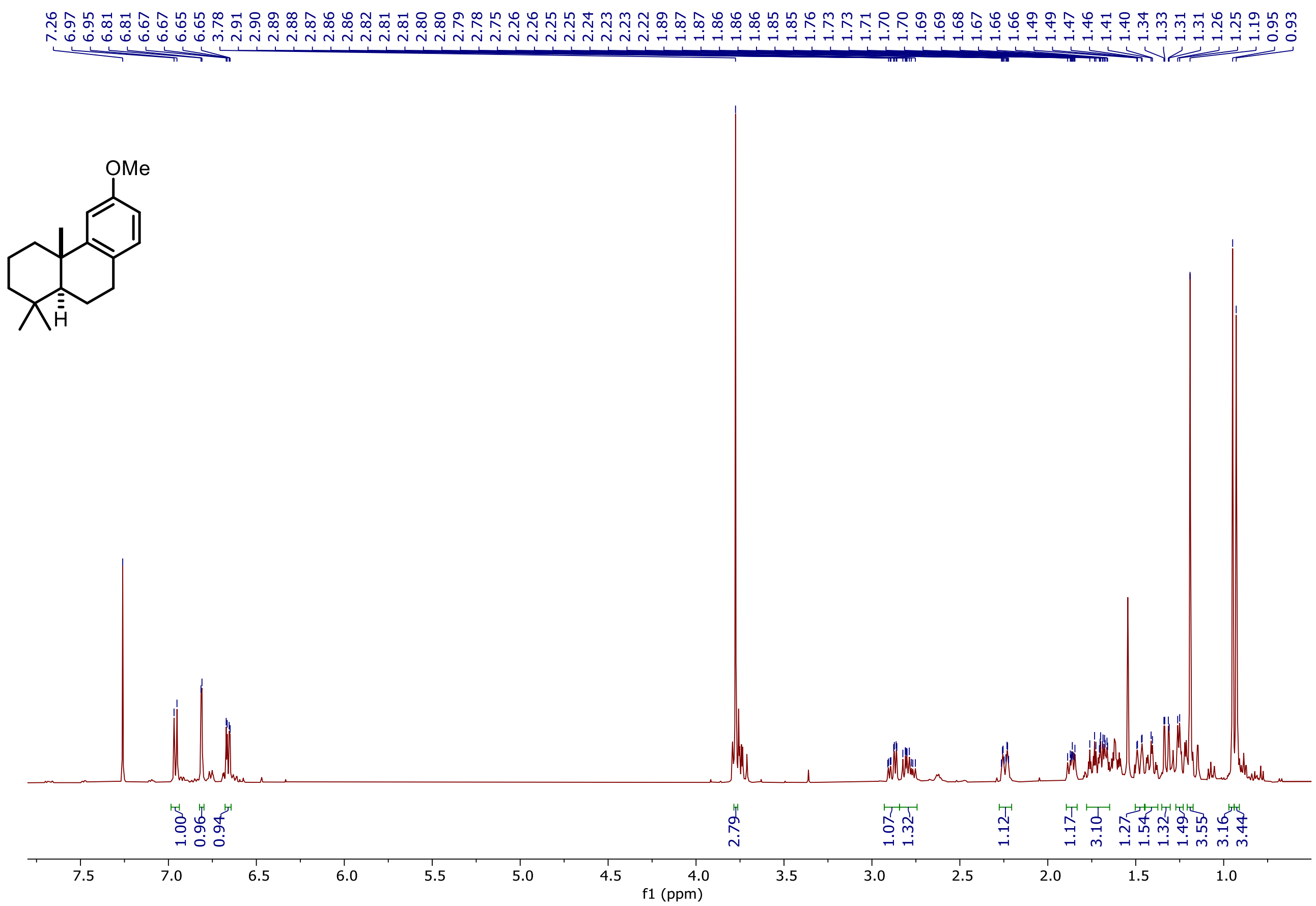

^{13}C NMR spectrum of **9e**

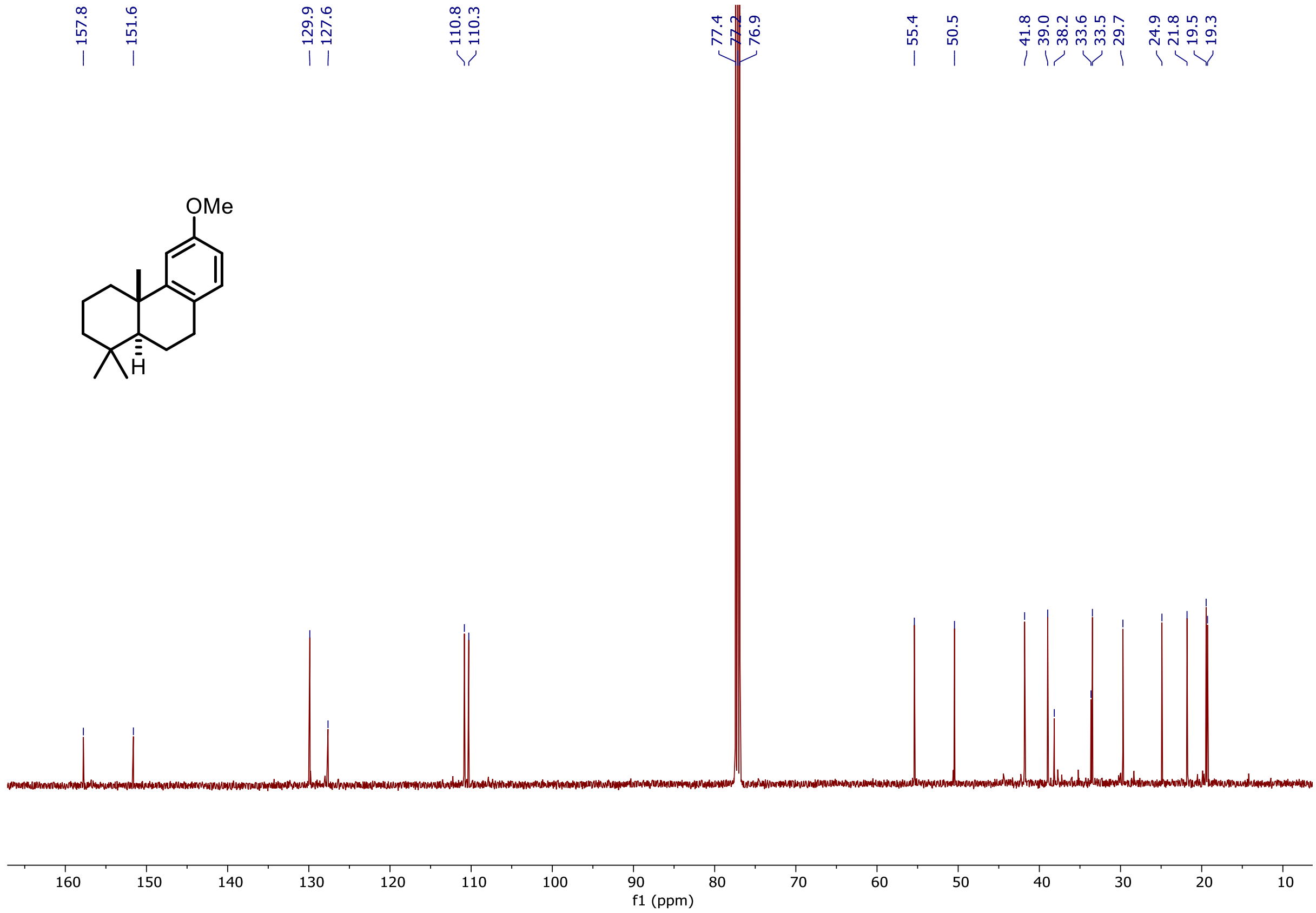

^{1}H NMR spectrum of **9f**

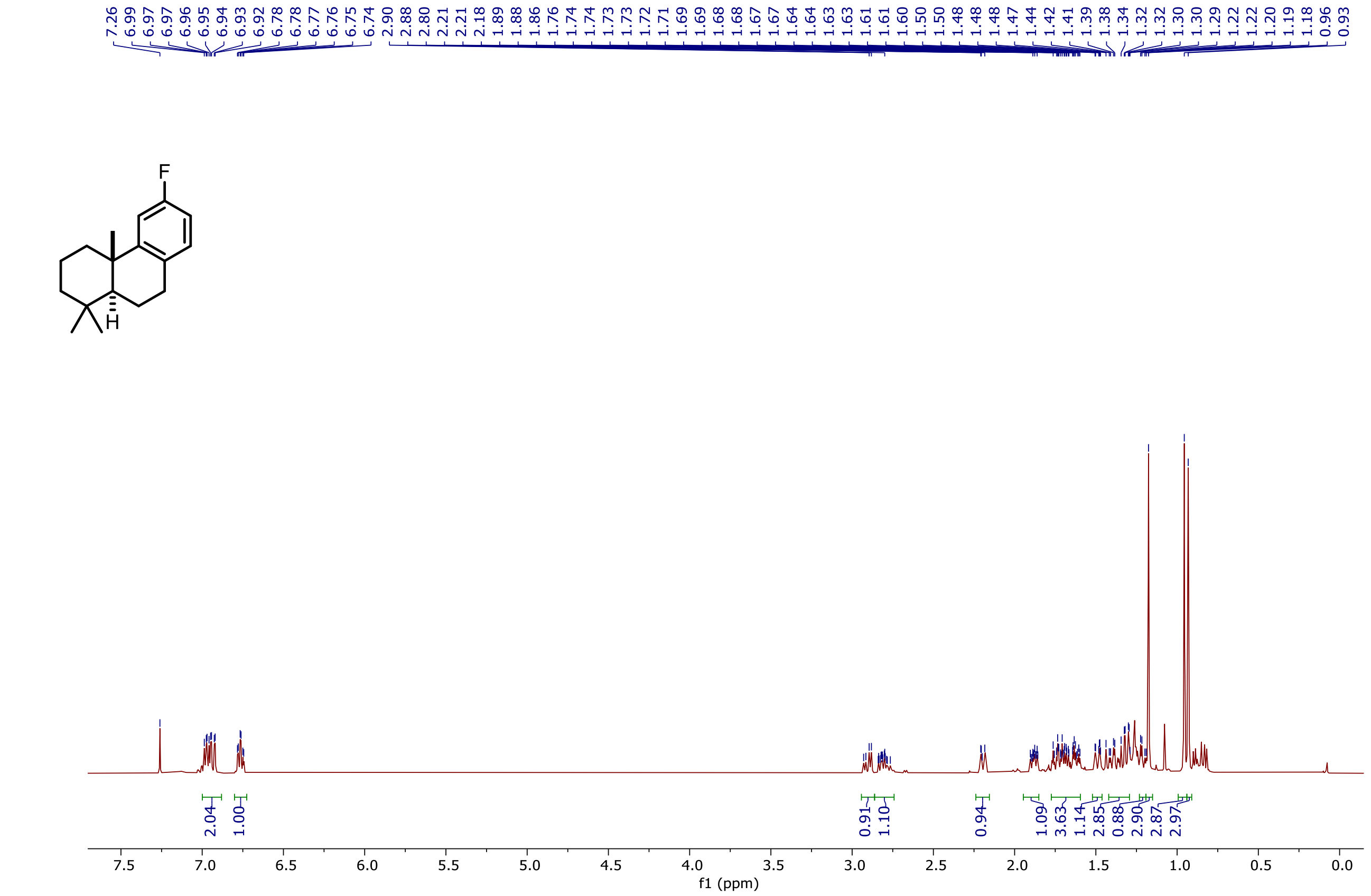

^{13}C NMR spectrum of **9f**

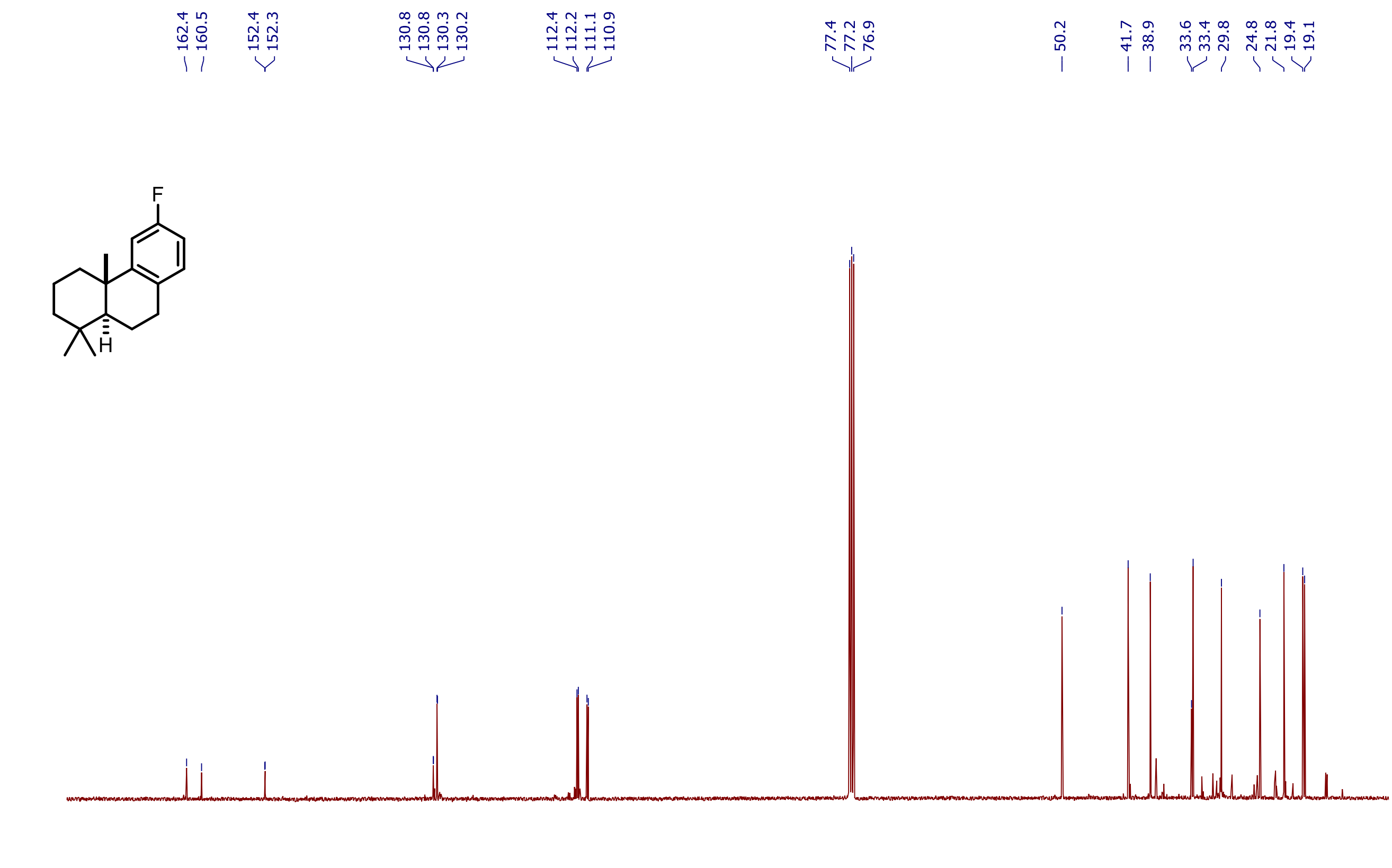

^{19}F NMR spectrum of **9f**

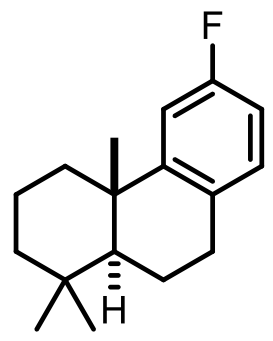

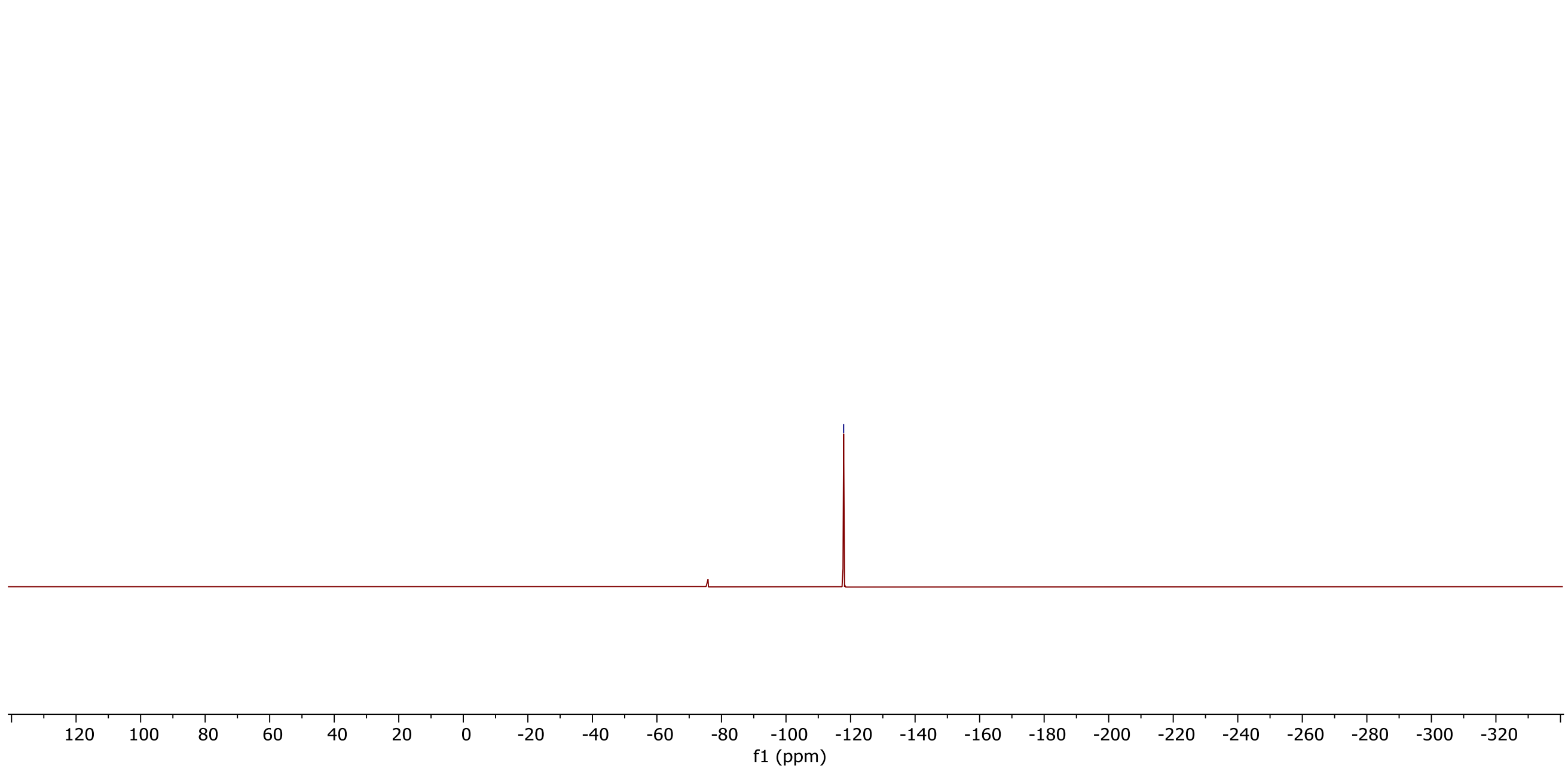

^{1}H NMR spectrum of **9g**

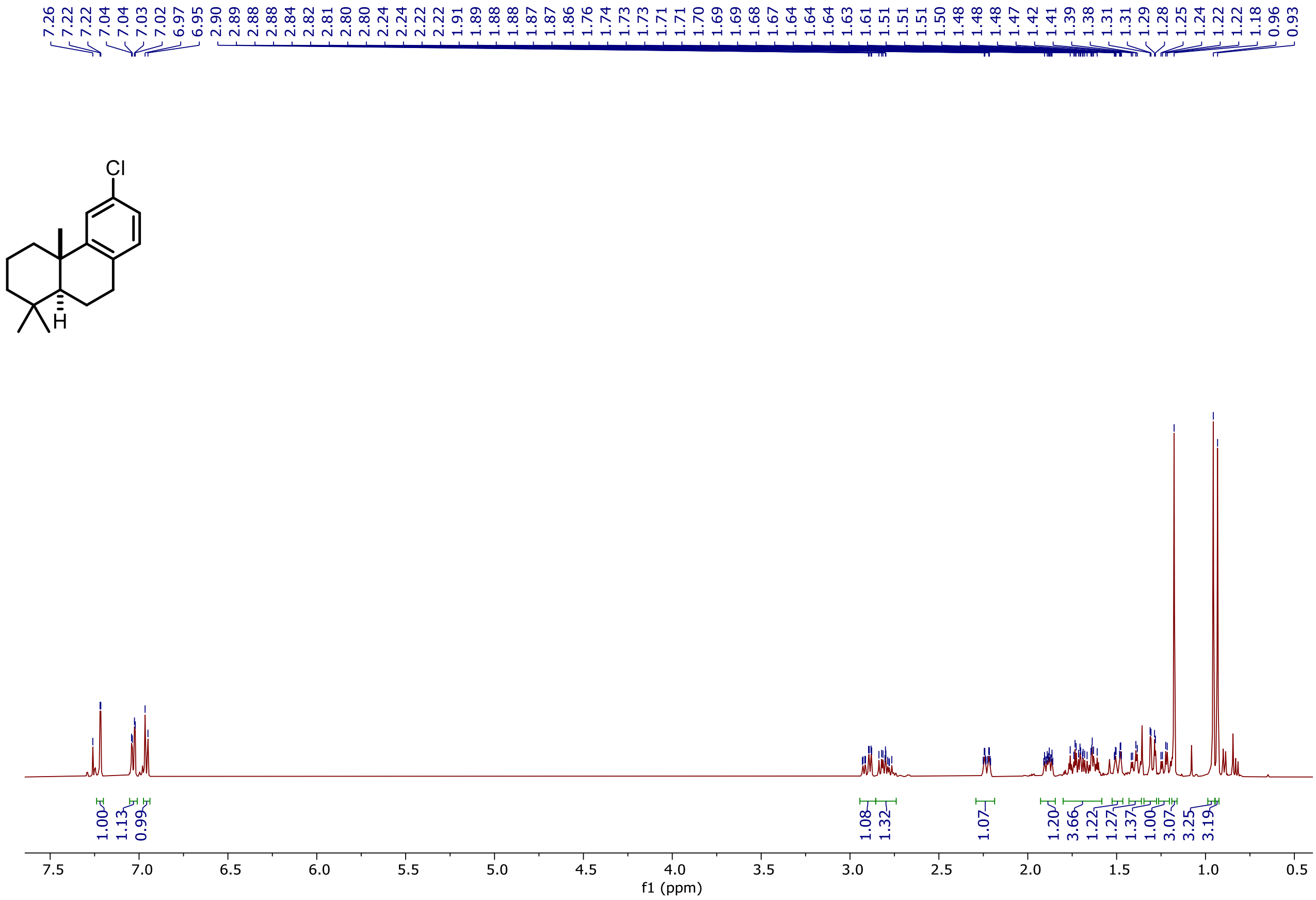

^{13}C NMR spectrum of **9g**

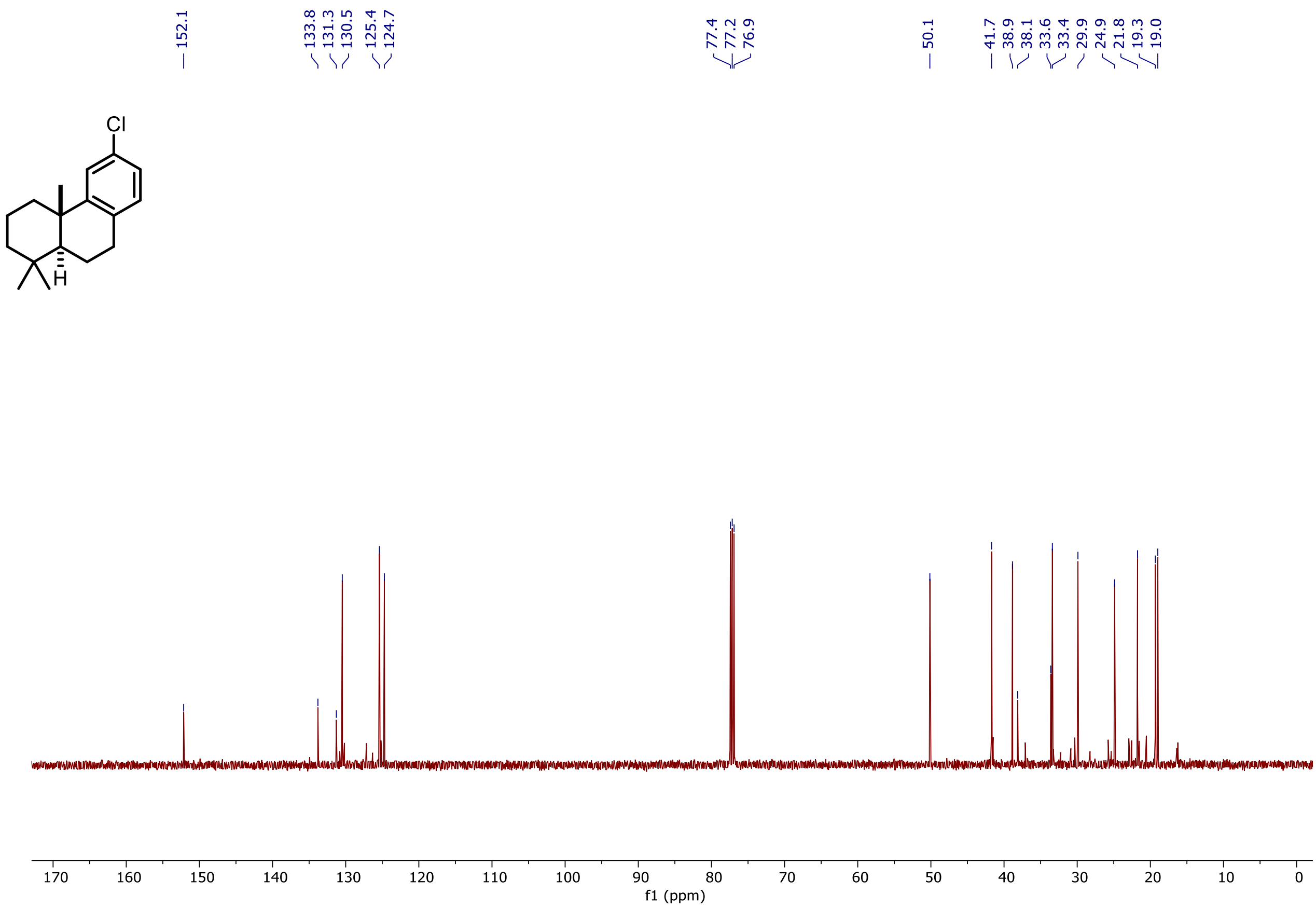

^{1}H NMR spectrum of **9h**

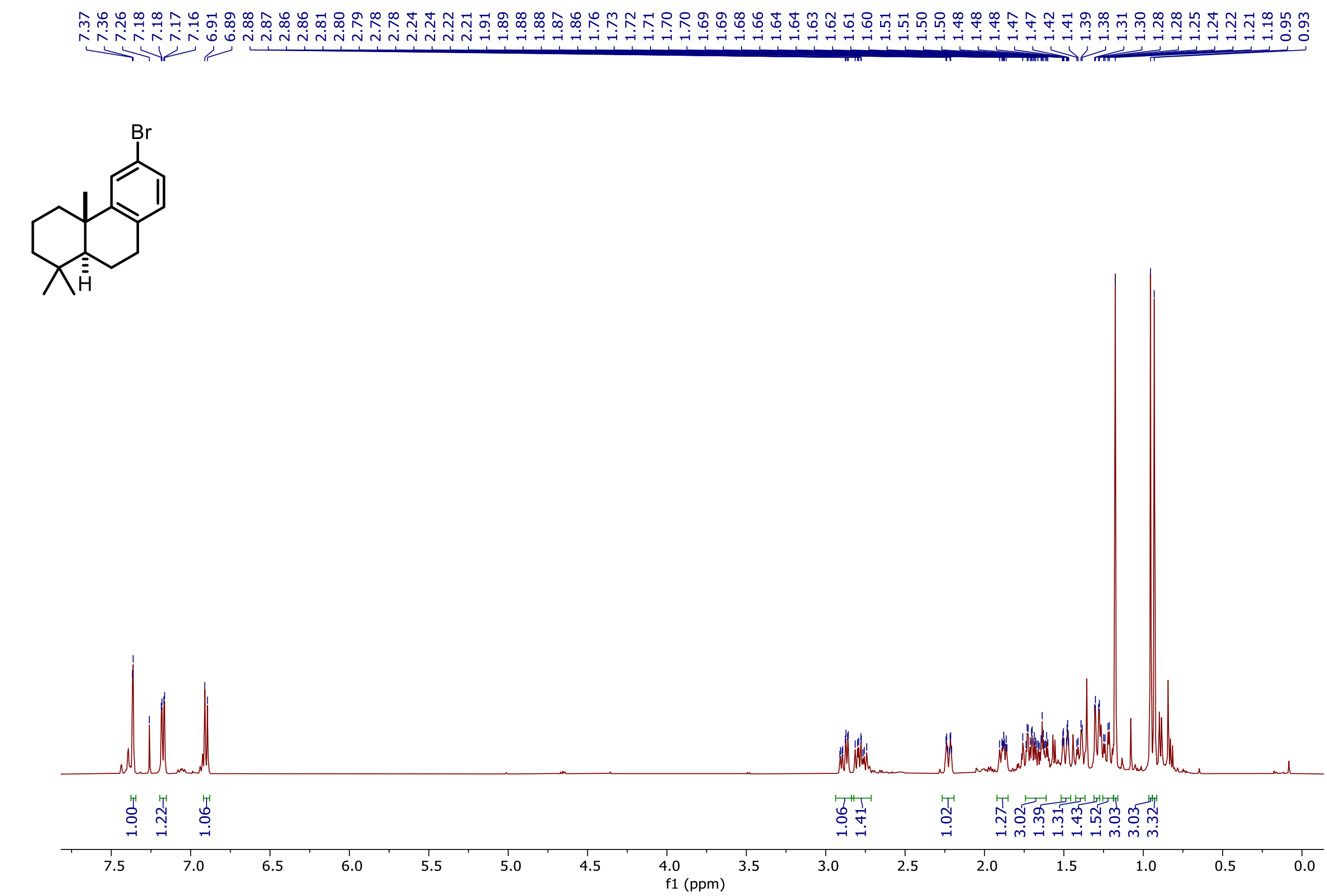

^{13}C NMR spectrum of **9h**

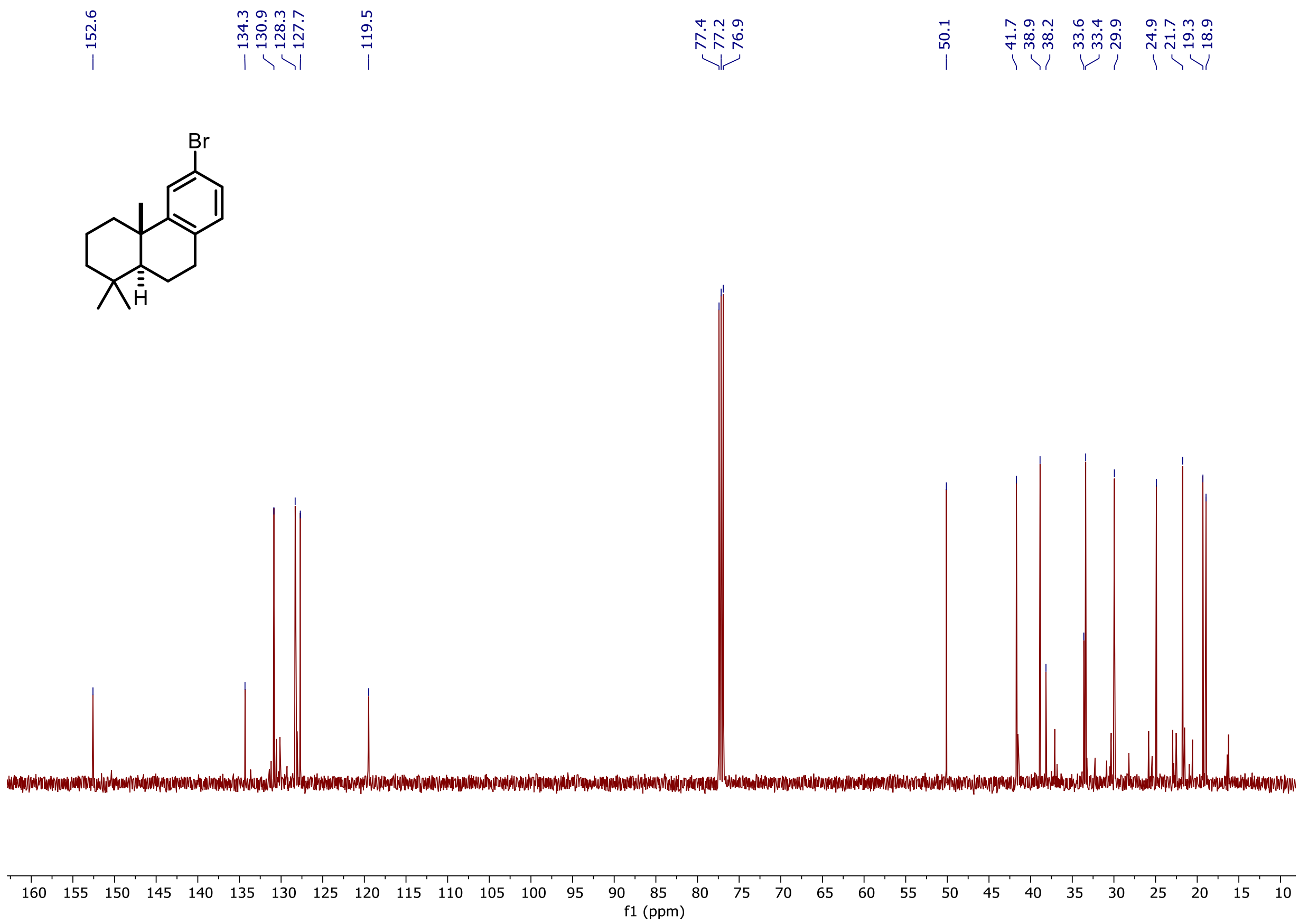

^{1}H NMR spectrum of **10a**

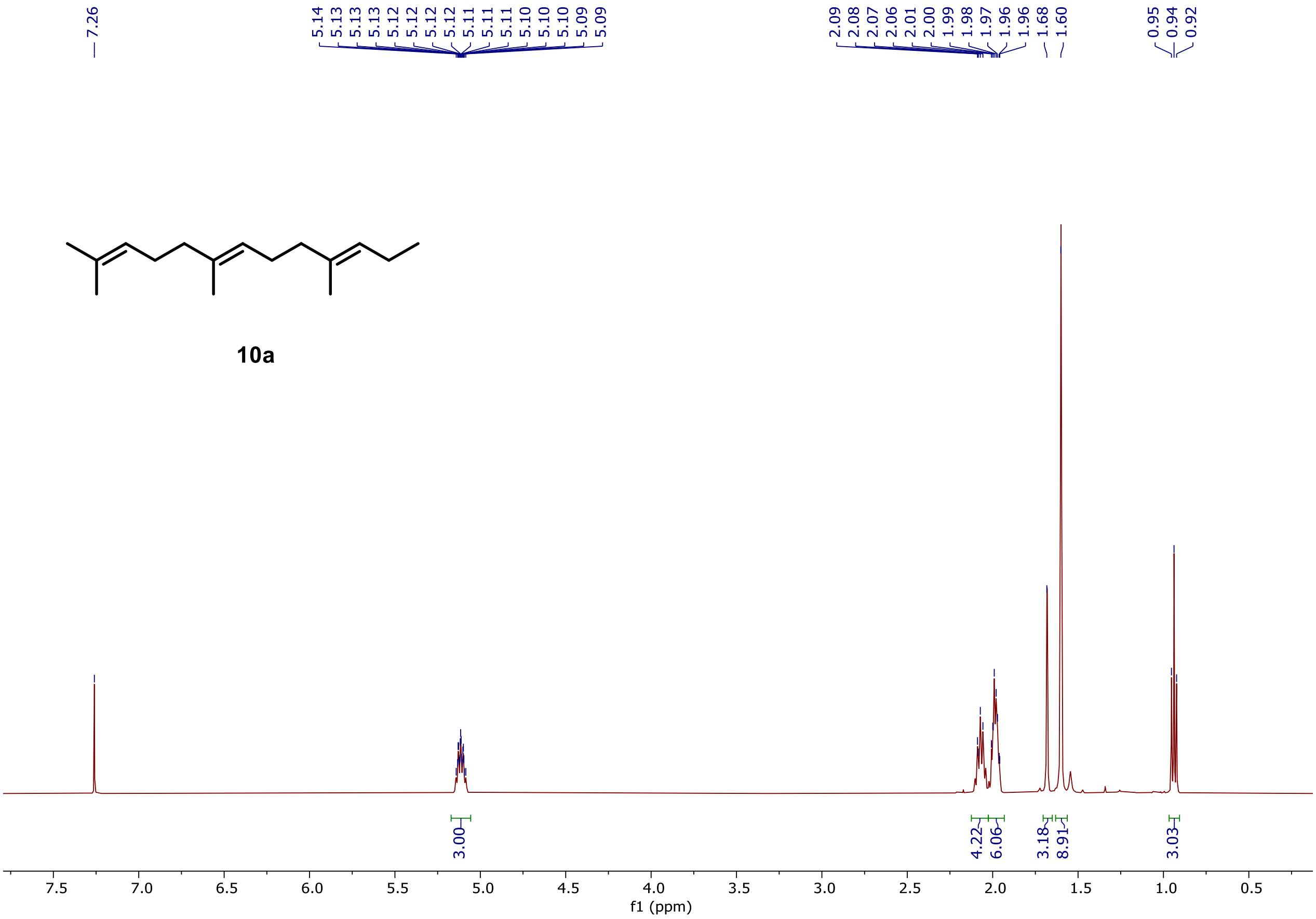

^{13}C NMR spectrum of **10a**

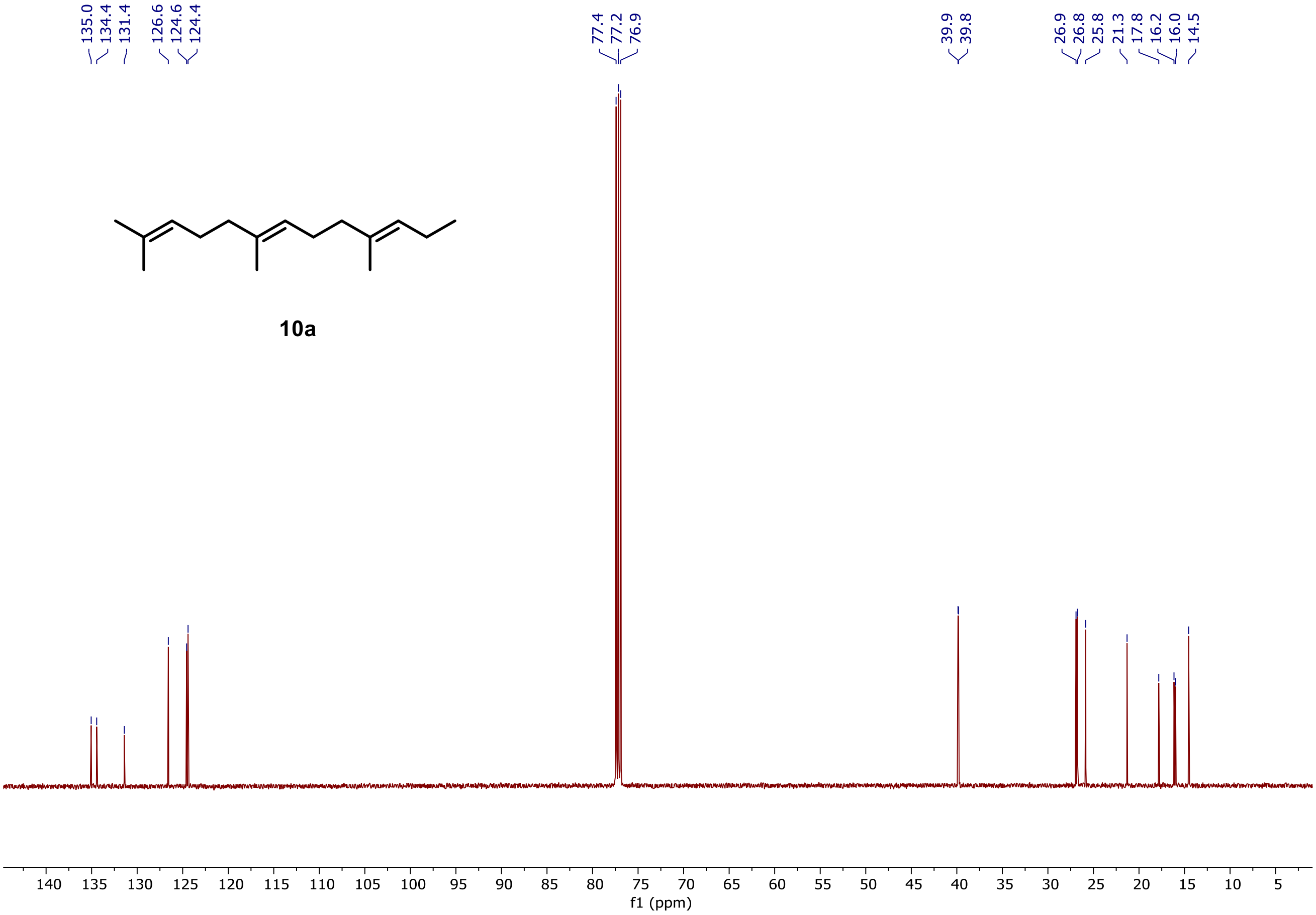

^{1}H NMR spectrum of **11a**

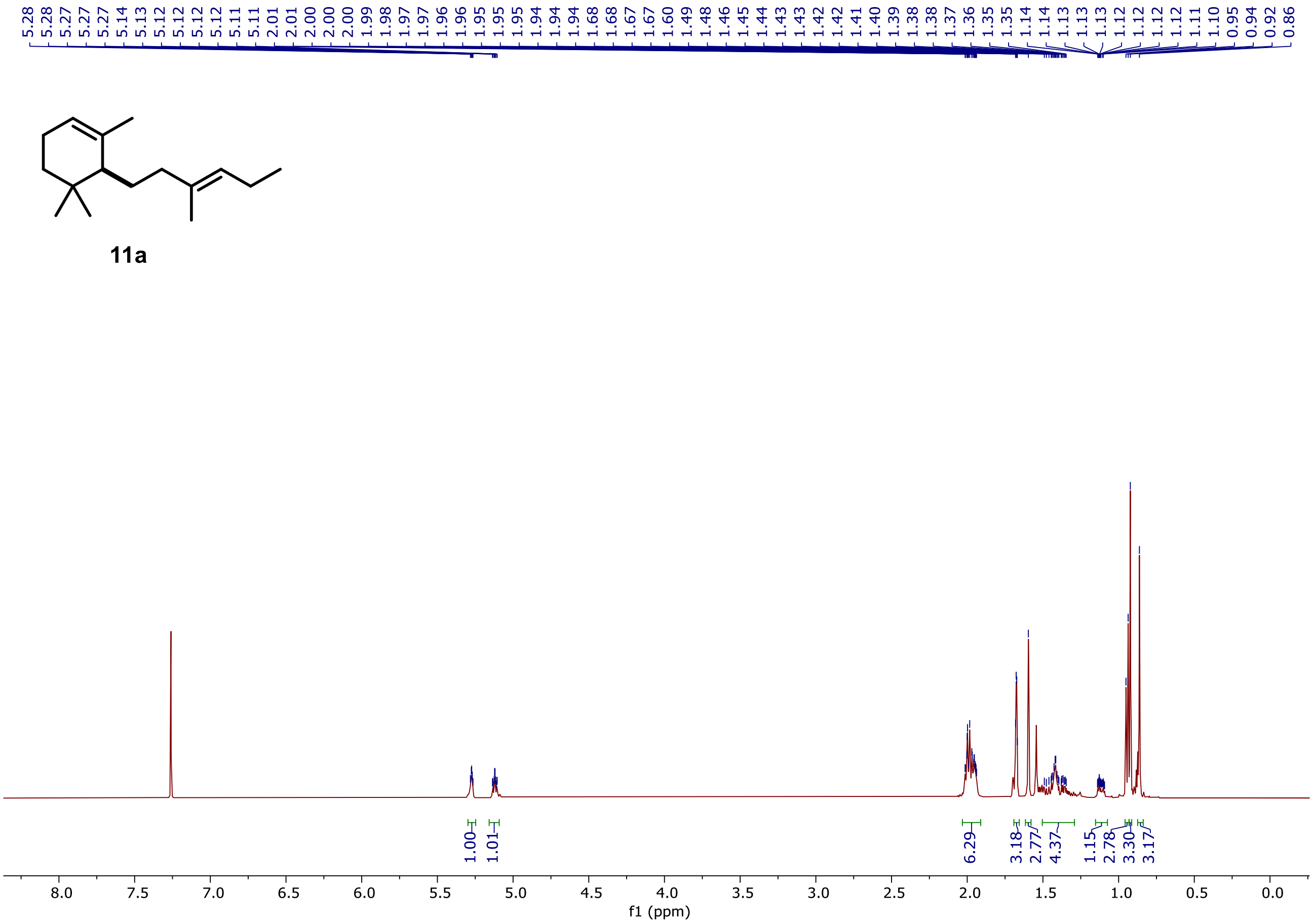

^{13}C NMR spectrum of **11a**

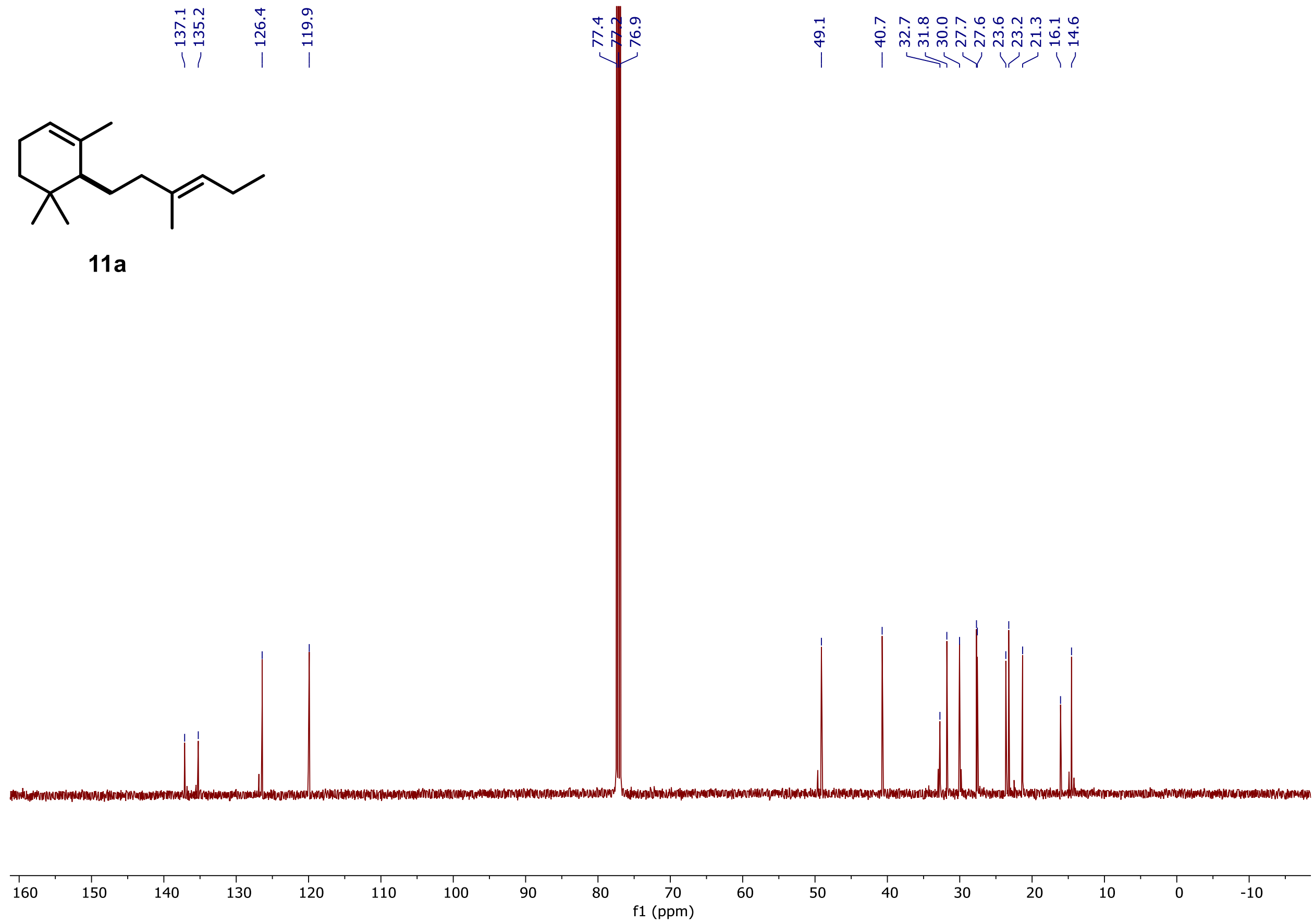

NMR signal assignment of **11b**

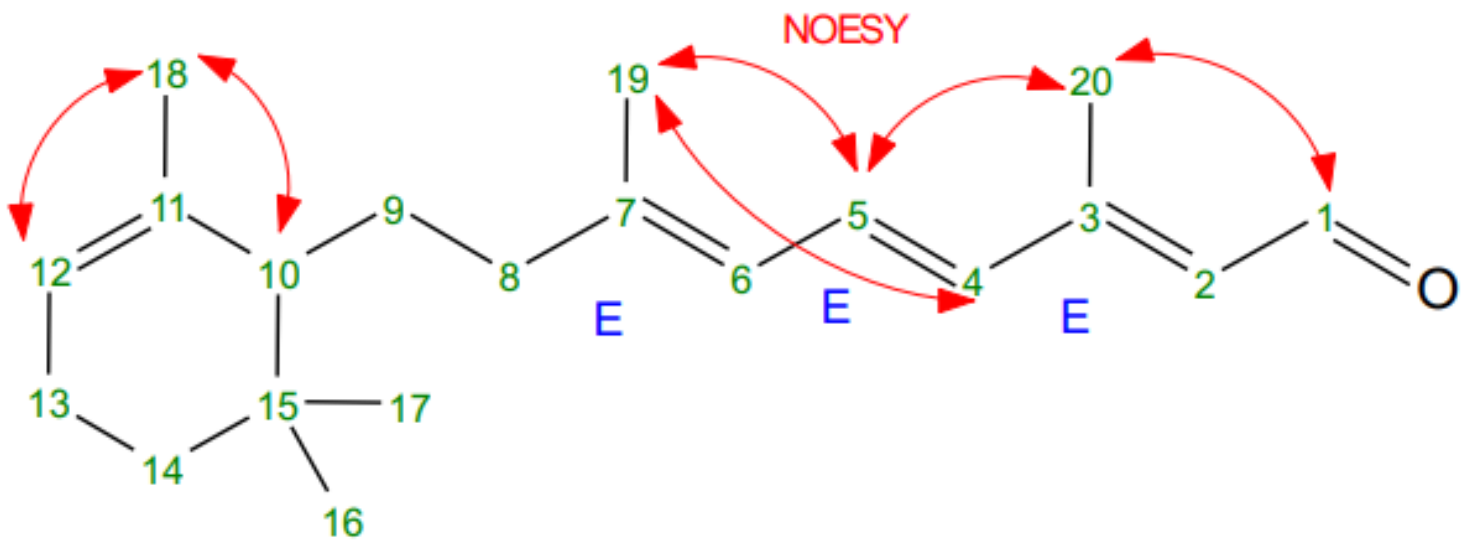

Atom	δ (ppm)	J	COSY	HSQC	HMBC	NOESY
1 C	190.01			1	20	
H	10.00	7.95(2)	2	1	2	2, 20
2 C	129.40			2	1, 4, 20	
H	5.97	7.95(1)	1, 20	2	4	1
3 C	153.64				4, 5, 20	
4 C	133.75			4	2, 6, 19, 20	
H	5.96	15.26(5) trans	5, 6	4	2, 3, 6, 20	6, 19
5 C	131.85			5	6, 19	
H	6.72	15.26(4) trans, 11.00(6)	4, 6	5	3, 6, 7	8, 19, 20
6 C	125.51			6	4, 5, 8, 19	
H	5.88	11.00(5)	4, 5, 19	6	4, 5, 8, 20	4, 8, 9a, 9b
7 C	145.40				5, 8, 19	
8 C	41.20			8	6, 9a, 9b, 10, 19	
H2	2.08		9a, 9b, 10	8	6, 7, 9, 10, 19	5, 6, 10
9 C	29.90			9a, 9b	8, 10, 19	
Ha	1.55		8, 9b	9	8, 10, 11, 15	6
Hb	1.45		8, 9a	9	8, 10, 11, 15	6
10 C	49.32			10	8, 9a, 9b, 14a, 14b, 16, 17, 18	
H	1.42		8, 14b, 18	10	8, 9, 11	8, 16, 17, 18

Atom	δ (ppm)	J	COSY	HSQC	HMBC	NOESY
11 C	136.31				9a, 9b, 10, 18	
12 C	120.93			12	14a, 14b, 18	
H	5.35		13, 18	12		13, 18
13 C	23.44			13	14a, 14b	
H2	1.96		12, 14b, 18	13		12, 14a, 14b, 16, 17
14 C	31.92			14a, 14b	16, 17	
Ha	1.43		14b	14	10, 12, 13, 15	12, 13, 14b, 16, 17
Hb	1.08		10, 13, 14a	14	10, 12, 13, 15	13, 14a, 16, 17
15 C	32.78				9a, 9b, 14a, 14b, 16	
16 C	27.12, 27.69			16	17	
H3	0.92, 1.07			16	10, 14, 15	10, 13, 14a, 14b
17 C	27.12, 27.69			17		
H3	0.92, 1.07			17	10, 14, 16	10, 13, 14a, 14b
18 C	23.70			18		
H3	1.70		10, 12, 13	18	10, 11, 12	10, 12
19 C	17.10			19	8	
H3	1.60	1.27(?)	6	19	4, 5, 6, 7, 8, 9	4, 5
20 C	12.54			20	4, 6	
H3	1.74	1.13(?)	2	20	1, 2, 3, 4	1, 5

^{1}H NMR spectrum of **11b**

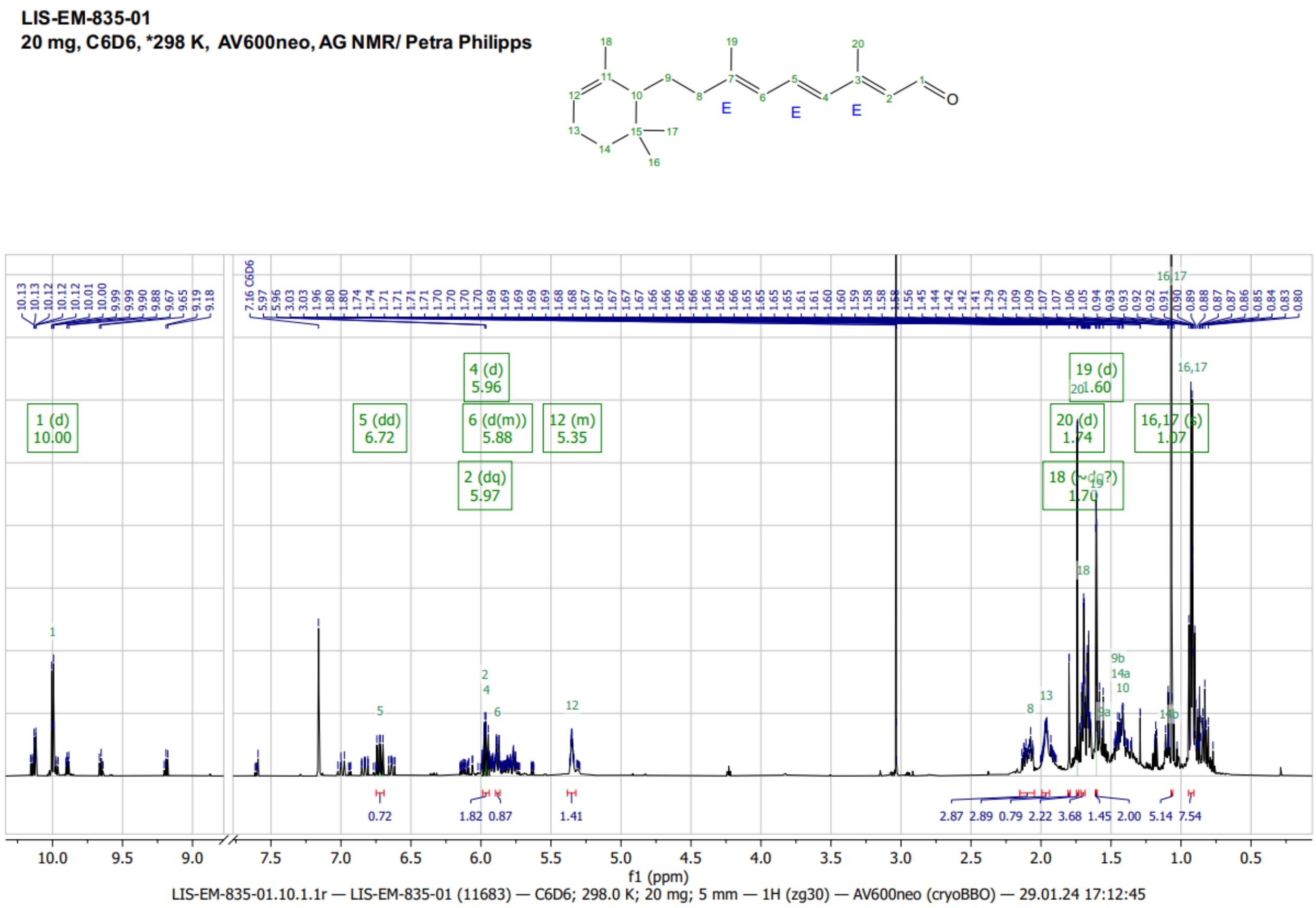

^{13}C NMR spectrum of **11b**

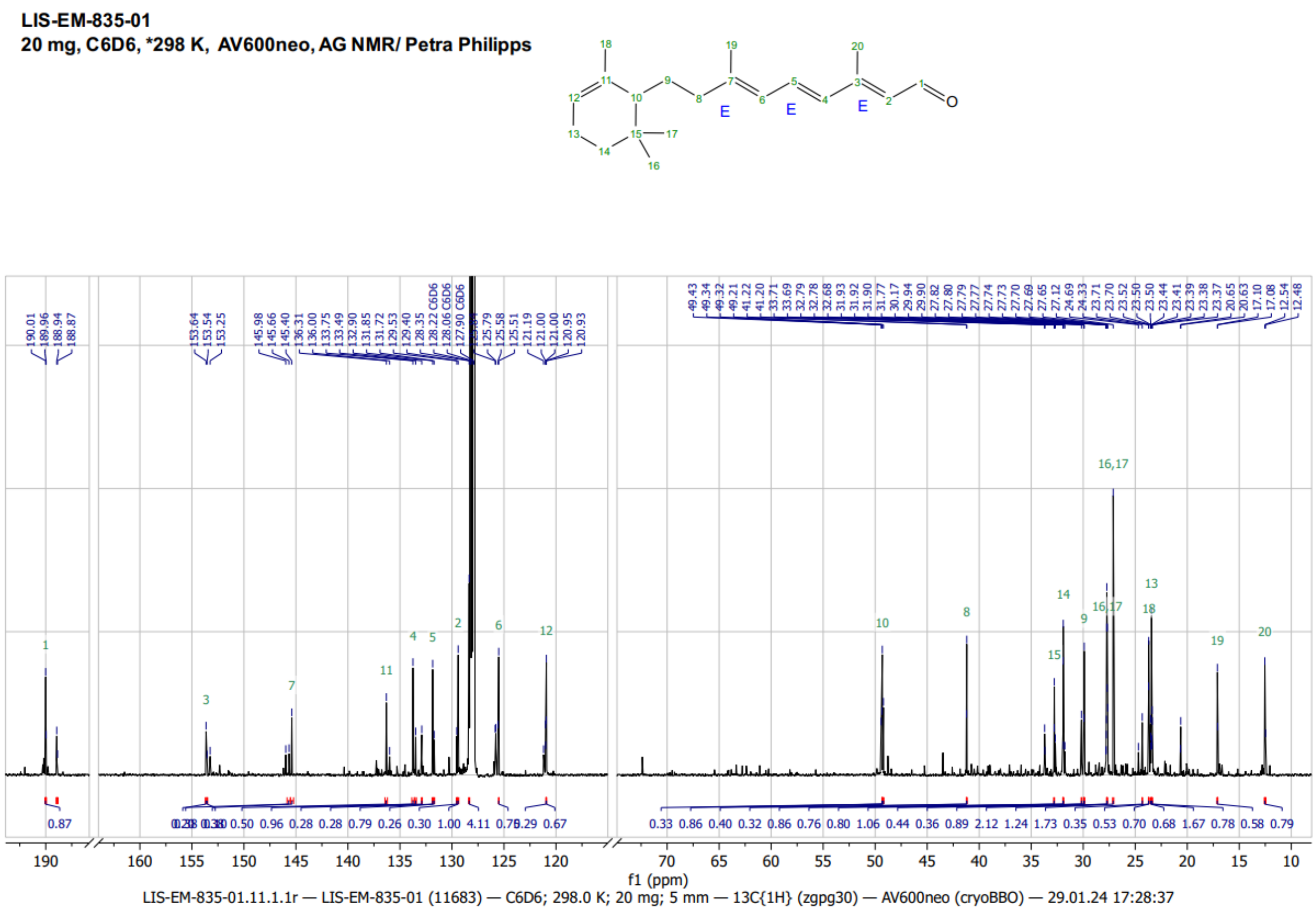

$^{1}H\{^{13}C\}$ HSQC NMR spectrum of **11b**

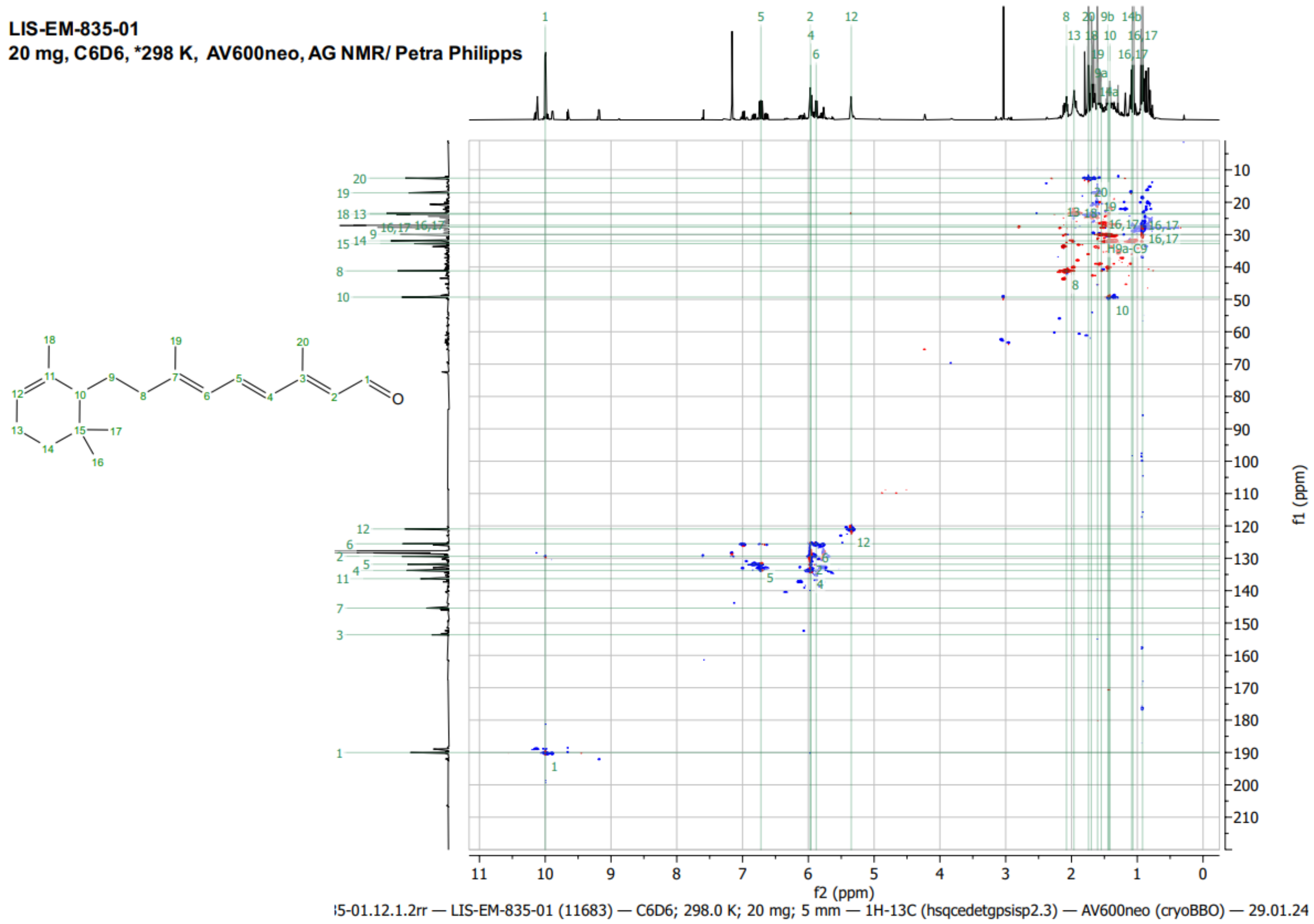

$^{1}H\{^{13}C\}$ HMBC NMR spectrum of **11b**

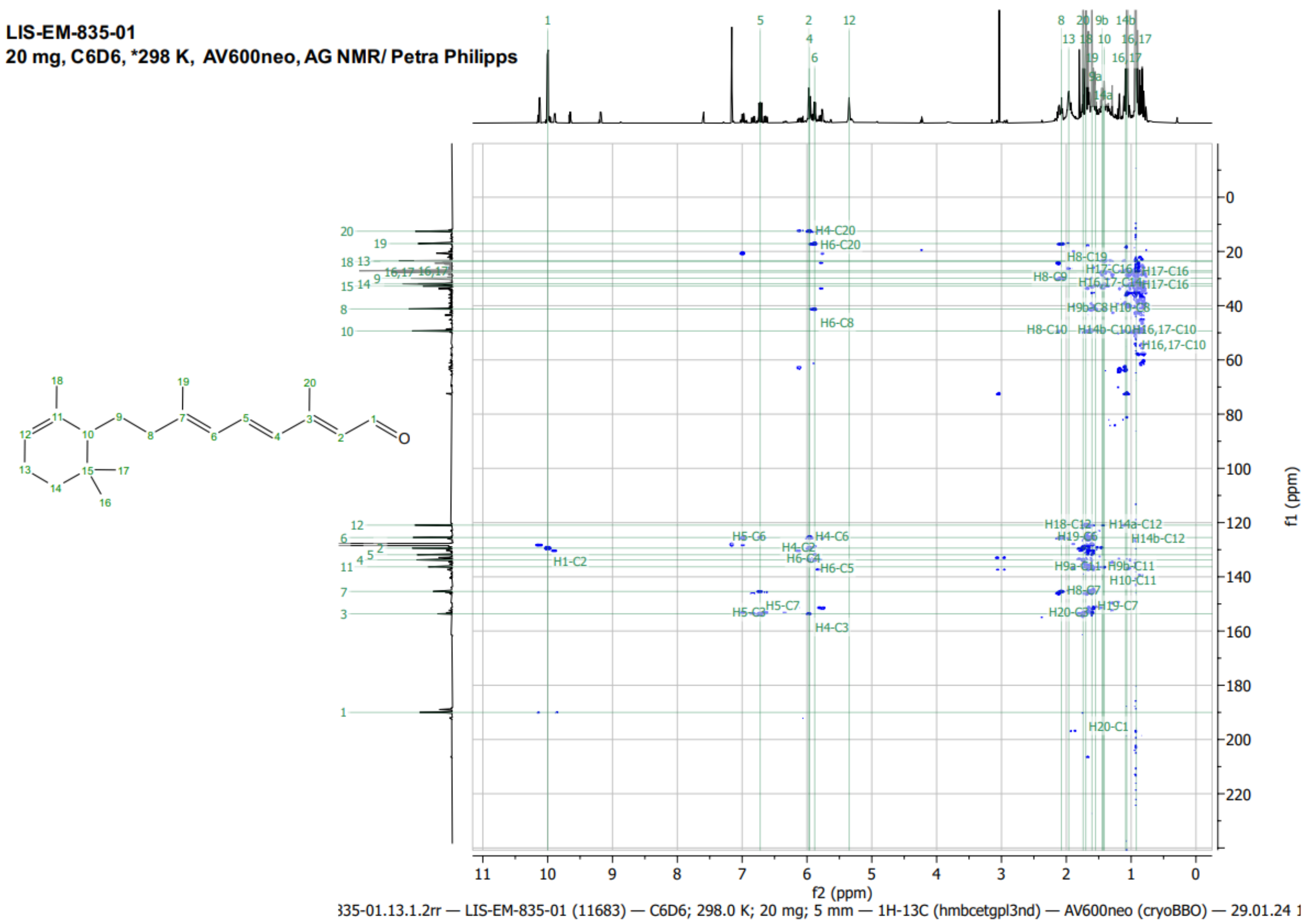

^{1}H{off} COSY NMR spectrum of **11b**

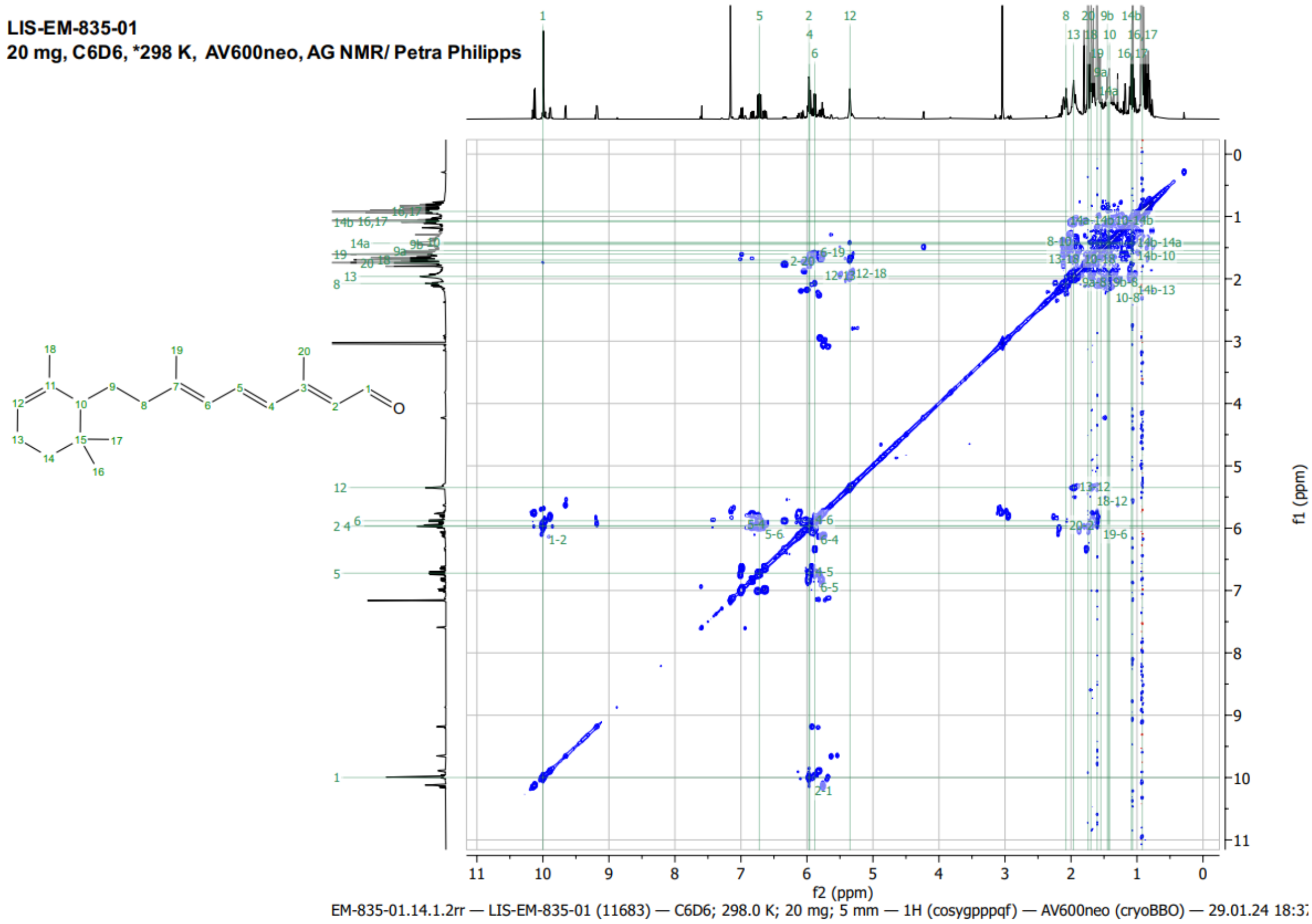

^{1}H{off} NOESY NMR spectrum of **11b**

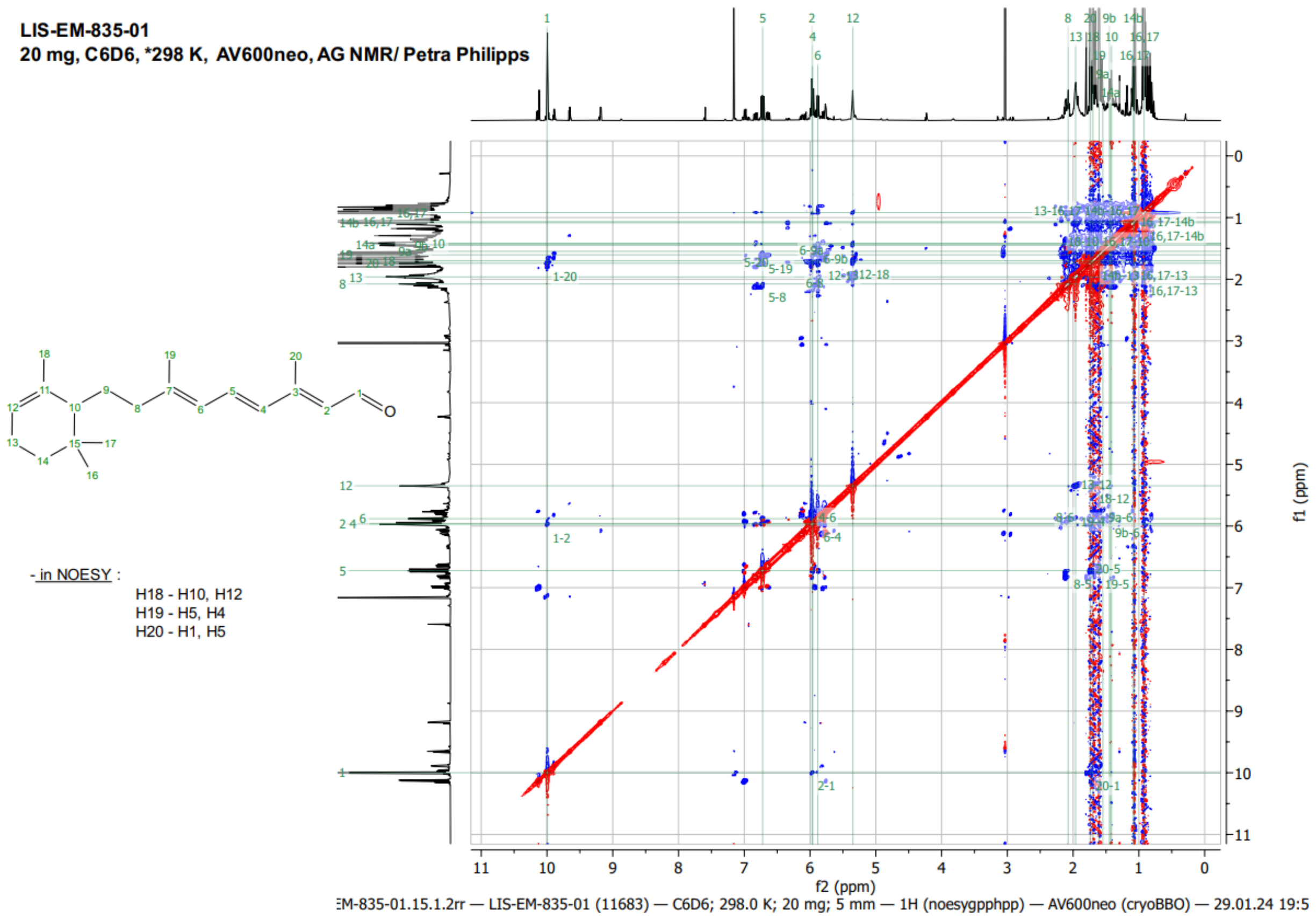

^1H NMR spectrum of **11b** (**11b**:**12b**= 54:46 in $CDCl_3$)

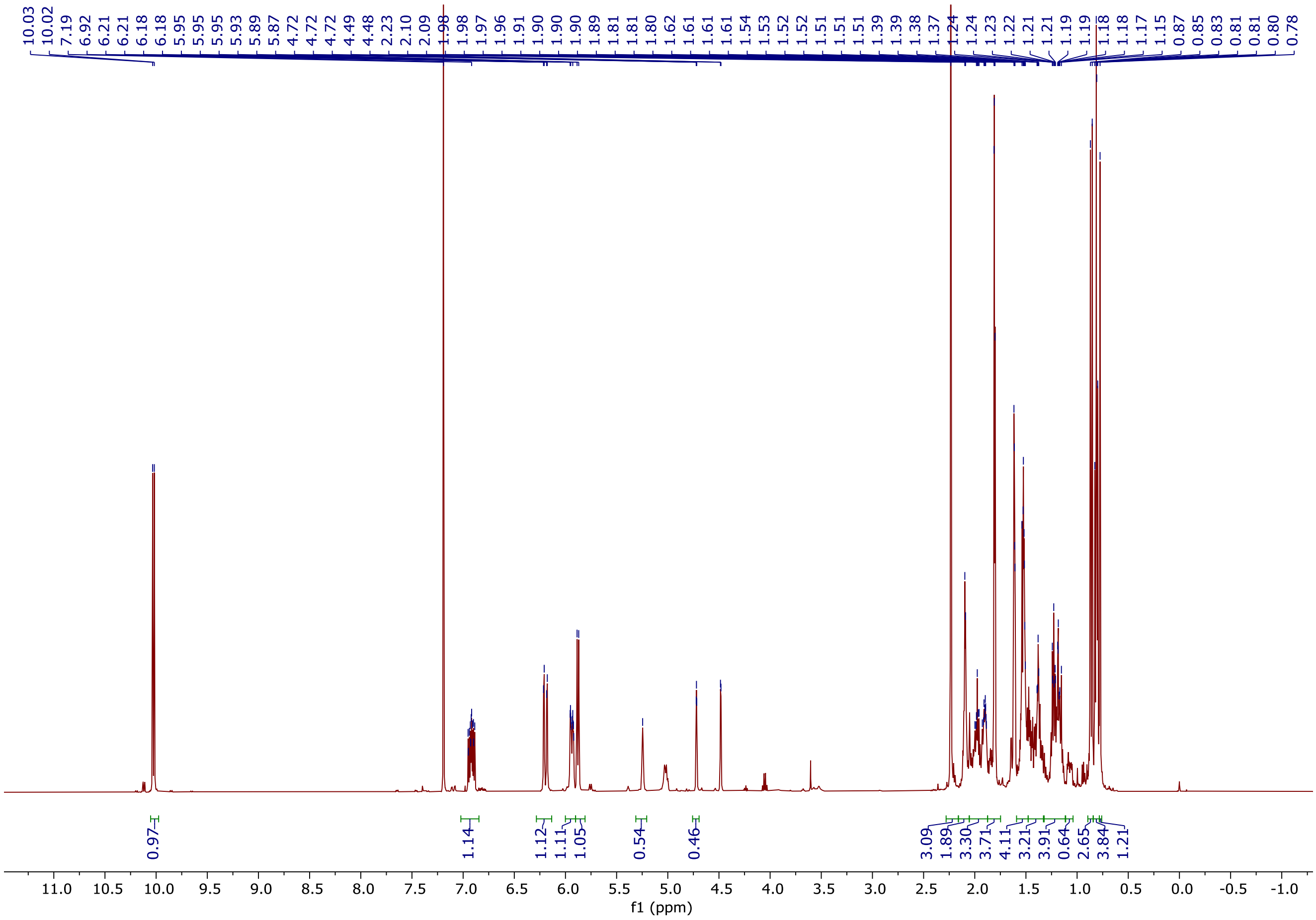

^1H NMR spectrum of **11b** (only **11b** in $CDCl_3$)

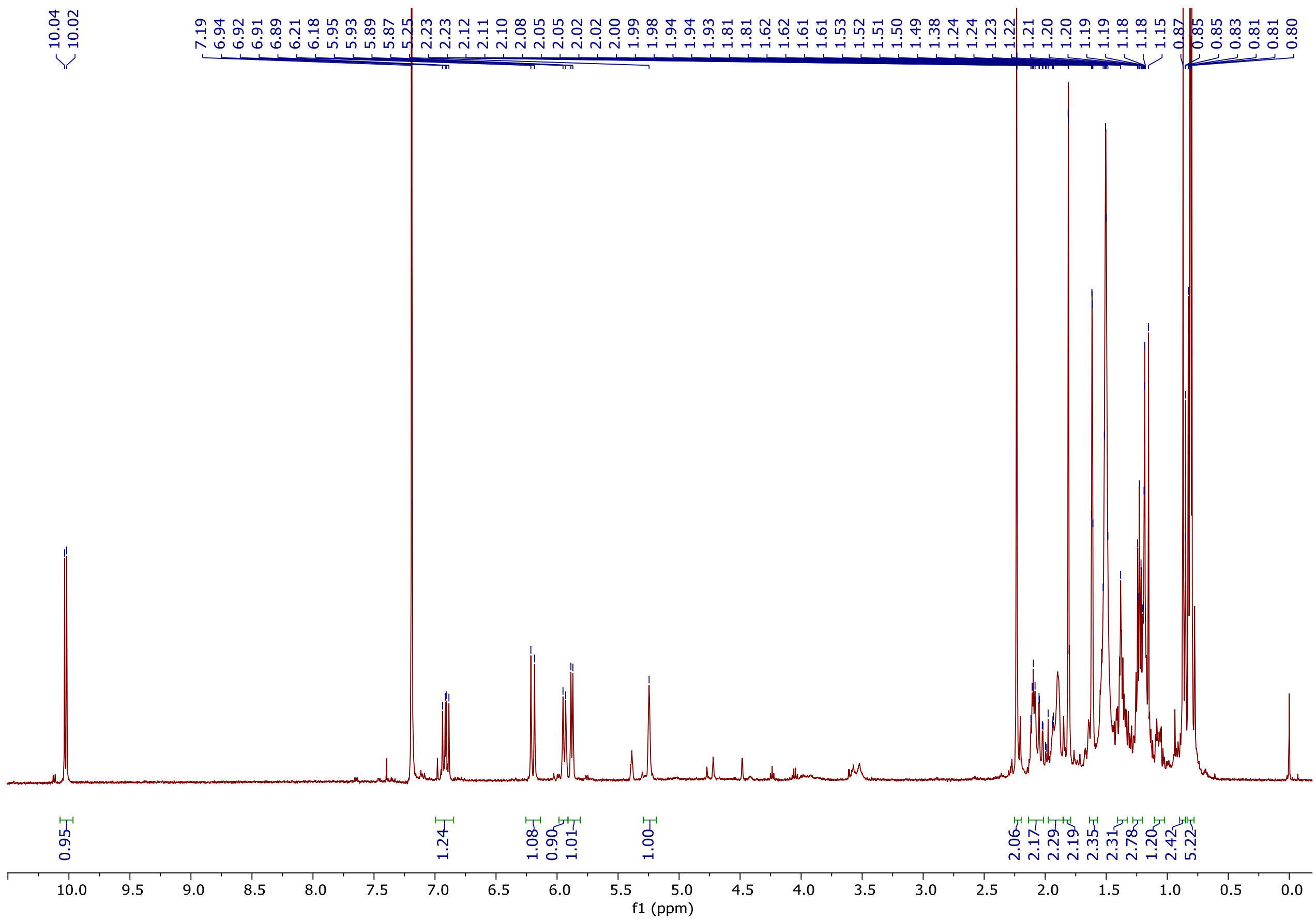

NMR signal assignment of **11c**

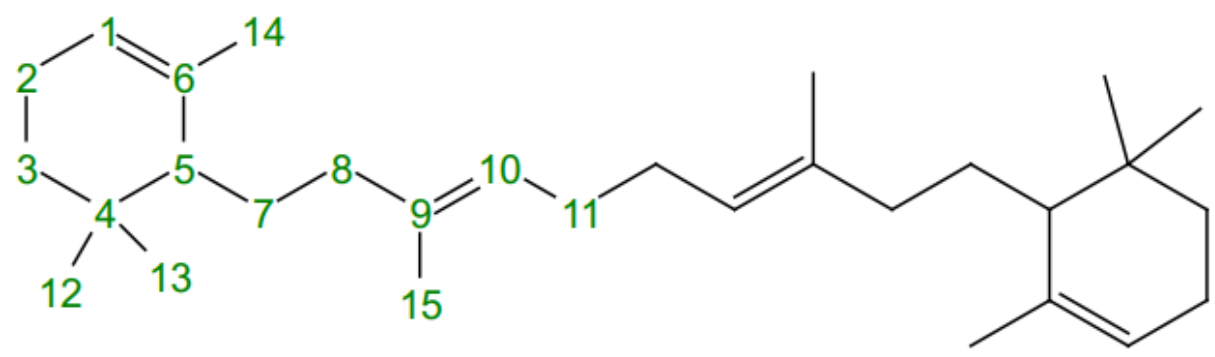

Atom	δ (ppm)	HSQC	COSY	HMBC	NOESY
1 C	119.928	1		3', 3", 14	
H	5.272	1	2, 14	2, 3, 5, 14	2, 14
2 C	23.223	2		1, 3', 3"	
H2	1.950	2	1, 3', 3", 14		1, 3', 3", 13
3 C	31.798	3', 3"		1, 5, 12, 13	
H'	1.112	3	2, 3"	1, 2, 4, 5, 12, 13	2, 3", 12, 13
H"	1.425	3	2, 3'	1, 2, 4, 5, 12, 13	2, 3'
4 C	32.737			3', 3", 7', 7", 12, 13	
5 C	49.131	5		1, 3', 3", 7', 7", 8, 12, 13, 14	
H	1.413	5	7', 7", 14	3, 6, 7, 8	8, 12, 13, 14
6 C	137.081			5, 7', 7", 14	
7 C	30.045	7', 7"		5, 8	
H'	1.501	7	5, 8	4, 5, 6, 8, 9	7", 12
H"	1.361	7	5, 8	4, 5, 6, 8, 9	7', 12, 14
8 C	40.794	8		5, 7', 7", 10, 15	
H2	1.997	8	7', 7"	5, 7, 9, 10, 15	5, 12
9 C	136.055			7', 7", 8, 11, 15	
10 C	124.288	10		8, 11, 15	
H	5.133	10	11, 15	8, 11, 15	11
11 C	28.394	11		10, 11	
H2	2.008	11	10, 15	9, 10, 11	10, 15
12 C	27.574	12		3', 3"	
H3	0.919	12	13	3, 4, 5	3', 5, 7', 7", 8
13 C	27.701	13		3', 3"	
H3	0.861	13	12	3, 4, 5	2, 3', 5
14 C	23.627	14		1	
H3	1.671	14	1, 2, 5	1, 5, 6	1, 5, 7"
15 C	16.271	15		8, 10	
H3	1.599	15	10, 11	8, 9, 10	11

^{1}H NMR spectrum of **11c**

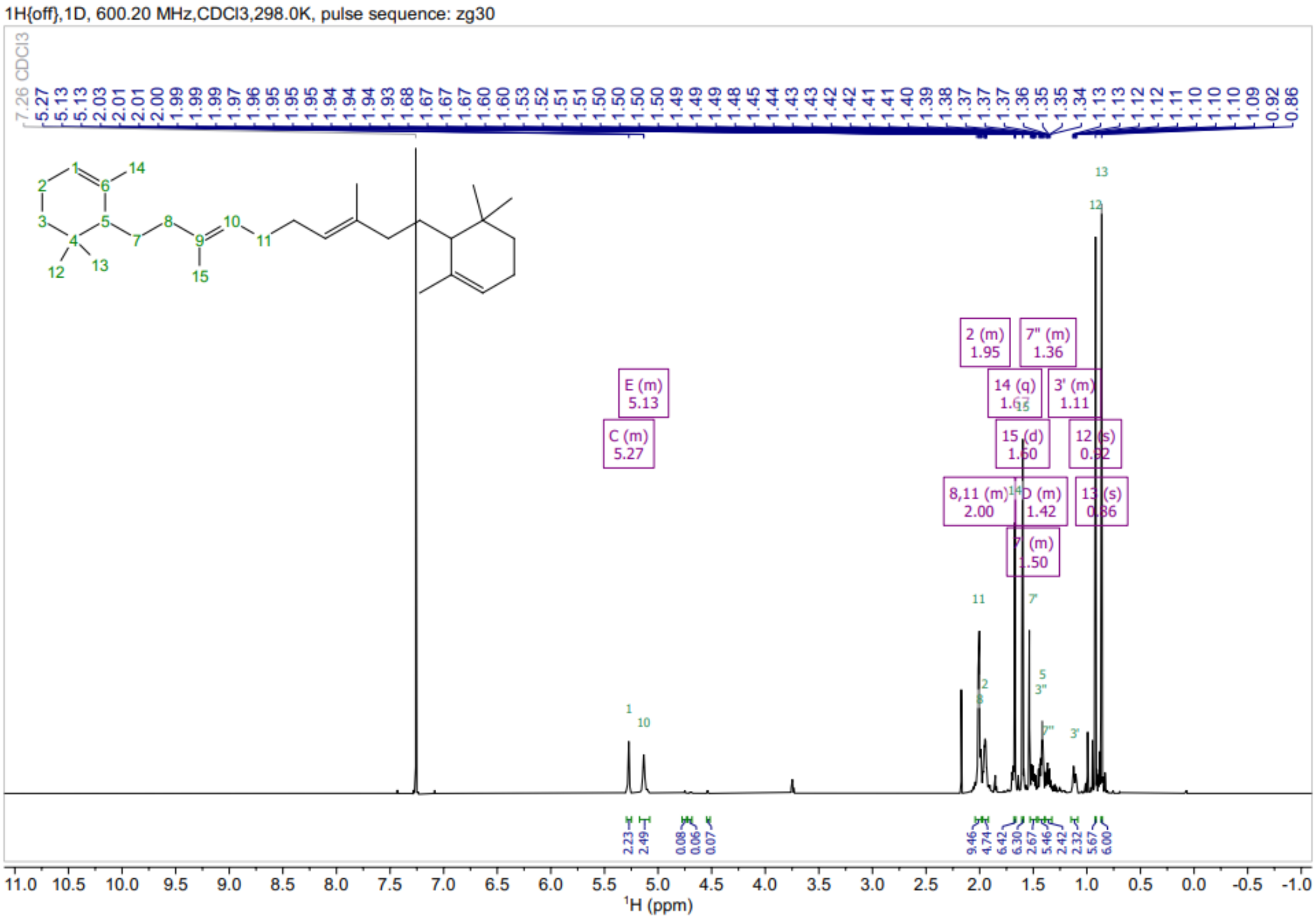

^{13}C NMR spectrum of **11c**

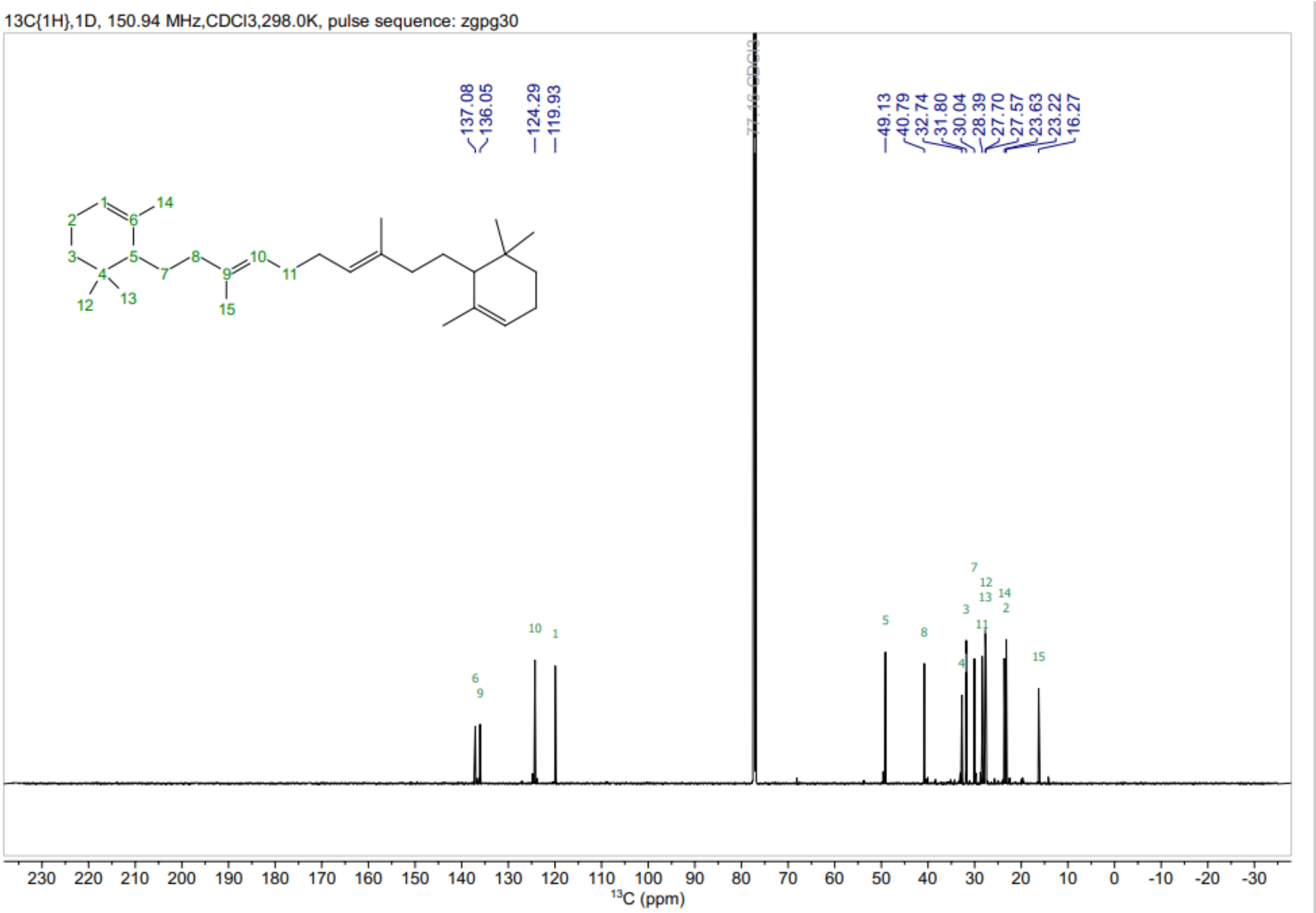

$^{1}H\{^{13}C\}$ HSQC NMR spectrum of **11c**

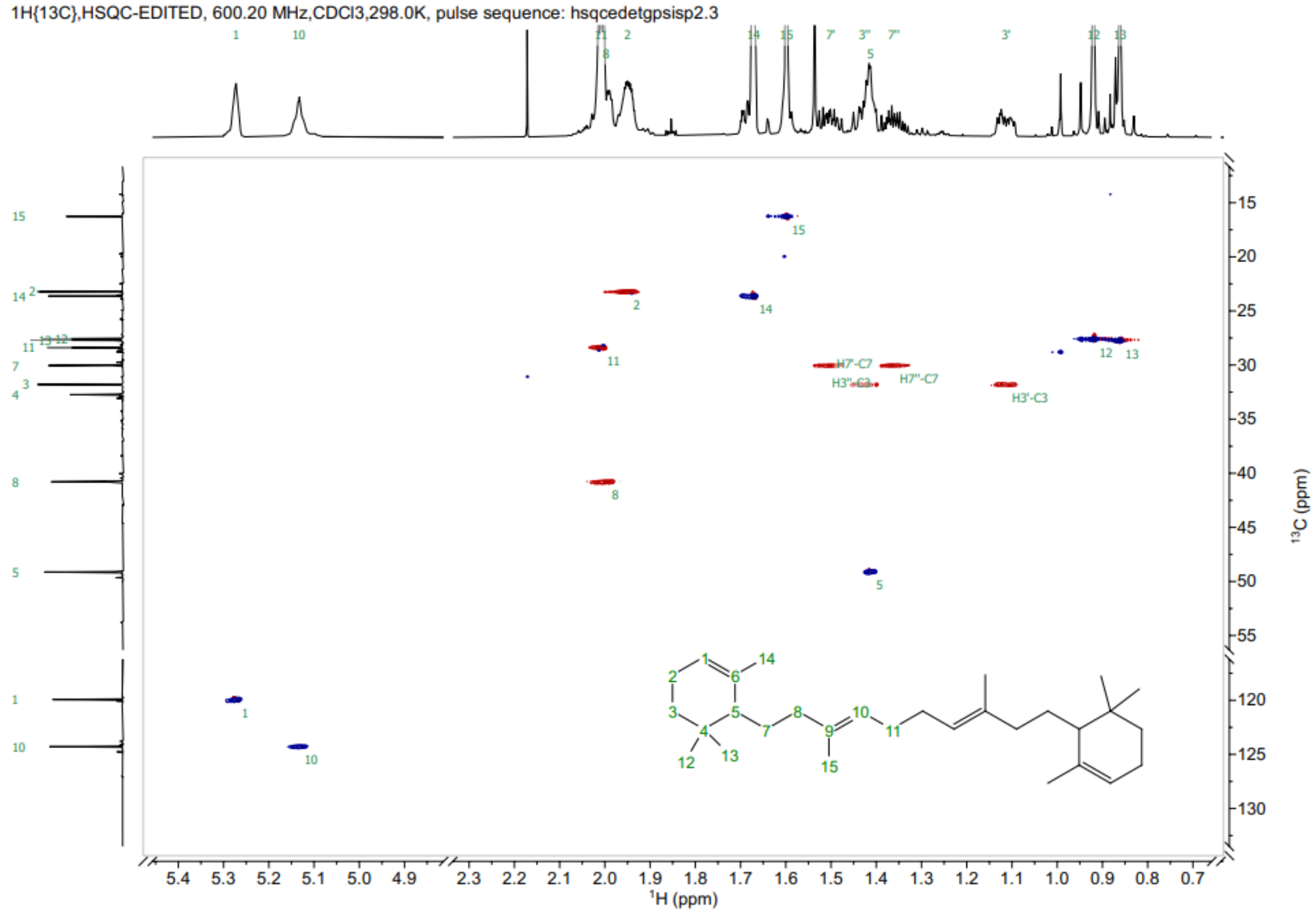

$^{1}H\{^{13}C\}$ HMBC NMR spectrum of **11c**

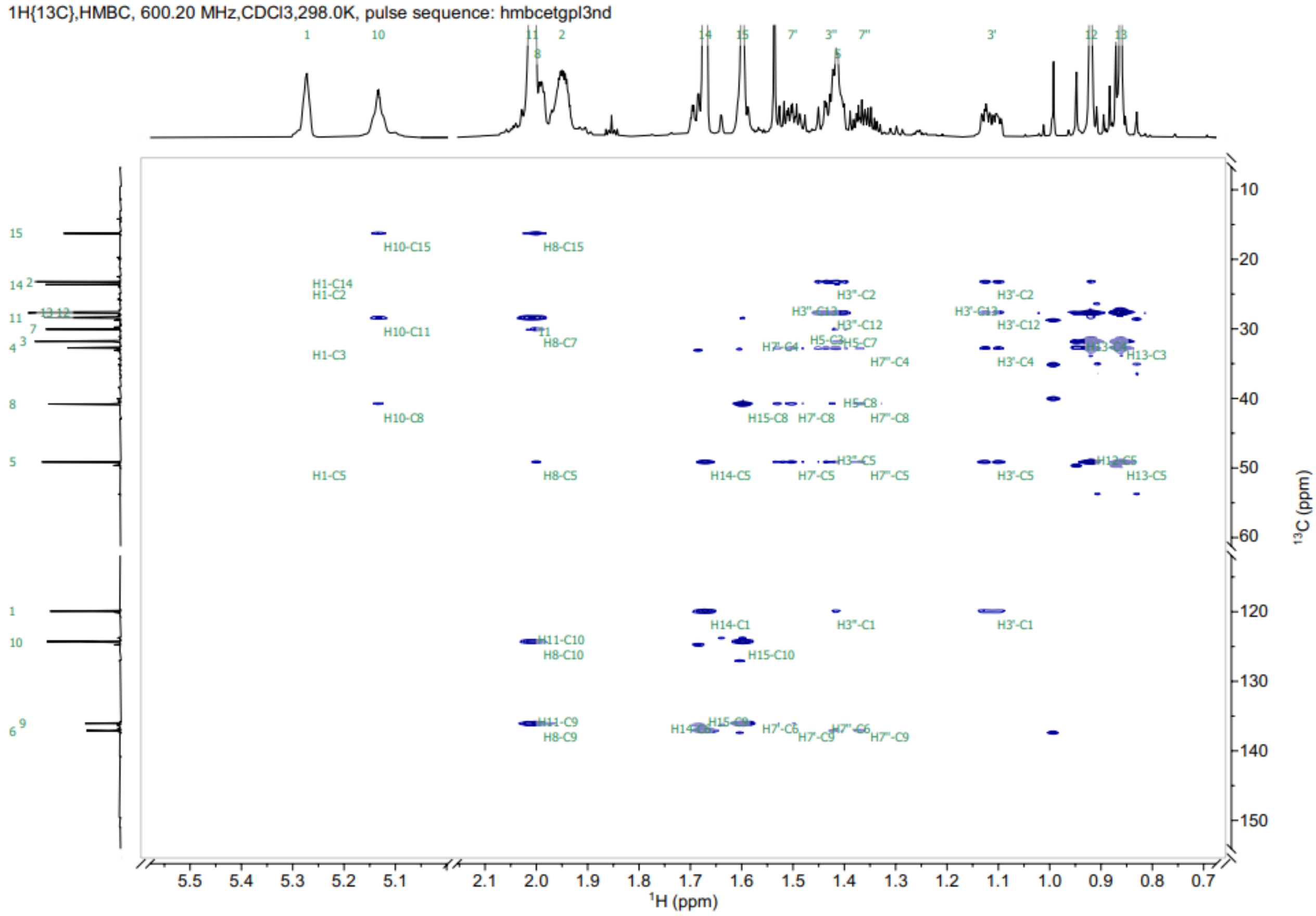

^{1}H{off} COSY NMR spectrum of **11c**

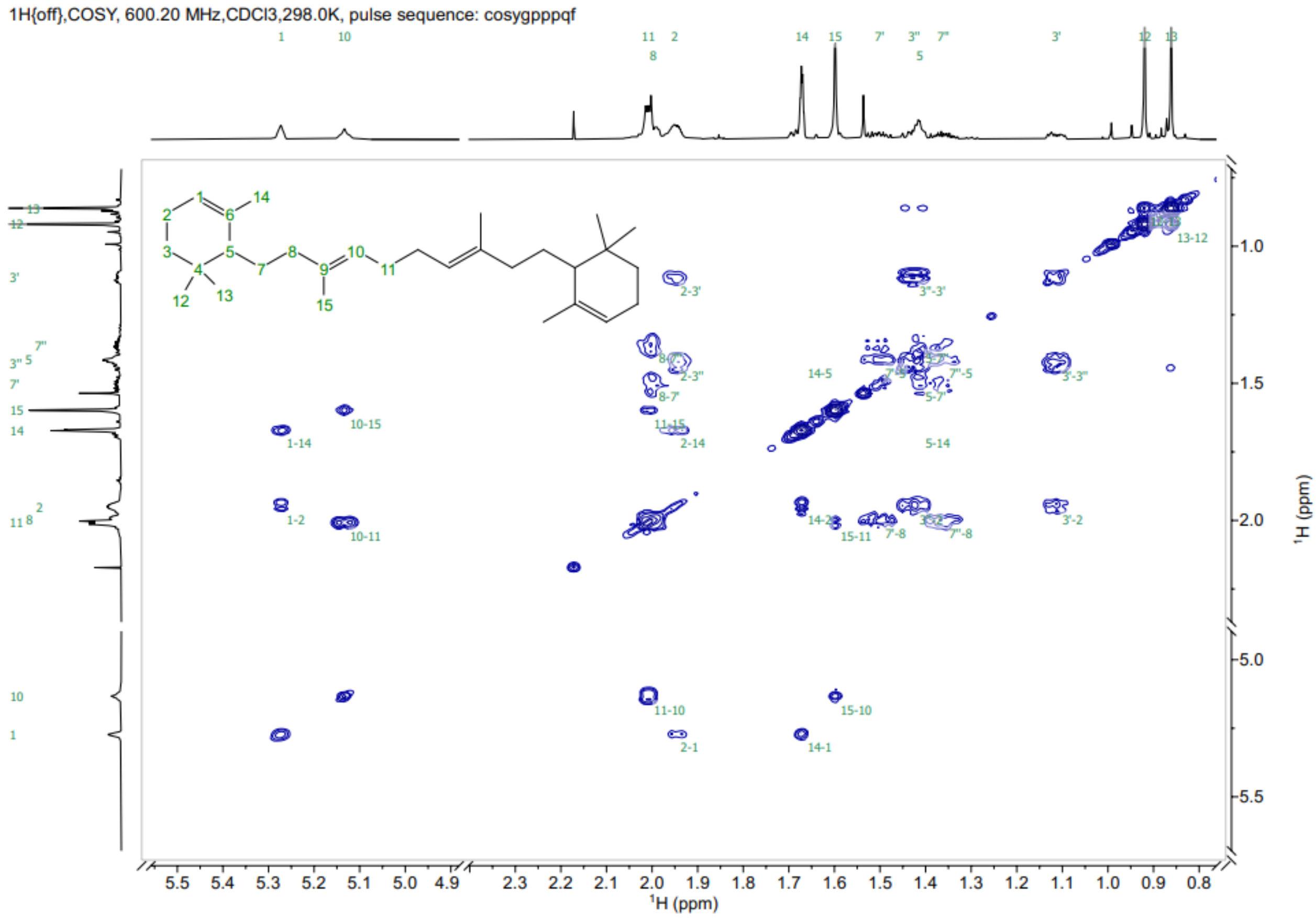

^{1}H{off} NOESY NMR spectrum of **11c**

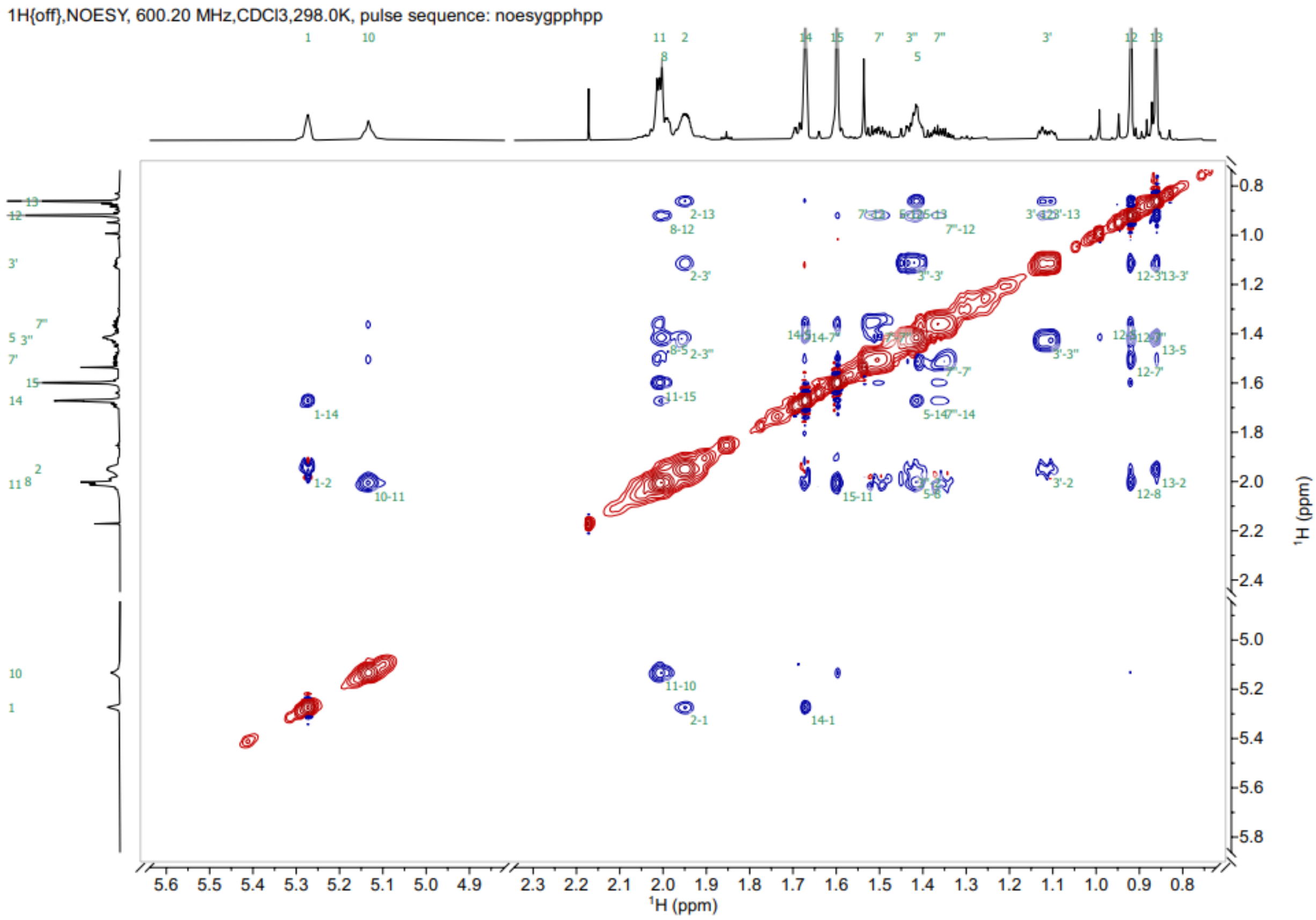

^{1}H NMR spectrum of **13a**

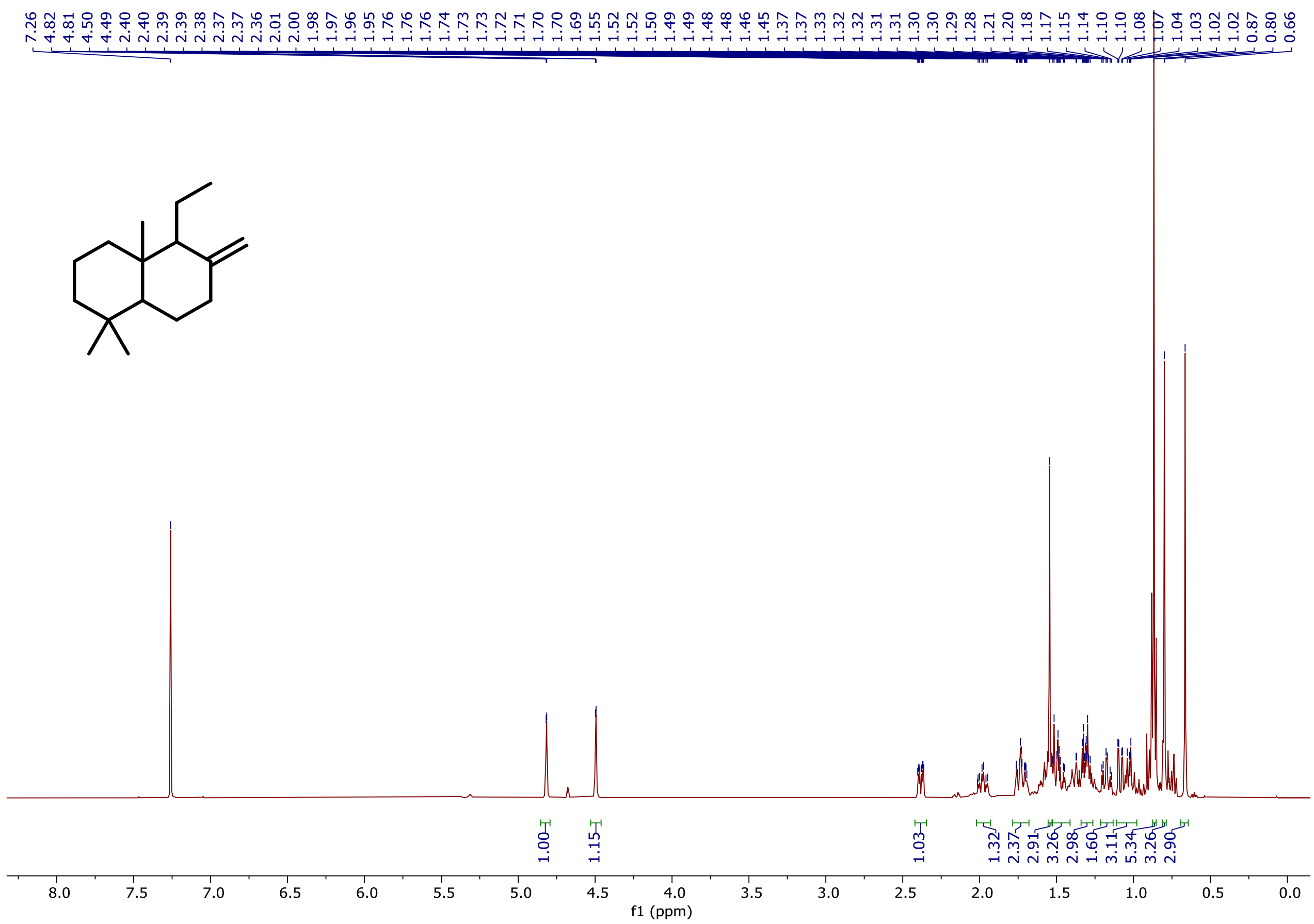

^{13}C NMR spectrum of **13a**

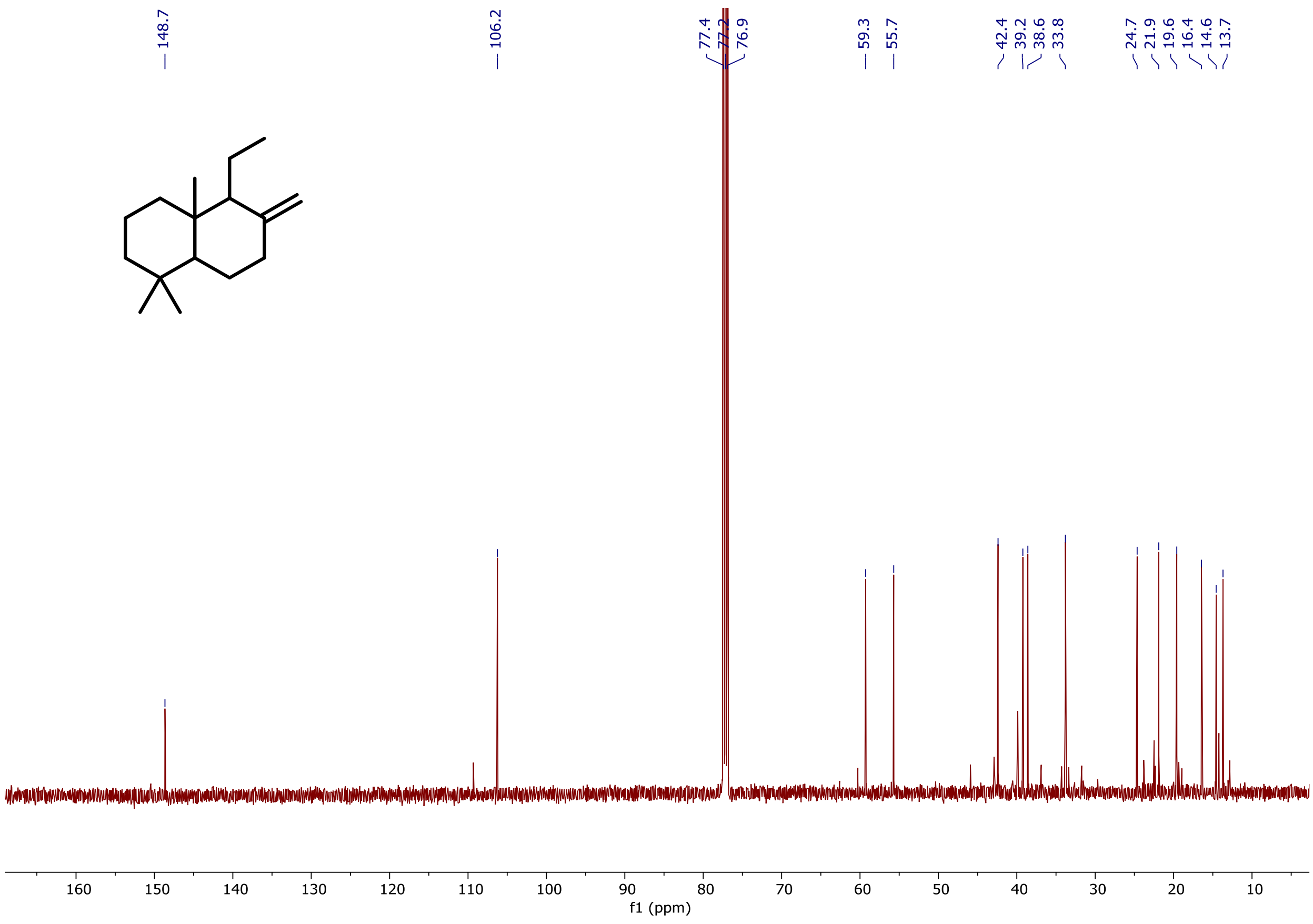

^{1}H NMR spectrum of **S8**

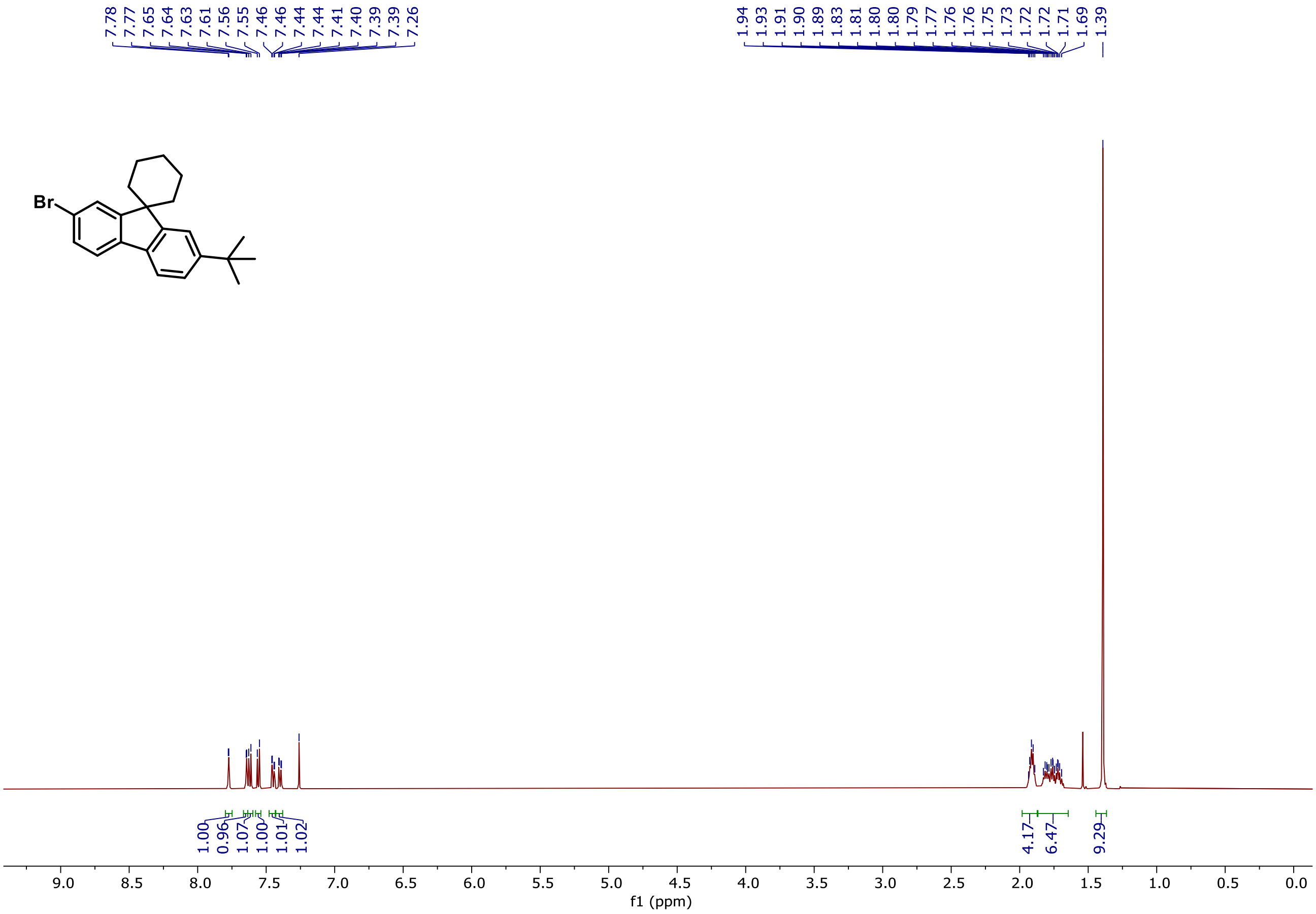

^{13}C NMR spectrum of **S8**

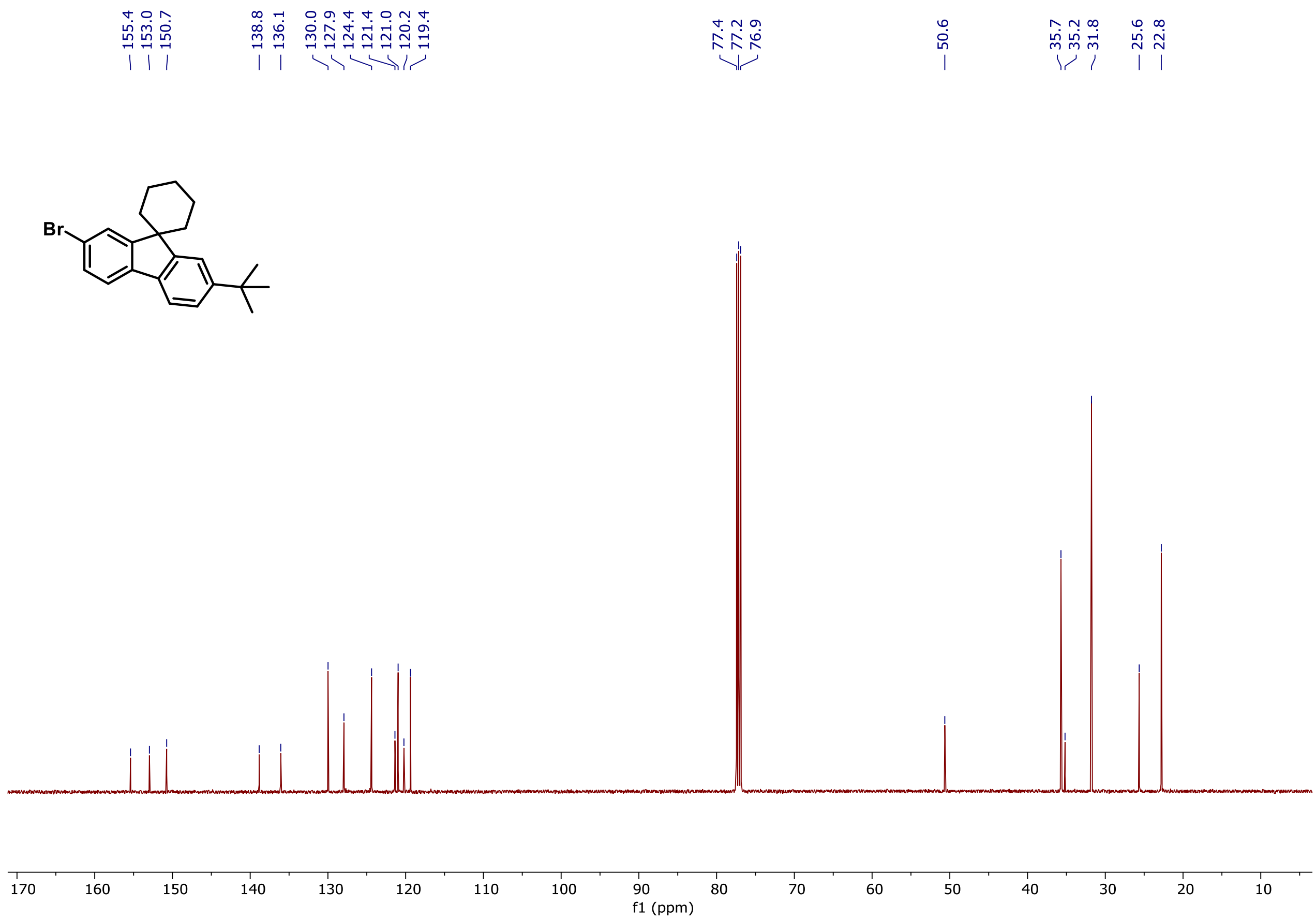

^{1}H NMR spectrum of **S9**

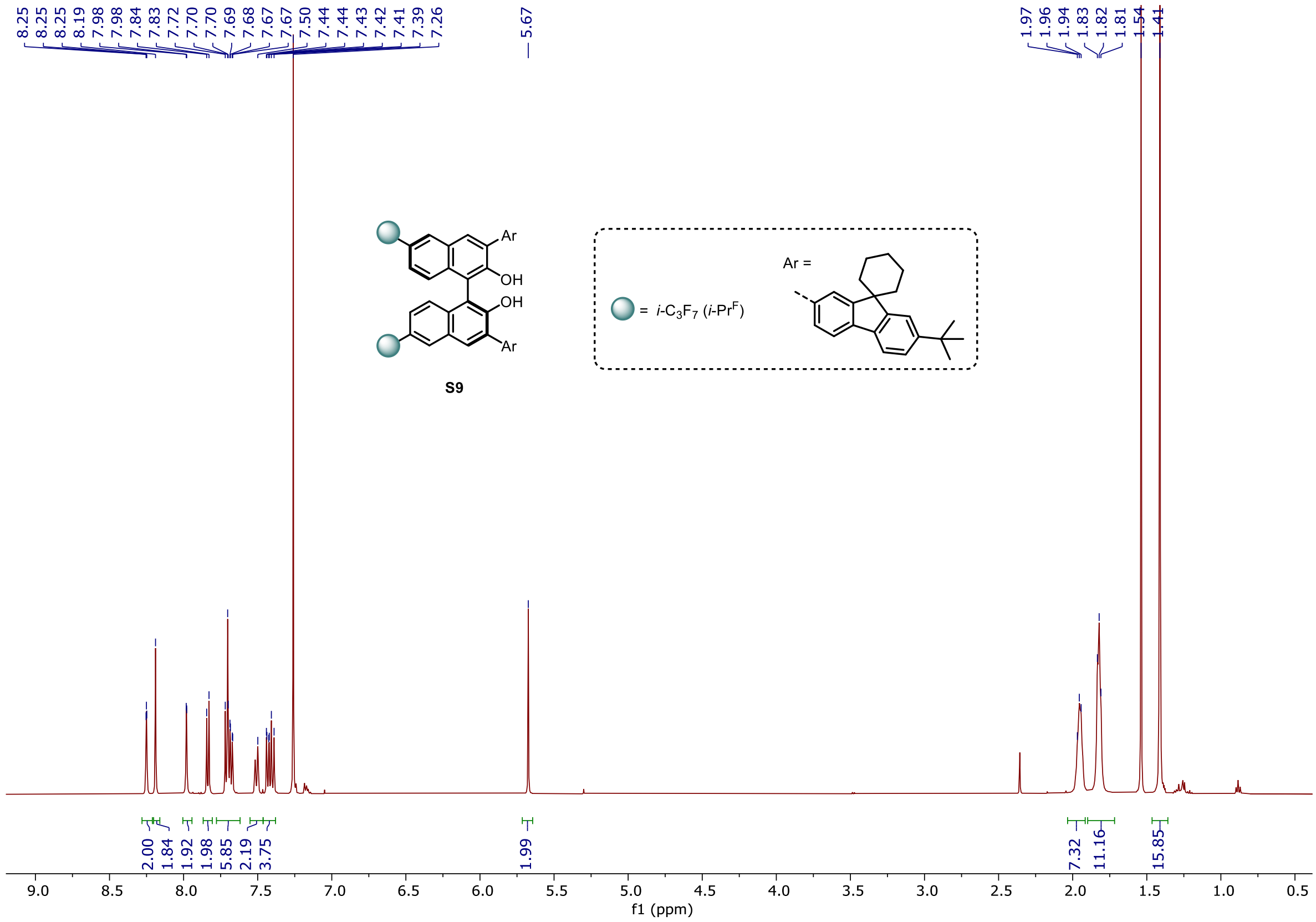

^{13}C NMR spectrum of **S9**

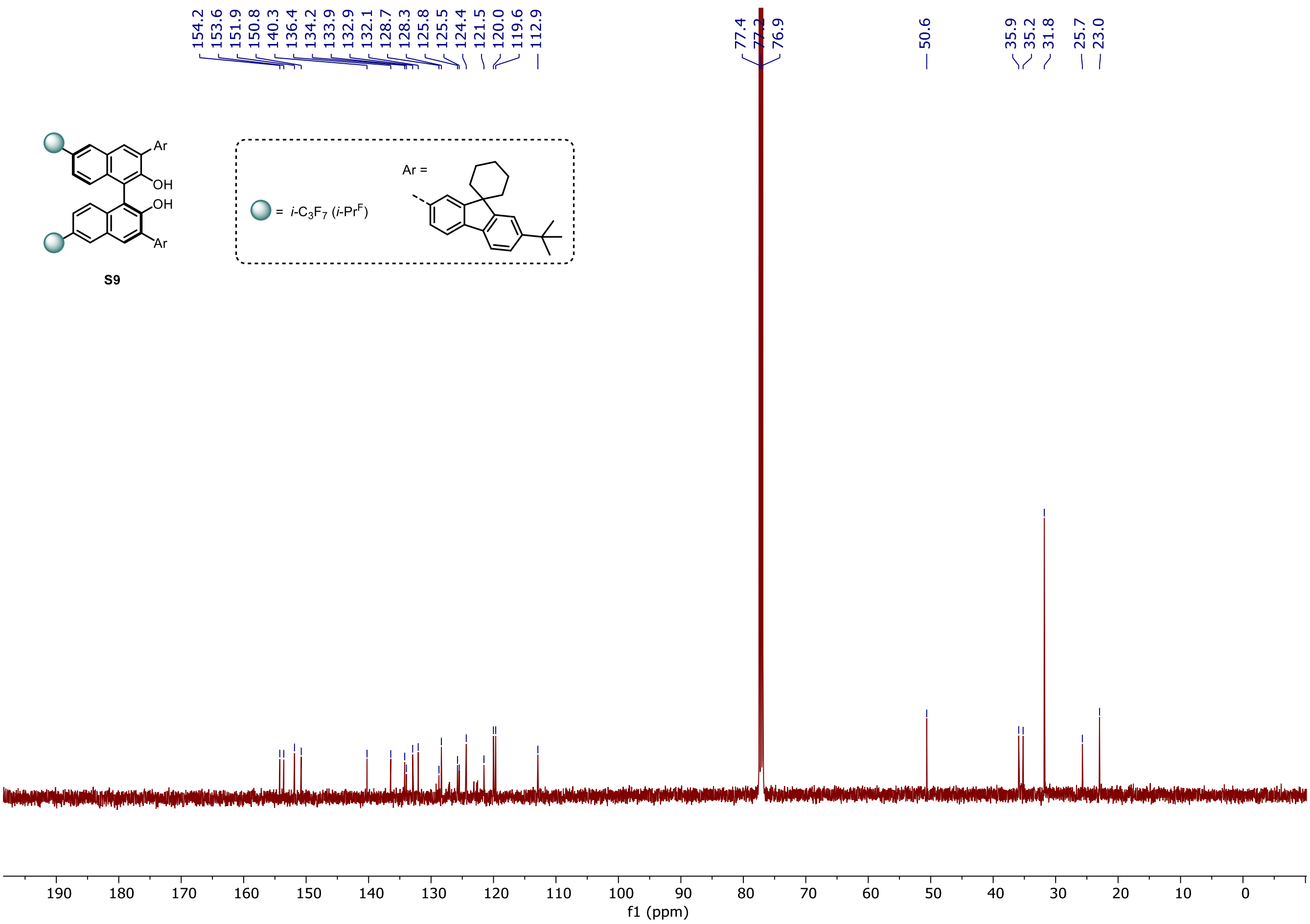

^{19}F NMR spectrum of **S9**

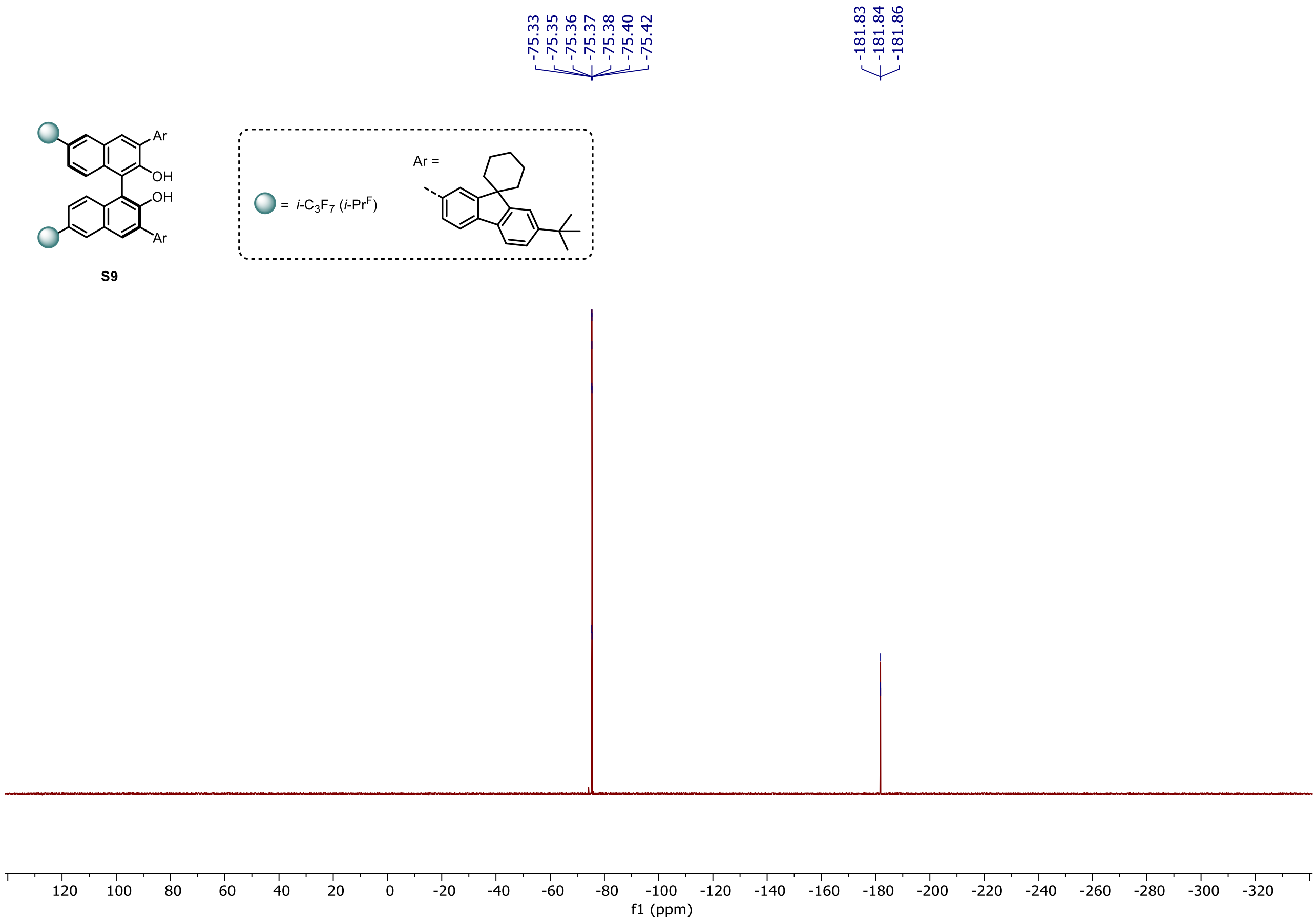

^{1}H NMR spectrum of **S13**

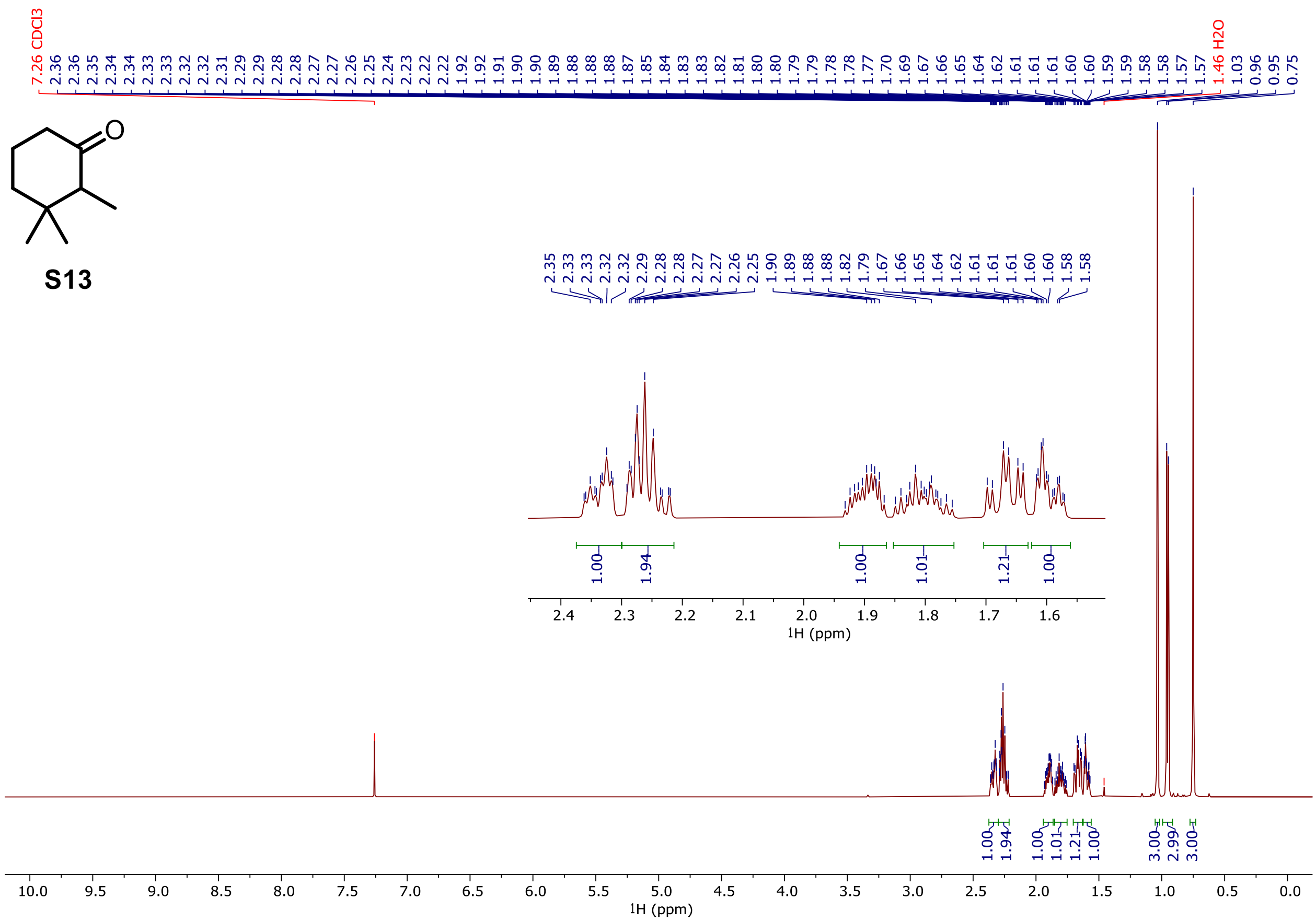

^{13}C NMR spectrum of **S13**

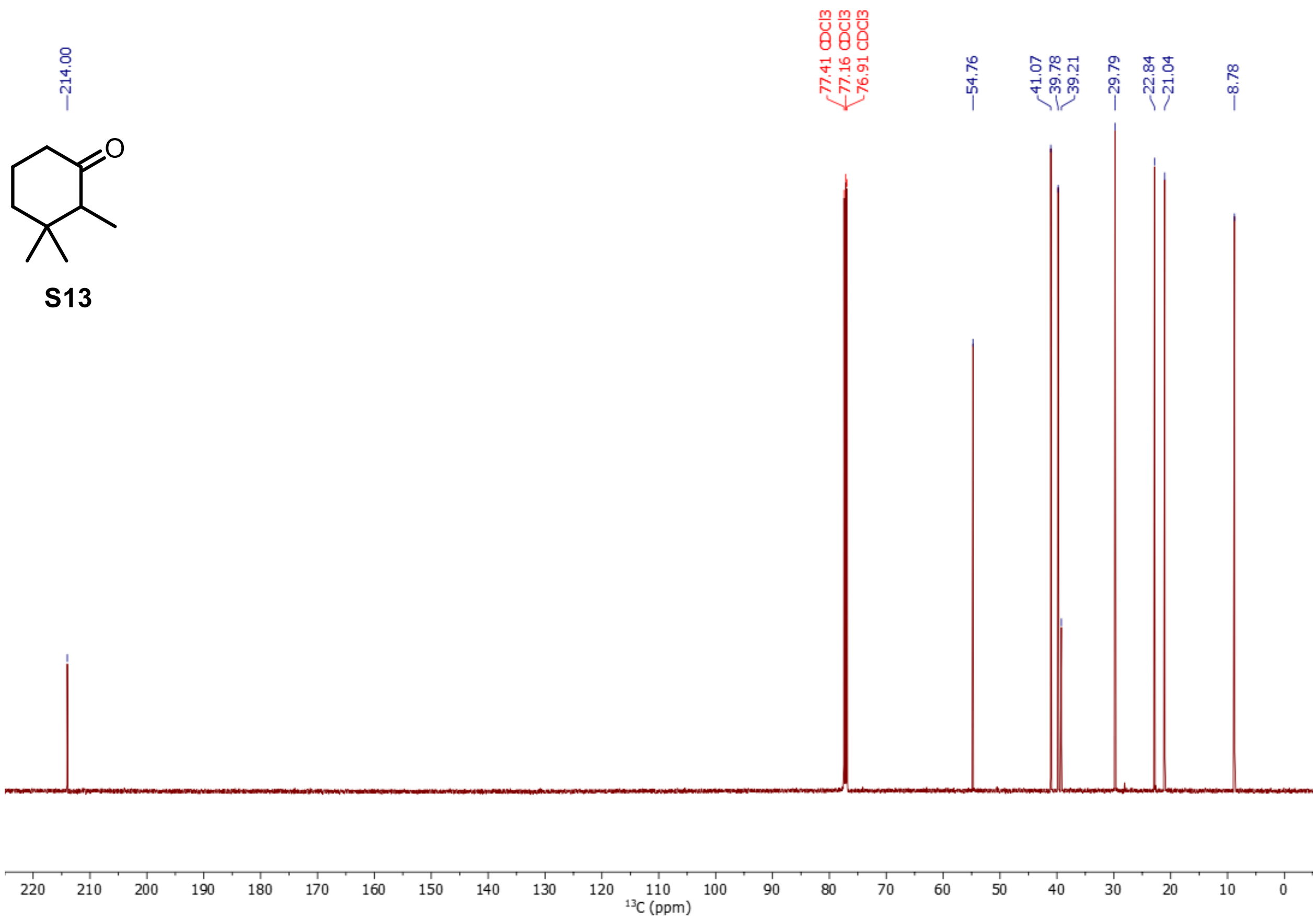

^{1}H NMR spectrum of **S19**

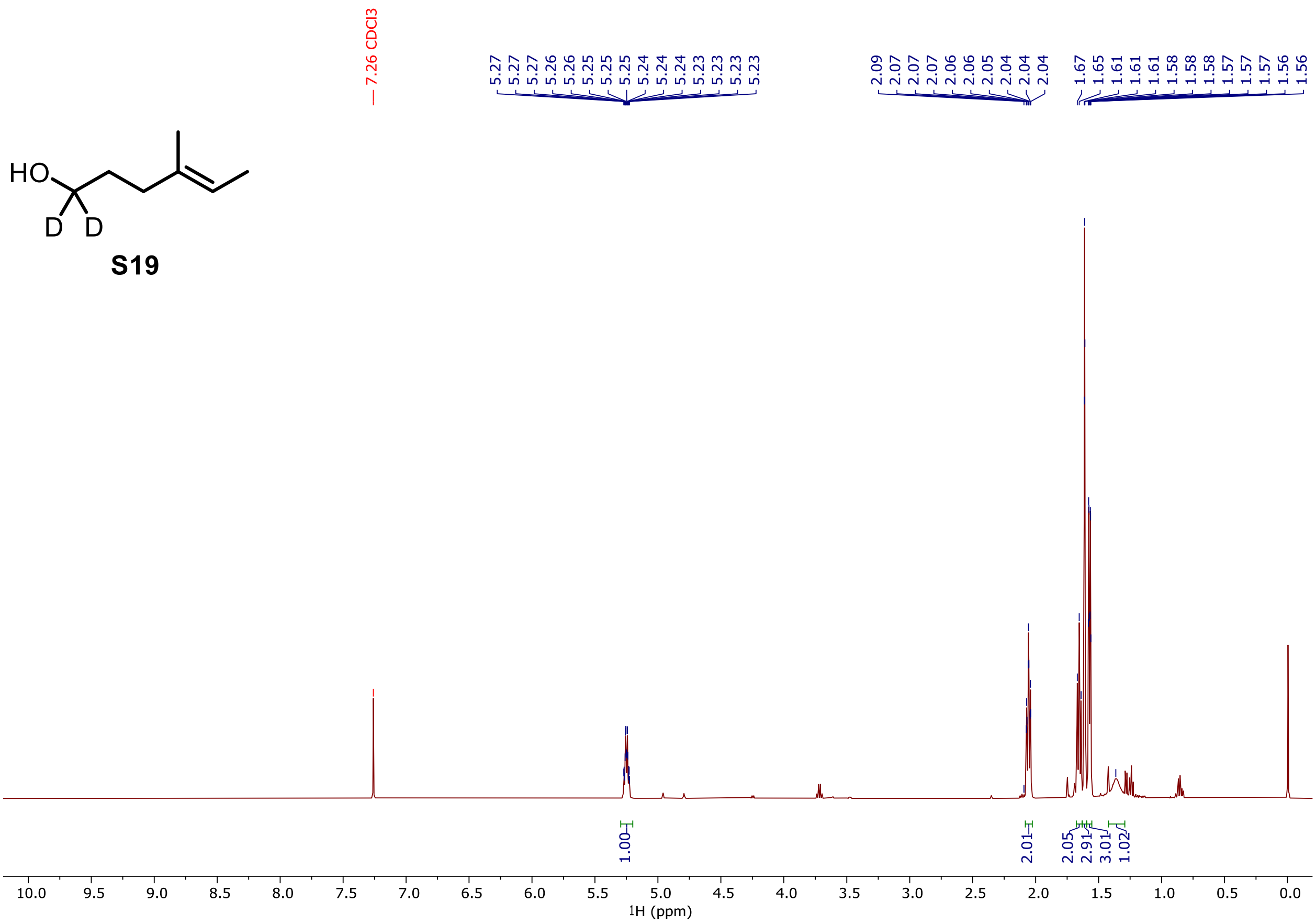

^{13}C NMR spectrum of **S19**

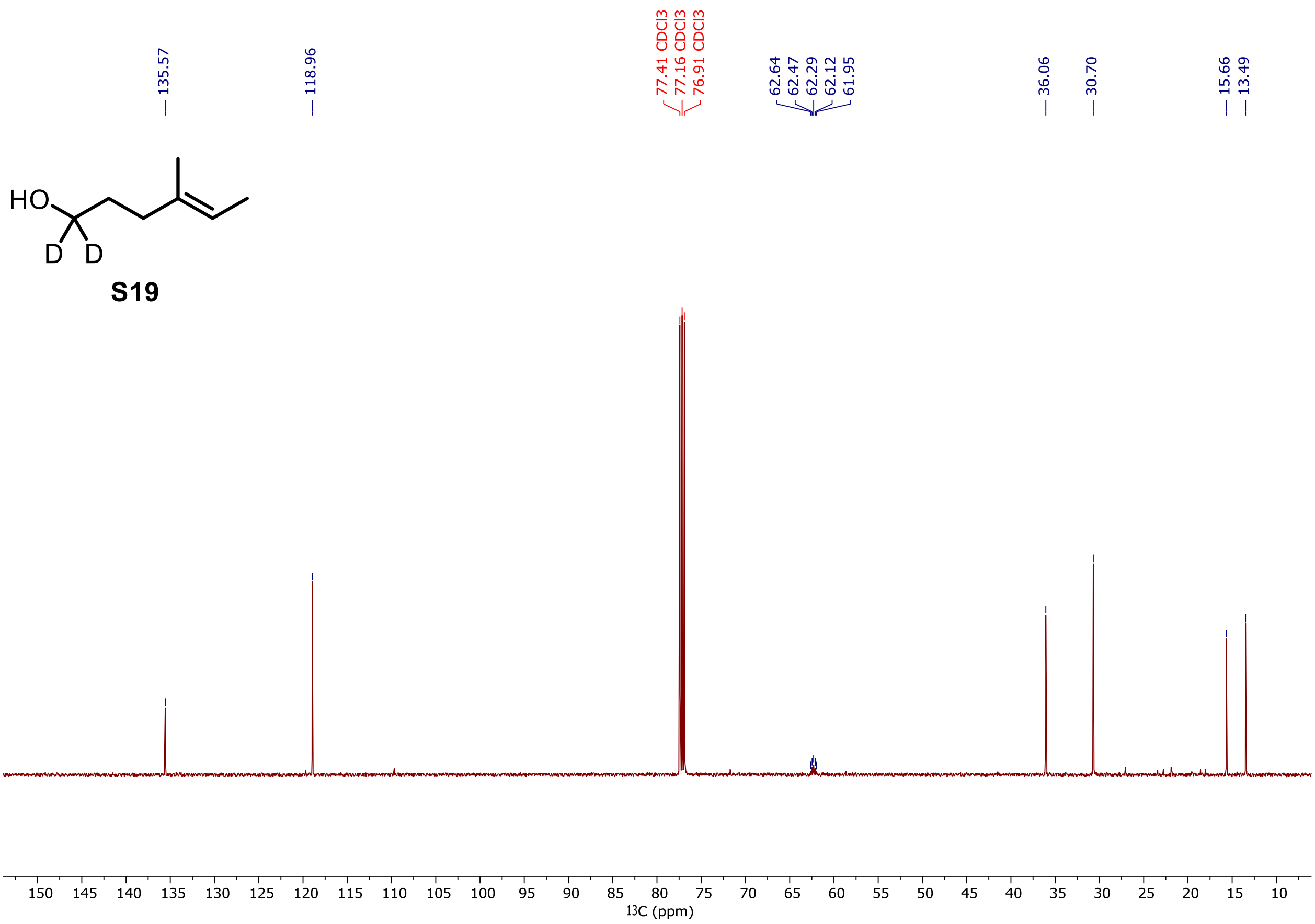

^{1}H NMR spectrum of **S22**

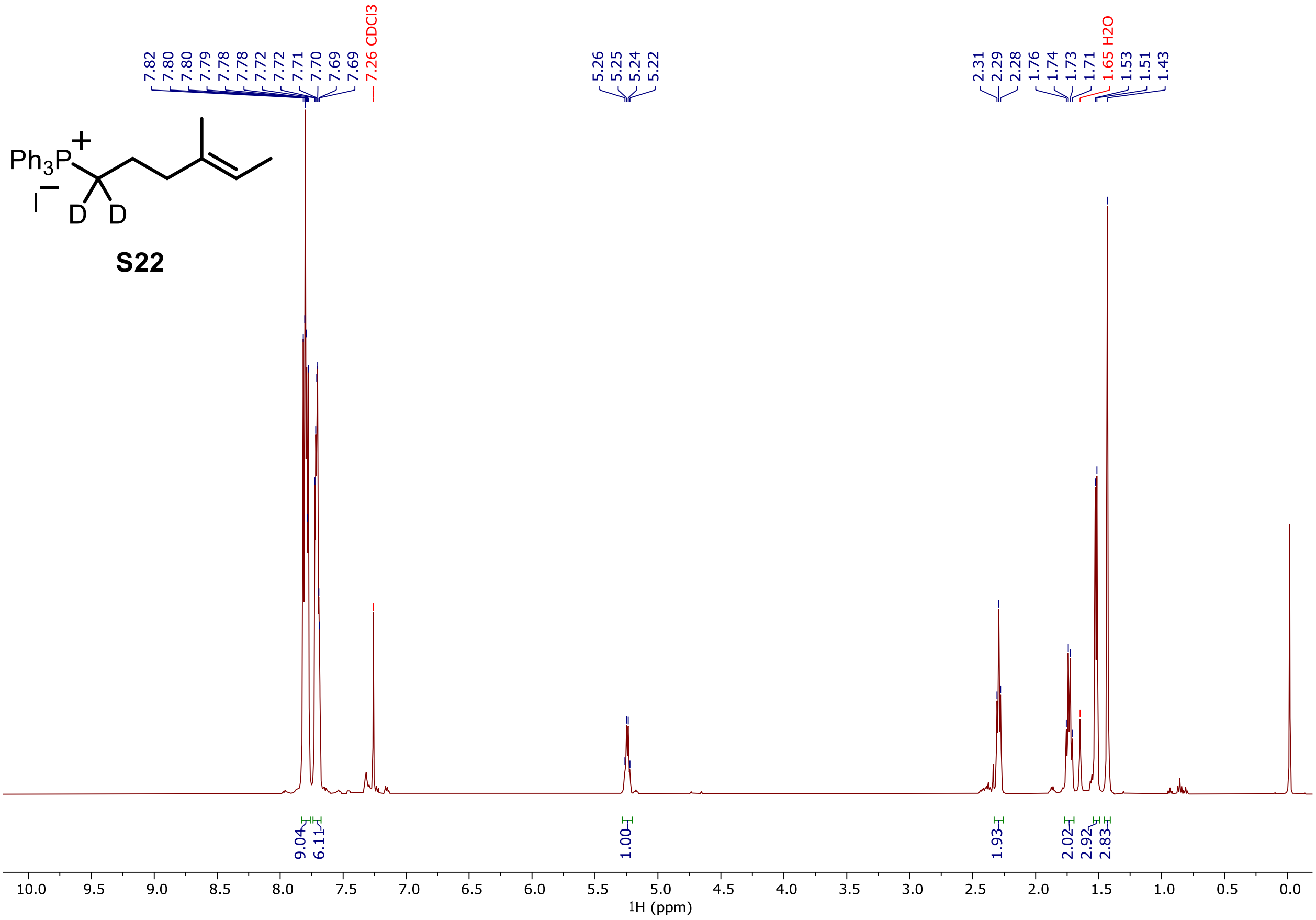

^{31}P NMR spectrum of **S22**

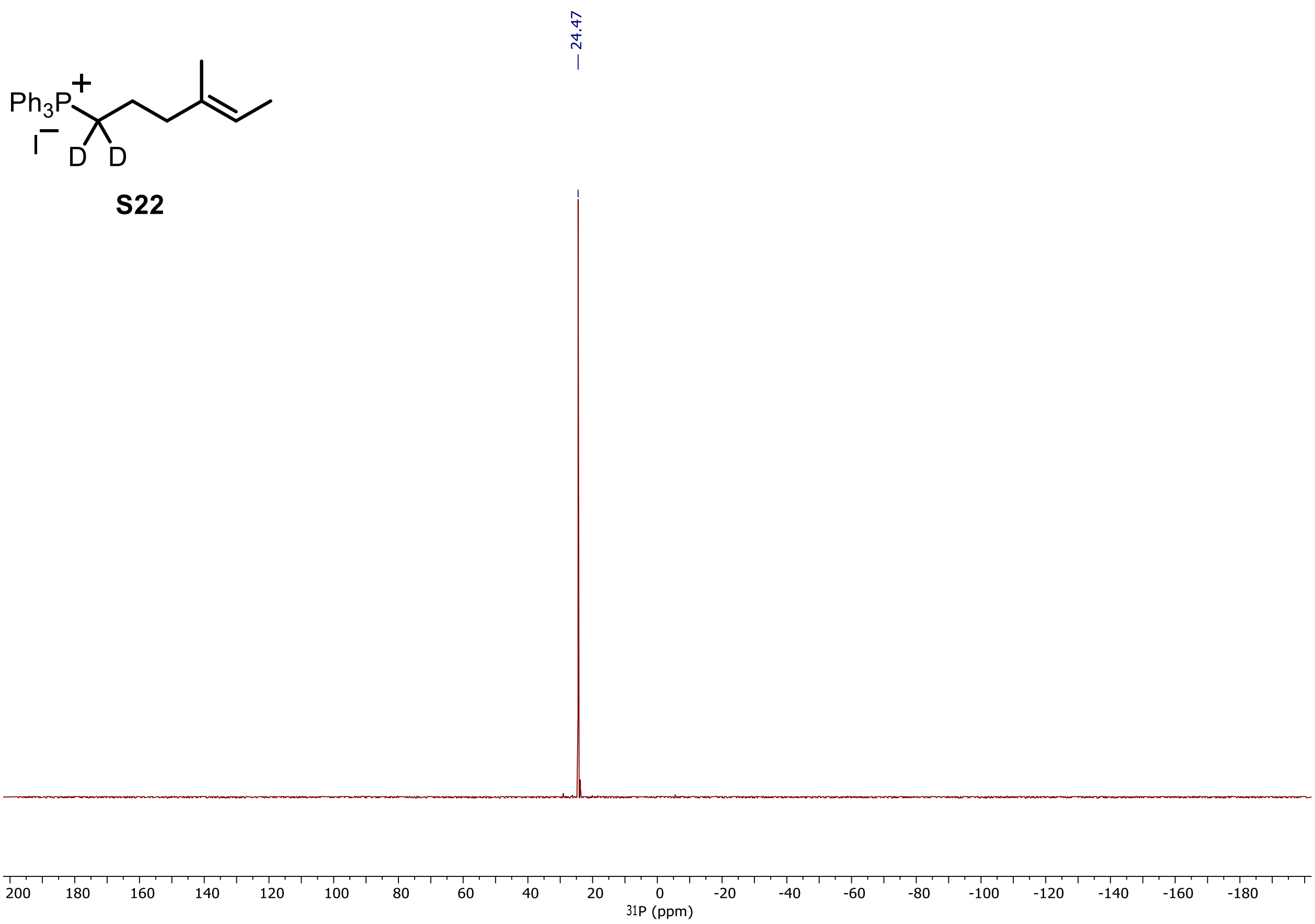

^{13}C NMR spectrum of **S22**

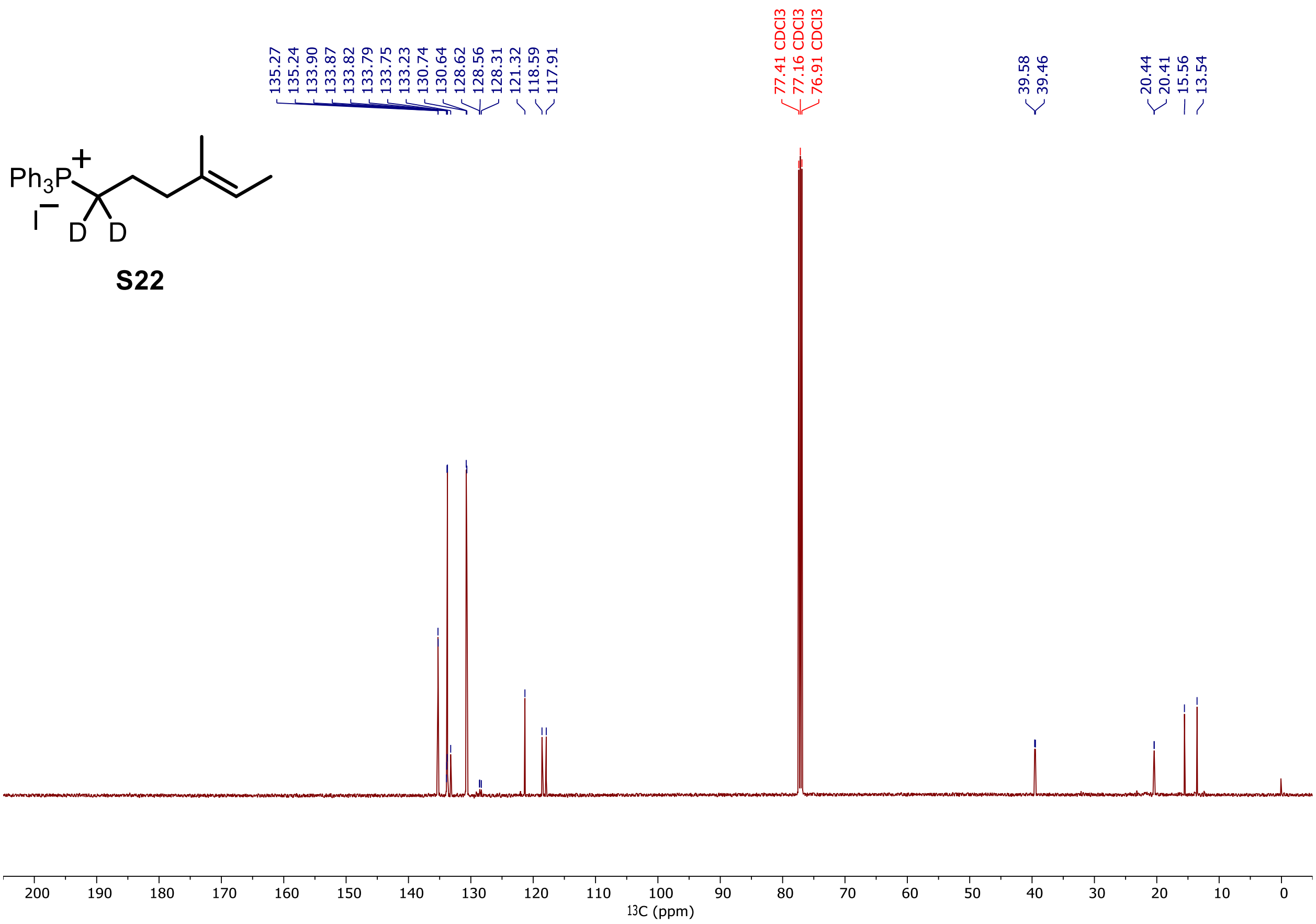

^{1}H NMR spectrum of **1a-d_1**

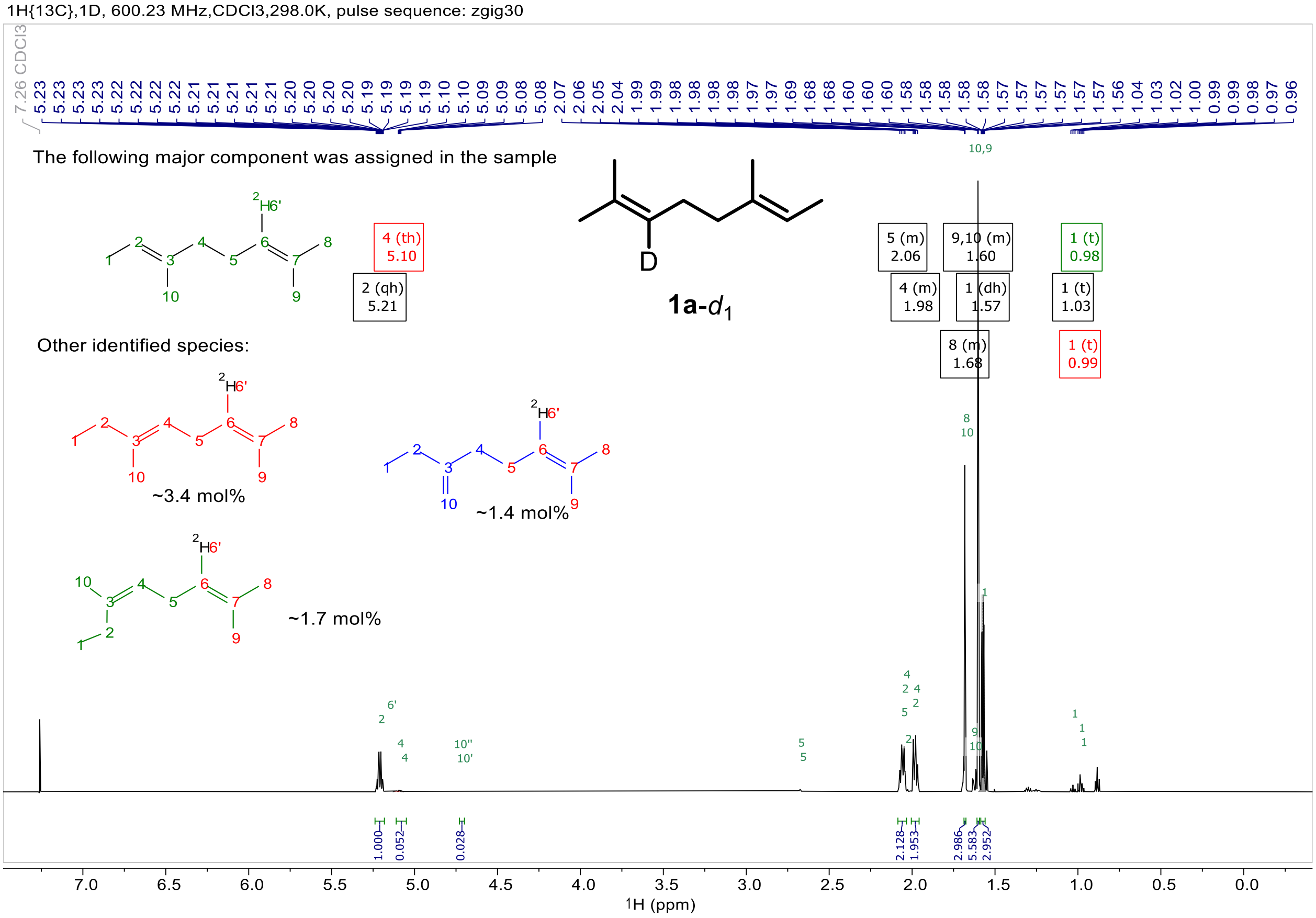

^{13}C NMR spectrum of **1a-*d*$_1$**

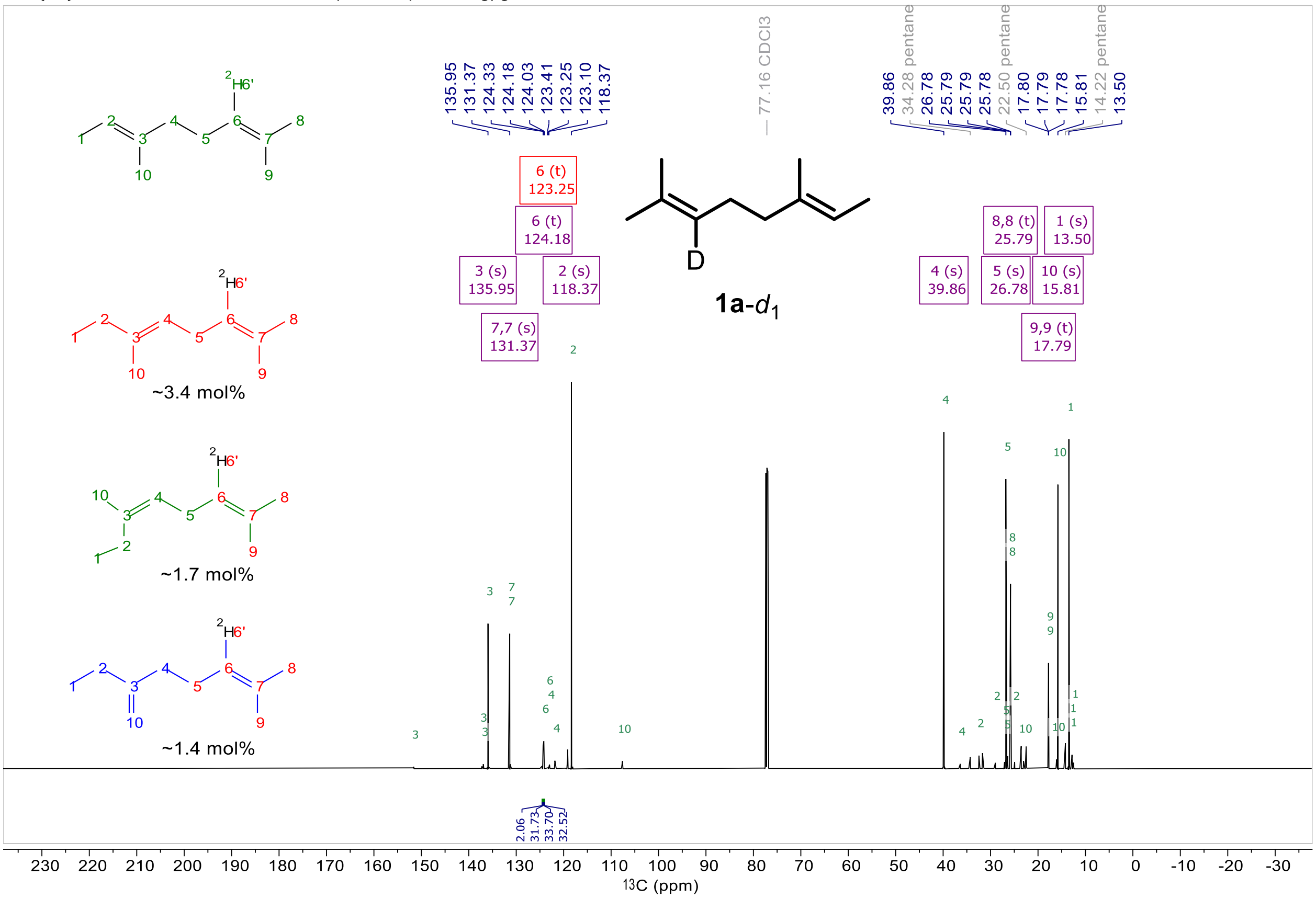

^{1}H,^{13}C-HSQC NMR spectrum of **1a-*d*$_1$**

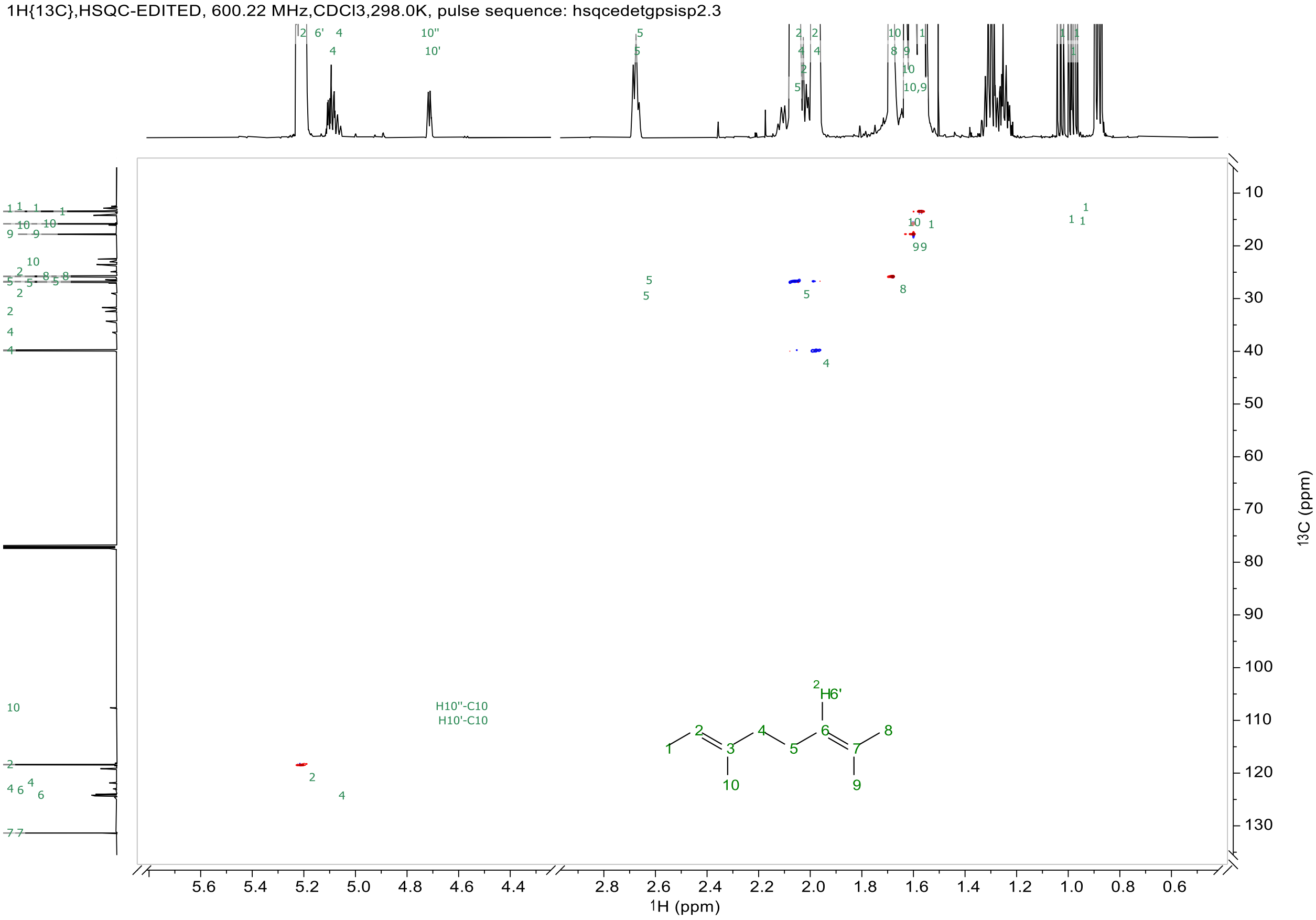

$^{1}H,^{13}C$-HMBC NMR spectrum of **1a-*d*$_1$**

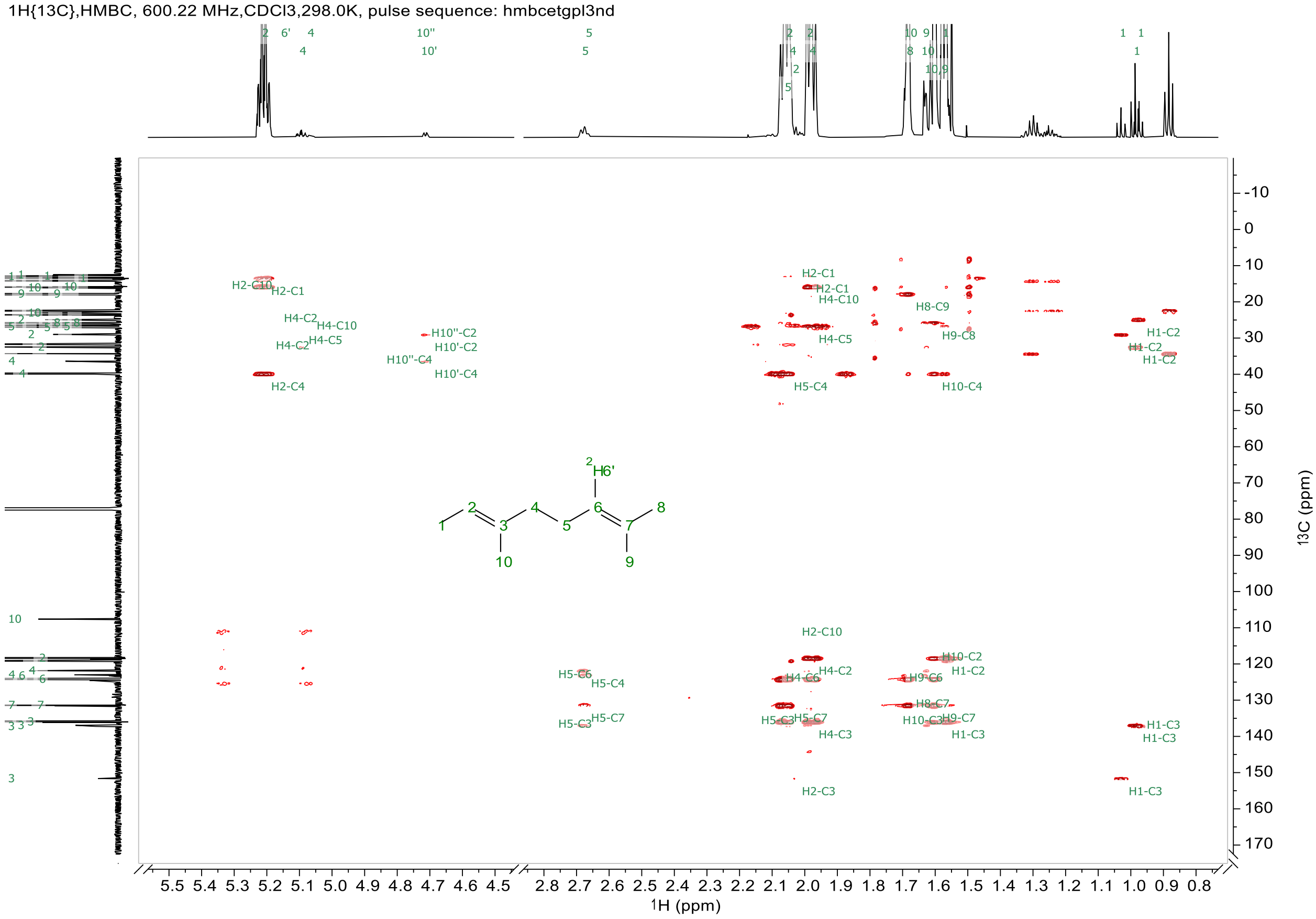

$^{1}H,^{1}H$-COSY NMR spectrum of **1a-*d*$_1$**

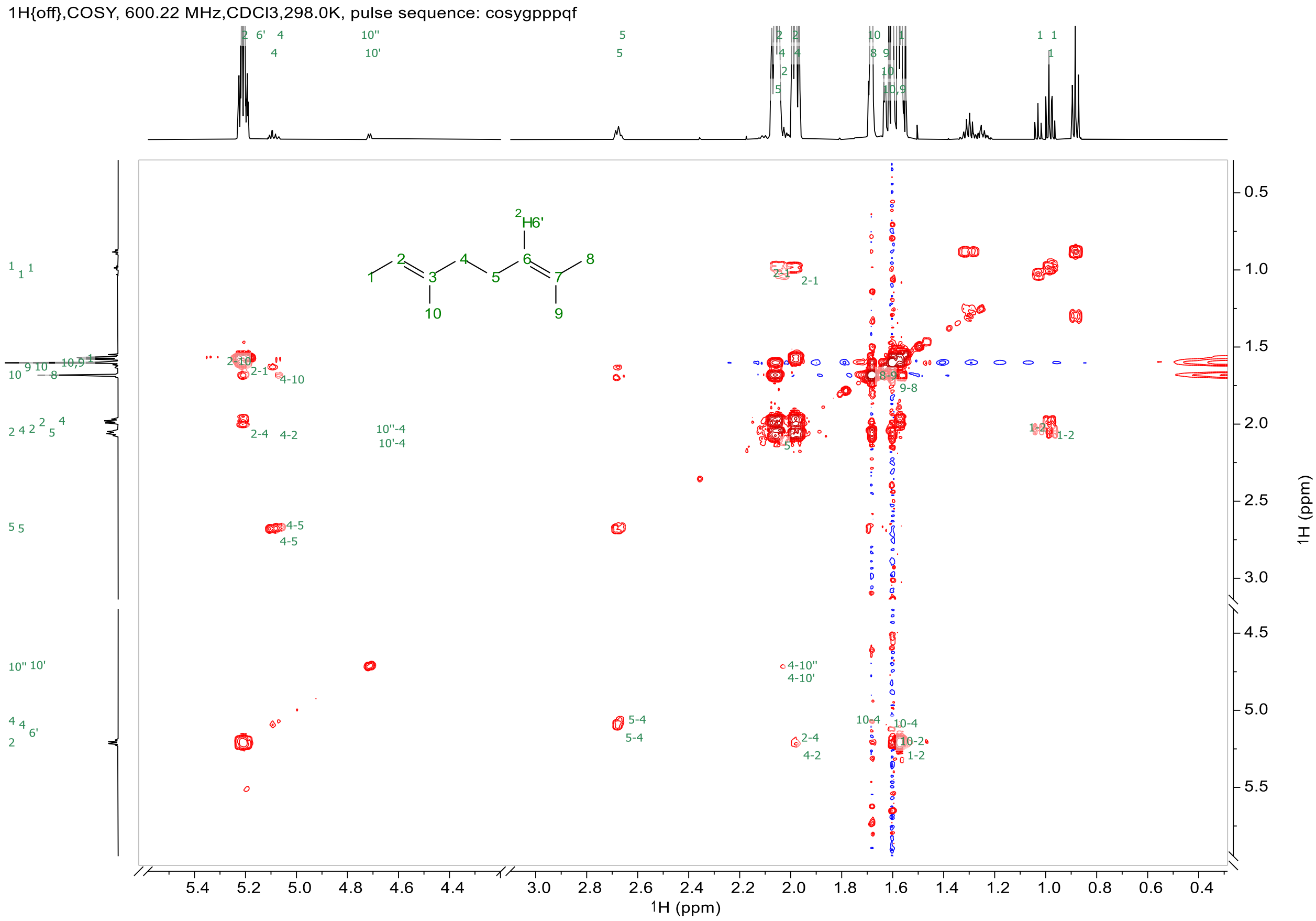

^{1}H,^{1}H-NOESY NMR spectrum of **1a-*d*$_1$**

1H{off},NOESY, 600.22 MHz,CDCl3,298.0K, pulse sequence: noesygpph

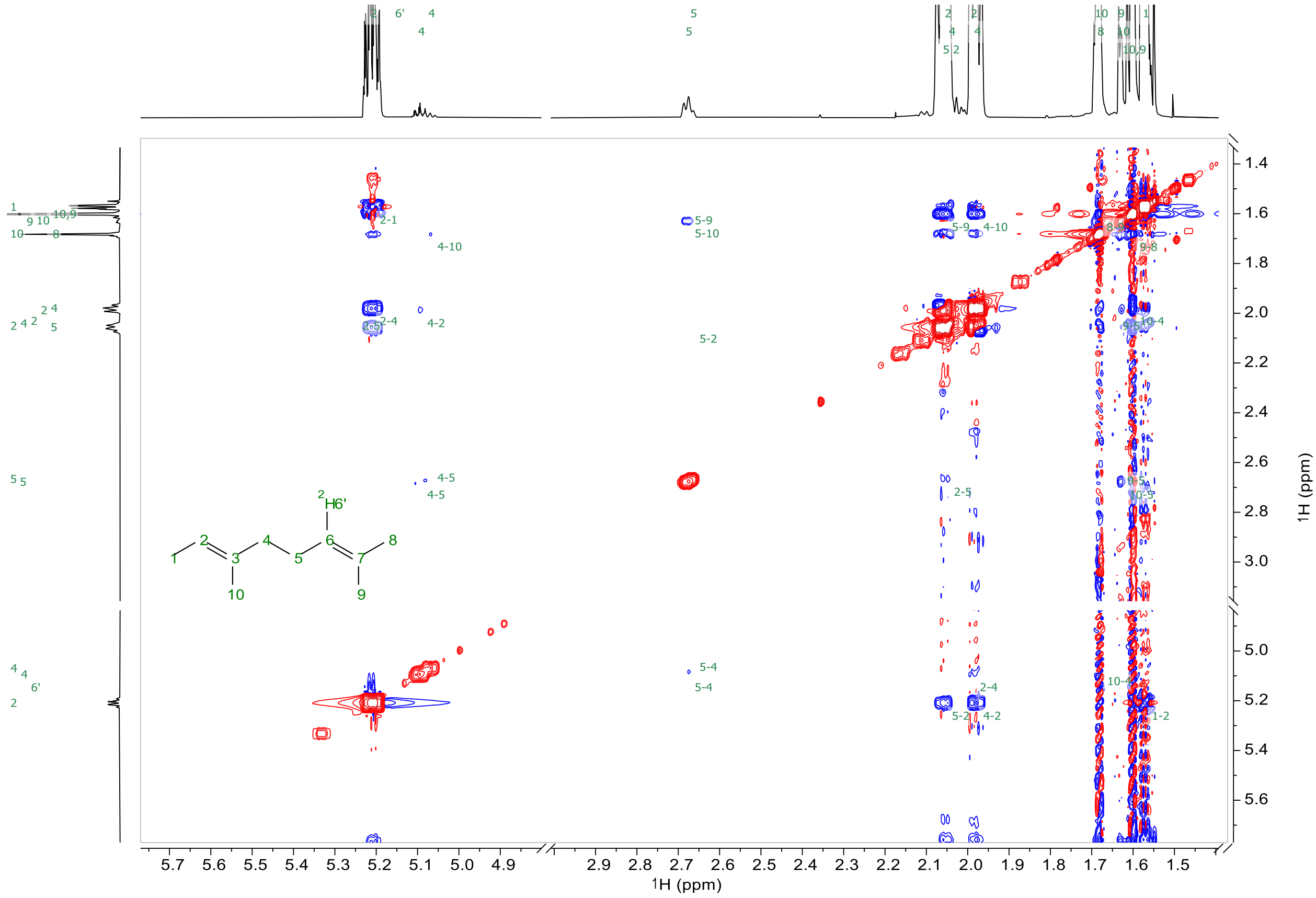

^{2}H NMR spectrum of **1a-*d*$_1$**

2H{1H},1D, 92.14 MHz,CDCl3,298.0K, pulse sequence: zgig2h

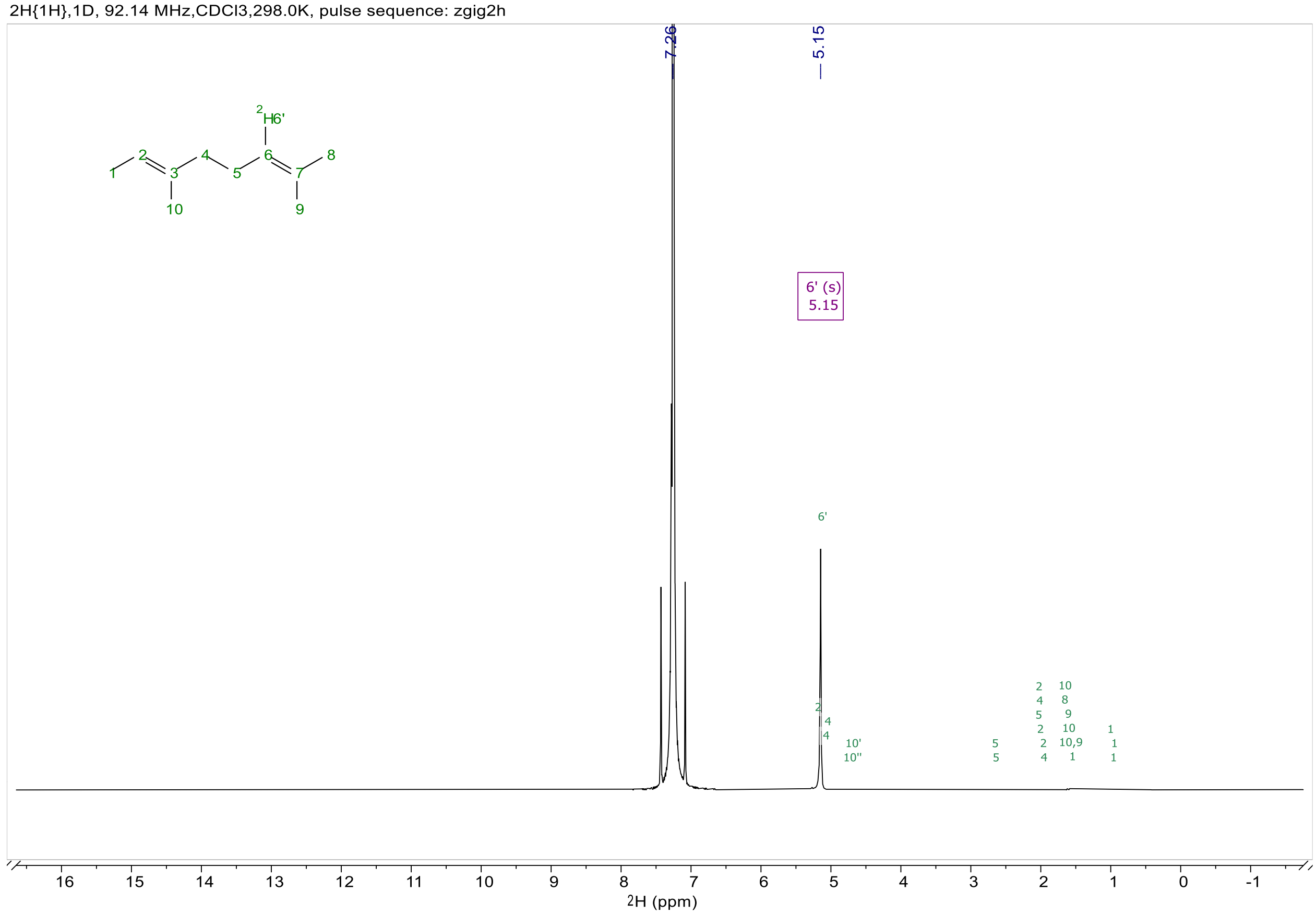

^{1}H NMR spectrum of **1a-*d*$_7$**

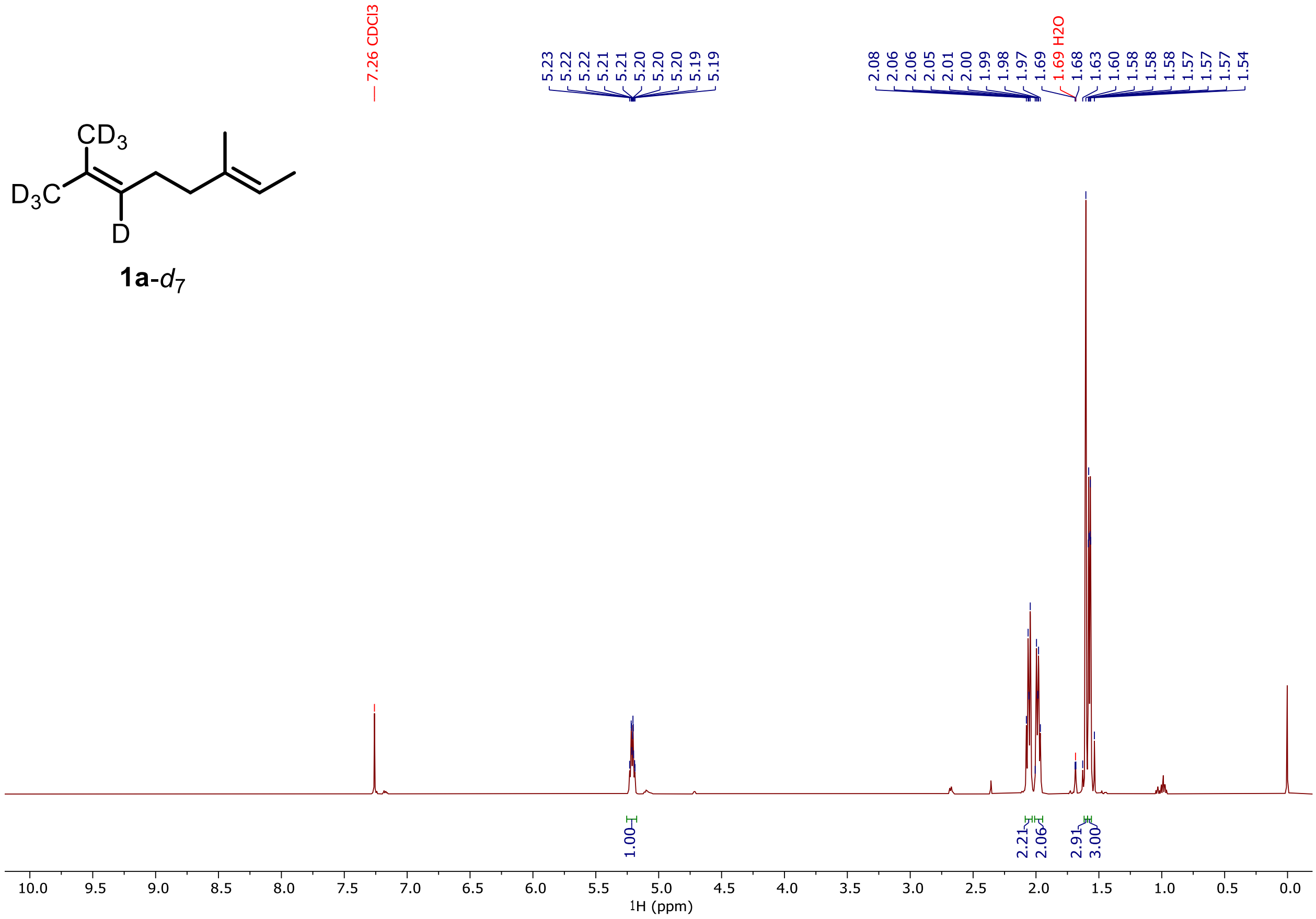

^{13}C NMR spectrum of **1a-*d*$_7$**

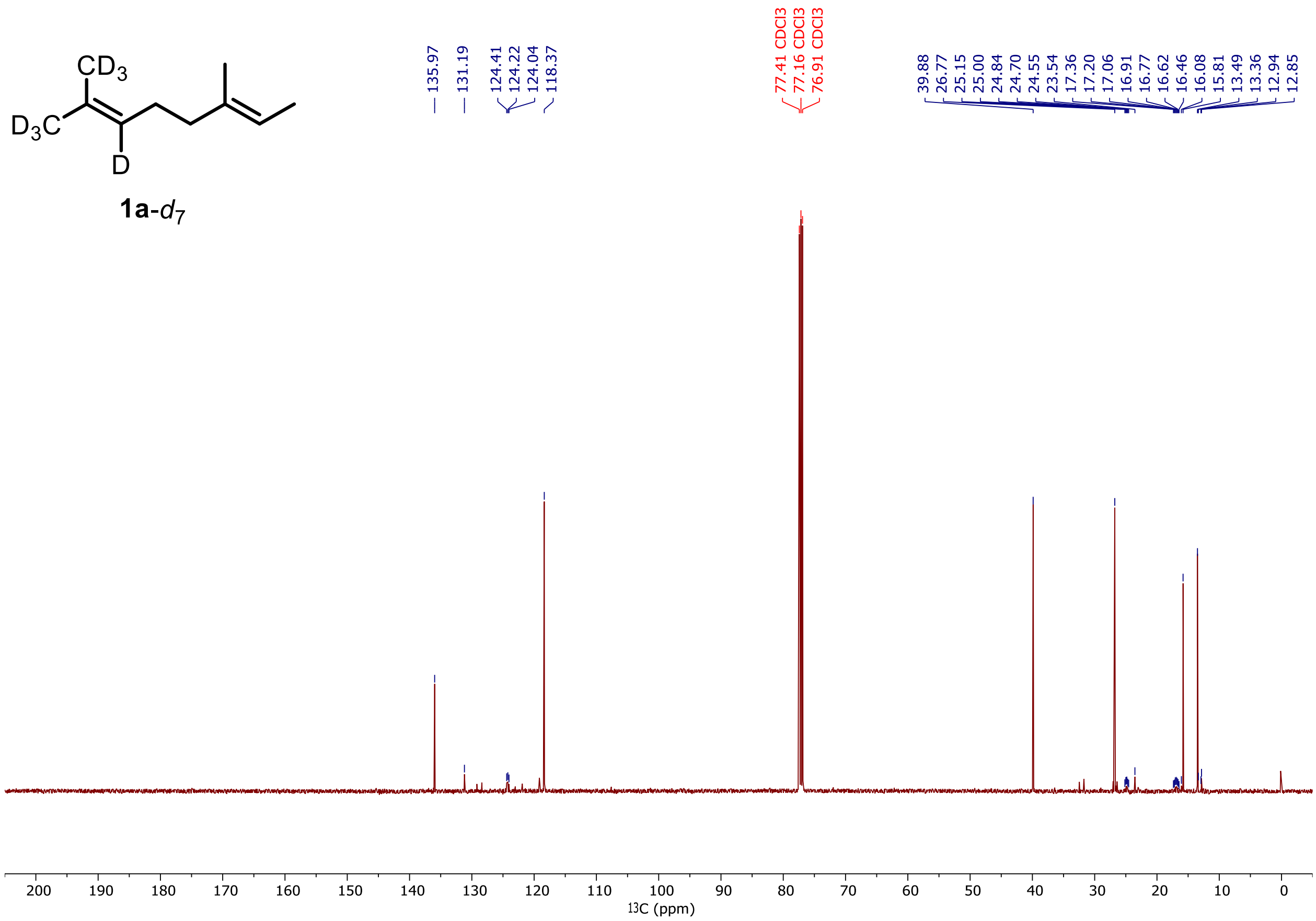

12. Crystallographic data

Crystallographic data for osmate ester 6a

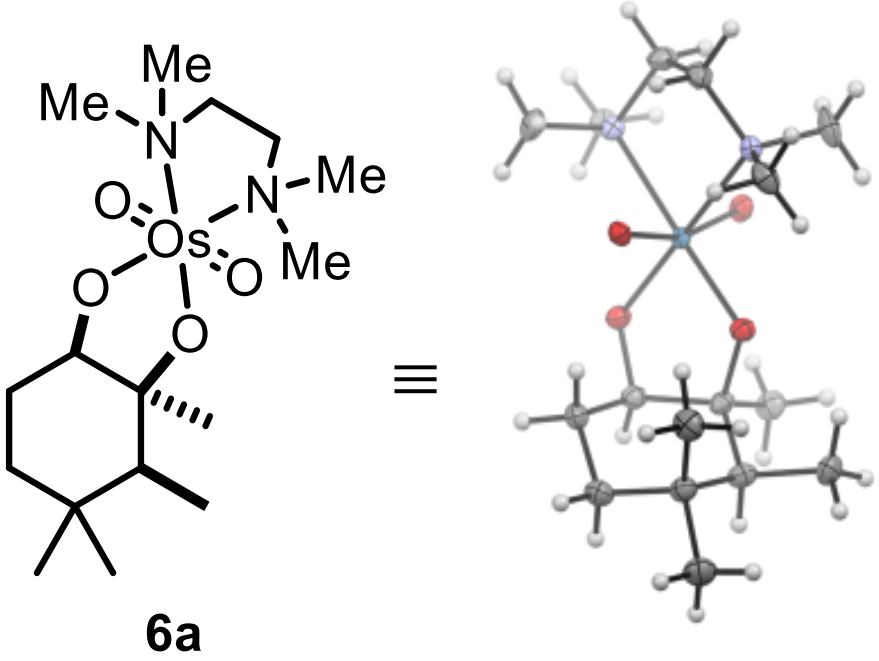

6a

(major diastereomer)
CCDC 2543369

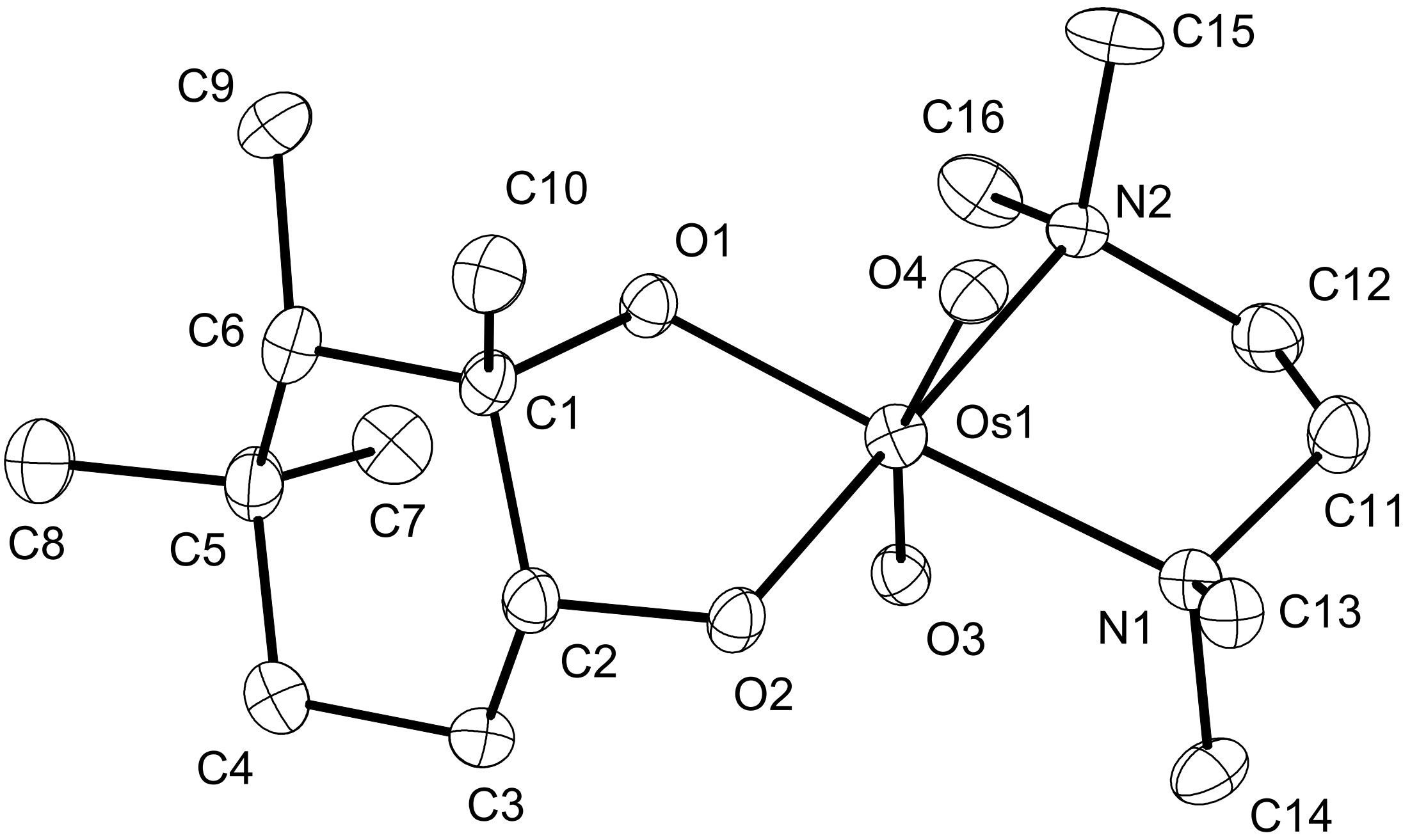

Figure S21. The structure of osmate ester **6a** in the solid state. Hydrogen atoms have been removed for clarity in the lower ORTEP representation. Atom numbering corresponds to the bond lengths and angles listed in Table S9.

Table S10. Crystal data and structure refinement for osmate ester **6a**.

Identification code	16443 (CCDC: 2543369)
Empirical formula	$C_{16} H_{34} N_2 O_4 Os$
Color	light yellow
Formula weight	508.684 g·mol^{-1}
Temperature	100(2) K
Wavelength	0.71073 Å
Crystal system	Orthorhombic
Space group	$P2_12_12_1$, (no. 19)
Unit cell dimensions	α = 7.6203(4) Å α= 90°<br>β = 11.0429(7) Å β= 90°<br>γ = 22.8394(16) Å γ = 90°.
Volume	1921.9(2) Å^3
Z	4
Density (calculated)	1.758 Mg·m^{-3}
Absorption coefficient	6.652 mm^{-1}
F(000)	1005.873 e
Crystal size	0.116 x 0.081 x 0.011 mm^3
θ range for data collection	2.05 to 31.55°.
Index ranges	-11 ≤ h ≤ 11, -16≤ k ≤ 16, -33 ≤ l ≤ 33
Reflections collected	226956
Independent reflections	6413 [R_{int} = 0.0593]
Reflections with I>2σ(I)	6273
Completeness to θ = 25.2417°	99.95 %
Absorption correction	Gaussian
Max. and min. transmission	0.93894 and 0.60801
Refinement method	Full-matrix least-squares on F^2
Data / restraints / parameters	6413 / 0 / 242
Goodness-of-fit on F^2	1.0386
Final R indices [I>2σ (I)]	R_1 = 0.0177 wR^2 = 0.0379
R indices (all data)	R_1 = 0.0184 wR^2 = 0.0382
Absolute structure parameter	0.031(7)
Largest diff. peak and hole	1.0858 and -0.6431 e·Å^{-3}

Table S11. Bond lengths [Å] and angles [°] of osmate ester **6a**.

Os(1)-O(1)	1.9350(16)	Os(1)-O(2)	1.958(2)	Os(1)-
O(3)	1.752(2)	Os(1)-O(4)	1.752(2)	Os(1)-
N(1)	2.248(2)	Os(1)-N(2)	2.224(3)	O(1)-
C(1)	1.451(3)	O(2)-C(2)	1.441(3)	N(1)-
C(11)	1.508(4)	N(1)-C(13)	1.489(4)	N(1)-
C(14)	1.480(3)	N(2)-C(12)	1.508(4)	N(2)-
C(15)	1.478(4)	N(2)-C(16)	1.500(4)	C(1)-
C(2)	1.531(4)	C(1)-C(6)	1.553(4)	C(1)-
C(10)	1.527(4)	C(2)-C(3)	1.524(4)	C(2)-
H(2)	1.0000	C(3)-C(4)	1.529(4)	C(3)-
H(3a)	0.9900	C(3)-H(3b)	0.9900	C(4)-
C(5)	1.541(4)	C(4)-H(4a)	0.9900	C(4)-
H(4b)	0.9900	C(5)-C(6)	1.561(4)	C(5)-
C(7)	1.542(4)	C(5)-C(8)	1.546(4)	C(6)-
C(9)	1.544(4)	C(6)-H(6)	1.0000	C(7)-
H(7a)	0.9800	C(7)-H(7b)	0.9800	C(7)-
H(7c)	0.9800	C(8)-H(8a)	0.9800	C(8)-
H(8b)	0.9800	C(8)-H(8c)	0.9800	C(9)-
H(9a)	0.9800	C(9)-H(9b)	0.9800	C(9)-
H(9c)	0.9800	C(10)-H(10a)	0.9800	C(10)-
H(10b)	0.9800	C(10)-H(10c)	0.9800	C(11)-
C(12)	1.502(4)	C(11)-H(11a)	0.9900	C(11)-
H(11b)	0.9900	C(12)-H(12a)	0.9900	C(12)-
H(12b)	0.9900	C(13)-H(13a)	0.9800	C(13)-
H(13b)	0.9800	C(13)-H(13c)	0.9800	C(14)-
H(14a)	0.9800	C(14)-H(14b)	0.9800	C(14)-
H(14c)	0.9800	C(15)-H(15a)	0.9800	C(15)-
H(15b)	0.9800	C(15)-H(15c)	0.9800	C(16)-
H(16a)	0.9800	C(16)-H(16b)	0.9800	C(16)-
H(16c)	0.9800			
O(2)-Os(1)-O(1)	84.33(9)	O(3)-Os(1)-O(1)	96.59(10)	O(3)-
Os(1)-O(2)	96.85(8)	O(4)-Os(1)-O(1)	98.07(9)	O(4)-
Os(1)-O(2)	96.55(10)	O(4)-Os(1)-O(3)	161.01(10)	N(1)-
Os(1)-O(1)	177.28(10)	N(1)-Os(1)-O(2)	98.30(9)	N(1)-
Os(1)-O(3)	82.42(10)	N(1)-Os(1)-O(4)	82.37(9)	N(2)-
Os(1)-O(1)	95.70(9)	N(2)-Os(1)-O(2)	179.27(10)	N(2)-
Os(1)-O(3)	82.42(9)	N(2)-Os(1)-O(4)	84.17(10)	N(2)-
Os(1)-N(1)	81.66(9)	C(1)-O(1)-Os(1)	113.24(17)	C(2)-
O(2)-Os(1)	110.97(16)	C(11)-N(1)-Os(1)	105.05(16)	C(13)-
N(1)-Os(1)	111.40(17)	C(13)-N(1)-C(11)	108.9(2)	C(14)-
N(1)-Os(1)	113.06(17)	C(14)-N(1)-C(11)	110.4(2)	C(14)-
N(1)-C(13)	107.9(2)	C(12)-N(2)-Os(1)	105.65(17)	C(15)-
N(2)-Os(1)	115.32(19)	C(15)-N(2)-C(12)	111.2(3)	C(16)-
N(2)-Os(1)	110.15(18)	C(16)-N(2)-C(12)	107.6(2)	C(16)-
N(2)-C(15)	106.7(2)	C(2)-C(1)-O(1)	107.2(2)	C(6)-
C(1)-O(1)	108.6(2)	C(6)-C(1)-C(2)	113.9(2)	C(10)-
C(1)-O(1)	107.7(2)	C(10)-C(1)-C(2)	108.6(2)	C(10)-

C(1)-C(6)	110.6(2)	C(1)-C(2)-O(2)	108.2(2)	C(3)-
C(2)-O(2)	109.3(2)	C(3)-C(2)-C(1)	115.0(2)	H(2)-
C(2)-O(2)	108.04(14)	H(2)-C(2)-C(1)	108.04(14)	H(2)-
C(2)-C(3)	108.04(15)	C(4)-C(3)-C(2)	112.0(2)	H(3a)-
C(3)-C(2)	109.21(15)	H(3a)-C(3)-C(4)	109.21(17)	H(3b)-
C(3)-C(2)	109.21(15)	H(3b)-C(3)-C(4)	109.21(16)	H(3b)-
C(3)-H(3a)	107.9	C(5)-C(4)-C(3)	112.2(2)	H(4a)-
C(4)-C(3)	109.17(17)	H(4a)-C(4)-C(5)	109.17(16)	H(4b)-
C(4)-C(3)	109.17(16)	H(4b)-C(4)-C(5)	109.17(16)	H(4b)-
C(4)-H(4a)	107.9	C(6)-C(5)-C(4)	107.7(2)	C(7)-
C(5)-C(4)	110.5(3)	C(7)-C(5)-C(6)	113.4(3)	C(8)-
C(5)-C(4)	106.9(3)	C(8)-C(5)-C(6)	109.9(3)	C(8)-
C(5)-C(7)	108.2(3)	C(5)-C(6)-C(1)	114.7(2)	C(9)-
C(6)-C(1)	110.1(2)	C(9)-C(6)-C(5)	112.3(3)	H(6)-
C(6)-C(1)	106.38(15)	H(6)-C(6)-C(5)	106.38(15)	H(6)-
C(6)-C(9)	106.38(19)	H(7a)-C(7)-C(5)	109.5	H(7b)-
C(7)-C(5)	109.5	H(7b)-C(7)-H(7a)	109.5	H(7c)-
C(7)-C(5)	109.5	H(7c)-C(7)-H(7a)	109.5	H(7c)-
C(7)-H(7b)	109.5	H(8a)-C(8)-C(5)	109.5	H(8b)-
C(8)-C(5)	109.5	H(8b)-C(8)-H(8a)	109.5	H(8c)-
C(8)-C(5)	109.5	H(8c)-C(8)-H(8a)	109.5	H(8c)-
C(8)-H(8b)	109.5	H(9a)-C(9)-C(6)	109.5	H(9b)-
C(9)-C(6)	109.5	H(9b)-C(9)-H(9a)	109.5	H(9c)-
C(9)-C(6)	109.5	H(9c)-C(9)-H(9a)	109.5	H(9c)-
C(9)-H(9b)	109.5	H(10a)-C(10)-C(1)	109.5	H(10b)-
C(10)-C(1)	109.5	H(10b)-C(10)-H(10a)	109.5	H(10c)-
C(10)-C(1)	109.5	H(10c)-C(10)-H(10a)	109.5	H(10c)-
C(10)-H(10b)	109.5	C(12)-C(11)-N(1)	111.9(2)	H(11a)-
C(11)-N(1)	109.23(16)	H(11a)-C(11)-C(12)	109.23(18)	H(11b)-
C(11)-N(1)	109.23(14)	H(11b)-C(11)-C(12)	109.23(16)	H(11b)-
C(11)-H(11a)	107.9	C(11)-C(12)-N(2)	109.4(2)	H(12a)-
C(12)-N(2)	109.78(16)	H(12a)-C(12)-C(11)	109.78(18)	H(12b)-
C(12)-N(2)	109.78(15)	H(12b)-C(12)-C(11)	109.78(17)	H(12b)-
C(12)-H(12a)	108.2	H(13a)-C(13)-N(1)	109.5	H(13b)-
C(13)-N(1)	109.5	H(13b)-C(13)-H(13a)	109.5	H(13c)-
C(13)-N(1)	109.5	H(13c)-C(13)-H(13a)	109.5	H(13c)-
C(13)-H(13b)	109.5	H(14a)-C(14)-N(1)	109.5	H(14b)-
C(14)-N(1)	109.5	H(14b)-C(14)-H(14a)	109.5	H(14c)-
C(14)-N(1)	109.5	H(14c)-C(14)-H(14a)	109.5	H(14c)-
C(14)-H(14b)	109.5	H(15a)-C(15)-N(2)	109.5	H(15b)-
C(15)-N(2)	109.5	H(15b)-C(15)-H(15a)	109.5	H(15c)-
C(15)-N(2)	109.5	H(15c)-C(15)-H(15a)	109.5	H(15c)-
C(15)-H(15b)	109.5	H(16a)-C(16)-N(2)	109.5	H(16b)-
C(16)-N(2)	109.5	H(16b)-C(16)-H(16a)	109.5	H(16c)-
C(16)-N(2)	109.5	H(16c)-C(16)-H(16a)	109.5	H(16c)-
C(16)-H(16b)	109.5			

Crystallographic data for osmate 6f · H_2O · 0.25 THF

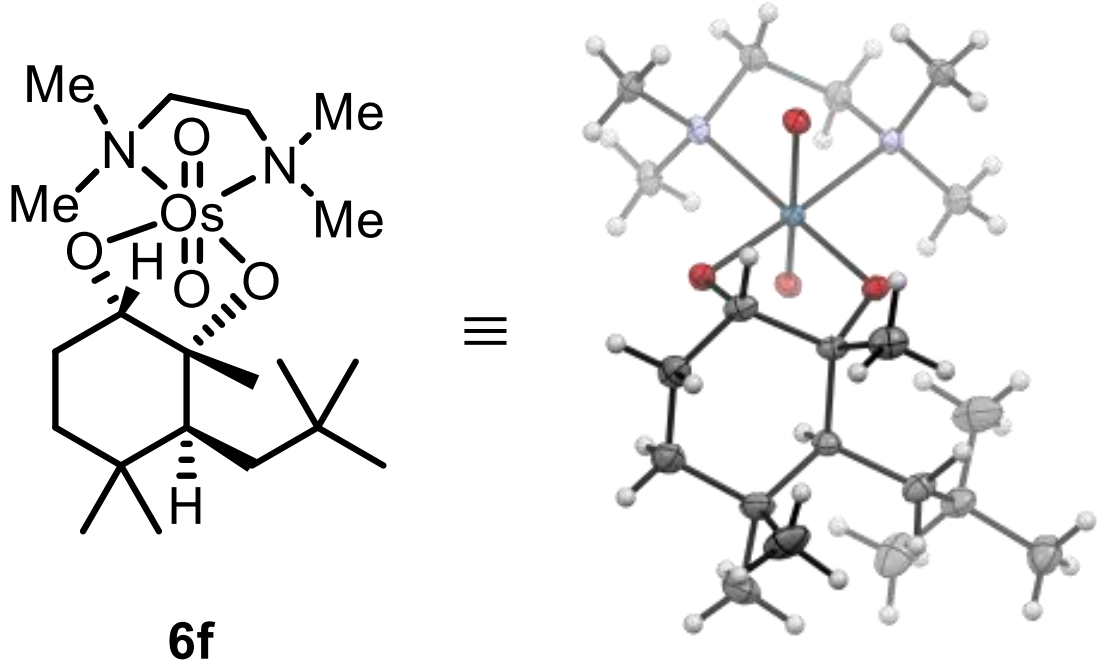

6f
(major diastereomer)
CCDC 2543368

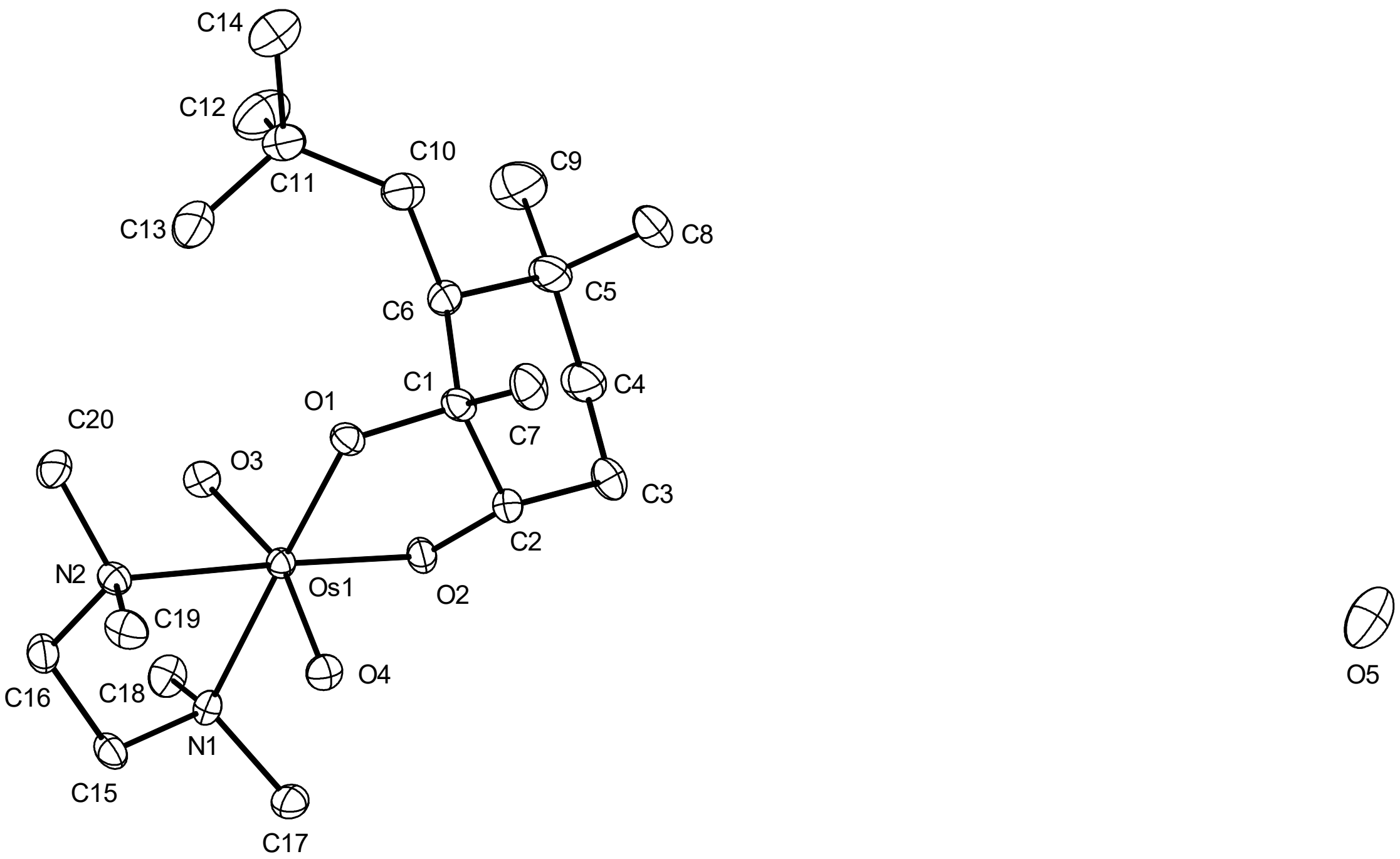

Figure S22. The structure of osmate ester **6f** · H_2O 0.25 THF in the solid state. The water molecule has been removed for clarity in the upper ORTEP representation. Disordered solvent (0.25 THF) has been removed for clarity (solvent was masked in the refinement process). Hydrogen atoms have been removed for clarity in the lower ORTEP representation. Atom numbering corresponds to the bond lengths and angles listed in Table S11.

Table S12. Crystal data and structure refinement for osmate ester **6f** · H_2O · 0.25 THF.

Identification code	16437 (CCDC: 2543368)
Empirical formula	$C_{21} H_{46.50} N_2 O_{5.25} Os$
Color	yellow
Formula weight	601.30 g·mol^{-1}
Temperature	150(2) K
Wavelength	0.71073 Å
Crystal system	Tetragonal
Space group	*I*4, (no. 79)
Unit cell dimensions	α = 24.9769(7) Å α= 90°.<br>β = 24.9769(7) Å β= 90°.<br>γ = 8.1036(3) Å γ= 90°.
Volume	5055.4(3) Å^3
Z	8
Density (calculated)	1.580 Mg·m^{-3}
Absorption coefficient	5.077 mm^{-1}
F(000)	2436 e
Crystal size	0.10 x 0.031 x 0.023 mm^3
θ range for data collection	1.153 to 31.283°.
Index ranges	$-36 \le h \le 36$, $-36 \le k \le 36$, $-11 \le l \le 11$
Reflections collected	85389
Independent reflections	8252 [R_{int} = 0.0511]
Reflections with I>2σ(I)	6920
Completeness to θ = 25.242°	100.0 %
Absorption correction	Gaussian
Max. and min. transmission	0.90625 and 0.71490
Refinement method	Full-matrix least-squares on F^2
Data / restraints / parameters	8252 / 1 / 270
Goodness-of-fit on F^2	1.029
Final R indices [I>2σ(I)]	R_1 = 0.0210 wR^2 = 0.0380
R indices (all data)	R_1 = 0.0309 wR^2 = 0.0398
Absolute structure parameter	-0.002(4)
Extinction coefficient	n/a
Largest diff. peak and hole	0.442 and -0.378 e·Å^{-3}

Table S13. Bond lengths [Å] and angles [°] for osmate ester **6f** · H_2O · 0.25 THF.

Os(1)-O(1)	1.963(3)	Os(1)-O(2)	1.943(3)	Os(1)-
O(3)	1.732(2)	Os(1)-O(4)	1.747(2)	Os(1)-
N(1)	2.226(3)	Os(1)-N(2)	2.250(3)	O(1)-
C(1)	1.453(5)	O(2)-C(2)	1.440(5)	N(1)-
C(15)	1.499(5)	N(1)-C(17)	1.486(5)	N(1)-
C(18)	1.475(5)	N(2)-C(16)	1.496(5)	N(2)-
C(19)	1.481(5)	N(2)-C(20)	1.481(5)	C(1)-
C(2)	1.523(5)	C(1)-C(6)	1.573(5)	C(1)-
C(7)	1.516(7)	C(2)-H(2)	1.0000	C(2)-
C(3)	1.539(7)	C(3)-H(3A)	0.9900	C(3)-
H(3B)	0.9900	C(3)-C(4)	1.493(7)	C(4)-
H(4A)	0.9900	C(4)-H(4B)	0.9900	C(4)-
C(5)	1.524(6)	C(5)-C(6)	1.564(6)	C(5)-
C(8)	1.523(6)	C(5)-C(9)	1.552(6)	C(6)-
H(6)	0.83(4)	C(6)-C(10)	1.548(6)	C(7)-
H(7A)	0.9800	C(7)-H(7B)	0.9800	C(7)-
H(7C)	0.9800	C(8)-H(8A)	0.9800	C(8)-
H(8B)	0.9800	C(8)-H(8C)	0.9800	C(9)-
H(9A)	0.9800	C(9)-H(9B)	0.9800	C(9)-
H(9C)	0.9800	C(10)-H(10A)	0.9900	C(10)-
H(10B)	0.9900	C(10)-C(11)	1.543(6)	C(11)-
C(12)	1.537(6)	C(11)-C(13)	1.511(6)	C(11)-
C(14)	1.514(6)	C(12)-H(12A)	0.9800	C(12)-
H(12B)	0.9800	C(12)-H(12C)	0.9800	C(13)-
H(13A)	0.9800	C(13)-H(13B)	0.9800	C(13)-
H(13C)	0.9800	C(14)-H(14A)	0.9800	C(14)-
H(14B)	0.9800	C(14)-H(14C)	0.9800	C(15)-
H(15A)	0.9900	C(15)-H(15B)	0.9900	C(15)-
C(16)	1.498(5)	C(16)-H(16A)	0.9900	C(16)-
H(16B)	0.9900	C(17)-H(17A)	0.9800	C(17)-
H(17B)	0.9800	C(17)-H(17C)	0.9800	C(18)-
H(18A)	0.9800	C(18)-H(18B)	0.9800	C(18)-
H(18C)	0.9800	C(19)-H(19A)	0.9800	C(19)-
H(19B)	0.9800	C(19)-H(19C)	0.9800	C(20)-
H(20A)	0.9800	C(20)-H(20B)	0.9800	C(20)-
H(20C)	0.9800	O(5)-H(5A)	0.8700	O(5)-
H(5B)	0.8700			
O(1)-Os(1)-N(1)	178.25(12)	O(1)-Os(1)-N(2)	99.12(12)	O(2)-
Os(1)-O(1)	83.64(10)	O(2)-Os(1)-N(1)	96.46(12)	O(2)-
Os(1)-N(2)	177.24(12)	O(3)-Os(1)-O(1)	96.78(11)	O(3)-
Os(1)-O(2)	97.01(12)	O(3)-Os(1)-O(4)	162.96(12)	O(3)-
Os(1)-N(1)	84.95(12)	O(3)-Os(1)-N(2)	82.49(12)	O(4)-
Os(1)-O(1)	96.33(11)	O(4)-Os(1)-O(2)	95.22(12)	O(4)-
Os(1)-N(1)	81.92(12)	O(4)-Os(1)-N(2)	84.72(12)	N(1)-
Os(1)-N(2)	80.79(10)	C(1)-O(1)-Os(1)	114.2(2)	C(2)-
O(2)-Os(1)	109.4(2)	C(15)-N(1)-Os(1)	106.3(2)	C(17)-

N(1)-Os(1)	111.8(2)	C(17)-N(1)-C(15)	108.1(3)	C(18)-
N(1)-Os(1)	113.4(2)	C(18)-N(1)-C(15)	109.9(3)	C(18)-
N(1)-C(17)	107.2(3)	C(16)-N(2)-Os(1)	105.6(2)	C(19)-
N(2)-Os(1)	112.4(2)	C(19)-N(2)-C(16)	109.6(3)	C(19)-
N(2)-C(20)	107.8(3)	C(20)-N(2)-Os(1)	113.6(2)	C(20)-
N(2)-C(16)	107.7(3)	O(1)-C(1)-C(2)	106.1(3)	O(1)-
C(1)-C(6)	108.0(3)	O(1)-C(1)-C(7)	106.3(3)	C(2)-
C(1)-C(6)	112.4(3)	C(7)-C(1)-C(2)	108.0(4)	C(7)-
C(1)-C(6)	115.4(4)	O(2)-C(2)-C(1)	109.5(3)	O(2)-
C(2)-H(2)	108.3	O(2)-C(2)-C(3)	107.0(4)	C(1)-
C(2)-H(2)	108.3	C(1)-C(2)-C(3)	115.1(4)	C(3)-
C(2)-H(2)	108.3	C(2)-C(3)-H(3A)	109.4	C(2)-
C(3)-H(3B)	109.4	H(3A)-C(3)-H(3B)	108.0	C(4)-
C(3)-C(2)	111.0(5)	C(4)-C(3)-H(3A)	109.4	C(4)-
C(3)-H(3B)	109.4	C(3)-C(4)-H(4A)	108.8	C(3)-
C(4)-H(4B)	108.8	C(3)-C(4)-C(5)	113.8(4)	H(4A)-
C(4)-H(4B)	107.7	C(5)-C(4)-H(4A)	108.8	C(5)-
C(4)-H(4B)	108.8	C(4)-C(5)-C(6)	109.4(3)	C(4)-
C(5)-C(9)	106.6(4)	C(8)-C(5)-C(4)	109.6(4)	C(8)-
C(5)-C(6)	115.1(4)	C(8)-C(5)-C(9)	106.4(4)	C(9)-
C(5)-C(6)	109.4(4)	C(1)-C(6)-H(6)	105(2)	C(5)-
C(6)-C(1)	113.5(3)	C(5)-C(6)-H(6)	98(3)	C(10)-
C(6)-C(1)	113.3(3)	C(10)-C(6)-C(5)	112.9(3)	C(10)-
C(6)-H(6)	112(2)	C(1)-C(7)-H(7A)	109.5	C(1)-
C(7)-H(7B)	109.5	C(1)-C(7)-H(7C)	109.5	H(7A)-
C(7)-H(7B)	109.5	H(7A)-C(7)-H(7C)	109.5	H(7B)-
C(7)-H(7C)	109.5	C(5)-C(8)-H(8A)	109.5	C(5)-
C(8)-H(8B)	109.5	C(5)-C(8)-H(8C)	109.5	H(8A)-
C(8)-H(8B)	109.5	H(8A)-C(8)-H(8C)	109.5	H(8B)-
C(8)-H(8C)	109.5	C(5)-C(9)-H(9A)	109.5	C(5)-
C(9)-H(9B)	109.5	C(5)-C(9)-H(9C)	109.5	H(9A)-
C(9)-H(9B)	109.5	H(9A)-C(9)-H(9C)	109.5	H(9B)-
C(9)-H(9C)	109.5	C(6)-C(10)-H(10A)	106.9	C(6)-
C(10)-H(10B)	106.9	H(10A)-C(10)-H(10B)	106.7	C(11)-
C(10)-C(6)	121.5(3)	C(11)-C(10)-H(10A)	106.9	C(11)-
C(10)-H(10B)	106.9	C(12)-C(11)-C(10)	112.5(4)	C(13)-
C(11)-C(10)	111.4(4)	C(13)-C(11)-C(12)	107.0(4)	C(13)-
C(11)-C(14)	110.2(4)	C(14)-C(11)-C(10)	107.9(4)	C(14)-
C(11)-C(12)	107.9(4)	C(11)-C(12)-H(12A)	109.5	C(11)-
C(12)-H(12B)	109.5	C(11)-C(12)-H(12C)	109.5	H(12A)-
C(12)-H(12B)	109.5	H(12A)-C(12)-H(12C)	109.5	H(12B)-
C(12)-H(12C)	109.5	C(11)-C(13)-H(13A)	109.5	C(11)-
C(13)-H(13B)	109.5	C(11)-C(13)-H(13C)	109.5	H(13A)-
C(13)-H(13B)	109.5	H(13A)-C(13)-H(13C)	109.5	H(13B)-
C(13)-H(13C)	109.5	C(11)-C(14)-H(14A)	109.5	C(11)-
C(14)-H(14B)	109.5	C(11)-C(14)-H(14C)	109.5	H(14A)-
C(14)-H(14B)	109.5	H(14A)-C(14)-H(14C)	109.5	H(14B)-
C(14)-H(14C)	109.5	N(1)-C(15)-H(15A)	109.7	N(1)-
C(15)-H(15B)	109.7	H(15A)-C(15)-H(15B)	108.2	C(16)-
C(15)-N(1)	109.9(3)	C(16)-C(15)-H(15A)	109.7	C(16)-

C(15)-H(15B)	109.7	N(2)-C(16)-C(15)	110.8(3)	N(2)-
C(16)-H(16A)	109.5	N(2)-C(16)-H(16B)	109.5	C(15)-
C(16)-H(16A)	109.5	C(15)-C(16)-H(16B)	109.5	H(16A)-
C(16)-H(16B)	108.1	N(1)-C(17)-H(17A)	109.5	N(1)-
C(17)-H(17B)	109.5	N(1)-C(17)-H(17C)	109.5	H(17A)-
C(17)-H(17B)	109.5	H(17A)-C(17)-H(17C)	109.5	H(17B)-
C(17)-H(17C)	109.5	N(1)-C(18)-H(18A)	109.5	N(1)-
C(18)-H(18B)	109.5	N(1)-C(18)-H(18C)	109.5	H(18A)-
C(18)-H(18B)	109.5	H(18A)-C(18)-H(18C)	109.5	H(18B)-
C(18)-H(18C)	109.5	N(2)-C(19)-H(19A)	109.5	N(2)-
C(19)-H(19B)	109.5	N(2)-C(19)-H(19C)	109.5	H(19A)-
C(19)-H(19B)	109.5	H(19A)-C(19)-H(19C)	109.5	H(19B)-
C(19)-H(19C)	109.5	N(2)-C(20)-H(20A)	109.5	N(2)-
C(20)-H(20B)	109.5	N(2)-C(20)-H(20C)	109.5	H(20A)-
C(20)-H(20B)	109.5	H(20A)-C(20)-H(20C)	109.5	H(20B)-
C(20)-H(20C)	109.5	H(5A)-O(5)-H(5B)	104.5	

13. Computational Studies

13.1 Method

Possible TS conformations were explored by the artificial force induced reaction (AFIR) method [23] implemented in the global route reaction mapping (GRRM) program.[24] An extensive conformational search has been performed on possible catalyst substrate orientations at AIMNet2 (model: aimnet2_wb97m_0.jpt)[25] via Orca 6.1.1,[26] using SC-AFIR with constraint. The obtained approximately 1000 structures were sorted by energy. The energies of the lowest 100 structures were re-evaluated at g-xTB[27]. The lowest ten geometries were then further optimized at r^2SCAN-3c theory,[28,29] and the most stable conformers were further optimized at their transition states. All geometries were optimized at the r^2SCAN-3c level of theory using Orca 6.1.1 program. Thermal free energy corrections have been performed at the same level of theory, and the temperature was set at 223.15K. Transition state structures were verified by the corresponding intrinsic reaction coordinates. Solvation effect has been accounted by using SMD (toluene) solvation model. All single point energy is calculated at SMD(Toluene)-ωB97M-V/def2-TZVPP level of theory[30,31]. RI approximation was used with RIJCOSX with def2/J as an auxiliary basis set. IGMH analysis was performed using Multiwfn.[32] The visualizations of the molecular geometries were generated using the ChimeraX[33] version 1.9 followed by rendering with Blender version 4.0.[34] Conversion of enantiomeric ratio and ΔΔG was performed based on the Boltzmann distribution as follows: $\Delta\Delta G = RT\ln(\text{pdt}(R)/\text{pdt}(S))$.[30] IGMH analysis[31] was conducted with Multiwfn 3.8(dev) using the default parameters with isovalue of 0.004 a.u..[35,36] The KIE calculation was performed with PyQuiver program[20] using I and TS1.

13.2 Results and Discussion

The DFT calculation is performed using substrate **1a**, catalyst **5d**, and two explicit molecules of PFTB. The calculated energy diagram is shown in Figure S22. Intermediates **I**, **II**, and **III** correspond to the pre-reaction complex, carbocation, and product, respectively, connected by the IRC pathway from each transition state. The calculated results at SMD($CHCl_3$)-ωB97M-V/def2-TZVPP//r^2SCAN-3c level of theory suggest that protonation **TS1** is the rate- and enantio-determining step, regardless of the enantiomers. Here, all transition states possess a few negative vibrational modes; however, each transition state was confirmed by a single distinct imaginary frequency corresponding to the reaction coordinate, as well as by IRC analysis. The additional low-magnitude negative frequencies (all > -10 cm^{-1}) are likely numerical artifacts and do not correspond to meaningful reaction coordinates. The calculated energy difference between **TS1** and **TS1′** agreed well with the experimental one (1.4 kcal/mol (calc) vs. 1.3 kcal/mol (exp)).

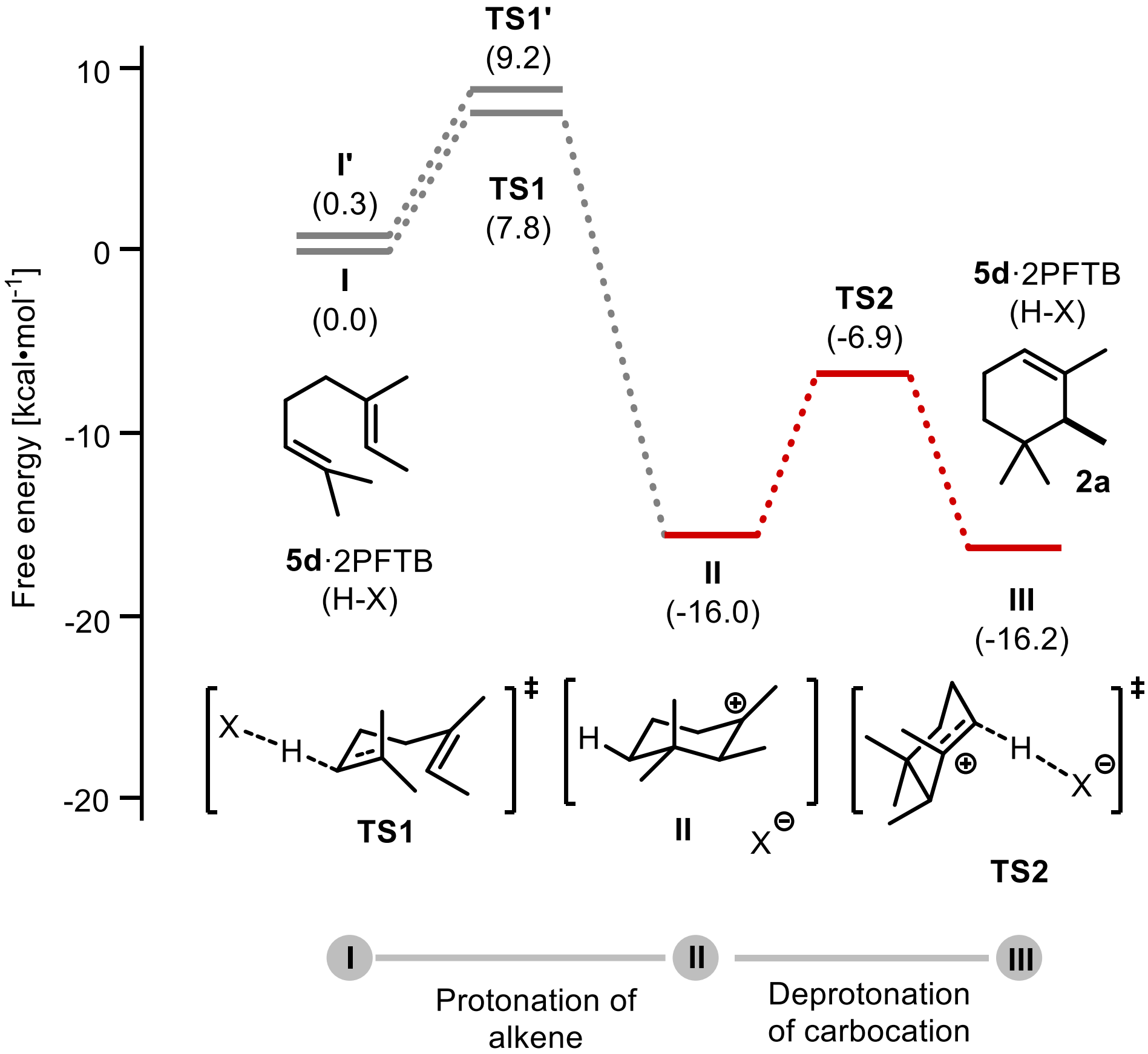

Figure S23. The calculated energy diagram of the proposed catalytic cycle.

Additionally, **TS1** and **TS1′** were compared to gain further insight into the reaction mechanism (Figure S24A). In both transition-state structures, the proton is located between the nitrogen and carbon atoms, whereas the forming C–C bond distances are greater than 3 Å, indicating that C–C bond formation has not yet occurred. Furthermore, the C–H and C–C bond distances were plotted together with the electronic energy along the IRC coordinates to visualize the asynchronicity of the process (Figure S24B). At **TS1**, the C–H bond is being formed, whereas the C–C bond remains largely unchanged; C–C bond formation instead begins afterward, as reflected by a shoulder in the energy profile. These results indicate that both **TS1** and **TS1′** are highly asynchronous transition states in which olefin protonation precedes C–C bond formation.

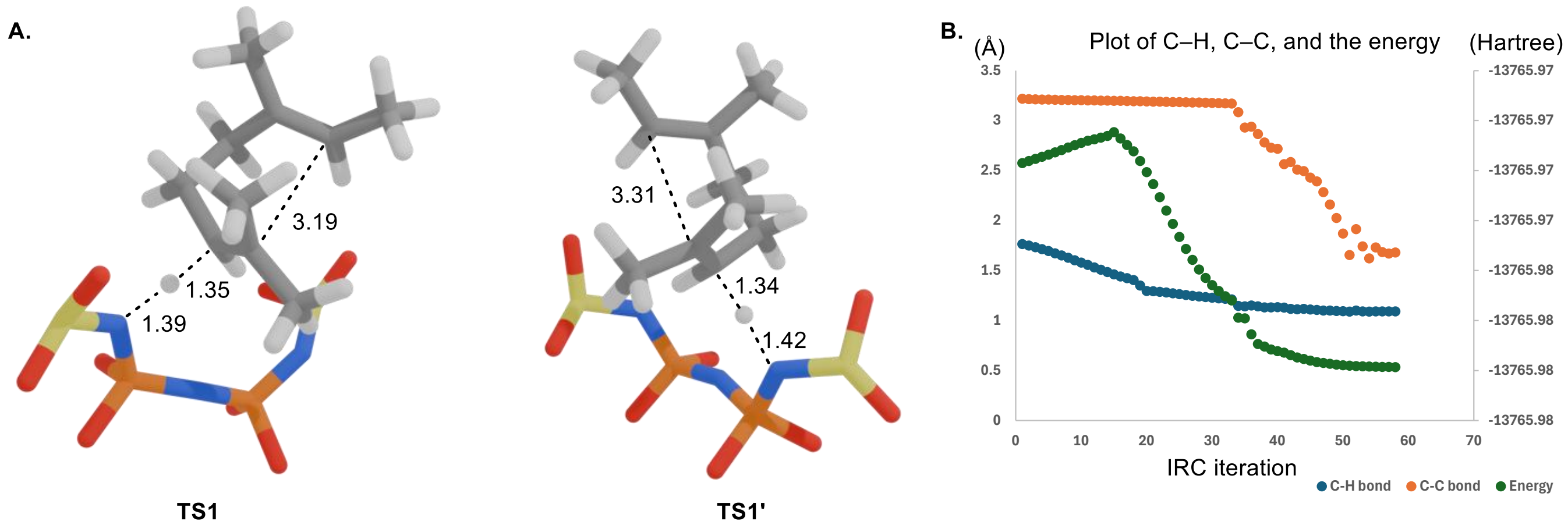

Figure S24. (A) Structural comparison between **TS1** and **TS1′**. For clarity, only the core of the catalytically active site and the substrate are shown. The numbers indicate the corresponding bond distances in Å. **(B)** Plot of the C–H and C–C bond distances and electronic energy along the IRC coordinates of **TS1**.

For a better understanding of the origin of enantioselectivity, distortion-interaction analysis was conducted following

the Houk-Bickelhaupt protocol[37] (Supplementary Table S14). Gas-phase single point energies of the optimized $\mathbf{TS_{maj}}$ and $\mathbf{TS_{min}}$, leading to the major enantiomer and minor enantiomer, respectively, were calculated at ωB97M-V/def2-TZVPP level of theory. Then they were decomposed into the catalyst fragment and the substrate fragment. The result suggests that the interactions between the catalyst and the substrates would be the major factor in controlling the enantioselectivity.

Table S14. Summary of the distortion-interaction analysis of **TS1** and **TS1′**.

TS	ωB97M-V/def2-TZVPP (in hartree)	Relative energy ΔΔE (kcal/mol)
**TS1**	-13768.87142	-1.64
**TS1′**	-13768.86881	
Substrate fragment		
subst- **TS1**	-392.1134327	1.39
subst- **TS1′**	-392.1156464	
Catalyst fragment		
cat- **TS1**	-13376.57018	-1.25
cat- **TS1′**	-13376.5682	
Total distortion		0.143
Total interaction		-1.78

Table S15. Energy table of the optimized structures. Energies are given in Hartree. Computed single point energies (E), Gibbs free energy corrections (Gcorr), Gibbs free energies (G), and negative imaginary frequencies for transition states are provided. Small imaginary frequencies are associated with remote, low-amplitude floppy motions of peripheral groups and are not involved in the bond-making/bond-breaking process.

Structures	E (solv) in Hartree	Gcorr in Hartree	G (solv) in Hartree	Imaginary frequency
**I**	-13768.97349	2.57989822	-13766.3936	-
**I′**	-13768.97355	2.58044937	-13766.39311	-
**TS1**	-13768.95741	2.57624326	-13766.38116	-981.49, -9.16
**TS1′**	-13768.95591	2.57697132	-13766.37894	-850.23, -4.65, -2.34
**II**	-13769.00334	2.58423049	-13766.4191	-
**TS2**	-13768.98777	2.58320456	-13766.40456	-978.23, -5.36, -3.99
**III**	-13769.00661	2.58714492	-13766.41947	-

13.3 Cartesian coordinates of the optimized structure

I

O -0.47083963898250 -1.50809034550692 -1.73619500548866

O -2.69462464862243 -1.20453256108380 -0.47067923098532

O -0.95613372825268 2.83380713873281 -0.07123203539704

O 1.10013892238327 1.51467289734318 -0.72646649335825

P	-0.10713924185049	1.54546879234074	0.32219011273982
P	-1.09919101182973	-1.24978958195318	-0.26252654550994
S	0.34533957675333	3.37408153773867	2.75756454623028
S	0.50840350990380	-3.48166050548528	0.51639112924913
O	-0.43674539700461	4.41060837417906	2.10575925192509
O	0.11767076455986	2.93575918854129	4.13789458787434
O	1.62670160183221	-2.98980692293233	-0.29958093631033
O	0.80473582515980	-4.07895927703866	1.83673899276272
N	-0.71968846139713	-2.48026944194792	0.67733657863547
N	0.41654253721385	1.94616968172218	1.86680702093179
N	-0.80390633960592	0.17641131311094	0.36716711841168
H	0.54303518437246	1.11222038192520	2.50949717103029
C	-3.23128589380328	-2.33349309420589	-1.10157968883962
C	-1.05590710604316	-1.02088537832242	-2.90050694276213
C	-1.14440774808090	3.17220404303762	-1.41913424573395
C	1.87898325921051	2.66371702399417	-0.90899767531925
C	-3.89371698240599	-3.29630716279502	-0.30066589563618
C	3.24543828448241	2.59512503343671	-0.52191389613637
C	-0.22953620620570	-0.24687588385273	-3.76257105750453
C	-2.45406400940883	3.03265266078771	-1.94598555146398
C	-3.65342028797790	-5.09490705482028	-5.03864613364614
C	-3.24949010311810	-3.91379630530332	-4.46782477532937
C	-3.48019668459358	-3.64458450297062	-3.10003147711763
C	-4.11167967394984	-4.65404310245856	-2.30965222269265

C	-4.52717724607925	-5.85693274457154	-2.92288324267953
C	-4.31068156849929	-6.07620964537373	-4.26495883553495
H	-3.46073102808112	-5.27692557182149	-6.08868434654970
H	-2.73536782137501	-3.17887774666544	-5.07752027165069
C	-3.05392964766910	-2.44013075107895	-2.46032595372090
C	-4.31825374124002	-4.44482114434715	-0.92611331166684
H	-5.01382737752330	-6.60084408869591	-2.30250989925159
H	-4.82990524540690	-5.20667104029069	-0.34322179173767
C	-4.21746701805318	0.81449310624904	-6.33371893301534
C	-2.87429265485650	0.97664679717649	-6.08572713206077
C	-2.23279915213373	0.24836687931881	-5.05891551402130
C	-2.98787755500020	-0.68823991067780	-4.29184587355165
C	-4.36247752494927	-0.84634311686368	-4.58431570276650
C	-4.96954415948262	-0.11161923739755	-5.57126432388391
H	-0.25318574423021	1.00183057159964	-5.49274213124683
H	-2.28643178466133	1.67644424208694	-6.66821011755179
C	-0.84924309275769	0.39700711897868	-4.81270875431243
C	-2.35532030500882	-1.37159953047587	-3.21224743726815
H	-4.95180352022629	-1.54425167630489	-3.99843674892168
H	-6.02838697025360	-0.24551421438958	-5.75435942171518
C	0.56653938636927	4.66683155630305	-5.68993415547256
C	0.79987411546421	4.34269155332774	-4.37781620685953
C	-0.25491469542181	3.98094735939376	-3.50564152802151
C	-1.57009393267730	3.89687622683794	-4.05192686986760

C	-1.79307718506462	4.26655173790297	-5.39989323350765
C	-0.75122068337041	4.65543439293832	-6.20625184083822
H	1.40479557821172	4.93206542753629	-6.32175868775090
H	1.81956664902061	4.35972502843098	-4.01137336624702
C	-0.05942593580276	3.65113825713189	-2.12554395459194
C	-2.63483859252916	3.43040371339796	-3.25630846437837
H	-2.80518070345137	4.22649037378884	-5.78647754148147
H	-3.62878227151597	3.38055754444701	-3.69226221284881
C	3.63394918339563	7.28201331649505	-1.79440503170604
C	4.20157095065062	6.10378159889067	-1.37370305285552
C	3.42280453376790	4.92995104548867	-1.24189761124103
C	2.04223963973638	4.96564872480814	-1.59402865540312
C	1.48103113654228	6.19950295794477	-1.99326192178773
C	2.24925396504973	7.33213924125996	-2.08780403654390
H	5.06077048180590	3.71949186645629	-0.56069298154358
H	5.25538647621109	6.05868818686348	-1.12273357988148
H	0.42125573793053	6.25495863890916	-2.21793668488526
H	1.78259965142156	8.26264418348782	-2.38515190395088
C	3.99382988372320	3.73520231370147	-0.76224160137032
C	1.28614092637122	3.75740238421962	-1.51232547565092
C	-3.58268407498468	2.47624394543843	-1.17342087550848
C	-3.83215967810598	2.84877093390442	0.15987539709810
C	-4.45271444250903	1.58490737241138	-1.81644192995716
C	-4.93170213661345	2.33259234098435	0.82711695159077

H	-3.16946417149057	3.55588080274916	0.64055647957222
C	-5.55608539140843	1.06252414908971	-1.15793940880000
H	-4.24535743659131	1.28184940282673	-2.83826738482239
C	-5.79567075659880	1.43829813586220	0.16051907428209
H	-6.21627591616765	0.36745947552780	-1.67094497891330
C	-6.65030745338078	1.68139335559114	2.29849186291731
C	-6.85070685477814	1.03192718226441	1.07726465770701
C	-7.47840090947498	1.42541195485721	3.38254607140316
C	-7.89542926342694	0.11973012432671	0.93689640837187
C	-8.51684792408320	0.49453587363963	3.27023093781597
H	-7.29920109082238	1.93320314479377	4.32494422345612
C	-8.70771400051288	-0.14143633951817	2.03061383929085
H	-8.06410558124103	-0.40144575728120	-0.00209455107218
H	-9.50447721156969	-0.87109443534292	1.92201725846360
C	-5.41172608914720	2.55926594056645	2.25951951714926
C	-5.76511927542657	4.03381640139522	2.57136126442187
C	-4.40576312687794	2.03243958783509	3.31253270513324
C	-4.54439010510611	4.93334646355726	2.77769514385190
H	-6.35951841626573	4.03808309637686	3.49627906740608
H	-6.41194896318617	4.42370197167544	1.77601476443703
C	-3.19046502231133	2.92840294988780	3.50562312649527
H	-4.93935003447147	1.96241819714539	4.27093255748550
H	-4.10277311744493	1.01419144387611	3.04096137922604
C	-3.59712080095362	4.36417461830919	3.83607749238614

H	-4.88479299412972	5.93234148282933	3.07688628556574
H	-4.00636878663376	5.06883599645738	1.83154028676525
H	-2.57898338885928	2.51376875937711	4.31406703023750
H	-2.56941532023068	2.91107444964490	2.60113186499447
H	-2.70944590693502	5.00134035690051	3.92924130216382
H	-4.09626649659980	4.37311067269472	4.81634316699560
C	3.87935769146276	1.40047373952543	0.06944451371521
C	4.97312707079507	1.58846959074781	0.94392511957409
C	3.49411958036688	0.09138078206189	-0.26230665468886
C	5.69143529843189	0.50662845868597	1.41842549087566
H	5.22212579051009	2.59622079815554	1.25220806617691
C	4.20954401537804	-0.99840578239626	0.20930063513222
H	2.66437732404934	-0.09329761571862	-0.93030074300445
C	5.31743702769568	-0.79174591307533	1.01767227759856
C	6.89712972271005	0.44014661524143	2.35599548465084
H	3.89464119534716	-1.99938975165095	-0.06039384649518
C	6.23272257039976	-1.75162906540716	1.60521549327078
C	7.15487447782959	-1.06078458158339	2.39575119554231
C	6.58452893633242	0.96358048884870	3.78024202957802
C	8.13858529270593	1.16941645819915	1.78468044442660
C	6.30704689098182	-3.13666713798421	1.48103424298785
C	8.15016609145040	-1.74648032277770	3.07753628752528
C	6.51843636349137	2.48751866455751	3.87604978049676
H	7.38228625061339	0.60608777387165	4.44695084427874

H	5.65047832009563	0.50740987027940	4.13175441347834
C	8.07479752681756	2.69248614784111	1.89880287860506
H	9.01800025311879	0.81484905643018	2.34113166280559
H	8.27513120373650	0.85861428784609	0.74234890355940
C	7.30734945183619	-3.81202802866743	2.16291594438841
H	5.59695911341321	-3.68110539434354	0.86473299126238
C	8.24263768769784	-3.13984874156539	2.96960889594593
H	8.86020960667606	-1.19869948578547	3.68920605478353
C	7.79628527700151	3.13287544478678	3.33691595250361
H	6.36014055922951	2.77635489483485	4.92260429320906
H	5.64895724327003	2.86196151845877	3.32186998326340
H	9.02366699868013	3.11851075403192	1.54944435721206
H	7.29948270791924	3.09057907789543	1.23091597716220
H	7.35877245877016	-4.89279991404969	2.06936064688557
H	7.72691844041829	4.22598692098162	3.39586904017204
H	8.64339342420454	2.83515151418526	3.97282736231666
C	-4.13257333927479	-3.02453758841336	1.13284704773865
C	-3.56850239484663	-3.83154341188703	2.13207175002188
C	-4.91681599202596	-1.91946633636834	1.47437682909065
C	-3.72719220533317	-3.47653914632636	3.46447459917531
H	-2.96074769438109	-4.67773553214676	1.83610417264490
C	-5.12198468758575	-1.58265969568406	2.80343651761868
H	-5.35840190303335	-1.31443190846725	0.68848198214193
C	-4.48675126505102	-2.33329476968047	3.78634697693963

C	-3.16156319018856	-4.10324312491049	4.73892263418084
H	-5.74398842191393	-0.73050002682026	3.06087523816756
C	-4.36324533777626	-2.07089787846993	5.21085966812533
C	-3.53098946467346	-3.04502945676695	5.77070264562667
C	-1.64058715534151	-4.37529358647770	4.71739142807035
C	-3.91989561616510	-5.41243151732109	5.10083613400906
C	-4.82130433789447	-1.00875580958711	5.98606257174764
C	-3.13521515494796	-2.95425411286741	7.09775207482529
C	-1.21915971764513	-5.61401718683690	3.92552649703700
H	-1.32392407158665	-4.51828910671545	5.75949282217225
H	-1.11430880476670	-3.48872671061922	4.34426091971081
C	-3.51347006956260	-6.60435701087556	4.23796137769290
H	-3.69251631975774	-5.64469739437723	6.15144992897101
H	-4.99893529631241	-5.22389530395436	5.04106740981103
C	-4.41131621042008	-0.92128645902789	7.30919220765148
H	-5.46515912464646	-0.24380332447134	5.55736549272224
C	-3.55351388986089	-1.87389319511878	7.88570427177809
H	-2.47900505871772	-3.71152259630977	7.51505724618097
C	-2.00844095859888	-6.85472991485457	4.33829054209988
H	-0.14602520654820	-5.78105282733038	4.07765332060813
H	-1.33750888043671	-5.43862405355334	2.85143041018977
H	-4.07240511536349	-7.49152607848377	4.56135007156336
H	-3.79527477769906	-6.42410815085500	3.19136920534029
H	-4.75101072224150	-0.07806947241029	7.90369318523463

H	-1.71528925108434	-7.70985400399147	3.71750536652465
H	-1.76017234477829	-7.11745230402249	5.37784261175994
C	1.24151425881578	-0.24444001197851	-3.61524059407710
C	1.92216964693537	-1.47270932216544	-3.50423802713565
C	1.97125175212254	0.94401909687145	-3.68898757116877
C	3.30377296928278	-1.49348365975547	-3.43107090156953
H	1.34613802267923	-2.39036016084293	-3.48635495232753
C	3.35523105587841	0.94015299490776	-3.55253680126077
H	1.43948483474780	1.88239070157022	-3.80623863314022
C	4.01506734376050	-0.27309847232122	-3.38726495593566
C	4.28755271955509	-2.66332633548146	-3.40052821224387
H	3.90359145667331	1.87890745483204	-3.53865476316876
C	5.40818863656827	-0.55916765865404	-3.07327373545424
C	5.56591472861820	-1.94746403094320	-2.99340864365772
C	4.49381017622582	-3.18954240501459	-4.85502599331628
C	3.95068528369918	-3.85214977188972	-2.48206710227073
C	6.47635483764772	0.28006726294999	-2.76761916937104
C	6.77563791030780	-2.50168595719837	-2.60655881836374
C	3.32508956053234	-4.03271132547714	-5.35642183054199
H	5.40560624686953	-3.80418214183584	-4.85386671508840
H	4.68280360367883	-2.33563261665489	-5.51690045226906
C	2.83209184728633	-4.76164219988864	-2.99366227372447
H	4.86331088053228	-4.45856073415309	-2.39128449348079
H	3.70658352692353	-3.49182310427868	-1.48079990585667

C	7.68341782546187	-0.28508461554018	-2.37465977884376
H	6.36877515618219	1.36136907501934	-2.80993674723307
C	7.85696273064795	-1.67602045140410	-2.27473319749004
H	6.87439965875934	-3.57971578256570	-2.53960571675403
C	3.07819238603191	-5.22344464124126	-4.42953810934063
H	3.53858762178212	-4.37841481530273	-6.37562503326359
H	2.41945173633727	-3.41353355041462	-5.42108454798393
H	2.75380087903575	-5.62746570413550	-2.32619705235510
H	1.86602177091263	-4.24876392279617	-2.92586769156281
H	8.51067004266806	0.37602990560682	-2.13267850397070
H	2.22942632660632	-5.81966313017811	-4.78624294069211
H	3.95917042000778	-5.88250959252435	-4.45228253172213
C	2.14372322788753	4.00060616511479	2.78635872560507
F	2.98154704773422	2.97613262481266	2.93568431563843
F	2.41516155101541	4.65591122252036	1.66198172351094
F	2.25543050578454	4.82916803365399	3.82222929442525
C	-0.22283590986820	-4.93095797436887	-0.47603487215734
F	-1.26867944301576	-5.46513779734697	0.16796396786802
F	-0.63163741010783	-4.51020171055628	-1.68175113463170
F	0.71051282069672	-5.87302875793028	-0.63570318958282
C	4.51035558422523	8.51177773895840	-1.92523329223140
F	5.82221333299994	8.19881449993819	-1.62117798565676
C	4.55549777886305	9.03095749416588	-3.39275500138603
F	5.46868700554429	9.99755768860425	-3.54810054549573

F	4.88579914198311	8.01271925962963	-4.20558617152658
F	3.36171088325635	9.51234516852548	-3.78474638348956
C	4.11510120246874	9.61387201730164	-0.89935494059328
F	4.94908612004719	10.66137698010841	-0.95046738777101
F	2.86487592968223	10.06228382575044	-1.11173202181549
F	4.17211842949379	9.09557820748088	0.33838787396889
C	-1.03859387226457	5.05629537266465	-7.63919585551866
F	-2.39173219831932	4.96882552207042	-7.89775769331862
C	-0.67708573258900	6.54892555539704	-7.89959236182412
C	-0.37085997981307	4.08453626025948	-8.65596006372303
F	-1.04970726326584	6.94032368402048	-9.12481185517810
F	-1.31930394897258	7.31467761332505	-7.00242677294095
F	0.64426301913774	6.76864495898205	-7.77132072189181
F	-0.75956134262258	4.33361996521576	-9.91073745299938
F	0.97127000902495	4.16112214833144	-8.60546422183985
F	-0.72726245726208	2.82074476928718	-8.35346539944726
C	-4.86726183117552	1.64833519678161	-7.41884268412111
F	-3.92438650689343	2.44607523195587	-8.03819286390489
C	-5.45763365984915	0.76777374411032	-8.55955884947668
C	-5.90426729860356	2.64719205222996	-6.82671018684302
F	-5.85594307079805	1.51143960311173	-9.59963234767638
F	-4.51210144537976	-0.08111433554386	-8.99225147715753
F	-6.51046566708972	0.04539205712781	-8.13228440370253
F	-6.40194907910209	3.46448604607110	-7.76137789766820

F	-6.92696869429617	2.01118548572617	-6.23141340671786
F	-5.29126650965460	3.40944101734856	-5.89580664164799
C	-4.75339927371597	-7.35964438941092	-4.94200120917670
F	-4.41826579198154	-7.33252053217825	-6.28333739486658
C	-4.00860016577049	-8.60168302934679	-4.37200997067988
C	-6.30191171534065	-7.51379502195725	-4.92372970944831
F	-4.29693156469402	-9.71199594641992	-5.06502583941176
F	-2.68479855453142	-8.39196986884565	-4.45312679600760
F	-4.32343469687937	-8.81974137382034	-3.08197383224744
F	-6.70391160435648	-8.56660804139692	-5.64833195925637
F	-6.77121149606940	-7.66297453761249	-3.67119514242420
F	-6.85672080004212	-6.40954179592616	-5.45159639089747
C	0.35942553381936	0.22551510488683	5.67314039075720
H	-0.44306103226058	0.91232667312062	5.96280384224695
H	1.31798736525373	0.73485231999114	5.78971193700731
H	0.33517949530106	-0.61058855584907	6.38567170823624
C	0.13013379745788	-0.31543198086726	4.29314147868280
C	1.10625922008753	-0.54757757322376	3.39443198290224
H	0.81150128039589	-1.05692514074088	2.47313693214991
C	-1.30323617509729	-0.62883049048476	3.98007151623041
H	-1.75836180010075	-1.18960107851249	4.80520826857280
H	-1.88810356239147	0.29769467378804	3.89239198081724
H	-1.41773835364698	-1.20510410297702	3.05810438229687
C	2.58360792252545	-0.36978014402629	3.57050582122993

H	3.01803777497245	0.03983365709835	2.64836683091300
H	2.81242130421575	0.33971108548012	4.37417159136580
C	3.26474637309188	-1.73327433460782	3.85719069171892
H	4.35165930847233	-1.61758057530053	3.74667589786584
H	2.93873558425984	-2.44622701355371	3.09118921894088
C	2.93964855516570	-2.25027374963408	5.23489794642666
C	1.95898376812812	-3.14338357610324	5.40752709069656
H	1.44761434154603	-3.49357870231737	4.50934710129258
C	3.76466135517099	-1.64978162455525	6.33881502536781
H	4.81904272035381	-1.93397665633732	6.22016246429087
H	3.73388332249365	-0.55273291215246	6.29651614973132
H	3.44110869606464	-1.95004952739226	7.33691178276395
C	1.45289479136585	-3.71910220484095	6.69260325677473
H	0.44200402184601	-3.34469037611590	6.91036803744667
H	1.37123508179671	-4.81066845174972	6.62148442074556
H	2.08723518450378	-3.48528693628309	7.55072751291168
O	-1.44325627548376	3.49028352608039	6.44335158251381
H	-0.85648522952795	3.44897068299231	5.66427967722058
C	-0.92526122867964	4.35488684778818	7.39405889174886
C	-1.95861789962992	4.36497975395750	8.56639913282985
C	0.46566650969782	3.83990885887404	7.90398756743321
C	-0.78032467382998	5.79701275739576	6.79263623872129
F	-1.97998590654667	6.38430232946896	6.66650819230914
F	-0.24136211945961	5.71659027365772	5.55852097249015

F	0.00363984991641	6.58965919169999	7.53796289798358
F	0.85569042731753	4.44725265916138	9.03500227466727
F	0.42631264504777	2.52029567141264	8.12529587569477
F	1.40575817901001	4.06086782357174	6.95925933433117
F	-1.73743773115013	5.40207975786661	9.39306572191370
F	-3.20764376939678	4.45734250169411	8.09651044717247
F	-1.87513967153140	3.23253383666965	9.28480902889509
O	1.80831858209187	-6.61586678163114	2.29409882487920
H	1.63927671176461	-5.66445460861747	2.11101091745378
C	3.15798632688782	-6.89969922857378	2.37443181344919
C	3.25146528107107	-8.43706279744728	2.64320385223218
C	3.87682198778059	-6.54986579616572	1.02561501842292
C	3.82730162453427	-6.10896313325004	3.55430462118791
F	3.03968620763930	-6.12903343859056	4.63536319778968
F	4.00517640518980	-4.82278600612340	3.19833187919840
F	5.02666424715822	-6.61544585080043	3.89828955419756
F	3.46932055771526	-5.34186121474737	0.59296803304091
F	5.21811696836936	-6.51246881435946	1.14728923211186
F	3.57133694766834	-7.44196412251532	0.07035960710633
F	4.49793048497767	-8.89423694939904	2.41631286326618
F	2.93265118528261	-8.71555237547449	3.91857803986136
F	2.40850642100727	-9.11161114026005	1.85525442714083
C	9.32175254278800	-3.95032076477461	3.68966017219475
C	10.26078867425804	-3.06702298060602	4.51940493842713

H	10.79148157956182	-2.34084967501446	3.89357855258041
H	9.72104506786447	-2.52405546199993	5.30305184638800
H	11.01296639122932	-3.69431763945311	5.00883236345564
C	8.64900656864573	-4.96419969071314	4.63553575717179
H	8.02809259108299	-4.44883711478749	5.37622667089485
H	8.00744577049004	-5.66418707858796	4.09214095999893
H	9.40950470696285	-5.54771937785403	5.16739723700232
C	10.16955949971589	-4.70496986730163	2.64700135541223
H	9.56440749547772	-5.39876177369641	2.05593044534843
H	10.64729026454422	-4.00204654634214	1.95562478907498
H	10.95396595442111	-5.28513688312387	3.14678799922273
C	-3.08776501490438	-1.68221417530242	9.33097267449666
C	-2.13296833265606	-2.79053850277823	9.78980373614286
C	-2.35015652928570	-0.33345589192331	9.44815106410398
C	-4.31127981280638	-1.67981200756410	10.26774806188414
H	-2.60911926356088	-3.77685805055998	9.76188676379264
H	-1.22626927584607	-2.82457677670913	9.17513549583721
H	-1.82508571291035	-2.60093435033079	10.82335296792139
H	-1.48899001603776	-0.29948455494127	8.77142070092238
H	-3.00173836974293	0.50926667072415	9.20020402427472
H	-1.98930995536385	-0.18693714365051	10.47293850723901
H	-5.00765221224903	-0.87319799198286	10.01970171006497
H	-4.85428634669686	-2.62824569043289	10.19544746336055
H	-3.99285785542519	-1.54199753098658	11.30760789519033

C	9.20845154249706	-2.24705487459479	-1.83528310479370
C	10.24857246788300	-1.95546944389866	-2.93431956355446
C	9.66006728778783	-1.59086436573924	-0.51754193458518
C	9.15134785970091	-3.76224359092944	-1.60363035334860
H	9.95130652958652	-2.41723396772770	-3.88213405333869
H	10.35835245840020	-0.87947837128876	-3.10410458624060
H	11.22788521946423	-2.35641657073044	-2.64694647845184
H	8.91767517090177	-1.74574307427720	0.27263425785410
H	10.60877511532588	-2.03159949658461	-0.18934064229331
H	9.82224677618900	-0.51453404535798	-0.62928090362303
H	8.41067997987539	-4.01915980818845	-0.83770282215691
H	8.91537017463382	-4.31031794456846	-2.52222877175301
H	10.12834422255401	-4.11335903662823	-1.25479125197321
C	-9.40931182432254	0.11631427962874	4.45361972476566
C	-10.88705911758094	0.34787360837921	4.08587376461247
C	-9.18403088110304	-1.37277082131156	4.78448257222174
C	-9.09550359239027	0.93751646594755	5.71004029574210
H	-11.06216320808427	1.39858236337288	3.83084469058382
H	-11.19677766475192	-0.26199912348574	3.23218688193550
H	-11.53122893589478	0.08711359597279	4.93331182827401
H	-9.43953354467628	-2.01833474173292	3.93869157675285
H	-8.13473779482577	-1.55765557261246	5.04183530846198
H	-9.80511646196149	-1.66950900098398	5.63762388588772
H	-8.07170567301898	0.76835812347449	6.06193098701449

H	-9.23456602300678	2.01099076340449	5.54037211154438
H	-9.77155083125662	0.63764633539977	6.51719625773727

I′

O	0.74527713714065	-1.40860448876490	-1.87313864562068
O	-1.68441237898149	-1.78134549563222	-1.12716535671752
O	-1.57490866781144	2.34786998235625	-0.26890533260312
O	0.94499468533315	2.06567935854290	-0.44566647954872
P	-0.35292626484561	1.48134883045401	0.28364944062042
P	-0.21450272478125	-1.44710299106334	-0.56378754891357
S	-1.16272331570454	3.02268184120504	2.81530788943982
S	1.70529007274709	-3.19013440684043	0.63348522244907
O	-1.85848032552173	3.96462281538795	1.95474383236203
O	-1.80082468658218	2.32307293560160	3.93126012538122
O	2.79552989763706	-2.30304684106579	0.20307552749860
O	1.77909949725378	-3.80860691545154	1.97490957404786
N	0.23183961596896	-2.63132259334264	0.39850452784995
N	-0.33589332636978	1.85857254262886	1.92133284137247
N	-0.39422049306441	-0.04467213841683	0.14288661724232
H	-0.01451757798445	1.06230721083665	2.53765586232354
C	-1.76161309045025	-2.97652338273821	-1.85362144561772
C	0.28145895073306	-1.00173900981488	-3.12094002330782
C	-1.60260429955968	2.72847245472664	-1.62264644401017
C	1.17437444476379	3.44770323251052	-0.41497979488740

C	-2.30594966250421	-4.12548474450828	-1.22230830816349
C	2.30016425620429	3.92057452696393	0.31233610622077
C	0.97051291628891	0.06875644509179	-3.75505951591927
C	-2.61233308360013	2.15955305738489	-2.44255457884731
C	-0.55361922581401	-5.50731429127611	-5.79048382512419
C	-0.60193228657723	-4.30143076207433	-5.13610388144485
C	-1.20481008649200	-4.18257461953196	-3.86392452326736
C	-1.72773463888802	-5.36093189767556	-3.25081106707112
C	-1.67924666738905	-6.58817048053144	-3.95032875431684
C	-1.10971014986277	-6.66425576743453	-5.20134461523040
H	-0.08076278709156	-5.57211513923418	-6.76258284196665
H	-0.15953362738428	-3.42767191045815	-5.60184412633491
C	-1.26839067919901	-2.95426502978421	-3.13787121982803
C	-2.28442950904826	-5.29435082763170	-1.95576316192586
H	-2.09488954967302	-7.46453671417052	-3.46645732836950
H	-2.71451722488176	-6.19472092007887	-1.52482952363104
C	-2.36705903638030	-0.06155384248229	-7.29233438544606
C	-1.25208284395285	0.50229905838392	-6.71825842258806
C	-0.71016109869722	-0.01891717352783	-5.52099964680939
C	-1.30797224031425	-1.16772375660296	-4.92528362938422
C	-2.46484849714824	-1.71263943482286	-5.53039439516883
C	-2.98945610328257	-1.17505394065441	-6.67820098451155
H	0.95210623073673	1.35637184921597	-5.45532005552304
H	-0.76980335811878	1.35935388119023	-7.17403763748845

C	0.44270293186514	0.54692704614843	-4.93725793096924
C	-0.75640384092005	-1.69212306635233	-3.71889534968536
H	-2.95416072742755	-2.56507280172045	-5.07079847120266
H	-3.88147300702926	-1.61506018377741	-7.10633743164773
C	0.27596201357079	5.13660372323915	-5.37320258424087
C	0.31376923380762	4.81780700858866	-4.03989568977936
C	-0.67230736713603	3.99113886890324	-3.45037566353893
C	-1.67371639527470	3.43508360640640	-4.29948841850578
C	-1.71135521270151	3.80113318872105	-5.66577604772576
C	-0.76631383444818	4.64606916336379	-6.19563544726179
H	1.05177455661677	5.77052893870223	-5.78408656440099
H	1.12289000586743	5.20520713615680	-3.43118964876417
C	-0.67756917154806	3.65353284766620	-2.05993885080823
C	-2.62335713221027	2.53872662367729	-3.76987518443964
H	-2.50200993388166	3.39707890484808	-6.28757238271513
H	-3.39825803766828	2.14586102346012	-4.42333549325404
C	0.84583382010482	8.44496341953712	-0.76944128425082
C	1.75314505380319	7.57980761459441	-0.20789002654821
C	1.57537653161745	6.17924002033497	-0.30364900077547
C	0.45855951147927	5.66558321369530	-1.02451575912901
C	-0.46877271762090	6.58087764945554	-1.57191467695657
C	-0.28835891317367	7.93500374374125	-1.44771357602583
H	3.36749975984096	5.70961222648791	0.78089533945392
H	2.61549052442517	7.95812379115850	0.32954671355069

H	-1.34556797196604	6.20644302990704	-2.08923572494917
H	-1.02548754591476	8.60600099350756	-1.87015239134960
C	2.48805811113501	5.29267990872450	0.29924854484449
C	0.31941139770546	4.25103792946217	-1.14286925072561
C	-3.63853357089533	1.23871895655104	-1.91866601933970
C	-4.33066478994120	1.54431152813272	-0.73463450220293
C	-3.97452197510321	0.08896024577184	-2.64212657733303
C	-5.31940212021868	0.69378478052718	-0.27141006553198
H	-4.08301552813014	2.45943308672070	-0.21265486340925
C	-4.95744402171848	-0.77713999603394	-2.18188093381294
H	-3.43389646528359	-0.14524769824739	-3.55439417049823
C	-5.61527801296977	-0.48377320747676	-0.99021778539796
H	-5.18316333509637	-1.68608936413728	-2.73403254826810
C	-6.88588497188326	-0.56885131960138	0.94535465007270
C	-6.58410541449381	-1.26194925648069	-0.23184867415212
C	-7.71831242607852	-1.12985498988481	1.90375604750198
C	-7.13170856717014	-2.52203916948784	-0.45909593365136
C	-8.26122328978892	-2.40561719824991	1.70439758855283
H	-7.93403777331794	-0.58100953799007	2.81513515479673
C	-7.96009431618051	-3.07531605679372	0.50585318171860
H	-6.89925756269890	-3.08115212309782	-1.36173305075311
H	-8.37452420241010	-4.06289309345604	0.32739147512075
C	-6.20585163694546	0.79127477474003	0.96698934824136
C	-7.30246120603501	1.87881336344863	0.78394721107837

C	-5.44148941626959	1.04149408517209	2.28542093062132
C	-6.77294922127202	3.30436626796582	0.91349136853685
H	-8.06272278270687	1.71103716075426	1.56053876835530
H	-7.79549375115738	1.72309028380749	-0.18344266806807
C	-4.94987018325748	2.48113735465417	2.44972199238545
H	-6.12236883959322	0.81201843925628	3.11671076970572
H	-4.60333257745830	0.33619181287916	2.36232204030993
C	-6.07129697920357	3.50081144820795	2.25736003822660
H	-7.60728694700592	4.00948419282902	0.81212271979471
H	-6.08079666087280	3.52560123837361	0.09047658431383
H	-4.51102637402438	2.59912521424735	3.44543042086463
H	-4.13840209039245	2.69152970265705	1.74598091097731
H	-5.66926647175174	4.51810915084208	2.33724923828863
H	-6.80854454962167	3.38655159932760	3.06568330685495
C	3.26096591118945	3.04727050161598	1.01310412740798
C	3.95594638869971	3.56000416127654	2.13162227026201
C	3.59266579608380	1.76994131842071	0.53605798253351
C	5.00224421303690	2.85106152913806	2.69507359590473
H	3.65009712579866	4.51479493072929	2.54098150885853
C	4.64042511201866	1.05604000636550	1.09423274857856
H	3.07321708036963	1.34622839599593	-0.31148911500020
C	5.36469273598411	1.60661636202237	2.14046701466625
C	5.91943849172652	3.18449712483779	3.87279647447581
H	4.89790225194846	0.08186060380772	0.69614565221254

C	6.51540647525829	1.08475011548304	2.85394266552016
C	6.84216582161021	1.97279107534184	3.88183396903696
C	5.16277277698236	3.29393820698539	5.21901734757853
C	6.75596814724342	4.46697038895336	3.63491125680738
C	7.27960607140400	-0.05979976460018	2.64335762203127
C	7.90295151990933	1.70330811767730	4.73528700108044
C	4.37109893245001	4.59198297588707	5.37742864613700
H	5.91127096125930	3.24569611330441	6.02277255214848
H	4.51566205676361	2.41638871615421	5.33816548834336
C	5.96557949622763	5.76463434472498	3.80627401835274
H	7.57991135758725	4.46441132437835	4.36273306290596
H	7.21316628523735	4.41000474837209	2.63923842344456
C	8.33332050494850	-0.32295260688711	3.50346685743984
H	7.05705567288688	-0.73292355680646	1.82125583521552
C	8.66005069863116	0.53713203941009	4.56624979557115
H	8.14178900077911	2.39966497435233	5.53292929230037
C	5.26687701257296	5.81442203100111	5.16671127742137
H	3.92152346747020	4.62029021605140	6.37788506116988
H	3.53475744064223	4.61606248062992	4.66689276572344
H	6.64791106899928	6.61730763381881	3.70063432590564
H	5.22556753890054	5.86689266630758	3.00158800034885
H	8.91666967174212	-1.22602805772642	3.34910917445428
H	4.68358733119726	6.73819906691621	5.26416933497371
H	6.03037717009166	5.83683415704332	5.95845001578185

C	-2.88485628309534	-4.08918633241374	0.13201072009966
C	-2.65765931647855	-5.16124772744128	1.01400913508475
C	-3.71960155888600	-3.03430957697150	0.51984772243308
C	-3.26140972598777	-5.17373197491405	2.26147667130762
H	-1.97485511642838	-5.94410285632139	0.70621468930149
C	-4.34355147288944	-3.04454854002473	1.75741265699688
H	-3.90211541690461	-2.21387560247231	-0.16324730400291
C	-4.11029106851551	-4.10708920744836	2.62129707194865
C	-3.15965411159734	-6.17558901612324	3.41195952281831
H	-5.01228263417200	-2.23545310726773	2.03823021322152
C	-4.57075927282635	-4.30811576666989	3.98442276325940
C	-4.02421407609149	-5.50026231391573	4.46751173987196
C	-1.71764927550529	-6.35682453004589	3.94659697915137
C	-3.75177353362775	-7.55976562552364	3.04531673173201
C	-5.36268703169137	-3.51167588885047	4.80871080731176
C	-4.27401653957149	-5.91121819432214	5.76918600796226
C	-0.81517926289443	-7.18280660451357	3.03189058374708
H	-1.79111542895859	-6.87138337461417	4.91605958213791
H	-1.27975166988670	-5.37489902924026	4.15137454385253
C	-2.85213087610149	-8.38101599925746	2.12105036805372
H	-3.89129757414547	-8.11740252406621	3.98274680218247
H	-4.74741593565055	-7.42044096490384	2.60603785908944
C	-5.61008579444330	-3.93412094922675	6.10661311059928
H	-5.77239073331122	-2.56961729752553	4.45017416183853

C	-5.07340974035812	-5.13034427726375	6.61360169417326
H	-3.83408189358001	-6.83408242048575	6.13307968951564
C	-1.44602303870970	-8.53809925607178	2.70587812700681
H	0.15424226209972	-7.32584832526158	3.52528828548115
H	-0.59821835838528	-6.63026851701020	2.10990904173486
H	-3.30606679577377	-9.36728298898041	1.96146033986222
H	-2.79880830899948	-7.90760426410851	1.13186658716706
H	-6.22366608133129	-3.30769836497899	6.74772861866706
H	-0.80713905561735	-9.10308617384342	2.01602879862563
H	-1.51451003577308	-9.13004438707205	3.63104810928264
C	2.26022544414057	0.58368829614505	-3.25117209906915
C	3.29646470408259	-0.31361298690321	-2.92842907676097
C	2.49946981830479	1.96109941016228	-3.21597032604571
C	4.54182730093623	0.17249529908978	-2.56632430632366
H	3.10358432464435	-1.37806072246319	-2.98733017675758
C	3.72775481057493	2.46029534076233	-2.80324731921717
H	1.69821677185634	2.64289088248806	-3.48361283026883
C	4.73673194713287	1.56667451319662	-2.45883126125254
C	5.84984562430778	-0.55752645510715	-2.25855127660427
H	3.88451219072667	3.53340908997373	-2.72571282416634
C	6.05102724342470	1.81199538194849	-1.88911450887178
C	6.68762349532679	0.58175880169852	-1.68946042701345
C	6.52686188926643	-0.99564373793475	-3.59498919357972
C	5.75362733077470	-1.77651706203875	-1.31687225349946

C	6.66289849370896	2.99329344935800	-1.48071482853632
C	7.93688907577255	0.52577690034994	-1.08742309110465
C	5.87331724474049	-2.21828265540566	-4.23165190104604
H	7.57625796642624	-1.23123461569750	-3.36655337817801
H	6.53264334882754	-0.14117256834731	-4.28258250581892
C	5.18356019162735	-3.04495320133104	-1.95796916774557
H	6.77175308742501	-1.99826567831014	-0.96476127476731
H	5.15636144692338	-1.52658152809893	-0.43575521559424
C	7.90835818987710	2.92522914432461	-0.87344596546782
H	6.17314494338688	3.95445790727106	-1.61592712902349
C	8.56625148002016	1.70243418895030	-0.65907352918101
H	8.41812272925627	-0.43540446233194	-0.93707285378324
C	5.88935587541456	-3.40209879525507	-3.26461499929875
H	6.40481777177339	-2.47094591511413	-5.15772751291797
H	4.83946591808295	-1.98359171351179	-4.52012259294675
H	5.27175004883186	-3.86669661064136	-1.23742866642187
H	4.10874105144439	-2.92746639989477	-2.13479825494377
H	8.38169436131916	3.84854538691620	-0.55276595291187
H	5.41748261370728	-4.28045299746247	-3.72164130707535
H	6.93395999645586	-3.67649093630697	-3.05380487574672
C	0.26348121640838	3.98105240813111	3.63921490466481
F	1.18830191569707	3.13193005481373	4.08439313383209
F	0.80588458764325	4.82528440175096	2.76582775099897
F	-0.25095513419607	4.66263831751682	4.66063938704778

C	1.75708874966154	-4.69343356359456	-0.52495628226296
F	0.76848873085591	-5.54412671259070	-0.22395402498673
F	1.61221930863115	-4.29970018777194	-1.79977928970811
F	2.92913243531111	-5.32028794072833	-0.39324187510169
C	1.07651830575094	9.93705878992487	-0.63586083761887
F	2.25656706496012	10.18026730969033	0.04227086110127
C	1.26832432267308	10.61650798075000	-2.02373347171385
F	1.63488262764311	11.89875808184098	-1.90102563341449
F	2.23596083976360	9.97031792357608	-2.69627677003205
F	0.14186156969230	10.56995898455034	-2.75794450927110
C	-0.02697213831435	10.61548145120421	0.22799012790173
F	0.23993893677463	11.91021057561628	0.44618828169170
F	-1.23548677780607	10.52816944777965	-0.35668418630725
F	-0.09027283834762	9.99690004845186	1.41841606849492
C	-0.86360049525409	5.03576989425826	-7.65683472645772
F	-1.93755152687594	4.40314672707412	-8.24910786445662
C	-1.15404068707615	6.55724112001350	-7.82121123329829
C	0.37871152773969	4.56677754083276	-8.47038374580536
F	-1.32863107684233	6.89013239803329	-9.10706528740119
F	-2.27946321158081	6.85918291961254	-7.15378418141782
F	-0.15606157783738	7.30936821682356	-7.32324058275085
F	0.19154196994934	4.70987534464006	-9.78610874789216
F	1.48453315983264	5.25052093751905	-8.12335076797003
F	0.60176553819167	3.26102336866727	-8.22258264325815

C	-2.90653720111965	0.52670120190312	-8.58012110765496
F	-2.14529552531467	1.61268042620789	-8.96484266580132
C	-2.79535487799776	-0.47989230501361	-9.76302868729987
C	-4.34600431258140	1.09270143984304	-8.40138411707022
F	-3.19933764202666	0.06720564796486	-10.91792257187239
F	-1.51204713719998	-0.84860297517509	-9.90034304471794
F	-3.53004135998226	-1.58531470882185	-9.54323830948716
F	-4.73431906867177	1.80695763091627	-9.46272054482287
F	-5.24425475936293	0.11296870903855	-8.19795111204357
F	-4.36790830863078	1.90731268126329	-7.32574656855871
C	-1.04975433951574	-7.97215514781965	-5.96829823577435
F	-0.44317029278587	-7.77948287385973	-7.19592505035190
C	-0.15956975656828	-9.02468053291983	-5.24595462246518
C	-2.47192267516403	-8.50387196463040	-6.30915513801742
F	0.00361089390418	-10.12925371751303	-5.98754961216422
F	1.05218968565295	-8.49092948737904	-5.02259752276031
F	-0.68560501499647	-9.38972561147565	-4.06223600003155
F	-2.42325579844354	-9.57907048919972	-7.10721058993222
F	-3.15670133066269	-8.83756694600802	-5.19947960168349
F	-3.15497961565519	-7.54084859394448	-6.95090399589455
C	0.75700699113765	0.29767122401267	5.69376474522295
H	1.07542224675261	-0.48339329622958	6.39719820678754
H	-0.31602330038034	0.44558544458895	5.83191301178561
H	1.27867442333794	1.21893964090038	5.97656174368391

C	1.12352937227899	-0.12814432465259	4.30187318203084
C	0.25903338296863	-0.68791741560657	3.43546625749162
H	0.67525763227102	-1.05620733910610	2.49660244331882
C	2.56880050042095	0.04309969668474	3.93733360320322
H	2.86012819715410	1.10200281207603	3.94808545175408
H	3.20655622079233	-0.46969394314319	4.66795177893154
H	2.79509155833833	-0.36458487628129	2.94865099359642
C	-1.17753376396299	-1.04273295844336	3.66863467580229
H	-1.61460715822180	-0.44477313950213	4.47416426644657
H	-1.76182893724649	-0.82890184824119	2.76069899135682
C	-1.34631177123734	-2.54773705029215	3.98801624021056
H	-0.87401517429658	-3.12859764953542	3.18597336014170
H	-2.41905086212295	-2.77072953487435	3.96327728381889
C	-0.79163030545938	-2.94377140689727	5.33229350181951
C	0.36628106413096	-3.60901826655335	5.41296670239057
H	0.85548012382600	-3.84861869816113	4.46932479474765
C	-1.65270380908764	-2.56018376684757	6.50049828024442
H	-2.60234184447056	-3.11365175906713	6.46169279399973
H	-1.18447041702523	-2.75236727594489	7.46809030956316
H	-1.91897758045935	-1.49748083903363	6.46072076544195
C	1.08269324623507	-4.08511684626204	6.63573204644684
H	2.09726683237178	-3.66989575880181	6.66703451901762
H	0.57301262608427	-3.82058806846421	7.56517735071844
H	1.19998393750102	-5.17677710201188	6.61482245843230

O	-3.80757715937727	0.80797681447798	5.51542942190105
H	-3.23171045760936	1.46116142640757	5.07949435685003
C	-4.27948879440932	1.29883350378439	6.72590675181460
C	-5.08748489054722	0.12762882202308	7.37357211994682
C	-3.08077785671879	1.70640058385532	7.65395081981107
C	-5.21773136772253	2.53306207010084	6.48967668545934
F	-6.08155182858023	2.28504857271952	5.49567140432489
F	-4.47521491023856	3.59896164421816	6.12169224653819
F	-5.92163711626026	2.86468830320105	7.58127069524002
F	-3.46388588633025	2.47830118457970	8.68084018208072
F	-2.46699724538138	0.62017835716319	8.14741886312049
F	-2.17107400560015	2.39003008719778	6.93169191942036
F	-5.34541471949569	0.38451309643801	8.66802761614430
F	-6.26255178266120	-0.04844312934682	6.74104742991316
F	-4.41048145931359	-1.01975966123206	7.29600950381881
O	3.41091731331247	-3.24688572952389	4.12596250772856
H	2.88989259586789	-3.48113673383833	3.32392137632896
C	4.69765518748909	-3.74330739090857	4.05482809133157
C	4.69222446476143	-5.29838896282184	4.25603948408700
C	5.36640570838755	-3.38531353173761	2.68173745042425
C	5.47610907220238	-3.04931087870970	5.21943286227842
F	4.74934591458406	-3.05126257731136	6.34464285685729
F	5.74288533300926	-1.76860127966450	4.91187004082142
F	6.64283801933291	-3.67100547094057	5.47284907565346

F	5.00522990842029	-2.15164662092393	2.30793540329486
F	6.71195839632422	-3.43512278810524	2.73358703455547
F	4.96132971022857	-4.23674052377307	1.72026256029823
F	5.86100715873711	-5.86355113462801	3.90078662837899
F	4.45085250643708	-5.61115506469515	5.54094160273078
F	3.71635003291891	-5.84869139146176	3.51632938505540
C	9.82237997176862	0.16759048140947	5.48859008979474
C	10.05449357906687	1.21436689547725	6.58439168134870
H	10.30557576455795	2.19378943762258	6.16215841746163
H	9.17627058649958	1.32652637549921	7.22949731293475
H	10.89194364703577	0.90199280159261	7.21701284127484
C	9.51452323452414	-1.18221475954163	6.16633428991824
H	8.59017155941036	-1.11811656274215	6.75051700003996
H	9.38966163519504	-1.98605384349931	5.43479941517868
H	10.33181555715103	-1.46256094921000	6.84098806119865
C	11.11514679569703	0.04631280640101	4.65879838563991
H	11.03515161898101	-0.72784864501670	3.88974965165151
H	11.34224213343659	0.99439430906248	4.15913089198121
H	11.95870188468483	-0.21229615842123	5.30935920337408
C	-5.36412879072139	-5.52257191521573	8.06375662851099
C	-4.68771236118943	-6.84029017201311	8.46010750006448
C	-4.84576907184527	-4.41594824168348	9.00283240850963
C	-6.88466882606548	-5.68555190058596	8.25276799569818
H	-5.03682675763714	-7.67720209815327	7.84528838575324

H	-3.59728567578994	-6.77645780739688	8.37621646355774
H	-4.92688903008162	-7.07323484303724	9.50301858163679
H	-3.76722318541883	-4.27368677276816	8.87419540718191
H	-5.33463643728138	-3.45657248209825	8.80901544371673
H	-5.03702323791212	-4.68664539897880	10.04778750165906
H	-7.42202318968922	-4.75622153837525	8.04080056772918
H	-7.27585379267613	-6.46080574394224	7.58504699093919
H	-7.10822054713430	-5.97526783702683	9.28616194860125
C	9.94577221258727	1.70580880448871	0.00416718486380
C	10.94354898080349	2.41213140030531	-0.93487623012262
C	9.88221842674736	2.46258602047754	1.34535763384019
C	10.46313897263734	0.28998945506271	0.28404775684860
H	11.00687848324456	1.89072030390662	-1.89615370974761
H	10.64421728864397	3.44639813466479	-1.13223329114513
H	11.94261522735509	2.42819738368164	-0.48346921709219
H	9.14691317164287	2.00684860388536	2.01558327886325
H	10.86192264200594	2.43084340348517	1.83634302797199
H	9.61570851977219	3.51534455147009	1.21036054363742
H	9.78520380942527	-0.25703057714061	0.94777064854891
H	10.59846755495457	-0.28662329145395	-0.63761378220532
H	11.43777984259387	0.35070961903953	0.77994714987232
C	-9.17868462200074	-3.07770345486435	2.72832044029993
C	-10.58554113386980	-3.22002802167902	2.11469038889162
C	-8.63292361506845	-4.47387428592833	3.08619106286174

C	-9.29839029913312	-2.26812996143575	4.02542269452065
H	-10.99647359375280	-2.23833491473408	1.85491550083180
H	-10.56773392129720	-3.82894657789776	1.20528715531274
H	-11.26329806955956	-3.69994304645506	2.83034280484590
H	-8.59692829052673	-5.13587009479761	2.21612223861823
H	-7.62217077187327	-4.40599039779648	3.50176047810023
H	-9.28006476429734	-4.94520921878653	3.83479654500564
H	-8.32482063745332	-2.13463307012261	4.51037883533968
H	-9.74200418855438	-1.28148751712122	3.85317810856857
H	-9.94696618309905	-2.80202600570748	4.72756604255478

TS1

O	-0.49738701809537	-1.44414560167972	-1.71831551699788
O	-2.72302365776769	-1.09917738965387	-0.46465607864753
O	-0.94072989686146	2.91139135856533	-0.10244669101643
O	1.10076341153055	1.55876306502132	-0.74435288997876
P	-0.08029799807933	1.63646179899564	0.34385749595244
P	-1.12542044449501	-1.14201099117786	-0.25106020004164
S	0.38925408530396	3.38705502915811	2.71510555528148
S	0.43222726055588	-3.40442458340752	0.56244094525969
O	-0.49177573499138	4.42248555961472	2.18593258336897
O	0.28427843661663	2.94615074246540	4.12168647691124
O	1.59270590610416	-2.92897393491055	-0.20344573050117
O	0.66966802588238	-4.02489627701462	1.88814852535872

N	-0.77432994989699	-2.38124615453917	0.70115665153757
N	0.47695303349244	2.00273568345253	1.84746875410361
N	-0.81342932350313	0.27246198815463	0.37061678260387
H	0.65396750212513	0.89246890210176	2.66186639872321
C	-3.26795266291373	-2.22784490518883	-1.08483931473632
C	-1.06837199880789	-0.96550834805037	-2.89274151826722
C	-1.12964490688551	3.21431922050986	-1.45294309903673
C	1.88852939005668	2.69341150153595	-0.95177859111577
C	-3.93891489731700	-3.18358263377193	-0.28046140434205
C	3.25503174911782	2.62708888823690	-0.56404367536515
C	-0.22621587164089	-0.22193481665224	-3.76577024873915
C	-2.44292551843460	3.07684061261013	-1.97539009793528
C	-3.71082741691844	-5.01288041340625	-5.00714739678700
C	-3.29231752125407	-3.83428806495955	-4.44172338564957
C	-3.52958491636206	-3.55076498181244	-3.07779725568105
C	-4.18242728484693	-4.54463006837855	-2.28599273576047
C	-4.61366651770039	-5.74514745781882	-2.89340380118210
C	-4.39056174847796	-5.97770998655199	-4.23201495020728
H	-3.51188885034360	-5.20580790347894	-6.05406331092825
H	-2.76077644753325	-3.11316908361479	-5.05269225846493
C	-3.09007223769675	-2.34812870830201	-2.44306869136710
C	-4.38435391311129	-4.32604282414979	-0.90439157737549
H	-5.11543068524976	-6.47712351906404	-2.27082517754447
H	-4.90403909123401	-5.07970047340932	-0.31781016209094

C	-4.17912459297498	0.83294405575479	-6.39491915361325
C	-2.83620486045490	0.98437907088543	-6.13866331752984
C	-2.21403458130524	0.27599077122972	-5.08599366347736
C	-2.98882646481529	-0.62811166447836	-4.30043147053815
C	-4.36413918579755	-0.77110751644824	-4.59717346655959
C	-4.95181687152163	-0.05716795412884	-5.61037867489464
H	-0.22061912932874	0.98973780405082	-5.52213187775758
H	-2.23315080797944	1.65888793986509	-6.73533267338873
C	-0.83006172651834	0.40848637644223	-4.83351047864733
C	-2.37278351054951	-1.29882119129546	-3.20394723161560
H	-4.96913078683454	-1.44134655453256	-3.99516685137351
H	-6.01124237599551	-0.17996027006618	-5.79754703792464
C	0.56554836270163	4.64446687277110	-5.75670806425566
C	0.80492139565566	4.32590729423320	-4.44446150899739
C	-0.24751614101346	3.98507444260387	-3.56062595967432
C	-1.56709662754663	3.91148418380897	-4.09696166840428
C	-1.79626549617301	4.27502935511679	-5.44558506423283
C	-0.75641304615429	4.64789954606991	-6.26221892415066
H	1.40208080512602	4.89551621240764	-6.39668787797301
H	1.82754147690493	4.33330885666237	-4.08599140313747
C	-0.04622959797548	3.67271950884032	-2.17765799926131
C	-2.62956823638643	3.46166007766736	-3.28890658121965
H	-2.81159241334090	4.24429816626158	-5.82415497430816
H	-3.62683499650260	3.41571529501788	-3.71813083369236

C	3.67868727260941	7.28030215310379	-1.93926463005994
C	4.23830947975968	6.10618734365900	-1.49667457712780
C	3.45055373670687	4.94211604190297	-1.33762807015408
C	2.06932284846795	4.98054725036437	-1.68574375134725
C	1.51638869049528	6.21116284359909	-2.10621142531854
C	2.29309371954801	7.33556531871995	-2.22700768608276
H	5.07951651630742	3.73598492613550	-0.62860083326125
H	5.29249611933675	6.05764974497995	-1.24789417840130
H	0.45588774799072	6.27103013723208	-2.32609629789613
H	1.83211005849733	8.26370283773938	-2.54028351693292
C	4.01261817426935	3.75448826456632	-0.83032806611112
C	1.30421384697182	3.78021150958113	-1.57606776409031
C	-3.57176467317584	2.53012328561720	-1.19500394300733
C	-3.81346834968882	2.90135281516475	0.14021453378211
C	-4.44768909420598	1.64114254961254	-1.83364207637582
C	-4.90824040400999	2.38196847258775	0.81376987096313
H	-3.14634357899954	3.60593172627947	0.61826962410182
C	-5.54737961438229	1.11790753693177	-1.17001796303514
H	-4.24624434429588	1.33882821782439	-2.85671946365270
C	-5.77692958250677	1.48930678347843	0.15134002608639
H	-6.21040464967212	0.42319198084717	-1.67985742594114
C	-6.60687108563818	1.71164098106026	2.30130191434153
C	-6.82024200058206	1.07235498898222	1.07721907772513
C	-7.42399992385592	1.44726855102233	3.39175879647332

C	-7.86304108135774	0.15724684690209	0.94195763031455
C	-8.46274873608382	0.51637418727208	3.28320185730482
H	-7.23662183104978	1.94992888397948	4.33534726202330
C	-8.66413210692980	-0.11246152090008	2.04171087029929
H	-8.03921746803422	-0.35822431656899	0.00119767893336
H	-9.46167594911289	-0.84174605560438	1.93575714290947
C	-5.37078129088243	2.59320908601845	2.25486137428955
C	-5.72228009425183	4.06211335303514	2.59244721763269
C	-4.34535809877681	2.05242295571374	3.28271057477227
C	-4.50093085472406	4.96056299758042	2.79884458213376
H	-6.30590979396863	4.05186784850247	3.52422697633573
H	-6.37921626311629	4.46168629308067	1.81031450532270
C	-3.13303870242527	2.95178818632699	3.47277297891420
H	-4.86433051688701	1.95295213080109	4.24714991250339
H	-4.03876977370241	1.04578525813153	2.97557071533297
C	-3.54218325902465	4.37752225330269	3.83909996822040
H	-4.83988549203569	5.95476735006618	3.11512505104116
H	-3.97124542981836	5.10946894070969	1.84999139042851
H	-2.49774507677409	2.52785006253041	4.25894408763923
H	-2.53326123597251	2.96309780902245	2.55590631510354
H	-2.65505114335882	5.01408073406787	3.93279151179368
H	-4.03207094288795	4.36688134319721	4.82421679658204
C	3.88186831559837	1.44242103235042	0.05542222882235
C	4.97464170463610	1.64571507638576	0.92710100589764

C	3.48858610463773	0.12775927812157	-0.24290337726763
C	5.68603150696963	0.57237483454929	1.43036751474634
H	5.22812258118661	2.65915070878195	1.21107005508994
C	4.19568497986533	-0.95422061570908	0.25999013544876
H	2.65743218140531	-0.06754279831277	-0.90627854505211
C	5.30450849337116	-0.73350422734906	1.06375362247890
C	6.89631418999945	0.52512203116365	2.36296841384097
H	3.87506105878339	-1.96033069189458	0.01652076187010
C	6.21705921761992	-1.68240806365716	1.67416736008421
C	7.14499587616583	-0.97567238498971	2.44388284074892
C	6.60333577566190	1.09472371476242	3.77315633651514
C	8.13817463341219	1.22726126861759	1.75833684743644
C	6.29023791279134	-3.06986490827019	1.57953006238535
C	8.14265796758957	-1.64747168610480	3.13597856374863
C	6.55155456619341	2.62151081714889	3.82504446089018
H	7.40482745648392	0.74989572977864	4.44214501416450
H	5.66878974680069	0.65920567343374	4.14968758388611
C	8.08847444304187	2.75354890026142	1.82774089137598
H	9.02058686563139	0.88117007137695	2.31551775701508
H	8.26104850316438	0.88464308317920	0.72449150062981
C	7.29395209782138	-3.73137527136363	2.27052522072248
H	5.57940164103537	-3.62633919883222	0.97505874003841
C	8.23343246601964	-3.04298419290845	3.05837698590661
H	8.85782687072307	-1.08653579419085	3.72948328126359

C	7.82955462189590	3.23895223485480	3.25485535609760
H	6.40682931324814	2.94194194698376	4.86436705898125
H	5.67994421256875	2.98851816594385	3.26966771320193
H	9.03705629138172	3.16006023698628	1.45515226700274
H	7.30908949104321	3.13847676568674	1.15700633959543
H	7.34646269175303	-4.81364120743049	2.19715718692798
H	7.76971900076565	4.33379845695036	3.28186951883513
H	8.68081741620852	2.95268533621338	3.89056053410177
C	-4.14152429500026	-2.94469292885146	1.16273363942346
C	-3.65168063672522	-3.85599284408901	2.11087730221159
C	-4.81904590073044	-1.79427213675346	1.57579546987836
C	-3.76369092296812	-3.56329904935950	3.46154681884023
H	-3.13312962648578	-4.74023270817428	1.76191924624123
C	-4.98399182677491	-1.51836580364474	2.92516840333155
H	-5.21378876027687	-1.11002351177506	0.83182008186837
C	-4.40717050844956	-2.37343122692793	3.85776824782435
C	-3.24739471072126	-4.31396454879266	4.68777254057701
H	-5.53308919995584	-0.63531418109803	3.23760880910541
C	-4.23169815380048	-2.20038871970625	5.29195848931101
C	-3.49503547249225	-3.28416276692040	5.78132560704892
C	-1.75956336928197	-4.72409760875877	4.62249347448788
C	-4.12245563959210	-5.56417161144936	4.98630522527478
C	-4.55451840116825	-1.13696411812265	6.13205250104289
C	-3.07285153500315	-3.31068393979655	7.10284571236871

C	-1.46323151204141	-5.94641859695728	3.75160053861588
H	-1.44108329312569	-4.95530746480435	5.64835139402077
H	-1.16154301735913	-3.86765598032588	4.28801950188255
C	-3.84197283287805	-6.73171854588582	4.04364001532370
H	-3.90457574691998	-5.88120490995843	6.01653613932150
H	-5.17886217358978	-5.27040762174869	4.95780695179602
C	-4.11112473446706	-1.16440653473666	7.44755969622839
H	-5.11797189678440	-0.28347679058926	5.76108555847484
C	-3.35860601727490	-2.23703498459239	7.95638706636297
H	-2.50051323339272	-4.15837576511133	7.46588800611568
C	-2.36630944706873	-7.12900227083571	4.10050437304854
H	-0.41058951059397	-6.22371072546221	3.88032519210906
H	-1.57184047579865	-5.69485036942118	2.69165097471657
H	-4.47875875009978	-7.58131268515372	4.32005585470169
H	-4.11743706918975	-6.45749834397474	3.01602655496341
H	-4.34131114770399	-0.31990030503081	8.09076758180970
H	-2.16554176299975	-7.96766429418048	3.42307956862951
H	-2.13123121699880	-7.48010608380616	5.11667910467845
C	1.24403945520992	-0.23078323758114	-3.60898293171599
C	1.91727771377838	-1.46058474618155	-3.47224822243498
C	1.98041275388796	0.95229484528468	-3.69830121493100
C	3.29873355139468	-1.48640536302350	-3.39115869742932
H	1.33591446821866	-2.37473716833977	-3.44068305189495
C	3.36317627572645	0.94455236117133	-3.55204218474466

H	1.45309306491134	1.89063325478914	-3.83254216829556
C	4.01557775184785	-0.26883230746245	-3.36261073452196
C	4.27780011538656	-2.65950258369235	-3.33657948356388
H	3.91531832309167	1.88112359680251	-3.54914821872199
C	5.40567100100595	-0.55583710714055	-3.03571165161565
C	5.55702863261347	-1.94317907067220	-2.93272643472320
C	4.49261848551543	-3.20793607355887	-4.78144750001981
C	3.92983366564624	-3.83242995319557	-2.40208373263594
C	6.47613141985836	0.28356963530822	-2.73888541657689
C	6.76295585095596	-2.49643760425485	-2.53277578513026
C	3.32438868382633	-4.05388172371836	-5.27871263084037
H	5.40163710866253	-3.82647862390945	-4.76402397768008
H	4.69049274953333	-2.36474368013806	-5.45435236033118
C	2.81547338353335	-4.74935285912331	-2.90986253157090
H	4.84060512888350	-4.43825609442217	-2.29173041196669
H	3.67554247871718	-3.45369890091884	-1.40998694212591
C	7.67972220583309	-0.28049395230944	-2.33348500895126
H	6.37307851038360	1.36450751113883	-2.79844736824322
C	7.84719166403521	-1.67030792996823	-2.21130775420782
H	6.85681776045648	-3.57370888234383	-2.44841609375664
C	3.07074167967954	-5.23159938959190	-4.33736298686512
H	3.54190229835246	-4.41319420184513	-6.29232116286276
H	2.42063612225028	-3.43333569781124	-5.35498317251324
H	2.73270838508423	-5.60601474609837	-2.23113714981492

H	1.84885474066832	-4.23603885504230	-2.85698825776728
H	8.50925588980156	0.38095460186773	-2.10022928483532
H	2.22363041456676	-5.83216826256962	-4.69075113273713
H	3.95138266747416	-5.89149801862506	-4.34552153840150
C	2.12980878425746	4.14500922299373	2.65630156819545
F	3.05754456961163	3.19384804203993	2.79876595578237
F	2.31435518012562	4.78571855637416	1.50302607349102
F	2.23083628613266	5.01454711125021	3.66240212733722
C	-0.27975359864146	-4.83493065470460	-0.47136170936317
F	-1.36195617817021	-5.35530952070377	0.12189409065562
F	-0.62903633834707	-4.40241050240132	-1.69082798865009
F	0.64386987075807	-5.79234818407112	-0.59438805956730
C	4.56424180596253	8.49983360642114	-2.09840132750976
F	5.87427742635227	8.18393109898026	-1.78833986694252
C	4.61257885165883	8.98575176898627	-3.57704709278366
F	5.52754633316559	9.94788630624144	-3.75232225016451
F	4.94334673583296	7.94941251946032	-4.36643313624647
F	3.42049475693330	9.45998288185566	-3.98234849369326
C	4.17915367777073	9.62838273461692	-1.09759264152226
F	5.02830023097928	10.66305386040769	-1.16626661535814
F	2.93658456394255	10.09006460042803	-1.32612626738401
F	4.22266054149574	9.13625261139916	0.15112056268438
C	-1.04905970279078	5.04738180661405	-7.69409231513977
F	-2.40008325660551	4.93780431106770	-7.95506752115798

C	-0.71276231743327	6.54691987735883	-7.94856482991636
C	-0.36274715063624	4.09347875863533	-8.71605307000412
F	-1.03243166141510	6.91999126044098	-9.19550885753450
F	-1.41970677589936	7.30137882157888	-7.09198244873279
F	0.59352104115214	6.80279689020578	-7.75535731176937
F	-0.79956861394265	4.30342398018736	-9.96185918465338
F	0.97507380830894	4.23851462969670	-8.70623868583341
F	-0.64578592316713	2.81718232924682	-8.38816702938679
C	-4.80665255153742	1.63559318517443	-7.51615389859351
F	-3.85094929559346	2.41326018609822	-8.14041033228916
C	-5.37310853498541	0.72047749615951	-8.64177597152658
C	-5.85532302498561	2.65152411200286	-6.97597824760532
F	-5.77066708150802	1.43408351578199	-9.70336867161829
F	-4.41038424057671	-0.12404859458008	-9.04431254132168
F	-6.41981505963776	-0.00745972897640	-8.20966026925583
F	-6.31779820332028	3.45349314907578	-7.94142184545283
F	-6.90112751537577	2.03167358830602	-6.40311723304790
F	-5.26935344107169	3.42777881842715	-6.04003384249216
C	-4.84551607257908	-7.26013989452891	-4.90263214743337
F	-4.50922928776214	-7.24342849064229	-6.24380787374042
C	-4.11199164161906	-8.50533920361438	-4.32496409614117
C	-6.39523558829088	-7.40104865136541	-4.88483160941920
F	-4.41184041022771	-9.61801565026313	-5.00935531798535
F	-2.78628220026802	-8.30905184530612	-4.40945114629447

F	-4.42677891968566	-8.71093975176386	-3.03279673530685
F	-6.80542012336127	-8.45103616592912	-5.60896703456433
F	-6.86637363774984	-7.54610161762762	-3.63227338011072
F	-6.94095126069418	-6.29276254163936	-5.41327768857623
C	0.27660285051329	0.30150135913605	5.59468767457849
H	-0.56158482870628	0.95755671812855	5.85536089701849
H	1.21401271105171	0.84291785848212	5.71282567432661
H	0.25695406449049	-0.52548089935547	6.31657528694785
C	0.08527093725470	-0.27286023323005	4.23827251419277
C	1.04753189592726	-0.26270522921316	3.24285399800147
H	0.80890701311201	-0.90676966641217	2.38894688761788
C	-1.28572123015873	-0.76699107253444	3.94472504957981
H	-1.67523495364525	-1.33314898531328	4.79971622818263
H	-1.95921071947033	0.09689274674127	3.83991500064062
H	-1.34435461236584	-1.37081269418138	3.03698066587345
C	2.52990766615727	-0.08981507809328	3.47004900656551
H	2.99689613283016	0.28261050391335	2.55135786122231
H	2.73807624439064	0.64814264276974	4.25317229638795
C	3.16039487897634	-1.46231515103374	3.82470412799063
H	4.25368807313565	-1.36245336901227	3.79570047500712
H	2.88370551399238	-2.17697961649278	3.04133253069117
C	2.71462586978487	-1.95331600308518	5.17416153002525
C	1.70132778065837	-2.82487265955477	5.27447925624337
H	1.27242730453027	-3.19613528775466	4.34199364446865

C	3.45823607173131	-1.35661685040656	6.33651036859898
H	4.48487858495749	-1.74537944529506	6.36196850171530
H	3.54512542834375	-0.26763408866927	6.22878576086430
H	2.99600738932860	-1.55955940450068	7.30415019383752
C	1.07627703180285	-3.36247692790787	6.52246256191374
H	0.02536329513079	-3.04408847987620	6.59945395127626
H	1.05934270187691	-4.45915635672398	6.50331673010322
H	1.59159036266189	-3.05089746926059	7.43394861335096
O	-1.27434786074457	3.48687130095106	6.32435905408960
H	-0.67364265315657	3.49107172684371	5.54874267591426
C	-0.80923053902563	4.33121745514775	7.31710884535761
C	-1.84112112571381	4.21613196142373	8.48461182857204
C	0.61091311555394	3.87957673387557	7.80806715342807
C	-0.74957818431127	5.80740418980962	6.78950025263785
F	-1.98221580782000	6.32953109058948	6.69342958630936
F	-0.20794937085703	5.82238183138587	5.55668175334653
F	-0.01317064322825	6.60452253215111	7.57964664336169
F	0.94857825126295	4.42927702732169	8.98448376519786
F	0.65934974566606	2.54518308377740	7.93922897621259
F	1.54363315045985	4.22551229529811	6.89680932199158
F	-1.68437872322490	5.21244627757990	9.37270317708624
F	-3.09400747791671	4.25900598060079	8.01803861620246
F	-1.68891531725940	3.04797275299283	9.13528151183455
O	1.74260221607695	-6.52164605935941	2.36650787323761

H	1.53779382280715	-5.58051928019742	2.16327642403340
C	3.09945583375665	-6.74407148808382	2.49971196933257
C	3.24524959349195	-8.25685296077367	2.86682841980155
C	3.83867060535963	-6.44909577516671	1.14907535886406
C	3.70425903564284	-5.85563945616017	3.64347594061438
F	2.88552178497847	-5.83782253312908	4.70278298208945
F	3.84307010391566	-4.58682570819667	3.21284463906651
F	4.90996608202909	-6.29131336204077	4.05159023172672
F	3.40370780022327	-5.28214209220241	0.63929229751494
F	5.17519045144175	-6.36178436578742	1.29834194361360
F	3.58379172282422	-7.40458428629236	0.24209076889924
F	4.51305796691408	-8.67789374062122	2.69760641511527
F	2.90527818914133	-8.46792428700575	4.15013745150743
F	2.44812463322608	-9.01125078831309	2.10427724596262
C	9.31905151990625	-3.83774887789502	3.78649868278052
C	10.25655458355943	-2.93764835946290	4.59982184600879
H	10.78406816012251	-2.22078277214296	3.96081683785359
H	9.71598175021957	-2.38270380347735	5.37451186436641
H	11.01150024832529	-3.55386761439667	5.09896106701717
C	8.65593760263515	-4.84212854070060	4.74887757735032
H	8.03600150211017	-4.32010885259150	5.48576764969063
H	8.01563326785086	-5.55220469167965	4.21719994299610
H	9.42173187021254	-5.41499993400798	5.28465784340039
C	10.16642703943518	-4.60239043152872	2.75075561444149

H	9.56252169573829	-5.31009903833784	2.17501114897718
H	10.63349433289824	-3.90683802215355	2.04488408214926
H	10.95871030390026	-5.16851772918838	3.25425763720027
C	-2.84608010696026	-2.17721875684614	9.39741364666083
C	-2.08180537319608	-3.44468869923575	9.79847916726754
C	-1.88910558699074	-0.97609194683588	9.53558174648501
C	-4.03156981604720	-2.00419808643045	10.36541029454520
H	-2.71140162528412	-4.33826506733391	9.72572643849421
H	-1.19112858106804	-3.59424628846655	9.17775033985600
H	-1.74850809398220	-3.35616831382880	10.83768184337520
H	-1.03370607759992	-1.08886900956691	8.85938569961618
H	-2.38447262433353	-0.03039192080571	9.29574653336729
H	-1.50880872847683	-0.90862389162622	10.56147745847066
H	-4.58410007987556	-1.07950794561051	10.17461681020891
H	-4.73258407120186	-2.84074659429683	10.27309239882113
H	-3.67147497828344	-1.96959317702655	11.40003353584216
C	9.19645738332259	-2.24083795993620	-1.76405270448295
C	10.23479373521629	-1.97647351106476	-2.87170567465671
C	9.65695715478843	-1.56347287395149	-0.46052628525830
C	9.13122749914748	-3.75103016272109	-1.50331438754371
H	9.93109860680378	-2.45351789403484	-3.80985797309520
H	10.35076486446626	-0.90450613642921	-3.06171930456548
H	11.21257355865933	-2.37845733734428	-2.58046677501315
H	8.91816589756073	-1.70268818375683	0.33595215716480

H	10.60605030054964	-2.00142516470500	-0.12963272414243
H	9.82180242790414	-0.48975944275930	-0.59131921515378
H	8.39038060118531	-3.98889012543327	-0.73131836107876
H	8.89098704132406	-4.31558639713518	-2.41071148928447
H	10.10693821792032	-4.10060500325914	-1.14923110472625
C	-9.34949906143002	0.13589983475231	4.47025755158137
C	-10.82679891022503	0.39199848515915	4.11671241257825
C	-9.14452158944710	-1.35961121379465	4.78431698566899
C	-9.01341307036817	0.93873406780166	5.73260000617467
H	-10.98897968101806	1.44767258684977	3.87404995394755
H	-11.15027528099606	-0.20415005463993	3.25839873012170
H	-11.46867352297934	0.13114709333118	4.96594630512818
H	-9.41512520925486	-1.99318653344581	3.93424604445810
H	-8.09670455461715	-1.56270560780324	5.03342788713860
H	-9.76495034262817	-1.65538932381264	5.63832365730924
H	-7.98623635339791	0.75691409844171	6.06838693965129
H	-9.14545144646758	2.01532373895354	5.57804930726609
H	-9.68191610341783	0.63486635238819	6.54458667367328

TS1′

O	0.70490901541633	-1.40473786014057	-1.89611385483138
O	-1.72846632097177	-1.79262368662245	-1.17151172793301
O	-1.61763941430709	2.36181705034238	-0.26416649455198
O	0.90052633569786	2.04965288771721	-0.42513146695376

P	-0.40575578442268	1.49099376355310	0.33089510153087
P	-0.27031234309759	-1.42194174523630	-0.59568109831154
S	-1.12915543336536	2.95158983400142	2.81839995380620
S	1.64800799397677	-3.18348954888977	0.58521397620002
O	-1.85052253261218	3.95419437223452	2.04162769190986
O	-1.75895591565246	2.28340502996425	3.97067933443486
O	2.75180130835643	-2.29873230976133	0.18396586469587
O	1.71637266071546	-3.82823240645721	1.91939177104834
N	0.18364694625593	-2.61032089869526	0.37392137662011
N	-0.37268134367893	1.79352000543982	1.94401670845109
N	-0.47567224066275	-0.03501762423896	0.10457042588847
H	-0.02574010632988	0.66273486212081	2.73348129987864
C	-1.78271216284233	-2.98706485228959	-1.89548354925925
C	0.25346328249881	-1.00132517220893	-3.14889166704881
C	-1.63078554799400	2.72021814344249	-1.61680966830065
C	1.14506159191824	3.42447849721648	-0.40990494175113
C	-2.31158194678671	-4.14330260312168	-1.26426595417310
C	2.27793498695681	3.89624981613681	0.30772173457912
C	0.94557793900180	0.06968759174954	-3.77821465048420
C	-2.63957352636389	2.15128093680252	-2.44033392657711
C	-0.53837005665484	-5.50734303864237	-5.82756035601255
C	-0.60303694315639	-4.30146613726676	-5.17461260538110
C	-1.20906363101166	-4.18854632137611	-3.90334010668651
C	-1.71875986473109	-5.37297408259015	-3.29026657011109

C	-1.65335856143384	-6.60018854674089	-3.98815046141620
C	-1.08013228796781	-6.67069757692120	-5.23787981788680
H	-0.06342935157244	-5.56704516812437	-6.79898185770347
H	-0.17126128787834	-3.42263851036120	-5.64072406909641
C	-1.28651733686369	-2.96024574875641	-3.17842136860606
C	-2.27573815442850	-5.31298520557898	-1.99459227248714
H	-2.05871100799098	-7.48130138987913	-3.50413511003330
H	-2.69170168278844	-6.21879641134647	-1.56088445928425
C	-2.37083730683689	-0.06327721846984	-7.33519394859578
C	-1.25999082848841	0.50225001531922	-6.75445670451236
C	-0.72381488097270	-0.01874293447845	-5.55449511711694
C	-1.32382601328450	-1.16884232178372	-4.96300338364849
C	-2.47672872921521	-1.71485212006048	-5.57444446928134
C	-2.99548482603110	-1.17759523302125	-6.72496249288032
H	0.93604192612245	1.35996287716663	-5.47666988230810
H	-0.77667205809367	1.36035848041408	-7.20699672029272
C	0.42533269858340	0.54820352419930	-4.96341955300119
C	-0.77829762029813	-1.69417324405863	-3.75470166973715
H	-2.96707214730260	-2.56840360870404	-5.11795727701424
H	-3.88464480667157	-1.61831743822998	-7.15826655679878
C	0.25662764679317	5.11210736516022	-5.37865198236814
C	0.29592285602813	4.79245839884761	-4.04563514948564
C	-0.69272049682298	3.97072466592833	-3.45285098316368
C	-1.69637432134441	3.41728476850847	-4.30103471282174

C	-1.73515799428922	3.78303082374994	-5.66736917609417
C	-0.78907397613204	4.62587709615841	-6.19910098546113
H	1.03379857786857	5.74376411483120	-5.79054332363694
H	1.10829292198360	5.17658476974658	-3.43923065629876
C	-0.70036062664191	3.63790046079609	-2.06119022100632
C	-2.64815996692232	2.52448648134557	-3.76911284869843
H	-2.52815586905801	3.38123205798322	-6.28769370829432
H	-3.42375637123450	2.13226009342445	-4.42225256310363
C	0.87986101953275	8.42421704294840	-0.82395362611145
C	1.77972332634698	7.55458809071926	-0.25712824560634
C	1.58357441046525	6.15553480419106	-0.33456254313016
C	0.45717197709235	5.64634894227334	-1.04296625040963
C	-0.46281541061686	6.56672125964450	-1.59451283337232
C	-0.26523233988342	7.91987944782736	-1.48798203315559
H	3.37239538540362	5.67582566695064	0.75086923029735
H	2.64974752538818	7.92857716859611	0.27090097424098
H	-1.34792063067828	6.19652686748086	-2.10056520579207
H	-0.99735432363243	8.59452578627430	-1.91330635155241
C	2.48647591091790	5.26433772832083	0.27627696338490
C	0.30095208087046	4.23256665289121	-1.14679612642302
C	-3.66985824955747	1.23507688955257	-1.91540752204851
C	-4.35597441766304	1.54198513946407	-0.72832602503612
C	-4.01391739127734	0.08721968689440	-2.63850432021799
C	-5.34429418554343	0.69374924285000	-0.25983332350423

H	-4.10171881940116	2.45478219170763	-0.20635910912179
C	-4.99930452328085	-0.77467040709397	-2.17516225819395
H	-3.47716971374154	-0.14953230416957	-3.55272543777865
C	-5.64870751257745	-0.48109763170075	-0.97890897114740
H	-5.23298390034395	-1.68120529670874	-2.72816091471168
C	-6.90733645338349	-0.56528316531114	0.96503180665840
C	-6.61525696438530	-1.25757047125645	-0.21540719547609
C	-7.73286951032493	-1.12700486714251	1.92907001105781
C	-7.16768185691487	-2.51589308313158	-0.44060984819215
C	-8.27794064855216	-2.40251324265870	1.73286390381366
H	-7.94060855980585	-0.57859490193556	2.84249497763842
C	-7.98860347529956	-3.07010998345059	0.53047777299927
H	-6.94389680686467	-3.07362216829850	-1.34633492986963
H	-8.40542898614179	-4.05706309936898	0.35374651353173
C	-6.22290216970211	0.79257923139775	0.98444276369740
C	-7.31521079317824	1.88585187890371	0.80960197154053
C	-5.45078711128161	1.03944725821104	2.29921073483338
C	-6.77566841427415	3.30833430127687	0.93405928945607
H	-8.07034847811897	1.72269024625714	1.59227686646771
H	-7.81642869558285	1.73267171388112	-0.15401452668341
C	-4.94394308535144	2.47426726836233	2.45574053719094
H	-6.13128713637062	0.82025359487802	3.13439009357379
H	-4.62009733560532	0.32548343378239	2.37512806682344
C	-6.05942124822472	3.50173876305142	2.27092032145779

H	-7.60679560856892	4.01835013700926	0.84031781739090
H	-6.09046244780667	3.52493869964526	0.10422014567582
H	-4.49570822719915	2.58939492581504	3.44701027132233
H	-4.13482791609040	2.67687333613027	1.74741002758178
H	-5.64866221945321	4.51587295083907	2.34500926086875
H	-6.79067735316863	3.39472594914982	3.08579989508447
C	3.22476118409980	3.02145372760946	1.02730469238213
C	3.91268499158332	3.54276011370404	2.14524191138626
C	3.54268839737765	1.73072098050320	0.57787805222870
C	4.93610695997017	2.82632081325617	2.73952914163597
H	3.61589256872377	4.50894813712736	2.53322640374839
C	4.57244150922817	1.01036396915437	1.16297710761357
H	3.02581919604622	1.30046007175233	-0.26827387244374
C	5.28876028256189	1.56730206337110	2.21237058094789
C	5.83356977254864	3.16549870259946	3.93021440677794
H	4.82314135325202	0.02737971312241	0.78098422941667
C	6.41943897506952	1.04166227870395	2.95632250359567
C	6.73661816302764	1.93995339952597	3.97861041100590
C	5.04791600685096	3.31148744639259	5.25643767598169
C	6.69738691736767	4.42960843300352	3.69027687183658
C	7.17799605044028	-0.11144814007136	2.77398212707668
C	7.77795851252848	1.67077374609915	4.85573522448025
C	4.27918346446241	4.62742741655778	5.37854416197945
H	5.77470571820401	3.25720597518935	6.07959223895957

H	4.37808098583572	2.44951619441972	5.37144982819548
C	5.92827389639220	5.74404709160837	3.82473103155751
H	7.50561214934383	4.42399780924932	4.43541960696226
H	7.17376389683934	4.34780780191668	2.70556356344115
C	8.21290886563267	-0.37416977204939	3.65745189392609
H	6.96966217628417	-0.78780657008635	1.95117428468656
C	8.52701062044235	0.49534691803889	4.71634426485199
H	8.00971105038559	2.37618595491992	5.64754416589208
C	5.20419494870584	5.82814078513391	5.16994417066292
H	3.80870956904099	4.67987718251802	6.36841137574056
H	3.45955898548619	4.65902166247833	4.64964328061259
H	6.62918583551197	6.58161549034700	3.72024130515946
H	5.20688824932940	5.84823934265296	3.00368545743720
H	8.79305604797291	-1.28243683015074	3.52203723282339
H	4.63722734513957	6.76415528599364	5.24220719112800
H	5.95192624354663	5.84686035730981	5.97674297895040
C	-2.88464182059873	-4.10965678378646	0.09328561136784
C	-2.61263451934281	-5.15789738591835	0.99035011999456
C	-3.74795772330437	-3.07498905994512	0.47223926280238
C	-3.19771974459770	-5.16366287639703	2.24713232425492
H	-1.90906365501778	-5.92416681386293	0.68763684267217
C	-4.35621155540040	-3.08096946034205	1.71793894968742
H	-3.96075947661119	-2.27210390231786	-0.22302347880405
C	-4.07530149958406	-4.11784992215819	2.59907169262477

C	-3.04707683596303	-6.13889368930522	3.41476659181594
H	-5.04757705914419	-2.28798415407342	1.99051103541330
C	-4.50496260413645	-4.30422719457208	3.97491996378174
C	-3.90980358863091	-5.46644229426917	4.47360098161798
C	-1.59105431085552	-6.27525074071721	3.92475266681845
C	-3.61263658962893	-7.54338221626680	3.08363602652278
C	-5.29966834836266	-3.51150319382284	4.80035880710648
C	-4.11789959183777	-5.85431665806217	5.78968191493473
C	-0.68589044866413	-7.10147016319387	3.01194707536460
H	-1.63437125489396	-6.76798383436859	4.90715997386530
H	-1.17170451602313	-5.27829312068834	4.09592118261174
C	-2.71085336445714	-8.35848901279011	2.15638584682921
H	-3.72263258044002	-8.08897084456744	4.03208028938727
H	-4.61883967320108	-7.43415880370557	2.66036555791908
C	-5.50154300087894	-3.90841473701113	6.11441056354703
H	-5.74766748865132	-2.59170180054167	4.42996741075998
C	-4.92046825879885	-5.07773822710538	6.63525837643033
H	-3.64370802053760	-6.75631506996751	6.16292971115223
C	-1.29311935642990	-8.47563321485889	2.72140835844976
H	0.29384293145322	-7.21512157120069	3.49251123942164
H	-0.49430862470824	-6.56253269843687	2.07659106272292
H	-3.14549500475654	-9.35659419521918	2.01873173354149
H	-2.68297413237440	-7.89843992982430	1.16002246560342
H	-6.11692793142874	-3.28375275996587	6.75558304071155

H	-0.65263579384204	-9.03891561074810	2.03176183581870
H	-1.33386729411354	-9.05182512292056	3.65810421668255
C	2.22920687886426	0.58594013119816	-3.25909188752319
C	3.26651429471045	-0.30937574347982	-2.93496606090183
C	2.45947362003966	1.96351876009904	-3.20079328375536
C	4.50243349154990	0.17914142237525	-2.54484202272634
H	3.08102831379807	-1.37394183185864	-3.01336875894512
C	3.67704824187950	2.46458741165790	-2.75902563554753
H	1.65799937031652	2.64311376853141	-3.47135948377205
C	4.68571687248103	1.57263110100775	-2.41029356032900
C	5.81048045779608	-0.54660549671390	-2.22767379791765
H	3.82345782696613	3.53746043064906	-2.66014133836540
C	5.98797560968760	1.81671703386246	-1.81151560155666
C	6.62978999652050	0.58745953671075	-1.62370439333918
C	6.51431474085695	-0.95382640418148	-3.55967492127328
C	5.70776132597001	-1.78280348871796	-1.31007992142837
C	6.58433483817380	2.99341958877923	-1.36802128032794
C	7.86928752407886	0.52773983402383	-1.00256738530911
C	5.88131145105778	-2.16841855676499	-4.23139671476171
H	7.56116324647253	-1.18669872459547	-3.31705017534692
H	6.52621046368013	-0.08605541929935	-4.23027871192608
C	5.15765969657210	-3.04256984089237	-1.98558799536592
H	6.72125053425982	-2.00435854577123	-0.94438651359922
H	5.09341416920841	-1.55129902735284	-0.43542972173060

C	7.81990465243639	2.92163630211576	-0.74048219529575
H	6.08988462193766	3.95368843299795	-1.49216390862698
C	8.48375012761766	1.69955646580665	-0.54085611278560
H	8.35443667051352	-0.43320730063467	-0.86392686659069
C	5.88875111831373	-3.36966177311760	-3.28629346591872
H	6.43147493505371	-2.40024201507660	-5.15201005128613
H	4.85113109366630	-1.93598479264543	-4.53454903666312
H	5.23910068365570	-3.87807839384489	-1.28078191736071
H	4.08545757463147	-2.92783955888294	-2.17926342526403
H	8.28093625142724	3.84181768947939	-0.39360889222482
H	5.43156897040813	-4.24280033560235	-3.76764643804643
H	6.93143504662297	-3.64061365735194	-3.06185072244619
C	0.32310806023979	3.86723422646247	3.63426699608613
F	1.26240880014587	2.99358341919455	4.02514245225311
F	0.85882507759166	4.74153322225614	2.78345526113515
F	-0.13606510721792	4.51943868650078	4.70321183604679
C	1.70912449958895	-4.67211216162720	-0.59310483631940
F	0.71238327854886	-5.52144849463096	-0.31405580666492
F	1.58364259954905	-4.26419469888619	-1.86422066718129
F	2.87590388064842	-5.30749334252930	-0.45204988085993
C	1.13080777241651	9.91464994290425	-0.71238374658352
F	2.31589664615002	10.15230114282818	-0.04056384876317
C	1.32848927533203	10.57046272563008	-2.11068540365170
F	1.70918568165641	11.85087498221600	-2.00859827294694

F	2.28858186454974	9.90417529316956	-2.77440210383756
F	0.20099324368655	10.52504250817644	-2.84316041362732
C	0.03904215829417	10.62162029218353	0.14325936575295
F	0.32673575030370	11.91513776119312	0.34435931255625
F	-1.17111447168770	10.54663858026930	-0.43975947451014
F	-0.03361445124803	10.02055227247999	1.34181494676976
C	-0.88956530044343	5.01998116974411	-7.65861145870652
F	-1.96006071746005	4.38336514564588	-8.25318911182160
C	-1.18919274957985	6.54028886944070	-7.81681723190048
C	0.35490879501380	4.56313395398048	-8.47591039257692
F	-1.35386377762013	6.88017838424989	-9.10275416252247
F	-2.32302049875702	6.83035787402142	-7.15872748793361
F	-0.20153706590207	7.29673957189644	-7.30529967729764
F	0.16099374941389	4.69988581688919	-9.79147658008967
F	1.45422717971071	5.26093019209118	-8.13581707917961
F	0.59508152955946	3.26065373397314	-8.22585607052357
C	-2.90373663815442	0.52356013748062	-8.62634969656851
F	-2.14206254941599	1.61028213225385	-9.00759353150197
C	-2.78415587120061	-0.48323212181792	-9.80828564145642
C	-4.34531414728506	1.08709482316385	-8.45638645578047
F	-3.18113742816859	0.06321440657545	-10.96599929890626
F	-1.49959730706761	-0.85088133147456	-9.93746211129382
F	-3.51909389424819	-1.58958003924861	-9.59355184132357
F	-4.72868497390504	1.79996232511973	-9.52072050758151

F	-5.24315474079025	0.10544674446439	-8.25843983406677
F	-4.37561927344091	1.90160857118603	-7.38157032163997
C	-0.99984457603516	-7.97918881367641	-6.00184489521118
F	-0.39059396456557	-7.78129127042349	-7.22727583903515
C	-0.09861729157662	-9.01802929351190	-5.27338270530917
C	-2.41338684118484	-8.53046308942182	-6.34714426589765
F	0.08861422889568	-10.11910198781245	-6.01452069069021
F	1.10277230433425	-8.46530001375044	-5.03983254125976
F	-0.62777401344541	-9.39297788196557	-4.09401368108071
F	-2.34741155328995	-9.60873700287684	-7.14000058944141
F	-3.09924857496269	-8.86780642854725	-5.23918101844895
F	-3.10529489630895	-7.57910146767616	-6.99641041100130
C	0.67453232991037	0.30511937327006	5.59298592899187
H	0.78221094039386	-0.53259657552768	6.29492684052357
H	-0.35317769255410	0.66427581390006	5.66248531107136
H	1.36686386117734	1.08974638477444	5.91268537161985
C	1.01774807199572	-0.19742802868106	4.23485259517330
C	0.06794667933915	-0.54817722971628	3.29082239794443
H	0.47678087990836	-1.07745024675938	2.42536992191992
C	2.45920539005225	-0.23109433408695	3.87949054216780
H	2.90692243999036	0.76853537266290	3.96424950457609
H	2.98893950094831	-0.86861095202532	4.60133849163692
H	2.64162881970717	-0.62071870372481	2.87579626641502
C	-1.36174025690792	-0.92430876038267	3.59473532999656

H	-1.78462598748501	-0.31643207320678	4.39791364279532
H	-1.97279261933148	-0.74461519234602	2.70131286571725
C	-1.45000037256322	-2.42556918779503	3.95777509552072
H	-1.01615994486741	-3.01002291319646	3.13710923723145
H	-2.51302615525588	-2.68135933689282	4.01602276507345
C	-0.78466941517007	-2.74893480144413	5.26677660547860
C	0.44577607542545	-3.27983631180947	5.28252508447073
H	0.89644261494230	-3.49104192647185	4.31378426309974
C	-1.60171911419907	-2.41709106216495	6.48150595852289
H	-2.49385547425661	-3.05825328528291	6.51843932969021
H	-1.05659899571924	-2.53285401793566	7.42071608020131
H	-1.97362298336281	-1.38671471674730	6.43142536717540
C	1.27986926830274	-3.63781876179657	6.47027143028505
H	2.17789569059684	-3.00743310690719	6.52118934949306
H	0.74207300897762	-3.54730437239612	7.41696438476226
H	1.64957128053978	-4.66652271511122	6.38366342998939
O	-3.73292323404132	0.98607071963218	5.55752206693335
H	-3.16727255296767	1.64035606161108	5.10305937535359
C	-4.18130708727247	1.46829482920009	6.77824366759489
C	-5.03868755144203	0.31801939280852	7.40098160759906
C	-2.96340032337046	1.79912536961763	7.71158536533627
C	-5.06481036663155	2.74795315523719	6.57681841566583
F	-5.94346262824303	2.56232150294114	5.58286346037372
F	-4.27881466325545	3.78855358873470	6.23224288304236

F	-5.74826758958718	3.08030466264602	7.68217367422021
F	-3.30684432305865	2.55256257223761	8.76530241786995
F	-2.39424515388035	0.66984394967250	8.16783755042732
F	-2.02626893179164	2.46304508050642	7.00784667481464
F	-5.26288937096143	0.54100069892807	8.70761608353007
F	-6.22947843049714	0.22000839719483	6.78365090594514
F	-4.42143709574318	-0.86102456232653	7.27301594584973
O	3.45809204774094	-3.64523295623631	3.98887665172211
H	2.93359572114802	-3.76263789570781	3.16075913812887
C	4.76481066773535	-4.06468519494311	3.82608260745574
C	4.83302344950006	-5.63065485564903	3.80630343521462
C	5.37845277278256	-3.48957074107035	2.50245346744797
C	5.54083023307862	-3.50077731971065	5.05937715923661
F	4.85192957111491	-3.70715603240008	6.18960502978699
F	5.72255003330581	-2.17396718986617	4.93191883070340
F	6.74780760176169	-4.07865530094828	5.19324786939681
F	4.94553210379955	-2.23672413632575	2.30823750897476
F	6.72484615445312	-3.47462948908128	2.52018088733802
F	4.99027663519082	-4.22350794603535	1.44361641730193
F	6.01970372111662	-6.08206150190795	3.36031111016388
F	4.63148703440629	-6.13234700812041	5.03664152911159
F	3.86952311479208	-6.11641055953280	3.00764718358213
C	9.66948806729650	0.12826613476555	5.66445298827488
C	9.89033975672931	1.18691822185961	6.75108367429159

H	10.15748201324851	2.15875263635171	6.32133179535833
H	9.00166364738412	1.31484072936341	7.37877331321209
H	10.71352750413950	0.87555686736696	7.40258358578836
C	9.33903978584466	-1.21060896684363	6.35325967103040
H	8.40790854594956	-1.13020422671152	6.92459438299343
H	9.21622906914784	-2.02127682920707	5.62880080687716
H	10.14437997130732	-1.49141414877024	7.04191089302886
C	10.97614352557479	-0.01399055703485	4.86012941976880
H	10.90560514142287	-0.80137706922923	4.10382222228900
H	11.21729219738230	0.92383802773891	4.34787448494076
H	11.80659613401967	-0.26544333122767	5.53002244073693
C	-5.17049534724448	-5.44750111892703	8.09899770200981
C	-4.46331023178156	-6.74655189687255	8.50333380000599
C	-4.64984305333305	-4.31545240396321	9.00605415801622
C	-6.68328265820704	-5.63202476691372	8.32630798225573
H	-4.81085177664254	-7.59966503810373	7.91031704617218
H	-3.37601661893653	-6.66658294682037	8.39558948810960
H	-4.67640070723960	-6.96506133086374	9.55496847783147
H	-3.57522577360555	-4.16384439016887	8.85620516650497
H	-5.15321182253625	-3.36587660282134	8.80108514270372
H	-4.81923903685241	-4.56674547016332	10.05957590973347
H	-7.24039234565167	-4.71414274536755	8.11590431927649
H	-7.07766164150573	-6.42182840375030	7.67786888592106
H	-6.87746484875359	-5.91166793993792	9.36830395406428

C	9.85541063423295	1.69764127385195	0.13882237201841
C	10.86021410699467	2.42478457335461	-0.77649792009683
C	9.77487922461887	2.42962231927867	1.49252803926093
C	10.37635427629248	0.27943900529378	0.39990725281511
H	10.93735014996350	1.92008608549303	-1.74565083599427
H	10.55712446597771	3.46047317482099	-0.96019631505787
H	11.85409053799157	2.43877771020225	-0.31361162303418
H	9.03614558275182	1.95732210950068	2.14731895580642
H	10.75017006356842	2.39463716592744	1.99203210854768
H	9.50359626384573	3.48316020092356	1.37423067388222
H	9.69410768588985	-0.28251035669993	1.04681825239507
H	10.52426807652689	-0.28078612494881	-0.52981500213515
H	11.34550459786599	0.33616730703649	0.90692919508278
C	-9.18170273171492	-3.07834576671639	2.76643934245773
C	-10.59419035420955	-3.23076230663748	2.16884196719876
C	-8.62241045717161	-4.47036906819792	3.11995422677742
C	-9.29245040962103	-2.26825767452843	4.06399403053277
H	-11.01414181380204	-2.25205807137849	1.91238405982138
H	-10.58275876090207	-3.84062273239385	1.26000781809478
H	-11.26099599518863	-3.71404335795337	2.89251292203903
H	-8.59243673874468	-5.13325639526035	2.25033077534136
H	-7.60659048565852	-4.39472916066004	3.52194543644066
H	-9.25644795895436	-4.94515013493837	3.87758498109861
H	-8.31453760543928	-2.12935167836021	4.53869644366135

H	-9.74213799162479	-1.28379584528189	3.89527288015508
H	-9.93140791339449	-2.80447928550601	4.77318079170381

II

O	1.19174827580399	-2.41600946735500	-1.47878462348572
O	-1.02888389619382	-1.20354183858100	-1.69914643988321
O	0.24615887841639	2.29414563861801	-1.05202729434381
O	2.46691469499188	1.52458587515605	-0.05041759291375
P	0.87575414092302	1.32298194077608	0.08876542557821
P	0.11721828383223	-1.55865318663199	-0.62318950968841
S	-0.85871656222388	2.71221138407356	1.90198512907910
S	-0.09048900583240	-3.88300348881210	1.15277507053455
O	-2.03108893596200	2.52134779504036	1.03352967356641
O	-1.07325004867947	2.57335403567376	3.37093785578371
O	0.83161379239756	-4.73312446098567	0.40047713441001
O	0.15169790478646	-3.70456243784106	2.61683255008510
N	-0.49769604545644	-2.49126747213328	0.52143612754779
N	0.44780258210878	1.91631229933807	1.51887994298927
N	0.69257555094730	-0.21027285820266	-0.10551506837742
H	-4.31434487335039	-2.40089421503379	5.00664758636801
C	-1.59818864975179	-2.27248858213444	-2.38852187957252
C	1.45320719443276	-2.04693691768363	-2.80069206645496
C	0.87747542526659	2.44829950102822	-2.28332713780736
C	2.91147995294789	2.84129144808902	0.01403436514339

C	-2.92909121453234	-2.65579268620939	-2.06719622104904
C	3.46109741330984	3.31011034177940	1.23576470553263
C	2.72488554000675	-1.48963936793176	-3.10599956601285
C	0.14269741253081	2.06761471013822	-3.43970703476024
C	-1.13697101984438	-5.89064342119785	-5.56203309826363
C	-0.58836519816468	-4.80300411042785	-4.92980066548321
C	-1.34839128573991	-4.01929421122605	-4.03343179492576
C	-2.69115599905554	-4.41237994580251	-3.75970746360679
C	-3.24200581039084	-5.52397967330601	-4.43868186732531
C	-2.48460088237971	-6.24836613414631	-5.33101426692502
H	-0.53157816410443	-6.48013893408761	-6.23929577540188
H	0.44913114846049	-4.54564382932772	-5.11454946699476
C	-0.83322128476765	-2.86453730048968	-3.37365325721463
C	-3.45274278891171	-3.69618962634809	-2.81490991314055
H	-4.27016035049056	-5.79267653504088	-4.22389432634857
H	-4.48859750311358	-3.98760081440698	-2.66946865331424
C	1.30740378034282	-1.07294245245246	-7.74332252442847
C	2.27050307386912	-0.96082934739428	-6.76966122583914
C	1.99532611378857	-1.32609071954882	-5.43066079655668
C	0.71941609862625	-1.86704749670939	-5.09644463076217
C	-0.26312808025170	-1.92362887529656	-6.11461046289376
C	0.01474593531237	-1.53738804215344	-7.40072740405670
H	3.94060575771245	-0.75953537866529	-4.69764435667841
H	3.25850437692661	-0.58603165014821	-7.01169743526802

C	2.96693193373117	-1.15879831424698	-4.42484693352488
C	0.47234653730134	-2.27480398336440	-3.74889981783795
H	-1.26117444985927	-2.27378300551318	-5.87662305895825
H	-0.76659515928138	-1.59572653164153	-8.14808035313751
C	4.84157155754827	3.31207021347736	-4.90523599524138
C	4.18503854962044	3.38217907206142	-3.70287893703062
C	2.83182429738051	2.98922401585927	-3.57946646560548
C	2.16408648714731	2.48677899862152	-4.73632147169040
C	2.85356292769187	2.44620504214881	-5.96969352811330
C	4.16491024947055	2.85107547770097	-6.06005748668780
H	5.88079298863603	3.61159258318804	-4.95665036313806
H	4.71815846809062	3.73055170117508	-2.82436681155321
C	2.14110500123523	3.00966803802082	-2.33159555427993
C	0.81516804601078	2.07832229521816	-4.64385477734458
H	2.32670171551048	2.08421391621282	-6.84493858917523
H	0.28651983138717	1.79296006220977	-5.55074468350829
C	3.89906428038522	7.68728755879175	-0.84152492386987
C	4.09360181812918	6.83813321895294	0.22136561017433
C	3.70364075718879	5.48120749307364	0.14928834569984
C	3.12806239636451	4.98258263022410	-1.05970925558744
C	2.91062632200414	5.89034750169026	-2.12155562427985
C	3.28281866186418	7.20731538909110	-2.02169876553298
H	4.31200509127623	5.02823104098424	2.16994371420010
H	4.54483494650418	7.19954490652382	1.13866975268006

H	2.43382226733983	5.54165958757662	-3.03101360651788
H	3.09417719193665	7.87366986393706	-2.85434722699920
C	3.86377300265685	4.62849862346047	1.26368706651079
C	2.75494363950930	3.60600159398263	-1.11948861463242
C	-1.30263386988495	1.76620535627555	-3.36805848633256
C	-2.16811426280196	2.65224571373751	-2.69797772046193
C	-1.83073985511590	0.65571914035952	-4.03178867234795
C	-3.52969527830261	2.40386674903883	-2.67275230347476
H	-1.74838718834104	3.52682699886735	-2.21600326216266
C	-3.18851352560446	0.36607643901042	-3.96731744790255
H	-1.15836766719043	-0.01282745429905	-4.56017328823443
C	-4.02986820559903	1.22223530094163	-3.26389297720390
H	-3.57462326206070	-0.53726171653800	-4.43334745872621
C	-5.82279877661091	2.21995098054413	-2.18918466507398
C	-5.44027444745879	1.09437190281630	-2.92682220591074
C	-7.10200762609894	2.32105690475165	-1.66455544092386
C	-6.34656537491062	0.05814660055288	-3.13565222141880
C	-8.02674493392671	1.28649621884042	-1.85412641446164
H	-7.37820394689254	3.20002214423480	-1.09185722828167
C	-7.62321525978427	0.16537640891915	-2.59919690205060
H	-6.05918507349259	-0.83402531379269	-3.68670724933603
H	-8.32311099975819	-0.64964875002243	-2.76010029191391
C	-4.68740942981619	3.22878902256214	-2.10907188056638
C	-5.02261919201694	4.38829073864260	-3.09719228855568

C	-4.50969018006965	3.81193535423607	-0.69331942095616
C	-4.02352127480544	5.54022811372294	-3.03259491938741
H	-6.02090328301302	4.76494002712095	-2.83104935721899
H	-5.09427707441797	3.97834965282303	-4.11194251223109
C	-3.56740932826821	5.01462094237413	-0.61064527286955
H	-5.50258416599920	4.13438269123290	-0.34799275505475
H	-4.17051270062233	3.03204766957853	-0.00724343739657
C	-3.94091121378372	6.10534819968704	-1.61414519549589
H	-4.32648074371776	6.32232436997868	-3.73995438884311
H	-3.03255662650496	5.19389463123797	-3.35660916958315
H	-3.59643189490116	5.41217886615477	0.41052736568790
H	-2.53254738110434	4.69509044759122	-0.77448509811702
H	-3.21638240089565	6.92756626979844	-1.56782014426999
H	-4.91973607390156	6.52788292416343	-1.34159852503483
C	3.55220577196699	2.43438883199882	2.42004906572078
C	3.17154508226270	2.92328721399367	3.68201168347311
C	4.00401853777279	1.11580074138095	2.29400207771215
C	3.21404859438106	2.08929590763171	4.78945332147707
H	2.80300827325751	3.93963815856158	3.75062921844708
C	4.07205771634677	0.27864062414157	3.39616667868973
H	4.31248829193579	0.74520304815933	1.32553834426133
C	3.65923436190893	0.76026538079382	4.63244933365988
C	2.83404243087446	2.34505845648366	6.24793972504278
H	4.44829314996855	-0.73459288806396	3.28607383689297

C	3.55233422340024	0.07071281445480	5.90802383115580
C	3.03874824944700	0.96341860695725	6.85386428688891
C	1.37885043957255	2.83394434350915	6.44422890571362
C	3.81646281182343	3.33372591819105	6.93261374130791
C	3.84884546917464	-1.24313419046863	6.26913011892117
C	2.81229913280584	0.55350114556449	8.16041885852459
C	1.15683861504181	4.30553214815061	6.09018704976226
H	1.11917704566582	2.68839039067784	7.50288793776604
H	0.69947764894102	2.20149275872182	5.85872624074438
C	3.59682927671532	4.79013452857637	6.52919057197350
H	3.67145051025312	3.24515201013206	8.01893557315020
H	4.84392477436124	3.01216711146977	6.72100231036083
C	3.62444412061495	-1.63927627868238	7.58043026643529
H	4.24621432334871	-1.94965279846890	5.54343886772199
C	3.10034009804658	-0.76217236790942	8.54504741249326
H	2.41341294596990	1.25835563303885	8.88317960310844
C	2.15496417263512	5.21874977983437	6.80245137127765
H	0.13322342987528	4.58385482262995	6.36393289745626
H	1.22085790174033	4.44609317560256	5.00573569563409
H	4.29552410803757	5.42712901646202	7.08573660434167
H	3.83633283544559	4.92699717865994	5.46644799775906
H	3.85937382685432	-2.66067364433634	7.86002398806045
H	1.99876260245151	6.26023342900680	6.49610209224201
H	1.97161342117158	5.17726347021599	7.88681889821721

C	-3.74415805938453	-2.00525877376726	-1.02274448537493
C	-4.75443638893813	-2.76494252256863	-0.39424540414247
C	-3.60383026034111	-0.65100186307848	-0.67756676998309
C	-5.63649476352167	-2.17293587808697	0.49013293122543
H	-4.81196063294703	-3.82542226429181	-0.60311229228127
C	-4.47686747628276	-0.05154913397440	0.21734888875071
H	-2.84196495009911	-0.03926213404404	-1.13893537596263
C	-5.50439244758627	-0.79942764407711	0.77452468331027
C	-6.80944598901105	-2.76298748158066	1.27265998978051
H	-4.35643333774220	0.99898275104674	0.46033622522848
C	-6.54961427986316	-0.40621271972346	1.70115341183538
C	-7.31878807716400	-1.53150604485379	2.01093340085381
C	-6.37572402829919	-3.85452383258928	2.28244652928795
C	-7.92534498427217	-3.31391220743235	0.34860044053541
C	-6.86901056115411	0.83706229875701	2.23986067321913
C	-8.40717564598744	-1.42608886995762	2.86559401705068
C	-6.05368134690520	-5.20360217092078	1.63820598544754
H	-7.20323443146757	-3.99707859475871	2.99250696607445
H	-5.52132320771504	-3.48522945025440	2.86332851570503
C	-7.60235527808400	-4.66854649465889	-0.28140033182177
H	-8.83549655031760	-3.42178257958030	0.95573775372242
H	-8.14314628641415	-2.56354005193923	-0.42025433315266
C	-7.96290407390098	0.93280754060871	3.08646412308677
H	-6.28070198451354	1.71757654078252	1.99739764087834

C	-8.74846985223620	-0.18461551136249	3.41782471677075
H	-8.99818784352673	-2.30720261160345	3.09543040298461
C	-7.21986516155628	-5.69956033910144	0.78203745134306
H	-5.82403234900421	-5.93239433699500	2.42563121019361
H	-5.14802939011738	-5.12438342022141	1.02446508144898
H	-8.47440848175640	-5.01637741054140	-0.84921021119553
H	-6.78762143183391	-4.56469808410607	-1.01004597370514
H	-8.21069233946654	1.90424602781801	3.50404224609335
H	-6.96881422845525	-6.65867088287853	0.31307243130078
H	-8.08902335822748	-5.88141186733278	1.43182676480602
C	3.76665974891908	-1.24911254240870	-2.08752563271743
C	4.11876560654528	-2.23178021679139	-1.14573144743568
C	4.44663583473831	-0.02479893998574	-2.09955376931644
C	5.10909938005378	-1.96767499065510	-0.21404213752494
H	3.60565172280223	-3.18486930361235	-1.16587928019447
C	5.42547134081795	0.25797395264953	-1.15701228860954
H	4.16605914502654	0.73079478718701	-2.82746657584315
C	5.73635670694848	-0.70313256271920	-0.20024929958847
C	5.66470407311925	-2.83573548680693	0.91275607516005
H	5.90811887264756	1.23243371955704	-1.14886233875424
C	6.57552122271186	-0.61072510617000	0.98732594738253
C	6.46985062499828	-1.81136221556119	1.69640511173751
C	6.67295549591740	-3.88578054449392	0.35774195496282
C	4.59536819112694	-3.53602385969244	1.77682864276291

C	7.29647717295909	0.45384187524894	1.52378899541502
C	7.05817341472959	-1.95156118533396	2.94499433800441
C	6.00012032360576	-5.07400792787514	-0.32195564120411
H	7.26262837647306	-4.25429797569130	1.20946113897480
H	7.37069863963419	-3.37607162126262	-0.31780493485478
C	3.97628036599611	-4.77819634288716	1.13450341817840
H	5.06955821057119	-3.83968327901256	2.71987345341359
H	3.81276716281642	-2.81114295884468	2.03841144092512
C	7.86738232130090	0.30971880862832	2.77983616917209
H	7.38326819309455	1.39770871944681	0.99128606510740
C	7.74672728999425	-0.87765233891187	3.52191927095646
H	6.95645016688401	-2.88862526532865	3.48307289189616
C	5.03610475453921	-5.76123968023007	0.64498645888236
H	6.76903430306190	-5.77566497222483	-0.66845679416291
H	5.45619164771945	-4.73796639580776	-1.21551978268361
H	3.32096688594110	-5.25943903011510	1.86589027883514
H	3.32170427427060	-4.50149252161294	0.30358857677679
H	8.40330733771730	1.15319125848607	3.20492574382838
H	4.55515459680748	-6.62331078735157	0.16749129694086
H	5.60431393489300	-6.14779528309086	1.50501832845742
C	-0.37274227483269	4.54122255988948	1.71140364571660
F	0.69866477223890	4.81803436697543	2.46596622769025
F	-0.07703470477774	4.81146768604289	0.43345694028794
F	-1.38801902199592	5.31706564793792	2.09973871712242

C	-1.76004200218479	-4.77321607889710	1.13250423117069
F	-2.15489252137018	-4.99577475800856	-0.12396356508261
F	-1.64108606516335	-5.94229965830890	1.76804628926306
F	-2.68956071758740	-4.03275226758281	1.75854966895957
C	4.33563380563986	9.13234901386264	-0.71364572441234
F	5.02718243278598	9.32032047096144	0.46891638616854
C	5.33843415360635	9.54440795601572	-1.83206128386511
F	5.92162650324777	10.71897644340104	-1.56129842506084
F	6.30394349924212	8.61520066132085	-1.92913427505214
F	4.72995207347533	9.64612187918478	-3.02934555282049
C	3.11231096882999	10.09035945684191	-0.61966226599199
F	3.49192616233030	11.36883503063159	-0.47370286399205
F	2.33395305050273	10.00640402098246	-1.71266285989587
F	2.37409404383153	9.75013468647264	0.44920992112865
C	4.86022627466710	2.79180610537299	-7.40516148592477
F	3.99418523468827	2.31854602376725	-8.37072799083774
C	5.27719619067888	4.20750146692392	-7.90221615738165
C	6.04319131316867	1.77981564576105	-7.39951280957823
F	5.77066752661647	4.16849157728205	-9.14725659499143
F	4.19613246181583	5.00381109475463	-7.90665894502660
F	6.20531440508284	4.76134570238843	-7.10014927916770
F	6.55084547725608	1.59930773012939	-8.62400638914862
F	7.03616254337207	2.18016765372549	-6.58587459646331
F	5.59751815276150	0.58687742617726	-6.95645348480059

C	1.65333126349588	-0.69611421481139	-9.16937079240366
F	2.95206175542828	-0.23333858928992	-9.24042883687031
C	1.61901240338670	-1.93723731134067	-10.10996254884136
C	0.77911758710572	0.48136183439580	-9.69255723975515
F	2.00191904257124	-1.62430191888667	-11.35600572711900
F	2.46365369944809	-2.86592351390378	-9.63390565524508
F	0.38936679872544	-2.47895212058724	-10.17311591292480
F	1.23140358270847	0.95492036775259	-10.85845380549697
F	-0.50444518361976	0.11259128721492	-9.85581167063870
F	0.81219563932391	1.48866130428830	-8.79716692800433
C	-3.05601642931025	-7.44405631838772	-6.06914778804973
F	-2.11498843552882	-7.96383668609035	-6.93861004546878
C	-3.39122631129602	-8.61297660660494	-5.09813817003065
C	-4.25538429915591	-7.04515095494165	-6.97777900715135
F	-3.77973942628479	-9.71175706772182	-5.76090255903661
F	-2.29420142309219	-8.92094887601099	-4.38845751357782
F	-4.36969929269284	-8.27537459945789	-4.23743570861136
F	-4.63905258121028	-8.06034008186402	-7.76333420701179
F	-5.31995047968167	-6.65319230415300	-6.25193587659903
F	-3.88782845148027	-6.02358100278014	-7.76894832706494
C	-2.49006124358863	-3.04508449047903	6.86685576388972
H	-2.19631523458046	-4.00179889834675	6.42055522471045
H	-3.52611312457913	-3.13855148949588	7.20982529351102
H	-1.86834917521508	-2.87522651583344	7.75102179469339

C	-2.38958023958425	-1.90392797851160	5.84612354753164
C	-3.28647130743370	-2.26019734913087	4.64982846100170
H	-2.95793965219771	-3.22566958894415	4.23833005269629
C	-2.85154905411754	-0.59094835235116	6.49016799856993
H	-3.92502949899706	-0.63853432328423	6.70242005889670
H	-2.33434240723642	-0.40244186558185	7.43611619482250
H	-2.68420233448571	0.27487503998328	5.84032170713243
C	-3.26149134923473	-1.20092114303738	3.55803626819067
H	-3.84118287657892	-1.52886596159811	2.69070763517002
H	-3.72800285217068	-0.27253198534230	3.90399959884879
C	-1.83467717168368	-0.92704210891221	3.10642021370024
H	-1.69637667133961	0.03263659868409	2.58277687699826
H	-1.53314744197902	-1.62186995680362	2.28442659008108
C	-0.74684362337345	-1.11004451260792	4.04455868891376
C	-0.92197132748234	-1.76419581629887	5.35368718885778
H	-0.57871497923387	-2.78781895656475	5.07000669886888
C	0.58794028801819	-0.68351261920565	3.62988493941762
H	0.68443683943574	-0.60820045797243	2.54556013086165
H	0.71819123992322	0.34479002701490	4.02098540278580
H	1.38565855284410	-1.28455880764140	4.07118597178264
C	0.06538573777549	-1.24865877552437	6.41910530390816
H	-0.03001270793119	-0.16734830431634	6.55000042556085
H	-0.15124581691559	-1.72823844830220	7.37357545318668
H	1.10570239097333	-1.47137036276091	6.17938669631552

O	-3.56804582848953	2.23178360834338	4.36839114753968
H	-2.71150627292171	2.49297755510340	3.94765903555716
C	-4.12711568849817	3.28048010294836	5.07527621512861
C	-5.17844332655643	2.64183906383004	6.04048104088164
C	-3.04230223351610	4.04674697553239	5.90810701162606
C	-4.83736981985966	4.27411559392220	4.08951538039935
F	-5.99817148797776	3.75480854923611	3.64484780837924
F	-4.05697442665479	4.48809256617311	3.01981151223496
F	-5.10606776478842	5.46013781135652	4.66155835850528
F	-2.33663198649336	4.87161262843486	5.11211186669559
F	-3.58046515040223	4.77929395312340	6.89632577733069
F	-2.17744765119406	3.17514059059512	6.45356962198495
F	-6.05249480437708	3.55813105431873	6.49295470752171
F	-5.86440758426356	1.67510190204330	5.42024793248907
F	-4.56914249235421	2.08865884674002	7.10644782753056
O	2.23855436967381	-4.50354204218226	4.16456098248236
H	1.44465307195488	-4.39828650452659	3.58698852081780
C	2.16502824301416	-5.63275896089382	4.95725303550168
C	1.88465000954812	-6.91891034626678	4.09875659486517
C	3.56003837444848	-5.76074299819020	5.65089104444170
C	1.04263908684560	-5.46297014752772	6.03915401015357
F	-0.17129229336674	-5.61207688708345	5.46467367894052
F	1.08076784038405	-4.22541897698079	6.56328690764509
F	1.14392602170285	-6.34249087042318	7.04546713187906

F	3.69345866094855	-4.83693346560104	6.62331159647665
F	3.72087037809137	-6.97232384340551	6.20756705981990
F	4.54842108499722	-5.56343346696913	4.77127681055440
F	1.46522961594961	-7.94849997105175	4.85315960036871
F	0.93854997190822	-6.65498279224032	3.18376580252248
F	2.98986454070049	-7.30527865243564	3.44128865040098
C	2.87839341077616	-1.27220392898593	9.97125512491998
C	4.23085225782190	-1.71644724767580	10.56254392235970
H	4.67780407884193	-2.52861910032903	9.98167123124093
H	4.93908763800290	-0.88113193295669	10.57444407930339
H	4.09720993859873	-2.07103573513627	11.59109854795074
C	2.28102443616741	-0.20050676979001	10.89008197324239
H	2.94539484336250	0.66475483285023	10.99029659384540
H	1.30731921100977	0.14827865626361	10.52810268465626
H	2.13207962456164	-0.61994090906949	11.89046708087971
C	1.91486807132685	-2.47466509210167	9.94445184314692
H	0.93798446867209	-2.17855010675329	9.54617075358627
H	2.29659138591557	-3.29182763994039	9.32486409908119
H	1.76627979151707	-2.86237442150840	10.95885614374266
C	-9.94510138121995	-0.00015565612724	4.35246982002447
C	-10.68632185539175	-1.31503736694733	4.62131148726664
C	-9.45750289788847	0.56294350194088	5.70169376123253
C	-10.93875916159611	0.98869259806567	3.71179563205590
H	-11.09002119879677	-1.74909570856787	3.69989189468107

H	-10.03744786130166	-2.05636178031517	5.10076571428160
H	-11.52899812071432	-1.12716173473229	5.29483989731596
H	-8.73557546469379	-0.11780123885003	6.16582862572566
H	-8.97200739409329	1.53645312451785	5.58539068517585
H	-10.30406070107893	0.68846839314858	6.38667732987662
H	-10.48403247568015	1.96960851281614	3.54403144366007
H	-11.28852594797916	0.61104927985119	2.74473598394264
H	-11.80859951202699	1.12734779480050	4.36445082444823
C	8.33679405759304	-0.94253815900947	4.93251471196937
C	9.85579845736364	-0.69640926451584	4.86583906349608
C	7.67478603568900	0.14520768925503	5.80260123060096
C	8.09776891344519	-2.29971710998208	5.60479700743745
H	10.34420069172575	-1.45655410879571	4.24662560119353
H	10.08863043027886	0.28422445486574	4.44014076744227
H	10.29106934748049	-0.73832987013127	5.87109696419594
H	6.59026567407697	-0.00298496815718	5.85038334612194
H	8.07274963530199	0.10590568538102	6.82339885294546
H	7.85903644493861	1.14870411420423	5.40691660201354
H	7.02874652280479	-2.51297309823165	5.71750236482180
H	8.56082505195705	-3.11959974966751	5.04493391803533
H	8.53881943065581	-2.29122018340376	6.60689105950954
C	-9.45353117124540	1.35817821081983	-1.30237357406550
C	-10.43301031103337	1.48823084503806	-2.48478845882361
C	-9.77909830387969	0.08254232650557	-0.50426689109177

C	-9.65778285525209	2.55677484935417	-0.36707257749422
H	-10.22410646667654	2.39511308519544	-3.06255495466792
H	-10.35729908030912	0.63282350335001	-3.16386915082797
H	-11.46547378651416	1.54180826120789	-2.11929704772293
H	-9.75152359429745	-0.81256871071363	-1.13269494043699
H	-9.06943021863615	-0.05330974439464	0.31840836439930
H	-10.78780724891898	0.15447157256823	-0.08061226398394
H	-8.97736200742942	2.51280024606510	0.49087363458787
H	-9.51465299605806	3.51132611544251	-0.88475912858286
H	-10.68268093682742	2.54425439764001	0.01893353640268

TS2

O	1.22048384099527	-2.37440756914799	-1.30053192443419
O	-1.04487017723460	-1.23682619724989	-1.47260775029321
O	0.20573778225908	2.30449569270013	-0.95096050570320
O	2.43332169927124	1.56726293447044	0.06730698131023
P	0.84261502750469	1.38978442930453	0.22776332059157
P	0.15944618021514	-1.52190488848993	-0.44978399508630
S	-0.92735413204700	2.78676809827398	1.98312382228251
S	0.06862696447021	-4.03596762590274	1.14129106542553
O	-2.06174380954049	2.56233142542736	1.07299219626387
O	-1.17729319616953	2.67108530551272	3.44142666214590
O	1.11484280864823	-4.63214910696812	0.31998673357144
O	0.13778051846338	-4.10869674982710	2.61665598929224

N	-0.31276404195407	-2.48820670099019	0.79531750730405
N	0.41055955589851	2.00878342746507	1.63659055889827
N	0.64396703531684	-0.15915891008215	0.08781489221072
H	-3.98815990334435	-2.66110128116520	4.25457528880868
C	-1.61331456261455	-2.30303745576450	-2.16895092744791
C	1.44714114727816	-2.04920669824989	-2.64452350110921
C	0.83640485884780	2.43640221605579	-2.18497706551545
C	2.88037638759489	2.88913949631002	0.09601415035242
C	-2.95958005246360	-2.65184016750627	-1.86932269818535
C	3.44161359466794	3.38321868000370	1.30151803195776
C	2.71225677133014	-1.50193755151940	-2.98576386670472
C	0.10164489223782	2.02714794984500	-3.33080821665635
C	-1.15871052000067	-5.96385049935127	-5.28765167159907
C	-0.60706624464805	-4.87019019088823	-4.66863905424904
C	-1.36914035949639	-4.06635783511356	-3.79258931634141
C	-2.71871979562462	-4.44106043858960	-3.52725314052486
C	-3.27223649351191	-5.55930941860655	-4.19305783542401
C	-2.51183716782182	-6.30530530302319	-5.06480406797206
H	-0.55193911669310	-6.57013836377261	-5.94863966128015
H	0.43428003256519	-4.62404569097501	-4.84722157066171
C	-0.84820815600929	-2.90435551020330	-3.14874211939340
C	-3.48664814904111	-3.69555609089541	-2.61063033865772
H	-4.30523702926437	-5.81477661096820	-3.98565804258858
H	-4.53141617849327	-3.96263376135765	-2.48435217825404

C	1.18574884445599	-1.16802807628585	-7.59335746887753
C	2.16986355344175	-1.03847417561957	-6.64359667056818
C	1.92784902728743	-1.38903156378923	-5.29435836281850
C	0.66340705132546	-1.93337438911365	-4.92289189602280
C	-0.33923518289930	-2.01508975423470	-5.91933175118766
C	-0.09343218307284	-1.64197924400995	-7.21600242696339
H	3.88908918008416	-0.80270596789281	-4.61851779533990
H	3.14877475855987	-0.65699485444082	-6.91112241034648
C	2.92307884786712	-1.19879758906372	-4.31592227668693
C	0.44633439111371	-2.31245982283184	-3.55986447969660
H	-1.32949481344110	-2.36926087431854	-5.65678761870111
H	-0.89111785452319	-1.71632125105046	-7.94433283961656
C	4.78403121869485	3.28500119079936	-4.83506293583302
C	4.12872960287754	3.37824742670389	-3.63343356792758
C	2.78131781315311	2.97005858768703	-3.49700671596541
C	2.11640143312612	2.43430414513106	-4.64050778861069
C	2.80436025755807	2.36987159433008	-5.87343810762601
C	4.11166195331328	2.78446787725919	-5.97587934786557
H	5.81923910105841	3.59619302417066	-4.89656646641203
H	4.65864626396931	3.75677162543202	-2.76552193708519
C	2.09397238647949	3.00777653962166	-2.24770181708849
C	0.77110791257834	2.01693650593680	-4.53651970683474
H	2.27987992941956	1.98118076888050	-6.73853929687079
H	0.24118447037040	1.71204489651604	-5.43626430232015

C	3.75771377751424	7.74122261389813	-0.83568549287278
C	3.97582750094738	6.91282275684653	0.23899340228652
C	3.62048987894222	5.54552476980975	0.18736634875456
C	3.05388614712598	5.01570387314591	-1.01251512496558
C	2.81430805817492	5.90133162296485	-2.08810368631730
C	3.15360175935914	7.22843794283226	-2.00820667264226
H	4.25998309485872	5.13011483795984	2.20824507122698
H	4.41894330266204	7.29904013224242	1.15010642587346
H	2.34477067630312	5.52791471313363	-2.99154314568232
H	2.94758499843013	7.87758270536532	-2.85025622095871
C	3.81130730005643	4.71094781567431	1.31101227848113
C	2.70471636768692	3.63178061253054	-1.04874648931107
C	-1.34350653447009	1.72743963864392	-3.25331683496932
C	-2.21000766131892	2.63637337895536	-2.61566746318409
C	-1.87085565862127	0.60375906700949	-3.89524616199868
C	-3.57480517770148	2.40417122131922	-2.61094559671854
H	-1.78911890131061	3.51788825726638	-2.14735016497979
C	-3.23261169400655	0.33111379402860	-3.84971946670875
H	-1.19684961696173	-0.08140471246885	-4.39948449180742
C	-4.07721127950020	1.21655293456218	-3.18772615737695
H	-3.62152160969006	-0.57789725207030	-4.30229136872163
C	-5.88618340039452	2.27016978510059	-2.19703128008453
C	-5.49887239104627	1.12119666480191	-2.89495593587500
C	-7.18666671089413	2.41269889985366	-1.73715454919526

C	-6.41810396807234	0.10123215494045	-3.12481302797824
C	-8.12593758405207	1.39626226186610	-1.95101745027581
H	-7.47039491392737	3.31172541848969	-1.20001778804691
C	-7.71492004864938	0.24944853198734	-2.65173162355212
H	-6.12809766058671	-0.80676124349389	-3.64789684513009
H	-8.42669470871275	-0.55145447478119	-2.83027403102853
C	-4.73311986667661	3.25748077683110	-2.09277809961041
C	-5.01834733849592	4.41122731612064	-3.10352017284113
C	-4.58378170333844	3.85452119384262	-0.67829857155704
C	-4.00541608993675	5.54991462164160	-3.02208369612007
H	-6.01838461974940	4.80616636491958	-2.87303083425435
H	-5.06557292148996	3.99008437475476	-4.11516787815189
C	-3.62778459985491	5.04547624479554	-0.58356040365691
H	-5.58127540999564	4.19126967239914	-0.36128335249619
H	-4.26914715055626	3.08015186597477	0.02552764410168
C	-3.95745151935633	6.13019193059684	-1.60818453074540
H	-4.27607557201957	6.32733448878766	-3.74743096435355
H	-3.01012878964641	5.18634551624215	-3.31175138933679
H	-3.67873533516215	5.45321622281248	0.43293779930263
H	-2.59363783458685	4.70911998369516	-0.71565885405511
H	-3.22344012818447	6.94310094472325	-1.54921666390987
H	-4.93802675481585	6.56879560170075	-1.36954556582128
C	3.59105782172379	2.51167617500025	2.48195624598635
C	3.18178601887389	2.96072414182868	3.74884219844452

C	4.13322338510872	1.23017440027616	2.33832013134865
C	3.28053025689293	2.11576291707425	4.84477746919221
H	2.74105740997365	3.94733091462808	3.82866165384969
C	4.26178149706169	0.38620219716382	3.42948198250731
H	4.45777955630795	0.89348777829991	1.36190102588237
C	3.81406423689574	0.82177058639234	4.67071998287445
C	2.85810009464959	2.31645200493336	6.30024665684061
H	4.69933862709954	-0.60016308610695	3.30350038960327
C	3.72132983136043	0.09787115904765	5.92838740392832
C	3.13875163329569	0.93791898290998	6.88187698064775
C	1.36775988791344	2.69886833489089	6.47318769442766
C	3.75470160880143	3.35626167912928	7.02399665930705
C	4.06425558129622	-1.21181437981717	6.26136537268254
C	2.89961351962194	0.48254656379006	8.17080081413422
C	1.05038023247445	4.15701721595878	6.13929638795923
H	1.09918143945339	2.51848866111076	7.52404722205139
H	0.74558482552405	2.02960419037034	5.86597319527994
C	3.44200118716466	4.80090492380684	6.63993020331706
H	3.59227202482554	3.23778972244419	8.10500740915856
H	4.80639470972052	3.11079580053254	6.83057241605846
C	3.82187281960693	-1.65572013057998	7.55374816736151
H	4.50638787325006	-1.88077252925497	5.52574438248258
C	3.23609185159358	-0.82900249253883	8.52775161065723
H	2.44524704424751	1.14646060521129	8.89938345811014

C	1.96796816650515	5.12336752388510	6.88902539637688
H	0.00375920071840	4.35615484044196	6.39392504175765
H	1.12703542779143	4.32233877329427	5.05935398047593
H	4.08294687201981	5.47559257828857	7.22114494138404
H	3.69243030636995	4.97157545119895	5.58459256028843
H	4.08848209735665	-2.67564477159468	7.80982476330973
H	1.74672745441725	6.15747955622515	6.59807037848753
H	1.76624141436649	5.04844610142722	7.96834927516163
C	-3.79224556344208	-1.94970900498899	-0.87295984660464
C	-4.84516734193982	-2.66324904851258	-0.25794192301233
C	-3.63783940065216	-0.58504821544196	-0.57721317840403
C	-5.75488628168755	-2.01671860464358	0.55800878164839
H	-4.91698906971299	-3.73002365755010	-0.42826086189230
C	-4.53571232675098	0.06641414866724	0.25428272158902
H	-2.84521038539840	-0.00498345842781	-1.02780719256000
C	-5.60621673537148	-0.63431838725910	0.78924942239300
C	-6.98725504850706	-2.54265845640222	1.29374144047813
H	-4.39804340634862	1.12062728605148	0.46669559982024
C	-6.69847725379426	-0.17414363641478	1.62561369750202
C	-7.51388894676269	-1.26588230963132	1.93541410616710
C	-6.64123964577919	-3.58032380895670	2.38947270683865
C	-8.05232875455712	-3.12757422851116	0.33258720357780
C	-7.02356024510418	1.10106600280272	2.07924612252935
C	-8.64579297689913	-1.09841647946973	2.72068019148388

C	-6.29645099651782	-4.96579450665447	1.84422315927578
H	-7.51836247648944	-3.67274429567535	3.04571376644283
H	-5.82453366816485	-3.18501898466312	3.00473897965517
C	-7.71741962663977	-4.52264862623543	-0.19653927804942
H	-9.00142360689651	-3.18329830022538	0.88443773052048
H	-8.20434687333119	-2.42110042944254	-0.49205243407937
C	-8.16162525183168	1.25923726437241	2.85453766514781
H	-6.40114867257347	1.95631678686155	1.83127267457064
C	-8.98684539579470	0.17408549839126	3.19669449373475
H	-9.26949393852378	-1.95479842099423	2.95733666473367
C	-7.41386824370749	-5.49591355284255	0.94442386615347
H	-6.13047749242866	-5.65135160756837	2.68464635831546
H	-5.35059785755521	-4.93201530131612	1.28901246372641
H	-8.56150587587249	-4.89124801858749	-0.79284809100747
H	-6.86156771000140	-4.47289251232802	-0.88271068069266
H	-8.41127198051845	2.25408654828195	3.21119427735414
H	-7.14966442753237	-6.48270383729088	0.54518374874921
H	-8.32346850661046	-5.63081331407599	1.54851633877218
C	3.77752123918826	-1.24335921221149	-1.99545265278606
C	4.17462471070925	-2.21887508248634	-1.06282339024149
C	4.43783336678475	-0.00924454029829	-2.03325201235077
C	5.19600669067815	-1.93909919180146	-0.16951437550027
H	3.67193400706662	-3.17857946436764	-1.05935126078818
C	5.44189416271682	0.29148490753453	-1.12321979649661

H	4.12364779104963	0.73849166396157	-2.75536922233611
C	5.80141094791092	-0.66333759721556	-0.17731998731981
C	5.81321854221876	-2.79758134361046	0.93343705237355
H	5.90779627666814	1.27401662966913	-1.13179647723131
C	6.67755722858215	-0.55439553007489	0.98193825967685
C	6.61415374997139	-1.75474605814765	1.69626543615654
C	6.84307436947128	-3.80521166768588	0.33710741034762
C	4.79920261612290	-3.54331090143960	1.82495015769929
C	7.39932399988151	0.52215703812089	1.49289249661842
C	7.24118664865523	-1.88232879312865	2.92705439455647
C	6.19924118261096	-5.02080647977564	-0.32103610249266
H	7.47982248774241	-4.14735841960026	1.16549383819103
H	7.49328703008982	-3.26536805945242	-0.36211288372821
C	4.21390839556924	-4.80768217581115	1.19297616042757
H	5.31130839311172	-3.83264395537392	2.75236140551927
H	3.99624281306223	-2.85186747840378	2.11427143416265
C	8.00891913549622	0.39076621042648	2.73193971944409
H	7.45733469186935	1.46560061677855	0.95585146824350
C	7.92693357424987	-0.79508322768153	3.48185393593643
H	7.17005324112693	-2.81949573238502	3.46951693457657
C	5.29824136794795	-5.74820366347814	0.67632807527445
H	6.98568768120130	-5.68807008798596	-0.69509067248276
H	5.61088160870965	-4.70805173511753	-1.19511788749273
H	3.59104711171698	-5.31301124938886	1.93709830556964

H	3.53950423558813	-4.55065655975045	0.37249090115265
H	8.54297338005854	1.24409155624546	3.13911054444377
H	4.84015916132153	-6.63082827747805	0.21394238019414
H	5.90777393268487	-6.10914123401546	1.51888816031269
C	-0.44715645831135	4.61371800008453	1.77185262709009
F	0.61756017732594	4.90576933917179	2.53026165256786
F	-0.14249320643891	4.87068965115906	0.49190816983475
F	-1.47008479897214	5.38803228164527	2.14226014584157
C	-1.50509158375320	-5.01477620553591	0.72645492790486
F	-1.59567477705112	-5.17974060467443	-0.59396985179229
F	-1.42160721526206	-6.20798256546834	1.31602907267128
F	-2.59033978724987	-4.37440235989078	1.17174320075816
C	4.15495886953735	9.19939834792143	-0.72922108118807
F	4.85126306374147	9.42131748059314	0.44435935666424
C	5.13530817868305	9.62638367039895	-1.86207832674786
F	5.69623791035560	10.81449150038776	-1.60498966349704
F	6.11942825672738	8.71753817992110	-1.96638776663502
F	4.50992340330894	9.70827596822928	-3.05241823578573
C	2.90516481315241	10.12309583425996	-0.63699210090982
F	3.24844446190501	11.41535378302563	-0.52671320803579
F	2.11337242776812	9.99009552239234	-1.71552377806781
F	2.19368104612891	9.78590399445616	0.45047749213409
C	4.80719425968613	2.68989108486567	-7.31891288392721
F	3.93974445963167	2.19454784002446	-8.27254379092641

C	5.23004562430386	4.09044721595906	-7.85244917690944
C	5.98515726670257	1.67291503311038	-7.28531947941155
F	5.70779662277254	4.01918666889178	-9.10183145499267
F	4.15601734499470	4.89602389547635	-7.86149909788577
F	6.17308122747520	4.65306613546776	-7.07401788271240
F	6.50868684776406	1.47487310444535	-8.50037874040495
F	6.96818789760320	2.07740018659464	-6.46248239493115
F	5.52603621288401	0.48751453941863	-6.83435287604969
C	1.49300093675806	-0.79221079841773	-9.02891974210324
F	2.79095867050456	-0.33430702408090	-9.13548850741573
C	1.42633180845202	-2.03126079573462	-9.96999082743463
C	0.60917105925865	0.39117247680542	-9.52214968069581
F	1.79242920951378	-1.72220160352078	-11.22128673789202
F	2.26581955242302	-2.97206985563891	-9.50858221806588
F	0.18795691727361	-2.55543174388079	-10.01218272128478
F	1.01471398931604	0.85163497490399	-10.71000527547789
F	-0.68421783005739	0.03621689702370	-9.62441402772412
F	0.69415821781635	1.40292618057154	-8.63501474581652
C	-3.08583396903755	-7.50841111541862	-5.78911969777812
F	-2.14445533733828	-8.04140188115943	-6.64976168437361
C	-3.42589482213414	-8.66275583097758	-4.80256767997356
C	-4.28257963526080	-7.11783590203823	-6.70497867859647
F	-3.82631418382020	-9.76608727404729	-5.44953835303847
F	-2.32734022612659	-8.97000685338169	-4.09438679134903

F	-4.39667277940502	-8.30579612958170	-3.94120446948590
F	-4.66045775358918	-8.13883006415395	-7.48484847606579
F	-5.35050214486730	-6.72351819188384	-5.98526115294735
F	-3.91405610508238	-6.10036060519244	-7.50110137172233
C	-2.45918720218032	-3.33449289776134	6.34412288643372
H	-2.11103735778879	-4.28142574087523	5.91631908197368
H	-3.53156592633883	-3.43020813156545	6.54866007387439
H	-1.95494140452753	-3.18835315963884	7.30463654288409
C	-2.21295860539987	-2.17058093258197	5.37683720818176
C	-2.92326765084683	-2.49231509911199	4.05282492605856
H	-2.52126061455012	-3.43448895944363	3.65427236762810
C	-2.78406935681307	-0.87644185148570	5.97061947378579
H	-3.87878156255720	-0.92111322339757	5.98451047536880
H	-2.44572497817883	-0.72255816893226	6.99997127984029
H	-2.49888857410105	0.00754621312903	5.39102271924138
C	-2.76279664077106	-1.38902455838141	3.01476953495588
H	-3.15818698527551	-1.71416763091654	2.04507953232947
H	-3.34966219768040	-0.50097820409599	3.28695412005083
C	-1.31080559338244	-1.00331777699945	2.86610625602067
H	-1.08125868933768	-0.15194033871735	2.21472012531666
H	-0.75287705887338	-1.81318360895672	1.94810791841406
C	-0.37592712728591	-1.24668759356359	3.85610193882286
C	-0.69089606150517	-2.02587323759194	5.10192982903528
H	-0.29411348013853	-3.03763547657770	4.90508168599968

C	1.02348863910898	-0.79129728836729	3.66704808090768
H	1.22992955188303	-0.49614779514836	2.63812610268800
H	1.20834278900958	0.08810350667956	4.29855287478136
H	1.73860424896483	-1.55581569136334	3.99229867521459
C	0.09364050785657	-1.46336455020915	6.30423062547643
H	-0.11695678343162	-0.40002809591762	6.45278645517754
H	-0.19339736195013	-1.99415641154628	7.21389809078625
H	1.17290919686123	-1.58357569900332	6.18798126376451
O	-3.70595201715124	2.12167898208331	4.29398269308886
H	-2.83512767737946	2.41983053567317	3.93664463756469
C	-4.26028368091943	3.07292285743962	5.12854212215913
C	-5.32527148305932	2.31883236117927	5.99138184226905
C	-3.18301441016048	3.71882687153437	6.06857340205056
C	-4.95189955385002	4.19524960306377	4.27701068348888
F	-6.10875744237163	3.74676297807670	3.75172778346173
F	-4.15725689418926	4.54724216492381	3.25392615988050
F	-5.22638833856467	5.29644050522669	4.99767790793440
F	-2.48526212827147	4.66173866243714	5.40656286531299
F	-3.73353545180444	4.29642871388535	7.15017295069692
F	-2.31097790248738	2.78884576432852	6.48713984624399
F	-6.19915626883691	3.17488939545399	6.55385613050262
F	-6.01280982149331	1.44925818169109	5.24505331713160
F	-4.72660246965824	1.62721205776831	6.97932592327038
O	2.39103868934893	-4.66586188948136	4.18948752519220

H	1.57316238605719	-4.62105484298095	3.64946769453477
C	2.40059246285861	-5.78821231656943	5.00073891203271
C	2.16016427573815	-7.09446608652092	4.16049953325061
C	3.82053313346501	-5.83588861481081	5.65046914914904
C	1.30247275379130	-5.66041036082941	6.11330865565977
F	0.08490712182201	-5.87233805869335	5.56935515841943
F	1.29898718250366	-4.42247659294714	6.62907169925378
F	1.47413741053579	-6.53217013570072	7.11792905234562
F	3.94003224614288	-4.89378027814046	6.60460475566312
F	4.05445759491538	-7.03147086466506	6.21599168164969
F	4.77099302136866	-5.60603882304994	4.73661760047606
F	1.81061014592494	-8.13707227330194	4.92958294698360
F	1.17718556229108	-6.88233926733745	3.26793704647495
F	3.26335652044712	-7.43175627360243	3.47263247736858
C	2.98709025866200	-1.39096850823114	9.92931055647462
C	4.32809763393199	-1.83906155390425	10.54275388705721
H	4.80186498435117	-2.62618608869991	9.94881608351576
H	5.02507167811150	-0.99640002234493	10.60384329212838
H	4.16953616603917	-2.23060545332902	11.55422755382941
C	2.35168086218401	-0.35903238530666	10.86831695954385
H	2.99600693030475	0.51678347448890	11.00245876347073
H	1.37652964903368	-0.02121664197008	10.50039699004212
H	2.19569903497679	-0.80990545545272	11.85385165965600
C	2.03948068428629	-2.60221505629432	9.83224293100122

H	1.07618347761628	-2.30497993975070	9.40363719380366
H	2.45443713128531	-3.39612430099096	9.20367282033129
H	1.85788925378662	-3.02102292923940	10.82888795246572
C	-10.22094835877412	0.42501552966832	4.06469316920017
C	-11.00562331857444	-0.86053607297134	4.35156802897722
C	-9.78018649250561	1.03113488756886	5.41168581250701
C	-11.15923706683173	1.40964870084394	3.33982538847356
H	-11.37974806033049	-1.32206825960217	3.43089622020774
H	-10.39568532372058	-1.59612041806871	4.88748030459939
H	-11.87136478310162	-0.62735782153410	4.98020860556551
H	-9.10275972773272	0.35009540892193	5.93798898489834
H	-9.25856959376612	1.98357371111111	5.27866680960280
H	-10.65404028101963	1.21100232323987	6.04857514280328
H	-10.67454275396732	2.37378235785191	3.15877809310111
H	-11.47042849789380	1.00325413666382	2.37127544474247
H	-12.05622583772250	1.59205542054551	3.94307436939214
C	8.54905474013923	-0.84307036229173	4.87885604116130
C	10.06070674154365	-0.56553022962455	4.77954709258592
C	7.88201358942831	0.23364476435721	5.75884527779048
C	8.35211125851253	-2.20269935532362	5.55965923610149
H	10.55145551621313	-1.31418612891247	4.14828870900569
H	10.26487251374982	0.42092262273579	4.35252635474981
H	10.51778374073082	-0.60092847696675	5.77524875964545
H	6.80286892960908	0.05991153868665	5.83447294977854

H	8.30558642300236	0.20976557885887	6.76968204659652
H	8.03194882915228	1.23878411332924	5.35326661905339
H	7.29022448802148	-2.43672895083131	5.69604294967165
H	8.81970243233098	-3.01488839700896	4.99223687952820
H	8.81404970484322	-2.18243983238831	6.55209556604482
C	-9.57568457572678	1.50901794960012	-1.47181700170329
C	-10.49756768402651	1.59362054548780	-2.70392423059088
C	-9.95182902490130	0.27387824099882	-0.63358101453655
C	-9.80856172662604	2.75081015537975	-0.60250138673396
H	-10.24529191276111	2.46495832261620	-3.31798528319265
H	-10.40781916367923	0.70186171738489	-3.33244693562981
H	-11.54408056776075	1.68367658845890	-2.38938126980229
H	-9.87192013253159	-0.65269613401823	-1.20980216036708
H	-9.30347677017351	0.18829262646710	0.24423793985678
H	-10.98932690325216	0.35901382982343	-0.28901942089596
H	-9.16897587601331	2.74048718253739	0.28751832947315
H	-9.63031976375893	3.67885416957292	-1.15656459661615
H	-10.84984968257366	2.76519665938523	-0.26313790320054

III

O	1.19256481046212	-2.25007417906997	-1.35791195925010
O	-1.07410133753478	-1.14235980222491	-1.58955302722588
O	0.18973918345665	2.37514647631068	-1.01594385727432
O	2.39932007646963	1.59002924128527	0.01196559447529
P	0.80593293841478	1.49027010103144	0.19510338763655

P	0.11511081444616	-1.39055088183916	-0.55461145879044
S	-0.92282909179528	2.91832301350572	1.95936889392385
S	-0.05713352360516	-4.07484284723994	0.95813835922447
O	-2.06931668544047	2.66559781687681	1.07269828318537
O	-1.13646174061909	2.83948846811852	3.42196637527148
O	0.70120869978318	-4.67416858813191	-0.12572362344045
O	0.31295211407525	-4.17430944883851	2.37118541573927
N	-0.35508431624024	-2.43902422350086	0.67698458603291
N	0.41802480031624	2.14997063780301	1.58736302236698
N	0.52093266900190	-0.06034661540389	0.08731574405692
H	-4.17590048367757	-2.48215588408039	4.62104376212866
C	-1.61669078027281	-2.21908022809910	-2.29826538815309
C	1.45314942783417	-1.94321997117354	-2.70792835491105
C	0.82823323862405	2.48464657195191	-2.24869399347912
C	2.88849392003948	2.90197469373883	0.03183577966746
C	-2.96235617618186	-2.58078151810785	-2.01891348091396
C	3.46221155544982	3.38981523085200	1.23342243761608
C	2.73107660961944	-1.40992507143464	-3.01993987480644
C	0.08762969035927	2.08938609002335	-3.39668021908922
C	-1.08223547296570	-5.85320474218343	-5.43245304360908
C	-0.54569850515505	-4.76478904914875	-4.79123438158902
C	-1.32920547602778	-3.96809144329265	-3.92783768188803
C	-2.68475018821801	-4.34525505822100	-3.69760823234764
C	-3.22201563393822	-5.45766000766325	-4.38676013317988

C	-2.44066488975590	-6.19601193013978	-5.24596037400173
H	-0.45949441966282	-6.45447858300016	-6.08308284412466
H	0.49967150774653	-4.51776640688337	-4.94285874100722
C	-0.82723236916090	-2.80841348612674	-3.26424653456798
C	-3.47280291012430	-3.61335462129989	-2.78771956376523
H	-4.25940220827569	-5.71519862087497	-4.20568426743020
H	-4.51739459126538	-3.88882320242960	-2.67881268852678
C	1.31530461793644	-1.10804083007877	-7.66544898069307
C	2.27591355014867	-0.96816646960409	-6.69344313864703
C	1.99874599327905	-1.29827540802287	-5.34554534650810
C	0.72287223751691	-1.83294736018936	-5.00076406810181
C	-0.25849351140794	-1.91438824731211	-6.01784686999365
C	0.02172378669640	-1.56119326742783	-7.31275273184786
H	3.95045113290560	-0.72641815270191	-4.62835765057176
H	3.26349970001927	-0.59807947379938	-6.94310815085766
C	2.97353127598862	-1.11047078622310	-4.34567986461437
C	0.47256811071226	-2.20898136322105	-3.64234747664317
H	-1.25778157098196	-2.25585722442559	-5.77357325123945
H	-0.75895793076748	-1.63737668136765	-8.05921075652646
C	4.78526551735267	3.29455888797816	-4.90213053290073
C	4.13820905679819	3.37175002935786	-3.69525211892405
C	2.78439738085308	2.98424451444649	-3.56061591140662
C	2.10661896854320	2.47810470040570	-4.70939005990202
C	2.78669296393716	2.42978993583475	-5.94808799665309

C	4.09822863485417	2.83132603943629	-6.05007955382119
H	5.82419881533404	3.59320576109285	-4.96326465796857
H	4.67765701021620	3.72598056984780	-2.82305359803886
C	2.09872186548814	3.02764775885629	-2.31096982839861
C	0.75624888625061	2.07914535022747	-4.60393974860953
H	2.25150077578919	2.06817304448306	-6.81843579828257
H	0.22025894379434	1.78876522496041	-5.50485953698500
C	3.86313255196815	7.73057614802894	-0.92726570609002
C	4.06958609922119	6.90229023442236	0.14971891719627
C	3.68428803189896	5.54270317986890	0.10605939395420
C	3.10050802419620	5.01995202686461	-1.08815986553063
C	2.87173353483424	5.90634798302286	-2.16546865413492
C	3.23955398967158	7.22630984884042	-2.09331830938970
H	4.32157549503167	5.12312706391643	2.12625667476868
H	4.52611912099993	7.28241867490433	1.05672858677893
H	2.38743583881357	5.54033585704588	-3.06408106394620
H	3.04098656094958	7.87643863627322	-2.93636823357894
C	3.86159877326822	4.70953959593011	1.23228261790425
C	2.72368031021237	3.64271131304665	-1.11543171924134
C	-1.35857977117039	1.79280284366086	-3.32264522130541
C	-2.22735065361521	2.68511457178096	-2.66448907307146
C	-1.88455011642320	0.67861843941569	-3.98307539992286
C	-3.59011602928420	2.44087332574843	-2.65119321819312
H	-1.81081869745043	3.56192311706500	-2.18391646731039

C	-3.24354514912600	0.39369116916559	-3.93020724066181
H	-1.20969867431395	0.00731924838125	-4.50519949311522
C	-4.08862349001632	1.25775232028855	-3.24110587914420
H	-3.62949470101838	-0.50977500150435	-4.39620754044667
C	-5.88950358463949	2.26719721001691	-2.19272929624742
C	-5.50281278119422	1.13735350017766	-2.92147681918102
C	-7.17700120271325	2.37759086157337	-1.69022328378005
C	-6.41117220269033	0.10550465306514	-3.14246579931504
C	-8.10443893532496	1.34805188331153	-1.89293093859289
H	-7.45803998078172	3.26013994500342	-1.12540922697645
C	-7.69533775951640	0.22173201762714	-2.62743121583264
H	-6.12067013146075	-0.78890025792359	-3.68834473591977
H	-8.39779757500095	-0.58913496486099	-2.79757701926905
C	-4.75039174578193	3.27124989227636	-2.10118058618711
C	-5.07262443580528	4.42978601723906	-3.09537940679646
C	-4.58279982106037	3.85555155637121	-0.68415076663344
C	-4.07593081075761	5.58306061389193	-3.02055730136486
H	-6.07413774989024	4.80637436109378	-2.84192716648728
H	-5.13245903427576	4.01811753853556	-4.11028922182378
C	-3.64518875781788	5.06123405311569	-0.59522940866904
H	-5.57882826583072	4.17342810323254	-0.34416368629921
H	-4.24318527637767	3.07801128087111	0.00437782556709
C	-4.01261572719092	6.15039548360928	-1.60205449889151
H	-4.37109242832316	6.36282164120897	-3.73375290630419

H	-3.08004336290530	5.23783324125647	-3.33069563626248
H	-3.68302521019625	5.45682206412702	0.42633647489266
H	-2.60824774929479	4.74495753437582	-0.75097368525606
H	-3.29197358562798	6.97548191972851	-1.54793166742742
H	-4.99624763492521	6.56914353841631	-1.34142613978376
C	3.59149312795915	2.53147246041170	2.42440259595943
C	3.19662565035563	3.01553738391764	3.68305466903964
C	4.10462003513082	1.23604329754131	2.30413198674445
C	3.28660325799336	2.19411082803698	4.79614963883056
H	2.77469606019252	4.01187569142197	3.74192082536000
C	4.22553776935932	0.41505834169048	3.41357551522223
H	4.41812998088015	0.87285275314969	1.33353383324012
C	3.79781802974845	0.88806636309646	4.64776881708892
C	2.87695512473930	2.43457086263118	6.24836919436270
H	4.64590683353213	-0.58132329653518	3.30990355417019
C	3.72330656283333	0.20217279598262	5.92682006892480
C	3.16944669250036	1.07535513128809	6.86700173650803
C	1.38198384510710	2.80419415080114	6.41420926711665
C	3.76532199258123	3.50319229449875	6.93795666430527
C	4.07174881977279	-1.09671920217033	6.29310278109167
C	2.95904321348815	0.66238980898314	8.17515003311575
C	1.05183676173671	4.24853859195263	6.03621621123290
H	1.11883101773222	2.65360021575811	7.47117786422214
H	0.76789529298998	2.10768060241579	5.82984244392831

C	3.43512087442643	4.93509758476229	6.52136607647830
H	3.61123666759603	3.41028987754514	8.02267523560444
H	4.81876410128135	3.26525084853832	6.74461700544650
C	3.86294500648003	-1.49649514835250	7.60487641866593
H	4.49186530345084	-1.79041021482636	5.56755704253061
C	3.30082206477089	-0.63793196702696	8.56500656911223
H	2.52386506450147	1.35091332273300	8.89249602178092
C	1.95696510283144	5.24534080976308	6.76101759714232
H	0.00286864292929	4.44571995424656	6.28076991761652
H	1.13090191587978	4.38242462241146	4.95198310241209
H	4.06724836800337	5.63036495833680	7.08808116208140
H	3.68491692639862	5.08625484566806	5.46288878041863
H	4.13486367432003	-2.50763394863927	7.88835357453322
H	1.72362422126470	6.26909659507969	6.44364158837673
H	1.75496861242890	5.19511488320446	7.84176265886998
C	-3.79643216179663	-1.92726889993265	-0.99345611302135
C	-4.82454049160649	-2.68581126674175	-0.39074225704755
C	-3.65972517227133	-0.57369345290739	-0.64156396310882
C	-5.72132247076072	-2.09412787398922	0.47772267308814
H	-4.88444199371173	-3.74490254092856	-0.60740816826009
C	-4.54894366374781	0.02455410593327	0.23699874688387
H	-2.89189082845170	0.04108645104056	-1.08873044738445
C	-5.58964156385088	-0.72188252165055	0.77027473280195
C	-6.90735113500136	-2.68354834978596	1.23952666097794

H	-4.42584270010613	1.07123315978149	0.49326351851486
C	-6.65500160539412	-0.32645913696270	1.67056857937769
C	-7.43443561183067	-1.44960419773511	1.96083114739041
C	-6.47942046491839	-3.76089889064891	2.26733072693758
C	-8.00242569897715	-3.25029526632413	0.30163884972100
C	-6.98583578175863	0.91890119755501	2.19674710790058
C	-8.54141361947116	-1.34154507065472	2.79051511683199
C	-6.14017720462141	-5.11436841669520	1.64165508868143
H	-7.31529713080202	-3.90228880201352	2.96737340767254
H	-5.63606039459553	-3.37719216248803	2.85398921338707
C	-7.66282390108352	-4.61093299225399	-0.30727037058279
H	-8.92263787647681	-3.35615839792264	0.89360249449414
H	-8.21081600376150	-2.51007279626307	-0.47967453233880
C	-8.09852456135529	1.01779842860515	3.01700297249830
H	-6.38795503288172	1.79664461041001	1.96950113875049
C	-8.89173919264132	-0.09872447127464	3.33394489602485
H	-9.13832700808361	-2.22125249769428	3.00978311464324
C	-7.29047867049116	-5.62726070400020	0.77393740922930
H	-5.91643640315278	-5.83296447757027	2.43997844365830
H	-5.22651974734431	-5.03508202370995	1.03874702899661
H	-8.52440182098219	-4.97032677997373	-0.88380002727315
H	-6.83793739156808	-4.51181567016408	-1.02544095429084
H	-8.35260515061011	1.98973957192458	3.42939897344968
H	-7.02864095299840	-6.59089384471069	0.32015396275023

H	-8.16777791034330	-5.80532964383532	1.41348109307460
C	3.77705621827442	-1.19372372038809	-2.00155088998774
C	4.09703540523755	-2.19827297681350	-1.07166219359484
C	4.49270493005848	0.00923928179384	-1.99864657651508
C	5.08892442738725	-1.97779152222589	-0.13192530193712
H	3.56205452296609	-3.13856139947197	-1.11413712002983
C	5.48222727918746	0.24513047898779	-1.05339585973869
H	4.23725162980547	0.78165730007636	-2.71861696407574
C	5.76146652893729	-0.73709287456326	-0.10720520146331
C	5.61065582390510	-2.87862607217837	0.98398897385828
H	6.00054193620196	1.20073851178370	-1.03605646107988
C	6.61924089606503	-0.69552380654983	1.06940853651528
C	6.47822399220870	-1.90267095835777	1.76165496972263
C	6.54427265026410	-3.98657419664793	0.41440802749651
C	4.51435914156614	-3.51553554272020	1.86455717211276
C	7.39527463821064	0.32919977721112	1.60678817723753
C	7.08746484392202	-2.08897295848688	2.99403191126118
C	5.79063226669986	-5.12528863977807	-0.26621011661138
H	7.11505275173670	-4.39784611215669	1.25924721177226
H	7.26857802448678	-3.52044156375863	-0.26459000687274
C	3.80282312939934	-4.70421237213653	1.21685150870092
H	4.98839428412221	-3.86707003910844	2.79079914753931
H	3.79069516079412	-2.74273505358604	2.15663089185467
C	7.98953181946881	0.13845191625296	2.84529798642677

H	7.50764953822743	1.27836024986378	1.08903488889803
C	7.83674847274829	-1.05619532135821	3.57008140949136
H	6.95771098325464	-3.02860713294946	3.52162623071327
C	4.78771516380312	-5.75227257957924	0.70186169238031
H	6.50923431475919	-5.87666354154640	-0.61594953614349
H	5.26921504466036	-4.75311191867036	-1.15855305068191
H	3.12943408195618	-5.15491993980020	1.95223350386657
H	3.16056661730381	-4.37481835085975	0.39460523927486
H	8.57080366329949	0.95061550860223	3.27144157491103
H	4.24355971456410	-6.57385989087275	0.22058912428700
H	5.33452887585771	-6.18609270572671	1.55220899442158
C	-0.44191906159659	4.73791499125255	1.69258391803055
F	0.63893272063278	5.04433413782377	2.42165811597664
F	-0.16133531915843	4.96239913337435	0.40011532856354
F	-1.45438353806326	5.52587610032916	2.06300227171069
C	-1.80031740949360	-4.84081261588711	0.88329323318110
F	-2.17391112084996	-4.98530246678496	-0.38560039088898
F	-1.73624260149744	-6.03425933516392	1.46995927416167
F	-2.66773614148792	-4.06505281077376	1.52738050105034
C	4.29363456875785	9.17994284483721	-0.82960488404519
F	5.00101018725177	9.39122149641034	0.33923102933255
C	5.27746982097928	9.57944122570688	-1.96930571576851
F	5.86216876535867	10.75841925225243	-1.72336115191386
F	6.24356028753439	8.65100788485676	-2.07046757012081

F	4.64943806756643	9.66441730682529	-3.15805169250669
C	3.06563365250528	10.13213337765895	-0.73453957926255
F	3.43917021489640	11.41585760633554	-0.62354739148714
F	2.26931974427301	10.01890481091432	-1.81212551576253
F	2.34843052326035	9.81015553047031	0.35358863164108
C	4.78190373524298	2.77275307196519	-7.40123602985736
F	3.91064153733756	2.28938430628648	-8.35716523420249
C	5.18230175521621	4.19086429076197	-7.90582106351695
C	5.97385498829458	1.77123686701894	-7.40461343640854
F	5.66846822588096	4.15163604106487	-9.15359588346015
F	4.09359406970595	4.97639132462712	-7.90624145511644
F	6.10974957672075	4.75556172990172	-7.11103443866808
F	6.46751778255309	1.58804165521213	-8.63389314354155
F	6.97294493026722	2.18293949870260	-6.60487947973083
F	5.54351299431087	0.57598081076238	-6.94978861031576
C	1.66548946628450	-0.77465948918154	-9.10164229918289
F	2.96126854789272	-0.30714101807109	-9.18082717059566
C	1.64187769949918	-2.04679251321547	-10.00050466283191
C	0.78507303875812	0.37876422712568	-9.66696730221101
F	2.01142416795146	-1.76950952265125	-11.25826889209462
F	2.50243373860837	-2.94757345659195	-9.49955835502226
F	0.41948588398159	-2.60692320289145	-10.03531488415138
F	1.24855009617136	0.82732302471861	-10.83741350461646
F	-0.49127589389573	-0.01180485555059	-9.83860485452230

F	0.79202983952616	1.40829761897315	-8.79707112241264
C	-2.99647791908191	-7.39428518142286	-5.99258977292748
F	-2.03585150663428	-7.91784865779628	-6.83722005812970
C	-3.35426662634183	-8.55717310556613	-5.02224770591307
C	-4.17499977654924	-6.99876267618389	-6.92973743558934
F	-3.73688708537243	-9.65640734551207	-5.68592525754258
F	-2.26994373712053	-8.86626946526338	-4.29285032312856
F	-4.34425297568220	-8.20920173798855	-4.17936366264257
F	-4.53787263869064	-8.01562385987881	-7.72149275946224
F	-5.25624379727439	-6.60761098325281	-6.22845812252954
F	-3.79066393762250	-5.97729435917863	-7.71316073065467
C	-2.52310382042355	-3.28600322047582	6.56333448940491
H	-2.24581312273389	-4.23764717937413	6.09363956533047
H	-3.58311501597836	-3.34128188968911	6.83790373988635
H	-1.94960210933479	-3.18627142583139	7.49037748531612
C	-2.28581193861435	-2.11191628517820	5.60519488835323
C	-3.12153256859055	-2.35938260060357	4.33942509076897
H	-2.79397089960204	-3.31086912544703	3.89780226808502
C	-2.73612175830933	-0.80682691970377	6.27371047589273
H	-3.82474021045473	-0.80788694502445	6.40247123763210
H	-2.28652026565514	-0.68807352220718	7.26481627836682
H	-2.47176763205834	0.07146838170269	5.67664756835335
C	-2.98153915079144	-1.24165177363103	3.31153262226055
H	-3.40825312102379	-1.55310053995978	2.34816851631044

H	-3.57119614231841	-0.36149401954842	3.61022411343318
C	-1.54855624320143	-0.84443097499400	3.12694924263526
H	-1.32791850433039	-0.15017789510949	2.31127713763861
H	-0.63567168055828	-1.96016490266148	1.56306233252460
C	-0.54897636886756	-1.20794757309398	3.95028986059215
C	-0.78808919657475	-2.04894472468568	5.19667055244371
H	-0.48379040787914	-3.08023560950779	4.94220593275386
C	0.86655511619209	-0.78372006067113	3.68882881641868
H	0.97399493382735	-0.29562781183316	2.71897625360554
H	1.21452917584894	-0.07436168218758	4.44712662971660
H	1.55334753899335	-1.64037931193771	3.73568288555663
C	0.11913682146515	-1.58721231066733	6.34943030454247
H	0.00177679836261	-0.51611071089504	6.54415950024209
H	-0.12068785530508	-2.12487001911147	7.26981184689876
H	1.17408940573625	-1.77480683256879	6.13431657616069
O	-3.61653904574345	2.17206698124278	4.37205819889110
H	-2.76039041492820	2.47814666182874	3.98868336321406
C	-4.21059908818402	3.16028598063946	5.13228363162363
C	-5.27323036452721	2.43429515331249	6.02142027692618
C	-3.16948932676222	3.89085035388438	6.05108001782558
C	-4.91216812764816	4.20903101982404	4.19984955620261
F	-6.05061273573155	3.70224222205610	3.68485660299023
F	-4.10827700500576	4.51390413636477	3.16891341464744
F	-5.22380922726769	5.34707367655989	4.84405308552563

F	-2.48355481056634	4.81187784536845	5.34676346529807
F	-3.75759562037979	4.51788428524555	7.08522377999480
F	-2.28409716585326	3.01124442333902	6.54002880906306
F	-6.18078846698329	3.30121290319029	6.51092362798889
F	-5.92420231324045	1.50127424419886	5.31841851554509
F	-4.67964461350868	1.82293126892655	7.06168909195717
O	2.34243690628181	-4.58333288739009	4.43419498562753
H	1.60333955527802	-4.60513067748238	3.79987366279655
C	2.38241104720141	-5.74750048463778	5.18794965135012
C	2.40110816217369	-7.01298411611876	4.25945613571551
C	3.71020614202821	-5.67775596987756	6.01027453591595
C	1.14989097973344	-5.81139605203645	6.15641959768875
F	0.03847252445374	-6.10776797631228	5.44864236932416
F	0.95118728062749	-4.62614763046598	6.74161095154663
F	1.29925603099525	-6.73738859774615	7.11640587791075
F	3.59055461674452	-4.82147922561046	7.03931462027550
F	4.03337633497989	-6.88300539887799	6.50768406307032
F	4.72291544169215	-5.25103029314552	5.24436704746903
F	2.10647925920065	-8.13983113904969	4.92184490259315
F	1.49727641785354	-6.85946113683189	3.27164325228705
F	3.60491927604599	-7.16419987432697	3.68256300834387
C	3.06931933699818	-1.16032893372217	9.98470786522409
C	4.40849413772764	-1.63421147267824	10.58195905815945
H	4.84640508922415	-2.44954094972521	9.99876845398478

H	5.13162922903148	-0.81198411634655	10.60894577910061
H	4.25876998478574	-1.99686127363206	11.60551061796491
C	2.48253507003278	-0.09149098019820	10.91376913365536
H	3.15150528034187	0.77071057175808	11.01223662404520
H	1.50743619563168	0.26241124298841	10.56167490273720
H	2.33888821805888	-0.51625938983846	11.91278110376010
C	2.08497763626428	-2.34516168475251	9.92956328059291
H	1.12240506835513	-2.02644325153280	9.51536893915165
H	2.46475854751332	-3.15883434849139	9.30364133712062
H	1.91375890965762	-2.74363072281874	10.93649049517742
C	-10.10318039041180	0.08722999143320	4.24859264346706
C	-10.85285266420671	-1.22612232494823	4.50115221898924
C	-9.63478083994940	0.64412325053412	5.60726964518307
C	-11.08294702615345	1.08161393940619	3.59522036182529
H	-11.24237587007526	-1.65660652238144	3.57187466647951
H	-10.21423699460287	-1.97022878400073	4.98981094194520
H	-11.70597216695491	-1.03738518037912	5.16108590553546
H	-8.91942700812957	-0.03822602446242	6.07873027891235
H	-9.14788441240896	1.61825658428279	5.50299750025029
H	-10.49161501556931	0.76641954029857	6.27989133580300
H	-10.62309368522230	2.06223302845893	3.44030996096044
H	-11.41757046093850	0.70944253753519	2.62067661903992
H	-11.96323071918230	1.21959169215441	4.23379374282162
C	8.46671550377819	-1.17553479115965	4.95946743994794

C	9.99529435318845	-1.02452259158295	4.84031186986142
C	7.90711738454864	-0.06020686910384	5.86541287298975
C	8.16513263011452	-2.52358144172051	5.62492879067238
H	10.41452612753478	-1.81076919276084	4.20311997760230
H	10.27314158463227	-0.05813933626343	4.40887733214672
H	10.46091014225427	-1.09742996951203	5.83000202450364
H	6.81786192972491	-0.13744565871762	5.95238566830277
H	8.33726505403302	-0.14195634606274	6.87034671494777
H	8.14418484470889	0.93567191780945	5.47921625146756
H	7.08869723824834	-2.67328754365662	5.76600984498044
H	8.56263597070422	-3.36407046114899	5.04540345017967
H	8.63374065426256	-2.55199048265799	6.61395166902829
C	-9.53999711238349	1.42781640293405	-1.36618162752043
C	-10.49964483230687	1.53906719731280	-2.56679845161564
C	-9.87658473710072	0.16393654755760	-0.55420430762318
C	-9.76057807096438	2.64045607901920	-0.45316572475521
H	-10.27918253273799	2.43510265108938	-3.15713046702544
H	-10.41579341906214	0.67138080694345	-3.22913361025893
H	-11.53752858606069	1.60170762485138	-2.21873746098079
H	-9.82090132689952	-0.74273774392497	-1.16394716211499
H	-9.18962091810885	0.05137286938311	0.29082618987345
H	-10.89763308453197	0.23372768846198	-0.16093555491866
H	-9.09706561066785	2.61002413545481	0.41855763357497
H	-9.60704614659358	3.58680373364321	-0.98296789433016

H	-10.79243233574176	2.63411322759434	-0.08611011578210

References

1. Ooi, T.; Maruoka, K.; Yamamoto, H. Unprecedented Stereochemical Control in the Intramolecular Ene Reactions of δ,ε-Unsaturated Aldehydes Using Exceptionally Bulky Organoaluminum Reagents: Elucidation of the Transition State. *Tetrahedron* **1994**, *50*, 6505–6522.
2. Ramirez, A. P.; Thomas, A. M.; Woerpel, K.A. Preparation of Bicyclic 1,2,4-Trioxanes from γ,δ-Unsaturated Ketones. *Org. Lett.* **2009**, 11, 3, 507–510.
3. Davies, J.; Sheikh, N. S.; Leonori, D. Photoredox Imino Functionalizations of Olefins. *Angew. Chem. Int. Ed*. **2017**, *56*, 13361–13365.
4. Zakarian, J. E.; El-Azizi, Y.; Collins, S. K. Exploiting Quadrupolar Interactions in the Synthesis of the Macrocyclic Portion of Longithorone C. *Org. Lett.* **2008**, *10*, 2927–2930.
5. Siegel, W.; Weingarten, M.; Breuer, M.; Schelwies, M. Method for the Preparation of (3*E*,7*E*)-Homofarnesic Acid or (3*E*,7*E*)-Homofarnesic Acid Ester. WO2018154048A1, 2018.
6. Schelwies, M.; Paciello, R.; Pelzer, R.; Siegel, W.; Breuer, M. Palladium-Catalyzed Low Pressure Carbonylation of Allylic Alcohols by Catalytic Anhydride Activation. *Chem. Eur. J.* **2021**, *27*, 9263–9266.
7. Surendra, K.; Corey, E. J. Highly Enantioselective Proton-Initiated Polycyclization of Polyenes. *J. Am. Chem. Soc*. **2012**, *134*, 11992–11994.
8. Luo, N.; Turberg, M.; Leutzsch, M.; Mitschke, B.; Brunen, S.; Wakchaure, V. N.; Nöthling, N.; Schelwies, M.; Pelzer, R.; List, B. The Catalytic Asymmetric Polyene Cyclization of Homofarnesol to Ambrox. *Nature* **2024**, *632*, 795–801.
9. Burns, A. S.; Dooley, C.; Carlson, P. R.; Ziller, J. W.; Rychnovsky, S. D. Relative and Absolute Structure Assignments of Alkenes Using Crystalline Osmate Derivatives for X-Ray Analysis. *Org. Lett*. **2019**, *21*, 10125–10129.
10. Chong, B. D.; Ji, Y. I.; Oh, S. S.; Yang, J. D.; Baik, W.; Koo, S. Highly Efficient Synthesis of Methyl-Substituted Conjugate Cyclohexenones. *J. Org. Chem*. **1997**, *62*, 9323–9325.
11. Reetz, M. T.; Kindler, A. The Kharasch Reaction Revisited: CuX_3Li_2-Catalyzed Conjugate Addition Reactions of Grignard Reagents. *J. Organomet. Chem.* **1995**, *502*, C5–C7.
12. Martinez-Solorio, D.; Jennings, M. P. Convergent Formal Syntheses of (±)-Brussonol and (±)-Abrotanone via an Intramolecular Marson-Type Cyclization. *Org. Lett*. **2009**, *11*, 189–192.
13. Burés, J. A Simple Graphical Method to Determine the Order in Catalyst. *Angew. Chem. Int. Ed*. **2016**, *55*, 2028–2031.
14. Nielsen, C. D.; Burés, J. Visual Kinetic Analysis. *Chem. Sci*. **2019**, *10*, 348–353.
15. Ho, N. H.; Le Noble, W. J. Pressure Effects in a Solvolysis Involving Extended π Participation. *J. Org. Chem*. **1989**, *54*, 2018–2021.
16. Lévesque, F.; Seeberger, P. H. Highly Efficient Continuous Flow Reactions Using Singlet Oxygen as a "Green" Reagent. *Org. Lett*. **2011**, *13*, 5008–5011.
17. Kobayashi, Y.; Yoshida, S.; Nakayama, Y. Total Synthesis of Korormicin. *Eur. J. Org. Chem*. **2001**, 1873–1881.
18. Citron, C. A.; Rabe, P.; Barra, L.; Nakano, C.; Hoshino, T.; Dickschat, J. S. Synthesis of Isotopically Labelled Oligoprenyl Diphosphates and Their Application in Mechanistic Investigations of Terpene Cyclases. *Eur. J. Org. Chem*. **2014**, 7684–7691.
19. Kutateladze, D. A.; Strassfeld, D. A.; Jacobsen, E. N. Enantioselective Tail-to-Head Cyclizations Catalyzed by Dual Hydrogen-Bond Donors. *J. Am. Chem. Soc*. **2020**, *142*, 6951–6956.
20. Anderson, T. L.; Kwan, E. E. PyQuiver; GitHub Repository. **2020**. https://github.com/ekwan/PyQuiver.
21. Melnikov, S.; Chugh, V.; Hwang, D.; Baik, M.-H.; Broere, D. L. J. The Role of Quantum Tunneling in Metal–Ligand Proton Tautomerism. *J. Am. Chem. Soc*. **2025**, *147*, 46324–46335.
22. Raptis, C.; Lykakis, I. N.; Tsangarakis, C.; Stratakis, M. Acid-Catalyzed Cyclization of Terpenes under Homogeneous and Heterogeneous Conditions as Probed through Stereoisotopic Studies: A Concerted Process with Competing Preorganized Chair and Boat Transition States. *Chem. Eur. J*. **2009**, *15*, 11918–11927.

23. Maeda, S.; Harabuchi, Y.; Takagi, M.; Taketsugu, T.; Morokuma, K. Artificial Force Induced Reaction (AFIR) Method for Exploring Quantum Chemical Potential Energy Surfaces. *Chem. Rec.* **2016**, *16*, 2232–2248.
24. Maeda, S.; Ohno, K.; Morokuma, K. Systematic exploration of the mechanism of chemical reactions: the global reaction route mapping (GRRM) strategy using the ADDF and AFIR methods. *Phys. Chem. Chem. Phys.* **2013**, *15*, 3683–3701.
25. Anstine, D. M.; Zubatyuk, R.; Isayev, O. AIMNet2: A Neural Network Potential to Meet Your Neutral, Charged, Organic, and Elemental-Organic Needs. *Chem. Sci.* **2025**, *16*, 10228–10244.
26. Neese, F. Software Update: The ORCA Program System—Version 6.0. *WIREs Comput. Mol. Sci.* **2025**, *15*, e70019.
27. Froitzheim, T.; Müller, M.; Hansen, A.; et al. g-xTB: A General-Purpose Extended Tight-Binding Electronic Structure Method for the Elements H to Lr (Z = 1–103). *ChemRxiv* **2025**.
28. Grimme, S.; Hansen, A.; Ehlert, S.; Mewes, J.-M. r2SCAN-3c: A "Swiss Army Knife" Composite Electronic-Structure Method. *J. Chem. Phys.* **2021**, *154*, 064103.
29. Weigend, F.; Ahlrichs, R. Balanced Basis Sets of Split Valence, Triple Zeta Valence and Quadruple Zeta Valence Quality for H to Rn: Design and Assessment of Accuracy. *Phys. Chem. Chem. Phys.* **2005**, *7*, 3297–3305.
30. Mardirossian, N.; Head-Gordon, M. ωB97M-V: A Combinatorially Optimized, Range-Separated Hybrid, Meta-GGA Density Functional with VV10 Nonlocal Correlation. *J. Chem. Phys.* **2016**, *144*, 214110.
31. Lu, T.; Chen, Q. Independent Gradient Model Based on Hirshfeld Partition: A New Method for Visual Study of Interactions in Chemical Systems. *J. Comput. Chem.* **2022**, *43*, 539–555.
32. Lu, T.; Chen, F. Multiwfn: A Multifunctional Wavefunction Analyzer. *J. Comput. Chem.* **2012**, *33*, 580–592.
33. Meng, E. C.; Goddard, T. D.; Pettersen, E. F.; Couch, G. S.; Pearson, Z. J.; Morris, J. H.; et al. UCSF ChimeraX: Tools for Structure Building and Analysis. *Protein Sci.* **2023**, *32*, e4792.
34. Blender Foundation. *Blender*; Blender Foundation: Amsterdam, 2018.
35. Peng, Q.; Duarte, F.; Paton, R. S. Computing Organic Stereoselectivity—From Concepts to Quantitative Calculations and Predictions. *Chem. Soc. Rev.* **2016**, *45*, 6093–6107.
36. Lu, T. A Comprehensive Electron Wavefunction Analysis Toolbox for Chemists, Multiwfn. *J. Chem. Phys.* **2024**, *161*, 082503.
37. Bickelhaupt, F. M.; Houk, K. N. Analyzing Reaction Rates with the Distortion/Interaction-Activation Strain Model. *Angew. Chem., Int. Ed.* **2017**, *56*, 10070–10086.
